# Modeling glycans with AlphaFold 3: capabilities, caveats, and limitations

**DOI:** 10.1093/glycob/cwaf048

**Published:** 2025-08-28

**Authors:** Chin Huang, Natarajan Kannan, Kelley W Moremen

**Affiliations:** Department of Biochemistry and Molecular Biology, University of Georgia, 120 Green Street, Athens, GA 30602, United States; Complex Carbohydrate Research Center, University of Georgia, 315 Riverbend Road, Athens, GA 30602, United States; Department of Biochemistry and Molecular Biology, University of Georgia, 120 Green Street, Athens, GA 30602, United States; Institute of Bioinformatics, University of Georgia, 120 Green Street, Athens, GA 30602, United States; Department of Biochemistry and Molecular Biology, University of Georgia, 120 Green Street, Athens, GA 30602, United States; Complex Carbohydrate Research Center, University of Georgia, 315 Riverbend Road, Athens, GA 30602, United States

**Keywords:** AlphaFold 3, glycan modeling, glycoprotein modeling, glycan stereochemistry, glycan-protein interaction

## Abstract

Glycans are complex carbohydrates that exhibit extraordinary structural complexity and stereochemical diversity while playing essential roles in many biological processes, including immune regulation, pathogen recognition, and cell communication. In humans, more than half of all proteins are glycosylated, particularly those in secretory and membrane-associated pathways, highlighting the importance of glycans in health and disease. The recent release of the AlphaFold 3 source code enables customizable modeling not only of proteins but also glycan-containing biomolecular complexes. We assessed the capacity of AlphaFold 3 to model glycans using several input formats and identified a hybrid syntax employing Chemical Component Dictionary (CCD)-based molecular building blocks linked by “*bondedAtomPairs”* (*BAP*) as most effective in generating stereochemically valid glycan models. This workflow was used to create a library of AlphaFold 3 input templates and corresponding structural models for various glycan classes. We further explored capabilities, limitations, and remediation strategies for modeling problematic structures. Glycan interactions were also modeled with glycosylation enzymes and lectins with benchmarking and validation against known crystal structures. This protocol-driven approach is valuable for generating stereochemically valid, static models of glycan-protein interactions to support hypothesis development and subsequent structural and functional validation. However, caution should be observed in overinterpretation of the static models since glycans are known to exhibit considerable conformational dynamics that can be further captured by equilibrium sampling using molecular dynamics-based approaches. By sharing benchmarked examples using the *BAP* syntax we aim to support broader evaluation of AlphaFold 3 in studying glycan-related mechanisms in biosynthesis, signaling, infection, and disease.

## Introduction

Glycans are ubiquitous in all domains of life (Varki 2017) and play essential roles in processes such as immune modulation (Pinho et al. 2023), cancer progression (Smith and Bertozzi 2021), and neural development (Schnaar et al. 2014). Glycan structures are assemblies of monosaccharide building blocks linked through the C1 or C2 anomeric carbon in glycosidic linkages to hydroxyl groups of other monosaccharides in linear or branched configurations or to functionalities in other molecule types (Moremen and Haltiwanger 2019). These structures can be found free in solution (e.g. human milk oligosaccharides (HMOs) and hyaluronic acid (HA)), covalently linked to proteins (*N*- and *O*-linked glycans), lipids (glycosphingolipids (GSLs), dolichol phosphate (Dol-P)-linked oligosaccharides), in bridging linkages between lipids and proteins (GPI anchors) (Moremen et al. 2012), to other macromolecules (xenobiotics) (Allain et al. 2020), and numerous other classes of glycoconjugates. Glycan structures are assembled by glycosyltransferases (Moremen and Haltiwanger 2019) and deconstructed by glycoside hydrolases (Moremen et al. 1994) and lyases (Garron and Cygler 2010), and play numerous roles in modulation of function including recognition by glycan-binding proteins (lectins) (Stowell et al. 2010).

The structural complexities of glycans arise from the diversity of glycan linkages between monosaccharides, numerous possibilities for glycan branching, two possible anomeric linkages, multiple sugar ring configurations and puckering, and diverse additional modifications (Woods 2018). Rotational flexibility of glycans at the anomeric linkage also leads to conformational dynamics that present challenges in structure determination. This glycan structural diversity, complexity, and dynamics remain a formidable challenge in glycobiology research. Computational approaches, including molecular docking (Grant and Woods 2014), molecular dynamics (MD) (Case et al. 2005; Kirschner et al. 2008; Fadda 2022), and quantum mechanics/molecular mechanics (QM/MM) simulations (Mendoza and Masgrau 2021; Perez and Makshakova 2022), can generate predictive models that complement empirical structural studies. Recent developments in AI approaches, including RoseTTAFold All-Atom (Krishna et al. 2024), AlphaFold 3 (Abramson et al. 2024), DeepGlycanSite (He et al. 2024), Chai-1 (Chai Discovery et al. 2024), CLIMBS (Luo and Parmeggiani 2025), and Boltz-2 (Passaro et al. 2025) now present an additional and complementary set of in silico tools for modeling glycan-protein interaction.

In 2018, AlphaFold initially demonstrated highly accurate predictive modeling capabilities for proteins, particularly when homologous protein templates were available in the PDB (Senior et al. 2020). AlphaFold 2 expanded its capabilities to novel proteins and dimers (Jumper et al. 2021; Evans et al. 2022). AlphaFold 3 (AF3), with an updated diffusion-based generative framework, enabled the modeling of diverse biological molecules, including a growing number of bound ligands. Initially released as a web server format (https://alphafoldserver.com/), AF3 provides support for modeling multimers, a limited library of ligand structures (e.g. nucleic acids, selected metal ions, and cofactors), and post-translational modifications (PTMs) (Abramson et al. 2024). While protein glycosylation was included among PTMs, the ambiguity in specifying linkages and higher-order stereochemistry presented limitations in evaluating the application of AF3 in modeling protein-glycan interactions. AF3 was also released as a command-line interface, standalone version in late 2024 (https://github.com/google-deepmind/alphafold3) that presented opportunities for universal ligand input, but the syntax for specifying glycan stereochemistry remained a challenge. Concurrently, the Protein Data Bank (PDB) underwent an effort in carbohydrate remediation (Shao et al. 2021) (7.7% of entries contain glycan structures (Dashti et al. 2020, Prestegard 2021)). Monosaccharides have been standardized, accompanied by the removal of obsolete polysaccharide entries, implementation of consistent linkage annotations, and the integration of cross-references to external glycoinformatics resources. Although not originally intended for deep learning applications, this curated dataset now serves as a valuable resource for model training and evaluation of AF3 predictions.

Here, we investigate the input syntax for specifying glycan structures using standalone AF3 and its impact in modeling key glycan conformational features, including anomeric configurations, epimeric orientations (axial versus equatorial), and ring puckering. We compare modeling outcomes across multiple input formats and identify the *bondedAtomPairs* (*BAP*) syntax that yields the most consistent and stereochemically plausible glycan models. Fidelity of modeling is assessed through comparison with empirical structures. The *BAP* syntax framework supports the generation of starting glycan structures for downstream analysis and expands the applicability of AF3 in modeling complex macromolecules beyond glycoconjugates.

## Results

### Enhanced structural fidelity through *bondedAtomPairs* (*BAP*)-defined glycosidic linkages

Monosaccharides possess distinct structural features, including absolute configurations (D/L nomenclature), ring forms (linear, pyranose, or furanose), and anomeric centers (α/β), which pose substantial challenges in cheminformatics (Seeberger 2022). Linkage of monosaccharide building blocks into larger glycan structures presents further complications, since multiple hydroxyl positions are available for glycosidic linkages, and combined modification of multiple hydroxyls on a single monosaccharide can lead to branched structures (Lebrilla et al. 2022). AF3 generally allows ligand definition through three input formats: Simplified Molecular Input Line Entry System (SMILES) (Weininger 1988), Chemical Component Dictionary (CCD) codes (Feng et al. 2004), and user-defined CCDs (*userCCD*). Although these formats are intended to enable universal ligand input, their performance in handling stereochemically complex glycans has yet to be systematically evaluated.

**Fig. 1:**
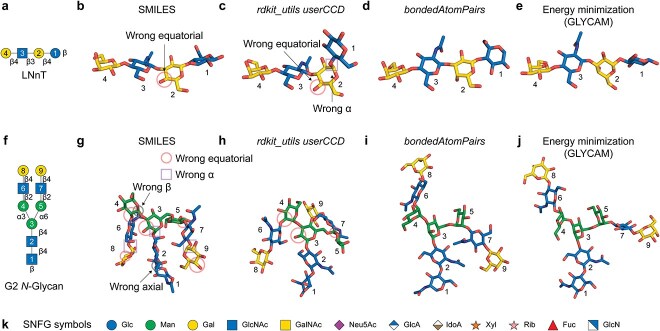
**Comparison of ligand input formats for glycan modeling in AlphaFold 3**. **a**) Symbol nomenclature for glycans (SNFG) cartoon of lacto-*N*-neotetraose (LNnT) with annotated residue numbers. **b**) AF3-modeled LNnT using simplified molecular input line entry system (SMILES) input, **c**) user-defined chemical component dictionary (*userCCD*) converted by *rdkit_utlis*, **d**) CCD with *bondedAtomPairs*, **e**) energy minimized LNnT simulated by GLYCAM. **f**) SNFG cartoon of biantennary G2 *N*-linked glycan. **g**) AF3-modeled G2 using SMILES, **h**) *userCCD*, **i**) CCD with *bondedAtomPairs*, **j**) GLYCAM-simulated energy-minimized G2. **k**) SNFG legend used in this article. Gray and pink circles, or purple square indicate wrong stereochemistry as labeled.

We first assessed the modeling quality in AF3 using a simple linear HMO, lacto-*N*-neotetraose (LNnT) (Fig. 1a). Given the simplicity of the SMILES format, we initially tested its feasibility. AF3 correctly represented the absolute configurations, ring forms, linkage order, and anomeric centers of LNnT. However, a Gal residue (residue 2) was incorrectly modeled as Glc, due to misassignment of the C4 hydroxyl from axial to equatorial (Fig. 1b). Furthermore, the SMILES input format does not support atom indexing, thereby limiting its applicability for specifying covalent linkages in multicomponent assemblies.

To circumvent this, the AF3 development team introduced *rdkit_utlis* (https://github.com/google-deepmind/alphafold3/blob/main/src/alphafold3/data/tools/rdkit_utils.py), which converts SMILES into a CCD-like format compatible with *userCCD* input. This tool generates idealized coordinates designed to enhance model performance. However, models generated with this approach still exhibited stereochemical problems. Specifically, the LNnT Gal residue 2 retained the erroneous C4 hydroxyl orientation, and the glycosidic bond between Glc and Gal (residues 1 and 2) was incorrectly rendered as an α- rather than β-linkage (Fig. 1c). Additional attempts to convert an energy-minimized PDB structure of LNnT (computed by GLYCAM (Grant et al. 2025); Fig. 1e) into *userCCD* format using *rdkit_utils* introduced further errors (data not shown). Alternative programs for CCD generation (Agirre 2017; Steiner and Tucker 2017) such as AceDRG (Long et al. 2017) yielded similarly incorrect configurations in AF3 (data not shown).

An alternative strategy for glycan structure input in AF3 is to specify individual monosaccharide building blocks in a glycan structure using their unique CCD identifiers. By leveraging the curated monosaccharide CCD library, larger glycan assemblies can be generated by explicitly defining glycosidic linkages between the building blocks using the *bondedAtomPairs* (*BAP*) syntax in the input JavaScript Object Notation (JSON) file (described in detail below). Using this approach, we were able to model the correct stereochemical structure of LNnT, including all anomeric configurations and axial/equatorial orientations (Fig. 1d).

To evaluate whether this approach generalizes to more complicated branched structures, we modeled a complex biantennary *N*-glycan G2 (Fig. 1f). SMILES input resulted in multiple structural errors: GlcNAc (residue 1) C4 hydroxyl was incorrectly modeled as axial; C2 hydroxyls of Man residues (residue 3–5) and C4 hydroxyls of Gal residues (residue 8 and 9) exhibited erroneous equatorial orientations; residues 3, 4, 8 and 9 were depicted in opposite anomeric configurations (α instead of β, or vice versa) (Fig. 1g). Models generated from *userCCD* converted through *rdkit_utils* were improved, though not flawless (Fig. 1h); of note, the C2 hydroxyls on Man residues 3–5 were still incorrect. Due to limitations in *rdkit_utils*, default parameters failed to generate correct conformers for G2; setting *useRandomCoords = True* allowed generation of idealized coordinates. Ultimately, using CCD input linked with *bondedAtomPairs* (*BAP*) enabled effective modeling of G2, recapitulating all stereochemical features correctly (Fig. 1i). These predictions were in strong agreement with energy-minimized structures simulated by GLYCAM (Grant et al. 2025) (Fig. 1j).

### Formalization of *bondedAtomPairs* (*BAP*) syntax for glycan topology specification

AF3 accepts protein sequence input in the form of a JSON file. This file also allows specification of additional inputs, including modeling random seeds, DNA, RNA, modified amino acid residues, and small-molecule ligands using SMILES, CCD, or *userCCD* formats (https://github.com/google-deepmind/alphafold3/blob/main/docs/input.md). In particular, the *bondedAtomPairs* (*BAP*) syntax can be used to define covalent linkages between distinct molecular entities; either between proteins and ligands or between individual ligand components. This syntax offers greater detail and flexibility compared to the simplified format used in the AF3 Server interface. Surprisingly, AF3 can also process input consisting solely of ligands without a protein context, which offers a convenient way to validate ligand syntax and bonding logic prior to full complex modeling.

**Fig. 2:**
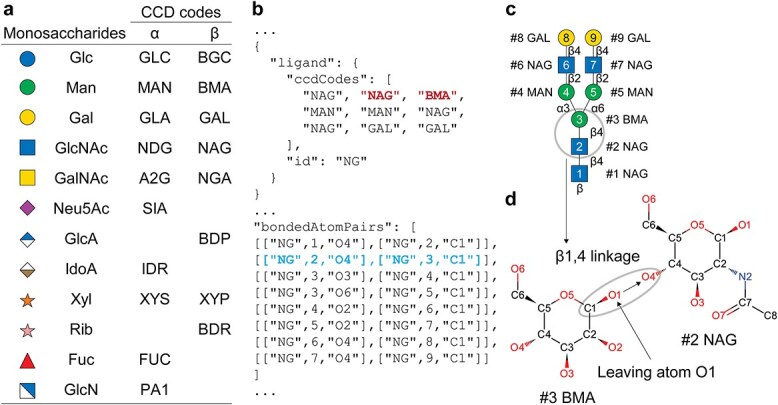
**Glycan specification using chemical component dictionary (CCD) and *bondedAtomPairs* (*BAP*) syntax in AlphaFold 3. a**) Representative CCD codes for common human monosaccharides. **b**) Partial JSON script used to generate G2 *N*-linked glycan. **c**) SNFG representation of G2 *N*-linked glycan with residue numbering equivalent to what is used in the JSON modeling input file. The order of the monosaccharides in the JSON file *ccdCodes* list corresponds with the residue numbering in the SNFG representation. **d**) Demonstration of a β1,4 linkage between GlcNAc and Man, specified by the second and third CCD entries (NAG and BMA shown in bold red font in panel **b**). The glycosidic bond is defined by the *bondedAtomPairs* (*BAP*) field (shown in bold blue font in panel **b**), specifying a bond between the O4 atom of NAG and the C1 atom of BMA (oval in panel **d**). The entry in the *bondedAtomPairs* (*BAP*) list leads to covalent bond formation between the respective hydroxyl oxygen (O4 of NAG) and the anomeric C1 (C1 of BMA) with the removal of the β-linked O1 (leaving atom) on the C1 of BMA. The resulting glycosidic linkage retains the anomeric configuration at C1 of the original CCD entry (β-linkage for BMA).

Specifying glycans through *bondedAtomPairs* (*BAP*) syntax requires the use of monosaccharide CCD codes with appropriate anomeric specification. For simplicity, a partial list of human-relevant monosaccharide CCD codes is presented in Fig. 2a. A comprehensive CCD code table, expanded to include L/D configurations, linear and cyclic (pyranose/furanose) forms, and α/β anomeric designations was compiled based on the SNFG (Varki et al. 2015) and is provided in Table S1. An example of *bondedAtomPairs* (*BAP*) syntax for the G2 *N*-glycan is illustrated in Fig. 2b. The order of the residues in the *ccdCodes* section of the JSON file corresponds directly to the residue numbering used in the *bondedAtomPairs* (*BAP*) field, as visualized in Fig. 2c. To support multiple glycan ligands or multiple glycosylation sites, unique identifiers can be defined in the *id* field; in this example, a single copy of G2 was assigned as “*NG*.”

To demonstrate bonding logic, we showcased the β1,4 linkage between chitobiose and β-Man. The CCD codes for 2-acetamido-2-deoxy-β-D-glucopyranose (NAG) and β-D-mannopyranose (BMA) were assigned as residues 2 and 3, respectively, in the *ccdCodes* section (Fig. 2b, red). A β1,4 linkage was defined by connecting the O4 atom of NAG (residue 2) with the C1 atom of BMA (residue 3) in the *bondedAtomPairs* (*BAP*) field (Fig. 2b, blue). In this case, the β-linkage is being specified by the CCD code for the β-anomer of mannose (BMA). Atom numbering for NAG and BMA is provided in Fig. 2d. By default, AF3 removes the O1 atom of the donor monosaccharide during glycosidic bond formation, as specified by the *.pdbx_leaving_atom_flag* in the official macromolecular Crystallographic Information File (mmCIF).

Extending our investigation to additional monosaccharides, we observed that not all leaving groups are automatically removed as expected. Sialic acid, specifically *N*-acetyl-α-neuraminic acid (CCD code SIA), a ketose unlike most aldose monosaccharides, links through its C2 position (Meng et al. 2013). In this case, the O2 atom of SIA is not removed by default (Fig. S1a), even when the *.pdbx_leaving_atom_flag* is correctly set. We also tested an alternative linkage by connecting the C6 atom of GAL to the O2 atom of SIA. In this configuration, both the O6 atom of GAL and the O2 atom of SIA were retained, generating unrealistic valence states and unfavorable torsion angles at the glycosidic junction (Fig. S1b). Manual deletion of leaving oxygens in the monosaccharide CCD entry, O2 in SIA (Fig. S1c and d), is necessary to maintain proper valency. The items removed were highlighted in the full standard SIA mmCIF (Supplementary Document 1). Similarly, CCDs converted into *userCCD* format without manual edits retain O1 atoms, leading to duplicate oxygen atoms at glycosidic junctions. This behavior also applies to non-carbohydrate ligands. Glycan modifications such as phosphorylation (Li et al. 2022), sulfation (Wu et al. 2023), methylation (Urbanowicz et al. 2012), and acetylation (Wang et al. 2021) follow the same principle: manual removal of leaving oxygen atoms is required to ensure chemically plausible connectivity. In light of the error-prone nature of glycan linkage specification and the advanced domain knowledge it demands, we have curated a collection of validated JSON input files encompassing all common *N*-glycans (Stanley et al. 2022) and other glycans modeled herein as reference templates for reproducible implementation (Supplementary Document 2).

### Benchmarking AlphaFold 3 for high-complexity glycan modeling

**Fig. 3:**
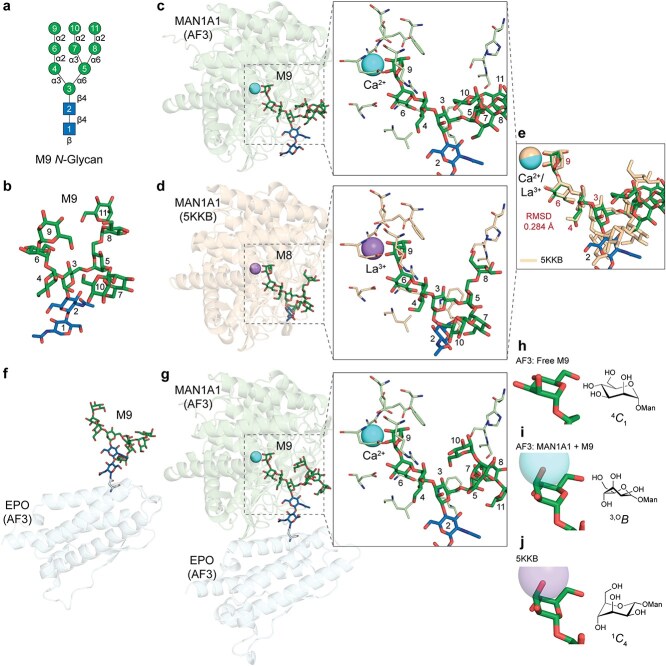
**Benchmarking AlphaFold 3 models with MAN1A1/M9/Ca**  ^**2+**^  **Michaelis complex. a**) SNFG cartoon representation of M9 *N*-glycan; residue numbers are consistently annotated across panels. **b**) AF3-modeled free-reducing end M9 glycan. **c**) Model of the *Mus musculus* Golgi mannosidase MAN1A1 bound to M9 and Ca^2+^, representing a Michaelis complex; the active site is highlighted, showing the glycan binding pose and interacting residues. **d**) Crystallographic structure of MAN1A1 in complex with M8 and La^3+^ (PDB: 5KKB). **e**) Structural alignment of the AF3 MAN1A1/M9/Ca^2+^ model (colored) with the crystallographic structure (tan); mannose residues on the 3-arm (residues 4, 6, and 9) overlay well in the catalytic pocket, while solvent-exposed residues display greater divergence from the complex in the crystal structure. The root-mean-square deviation (RMSD) of residues 3, 4, 6, and 9 (colored in red) between the AF3 model and 5KKB is annotated. **f**) M9 glycan modeled as an *N*-glycan attached to *H. sapiens* erythropoietin (EPO). **g**) MAN1A1 (green) modeled in complex with Ca^2+^ and M9 carried on EPO (cyan). **h-j**) Cremer-Pople ring puckering conformations of α1,2-mannose (residue 9) in different contexts: **h**) free-reducing end M9 (panel **b**) exhibiting a stable ^4^*C*_1_ chair; **i**) MAN1A1-M9-Ca^2+^ model (panel **c**) showing a distorted ^3,O^*B* boat; **j**) crystallographic 5KKB structure (panel **d**) adopting an unfavorable ^1^*C*_4_ chair.

After establishing the syntax for consistent glycan modeling, we benchmarked the capacity of AF3 to model glycans within glycoprotein complexes. We began with the highly branched M9 *N*-glycan (Fig. 3a). As a free-reducing end ligand, M9 was appropriately modeled with expected conformations both as a free glycan (Fig. 3b) and when bound as ligand in the active site of an α-mannosidase involved in trimming M9 to M5 during *N*-glycan maturation (MAN1A1 (Lal et al. 1994), Fig. 3c). The α1,3-Man branch of M9 (3-arm) extended deep into the catalytic pocket, with the terminal α1,2-Man glycone residue (residue 9) coordinated to an enzyme-bound Ca^2+^ ion. Notably, residue 9 adopted a distorted ^3,O^*B* boat conformation (Fig. 3i) rather than its solution-phase ^4^*C*_1_ chair ground state (Fig. 3h). Compared with the crystallographic structure (Xiang et al. 2016) (Fig. 3d), the 3-arm adopted a similar binding pose (Fig. 3e) (root-mean-square deviation (RMSD) 0.284 Å), whereas the core GlcNAc (residue 2) and α1,6-Man branch (6-arm) deviated. These latter differences from the empirical crystal structure are likely due to their solvent exposure and increased conformational flexibility. The distorted ^3,O^*B* boat conformation of the terminal Man (residue 9) in the AF3 model contrasts with an unfavorable, high-energy ^1^*C*_4_ chair conformation for the glycone in the crystal structure (Fig. 3j). Although this may initially appear inconsistent, the AF3 prediction aligns with the Cremer-Pople ring puckering itinerary (Cremer and Pople 1975) proposed for a homologous GH47 α-mannosidase (Thompson et al. 2012) where glycone conformation transitions during catalysis from ^3,O^*B*/^3^*S*_1_ through ^3^*H*_4_ intermediate *en route* to a ^1^*C*_4_ enzymatic product.

It is noteworthy that the crystal structure of MAN1A1 was solved in complex with a lanthanum ion (La^3+^) as an inhibitor (Xiang et al. 2016). However, AF3 models incorporating either Ca^2+^ or La^3+^ generated the same ^3,O^*B* structure for the glycone residue rather than ^1^*C*_4_ conformation (data not shown). This observation suggests that even if a MAN1A1 structure was included in AF3 training set, its internal representation does not rigidly duplicate the training input. Instead, AF3 accommodates alternative, yet chemically plausible, conformations including more flexible branches like the 6-arm and distinct ring-puckering states, presumably due to the diffusion-based architecture and learned representations of relevant complexes from the PDB such as the co-crystal structure of MAN1A1 with M9 and La^3+^ (Xiang et al. 2016) and glycoside hydrolase family 47 (GH47) members co-crystalized with a non-hydrolyzed thiomannobiose (Karaveg et al. 2005; Thompson et al. 2012) or glycone mimic inhibitors, 1-deoxymannojirimycin or kifunensine (Vallee et al. 2000; Lobsanov et al. 2002).

To evaluate generalizability, we modeled M9 as a PTM on erythropoietin (EPO), which features solvent-exposed *N*-glycosylation sites with minimal steric hindrance (Adams et al. 2022) (Fig. 3f). When co-modeled with MAN1A1, the 3-arm adopted a binding pose (Fig. 3g) nearly identical to the complex that was observed using the free-reducing M9 (RMSD 0.264 Å). These results highlight the capacity of AF3 to effectively model enzyme-glycoprotein complexes and recapitulate physiologically relevant intermediates.

We next evaluated a more complex case involving a glycosyltransferase bound to a divalent cation, a sugar nucleotide donor, and a glycan acceptor. The A1 *N*-glycan (Fig. S2a and b) was modeled in complex with MGAT2 (Kadirvelraj et al. 2018) (Fig. S2c), the GlcNAc transferase responsible for converting hybrid to complex *N*-glycans. The 3-arm GlcNAc (residue 6) oriented away from the catalytic pocket toward an “exosite” identified in the MGAT2 crystal structure, while the 6-arm mannose (residue 5) extended into the active site and aligned with UDP-GlcNAc, which was coordinated with a manganese ion (Mn^2+^). The modeled complex closely matched the crystallographic pose (Kadirvelraj et al. 2018), including the glycan (RMSD 0.241 Å), nucleotide, and metal ion arrangement (Fig. S2d and e). This model was also recapitulated on EPO as a glycoprotein scaffold (Fig. S2f) and in the ternary complex with MGAT2 (Fig. S2g).

To further test the performance of AF3, we benchmarked G2F, a fully extended, biantennary, core-fucosylated *N*-glycan (Fig. S3a and b), as the ligand for ST6GAL1 (Fig. S3c), an α2,6-sialyltransferase. The 3-arm of G2F showed extensive interactions within the ST6GAL1 catalytic pocket, consistent with the known glycan branch preference for the enzyme (Barb et al. 2009), while the 6-arm remained more disordered due to solvent exposure and minimal interaction with the protein surface. The modeled conformation corresponded closely to the crystallographic structure (RMSD 0.236 Å) (Kuhn et al. 2013) (Fig. S3d and e), where the G2F glycan originated from the *N*-glycosylation of a crystallographic symmetry mate. Surprisingly, AF3 was able to model this substrate interaction as a ligand despite the complex not being explicitly present in the PDB file used for training (Kuhn et al. 2013). We also placed G2F on EPO as a PTM (Fig. S3f) and modeled an equivalent glycan-protein complex with ST6GAL1 (Fig. S3g).

Across all three high-complexity scenarios, M9 with MAN1A1, A1 with MGAT2, and G2F with ST6GAL1, AF3 generated stereochemically and conformationally plausible models that were in close agreement with crystallographic data. In several cases, it also captured subtleties such as ring puckering and flexible branching, offering valuable structural insight into glycan-protein interactions.

### Modeling lipid-linked glycans for covalent glycan-lipid-protein assemblies

**Fig. 4:**
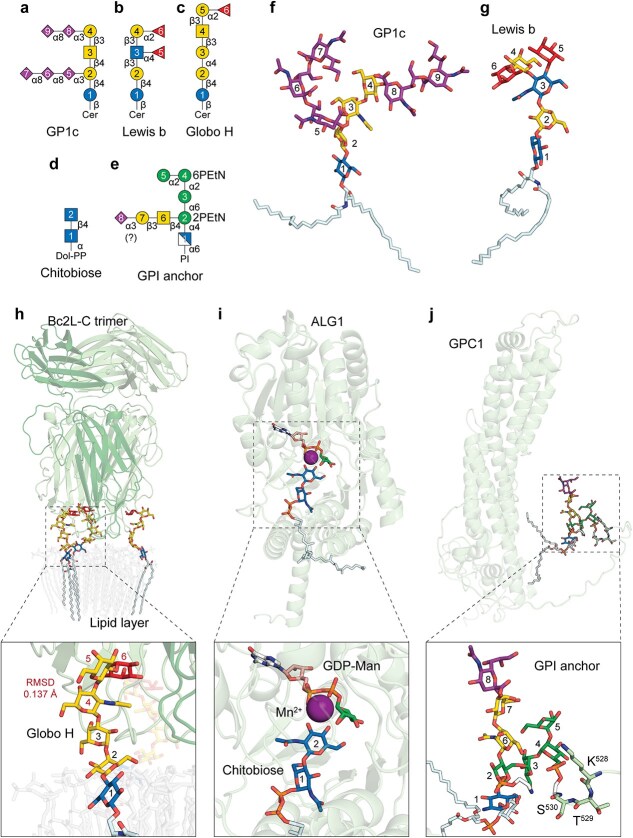
**Gallery of lipid-linked glycans modeled by AlphaFold 3. a-e**) SNFG cartoon representations of lipid-linked glycans. **f**) Ganglio-series glycosphingolipid (GSL) GP1c (panel **a**) featuring a β-linkage between Glc and ceramide (Cer). **g**) Lacto-series GSL modified with a Lewis b (Le^b^) blood group antigen (panel **b**). **h**) Globo-series GSL Globo H (panel **c**) modeled in complex with *B. cenocepacia* lectin Bc2L-C; Bc2L-C homotrimer binds to three Globo H molecules. A mock lipid layer was modeled to orient the lipid tail positioning. The root-mean-square deviation (RMSD) of residues 4–6 (colored in red) between the AF3 model and 6TIG is annotated. **i**) Dolichol pyrophosphate (dol-PP)-linked chitobiose (panel **d**) forming Michaelis complex with GDP-Man, Mn^2+^, and *H. sapiens* mannosyltransferase ALG1. Notably, chitobiose is linked to dol-PP with an α anomeric configuration. **j**) Extended glycosylphosphatidylinositol (GPI) anchor (panel **e**) linked to *H. sapiens* glypican-1 (GPC1) through phosphoethanolamine (PEtN) and the C-terminal serine (S^530^). The sialyl linkage has not been determined; an α2,3-sialic acid was modeled provisionally.

Building upon the optimized *bondedAtomPairs* (*BAP*) syntax and effective benchmarking of protein-glycan complexes, we next extended our evaluation to lipid-linked glycoconjugates. Glycosphingolipids (GSLs), composed of glycans linked to ceramide, represent a structurally diverse and biologically significant class of glycolipids (Schnaar et al. 2022). As the CCD database lacks a direct entry for ceramide, we modeled it by covalently linking sphingosine and stearic acid (CCD codes: SPH and STE). Complex GSLs such as the fully extended, sialylated ganglioside GP1c (Fig. 4a and 4f) and the highly fucosylated Lewis b antigen lactoside (Fig. 4b and 4g) were correctly modeled without notable conformational distortions. To assess receptor interactions, we modeled the globoside Globo H (Fig. 4c) in complex with Bc2L-C, a homotrimeric lectin from the opportunistic pathogen *Burkholderia cenocepacia* (Bermeo et al. 2020) (Fig. 4h). To reduce lipid tail disorder, ceramide-containing glycolipids were positioned on a mock lipid layer, resulting in more extended and physiologically plausible lipid tail conformations. Despite the inclusion of additional protein domains and full GSL components in the AF3 model, the binding pose remains consistent (RMSD 0.137 Å) with the known protein structure (PDB: 6TIG) (Bermeo et al. 2020).

We also modeled dolichol pyrophosphate (Dol-PP)-linked chitobiose, a key intermediate in *N*-glycan biosynthesis (Ramírez and Locher 2023) (Fig. 4d). Octaprenyl pyrophosphate (CCD code: OTP) was used as a Dol-PP analog. Unlike the β-linked chitobiose commonly found as a protein PTM, AF3 correctly modeled the α-linked Dol-PP glycan (Ramírez et al. 2019) (Fig. 4i). The model generated in complex with ALG1, Mn^2+^, and GDP-mannose exhibited a plausible geometry for each ligand component, with the C4 hydroxyl of the terminal GlcNAc (residue 2) properly oriented toward the donor GDP-Man.

As the most structurally complex example, we modeled a glycosylphosphatidylinositol (GPI)-anchored protein (Kinoshita 2020) using glypican-1 (GPC1) as the scaffold (Fig. 4e and 4j). The phosphatidylinositol (PI) anchor was assembled from glycerol (GOL), palmityl alcohol (PL3), stearic acid (STE), and inositol-1-phosphate (IPD) (Kinoshita and Fujita 2016). The ethanolamine phosphate moiety was built using ETA and PO4. To establish a realistic covalent linkage between the GPI anchor and the C-terminal serine (Xu et al. 2023) of GPC1, we employed the *ptmType* function to define a more plausible amide bond length. While many top-ranked models exhibited conformational artifacts, reasonable models were obtained by increasing the number of random seeds. This challenging case illustrates the capacity of AF3 to accommodate a wide range of covalent bonding topologies across lipid, glycan, and protein domains.

### Structural modeling of *O*-linked glycans in protein contexts

*O*-linked glycans attached to disordered or flexible regions of proteins pose significant obstacles for structural elucidation. Consequently, these glycoprotein architectures are underrepresented in experimental datasets and, by extension, likely underrepresented in the AF3 training set. To assess whether AF3 can generate feasible glycan conformations in such contexts, we systematically modeled several representative *O*-glycan structures.

**Fig. 5:**
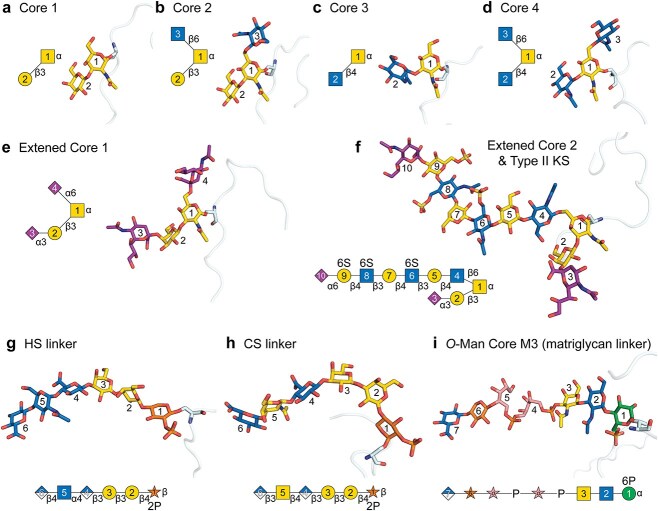
**AlphaFold 3 modeling of diverse *O*-linked glycans. a-d**) Core 1–4 *O*-GalNAc glycans modeled on *Homo sapiens* erythropoietin (EPO); only the loop regions are displayed to highlight the glycans. **e**) Extended disialyl core 1 structure on EPO. **f**) Extended core 2 glycan featuring keratan sulfate (KS) on EPO, with 6-*O*-sulfation on GlcNAc and Gal. **g**) Heparan sulfate (HS) linker modeled on *H. sapiens* glypican-1 (GPC1), including 2-*O*-phosphorylation on Xyl. **h**) Chondroitin sulfate (CS) linker modeled on *H. sapiens* bikunin, also containing 2-*O*-phosphorylation on Xyl. **i**) Extended *O*-Man Core M3 glycan modeled on *H. sapiens* α-dystroglycan with 6-*O*-phosphorylation on Man.

Four common *O*-GalNAc-linked core structures (Wandall et al. 2021), including the branched core 2 and core 4 structures, were modeled on EPO and yielded stereochemically correct conformations (Fig. 5a-d). We further extended core 1 into a common disialylated configuration (Fig. 5e) and core 2 into a glycosaminoglycan (GAG) keratan sulfate (KS) (Wu et al. 2024) (Fig. 5f). Both extensions were modeled and exhibited chemically and conformationally plausible geometries.

We next examined the *O*-Xyl linker motif that initiates heparan sulfate (HS) and chondroitin sulfate (CS) biosynthesis (Lin et al. 2024). AF3 can model HS-modified GPC1 (Fig. 5g) and CS-modified bikunin (Fig. 5h), albeit with credible conformers emerging from lower-ranked predictions, similar to the behavior observed for GPI-anchored models. Another structurally complex example was the extended *O*-Man core M3 glycan (Fig. 5i), which serves as the glycan core for matriglycan biosynthesis (Sheikh et al. 2022). This structure incorporates a non-canonical, non-cyclic moiety, ribitol phosphate, within the glycan backbone (Praissman et al. 2016). AF3 was able to resolve both the linkages and overall topology of this structure.

Collectively, these findings suggest that AF3 can be a valuable tool in generating plausible conformations of *O*-linked glycans, despite the limited representation of such structures in the training set, particularly when supplemented by input optimization and increased sampling.

### Accurate syntax enables modeling of biologically complex glycoprotein architectures

**Fig. 6:**
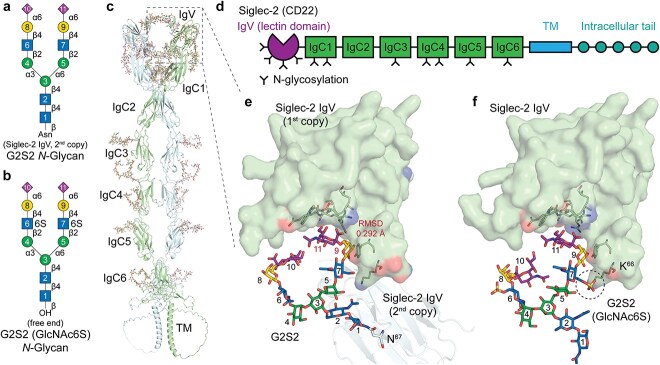
**Cis- and trans-interactions between fully *N*-glycosylated Siglec-2 (CD22) molecules revealed by AlphaFold 3. a**) SNFG cartoon representations of G2S2 *N*-glycan and **b**) G2S2 carrying a 6-*O*-sulfated GlcNAc (GlcNAc6S) modification. **c**) AF3 model of two fully *N*-glycosylated *H. sapiens* Siglec-2 molecules; one chain is colored green and the second cyan. Domains on the green chain are annotated. **d**) Domain architecture of Siglec-2, comprising an N-terminal immunoglobulin variable (IgV)-like lectin domain, six immunoglobulin constant (IgC)-like domains, a transmembrane region (TM), and intracellular signaling motifs. *N*-glycosylation sites on each domain are indicated. **e**) Close-up of the cis-interaction captured by AF3: The IgV domain one Siglec-2 chain (green, surface representation) engages the G2S2 *N*-glycan from the adjoining Siglec-2 chain (cyan, cartoon representation), with interacting amino acids shown in line and the glycan shown as sticks with SNFG coloring for the respective monosaccharides. The root-mean-square deviation (RMSD) of residues 9 and 11 (colored in red) between the AF3 model and 5VKM is annotated. **f**) AF3 model of two Siglec-2 molecules in the presence of a free-reducing end G2S2 (GlcNAc6S) glycan mimicking trans-ligand binding. The original cis-interaction is disrupted and replaced by binding to the free G2S2 (GlcNAc6S) glycan. An additional electrostatic interaction between lysine (Lys66) and the sulfate group suggests enhanced affinity relative to the cis-bound G2S2.

AF3 can model protein-glycan complexes with low computational cost and rapid turnaround, making it feasible to investigate larger and more intricate glycoprotein assemblies. To demonstrate its broader applicability, we modeled CD22, also known as sialic acid-binding immunoglobulin-like lectin 2 (Siglec-2), a transmembrane lectin comprising six immunoglobulin constant (IgC)-like domains and a membrane-distal immunoglobulin variable (IgV)-like lectin domain that projects beyond the cell-surface glycocalyx (Macauley et al. 2014; Duan and Paulson 2020) (Fig. 6d). Siglec-2 contains four *N*-glycosylation sites on the IgV domain and seven additional sites distributed across the IgC domains (Wasim et al. 2019). While the IgV domain mediates recognition of α2,6-sialylated glycans to activate downstream intracellular signaling (Duan and Paulson 2020), its binding pocket is frequently masked by cis-interactions with α2,6-sialylated glycans on adjacent Siglec-2 molecules (Razi and Varki 1998; Collins et al. 2004; Macauley et al. 2015; Wasim et al. 2019). This masking can be disrupted upon encountering high-avidity or high-affinity ligands, such as 6-O-sulfo-6′-sialyl LacNAc (Collins et al. 2006; Macauley et al. 2015; Wu et al. 2023; Ma et al. 2024; Jung et al. 2025).

To investigate this behavior, we modeled two Siglec-2 molecules, each fully modified with eleven G2S2 *N*-glycans (Fig. 6a). The resulting structure (Fig. 6c) recapitulated features consistent with prior negative-stain electron microscopy (EM) and small-angle X-ray scattering (SAXS) studies (Ereño-Orbea et al. 2017), including a ~ 120° angular displacement between the IgV-IgC2 and IgC3-IgC6 domains. The C-terminal intracellular tails were erroneously folded back toward IgC6, an artifact resulting from the lack of an explicit membrane boundary for the model.

The two IgV lectin domains displayed cis-interactions in which G2S2 glycans at Asn67 of one Siglec-2 interacted with the opposing IgV domain (Fig. 6e). The glycan-lectin binding pose aligned closely with the crystallographic structure (RMSD 0.292 Å, PDB: 5VKM); notably, the crystal structure includes only a terminal disaccharide and comprises just the IgV-IgC2 domains, with five *N*-glycosylation sites mutated to alanine, thereby precluding cis-interaction (Ereño-Orbea et al. 2017). To probe trans-interactions, we introduced a free-reducing G2S2 glycan carrying GlcNAc-6-*O*-sulfate (Fig. 6b), which mimics a high-affinity trans-ligand. In this context, the cis-interaction was disrupted, and Lys66 was positioned in proximity to the 6-*O*-sulfate group on GlcNAc, suggesting an electrostatic interaction for the free G2S2 ligand (Fig. 6f). Although further empirical validation is required to confirm the binding mechanism, it is remarkable that AF3 captured such a nuanced glycan-glycoprotein interaction within a highly complex system. This example highlights the potential of AF3 to explore structural hypotheses involving post-translational modifications and dynamic receptor-ligand switching.

### AlphaFold 3 models glycan complexes not present in the training data set

The example complexes above were all compared with protein crystal structures that were deposited in PDB prior to the cutoff date used in AF3 training and could potentially be viewed as directly replicating data used in its training. To determine if AF3 could effectively model glycan-protein complexes not present in its training data set, we examined protein-glycan complex structures in PDB that were released after 2023 January 13, since the cutoff date for the PDB training dataset of AF3 was 2021 September 30, with the additional validation dataset extending to 2023 January 12 (Abramson et al. 2024). To evaluate if glycan-models guided by the *bondedAtomPairs* (*BAP*) syntax could recapitulate the respective crystal structures of protein-glycan complexes, we used three criteria to identify potential candidates for testing our modeling strategy. The protein structure should contain a bound disaccharide or larger, the protein structure should not contain a close homolog containing a bound ligand deposited before 2023 January 13, and the modeled glycan should have appropriate stereochemistry for all carbohydrate structural components. Four example complexes were chosen: *Homo sapiens* B3GALT5 (Lo et al. 2025), *Pasteurella multocida* heparosan synthase 2 (PmHS2) (Stancanelli et al. 2024), *H. sapiens* aggrecan (Otsuka et al. 2025), and *Cucumis sativus* phloem protein 2 (PP2) (Bobbili et al. 2023).

The human β-galactosyltransferase, B3GALT5, involved in core 3 *O*-glycan synthesis and β1,3-galactosylation of glyco-lipids, was co-crystalized with the GlcNAc-β1,3-GalNAc-Thr Core 3 *O*-GalNAc disaccharide, UDP, and Mn^2+^ to obtain the ligand-bound crystal structure (8ZX3 (Lo et al. 2025), PDB release: 2025 April 9; resolution 2.09 Å, Fig. S4a and b). The corresponding AF3 Michaelis complex model (Fig. S4c) recapitulated the binding pose of the terminal GlcNAc with close agreement to the crystal structure (RMSD: 0.096 Å; Fig. S4d).

PmHS2 is a dual-domain glycosyltransferase that forms a homodimer, catalyzing the transfer of GlcNAc and GlcA to form a (-4-GlcA-β1,4-GlcNAc-α-)_n_ heparosan polymer. The structure of PmHS2 was solved by cryo-EM with bound 2-*O*-sulfated HS 5-mer, UDP, and Mn^2+^ (8VIW (Stancanelli et al. 2024), PDB release 2024 July 24, resolution 3.30 Å, Fig. S5a and b). AF3 modeled the dimeric complex with UDP-GlcNAc, Mn^2+^, and HS as a Michaelis complex (Fig. S5c) showing good agreement with the empirical structure (RMSD: 0.360 Å; Fig. S5d).

The G1 domain of aggrecan shows hyaluronan (HA) binding activity and the crystal structure of the protein complex with bound HA 10-mer was determined (9DFF (Otsuka et al. 2025), PDB release 2025 April 30, resolution 2.59 Å, Fig. S6a and b). The non-reducing end unsaturated GlcA, a lyase-derived product, was excluded from the comparison. The AF3 model recapitulated the observed binding pose for the remainder of the bound HA product with good agreement (RMSD: 0.700 Å; Fig. S6c and d).

PP2 is a plant lectin recognizing GlcNAc moiety on a glycan chain. The structure of chitotriose-bound PP2 was characterized (7W4B (Bobbili et al. 2023), PDB release 2023 March 8, resolution 2.50 Å, Fig. S7a and b). The AF3 model replicated the binding pose with high accuracy (RMSD: 0.252 Å; Fig. S7c and d).

We further broadened our filtering criteria to include newly released protein structures that contain homologous sequences or are complexed with ligands not represented in the AlphaFold 3 training set. Examples include *Bacteroides thetaiotamicron* endoglycosidase BT1285 (Sastre et al. 2024), *Bacteroides ovatus* polysaccharide lyase family 38 (BoPL38), *Pedobacter terrae* glucanase (Tandrup et al. 2025), *Bacillus circulans* xylanase (PDB: 7WWC), *Escherichia coli* fimbrial adhesin FimH (Krammer et al. 2023), and botulinum neurotoxin A (PDB: 8RVG).

The endoglycosidase BT1285 was crystallized with an M9 *N*-glycan (8U48 (Sastre et al. 2024), PDB release 2024 May 29, resolution 1.90 Å, Fig. S8a and b), showing extensive interaction with glycan residues 1 through 7, where the cleavage site is between residues 1 and 2. AF3 reproduced the binding pose with high accuracy (RMSD: 0.324 Å; Fig. S8c and d).

The crystal structure of BoPL38 was determined with a bound alginate substrate (-4-D-mannuronic acid (ManA)-β-)_4_ (9FHU (Tandrup et al. 2025), PDB release 2025 July 9, resolution 2.09 Å, Fig. S9a and b). We modeled BoPL38 with the same alginate 4-mer (Fig. S9b) and the AF3 model (Fig. S9c) aligns well with the binding pose of residues 2 to 4 within the active site, where the cleavage occurs between residues 3 and 4 (RMSD: 0.261 Å; Fig. S9d).

*P. terrae* glucanase was crystallized with laminarin (7WWC, PDB release 2023 February 15, resolution 2.20 Å, Fig. S10a), revealing a cleavage site between glycan residues 2 and 3 (Fig. S10b). As no accompanying publication was available, we highlighted side chains for potential protein-glycan interactions within 4 Å. The AF3 model demonstrated good agreement with the crystal structure (RMSD 0.214 Å; Fig. S10c and d).

The catalytically inactive *B. circulans* xylanase was co-crystallized with xylotriose (8QXY, PDB release 2024 August 21, resolution 1.41 Å, Fig. S11a and b). Extensive contacts were observed between the enzyme and all three glycan residues. AF3 successfully reproduced the binding configuration (RMSD 0.386 Å; Fig. S11c and d).

The crystal structure of *E. coli* fimbrial adhesin FimH-M3F *N*-glycan complex was characterized (7BHD (Krammer et al. 2023), PDB release 2022 July 20, resolution 1.40 Å, Fig. S12a and b**)** showing specific interactions with the 3-arm of the glycan, while the solvent-exposed core and fucose residues remained flexible. The AF3 model reflected this binding pose well (RMSD 0.189 Å; Fig. S12c and d).

Botulinum neurotoxin A (BoNT/A) mutant was crystallized in complex with GM1b ganglioside (8RVG, PDB release 2025 February 12, resolution 1.9 Å, Fig. S13a and b), highlighting interactions with residues near the reducing end. Amino acid residues within 4 Å of the glycan were used for comparison. The AF3 model reproduced the binding pose closely (RMSD 0.223 Å; Fig. S13c and d).

Together, these examples span diverse protein classes, including glycosyltransferases, glycoside hydrolases, lectins, and polysaccharide lyases. In each case, the corresponding modeled complex closely matches the bound pose of the glycan ligand observed in the crystal structure, despite these specific complexes not being present in the AF3 training set. These results suggest a broader applicability of AF3 in modeling glycan-protein complexes than previously anticipated. Notably, the modeling of complex polysaccharides such as heparan sulfate (HS), hyaluronic acid (HA), and alginate highlights the capability of AF3 to model such challenging polymer ligands when bound to enzymes and lectins.

However, we emphasize that glycan structure modeling in AF3 is highly context dependent. We observed numerous instances where modeled glycan structures did not maintain appropriate glycan stereochemistry, or the modeled complexes failed to replicate interactions present in the corresponding crystal structures. Therefore, while this approach offers a valuable tool for hypothesis generation in biologically relevant glycan-protein interactions, each model must be carefully evaluated, and appropriate structural and functional validation remains essential.

## Discussion

AF3 presents new opportunities for ligand modeling, and our evaluation across multiple glycan classes demonstrates its potential as a powerful tool for developing structural hypotheses that can be further tested empirically. While our study focused on human glycans, the modeling strategies, input syntax, and curated templates provided here can be generalized to glycan systems across all domains of life. Notably, we present pre-built input files for structurally complex ligands, including GPI anchors, featuring covalent linkages between glycan, lipid, and protein components.

Among the available ligand input formats, SMILES is the most straightforward, requiring minimal pre- or post-processing. However, when applied to stereochemically complex molecules such as glycans, SMILES frequently fails to reproduce correct conformations, even for relatively simple linear oligosaccharides like LNnT. Converting SMILES or structural files into *userCCD* using tools such as *rdkit_utils* offers more control over conformer generation and optimization, but this approach also lacks reliability in glycan modeling. This may be attributed to the reliance of AF3 on RDKit (https://www.rdkit.org) for internal ligand handling, which struggles with the conformational diversity and stereochemical precision required for glycans.

By contrast, idealized coordinates (Westbrook et al. 2015) generated through proprietary cheminformatics engines such as Corina (Molecular Networks) (Gasteiger et al. 1990; Sadowski et al. 1994) or OMEGA (OpenEye) (Hawkins et al. 2010; Hawkins and Nicholls 2012), are routinely used in novel PDB ligand deposition and exhibit higher structural fidelity. These coordinates are incorporated into CCD entries that undergo rigorous biocuration under the Worldwide Protein Data Bank (wwPDB) (Young et al. 2018), ensuring consistency and accuracy. Accordingly, idealized CCD input for monosaccharide building blocks with appropriate anomeric configurations coupled with *bondedAtomPairs* (*BAP*) syntax yielded the most reliable and stereochemically valid glycan conformations in our evaluation of structural models.

### Caveats and limitations

The use of *bondedAtomPairs* (*BAP*) syntax requires both glycobiological expertise to define the correct glycan types and linkages and a strong foundation in glycochemistry to interpret conformational plausibility. We addressed the former by compiling input templates for common glycan motifs, and the latter by systematically evaluating the structural plausibility of modeled glycans presented in this study. Analytic tools such as GlyProbity (Woods 2019) and Privateer (Dialpuri et al. 2023) are available for assessing ring puckering and glycosidic torsions based on CCD identifiers, which may increase the throughput of model quality assessment. However, these tools were originally developed for validating models derived from X-ray crystallography or cryo-electron microscopy, and their applicability to predictions from AF3 remains to be fully validated. A more thorough evaluation across diverse monosaccharides and glycan types is warranted, especially with an emphasis on comparison with empirically derived structures to validate the computational models. We anticipate that AF3 will expand access to modeling of glycan-protein complexes and generation of structural hypotheses for biological function. However, like any modeling approach, extreme care must be taken in overinterpretation of modeling results and all models should be further tested and validated by direct empirical experimentation.

If a glycan is modeled into an incorrect conformation, even a single anomeric or epimeric misassignment can significantly impact the interpretation of structural and biological function. However, AF3 currently lacks discernible metrics to penalize such conformational issues in glycan models. The predicted local-distance difference test (pLDDT) (Mariani et al. 2013), commonly used to assess protein confidence, is unreliable for ligands. We observed instances in which glycans displayed incorrect linkages, epimers, or conformations while retaining high pLDDT scores, particularly when located in catalytic pockets. Conversely, when glycans extend into solvent as post-translational modifications, pLDDT scores often decrease with distance from the protein core, even when conformations are plausible (Fig. S14**)**. Other AF3 scoring metrics such as predicted aligned error (PAE) (Evans et al. 2022), predicted template modeling (pTM) (Zhang and Skolnick 2004), interface PTM (ipTM) (Evans et al. 2022), and the sample ranking score (Abramson et al. 2024) also fail to capture glycan-specific conformational errors. RMSD comparisons with crystallographic or energy-minimized structures are similarly insufficient, as glycan flexibility can obscure subtle but critical deviations. Although glycosidic torsion angles offer more direct insight, comprehensive reference data exist primarily for *N*-glycans (e.g. GlyProbity and Privateer), limiting broader evaluation of glycan model accuracy.

We also observed that certain glycan linkages, particularly those underrepresented in the PDB or the AF3 training set, required higher sampling (i.e. more seeds) to recover conformationally plausible models. Interestingly, false conformations were often found among top sample ranking score models, whereas effective geometries frequently appeared in lower-ranked outputs. It is unclear how chirality penalties are enforced in AF3, especially for stereochemically complicated glycans. As such, we recommend evaluating both high- and low-scoring models to identify structurally plausible predictions when encountering difficulties. This approach proved useful in modeling GPI anchors and HS/CS linkers. However, when models were extended for HS or CS polymers beyond the linker region, no models with correct configurations were recovered. Common artifacts included incorrect anomeric configurations, misassigned epimers, and high-energy ring conformations (data not shown). In many cases, only the regions of HS/CS chains directly interacting with target proteins yielded plausible structures. Modeling specific degrees of polymerization may mitigate this issue.

In the case of matriglycan, which consists of [Xyl-α1,3-GlcA-β1,3] disaccharide repeats (Sheikh et al. 2022), AF3 consistently misassigns α-xylose as β, even in contexts where structures of matriglycan-bound proteins exist in the PDB (e.g. laminin (Briggs et al. 2016), Lassa virus glycoprotein (Katz et al. 2022)). Given the limited natural occurrence and greater conformational diversity of α-xylose compared to β-xylose, it may be beneficial to test SMILES or *userCCD* input formats when ambiguity in stereochemical encoding exists. While idealized coordinates in the CCD offer necessary flexibility to accommodate electron density maps, this same flexibility can be a double-edged sword for AF3. A notable advantage of this adaptability was observed when modeling glycone interactions of an M9 glycan substrate with MAN1A1, where the bound α-Man residue appropriately adopted a high-energy transition state conformation. Such conformational plasticity offers opportunities for mechanistic insights into enzyme catalysis. Nonetheless, this conformational freedom can also obscure critical features, such as incorrect assignment of anomeric configurations and epimeric orientations, thereby complicating the accurate prediction of glycan structures.

We also attempted to model a more complex case of glycoenzyme interactions with the conserved Fc *N*-glycan on immunoglobulin G (IgG). There are numerous structures in PDB of IgG or Fc domains containing complex type biantennary *N*-glycan structures tightly packed between Fc chains, effectively inaccessible to glycan-processing enzymes. Attempts to model glycan processing enzymes such as MGAT1, MAN2A1, MGAT2, FUT8, and B4GALT1 in complex with the Fc domain *N*-glycan structure were unsuccessful. The positions of the glycan structures remained consistent with crystallographic observations within the interior of the Fc homodimer core. In each case, AF3 prioritized interactions between the glycan processing enzymes and the Fc polypeptide rather than the *N*-glycan structures (data not shown). This suggests that the AF3 modeling was strongly biased by the abundant Fc domain crystal structures in the PDB training set. As a result, AF3 presently lacks the capacity to capture the dynamic and transient positioning of glycans and glycan-bearing C’E loops in the Fc domain (Borrok et al. 2012), a feature that has been experimentally validated by nuclear magnetic resonance (NMR) (Barb and Prestegard 2011) and in IgG Fc-endoglycosidase S (EndoS) co-crystal structures (Trastoy et al. 2018). Efforts to model an IgG Fc-endoglycosidase S (EndoS) complex with the glycan positioned in the active site were also unsuccessful, despite this complex presumably being present in the training dataset for modeling. In contrast, AF3 was able to recapitulate key aspects of the interaction between glycosylated IgG Fc and glycosylated FcγRIIIa (CD16a) (Falconer et al. 2018) (data not shown), consistent with known binding modes. These observations underscore the importance of critically evaluating AF3-generated models in a context-dependent manner.

AF3 does not incorporate an explicit energy function; therefore, the generated models do not represent thermodynamic equilibria nor do they explicitly represent energetically favored conformations. Furthermore, the predicted structures should be interpreted only as static snapshots, similar to those obtained from empirical methods such as X-ray crystallography, which contribute substantially to the PDB dataset used in AF3 training. The AF3 models of bound glycans commonly deviate from the equivalent crystal structure complexes when the glycan termini extend into solvent. These differences in modeled conformation correlate with lower pLDDT scores for the modeled ligands and higher B-factors for the same regions of the ligand in the crystal structures indicating greater disorder for solvent oriented ligand subregions (Fig. S14**)**. Users interested in glycan dynamics or equilibrium conformational sampling are advised to employ complementary computational methods, such as molecular dynamics simulations or ensemble-based tools like GlycoShield (Tsai et al. 2024) or GlycoShape (Ives et al. 2024), which provide structural representations grounded in exhaustive conformational sampling.

### Unanticipated and versatile applications in glycoscience and beyond

The relative scarcity of glycan structural data in the PDB, compared to protein structural data, would seemingly pose a challenge when training AF3 for glycan structural modeling. Thus, it would be reasonable to assume that effective glycan modeling with AF3 would be limited to “pre-trained” structures or simple models constructed from the fragmentary glycan information available in the PDB. Surprisingly, this assumption appears to be incorrect. AF3 is capable of modeling a wide range of glycan-binding proteins, including glycosyltransferases, glycoside hydrolases, lectins, and polysaccharide lyases, containing bound glycan structures that closely resemble crystallographic complexes not included in its training set. AF3 has clearly “learned” to generalize its predictions and generate plausible glycan models that extend well beyond the scope of its original training data. It remains unclear whether this capability stems from the structural constraints embedded in the monosaccharide building block CCD files, the partial glycan structures in the training data, or from patterns derived from non-carbohydrate chemical moieties included in its datasets. While this ability may seem counterintuitive given the limited scale of glycan training data, the resulting models offer a powerful tool for hypothesis generation.

Our investigations underscore the broad applicability of AF3 in modeling glycans, both as post-translational modifications and as free ligands. This capability opens new pathways for translational glycobiology applications. For instance, Bc2L-C, a dual-domain lectin from *B. cenocepacia*, can be modeled in complex with both glycolipid and glycoprotein ligands to better understand its multivalent binding and role in infection (Sulák et al. 2011). Similarly, AF3-generated models of cis-interactions between CD22 molecules may offer insights into dynamic regulatory mechanisms that are difficult to capture through crystallography alone.

Although further improvements in accuracy are needed for antibody–antigen modeling, AF3 shows promise in the structural prediction of anti-glycan antibodies and glycan-containing epitopes. Given that most biologics are glycoproteins targeting glycoprotein antigens in vivo, AF3 offers a valuable framework for evaluating both the structural consequences of glycoform variation and the functional effects of glycosylation on therapeutic efficacy.

We have also noted that the AF3 modeling approach could be extended beyond glycoconjugates to other complex molecule types. This is made possible by the use of other molecular building blocks encoded with idealized CCD codes and linked via *bondedAtomPairs* (*BAP*) syntax. The simplicity of the JSON scripting strategy for input into AF3 enables an accessible, flexible, and universal workflow for assembling stereochemically complex macromolecules from the molecular elements found in the CCD library. Consequently, this approach significantly broadens future capabilities for modeling biologically important and structurally complex molecular interactions.

In conclusion, although structural validation using orthogonal methods remains essential, AF3 presents a transformative platform for modeling glycan-mediated interactions across proteins, lipids, and carbohydrates. With thoughtful syntax planning and critical evaluation, AF3 has the potential to dramatically streamline and accelerate research in the field of glycoscience.

## Methods

### AlphaFold 3 version

AlphaFold 3 Version 3.0.1 (available at https://github.com/google-deepmind/alphafold3) was run as a Singularity container on the Sapelo2 high-performance computing cluster at the Georgia Advanced Computing Resource Center (GACRC), University of Georgia. Modeling was performed using NVIDIA A100 or H100 GPUs. The AlphaFold 3 model parameter permission file was granted by the DeepMind team.

### AlphaFold 3 input JSON file

A full description of the input JSON file structure is available on AlphaFold 3 GitHub repository (https://github.com/google-deepmind/alphafold3/blob/main/docs/input.md). The initial JSON template used in this study was adapted from AFusion (https://github.com/Hanziwww/AlphaFold3-GUI). All modeling was conducted using version 2 of the JSON input format.

### SMILES input and glycan energy minimization

Energy minimized glycan PDBs were generated using GLYCAM Carbohydrate Builder web tool (https://glycam.org/) (Grant et al. 2025) and subsequently converted to SMILES format through Open Babel web-based converter (https://www.cheminfo.org/Chemistry/Cheminformatics/FormatConverter/index.html) (O'Boyle et al. 2011).

### Conversion of SMILES and PDB to *userCCD* format

The *rdkit_utils* Python script from the AlphaFold 3 repository (https://github.com/google-deepmind/alphafold3/blob/main/src/alphafold3/data/tools/rdkit_utils.py) was used to convert SMILES strings or PDB files into CCD-like mmCIF files for use as *userCCD* inputs. *rdkit_utils* depends on RDKit (RDKit: Open-source cheminformatics. https://www.rdkit.org. DOI: 10.5281/zenodo.15286010).

### C‌CD library for AlphaFold 3 input

Chemical Component Dictionary (CCD) codes and associated mmCIF files were retrieved and manually curated from PDBeChem database (https://www.ebi.ac.uk/pdbe-srv/pdbechem/) for compatibility with AlphaFold 3 input requirements.

### Protein sequences for AlphaFold 3 input

Protein sequences were obtained from UniProt (https://www.uniprot.org/) using the following accession numbers: *M. musculus* MAN1A1 (P45700), *H. sapiens* MGAT2 (Q10469), *H. sapiens* ST6GAL1 (P15907), *H. sapiens* EPO (P01588), *B. cenocepacia* Bc2L-C (B4EH86), *H. sapiens* ALG1 (Q9BT22), *H. sapiens* GPC1 (P35052), *H. sapiens* Bikunin (P02760), *H. sapiens* α-dystroglycan (Q14118), *H. sapiens* Siglec-2 (P20273), *H. sapiens* B3GALT5 (Q9Y2C3), *P. multocida* heparosan synthase 2 (Q5SGE1), *H. sapiens* aggrecan (P16112), *C. sativus* phloem protein 2 (Q8LK69), *B. thetaiotamicron* BT1258 (Q8A889), *B. ovatus* polysaccharide lyase family 38 (A0A5M5BWR5), *P. terrae* glucanase (A0A1G7XNR7), *B. circulans* xylanase (P09850), *E. coli* fimbrial adhesin FimH (P08191), and *C. botulinum* neurotoxin A (P0DPI0).

### Glycan and protein visualization

Glycan cartoon representations were generated using GlycoWorkbench 2.1 (stable build 146) (Ceroni et al. 2008), with custom modifications. Protein structures were visualized, aligned and rendered using PyMOL version 3.1.3.1. Root-mean-square deviation (RMSD) was calculated by PyMol *align* function.

## Supplementary Material

Supplementary_Figures_cwaf048

Supplementary_Table_I_cwaf048

Supplementary_Document_1_cwaf048

Supplementary_Document_2_cwaf048

## Data Availability

All JSON input files and *userCCD* examples used in this study are provided in the Supplementary Document 2. All AF3 models and metadata are also available from ModelArchive using the following link: https://www.modelarchive.org/doi/10.5452/ma-af3glycan. Availability of predicted structural models is subject to the AlphaFold 3 Output Terms of Use (https://github.com/google-deepmind/alphafold3/blob/main/OUTPUT_TERMS_OF_USE.md).
