# Supplementary material for: Modeling glycans with AlphaFold 3: capabilities, caveats, and limitations
Source: Glycobiology. 2025 Aug 28;35(10):cwaf048. doi: 10.1093/glycob/cwaf048 (PMC12448869; doi:10.1093/glycob/cwaf048)
Supplement: Supplementary_Figures_cwaf048 [file supplementary_figures_cwaf048.pdf]

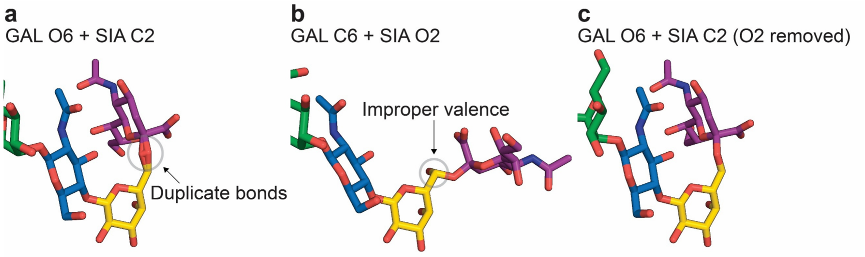

d

N-acetyl-α-neuraminic acid mmCIF file (CCD code: SIA)

data_SIA
#
_chem_comp.id SIA
_chem_comp.name 'N-acetyl-alpha-neuraminic acid'
_chem_comp.type 'D-saccharide, alpha linking'
...
~~SIA O2 O2 O 0 1 N Y N N N N -1.768 59.214 -7.992 -3.312 1.416 -0.003 O2 SIA 15~~
...
~~SIA HO2 HO2 H 0 1 N Y N N N N -0.905 59.113 -8.438 -3.519 1.217 -0.926 HO2 SIA 36~~
...
~~SIA C2 O2 SING N N 5~~
...
~~SIA O2 HO2 SING N N 36~~
...
~~SIA O2 O 7.154 -3.375 15~~
...
~~SIA C2 O2 SINGLE BEGINDASH 5~~
...
~~SIA O2 F2 1~~
...
~~SIA O2 -0.487 3.469 1.305 ETKDGv3 15~~
...
~~SIA HO2 -1.104 4.204 1.047 ETKDGv3 36~~
...

The complete JSON file is present in the supplement with the deleted lines highlighted

Sup. Fig. 1. Correcting valence errors in *N*-acetyl-α-neuraminic acid (Sia) using *userCCD*. a, Standard *bondedAtomPairs* syntax connecting O6 atom of Gal to C2 atom of Sia produces unrealistic duplicate bonds between Gal and Sia when modeled with AF3. **b,** Alternative *bondedAtomPairs* syntax connecting C6 atom of Gal to O2 atom of Sia generates improper valence on C6 atom of Gal, resulting in an unfavorable bond angle. **c,** Standard *bondedAtomPairs* syntax (O6 atom of Gal connected to C2 atom of Sia) combined with *userCCD* (manual removal of the O2 atom from the SIA mmCIF) correctly models Sia without valence or bonding artifacts. **d,** Demonstration of the deleted lines in SIA mmCIF file necessary to create the corrected *userCCD* input. Both the O2 atom and its associated hydrogen atom (HO2) must be removed to eliminate errors.

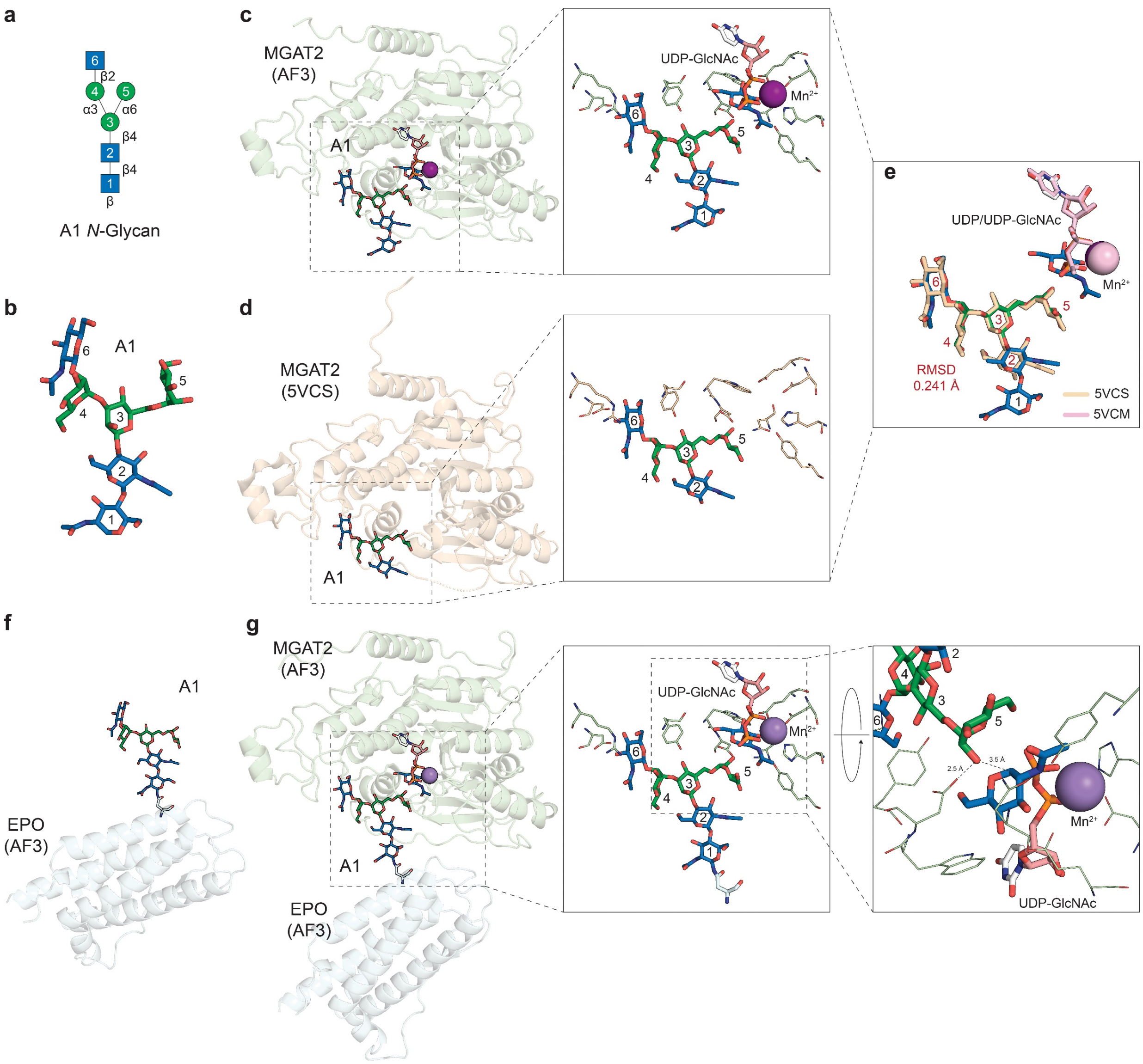

Sup. Fig. 2. Benchmarking AF3 with MGAT2/UDP-GlcNAc/Mn^{2+}/A1 Michaelis complex. a, SNFG cartoon representation of A1 *N*-glycan; residue numbers are consistently annotated across panels. **b,** AF3-modeled free-reducing end A1 glycan. **c,** Model of the *Homo sapiens N*-acetylglucosaminyltransferase MGAT2 bound to UDP-GlcNAc, Mn^{2+} and A1 *N*-glycan, representing a Michaelis complex; the active site is highlighted, showing the glycan binding pose and interacting residues. **d,** Crystallographic structure of MGAT2 in complex with the truncated A1 *N*-glycan (PDB: 5VCS). **e,** Structural alignment of the AF3 MGAT2/UDP-GlcNAc/Mn^{2+}/A1 model (SNFG coloring for the respective monosaccharides) with the crystallographic structure (tan), and a separate MGAT2 structure containing UDP and Mn^{2+} (pink); residues

align well within the catalytic pocket. The root mean square deviation (RMSD) of residues 2-6 (colored in red) between the AF3 model and 5VCS is annotated. **f,** A1 glycan modeled as an *N*-glycan attached to *Homo sapiens* erythropoietin (EPO). **g,** MGAT2 (green) modeled in complex with UDP-GlcNAc, Mn^{2+} and the A1 glycan carried on EPO (cyan). The active site is further zoomed in to highlight the positioning of the C2 hydroxyl group of Man (residue 5) in proximity to the catalytic base and the C1 atom of GlcNAc.

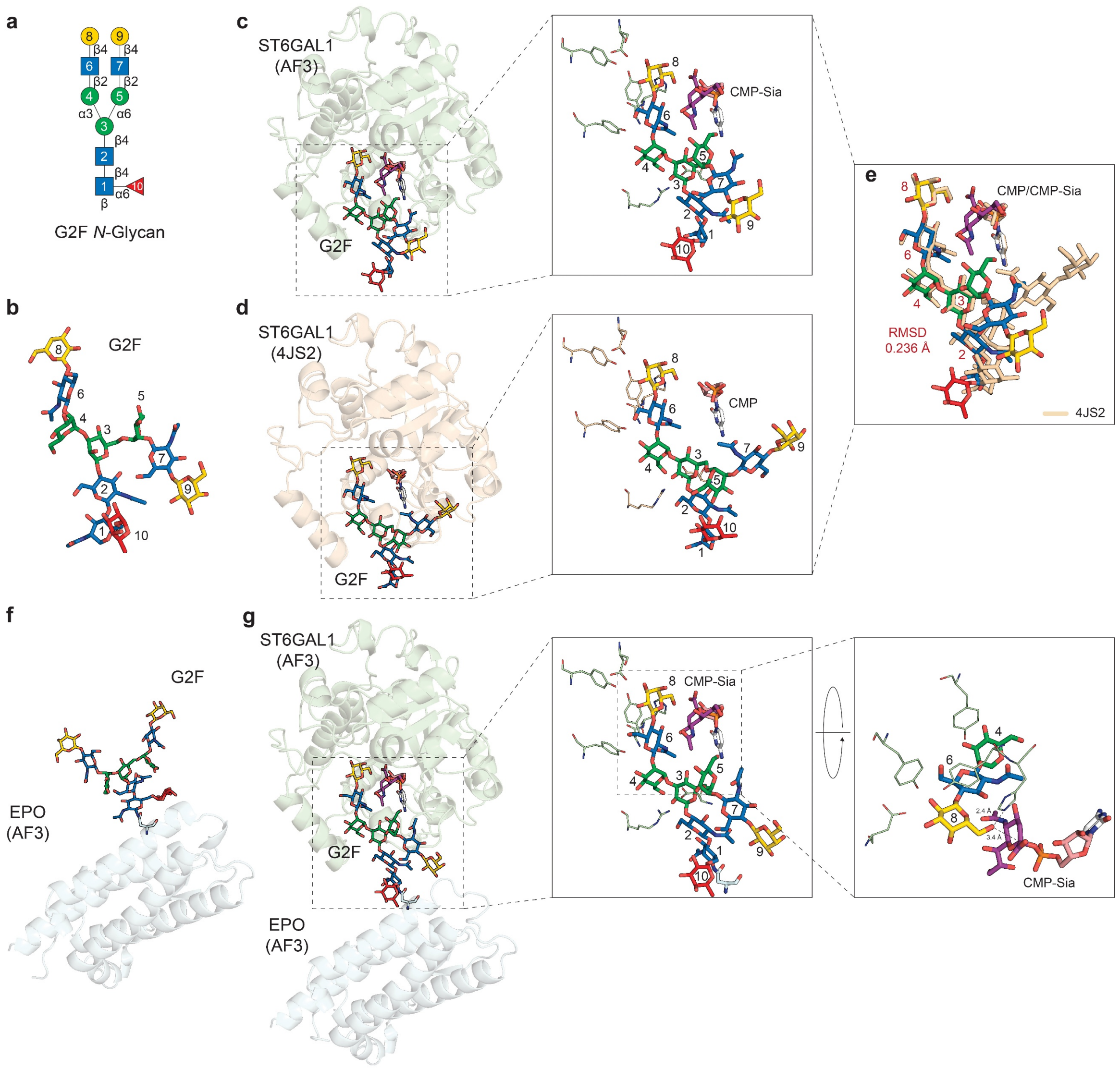

Sup. Fig. 3. Benchmarking AF3 with ST6GAL1/CMP-Sia/G2F Michaelis complex. a, SNFG cartoon representation of G2F *N*-glycan; residue numbers are consistently annotated across panels. **b,** AF3-modeled free-reducing end G2F glycan. **c,** Model of the *Homo sapiens* sialyltransferase ST6GAL1 bound to CMP-Sia and G2F, representing a Michaelis complex; the active site is highlighted, showing the glycan binding pose and interacting residues. **d,** Crystallographic structure of ST6GAL1, where a G2F modification from a crystallographic symmetry mate is positioned within the catalytic pocket (PDB: 4JS2). **e,** Structural alignment of the AF3 ST6GAL1/CMP-Sia/G2F (SNFG coloring for the respective monosaccharides) with the crystallographic structure (tan); GlcNAc (residue 2), β-Man (residue 3) and the α1,3-branched arm

(residues 4, 6, and 8) align closely within the catalytic pocket, whereas the solvent-exposed α1,6-branched arm exhibits greater conformational disorder. The root mean square deviation (RMSD) of residues 2, 3, 4, 6, and 8 (colored in red) between the AF3 model and 4JS2 is annotated. **f,** G2F glycan modeled as an *N*-glycan attached to *Homo sapiens* erythropoietin (EPO). **g,** ST6GAL1 (green) modeled in complex with CMP-Sia and the G2F glycan carried on EPO (cyan). The active site is further magnified in to highlight the positioning of the C6 hydroxyl group of Gal (residue 8) in proximity to the catalytic base and the C2 atom of Sia.

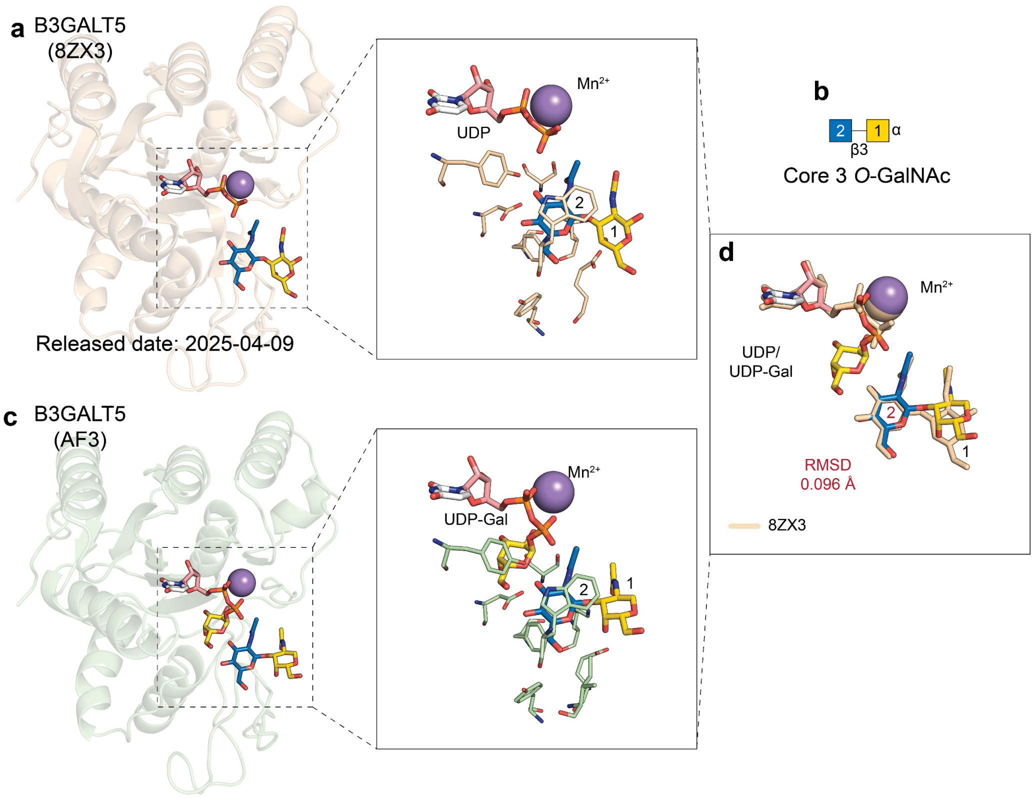

Sup. Fig. 4. Validation of B3GALT5 AF3 model using newly released crystallographic data. a, Crystal structure of *Homo sapiens* B3GALT5 in complex with Core 3 *O*-GalNAc disaccharide, UDP and Mn^{2+} (PDB: 8ZX3), released on April 9, 2025, after the AF3 training and validation cutoffs. The active site is highlighted, showing the glycan binding pose and interacting residues. **b,** SNFG cartoon representation of Core 3 *O*-GalNAc glycan. **c,** AF3 Michaelis complex model of B3GALT5 with Core 3 *O*-GalNAc glycan, UDP-Gal, and Mn^{2+}. **d,** Structural alignment of the AF3 model (colored) with the crystal structure (tan); the root-mean-square deviation (RMSD) of residue 2 (colored in red) is annotated.

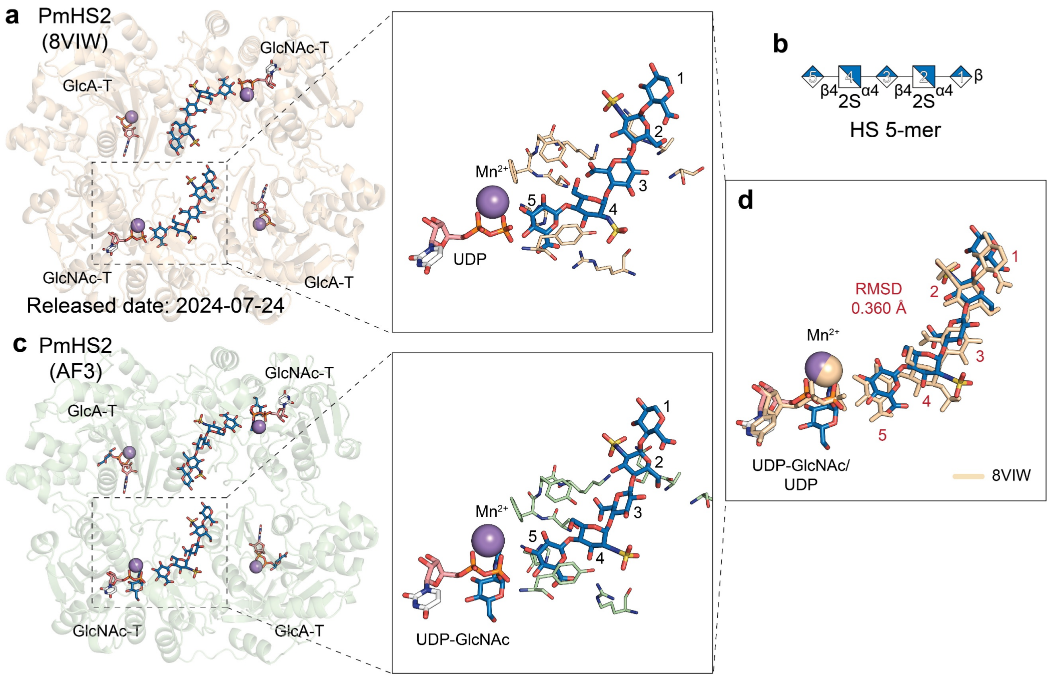

Sup. Fig. 5. Validation of PmHS2 AF3 model using newly released Cryo-EM data. a, Cryo-EM structure of *Pasteurella multocida* heparosan synthase 2 (PmHS2) in complex with 2-*O*-sulfated heparan sulfate (HS) 5-mer, UDP and Mn^{2+} (PDB: 8VIW), released on July 24, 2024, after the AF3 training and validation cutoffs. PmHS2 is a dual-domain glycosyltransferase that forms a functional homodimer, with corresponding sugar transfer activities annotated. The active site is highlighted, showing the glycan binding pose and interacting residues. **b,** SNFG cartoon representation of 2-*O*-sulfated HS 5-mer. **c,** AF3 Michaelis complex model of PmHS2 with 2-*O*-sulfated HS 5-mer, UDP-GlcNAc, and Mn^{2+}. **d,** Structural alignment of the AF3 model (colored) with the cryo-EM structure (tan); the root-mean-square deviation (RMSD) of residues 1-5 (colored in red) is annotated.

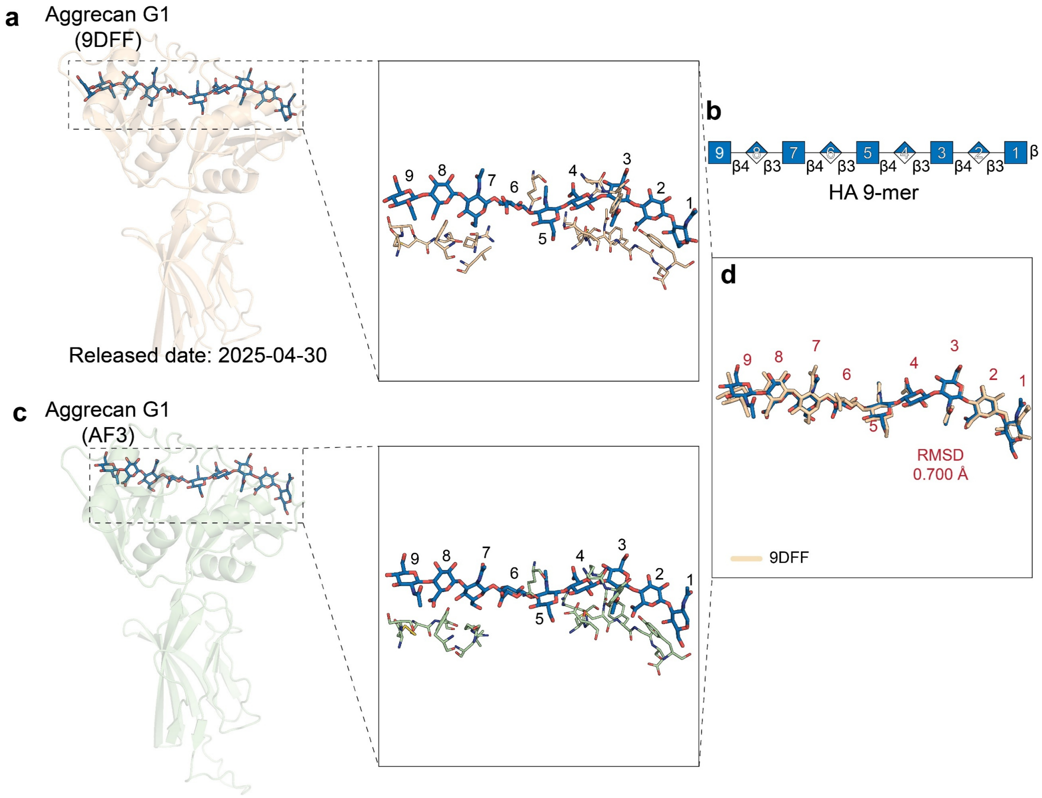

Sup. Fig. 6. Validation of aggrecan G1 domain AF3 model using newly released crystallographic data. **a,** Crystal structure of *Homo sapiens* aggrecan G1 domain in complex with hyaluronan (HA) 10-mer (PDB: 9DFF), released on April 30, 2025, after the AF3 training and validation cutoffs. The non-reducing end unsaturated GlcA, a lyase-derived product, was excluded. The active site is highlighted, showing the glycan binding pose and interacting residues. **b,** SNFG cartoon representation of HA 9-mer. **c,** AF3 model of aggrecan G1 with HA 9-mer. **d,** Structural alignment of the AF3 model (colored) with the crystal structure (tan); the root-mean-square deviation (RMSD) of residues 1-9 (colored in red) is annotated.

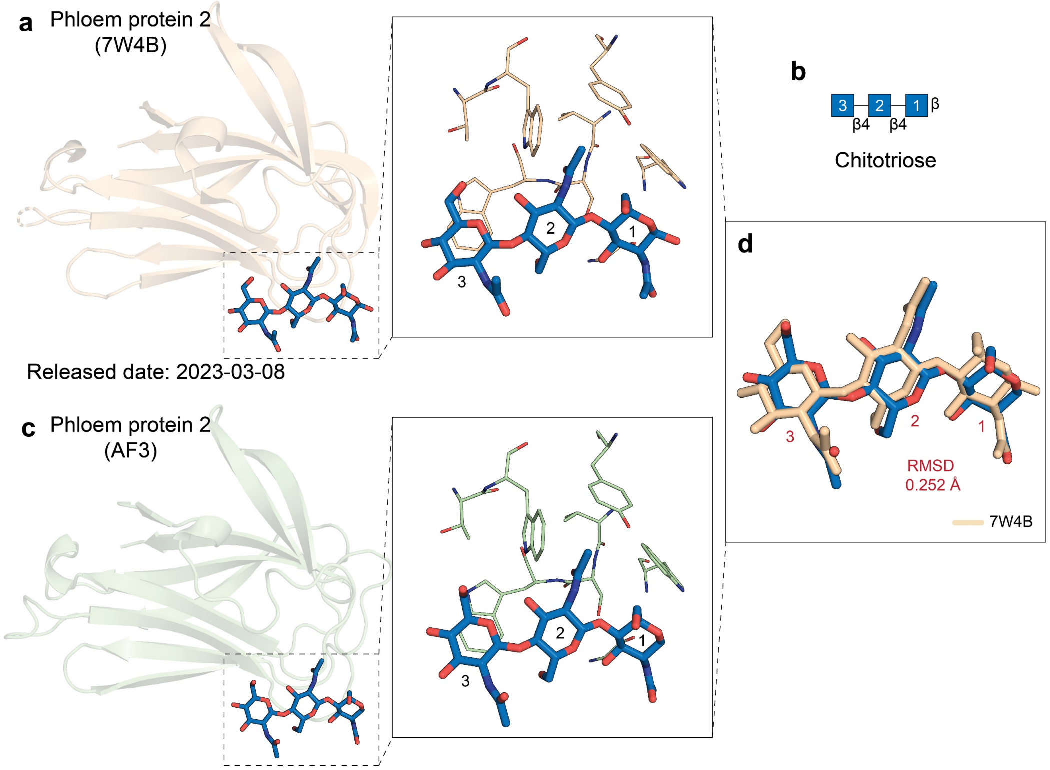

Sup. Fig. 7. Validation of phloem protein 2 AF3 model using newly released crystallographic data. a, Crystal structure *Cucumis sativus* phloem protein 2 (PP2) in complex chitotriose (PDB: 7W4B), released on March 8, 2023, after the AF3 training and validation cutoffs. The active site is highlighted, showing the glycan binding pose and interacting residues. **b,** SNFG cartoon representation of chitotriose. **c,** AF3 model of PP2 with chitotriose. **d,** Structural alignment of the AF3 model (colored) with the crystal structure (tan); the root-mean-square deviation (RMSD) of residues 1-3 (colored in red) is annotated.

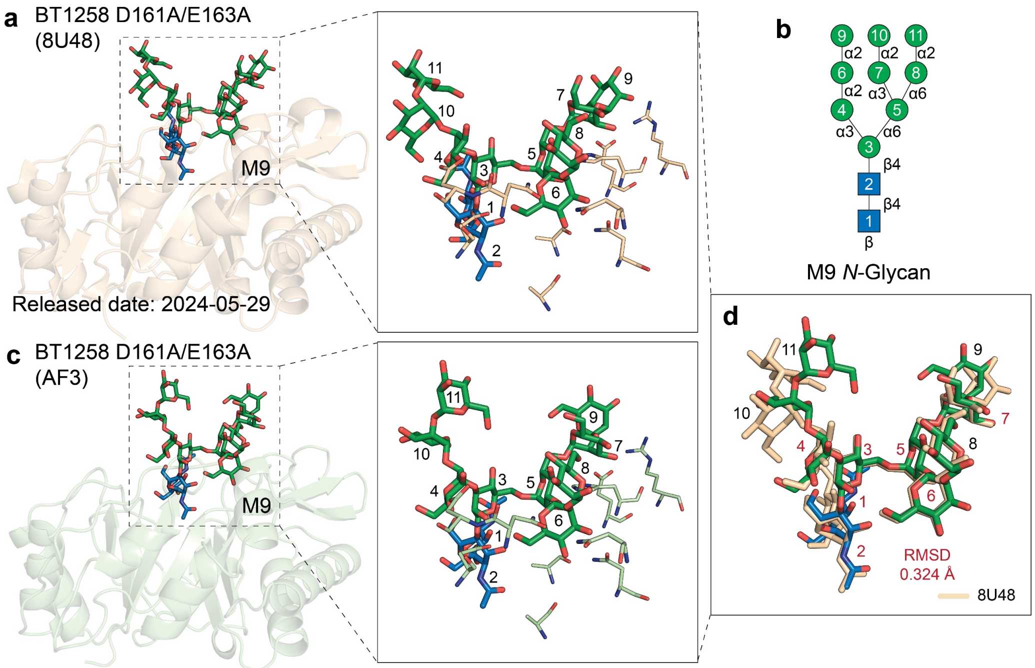

Sup. Fig. 8. Validation of BT1258 D161A/E163A AF3 model using newly released crystallographic data. a, Crystal structure of *Bacteroides thetaiotamicron* endoglycosidase BT1258 D161A/E163A in complex with M9 *N*-glycan (PDB: 8U48), released on May 29, 2024, after the AF3 training and validation cutoffs. The active site is highlighted, showing the glycan binding pose and interacting residues. **b,** SNFG cartoon representation of M9 *N*-glycan. **c,** AF3 model of BT1258 D161A/E163A with M9 *N*-glycan. **d,** Structural alignment of the AF3 model (colored) with the crystal structure (tan); the root-mean-square deviation (RMSD) of residues 1-7 (colored in red) is annotated.

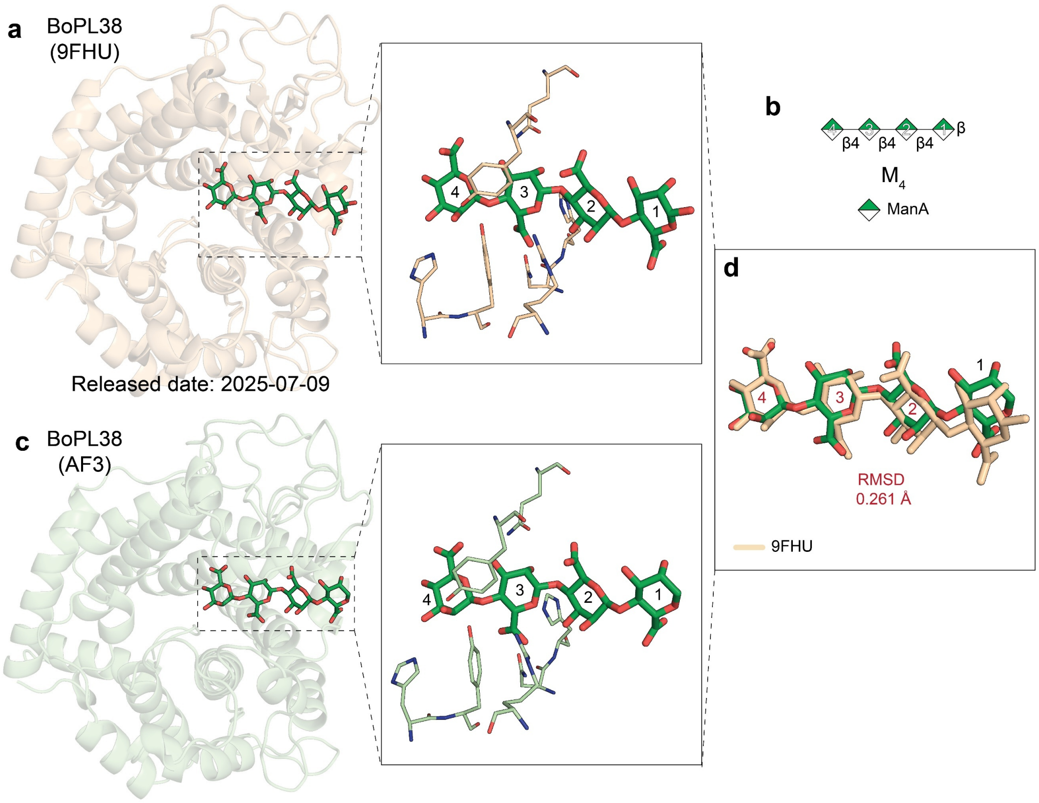

Sup. Fig. 9. Validation of polysaccharide lyase family 38 AF3 model using newly released crystallographic data. a, Crystal structure of *Bacteroides ovatus* polysaccharide lyase family 38 (BoPL38) (PDB: 9FHU), released on July 9, 2025, after the AF3 training and validation cutoffs. The active site is highlighted, showing the glycan binding pose and interacting residues. **b,** SNFG cartoon representation of M_4, in which M represents β-D-mannuronic acid. **c,** AF3 model of BoPL38 with M_4 alginate. **d,** Structural alignment of the AF3 model (colored) with the crystal structure (tan); the root-mean-square deviation (RMSD) of residues 2-4 (colored in red) is annotated.

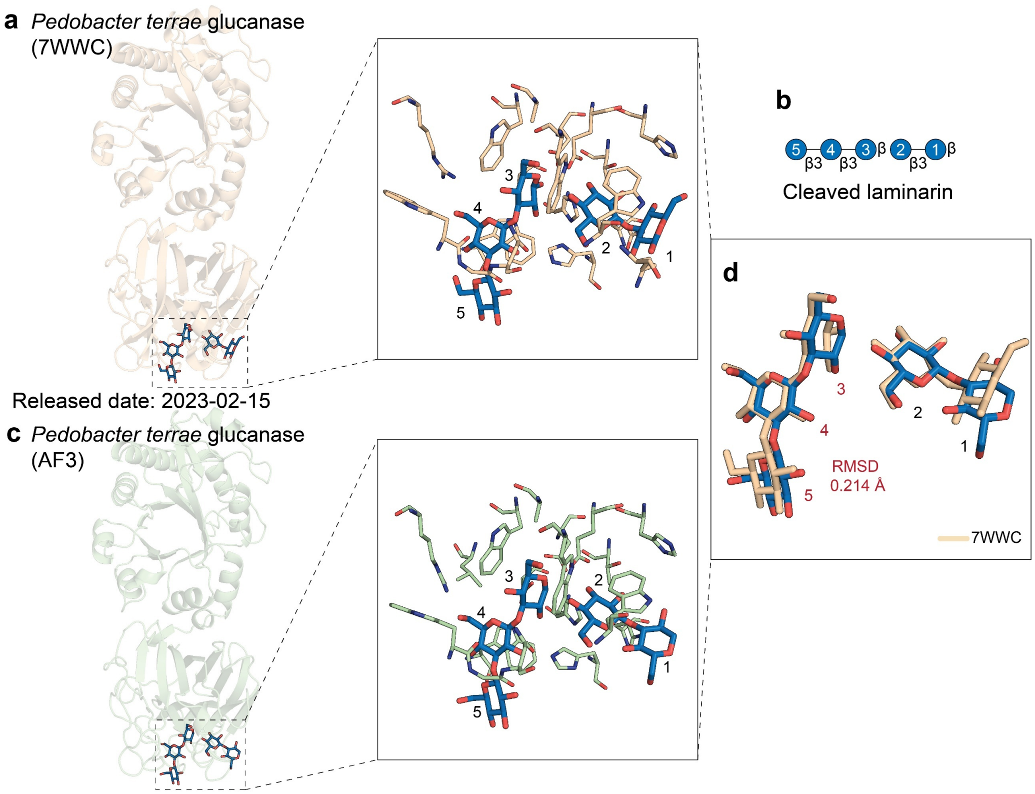

Sup. Fig. 10. Validation of *Pedobacter terrae* glucanase AF3 model using newly released crystallographic data. a, Crystal structure of *Pedobacter terrae* glucanase (PDB: 7WWC), released on February 15, 2023, after the AF3 training and validation cutoffs. The active site is highlighted, showing the glycan binding pose and interacting residues. **b,** SNFG cartoon representation of laminarin, where the cleavage site is between residue 2 and 3. **c,** AF3 model of glucanase with laminarin. **d,** Structural alignment of the AF3 model (colored) with the crystal structure (tan); the root-mean-square deviation (RMSD) of residues 3-5 (colored in red) is annotated.

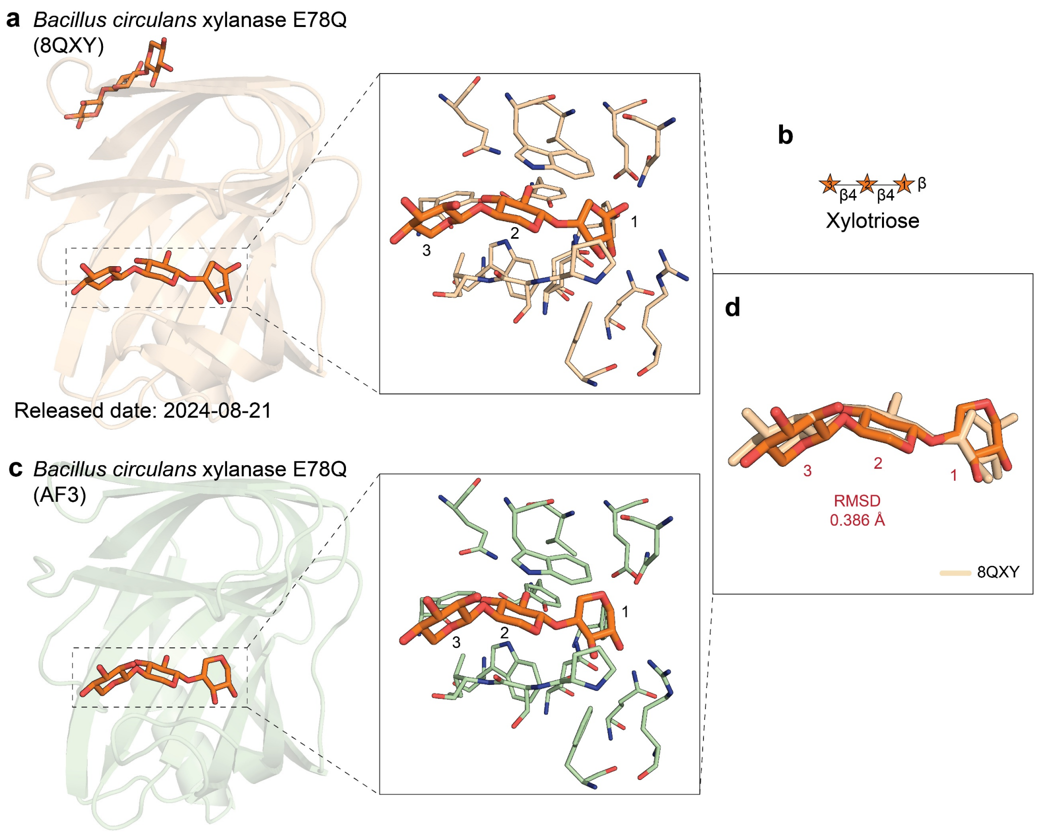

Sup. Fig. 11. Validation of *Bacillus circulans* xylanase E78Q AF3 model using newly released crystallographic data. a, Crystal structure of *Bacillus circulans* xylanase E78Q (PDB: 8QXY), released on August 21, 2024, after the AF3 training and validation cutoffs. The active site is highlighted, showing the glycan binding pose and interacting residues. **b,** SNFG cartoon representation of xylotriose. **c,** AF3 model of xylanse with xylotriose. **d,** Structural alignment of the AF3 model (colored) with the crystal structure (tan); the root-mean-square deviation (RMSD) of residues 1-3 (colored in red) is annotated.

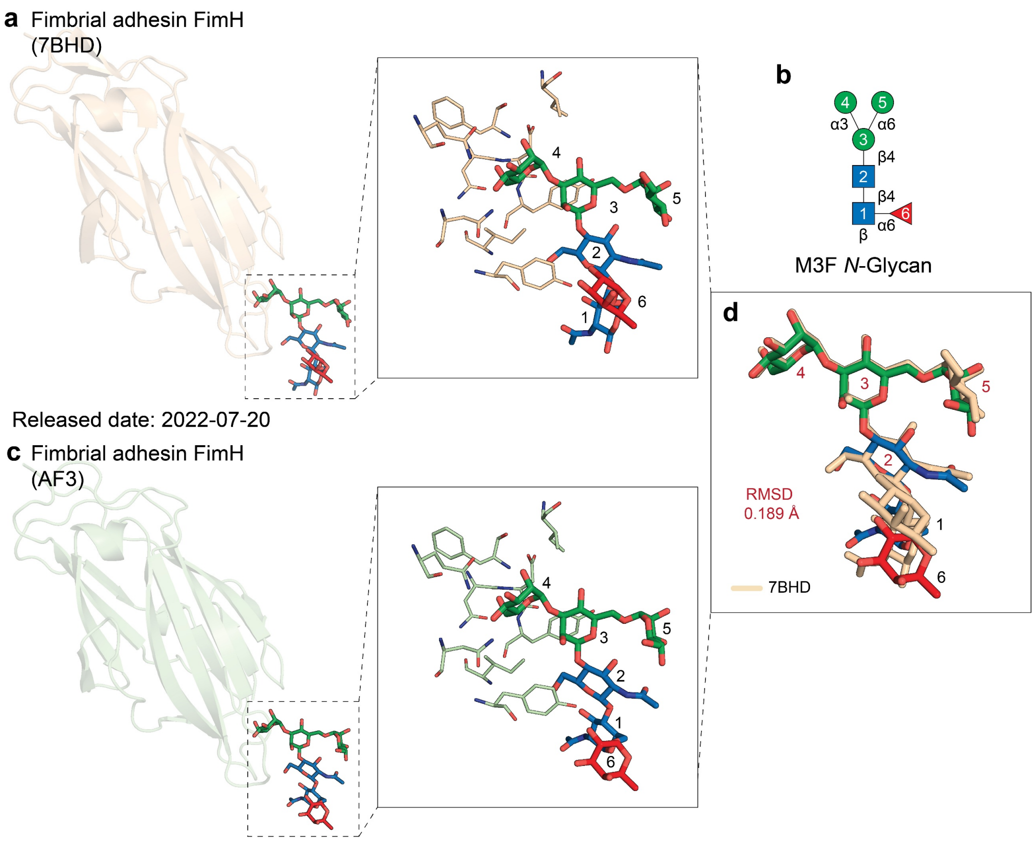

Sup. Fig. 12. Validation of fimbrial adhesin FimH AF3 model using newly released crystallographic data. a, Crystal structure of *Escherichia coli* fimbrial adhesin FimH (PDB: 7BHD), released on July 20, 2022, after the AF3 training and validation cutoffs. The active site is highlighted, showing the glycan binding pose and interacting residues. **b,** SNFG cartoon representation of M3F *N*-glycan. **c,** AF3 model of FimH with M3F *N*-glycan. **d,** Structural alignment of the AF3 model (colored) with the crystal structure (tan); the root-mean-square deviation (RMSD) of residues 2-5 (colored in red) is annotated.

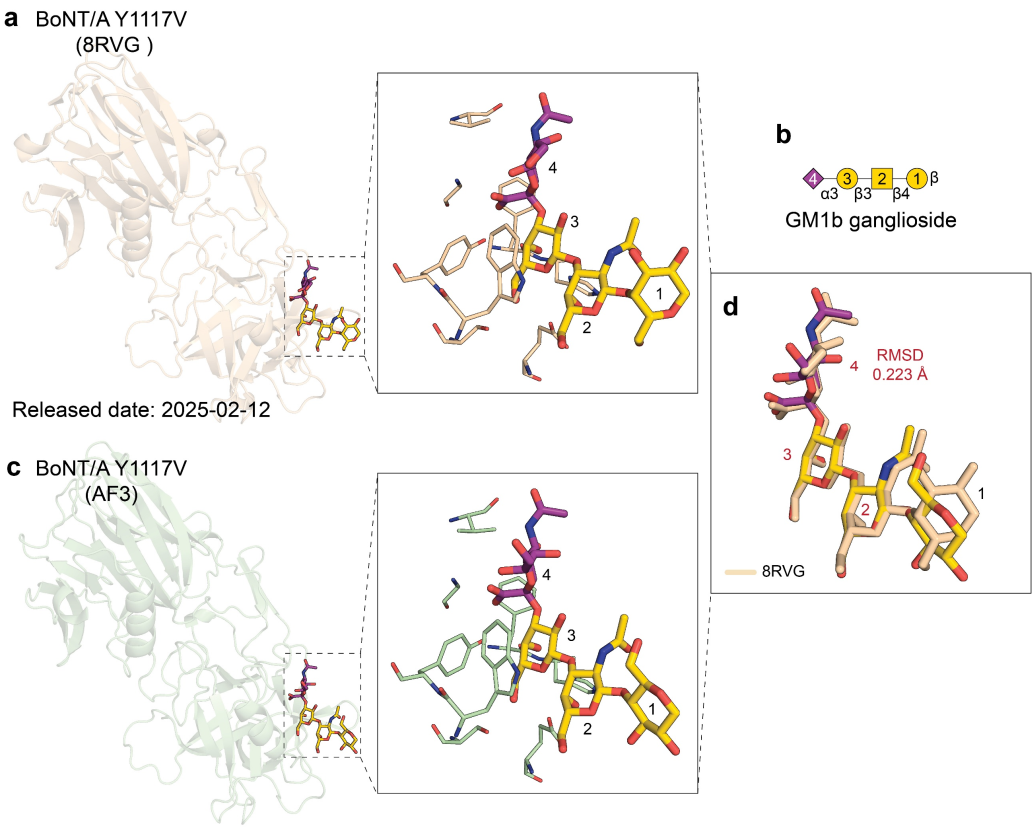

Sup. Fig. 13. Validation of botulinum neurotoxin A Y1117V AF3 model using newly released crystallographic data. a, Crystal structure of of botulinum neurotoxin A (BoNT/A) Y1117V from *Clostridium botulinum* (PDB: 8RVG), released on February 12, 2025, after the AF3 training and validation cutoffs. The active site is highlighted, showing the glycan binding pose and interacting residues. **b,** SNFG cartoon representation of GM1b ganglioside. **c,** AF3 model of BoNT/A Y1117V with GM1b. **d,** Structural alignment of the AF3 model (colored) with the crystal structure (tan); the root-mean-square deviation (RMSD) of residues 2-4 (colored in red) is annotated.

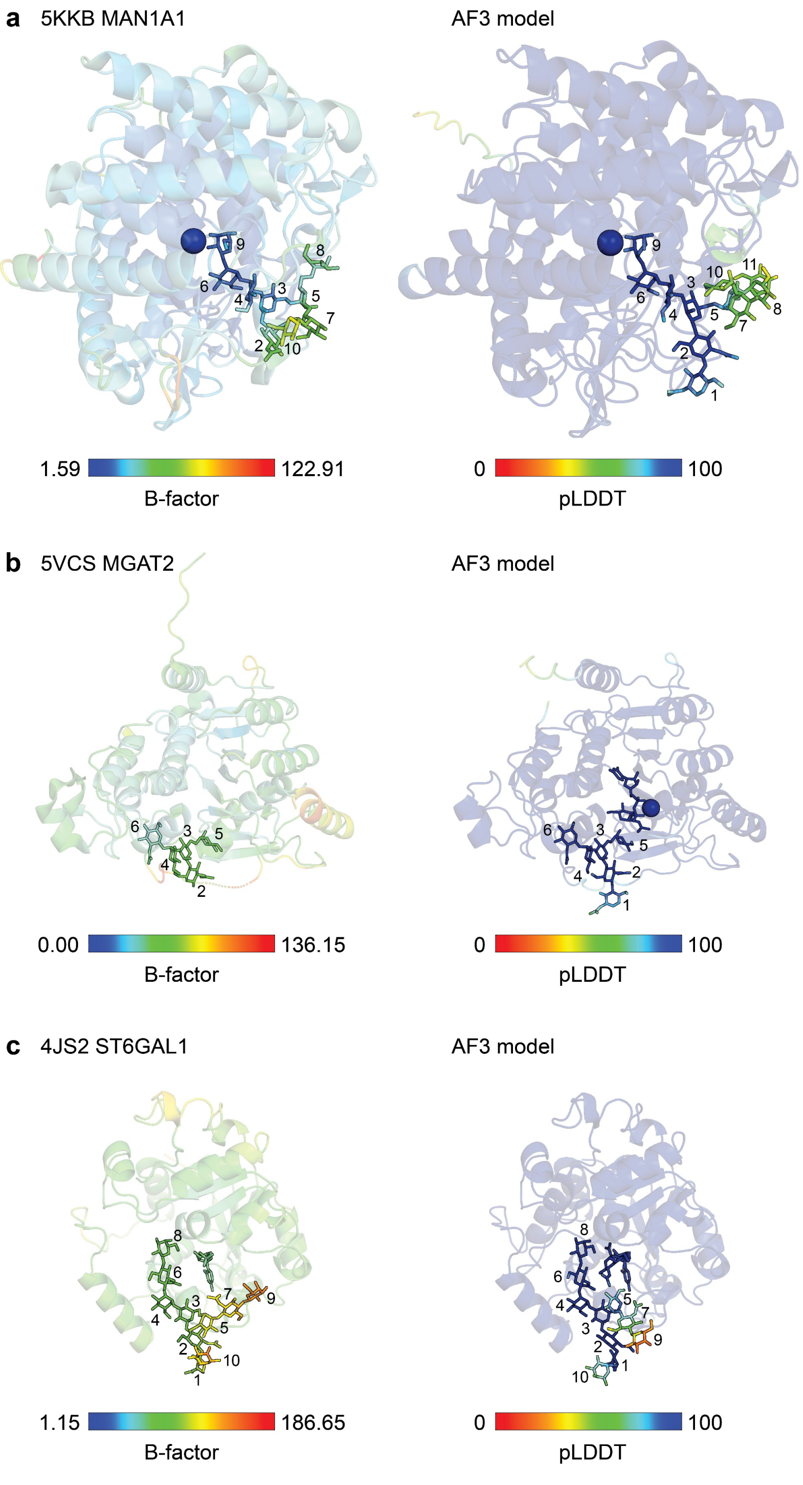

Sup. Fig. 14. Comparisons of crystallographic B-factor and AF3 pLDDT scores. a, The crystal structure B-factor is shown alongside the corresponding predicted local-distance difference test (pLDDT) score from the AF3 model of *Mus musculus* Golgi mannosidase MAN1A1, **b,** *Homo sapiens N*-

acetylglucosaminyltransferase MGAT2, and **c,** *Homo sapiens* sialyltransferase ST6GAL. B-factor color bars are scaled according to the values reported in the corresponding crystal structures.
