# Supplementary material for: Modeling glycans with AlphaFold 3: capabilities, caveats, and limitations
Source: Glycobiology. 2025 Aug 28;35(10):cwaf048. doi: 10.1093/glycob/cwaf048 (PMC12448869; doi:10.1093/glycob/cwaf048)
Supplement: Supplementary_Document_1_cwaf048 [file supplementary_document_1_cwaf048.pdf]

Supplementary Document 1
Highlighted lines indicate the modifications required for generating the user-defined CCD (*userCCD*) of α-linked N-acetylneuraminic acid (SIA). These lines should be removed from the original mmCIF file to ensure proper valence and bonding during AlphaFold 3 modeling.

```
data_SIA
#
_chem_comp.id SIA
_chem_comp.name 'N-acetyl-alpha-neuraminic acid'
_chem_comp.type 'D-saccharide, alpha linking'
_chem_comp.pdbx_type ATOMS
_chem_comp.formula 'C11 H19 N O9'
_chem_comp.mon_nstd_parent_comp_id ?
_chem_comp.pdbx_synonyms 'N-acetylneuraminic acid; sialic acid; alpha-
sialic acid; O-SIALIC ACID'
_chem_comp.pdbx_formal_charge 0
_chem_comp.pdbx_initial_date 1999-07-08
_chem_comp.pdbx_modified_date 2024-09-27
_chem_comp.pdbx_ambiguous_flag N
_chem_comp.pdbx_release_status REL
_chem_comp.pdbx_replaced_by ?
_chem_comp.pdbx_replaces NAN
_chem_comp.formula_weight 309.270
_chem_comp.one_letter_code ?
_chem_comp.three_letter_code SIA
_chem_comp.pdbx_model_coordinates_details ?
_chem_comp.pdbx_model_coordinates_missing_flag N
_chem_comp.pdbx_ideal_coordinates_details ?
_chem_comp.pdbx_ideal_coordinates_missing_flag N
_chem_comp.pdbx_model_coordinates_db_code ?
_chem_comp.pdbx_subcomponent_list ?
_chem_comp.pdbx_processing_site EBI
_chem_comp.pdbx_pcm Y
#
loop_
_pdbx_chem_comp_synonyms.ordinal
_pdbx_chem_comp_synonyms.comp_id
_pdbx_chem_comp_synonyms.name
_pdbx_chem_comp_synonyms.provenance
_pdbx_chem_comp_synonyms.type
1 SIA 'N-acetylneuraminic acid' PDB ?
2 SIA 'sialic acid' PDB ?
3 SIA 'alpha-sialic acid' PDB ?
4 SIA 'O-SIALIC ACID' PDB ?
#
loop_
_chem_comp_atom.comp_id
_chem_comp_atom.atom_id
_chem_comp_atom.alt_atom_id
_chem_comp_atom.type_symbol
_chem_comp_atom.charge
```

```
_chem_comp_atom.pdbx_align
_chem_comp_atom.pdbx_aromatic_flag
_chem_comp_atom.pdbx_leaving_atom_flag
_chem_comp_atom.pdbx_stereo_config
_chem_comp_atom.pdbx_backbone_atom_flag
_chem_comp_atom.pdbx_n_terminal_atom_flag
_chem_comp_atom.pdbx_c_terminal_atom_flag
_chem_comp_atom.model_Cartn_x
_chem_comp_atom.model_Cartn_y
_chem_comp_atom.model_Cartn_z
_chem_comp_atom.pdbx_model_Cartn_x_ideal
_chem_comp_atom.pdbx_model_Cartn_y_ideal
_chem_comp_atom.pdbx_model_Cartn_z_ideal
_chem_comp_atom.pdbx_component_atom_id
_chem_comp_atom.pdbx_component_comp_id
_chem_comp_atom.pdbx_ordinal
SIA C1 C1 C 0 1 N N N N N N -2.196 58.872 -5.981 -2.502 -0.832 0.174
C1 SIA 1
SIA C2 C2 C 0 1 N N R N N N -1.870 58.021 -7.211 -2.171 0.628 0.342 C2
SIA 2
SIA C3 C3 C 0 1 N N N N N N -0.844 56.899 -7.306 -1.789 0.898 1.800 C3
SIA 3
SIA C4 C4 C 0 1 N N S N N N -1.157 55.904 -8.413 -0.586 0.023 2.171 C4
SIA 4
SIA C5 C5 C 0 1 N N R N N N -2.015 56.516 -9.517 0.529 0.264 1.148 C5
SIA 5
SIA C6 C6 C 0 1 N N R N N N -3.352 56.956 -8.912 -0.026 0.043 -0.259
C6 SIA 6
SIA C7 C7 C 0 1 N N R N N N -4.224 57.698 -9.942 1.088 0.251 -1.286 C7
SIA 7
SIA C8 C8 C 0 1 N N R N N N -5.571 58.131 -9.360 0.535 0.021 -2.694 C8
SIA 8
SIA C9 C9 C 0 1 N N N N N N -6.601 58.674 -10.381 1.650 0.229 -3.721
C9 SIA 9
SIA C10 C10 C 0 1 N N N N N N -1.897 55.374 -11.759 2.632 -0.329 2.226
C10 SIA 10
SIA C11 C11 C 0 1 N N N N N N -2.200 54.057 -12.454 3.763 -1.292 2.478
C11 SIA 11
SIA N5 N5 N 0 1 N N N N N N -2.202 55.444 -10.478 1.629 -0.671 1.394
N5 SIA 12
SIA O1A O1A O 0 1 N N N N N N -1.289 58.815 -5.130 -2.191 -1.408 -
0.841 O1A SIA 13
SIA O1B O1B O 0 1 N N N N N N -3.210 59.504 -5.631 -3.141 -1.493 1.152
O1B SIA 14
SIA O2 O2 O 0 1 N Y N N N N -1.768 59.214 -7.992 -3.312 1.416 -0.003
O2 SIA 15
SIA O4 O4 O 0 1 N N N N N N 0.072 55.523 -8.986 -0.123 0.370 3.478 O4
SIA 16
SIA O6 O6 O 0 1 N N N N N N -3.149 57.908 -7.847 -1.082 0.968 -0.513
O6 SIA 17
SIA O7 O7 O 0 1 N N N N N N -3.594 58.883 -10.402 1.588 1.586 -1.183
O7 SIA 18
```

SIA O8 O8 O 0 1 N N N N N N -6.119 56.946 -8.828 0.035 -1.313 -2.797
O8 SIA 19
SIA O9 O9 O 0 1 N N N N N N -6.931 57.687 -11.346 1.133 0.014 -5.035
O9 SIA 20
SIA O10 O10 O 0 1 N N N N N N -1.423 56.357 -12.331 2.624 0.753 2.772
O10 SIA 21
SIA H32 H31 H 0 1 N N N N N N -0.702 56.484 -6.300 -2.631 0.655 2.448
H32 SIA 22
SIA H31 H32 H 0 1 N N N N N N 0.120 57.408 -7.182 -1.526 1.949 1.919
H31 SIA 23
SIA H4 H4 H 0 1 N N N N N N -1.651 55.060 -7.897 -0.878 -1.026 2.153
H4 SIA 24
SIA H5 H5 H 0 1 N N N N N N -1.506 57.375 -9.979 0.893 1.287 1.240 H5
SIA 25
SIA H6 H6 H 0 1 N N N N N N -3.850 56.075 -8.492 -0.408 -0.973 -0.341
H6 SIA 26
SIA H7 H7 H 0 1 N N N N N N -4.339 57.176 -10.907 1.896 -0.454 -1.093
H7 SIA 27
SIA H8 H8 H 0 1 N N N N N N -5.473 58.871 -8.553 -0.272 0.728 -2.887
H8 SIA 28
SIA H92 H91 H 0 1 N N N N N N -6.054 59.459 -10.925 2.031 1.247 -3.642
H92 SIA 29
SIA H91 H92 H 0 1 N N N N N N -7.587 59.029 -10.055 2.457 -0.476 -
3.528 H91 SIA 30
SIA H111 H111 H 0 0 N N N N N N -3.215 53.728 -12.207 4.474 -0.844
3.172 H111 SIA 31
SIA H113 H112 H 0 0 N N N N N N -1.550 53.279 -12.033 3.368 -2.213
2.907 H113 SIA 32
SIA H112 H113 H 0 0 N N N N N N -2.005 54.041 -13.531 4.266 -1.516
1.537 H112 SIA 33
SIA HN5 HN5 H 0 1 N N N N N N -2.566 54.658 -10.003 1.635 -1.538 0.957
HN5 SIA 34
SIA HO1B HOB1 H 0 0 N N N N N N -3.412 60.032 -4.867 -3.353 -2.430
1.044 HO1B SIA 35
SIA HO2 HO2 H 0 1 N Y N N N N -0.905 59.113 -8.438 -3.519 1.217 -0.926
HO2 SIA 36
SIA HO4 HO4 H 0 1 N Y N N N N 0.427 54.801 -8.430 -0.854 0.203 4.087
HO4 SIA 37
SIA HO7 HO7 H 0 1 N Y N N N N -3.109 58.884 -9.548 0.844 2.177 -1.360
HO7 SIA 38
SIA HO8 HO8 H 0 1 N Y N N N N -7.071 57.051 -8.962 0.779 -1.904 -2.620
HO8 SIA 39
SIA HO9 HO9 H 0 1 N Y N N N N -6.783 56.885 -10.808 1.866 0.155 -5.650
HO9 SIA 40
#
loop_
_chem_comp_bond.comp_id
_chem_comp_bond.atom_id_1
_chem_comp_bond.atom_id_2
_chem_comp_bond.value_order
_chem_comp_bond.pdbx_aromatic_flag
_chem_comp_bond.pdbx_stereo_config

```
_chem_comp_bond.pdbx_ordinal
SIA C1 C2 SING N N 1
SIA C1 O1A DOUB N N 2
SIA C1 O1B SING N N 3
SIA C2 C3 SING N N 4
SIA C2 O2 SING N N 5
SIA C2 O6 SING N N 6
SIA C3 C4 SING N N 7
SIA C3 H32 SING N N 8
SIA C3 H31 SING N N 9
SIA C4 C5 SING N N 10
SIA C4 O4 SING N N 11
SIA C4 H4 SING N N 12
SIA C5 C6 SING N N 13
SIA C5 N5 SING N N 14
SIA C5 H5 SING N N 15
SIA C6 C7 SING N N 16
SIA C6 O6 SING N N 17
SIA C6 H6 SING N N 18
SIA C7 C8 SING N N 19
SIA C7 O7 SING N N 20
SIA C7 H7 SING N N 21
SIA C8 C9 SING N N 22
SIA C8 O8 SING N N 23
SIA C8 H8 SING N N 24
SIA C9 O9 SING N N 25
SIA C9 H92 SING N N 26
SIA C9 H91 SING N N 27
SIA C10 C11 SING N N 28
SIA C10 N5 SING N N 29
SIA C10 O10 DOUB N N 30
SIA C11 H111 SING N N 31
SIA C11 H113 SING N N 32
SIA C11 H112 SING N N 33
SIA N5 HN5 SING N N 34
SIA O1B HO1B SING N N 35
SIA O2 HO2 SING N N 36
SIA O4 HO4 SING N N 37
SIA O7 HO7 SING N N 38
SIA O8 HO8 SING N N 39
SIA O9 HO9 SING N N 40
#
loop_
_pdbx_chem_comp_descriptor.comp_id
_pdbx_chem_comp_descriptor.type
_pdbx_chem_comp_descriptor.program
_pdbx_chem_comp_descriptor.program_version
_pdbx_chem_comp_descriptor.descriptor
SIA SMILES ACDLabs 10.04 'O=C(O)C1(O)OC(C(O)C(O)CO)C(NC(=O)C)C(O)C1'
SIA SMILES_CANONICAL CACTVS 3.341
'CC(=O)N[C@@H]1[C@@H](O)C[C@@](O)(O[C@H]1[C@H](O)[C@H](O)CO)C(O)=O'
```

SIA SMILES CACTVS 3.341
'CC(=O)N[CH]1[CH](O)C[C](O)(O[CH]1[CH](O)[CH](O)CO)C(O)=O'
SIA SMILES_CANONICAL 'OpenEye OEToolkits' 1.5.0
'CC(=O)N[C@@H]1[C@H](C[C@@](O[C@H]1[C@@H]([C@@H](CO)O)O)(C(=O)O)O)O'
SIA SMILES 'OpenEye OEToolkits' 1.5.0
'CC(=O)NC1C(CC(OC1C(C(CO)O)O)(C(=O)O)O)O'
SIA InChI InChI 1.03 'InChI=1S/C11H19NO9/c1-4(14)12-7-5(15)2-
11(20,10(18)19)21-9(7)8(17)6(16)3-13/h5-9,13,15-17,20H,2-
3H2,1H3,(H,12,14)(H,18,19)/t5-,6+,7+,8+,9+,11+/m0/s1'
SIA InChIKey InChI 1.03 SQVRNKJHWKZAKO-YRMXFSIDSA-N
#
loop_
_pdbx_chem_comp_identifier.comp_id
_pdbx_chem_comp_identifier.type
_pdbx_chem_comp_identifier.program
_pdbx_chem_comp_identifier.program_version
_pdbx_chem_comp_identifier.identifier
SIA 'SYSTEMATIC NAME' ACDLabs 10.04 '5-(acetylamino)-3,5-dideoxy-D-
glycero-alpha-D-galacto-non-2-ulopyranosonic acid'
SIA 'SYSTEMATIC NAME' 'OpenEye OEToolkits' 1.5.0 '(2R,4S,5R,6R)-5-
acetamido-2,4-dihydroxy-6-[(1R,2R)-1,2,3-trihydroxypropyl]oxane-2-
carboxylic acid'
SIA 'CONDENSED IUPAC CARBOHYDRATE SYMBOL' GMML 1.0 DNeup5Aca
SIA 'COMMON NAME' GMML 1.0 'N-acetyl-a-D-neuraminic acid'
SIA 'IUPAC CARBOHYDRATE SYMBOL' PDB-CARE 1.0 a-D-Neup5Ac
SIA 'SNFG CARBOHYDRATE SYMBOL' GMML 1.0 Neu5Ac
#
loop_
_pdbx_chem_comp_feature.comp_id
_pdbx_chem_comp_feature.type
_pdbx_chem_comp_feature.value
_pdbx_chem_comp_feature.source
_pdbx_chem_comp_feature.support
SIA 'CARBOHYDRATE ISOMER' D PDB ?
SIA 'CARBOHYDRATE RING' pyranose PDB ?
SIA 'CARBOHYDRATE ANOMER' alpha PDB ?
SIA 'CARBOHYDRATE PRIMARY CARBONYL GROUP' ketose PDB ?
#
loop_
_pdbx_chem_comp_audit.comp_id
_pdbx_chem_comp_audit.action_type
_pdbx_chem_comp_audit.date
_pdbx_chem_comp_audit.processing_site
SIA 'Create component' 1999-07-08 EBI
SIA 'Modify descriptor' 2011-06-04 RCSB
SIA 'Other modification' 2019-08-12 RCSB
SIA 'Other modification' 2019-12-19 RCSB
SIA 'Other modification' 2020-07-03 RCSB
SIA 'Modify name' 2020-07-17 RCSB
SIA 'Modify synonyms' 2020-07-17 RCSB
SIA 'Modify atom id' 2020-07-17 RCSB
SIA 'Modify component atom id' 2020-07-17 RCSB

```
SIA 'Modify PCM' 2024-09-27 PDBE
#
_pdbx_chem_comp_pcm.pcm_id 1
_pdbx_chem_comp_pcm.comp_id SIA
_pdbx_chem_comp_pcm.modified_residue_id THR
_pdbx_chem_comp_pcm.type None
_pdbx_chem_comp_pcm.category Carbohydrate
_pdbx_chem_comp_pcm.position 'Amino-acid side chain'
_pdbx_chem_comp_pcm.polypeptide_position 'Any position'
_pdbx_chem_comp_pcm.comp_id_linking_atom C2
_pdbx_chem_comp_pcm.modified_residue_id_linking_atom OG1
_pdbx_chem_comp_pcm.uniprot_specific_ptm_accession ?
_pdbx_chem_comp_pcm.uniprot_generic_ptm_accession ?
#
_pdbe_chem_comp_drugbank_details.comp_id SIA
_pdbe_chem_comp_drugbank_details.drugbank_id DB03721
_pdbe_chem_comp_drugbank_details.type 'small molecule'
_pdbe_chem_comp_drugbank_details.name 'N-acetyl-alpha-neuraminic acid'
_pdbe_chem_comp_drugbank_details.description
'An N-acyl derivative of neuraminic acid. N-acetylneuraminic acid
occurs in many polysaccharides, glycoproteins, and glycolipids in
animals and bacteria. (From Dorland, 28th ed, p1518)'
_pdbe_chem_comp_drugbank_details.cas_number 21646-00-4
_pdbe_chem_comp_drugbank_details.mechanism_of_action ?
#
loop_
_pdbe_chem_comp_synonyms.comp_id
_pdbe_chem_comp_synonyms.name
_pdbe_chem_comp_synonyms.provenance
_pdbe_chem_comp_synonyms.type
SIA 'N-acetylneuraminic acid' wwPDB ?
SIA 'sialic acid' wwPDB ?
SIA 'alpha-sialic acid' wwPDB ?
SIA 'O-SIALIC ACID' wwPDB ?
SIA 'N-Acetyl-alpha-D-neuraminic acid' DrugBank ?
SIA 'O-sialic acid' DrugBank ?
SIA 'α-Neu5Ac' DrugBank ?
#
_pdbe_chem_comp_drugbank_classification.comp_id SIA
_pdbe_chem_comp_drugbank_classification.drugbank_id DB03721
_pdbe_chem_comp_drugbank_classification.parent 'N-acylneuraminic
acids'
_pdbe_chem_comp_drugbank_classification.kingdom 'Organic compounds'
_pdbe_chem_comp_drugbank_classification.class 'Organooxygen compounds'
_pdbe_chem_comp_drugbank_classification.superclass 'Organic oxygen
compounds'
_pdbe_chem_comp_drugbank_classification.description
'This compound belongs to the class of organic compounds known as n-
acylneuraminic acids. These are neuraminic acids carrying an N-acyl
substituent.'
#
loop_
```

_pdbe_chem_comp_drugbank_targets.comp_id
_pdbe_chem_comp_drugbank_targets.drugbank_id
_pdbe_chem_comp_drugbank_targets.name
_pdbe_chem_comp_drugbank_targets.organism
_pdbe_chem_comp_drugbank_targets.uniprot_id
_pdbe_chem_comp_drugbank_targets.pharmacologically_active
_pdbe_chem_comp_drugbank_targets.ordinal
SIA DB03721 P-selectin Humans P16109 yes 1
SIA DB03721 E-selectin Humans P16581 yes 2
SIA DB03721 'Liver carboxylesterase 1' Humans P23141 yes 3
SIA DB03721 '3-deoxy-manno-octulosonate cytidylyltransferase'
'Escherichia coli' P42216 unknown 4
SIA DB03721 'Tetanus toxin' 'Clostridium tetani (strain Massachusetts
/ E88)' P04958 unknown 5
SIA DB03721 'Cholera enterotoxin subunit B' 'Vibrio cholerae serotype
O1 (strain ATCC 39315 / El Tor Inaba N16961)' P01556 unknown 6
SIA DB03721 'Botulinum neurotoxin type B' 'Clostridium botulinum'
P10844 unknown 7
SIA DB03721 'Mannose-binding protein C' Humans P11226 unknown 8
SIA DB03721 Lithostathine-1-alpha Humans P05451 unknown 9
SIA DB03721 Endo-N-acetylneuraminidase 'Enterobacteria phage K1F'
Q04830 unknown 10
SIA DB03721 'Enterotoxin type B' 'Staphylococcus aureus' P01552
unknown 11
SIA DB03721 Neuraminidase 'Influenza A virus (strain
A/Tern/Australia/G70C/1975 H11N9)' P03472 unknown 12
SIA DB03721 Hemagglutinin-neuraminidase NDV P32884 unknown 13
SIA DB03721 Fiber 'Human adenovirus 19' Q64822 unknown 14
SIA DB03721 Sialoadhesin Humans Q9BZZ2 unknown 15
SIA DB03721 Zinc-alpha-2-glycoprotein Humans P25311 unknown 16
SIA DB03721 'Capsid protein VP1' MPyV P49302 unknown 17
SIA DB03721 Fiber 'Human adenovirus D37' Q64823 unknown 18
#
loop_
_software.name
_software.version
_software.description
rdkit 2023.09.6 'Core functionality.'
pdbeccdutils 0.8.6 'Wrapper to provide 2D templates and molecular
fragments.'
#
loop_
_pdbe_chem_comp_atom_depiction.comp_id
_pdbe_chem_comp_atom_depiction.atom_id
_pdbe_chem_comp_atom_depiction.element
_pdbe_chem_comp_atom_depiction.model_Cartn_x
_pdbe_chem_comp_atom_depiction.model_Cartn_y
_pdbe_chem_comp_atom_depiction.pdbx_ordinal
SIA C1 C 5.654 -3.375 1
SIA C2 C 6.404 -2.076 2
SIA C3 C 5.104 -1.326 3
SIA C4 C 5.104 0.174 4

```
SIA C5 C 6.404 0.924 5
SIA C6 C 7.702 0.174 6
SIA C7 C 9.002 0.924 7
SIA C8 C 10.301 0.174 8
SIA C9 C 11.600 0.924 9
SIA C10 C 5.104 3.174 10
SIA C11 C 3.805 2.424 11
SIA N5 N 6.404 2.424 12
SIA O1A O 4.154 -3.375 13
SIA O1B O 6.404 -4.674 14
SIA O2 O 7.154 -3.375 15
SIA O4 O 3.805 0.924 16
SIA O6 O 7.702 -1.326 17
SIA O7 O 9.002 2.424 18
SIA O8 O 10.301 -1.326 19
SIA O9 O 12.899 0.174 20
SIA O10 O 5.104 4.674 21
#
loop_
_pdbe_chem_comp_bond_depiction.comp_id
_pdbe_chem_comp_bond_depiction.atom_id_1
_pdbe_chem_comp_bond_depiction.atom_id_2
_pdbe_chem_comp_bond_depiction.value_order
_pdbe_chem_comp_bond_depiction.bond_dir
_pdbe_chem_comp_bond_depiction.pdbx_ordinal
SIA C1 C2 SINGLE NONE 1
SIA C1 O1A DOUBLE NONE 2
SIA C1 O1B SINGLE NONE 3
SIA C2 C3 SINGLE NONE 4
SIA C2 O2 SINGLE BEGINDASH 5
SIA C2 O6 SINGLE NONE 6
SIA C3 C4 SINGLE NONE 7
SIA C4 C5 SINGLE NONE 8
SIA C4 O4 SINGLE BEGINDASH 9
SIA C5 C6 SINGLE NONE 10
SIA C5 N5 SINGLE BEGINWEDGE 11
SIA C6 C7 SINGLE NONE 12
SIA C6 O6 SINGLE BEGINDASH 13
SIA C7 C8 SINGLE NONE 14
SIA C7 O7 SINGLE BEGINDASH 15
SIA C8 C9 SINGLE NONE 16
SIA C8 O8 SINGLE BEGINWEDGE 17
SIA C9 O9 SINGLE NONE 18
SIA C10 C11 SINGLE NONE 19
SIA C10 N5 SINGLE NONE 20
SIA C10 O10 DOUBLE NONE 21
#
loop_
_pdbe_chem_comp_substructure.comp_id
_pdbe_chem_comp_substructure.substructure_name
_pdbe_chem_comp_substructure.id
_pdbe_chem_comp_substructure.substructure_type
```

```
_pdbe_chem_comp_substructure.substructure_smiles
_pdbe_chem_comp_substructure.substructure_inchis
_pdbe_chem_comp_substructure.substructure_inchikeys
SIA MurckoScaffold S1 scaffold C1CCOCC1 InChI=1S/C5H10O/c1-2-4-6-5-3-
1/h1-5H2 DHXVGJBLRPWPCS-UHFFFAOYSA-N
SIA amide F1 fragment CC(N)=O InChI=1S/C2H5NO/c1-2(3)4/h1H3,(H2,3,4)
DLFVBJFMPXGRIB-UHFFFAOYSA-N
SIA pyranose F2 fragment OC1CCCCO1 InChI=1S/C5H10O2/c6-5-3-1-2-4-7-
5/h5-6H,1-4H2 CELWCAITJAEQNL-UHFFFAOYSA-N
#
loop_
_pdbe_chem_comp_substructure_mapping.comp_id
_pdbe_chem_comp_substructure_mapping.atom_id
_pdbe_chem_comp_substructure_mapping.substructure_id
_pdbe_chem_comp_substructure_mapping.substructure_ordinal
SIA C2 S1 1
SIA C3 S1 1
SIA C4 S1 1
SIA C5 S1 1
SIA C6 S1 1
SIA O6 S1 1
SIA N5 F1 1
SIA C10 F1 1
SIA O10 F1 1
SIA C11 F1 1
SIA C5 F2 1
SIA C6 F2 1
SIA O6 F2 1
SIA C2 F2 1
SIA C3 F2 1
SIA C4 F2 1
SIA O2 F2 1
#
_pdbe_chem_comp_rdkit_properties.comp_id SIA
_pdbe_chem_comp_rdkit_properties.exactmw 309.106
_pdbe_chem_comp_rdkit_properties.amw 309.271
_pdbe_chem_comp_rdkit_properties.lipinskiHBA 10
_pdbe_chem_comp_rdkit_properties.lipinskiHBD 7
_pdbe_chem_comp_rdkit_properties.NumRotatableBonds 11
_pdbe_chem_comp_rdkit_properties.NumHBD 7
_pdbe_chem_comp_rdkit_properties.NumHBA 9
_pdbe_chem_comp_rdkit_properties.NumHeavyAtoms 21
_pdbe_chem_comp_rdkit_properties.NumAtoms 40
_pdbe_chem_comp_rdkit_properties.NumHeteroatoms 10
_pdbe_chem_comp_rdkit_properties.NumAmideBonds 1
_pdbe_chem_comp_rdkit_properties.FractionCSP3 0.818
_pdbe_chem_comp_rdkit_properties.NumRings 1
_pdbe_chem_comp_rdkit_properties.NumAromaticRings 0
_pdbe_chem_comp_rdkit_properties.NumAliphaticRings 1
_pdbe_chem_comp_rdkit_properties.NumSaturatedRings 1
_pdbe_chem_comp_rdkit_properties.NumHeterocycles 1
_pdbe_chem_comp_rdkit_properties.NumAromaticHeterocycles 0
```

```
_pdbe_chem_comp_rdkit_properties.NumSaturatedHeterocycles 1
_pdbe_chem_comp_rdkit_properties.NumAliphaticHeterocycles 1
_pdbe_chem_comp_rdkit_properties.NumSpiroAtoms 0
_pdbe_chem_comp_rdkit_properties.NumBridgeheadAtoms 0
_pdbe_chem_comp_rdkit_properties.NumAtomStereoCenters 6
_pdbe_chem_comp_rdkit_properties.NumUnspecifiedAtomStereoCenters 0
_pdbe_chem_comp_rdkit_properties.labuteASA 146.407
_pdbe_chem_comp_rdkit_properties.tpsa 176.780
_pdbe_chem_comp_rdkit_properties.CrippenClogP -3.872
_pdbe_chem_comp_rdkit_properties.CrippenMR 64.787
_pdbe_chem_comp_rdkit_properties.chi0v 9.621
_pdbe_chem_comp_rdkit_properties.chi1v 4.738
_pdbe_chem_comp_rdkit_properties.chi2v 1.941
_pdbe_chem_comp_rdkit_properties.chi3v 1.941
_pdbe_chem_comp_rdkit_properties.chi4v 1.128
_pdbe_chem_comp_rdkit_properties.chi0n 28.621
_pdbe_chem_comp_rdkit_properties.chi1n 13.635
_pdbe_chem_comp_rdkit_properties.chi2n 1.941
_pdbe_chem_comp_rdkit_properties.chi3n 1.941
_pdbe_chem_comp_rdkit_properties.chi4n 1.128
_pdbe_chem_comp_rdkit_properties.hallKierAlpha -1.300
_pdbe_chem_comp_rdkit_properties.kappa1 4.600
_pdbe_chem_comp_rdkit_properties.kappa2 6.642
_pdbe_chem_comp_rdkit_properties.kappa3 3.872
_pdbe_chem_comp_rdkit_properties.Phi 1.455
#
loop_
_pdbe_chem_comp_external_mappings.comp_id
_pdbe_chem_comp_external_mappings.source
_pdbe_chem_comp_external_mappings.resource
_pdbe_chem_comp_external_mappings.resource_id
SIA UniChem ChEMBL CHEMBL1234621
SIA UniChem DrugBank DB03721
SIA UniChem ChEBI 49026
SIA UniChem ZINC ZINC000004081651
SIA UniChem eMolecules 474793
SIA UniChem fdasrs 04A90EXP8V
SIA UniChem SureChEMBL SCHEMBL79085
SIA UniChem HMDB HMDB0000773
SIA UniChem 'PubChem TPHARMA' 14776495
SIA UniChem 'PubChem TPHARMA' 15395566
SIA UniChem PubChem 444885
SIA UniChem Nikkaji J614.853K
SIA UniChem MetaboLights MTBLC49026
SIA UniChem BRENDA 141715
SIA UniChem BRENDA 233672
SIA UniChem BRENDA 6105
SIA UniChem BRENDA 84245
SIA UniChem BRENDA 85625
SIA UniChem 'Probes And Drugs' PD041137
#
loop_
```

```
_pdbe_chem_comp_rdkit_conformer.comp_id
_pdbe_chem_comp_rdkit_conformer.atom_id
_pdbe_chem_comp_rdkit_conformer.Cartn_x_rdkit
_pdbe_chem_comp_rdkit_conformer.Cartn_y_rdkit
_pdbe_chem_comp_rdkit_conformer.Cartn_z_rdkit
_pdbe_chem_comp_rdkit_conformer.rdkit_method
_pdbe_chem_comp_rdkit_conformer.rdkit_ordinal
SIA C1 3.206 -1.056 -1.015 ETKDGv3 1
SIA C2 2.493 -0.686 0.273 ETKDGv3 2
SIA C3 2.529 0.857 0.544 ETKDGv3 3
SIA C4 1.285 1.315 1.318 ETKDGv3 4
SIA C5 -0.015 0.904 0.574 ETKDGv3 5
SIA C6 0.234 -0.314 -0.366 ETKDGv3 6
SIA C7 -1.052 -1.097 -0.756 ETKDGv3 7
SIA C8 -1.784 -1.809 0.425 ETKDGv3 8
SIA C9 -3.021 -2.601 -0.043 ETKDGv3 9
SIA C10 -1.495 2.960 0.298 ETKDGv3 10
SIA C11 -1.894 4.146 -0.517 ETKDGv3 11
SIA N5 -0.512 2.045 -0.206 ETKDGv3 12
SIA O1A 3.135 -0.314 -2.032 ETKDGv3 13
SIA O1B 3.886 -2.268 -1.102 ETKDGv3 14
SIA O2 3.137 -1.336 1.344 ETKDGv3 15
SIA O4 1.308 0.822 2.637 ETKDGv3 16
SIA O6 1.167 -1.208 0.219 ETKDGv3 17
SIA O7 -1.931 -0.267 -1.477 ETKDGv3 18
SIA O8 -0.916 -2.652 1.142 ETKDGv3 19
SIA O9 -2.666 -3.625 -0.935 ETKDGv3 20
SIA O10 -2.007 2.788 1.437 ETKDGv3 21
SIA H32 3.445 1.144 1.106 ETKDGv3 22
SIA H31 2.556 1.430 -0.408 ETKDGv3 23
SIA H4 1.328 2.424 1.394 ETKDGv3 24
SIA H5 -0.768 0.631 1.340 ETKDGv3 25
SIA H6 0.650 0.061 -1.331 ETKDGv3 26
SIA H7 -0.718 -1.891 -1.459 ETKDGv3 27
SIA H8 -2.163 -1.045 1.133 ETKDGv3 28
SIA H92 -3.506 -3.063 0.843 ETKDGv3 29
SIA H91 -3.772 -1.916 -0.500 ETKDGv3 30
SIA H111 -2.794 4.632 -0.085 ETKDGv3 31
SIA H113 -2.123 3.827 -1.555 ETKDGv3 32
SIA H112 -1.060 4.878 -0.536 ETKDGv3 33
SIA HN5 -0.027 2.291 -1.099 ETKDGv3 34
SIA HO1B 4.346 -2.545 -1.960 ETKDGv3 35
SIA HO2 4.091 -1.056 1.352 ETKDGv3 36
SIA HO4 1.114 -0.150 2.611 ETKDGv3 37
SIA HO7 -2.406 0.311 -0.826 ETKDGv3 38
SIA HO8 -0.557 -3.325 0.507 ETKDGv3 39
SIA HO9 -2.722 -3.244 -1.850 ETKDGv3 40
#
```
