# Supplementary material for: Modeling glycans with AlphaFold 3: capabilities, caveats, and limitations
Source: Glycobiology. 2025 Aug 28;35(10):cwaf048. doi: 10.1093/glycob/cwaf048 (PMC12448869; doi:10.1093/glycob/cwaf048)
Supplement: Supplementary_Document_2_cwaf048 [file supplementary_document_2_cwaf048.pdf]

Supplementary Document 2
AlphaFold 3 input JSON files

Contents

Fig. 1d. LNnT 3
Fig. 1i. G2 4
Fig. 3b. M9 5
Fig. 3c. MmMAN1A1 complexed with Ca^{2+} and M9 6
Fig. 3f. HsEPO carrying M9 8
Fig. 3g. MmMAN1A1 complexed with Ca^{2+} and HsEPO carrying M9 9
Fig. 4f. GP1c 11
Fig. 4g. Lewis b 24
Fig. 4h. Bc2L-C trimer in complex with three Globo H molecules on mock lipid layer 31
Fig. 4i. HsALG1 in complex with GDP-Man, Mn^{2+} and chitobiose 39
Fig. 4j. HsGPC1 linked to GPI anchor 44
Fig. 5a. HsEPO carrying core 1 *O*-GalNAc glycan 113
Fig. 5b. HsEPO carrying core 2 *O*-GalNAc glycan 114
Fig. 5c. HsEPO carrying core 3 *O*-GalNAc glycan 115
Fig. 5d. HsEPO carrying core 4 *O*-GalNAc glycan 116
Fig. 5e. HsEPO carrying extended disialyl core 1 *O*-GalNAc glycan ... 117
Fig. 5f. HsEPO carrying extended core 2 O-GalNAc glycan & KS 124
Fig. 5g. HsGPC1 carrying HS 132
Fig. 5h. HsBikunin carrying CS 137
Fig. 5i. Hsα-DG carrying core M3 *O*-Man glycan 141
Fig. 6c and 6e. Cis-interaction of two glycosylated HsSiglec-2 146
Fig. 6f. G2S2 (GlcNAc6S) acting as trans-ligand interrupts HsSiglec-2 cis-interaction 158
Sup. Fig. 1c. G2S2 (α2,6-sia) 171
Sup. Fig. 2b. A1 178
Sup. Fig. 2c. HsMGAT2 complexed with Mn^{2+}, UDP-GlcNAc and A1 179
Sup. Fig. 2f. HsEPO carrying A1 181
Sup. Fig. 2g. HsMGAT2 complexed with Mn^{2+}, UDP-GlcNAc and HsEPO carrying A1 182
Sup. Fig. 3b. G2F 184
Sup. Fig. 3c. *N*-glycosylated HsST6GAL1 complexed with CMP-Sia and G2F 185
Sup. Fig. 3f. HsEPO carrying G2F 187
Sup. Fig. 3g. HsST6GAL1 complexed with CMP-Sia and HsEPO carrying G2F 188
Sup. Fig. 4. HsB3GALT5 complexed with Core 3 O-GalNAc 190
Sup. Fig. 5. PmHS2 complexed with HS 5-mer 191
Sup. Fig. 6. HsAggrecan G1 complexed with HA 10-mer 193
Sup. Fig. 7. CsPP2 complexed with chitotriose 194
Sup. Fig. 8. BT1258 D161A/E163A complexed with M9 195
Sup. Fig. 9. BoPL38 complexed with M4 alginate 196
Sup. Fig. 10. PtGlucanase complexed with laminarin 197
Sup. Fig. 11. BcXylanase E78Q complexed with xylotriose 198
Sup. Fig. 12. EcFimH complexed with M3F 199
Sup. Fig. 12. BoNT/A Y1117V complexed with GM1b 200
Template 2. A2F 202

Template 3. A3 (3-arm) 203
Template 4. A3F (3-arm) 204
Template 5. A3 (6-arm) 205
Template 6. A3F (6-arm) 206
Template 7. A4 (3- and 6-arm) 207
Template 8. A4F (3- and 6-arm) 208
Template 9. G2F 209
Template 10. G2S2 (α2,3-sia) 210
Template 11. G2S2F (α2,3-sia) 218
Template 12. G2S2F (α2,6-sia) 226
Template 13. M3 234
Template 14. M5 235
Template 15. M5F 236

Fig. 1d. LNnT


```
{
    "name": "lnnt",
    "modelSeeds": [
      1
    ],
    "sequences": [
      {
        "ligand": {
          "ccdCodes": [
            "BGC", "GAL", "NAG", "GAL"
          ],
          "id": "LNNT"
        }
      }
    ],
    "dialect": "alphafold3",
    "version": 2,
    "bondedAtomPairs": [
    [["LNNT",1,"O4"],["LNNT",2,"C1"]],
    [["LNNT",2,"O3"],["LNNT",3,"C1"]],
    [["LNNT",3,"O4"],["LNNT",4,"C1"]]
    ]
  }
```

Fig. 1i. G2


```
{
    "name": "g2",
    "modelSeeds": [
      1
    ],
    "sequences": [
      {
        "ligand": {
          "ccdCodes": [
            "NAG", "NAG", "BMA", "MAN", "MAN", "NAG", "NAG", "GAL",
"GAL"
          ],
          "id": "NG"
        }
      }
    ],
    "dialect": "alphafold3",
    "version": 2,
    "bondedAtomPairs": [
    [["NG",1,"O4"],["NG",2,"C1"]],
    [["NG",2,"O4"],["NG",3,"C1"]],
    [["NG",3,"O3"],["NG",4,"C1"]],
    [["NG",3,"O6"],["NG",5,"C1"]],
    [["NG",4,"O2"],["NG",6,"C1"]],
    [["NG",5,"O2"],["NG",7,"C1"]],
    [["NG",6,"O4"],["NG",8,"C1"]],
    [["NG",7,"O4"],["NG",9,"C1"]]
    ]
  }
```

Fig. 3b. M9

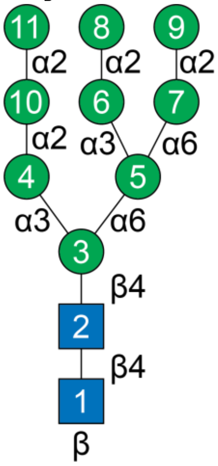


```
{
    "name": "m9",
    "modelSeeds": [
      1
    ],
    "sequences": [
      {
        "ligand": {
          "ccdCodes": [
            "NAG", "NAG", "BMA", "MAN", "MAN", "MAN", "MAN", "MAN",
"MAN", "MAN", "MAN"
          ],
          "id": "NG"
        }
      }
    ],
    "dialect": "alphafold3",
    "version": 2,
    "bondedAtomPairs": [
    [["NG",1,"O4"],["NG",2,"C1"]],
    [["NG",2,"O4"],["NG",3,"C1"]],
    [["NG",3,"O3"],["NG",4,"C1"]],
    [["NG",3,"O6"],["NG",5,"C1"]],
    [["NG",5,"O3"],["NG",6,"C1"]],
    [["NG",5,"O6"],["NG",7,"C1"]],
    [["NG",6,"O2"],["NG",8,"C1"]],
    [["NG",7,"O2"],["NG",9,"C1"]],
    [["NG",4,"O2"],["NG",10,"C1"]],
    [["NG",10,"O2"],["NG",11,"C1"]]
    ]
  }
```

Fig. 3c. MmMAN1A1 complexed with Ca^{2+} and M9


```
{
    "name": "golgiman1a1_m9_ca",
    "modelSeeds": [
      1
    ],
    "sequences": [
      {
        "ligand": {
          "ccdCodes": [
            "NAG", "NAG", "BMA", "MAN", "MAN", "MAN", "MAN", "MAN",
"MAN", "MAN", "MAN"
          ],
          "id": "NG"
        }
      },
      {
        "ligand": {
          "ccdCodes": [
            "CA"
          ],
          "id": "CA"
        }
      },
      {
        "protein": {
          "sequence":
"MPVGGLLPLFSSPGGGGLGSGLGGGLGGGRKGSGPAAFRLTEKFVLLLVFSAFITLCFGAIFFLPDSSK
LLSGVLFHSNPALQPPAEHKPGLGARAEDAAEGRVRHREEGAPGDPGAGLEDNLARIRENHERALREAKE
TLQKLPEEIQRDILLEKEKVAQDQLRDKDLFRGLPKVDFLPPVGVENREPADATIREKRAKIKEMMTHAW
NNYKRYAWGLNELKPISKEGHSSSLFGNIKGATIVDALDTLFIMGMKTEFQEAKSWIKKYLDFNVNAEVS
VFEVNIRFVGGLLSAYYLSGEEIFRKKAVELGVKLLPAFHTPSGIPWALLNMKSGIGRNWPWASGGSSIL
AEFGTLHLEFMHLSHLSGDPVFAEKVMKIRTVLNKLDKPEGLYPNYLNPSSGQWGQHHVSVGGLGDSFYE
YLLKAWLMSDKTDLEAKKMYFDAVQAIETHLIRKSSGGLTYIAEWKGGLLEHKMGHLTCFAGGMFALGAD
GAPEARAQHYLELGAEIARTCHESYNRTYVKLGPEAFRFDGGVEAIATRQNEKYYILRPEVIETYMYMWR
LTHDPKYRTWAWEAVEALESHCRVNGGYSGLRDVYIARESYDDVQQSFFLAETLKYLYLIFSDDDLLPLE
HWIFNTEAHPFPILREQKKEIDGKEK",
          "id": "GGMAN"
        }
      }
    ],
    "dialect": "alphafold3",
    "version": 2,
    "bondedAtomPairs": [
    [["NG",1,"O4"],["NG",2,"C1"]],
    [["NG",2,"O4"],["NG",3,"C1"]],
    [["NG",3,"O3"],["NG",4,"C1"]],
    [["NG",3,"O6"],["NG",5,"C1"]],
    [["NG",5,"O3"],["NG",6,"C1"]],
    [["NG",5,"O6"],["NG",7,"C1"]],
    [["NG",6,"O2"],["NG",8,"C1"]],
    [["NG",7,"O2"],["NG",9,"C1"]],
```

```
  [["NG",4,"O2"],["NG",10,"C1"]],
  [["NG",10,"O2"],["NG",11,"C1"]]
  ]
}
```

Fig. 3f. HsEPO carrying M9


```
{
    "name": "epo_m9",
    "modelSeeds": [
      1
    ],
    "sequences": [
      {
        "protein": {
          "sequence":
"MGVHECPAWLWLLLSLLSLPLGLPVLGAPPRLICDSRVLERYLLEAKEAENITTGCAEHCSLNENITVP
DTKVNFYAWKRMEVGQQAVEVWQGLALLSEAVLRGQALLVNSSQPWEPLQLHVDKAVSGLRSLTTLLRAL
GAQKEAISPPDAASAAPLRTITADTFRKLFRVYSNFLRGKLKLYTGEACRTGDR",
          "id": "EPO"
        }
      },
      {
        "ligand": {
          "ccdCodes": [
            "NAG", "NAG", "BMA", "MAN", "MAN", "MAN", "MAN", "MAN",
"MAN", "MAN", "MAN"
          ],
          "id": "NG"
        }
      }
    ],
    "dialect": "alphafold3",
    "version": 2,
    "bondedAtomPairs": [
    [["EPO",51,"ND2"],["NG",1,"C1"]],
    [["NG",1,"O4"],["NG",2,"C1"]],
    [["NG",2,"O4"],["NG",3,"C1"]],
    [["NG",3,"O3"],["NG",4,"C1"]],
    [["NG",3,"O6"],["NG",5,"C1"]],
    [["NG",5,"O3"],["NG",6,"C1"]],
    [["NG",5,"O6"],["NG",7,"C1"]],
    [["NG",6,"O2"],["NG",8,"C1"]],
    [["NG",7,"O2"],["NG",9,"C1"]],
    [["NG",4,"O2"],["NG",10,"C1"]],
    [["NG",10,"O2"],["NG",11,"C1"]]
    ]
  }
```

Fig. 3g. MmMAN1A1 complexed with Ca^{2+} and HsEPO carrying M9


```
{
    "name": "golgiman1a1_epo_m9_ca",
    "modelSeeds": [
      1
    ],
    "sequences": [
      {
        "protein": {
          "sequence":
"MGVHECPAWLWLLLSLLSLPLGLPVLGAPPRLICDSRVLERYLLEAKEAENITTGCAEHCSLNENITVP
DTKVNFYAWKRMEVGQQAVEVWQGLALLSEAVLRGQALLVNSSQPWEPLQLHVDKAVSGLRSLTTLLRAL
GAQKEAISPPDAASAAPLRTITADTFRKLFRVYSNFLRGKLKLYTGEACRTGDR",
          "id": "EPO"
        }
      },
      {
        "ligand": {
          "ccdCodes": [
            "NAG", "NAG", "BMA", "MAN", "MAN", "MAN", "MAN", "MAN",
"MAN", "MAN", "MAN"
          ],
          "id": "NG"
        }
      },
      {
        "ligand": {
          "ccdCodes": [
            "CA"
          ],
          "id": "CA"
        }
      },
      {
        "protein": {
          "sequence":
"MPVGGLLPLFSSPGGGGLGSGLGGGLGGGRKGSGPAAFRLTEKFVLLLVFSAFITLCFGAIFFLPDSSK
LLSGVLFHSNPALQPPAEHKPGLGARAEDAAEGRVRHREEGAPGDPGAGLEDNLARIRENHERALREAKE
TLQKLPEEIQRDILLEKEKVAQDQLRDKDLFRGLPKVDFLPPVGVENREPADATIREKRAKIKEMMTHAW
NNYKRYAWGLNELKPISKEGHSSSLFGNIKGATIVDALDTLFIMGMKTEFQEAKSWIKKYLDFNVNAEVS
VFEVNIRFVGGLLSAYYLSGEEIFRKKAVELGVKLLPAFHTPSGIPWALLNMKSGIGRNWPWASGGSSIL
AEFGTLHLEFMHLSHLSGDPVFAEKVMKIRTVLNKLDKPEGLYPNYLNPSSGQWGQHHVSVGGLGDSFYE
YLLKAWLMSDKTDLEAKKMYFDAVQAIETHLIRKSSGGLTYIAEWKGGLLEHKMGHLTCFAGGMFALGAD
GAPEARAQHYLELGAEIARTCHESYNRTYVKLGPEAFRFDGGVEAIATRQNEKYYILRPEVIETYMYMWR
LTHDPKYRTWAWEAVEALESHCRVNGGYSGLRDVYIARESYDDVQQSFFLAETLKYLYLIFSDDDLLPLE
HWIFNTEAHPFPILREQKKEIDGKEK",
          "id": "GGMAN"
        }
      }
    ],
    "dialect": "alphafold3",
    "version": 2,
```

```
  "bondedAtomPairs": [
  [["EPO",51,"ND2"],["NG",1,"C1"]],
  [["NG",1,"O4"],["NG",2,"C1"]],
  [["NG",2,"O4"],["NG",3,"C1"]],
  [["NG",3,"O3"],["NG",4,"C1"]],
  [["NG",3,"O6"],["NG",5,"C1"]],
  [["NG",5,"O3"],["NG",6,"C1"]],
  [["NG",5,"O6"],["NG",7,"C1"]],
  [["NG",6,"O2"],["NG",8,"C1"]],
  [["NG",7,"O2"],["NG",9,"C1"]],
  [["NG",4,"O2"],["NG",10,"C1"]],
  [["NG",10,"O2"],["NG",11,"C1"]]
  ]
}
```

Fig. 4f. GP1c


```
{
    "name": "gp1c",
    "modelSeeds": [
      1
    ],
    "sequences": [
      {
        "ligand": {
          "ccdCodes": [
            "SPH", "STE2", "BGC", "GAL", "NGA", "GAL", "SIA2", "SIA2",
"SIA2", "SIA2", "SIA2"
          ],
          "id": "GSL"
        }
      }
    ],
    "dialect": "alphafold3",
    "version": 2,
    "bondedAtomPairs": [
    [["GSL",1,"N2"],["GSL",2,"C1"]],
    [["GSL",1,"O1"],["GSL",3,"C1"]],
    [["GSL",3,"O4"],["GSL",4,"C1"]],
    [["GSL",4,"O4"],["GSL",5,"C1"]],
    [["GSL",5,"O3"],["GSL",6,"C1"]],
    [["GSL",4,"O3"],["GSL",7,"C2"]],
    [["GSL",7,"O8"],["GSL",8,"C2"]],
    [["GSL",8,"O8"],["GSL",9,"C2"]],
    [["GSL",6,"O3"],["GSL",10,"C2"]],
    [["GSL",10,"O8"],["GSL",11,"C2"]]
    ],
    "userCCD": "data_SIA2\n#\n_chem_comp.id SIA\n_chem_comp.name 'N-
acetyl-alpha-neuraminic acid'\n_chem_comp.type 'D-saccharide, alpha
linking'\n_chem_comp.pdbx_type ATOMS\n_chem_comp.formula 'C11 H19 N
O9'\n_chem_comp.mon_nstd_parent_comp_id ?\n_chem_comp.pdbx_synonyms
'N-acetylneuraminic acid; sialic acid; alpha-sialic acid; O-SIALIC
ACID'\n_chem_comp.pdbx_formal_charge 0\n_chem_comp.pdbx_initial_date
1999-07-08\n_chem_comp.pdbx_modified_date 2024-09-
27\n_chem_comp.pdbx_ambiguous_flag N\n_chem_comp.pdbx_release_status
REL\n_chem_comp.pdbx_replaced_by ?\n_chem_comp.pdbx_replaces
NAN\n_chem_comp.formula_weight
309.270\n_chem_comp.one_letter_code ?\n_chem_comp.three_letter_code
SIA\n_chem_comp.pdbx_model_coordinates_details ?\n_chem_comp.pdbx_mode
l_coordinates_missing_flag
N\n_chem_comp.pdbx_ideal_coordinates_details ?\n_chem_comp.pdbx_ideal_
coordinates_missing_flag
N\n_chem_comp.pdbx_model_coordinates_db_code ?\n_chem_comp.pdbx_subcom
ponent_list ?\n_chem_comp.pdbx_processing_site
EBI\n_chem_comp.pdbx_pcm
Y\n#\nloop_\n_pdbx_chem_comp_synonyms.ordinal\n_pdbx_chem_comp_synonym
s.comp_id\n_pdbx_chem_comp_synonyms.name\n_pdbx_chem_comp_synonyms.pro
venance\n_pdbx_chem_comp_synonyms.type\n1 SIA 'N-acetylneuraminic
```

acid' PDB ?\n2 SIA 'sialic acid' PDB ?\n3 SIA 'alpha-sialic acid'
PDB ?\n4 SIA 'O-SIALIC ACID'
PDB ?\n#\nloop_\n_chem_comp_atom.comp_id\n_chem_comp_atom.atom_id\n_chem_comp_atom.alt_atom_id\n_chem_comp_atom.type_symbol\n_chem_comp_atom.charge\n_chem_comp_atom.pdbx_align\n_chem_comp_atom.pdbx_aromatic_flag\n_chem_comp_atom.pdbx_leaving_atom_flag\n_chem_comp_atom.pdbx_stereo_config\n_chem_comp_atom.pdbx_backbone_atom_flag\n_chem_comp_atom.pdbx_n_terminal_atom_flag\n_chem_comp_atom.pdbx_c_terminal_atom_flag\n_chem_comp_atom.model_Cartn_x\n_chem_comp_atom.model_Cartn_y\n_chem_comp_atom.model_Cartn_z\n_chem_comp_atom.pdbx_model_Cartn_x_ideal\n_chem_comp_atom.pdbx_model_Cartn_y_ideal\n_chem_comp_atom.pdbx_model_Cartn_z_ideal\n_chem_comp_atom.pdbx_component_atom_id\n_chem_comp_atom.pdbx_component_comp_id\n_chem_comp_atom.pdbx_ordinal\nSIA C1 C1 C 0 1 N N N N N N -2.196 58.872 -5.981 -2.502 -0.832 0.174 C1 SIA 1\nSIA C2 C2 C 0 1 N N R N N N -1.870 58.021 -7.211 -2.171 0.628 0.342 C2 SIA 2\nSIA C3 C3 C 0 1 N N N N N N -0.844 56.899 -7.306 -1.789 0.898 1.800 C3 SIA 3\nSIA C4 C4 C 0 1 N N S N N N -1.157 55.904 -8.413 -0.586 0.023 2.171 C4 SIA 4\nSIA C5 C5 C 0 1 N N R N N N -2.015 56.516 -9.517 0.529 0.264 1.148 C5 SIA 5\nSIA C6 C6 C 0 1 N N R N N N -3.352 56.956 -8.912 -0.026 0.043 -0.259 C6 SIA 6\nSIA C7 C7 C 0 1 N N R N N N -4.224 57.698 -9.942 1.088 0.251 -1.286 C7 SIA 7\nSIA C8 C8 C 0 1 N N R N N N -5.571 58.131 -9.360 0.535 0.021 -2.694 C8 SIA 8\nSIA C9 C9 C 0 1 N N N N N N -6.601 58.674 -10.381 1.650 0.229 -3.721 C9 SIA 9\nSIA C10 C10 C 0 1 N N N N N N -1.897 55.374 -11.759 2.632 -0.329 2.226 C10 SIA 10\nSIA C11 C11 C 0 1 N N N N N N -2.200 54.057 -12.454 3.763 -1.292 2.478 C11 SIA 11\nSIA N5 N5 N 0 1 N N N N N N -2.202 55.444 -10.478 1.629 -0.671 1.394 N5 SIA 12\nSIA O1A O1A O 0 1 N N N N N N -1.289 58.815 -5.130 -2.191 -1.408 -0.841 O1A SIA 13\nSIA O1B O1B O 0 1 N N N N N N -3.210 59.504 -5.631 -3.141 -1.493 1.152 O1B SIA 14\nSIA O4 O4 O 0 1 N N N N N N 0.072 55.523 -8.986 -0.123 0.370 3.478 O4 SIA 16\nSIA O6 O6 O 0 1 N N N N N N -3.149 57.908 -7.847 -1.082 0.968 -0.513 O6 SIA 17\nSIA O7 O7 O 0 1 N N N N N N -3.594 58.883 -10.402 1.588 1.586 -1.183 O7 SIA 18\nSIA O8 O8 O 0 1 N N N N N N -6.119 56.946 -8.828 0.035 -1.313 -2.797 O8 SIA 19\nSIA O9 O9 O 0 1 N N N N N N -6.931 57.687 -11.346 1.133 0.014 -5.035 O9 SIA 20\nSIA O10 O10 O 0 1 N N N N N N -1.423 56.357 -12.331 2.624 0.753 2.772 O10 SIA 21\nSIA H32 H31 H 0 1 N N N N N N -0.702 56.484 -6.300 -2.631 0.655 2.448 H32 SIA 22\nSIA H31 H32 H 0 1 N N N N N N 0.120 57.408 -7.182 -1.526 1.949 1.919 H31 SIA 23\nSIA H4 H4 H 0 1 N N N N N N -1.651 55.060 -7.897 -0.878 -1.026 2.153 H4 SIA 24\nSIA H5 H5 H 0 1 N N N N N N -1.506 57.375 -9.979 0.893 1.287 1.240 H5 SIA 25\nSIA H6 H6 H 0 1 N N N N N N -3.850 56.075 -8.492 -0.408 -0.973 -0.341 H6 SIA 26\nSIA H7 H7 H 0 1 N N N N N N -4.339 57.176 -10.907 1.896 -0.454 -1.093 H7 SIA 27\nSIA H8 H8 H 0 1 N N N N N N -5.473 58.871 -8.553 -0.272 0.728 -2.887 H8 SIA 28\nSIA H92 H91 H 0 1 N N N N N N -6.054 59.459 -10.925 2.031 1.247 -3.642 H92 SIA 29\nSIA H91 H92 H 0 1 N N N N N N -7.587 59.029 -10.055 2.457 -0.476 -3.528 H91 SIA 30\nSIA H111 H111 H 0 0 N N N N N N -3.215 53.728 -12.207 4.474 -0.844 3.172 H111 SIA 31\nSIA H113 H112 H 0 0 N N N N N N -1.550 53.279 -12.033 3.368 -2.213 2.907 H113 SIA 32\nSIA H112 H113 H 0 0 N N N N N N -2.005 54.041 -13.531 4.266 -1.516 1.537 H112 SIA 33\nSIA HN5 HN5 H 0 1 N N N N N N -2.566 54.658 -10.003 1.635 -1.538 0.957 HN5 SIA 34\nSIA HO1B HOB1 H 0 0 N N N N N N -3.412 60.032 -4.867

-3.353 -2.430 1.044 HO1B SIA 35\nSIA HO4 HO4 H 0 1 N Y N N N N 0.427 54.801 -8.430 -0.854 0.203 4.087 HO4 SIA 37\nSIA HO7 HO7 H 0 1 N Y N N N N -3.109 58.884 -9.548 0.844 2.177 -1.360 HO7 SIA 38\nSIA HO8 HO8 H 0 1 N Y N N N N -7.071 57.051 -8.962 0.779 -1.904 -2.620 HO8 SIA 39\nSIA HO9 HO9 H 0 1 N Y N N N N -6.783 56.885 -10.808 1.866 0.155 -5.650 HO9 SIA
40\n#\nloop_\n_chem_comp_bond.comp_id\n_chem_comp_bond.atom_id_1\n_chem_comp_bond.atom_id_2\n_chem_comp_bond.value_order\n_chem_comp_bond.pdbx_aromatic_flag\n_chem_comp_bond.pdbx_stereo_config\n_chem_comp_bond.pdbx_ordinal\nSIA C1 C2 SING N N 1\nSIA C1 O1A DOUB N N 2\nSIA C1 O1B SING N N 3\nSIA C2 C3 SING N N 4\nSIA C2 O6 SING N N 6\nSIA C3 C4 SING N N 7\nSIA C3 H32 SING N N 8\nSIA C3 H31 SING N N 9\nSIA C4 C5 SING N N 10\nSIA C4 O4 SING N N 11\nSIA C4 H4 SING N N 12\nSIA C5 C6 SING N N 13\nSIA C5 N5 SING N N 14\nSIA C5 H5 SING N N 15\nSIA C6 C7 SING N N 16\nSIA C6 O6 SING N N 17\nSIA C6 H6 SING N N 18\nSIA C7 C8 SING N N 19\nSIA C7 O7 SING N N 20\nSIA C7 H7 SING N N 21\nSIA C8 C9 SING N N 22\nSIA C8 O8 SING N N 23\nSIA C8 H8 SING N N 24\nSIA C9 O9 SING N N 25\nSIA C9 H92 SING N N 26\nSIA C9 H91 SING N N 27\nSIA C10 C11 SING N N 28\nSIA C10 N5 SING N N 29\nSIA C10 O10 DOUB N N 30\nSIA C11 H111 SING N N 31\nSIA C11 H113 SING N N 32\nSIA C11 H112 SING N N 33\nSIA N5 HN5 SING N N 34\nSIA O1B HO1B SING N N 35\nSIA O4 HO4 SING N N 37\nSIA O7 HO7 SING N N 38\nSIA O8 HO8 SING N N 39\nSIA O9 HO9 SING N N
40\n#\nloop_\n_pdbx_chem_comp_descriptor.comp_id\n_pdbx_chem_comp_descriptor.type\n_pdbx_chem_comp_descriptor.program\n_pdbx_chem_comp_descriptor.program_version\n_pdbx_chem_comp_descriptor.descriptor\nSIA SMILES ACDLabs 10.04 'O=C(O)C1(O)OC(C(O)C(O)CO)C(NC(=O)C)C(O)C1'\nSIA SMILES_CANONICAL CACTVS 3.341
'CC(=O)N[C@@H]1[C@@H](O)C[C@@](O)(O[C@H]1[C@H](O)[C@H](O)CO)C(O)=O'\nSIA SMILES CACTVS 3.341
'CC(=O)N[CH]1[CH](O)C[C](O)(O[CH]1[CH](O)[CH](O)CO)C(O)=O'\nSIA SMILES_CANONICAL 'OpenEye OEToolkits' 1.5.0
'CC(=O)N[C@@H]1[C@H](C[C@@](O[C@H]1[C@@H]([C@@H](CO)O)O)(C(=O)O)O)O'\nSIA SMILES 'OpenEye OEToolkits' 1.5.0
'CC(=O)NC1C(CC(OC1C(C(CO)O)O)(C(=O)O)O)O'\nSIA InChI InChI 1.03 'InChI=1S/C11H19NO9/c1-4(14)12-7-5(15)2-11(20,10(18)19)21-9(7)8(17)6(16)3-13/h5-9,13,15-17,20H,2-3H2,1H3,(H,12,14)(H,18,19)/t5-,6+,7+,8+,9+,11+/m0/s1'\nSIA InChIKey InChI 1.03 SQVRNKJHWKZAKO-YRMXFSIDSA-N\n#\nloop_\n_pdbx_chem_comp_identifier.comp_id\n_pdbx_chem_comp_identifier.type\n_pdbx_chem_comp_identifier.program\n_pdbx_chem_comp_identifier.program_version\n_pdbx_chem_comp_identifier.identifier\nSIA 'SYSTEMATIC NAME' ACDLabs 10.04 '5-(acetylamino)-3,5-dideoxy-D-glycero-alpha-D-galacto-non-2-ulopyranosonic acid'\nSIA 'SYSTEMATIC NAME' 'OpenEye OEToolkits' 1.5.0 '(2R,4S,5R,6R)-5-acetamido-2,4-dihydroxy-6-[(1R,2R)-1,2,3-trihydroxypropyl]oxane-2-carboxylic acid'\nSIA 'CONDENSED IUPAC CARBOHYDRATE SYMBOL' GMML 1.0 DNeup5Aca\nSIA 'COMMON NAME' GMML 1.0 'N-acetyl-a-D-neuraminic acid'\nSIA 'IUPAC CARBOHYDRATE SYMBOL' PDB-CARE 1.0 a-D-Neup5Ac\nSIA 'SNFG CARBOHYDRATE SYMBOL' GMML 1.0
Neu5Ac\n#\nloop_\n_pdbx_chem_comp_feature.comp_id\n_pdbx_chem_comp_feature.type\n_pdbx_chem_comp_feature.value\n_pdbx_chem_comp_feature.sour

ce\n_pdbx_chem_comp_feature.support\nSIA 'CARBOHYDRATE ISOMER' D PDB ?\nSIA 'CARBOHYDRATE RING' pyranose PDB ?\nSIA 'CARBOHYDRATE ANOMER' alpha PDB ?\nSIA 'CARBOHYDRATE PRIMARY CARBONYL GROUP' ketose PDB ?\n#\nloop_\n_pdbx_chem_comp_audit.comp_id\n_pdbx_chem_comp_audit.action_type\n_pdbx_chem_comp_audit.date\n_pdbx_chem_comp_audit.processing_site\nSIA 'Create component' 1999-07-08 EBI\nSIA 'Modify descriptor' 2011-06-04 RCSB\nSIA 'Other modification' 2019-08-12 RCSB\nSIA 'Other modification' 2019-12-19 RCSB\nSIA 'Other modification' 2020-07-03 RCSB\nSIA 'Modify name' 2020-07-17 RCSB\nSIA 'Modify synonyms' 2020-07-17 RCSB\nSIA 'Modify atom id' 2020-07-17 RCSB\nSIA 'Modify component atom id' 2020-07-17 RCSB\nSIA 'Modify PCM' 2024-09-27 PDBE\n#\n_pdbx_chem_comp_pcm.pcm_id 1\n_pdbx_chem_comp_pcm.comp_id SIA\n_pdbx_chem_comp_pcm.modified_residue_id THR\n_pdbx_chem_comp_pcm.type None\n_pdbx_chem_comp_pcm.category Carbohydrate\n_pdbx_chem_comp_pcm.position 'Amino-acid side chain'\n_pdbx_chem_comp_pcm.polypeptide_position 'Any position'\n_pdbx_chem_comp_pcm.comp_id_linking_atom C2\n_pdbx_chem_comp_pcm.modified_residue_id_linking_atom OG1\n_pdbx_chem_comp_pcm.uniprot_specific_ptm_accession ?\n_pdbx_chem_comp_pcm.uniprot_generic_ptm_accession ?\n#\n_pdbe_chem_comp_drugbank_details.comp_id SIA\n_pdbe_chem_comp_drugbank_details.drugbank_id DB03721\n_pdbe_chem_comp_drugbank_details.type 'small molecule'\n_pdbe_chem_comp_drugbank_details.name 'N-acetyl-alpha-neuraminic acid'\n_pdbe_chem_comp_drugbank_details.description\n'An N-acyl derivative of neuraminic acid. N-acetylneuraminic acid occurs in many polysaccharides, glycoproteins, and glycolipids in animals and bacteria. (From Dorland, 28th ed, p1518)'\n_pdbe_chem_comp_drugbank_details.cas_number 21646-00-4\n_pdbe_chem_comp_drugbank_details.mechanism_of_action ?\n#\nloop_\n_pdbe_chem_comp_synonyms.comp_id\n_pdbe_chem_comp_synonyms.name\n_pdbe_chem_comp_synonyms.provenance\n_pdbe_chem_comp_synonyms.type\nSIA 'N-acetylneuraminic acid' wwPDB ?\nSIA 'sialic acid' wwPDB ?\nSIA 'alpha-sialic acid' wwPDB ?\nSIA 'O-SIALIC ACID' wwPDB ?\nSIA 'N-Acetyl-alpha-D-neuraminic acid' DrugBank ?\nSIA 'O-sialic acid' DrugBank ?\nSIA 'α-Neu5Ac' DrugBank ?\n#\n_pdbe_chem_comp_drugbank_classification.comp_id SIA\n_pdbe_chem_comp_drugbank_classification.drugbank_id DB03721\n_pdbe_chem_comp_drugbank_classification.parent 'N-acylneuraminic acids'\n_pdbe_chem_comp_drugbank_classification.kingdom 'Organic compounds'\n_pdbe_chem_comp_drugbank_classification.class 'Organooxygen compounds'\n_pdbe_chem_comp_drugbank_classification.superclass 'Organic oxygen compounds'\n_pdbe_chem_comp_drugbank_classification.description\n'This compound belongs to the class of organic compounds known as n-acylneuraminic acids. These are neuraminic acids carrying an N-acyl substituent.'\n#\nloop_\n_pdbe_chem_comp_drugbank_targets.comp_id\n_pdbe_chem_comp_drugbank_targets.drugbank_id\n_pdbe_chem_comp_drugbank_targets.name\n_pdbe_chem_comp_drugbank_targets.organism\n_pdbe_chem_comp_drugbank_targets.uniprot_id\n_pdbe_chem_comp_drugbank_targets.pharmacologically_active\n_pdbe_chem_comp_drugbank_targets.ordinal\nSIA

DB03721 P-selectin Humans P16109 yes 1\nSIA DB03721 E-selectin Humans P16581 yes 2\nSIA DB03721 'Liver carboxylesterase 1' Humans P23141 yes 3\nSIA DB03721 '3-deoxy-manno-octulosonate cytidylyltransferase' 'Escherichia coli' P42216 unknown 4\nSIA DB03721 'Tetanus toxin' 'Clostridium tetani (strain Massachusetts / E88)' P04958 unknown 5\nSIA DB03721 'Cholera enterotoxin subunit B' 'Vibrio cholerae serotype O1 (strain ATCC 39315 / El Tor Inaba N16961)' P01556 unknown 6\nSIA DB03721 'Botulinum neurotoxin type B' 'Clostridium botulinum' P10844 unknown 7\nSIA DB03721 'Mannose-binding protein C' Humans P11226 unknown 8\nSIA DB03721 Lithostathine-1-alpha Humans P05451 unknown 9\nSIA DB03721 Endo-N-acetylneuraminidase 'Enterobacteria phage K1F' Q04830 unknown 10\nSIA DB03721 'Enterotoxin type B' 'Staphylococcus aureus' P01552 unknown 11\nSIA DB03721 Neuraminidase 'Influenza A virus (strain A/Tern/Australia/G70C/1975 H11N9)' P03472 unknown 12\nSIA DB03721 Hemagglutinin-neuraminidase NDV P32884 unknown 13\nSIA DB03721 Fiber 'Human adenovirus 19' Q64822 unknown 14\nSIA DB03721 Sialoadhesin Humans Q9BZZ2 unknown 15\nSIA DB03721 Zinc-alpha-2-glycoprotein Humans P25311 unknown 16\nSIA DB03721 'Capsid protein VP1' MPyV P49302 unknown 17\nSIA DB03721 Fiber 'Human adenovirus D37' Q64823 unknown 18\n#\nloop_\n_software.name\n_software.version\n_software.description\nrdkit 2023.09.6 'Core functionality.'\npdbeccdutils 0.8.6 'Wrapper to provide 2D templates and molecular fragments.'\n#\nloop_\n_pdbe_chem_comp_atom_depiction.comp_id\n_pdbe_chem_comp_atom_depiction.atom_id\n_pdbe_chem_comp_atom_depiction.element\n_pdbe_chem_comp_atom_depiction.model_Cartn_x\n_pdbe_chem_comp_atom_depiction.model_Cartn_y\n_pdbe_chem_comp_atom_depiction.pdbx_ordinal\nSIA C1 C 5.654 -3.375 1\nSIA C2 C 6.404 -2.076 2\nSIA C3 C 5.104 -1.326 3\nSIA C4 C 5.104 0.174 4\nSIA C5 C 6.404 0.924 5\nSIA C6 C 7.702 0.174 6\nSIA C7 C 9.002 0.924 7\nSIA C8 C 10.301 0.174 8\nSIA C9 C 11.600 0.924 9\nSIA C10 C 5.104 3.174 10\nSIA C11 C 3.805 2.424 11\nSIA N5 N 6.404 2.424 12\nSIA O1A O 4.154 -3.375 13\nSIA O1B O 6.404 -4.674 14\nSIA O4 O 3.805 0.924 16\nSIA O6 O 7.702 -1.326 17\nSIA O7 O 9.002 2.424 18\nSIA O8 O 10.301 -1.326 19\nSIA O9 O 12.899 0.174 20\nSIA O10 O 5.104 4.674 21\n#\nloop_\n_pdbe_chem_comp_bond_depiction.comp_id\n_pdbe_chem_comp_bond_depiction.atom_id_1\n_pdbe_chem_comp_bond_depiction.atom_id_2\n_pdbe_chem_comp_bond_depiction.value_order\n_pdbe_chem_comp_bond_depiction.bond_dir\n_pdbe_chem_comp_bond_depiction.pdbx_ordinal\nSIA C1 C2 SINGLE NONE 1\nSIA C1 O1A DOUBLE NONE 2\nSIA C1 O1B SINGLE NONE 3\nSIA C2 C3 SINGLE NONE 4\nSIA C2 O6 SINGLE NONE 6\nSIA C3 C4 SINGLE NONE 7\nSIA C4 C5 SINGLE NONE 8\nSIA C4 O4 SINGLE BEGINDASH 9\nSIA C5 C6 SINGLE NONE 10\nSIA C5 N5 SINGLE BEGINWEDGE 11\nSIA C6 C7 SINGLE NONE 12\nSIA C6 O6 SINGLE BEGINDASH 13\nSIA C7 C8 SINGLE NONE 14\nSIA C7 O7 SINGLE BEGINDASH 15\nSIA C8 C9 SINGLE NONE 16\nSIA C8 O8 SINGLE BEGINWEDGE 17\nSIA C9 O9 SINGLE NONE 18\nSIA C10 C11 SINGLE NONE 19\nSIA C10 N5 SINGLE NONE 20\nSIA C10 O10 DOUBLE NONE 21\n#\nloop_\n_pdbe_chem_comp_substructure.comp_id\n_pdbe_chem_comp_substructure.substructure_name\n_pdbe_chem_comp_substructure.id\n_pdbe_chem_comp_substructure.substructure_type\n_pdbe_chem_comp_substructure.substructure_smiles\n_pdbe_chem_comp_substructure.substructure_inchis\n_pdbe_chem_comp_substructure.substructure_inchikeys\nSIA

MurckoScaffold S1 scaffold C1CCOCC1 InChI=1S/C5H10O/c1-2-4-6-5-3-1/h1-5H2 DHXVGJBLRPWPCS-UHFFFAOYSA-N\nSIA amide F1 fragment CC(N)=O InChI=1S/C2H5NO/c1-2(3)4/h1H3,(H2,3,4) DLFVBJFMPXGRIB-UHFFFAOYSA-N\nSIA pyranose F2 fragment OC1CCCCO1 InChI=1S/C5H10O2/c6-5-3-1-2-4-7-5/h5-6H,1-4H2 CELWCAITJAEQNL-UHFFFAOYSA-N\n#\nloop_\n_pdbe_chem_comp_substructure_mapping.comp_id\n_pdbe_chem_comp_substructure_mapping.atom_id\n_pdbe_chem_comp_substructure_mapping.substructure_id\n_pdbe_chem_comp_substructure_mapping.substructure_ordinal\nSIA C2 S1 1\nSIA C3 S1 1\nSIA C4 S1 1\nSIA C5 S1 1\nSIA C6 S1 1\nSIA O6 S1 1\nSIA N5 F1 1\nSIA C10 F1 1\nSIA O10 F1 1\nSIA C11 F1 1\nSIA C5 F2 1\nSIA C6 F2 1\nSIA O6 F2 1\nSIA C2 F2 1\nSIA C3 F2 1\nSIA C4 F2 1\n#\n_pdbe_chem_comp_rdkit_properties.comp_id SIA\n_pdbe_chem_comp_rdkit_properties.exactmw 309.106\n_pdbe_chem_comp_rdkit_properties.amw 309.271\n_pdbe_chem_comp_rdkit_properties.lipinskiHBA 10\n_pdbe_chem_comp_rdkit_properties.lipinskiHBD 7\n_pdbe_chem_comp_rdkit_properties.NumRotatableBonds 11\n_pdbe_chem_comp_rdkit_properties.NumHBD 7\n_pdbe_chem_comp_rdkit_properties.NumHBA 9\n_pdbe_chem_comp_rdkit_properties.NumHeavyAtoms 21\n_pdbe_chem_comp_rdkit_properties.NumAtoms 40\n_pdbe_chem_comp_rdkit_properties.NumHeteroatoms 10\n_pdbe_chem_comp_rdkit_properties.NumAmideBonds 1\n_pdbe_chem_comp_rdkit_properties.FractionCSP3 0.818\n_pdbe_chem_comp_rdkit_properties.NumRings 1\n_pdbe_chem_comp_rdkit_properties.NumAromaticRings 0\n_pdbe_chem_comp_rdkit_properties.NumAliphaticRings 1\n_pdbe_chem_comp_rdkit_properties.NumSaturatedRings 1\n_pdbe_chem_comp_rdkit_properties.NumHeterocycles 1\n_pdbe_chem_comp_rdkit_properties.NumAromaticHeterocycles 0\n_pdbe_chem_comp_rdkit_properties.NumSaturatedHeterocycles 1\n_pdbe_chem_comp_rdkit_properties.NumAliphaticHeterocycles 1\n_pdbe_chem_comp_rdkit_properties.NumSpiroAtoms 0\n_pdbe_chem_comp_rdkit_properties.NumBridgeheadAtoms 0\n_pdbe_chem_comp_rdkit_properties.NumAtomStereoCenters 6\n_pdbe_chem_comp_rdkit_properties.NumUnspecifiedAtomStereoCenters 0\n_pdbe_chem_comp_rdkit_properties.labuteASA 146.407\n_pdbe_chem_comp_rdkit_properties.tpsa 176.780\n_pdbe_chem_comp_rdkit_properties.CrippenClogP -3.872\n_pdbe_chem_comp_rdkit_properties.CrippenMR 64.787\n_pdbe_chem_comp_rdkit_properties.chi0v 9.621\n_pdbe_chem_comp_rdkit_properties.chi1v 4.738\n_pdbe_chem_comp_rdkit_properties.chi2v 1.941\n_pdbe_chem_comp_rdkit_properties.chi3v 1.941\n_pdbe_chem_comp_rdkit_properties.chi4v 1.128\n_pdbe_chem_comp_rdkit_properties.chi0n 28.621\n_pdbe_chem_comp_rdkit_properties.chi1n 13.635\n_pdbe_chem_comp_rdkit_properties.chi2n 1.941\n_pdbe_chem_comp_rdkit_properties.chi3n 1.941\n_pdbe_chem_comp_rdkit_properties.chi4n 1.128\n_pdbe_chem_comp_rdkit_properties.hallKierAlpha -1.300\n_pdbe_chem_comp_rdkit_properties.kappa1

4.600\n_pdbe_chem_comp_rdkit_properties.kappa2
6.642\n_pdbe_chem_comp_rdkit_properties.kappa3
3.872\n_pdbe_chem_comp_rdkit_properties.Phi
1.455\n#\nloop_\n_pdbe_chem_comp_external_mappings.comp_id\n_pdbe_chem
_comp_external_mappings.source\n_pdbe_chem_comp_external_mappings.reso
urce\n_pdbe_chem_comp_external_mappings.resource_id\nSIA UniChem
ChEMBL CHEMBL1234621\nSIA UniChem DrugBank DB03721\nSIA UniChem ChEBI
49026\nSIA UniChem ZINC ZINC000004081651\nSIA UniChem fdasrs
04A90EXP8V\nSIA UniChem HMDB HMDB0000773\nSIA UniChem Nikkaji
J614.853K\nSIA UniChem MetaboLights MTBLC49026\nSIA UniChem BRENDA
141715\nSIA UniChem BRENDA 233672\nSIA UniChem BRENDA 6105\nSIA
UniChem BRENDA 84245\nSIA UniChem BRENDA 85625\nSIA UniChem 'Probes
And Drugs' PD041137\nSIA UniChem PubChem 444885\nSIA UniChem
eMolecules 474793\nSIA UniChem SureChEMBL SCHEMBL79085\nSIA UniChem
'PubChem TPHARMA' 14776495\nSIA UniChem 'PubChem TPHARMA'
15395566\n#\nloop_\n_pdbe_chem_comp_rdkit_conformer.comp_id\n_pdbe_che
m_comp_rdkit_conformer.atom_id\n_pdbe_chem_comp_rdkit_conformer.Cartn_
x_rdkit\n_pdbe_chem_comp_rdkit_conformer.Cartn_y_rdkit\n_pdbe_chem_com
p_rdkit_conformer.Cartn_z_rdkit\n_pdbe_chem_comp_rdkit_conformer.rdkit
_method\n_pdbe_chem_comp_rdkit_conformer.rdkit_ordinal\nSIA C1 -1.164
-2.811 1.058 ETKDGv3 1\nSIA C2 -1.666 -1.928 -0.070 ETKDGv3 2\nSIA C3
-2.639 -0.809 0.442 ETKDGv3 3\nSIA C4 -2.546 0.454 -0.428 ETKDGv3
4\nSIA C5 -1.079 0.960 -0.528 ETKDGv3 5\nSIA C6 -0.071 -0.181 -0.219
ETKDGv3 6\nSIA C7 1.341 0.133 -0.777 ETKDGv3 7\nSIA C8 2.378 -0.960 -
0.400 ETKDGv3 8\nSIA C9 3.769 -0.651 -0.979 ETKDGv3 9\nSIA C10 -0.223
3.295 0.052 ETKDGv3 10\nSIA C11 0.035 4.333 1.094 ETKDGv3 11\nSIA N5 -
0.868 2.066 0.414 ETKDGv3 12\nSIA O1A -0.936 -2.335 2.203 ETKDGv3
13\nSIA O1B -0.875 -4.149 0.806 ETKDGv3 14\nSIA O4 -3.104 0.224 -1.700
ETKDGv3 16\nSIA O6 -0.537 -1.404 -0.765 ETKDGv3 17\nSIA O7 1.298 0.320
-2.172 ETKDGv3 18\nSIA O8 2.484 -1.066 0.998 ETKDGv3 19\nSIA O9 4.246
0.587 -0.524 ETKDGv3 20\nSIA O10 0.157 3.497 -1.133 ETKDGv3 21\nSIA
H32 -2.392 -0.510 1.484 ETKDGv3 22\nSIA H31 -3.691 -1.171 0.465
ETKDGv3 23\nSIA H4 -3.171 1.239 0.053 ETKDGv3 24\nSIA H5 -0.925 1.324
-1.568 ETKDGv3 25\nSIA H6 0.030 -0.270 0.887 ETKDGv3 26\nSIA H7 1.678
1.089 -0.320 ETKDGv3 27\nSIA H8 2.053 -1.935 -0.838 ETKDGv3 28\nSIA
H92 3.719 -0.629 -2.088 ETKDGv3 29\nSIA H91 4.478 -1.464 -0.703
ETKDGv3 30\nSIA H111 0.843 3.984 1.770 ETKDGv3 31\nSIA H113 -0.888
4.508 1.686 ETKDGv3 32\nSIA H112 0.344 5.291 0.624 ETKDGv3 33\nSIA HN5
-1.113 1.913 1.419 ETKDGv3 34\nSIA HO1B -0.511 -4.749 1.536 ETKDGv3
35\nSIA HO4 -2.492 -0.372 -2.205 ETKDGv3 37\nSIA HO7 1.040 -0.545 -
2.585 ETKDGv3 38\nSIA HO8 1.886 -1.804 1.285 ETKDGv3 39\nSIA HO9 4.592
0.441 0.395 ETKDGv3 40\n#\ndata_STE2\n#\n_chem_comp.id
STE\n_chem_comp.name 'STEARIC ACID'\n_chem_comp.type NON-
POLYMER\n_chem_comp.pdbx_type HETAIN\n_chem_comp.formula 'C18 H36
O2'\n_chem_comp.mon_nstd_parent_comp_id ?\n_chem_comp.pdbx_synonyms ?\
n_chem_comp.pdbx_formal_charge 0\n_chem_comp.pdbx_initial_date 1999-
07-08\n_chem_comp.pdbx_modified_date 2024-09-
27\n_chem_comp.pdbx_ambiguous_flag N\n_chem_comp.pdbx_release_status
REL\n_chem_comp.pdbx_replaced_by ?\n_chem_comp.pdbx_replaces ?\n_chem_
comp.formula_weight
284.477\n_chem_comp.one_letter_code ?\n_chem_comp.three_letter_code
STE\n_chem_comp.pdbx_model_coordinates_details ?\n_chem_comp.pdbx_mode

l_coordinates_missing_flag
N\n_chem_comp.pdbx_ideal_coordinates_details ?\n_chem_comp.pdbx_ideal_coordinates_missing_flag N\n_chem_comp.pdbx_model_coordinates_db_code 1HMT\n_chem_comp.pdbx_subcomponent_list ?\n_chem_comp.pdbx_processing_site RCSB\n_chem_comp.pdbx_pcm
Y\n#\nloop_\n_chem_comp_atom.comp_id\n_chem_comp_atom.atom_id\n_chem_comp_atom.alt_atom_id\n_chem_comp_atom.type_symbol\n_chem_comp_atom.charge\n_chem_comp_atom.pdbx_align\n_chem_comp_atom.pdbx_aromatic_flag\n_chem_comp_atom.pdbx_leaving_atom_flag\n_chem_comp_atom.pdbx_stereo_config\n_chem_comp_atom.pdbx_backbone_atom_flag\n_chem_comp_atom.pdbx_n_terminal_atom_flag\n_chem_comp_atom.pdbx_c_terminal_atom_flag\n_chem_comp_atom.model_Cartn_x\n_chem_comp_atom.model_Cartn_y\n_chem_comp_atom.model_Cartn_z\n_chem_comp_atom.pdbx_model_Cartn_x_ideal\n_chem_comp_atom.pdbx_model_Cartn_y_ideal\n_chem_comp_atom.pdbx_model_Cartn_z_ideal\n_chem_comp_atom.pdbx_component_atom_id\n_chem_comp_atom.pdbx_component_comp_id\n_chem_comp_atom.pdbx_ordinal\nSTE C1 C1 C 0 1 N N N N N N 19.059 33.240 52.247 0.160 0.001 -9.422 C1 STE 1\nSTE O1 O1 O 0 1 N N N N N N 18.383 32.247 52.074 1.365 0.014 -9.335 O1 STE 2\nSTE C2 C2 C 0 1 N N N N N N 20.059 33.155 53.319 -0.684 -0.007 -8.174 C2 STE 4\nSTE C3 C3 C 0 1 N N N N N N 20.932 34.263 53.667 0.223 0.002 -6.943 C3 STE 5\nSTE C4 C4 C 0 1 N N N N N N 21.956 34.081 54.790 -0.634 -0.006 -5.676 C4 STE 6\nSTE C5 C5 C 0 1 N N N N N N 22.334 35.382 55.440 0.274 0.003 -4.445 C5 STE 7\nSTE C6 C6 C 0 1 N N N N N N 23.534 35.332 56.339 -0.584 -0.006 -3.178 C6 STE 8\nSTE C7 C7 C 0 1 N N N N N N 23.417 34.451 57.545 0.324 0.003 -1.947 C7 STE 9\nSTE C8 C8 C 0 1 N N N N N N 22.312 34.887 58.510 -0.533 -0.005 -0.680 C8 STE 10\nSTE C9 C9 C 0 1 N N N N N N 22.118 33.940 59.640 0.374 0.004 0.550 C9 STE 11\nSTE C10 C10 C 0 1 N N N N N N 21.237 34.529 60.741 -0.483 -0.005 1.817 C10 STE 12\nSTE C11 C11 C 0 1 N N N N N N 19.925 35.023 60.159 0.424 0.004 3.048 C11 STE 13\nSTE C12 C12 C 0 1 N N N N N N 19.184 34.008 59.369 -0.433 -0.004 4.315 C12 STE 14\nSTE C13 C13 C 0 1 N N N N N N 17.705 34.094 59.394 0.475 0.005 5.546 C13 STE 15\nSTE C14 C14 C 0 1 N N N N N N 17.002 34.893 58.383 -0.382 -0.004 6.813 C14 STE 16\nSTE C15 C15 C 0 1 N N N N N N 17.078 34.335 56.971 0.525 0.005 8.044 C15 STE 17\nSTE C16 C16 C 0 1 N N N N N N 16.780 35.466 55.970 -0.332 -0.003 9.311 C16 STE 18\nSTE C17 C17 C 0 1 N N N N N N 16.099 36.593 56.719 0.575 0.006 10.542 C17 STE 19\nSTE C18 C18 C 0 1 N N N N N N 16.704 37.939 56.339 -0.282 -0.003 11.809 C18 STE 20\nSTE H21 1H2 H 0 1 N N N N N N 19.528 32.840 54.248 -1.304 -0.904 -8.162 H21 STE 22\nSTE H22 2H2 H 0 1 N N N N N N 20.705 32.272 53.105 -1.323 0.875 -8.162 H22 STE 23\nSTE H31 1H3 H 0 1 N N N N N N 21.464 34.601 52.747 0.843 0.899 -6.956 H31 STE 24\nSTE H32 2H3 H 0 1 N N N N N N 20.304 35.155 53.896 0.862 -0.880 -6.956 H32 STE 25\nSTE H41 1H4 H 0 1 N N N N N N 21.596 33.344 55.545 -1.253 -0.903 -5.664 H41 STE 26\nSTE H42 2H4 H 0 1 N N N N N N 22.858 33.537 54.425 -1.273 0.876 -5.664 H42 STE 27\nSTE H51 1H5 H 0 1 N N N N N N 22.472 36.170 54.663 0.893 0.899 -4.458 H51 STE 28\nSTE H52 2H5 H 0 1 N N N N N N 21.460 35.799 55.993 0.912 -0.880 -4.458 H52 STE 29\nSTE H61 1H6 H 0 1 N N N N N N 24.436 35.047 55.749 -1.203 -0.903 -3.166 H61 STE 30\nSTE H62 2H6 H 0 1 N N N N N N 23.815 36.364 56.651 -1.222 0.876 -3.166 H62 STE 31\nSTE H71 1H7 H 0 1 N N N N N N 23.282 33.385 57.245 0.943 0.900 -1.960 H71 STE 32\nSTE H72 2H7 H 0 1 N N N N N N 24.395 34.371 58.073 0.963 -0.879 -

1.960 H72 STE 33\nSTE H81 1H8 H 0 1 N N N N N N 22.496 35.920 58.884 -1.153 -0.902 -0.668 H81 STE 34\nSTE H82 2H8 H 0 1 N N N N N N 21.353 35.058 57.966 -1.172 0.877 -0.668 H82 STE 35\nSTE H91 1H9 H 0 1 N N N N N N 21.718 32.962 59.283 0.993 0.900 0.537 H91 STE 36\nSTE H92 2H9 H 0 1 N N N N N N 23.095 33.592 60.048 1.013 -0.879 0.537 H92 STE 37\nSTE H101 1H10 H 0 0 N N N N N N 21.076 33.807 61.575 -1.102 -0.902 1.829 H101 STE 38\nSTE H102 2H10 H 0 0 N N N N N N 21.764 35.327 61.313 -1.122 0.877 1.829 H102 STE 39\nSTE H111 1H11 H 0 0 N N N N N N 19.275 35.439 60.963 1.044 0.901 3.035 H111 STE 40\nSTE H112 2H11 H 0 0 N N N N N N 20.091 35.943 59.551 1.063 -0.878 3.035 H112 STE 41\nSTE H121 1H12 H 0 0 N N N N N N 19.541 34.022 58.312 -1.052 -0.901 4.327 H121 STE 42\nSTE H122 2H12 H 0 0 N N N N N N 19.504 32.986 59.679 -1.072 0.878 4.327 H122 STE 43\nSTE H131 1H13 H 0 0 N N N N N N 17.290 33.059 59.377 1.094 0.902 5.533 H131 STE 44\nSTE H132 2H13 H 0 0 N N N N N N 17.388 34.444 60.404 1.113 -0.877 5.533 H132 STE 45\nSTE H141 1H14 H 0 0 N N N N N N 15.939 35.048 58.681 -1.002 -0.901 6.825 H141 STE 46\nSTE H142 2H14 H 0 0 N N N N N N 17.362 35.947 58.402 -1.021 0.878 6.825 H142 STE 47\nSTE H151 1H15 H 0 0 N N N N N N 18.050 33.830 56.765 1.144 0.902 8.031 H151 STE 48\nSTE H152 2H15 H 0 0 N N N N N N 16.411 33.452 56.826 1.164 -0.877 8.031 H152 STE 49\nSTE H161 1H16 H 0 0 N N N N N N 17.688 35.803 55.419 -0.952 -0.900 9.323 H161 STE 50\nSTE H162 2H16 H 0 0 N N N N N N 16.191 35.117 55.089 -0.971 0.879 9.323 H162 STE 51\nSTE H171 1H17 H 0 0 N N N N N N 14.994 36.580 56.568 1.195 0.903 10.529 H171 STE 52\nSTE H172 2H17 H 0 0 N N N N N N 16.120 36.427 57.821 1.214 -0.876 10.529 H172 STE 53\nSTE H181 1H18 H 0 0 N N N N N N 16.202 38.768 56.890 0.364 0.004 12.686 H181 STE 54\nSTE H182 2H18 H 0 0 N N N N N N 17.808 37.952 56.489 -0.921 0.880 11.821 H182 STE 55\nSTE H183 3H18 H 0 0 N N N N N N 16.682 38.104 55.236 -0.901 -0.899 11.821 H183 STE 56\n#\nloop_\n_chem_comp_bond.comp_id\n_chem_comp_bond.atom_id_1\n_chem_comp_bond.atom_id_2\n_chem_comp_bond.value_order\n_chem_comp_bond.pdbx_aromatic_flag\n_chem_comp_bond.pdbx_stereo_config\n_chem_comp_bond.pdbx_ordinal\nSTE C1 O1 DOUB N N 1\nSTE C1 C2 SING N N 3\nSTE C2 C3 SING N N 5\nSTE C2 H21 SING N N 6\nSTE C2 H22 SING N N 7\nSTE C3 C4 SING N N 8\nSTE C3 H31 SING N N 9\nSTE C3 H32 SING N N 10\nSTE C4 C5 SING N N 11\nSTE C4 H41 SING N N 12\nSTE C4 H42 SING N N 13\nSTE C5 C6 SING N N 14\nSTE C5 H51 SING N N 15\nSTE C5 H52 SING N N 16\nSTE C6 C7 SING N N 17\nSTE C6 H61 SING N N 18\nSTE C6 H62 SING N N 19\nSTE C7 C8 SING N N 20\nSTE C7 H71 SING N N 21\nSTE C7 H72 SING N N 22\nSTE C8 C9 SING N N 23\nSTE C8 H81 SING N N 24\nSTE C8 H82 SING N N 25\nSTE C9 C10 SING N N 26\nSTE C9 H91 SING N N 27\nSTE C9 H92 SING N N 28\nSTE C10 C11 SING N N 29\nSTE C10 H101 SING N N 30\nSTE C10 H102 SING N N 31\nSTE C11 C12 SING N N 32\nSTE C11 H111 SING N N 33\nSTE C11 H112 SING N N 34\nSTE C12 C13 SING N N 35\nSTE C12 H121 SING N N 36\nSTE C12 H122 SING N N 37\nSTE C13 C14 SING N N 38\nSTE C13 H131 SING N N 39\nSTE C13 H132 SING N N 40\nSTE C14 C15 SING N N 41\nSTE C14 H141 SING N N 42\nSTE C14 H142 SING N N 43\nSTE C15 C16 SING N N 44\nSTE C15 H151 SING N N 45\nSTE C15 H152 SING N N 46\nSTE C16 C17 SING N N 47\nSTE C16 H161 SING N N 48\nSTE C16 H162 SING N N 49\nSTE C17 C18 SING N N 50\nSTE C17 H171 SING N N 51\nSTE C17 H172 SING N N 52\nSTE C18 H181 SING N N 53\nSTE C18 H182 SING N N 54\nSTE C18 H183 SING N N 55\n#\nloop_\n_pdbx_chem_comp_descriptor.comp_id\n_pdbx_chem_comp_desc

riptor.type\n_pdbx_chem_comp_descriptor.program\n_pdbx_chem_comp_descr
iptor.program_version\n_pdbx_chem_comp_descriptor.descriptor\nSTE
SMILES ACDLabs 10.04 'O=C(O)CCCCCCCCCCCCCCCCC'\nSTE SMILES_CANONICAL
CACTVS 3.341 'CCCCCCCCCCCCCCCCCC(O)=O'\nSTE SMILES CACTVS 3.341
'CCCCCCCCCCCCCCCCCC(O)=O'\nSTE SMILES_CANONICAL 'OpenEye OEToolkits'
1.5.0 'CCCCCCCCCCCCCCCCCC(=O)O'\nSTE SMILES 'OpenEye OEToolkits' 1.5.0
'CCCCCCCCCCCCCCCCCC(=O)O'\nSTE InChI InChI 1.03 'InChI=1S/C18H36O2/c1-
2-3-4-5-6-7-8-9-10-11-12-13-14-15-16-17-18(19)20/h2-
17H2,1H3,(H,19,20)'\nSTE InChIKey InChI 1.03 QIQXTHQIDYTFRH-
UHFFFAOYSA-
N\n#\nloop_\n_pdbx_chem_comp_identifier.comp_id\n_pdbx_chem_comp_ident
ifier.type\n_pdbx_chem_comp_identifier.program\n_pdbx_chem_comp_identi
fier.program_version\n_pdbx_chem_comp_identifier.identifier\nSTE
'SYSTEMATIC NAME' ACDLabs 10.04 'octadecanoic acid'\nSTE 'SYSTEMATIC
NAME' 'OpenEye OEToolkits' 1.5.0 'octadecanoic
acid'\n#\nloop_\n_pdbx_chem_comp_audit.comp_id\n_pdbx_chem_comp_audit.
action_type\n_pdbx_chem_comp_audit.date\n_pdbx_chem_comp_audit.process
ing_site\nSTE 'Create component' 1999-07-08 RCSB\nSTE 'Modify
descriptor' 2011-06-04 RCSB\nSTE 'Modify PCM' 2024-09-27
PDBE\n#\nloop_\n_pdbx_chem_comp_pcm.pcm_id\n_pdbx_chem_comp_pcm.comp_i
d\n_pdbx_chem_comp_pcm.modified_residue_id\n_pdbx_chem_comp_pcm.type\n
_pdbx_chem_comp_pcm.category\n_pdbx_chem_comp_pcm.position\n_pdbx_chem
_comp_pcm.polypeptide_position\n_pdbx_chem_comp_pcm.comp_id_linking_at
om\n_pdbx_chem_comp_pcm.modified_residue_id_linking_atom\n_pdbx_chem_c
omp_pcm.uniprot_specific_ptm_accession\n_pdbx_chem_comp_pcm.uniprot_ge
neric_ptm_accession\n1 STE CYS Stearoylation Lipid/lipid-like 'Amino-
acid side chain' 'Any position' C1 SG PTM-0283 ?\n2 STE LYS
Stearoylation Lipid/lipid-like 'Amino-acid side chain' 'Any position'
C1 NZ PTM-0717 ?\n3 STE 4HH Stearoylation Lipid/lipid-like 'Amino-acid
side chain' 'Any position' C1
SU ? ?\n#\n_pdbe_chem_comp_drugbank_details.comp_id
STE\n_pdbe_chem_comp_drugbank_details.drugbank_id
DB03193\n_pdbe_chem_comp_drugbank_details.type 'small
molecule'\n_pdbe_chem_comp_drugbank_details.name 'Stearic
acid'\n_pdbe_chem_comp_drugbank_details.description\n'Stearic acid
(IUPAC systematic name: octadecanoic acid) is one of the useful types
of saturated fatty acids that comes from many animal and vegetable
fats and oils. It is a waxy
solid.'\n_pdbe_chem_comp_drugbank_details.cas_number 57-11-
4\n_pdbe_chem_comp_drugbank_details.mechanism_of_action ?\n#\nloop_\n_
pdbe_chem_comp_synonyms.comp_id\n_pdbe_chem_comp_synonyms.name\n_pdbe_
chem_comp_synonyms.provenance\n_pdbe_chem_comp_synonyms.type\nSTE
C18:0 DrugBank ?\nSTE 'n-octadecanoic acid' DrugBank ?\nSTE
'Octadecanoic acid' DrugBank ?\nSTE 'Octadecoic acid' DrugBank ?\nSTE
'Stearic acid'
DrugBank ?\n#\n_pdbe_chem_comp_drugbank_classification.comp_id
STE\n_pdbe_chem_comp_drugbank_classification.drugbank_id
DB03193\n_pdbe_chem_comp_drugbank_classification.parent 'Long-chain
fatty acids'\n_pdbe_chem_comp_drugbank_classification.kingdom 'Organic
compounds'\n_pdbe_chem_comp_drugbank_classification.class 'Fatty
Acyls'\n_pdbe_chem_comp_drugbank_classification.superclass 'Lipids and
lipid-like

molecules'\n_pdbe_chem_comp_drugbank_classification.description\n'This compound belongs to the class of organic compounds known as long-chain fatty acids. These are fatty acids with an aliphatic tail that contains between 13 and 21 carbon atoms.'\n#\nloop_\n_pdbe_chem_comp_drugbank_targets.comp_id\n_pdbe_chem_comp_drugbank_targets.drugbank_id\n_pdbe_chem_comp_drugbank_targets.name\n_pdbe_chem_comp_drugbank_targets.organism\n_pdbe_chem_comp_drugbank_targets.uniprot_id\n_pdbe_chem_comp_drugbank_targets.pharmacologically_active\n_pdbe_chem_comp_drugbank_targets.ordinal\nSTE DB03193 'Peroxisome proliferator-activated receptor alpha' Humans Q07869 unknown 1\nSTE DB03193 'Group IID secretory phospholipase A2' Humans Q9UNK4 unknown 2\n#\nloop_\n_software.name\n_software.version\n_software.description\nrdkit 2023.09.6 'Core functionality.'\npdbeccdutils 0.8.6 'Wrapper to provide 2D templates and molecular fragments.'\n#\nloop_\n_pdbe_chem_comp_atom_depiction.comp_id\n_pdbe_chem_comp_atom_depiction.atom_id\n_pdbe_chem_comp_atom_depiction.element\n_pdbe_chem_comp_atom_depiction.model_Cartn_x\n_pdbe_chem_comp_atom_depiction.model_Cartn_y\n_pdbe_chem_comp_atom_depiction.pdbx_ordinal\nSTE C1 C 5.104 0.375 1\nSTE O1 O 3.805 1.125 2\nSTE C2 C 6.404 1.125 4\nSTE C3 C 7.702 0.375 5\nSTE C4 C 9.002 1.125 6\nSTE C5 C 10.301 0.375 7\nSTE C6 C 11.600 1.125 8\nSTE C7 C 12.899 0.375 9\nSTE C8 C 14.198 1.125 10\nSTE C9 C 15.497 0.375 11\nSTE C10 C 16.796 1.125 12\nSTE C11 C 18.095 0.375 13\nSTE C12 C 19.394 1.125 14\nSTE C13 C 20.693 0.375 15\nSTE C14 C 21.992 1.125 16\nSTE C15 C 23.291 0.375 17\nSTE C16 C 24.590 1.125 18\nSTE C17 C 25.889 0.375 19\nSTE C18 C 27.188 1.125 20\n#\nloop_\n_pdbe_chem_comp_bond_depiction.comp_id\n_pdbe_chem_comp_bond_depiction.atom_id_1\n_pdbe_chem_comp_bond_depiction.atom_id_2\n_pdbe_chem_comp_bond_depiction.value_order\n_pdbe_chem_comp_bond_depiction.bond_dir\n_pdbe_chem_comp_bond_depiction.pdbx_ordinal\nSTE C1 O1 DOUBLE NONE 1\nSTE C1 C2 SINGLE NONE 3\nSTE C2 C3 SINGLE NONE 4\nSTE C3 C4 SINGLE NONE 5\nSTE C4 C5 SINGLE NONE 6\nSTE C5 C6 SINGLE NONE 7\nSTE C6 C7 SINGLE NONE 8\nSTE C7 C8 SINGLE NONE 9\nSTE C8 C9 SINGLE NONE 10\nSTE C9 C10 SINGLE NONE 11\nSTE C10 C11 SINGLE NONE 12\nSTE C11 C12 SINGLE NONE 13\nSTE C12 C13 SINGLE NONE 14\nSTE C13 C14 SINGLE NONE 15\nSTE C14 C15 SINGLE NONE 16\nSTE C15 C16 SINGLE NONE 17\nSTE C16 C17 SINGLE NONE 18\nSTE C17 C18 SINGLE NONE 19\n#\n#\n#\n_pdbe_chem_comp_rdkit_properties.comp_id STE\n_pdbe_chem_comp_rdkit_properties.exactmw 284.272\n_pdbe_chem_comp_rdkit_properties.amw 284.484\n_pdbe_chem_comp_rdkit_properties.lipinskiHBA 2\n_pdbe_chem_comp_rdkit_properties.lipinskiHBD 1\n_pdbe_chem_comp_rdkit_properties.NumRotatableBonds 17\n_pdbe_chem_comp_rdkit_properties.NumHBD 1\n_pdbe_chem_comp_rdkit_properties.NumHBA 2\n_pdbe_chem_comp_rdkit_properties.NumHeavyAtoms 20\n_pdbe_chem_comp_rdkit_properties.NumAtoms 56\n_pdbe_chem_comp_rdkit_properties.NumHeteroatoms 2\n_pdbe_chem_comp_rdkit_properties.NumAmideBonds 0\n_pdbe_chem_comp_rdkit_properties.FractionCSP3 0.944\n_pdbe_chem_comp_rdkit_properties.NumRings

0\n_pdbe_chem_comp_rdkit_properties.NumAromaticRings
0\n_pdbe_chem_comp_rdkit_properties.NumAliphaticRings
0\n_pdbe_chem_comp_rdkit_properties.NumSaturatedRings
0\n_pdbe_chem_comp_rdkit_properties.NumHeterocycles
0\n_pdbe_chem_comp_rdkit_properties.NumAromaticHeterocycles
0\n_pdbe_chem_comp_rdkit_properties.NumSaturatedHeterocycles
0\n_pdbe_chem_comp_rdkit_properties.NumAliphaticHeterocycles
0\n_pdbe_chem_comp_rdkit_properties.NumSpiroAtoms
0\n_pdbe_chem_comp_rdkit_properties.NumBridgeheadAtoms
0\n_pdbe_chem_comp_rdkit_properties.NumAtomStereoCenters
0\n_pdbe_chem_comp_rdkit_properties.NumUnspecifiedAtomStereoCenters
0\n_pdbe_chem_comp_rdkit_properties.labuteASA
176.565\n_pdbe_chem_comp_rdkit_properties.tpsa
37.300\n_pdbe_chem_comp_rdkit_properties.CrippenClogP
6.333\n_pdbe_chem_comp_rdkit_properties.CrippenMR
87.182\n_pdbe_chem_comp_rdkit_properties.chi0v
9.816\n_pdbe_chem_comp_rdkit_properties.chi1v
4.658\n_pdbe_chem_comp_rdkit_properties.chi2v
1.040\n_pdbe_chem_comp_rdkit_properties.chi3v
1.040\n_pdbe_chem_comp_rdkit_properties.chi4v
0.489\n_pdbe_chem_comp_rdkit_properties.chi0n
45.816\n_pdbe_chem_comp_rdkit_properties.chi1n
22.566\n_pdbe_chem_comp_rdkit_properties.chi2n
1.040\n_pdbe_chem_comp_rdkit_properties.chi3n
1.040\n_pdbe_chem_comp_rdkit_properties.chi4n
0.489\n_pdbe_chem_comp_rdkit_properties.hallKierAlpha -
0.530\n_pdbe_chem_comp_rdkit_properties.kappa1
2.239\n_pdbe_chem_comp_rdkit_properties.kappa2
16.524\n_pdbe_chem_comp_rdkit_properties.kappa3
17.470\n_pdbe_chem_comp_rdkit_properties.Phi
1.850\n#\nloop_\n_pdbe_chem_comp_external_mappings.comp_id\n_pdbe_chem
_comp_external_mappings.source\n_pdbe_chem_comp_external_mappings.reso
urce\n_pdbe_chem_comp_external_mappings.resource_id\nSTE UniChem
ChEMBL CHEMBL46403\nSTE UniChem DrugBank DB03193\nSTE UniChem 'Guide
to Pharmacology' 3377\nSTE UniChem ChEBI 28842\nSTE UniChem ZINC
ZINC000004978673\nSTE UniChem fdasrs 4ELV7Z65AP\nSTE UniChem HMDB
HMDB0000827\nSTE UniChem NMRShiftDB 10016909\nSTE UniChem BindingDb
50240485\nSTE UniChem DrugCentral 4611\nSTE UniChem MetaboLights
MTBLC28842\nSTE UniChem BRENDA 101697\nSTE UniChem BRENDA 10738\nSTE
UniChem BRENDA 1359\nSTE UniChem BRENDA 180906\nSTE UniChem BRENDA
20881\nSTE UniChem BRENDA 2739\nSTE UniChem BRENDA 3687\nSTE UniChem
BRENDA 6047\nSTE UniChem BRENDA 7263\nSTE UniChem BRENDA 833\nSTE
UniChem BRENDA 97314\nSTE UniChem BRENDA 98336\nSTE UniChem
ChemicalBook CB4853859\nSTE UniChem DailyMed 'STEARIC ACID'\nSTE
UniChem ClinicalTrials 'STEARIC ACID'\nSTE UniChem rxnorm 'STEARIC
ACID'\nSTE UniChem MedChemExpress HY-B2219\nSTE UniChem 'Probes And
Drugs' PD007733\nSTE UniChem CCDC STARAC\nSTE UniChem 'EPA CompTox
Dashboard' DTXSID8021642\nSTE UniChem eMolecules 26756663\nSTE UniChem
eMolecules 477279\nSTE UniChem SureChEMBL SCHEMBL659\nSTE UniChem
'PubChem TPHARMA' 14824610\nSTE UniChem PubChem 139057051\nSTE UniChem
PubChem 5281\nSTE UniChem Mcule MCULE-5127577640\nSTE UniChem ACTor
126539-56-8\nSTE UniChem ACTor 134503-33-6\nSTE UniChem ACTor 57-11-

4\nSTE UniChem ACTor 609343-71-7\nSTE UniChem ACTor 68937-76-8\nSTE UniChem Nikkaji J1.379J\n#\nloop_\n_pdbe_chem_comp_rdkit_conformer.comp_id\n_pdbe_chem_comp_rdkit_conformer.atom_id\n_pdbe_chem_comp_rdkit_conformer.Cartn_x_rdkit\n_pdbe_chem_comp_rdkit_conformer.Cartn_y_rdkit\n_pdbe_chem_comp_rdkit_conformer.Cartn_z_rdkit\n_pdbe_chem_comp_rdkit_conformer.rdkit_method\n_pdbe_chem_comp_rdkit_conformer.rdkit_ordinal\nSTE C1 5.848 2.083 -3.953 ETKDGv3 1\nSTE O1 6.394 0.973 -4.195 ETKDGv3 2\nSTE C2 4.518 2.131 -3.270 ETKDGv3 4\nSTE C3 4.702 2.228 -1.750 ETKDGv3 5\nSTE C4 3.370 2.154 -0.985 ETKDGv3 6\nSTE C5 2.547 3.448 -1.107 ETKDGv3 7\nSTE C6 1.208 3.367 -0.357 ETKDGv3 8\nSTE C7 1.382 3.386 1.172 ETKDGv3 9\nSTE C8 0.054 3.595 1.922 ETKDGv3 10\nSTE C9 -1.010 2.512 1.670 ETKDGv3 11\nSTE C10 -0.558 1.113 2.115 ETKDGv3 12\nSTE C11 -1.708 0.104 2.009 ETKDGv3 13\nSTE C12 -1.246 -1.301 2.424 ETKDGv3 14\nSTE C13 -2.399 -2.318 2.457 ETKDGv3 15\nSTE C14 -2.913 -2.676 1.054 ETKDGv3 16\nSTE C15 -3.949 -3.808 1.123 ETKDGv3 17\nSTE C16 -4.638 -4.075 -0.228 ETKDGv3 18\nSTE C17 -3.696 -4.540 -1.353 ETKDGv3 19\nSTE C18 -3.004 -5.867 -1.043 ETKDGv3 20\nSTE H21 3.940 1.216 -3.526 ETKDGv3 22\nSTE H22 3.960 3.012 -3.649 ETKDGv3 23\nSTE H31 5.337 1.378 -1.413 ETKDGv3 24\nSTE H32 5.235 3.169 -1.490 ETKDGv3 25\nSTE H41 3.609 1.975 0.084 ETKDGv3 26\nSTE H42 2.775 1.287 -1.347 ETKDGv3 27\nSTE H51 2.318 3.638 -2.177 ETKDGv3 28\nSTE H52 3.138 4.312 -0.731 ETKDGv3 29\nSTE H61 0.591 4.241 -0.660 ETKDGv3 30\nSTE H62 0.670 2.446 -0.670 ETKDGv3 31\nSTE H71 1.863 2.449 1.520 ETKDGv3 32\nSTE H72 2.060 4.223 1.449 ETKDGv3 33\nSTE H81 0.267 3.643 3.012 ETKDGv3 34\nSTE H82 -0.370 4.581 1.633 ETKDGv3 35\nSTE H91 -1.295 2.496 0.597 ETKDGv3 36\nSTE H92 -1.920 2.795 2.243 ETKDGv3 37\nSTE H101 0.278 0.768 1.469 ETKDGv3 38\nSTE H102 -0.202 1.156 3.168 ETKDGv3 39\nSTE H111 -2.543 0.428 2.668 ETKDGv3 40\nSTE H112 -2.070 0.084 0.959 ETKDGv3 41\nSTE H121 -0.450 -1.657 1.734 ETKDGv3 42\nSTE H122 -0.808 -1.248 3.444 ETKDGv3 43\nSTE H131 -2.025 -3.242 2.949 ETKDGv3 44\nSTE H132 -3.233 -1.921 3.076 ETKDGv3 45\nSTE H141 -3.383 -1.784 0.589 ETKDGv3 46\nSTE H142 -2.054 -2.998 0.428 ETKDGv3 47\nSTE H151 -3.473 -4.737 1.502 ETKDGv3 48\nSTE H152 -4.739 -3.527 1.853 ETKDGv3 49\nSTE H161 -5.425 -4.845 -0.077 ETKDGv3 50\nSTE H162 -5.153 -3.147 -0.558 ETKDGv3 51\nSTE H171 -2.935 -3.762 -1.574 ETKDGv3 52\nSTE H172 -4.301 -4.671 -2.276 ETKDGv3 53\nSTE H181 -2.469 -6.224 -1.948 ETKDGv3 54\nSTE H182 -3.750 -6.635 -0.747 ETKDGv3 55\nSTE H183 -2.258 -5.739 -0.232 ETKDGv3 56\n#\n"
}

Fig. 4g. Lewis b


```
{
    "name": "lewisb",
    "modelSeeds": [
      1
    ],
    "sequences": [
      {
        "ligand": {
          "ccdCodes": [
            "SPH", "STE-2", "BGC", "GAL", "NAG", "GAL", "FUC", "FUC"
          ],
          "id": "GSL"
        }
      }
    ],
    "dialect": "alphafold3",
    "version": 2,
    "bondedAtomPairs": [
    [["GSL",1,"N2"],["GSL",2,"C1"]],
    [["GSL",1,"O1"],["GSL",3,"C1"]],
    [["GSL",3,"O4"],["GSL",4,"C1"]],
    [["GSL",4,"O3"],["GSL",5,"C1"]],
    [["GSL",5,"O3"],["GSL",6,"C1"]],
    [["GSL",5,"O4"],["GSL",7,"C1"]],
    [["GSL",6,"O2"],["GSL",8,"C1"]]
    ],
    "userCCD": "data_STE-2\n#\n_chem_comp.id STE\n_chem_comp.name
'STEARIC ACID'\n_chem_comp.type NON-POLYMER\n_chem_comp.pdbx_type
HETAIN\n_chem_comp.formula 'C18 H36
O2'\n_chem_comp.mon_nstd_parent_comp_id ?\n_chem_comp.pdbx_synonyms ?\
n_chem_comp.pdbx_formal_charge 0\n_chem_comp.pdbx_initial_date 1999-
07-08\n_chem_comp.pdbx_modified_date 2024-09-
27\n_chem_comp.pdbx_ambiguous_flag N\n_chem_comp.pdbx_release_status
REL\n_chem_comp.pdbx_replaced_by ?\n_chem_comp.pdbx_replaces ?\n_chem_
comp.formula_weight
284.477\n_chem_comp.one_letter_code ?\n_chem_comp.three_letter_code
STE\n_chem_comp.pdbx_model_coordinates_details ?\n_chem_comp.pdbx_mode
l_coordinates_missing_flag
N\n_chem_comp.pdbx_ideal_coordinates_details ?\n_chem_comp.pdbx_ideal_
coordinates_missing_flag N\n_chem_comp.pdbx_model_coordinates_db_code
1HMT\n_chem_comp.pdbx_subcomponent_list ?\n_chem_comp.pdbx_processing_
site RCSB\n_chem_comp.pdbx_pcm
Y\n#\nloop_\n_chem_comp_atom.comp_id\n_chem_comp_atom.atom_id\n_chem_c
omp_atom.alt_atom_id\n_chem_comp_atom.type_symbol\n_chem_comp_atom.cha
rge\n_chem_comp_atom.pdbx_align\n_chem_comp_atom.pdbx_aromatic_flag\n_
chem_comp_atom.pdbx_leaving_atom_flag\n_chem_comp_atom.pdbx_stereo_con
fig\n_chem_comp_atom.pdbx_backbone_atom_flag\n_chem_comp_atom.pdbx_n_t
erminal_atom_flag\n_chem_comp_atom.pdbx_c_terminal_atom_flag\n_chem_co
mp_atom.model_Cartn_x\n_chem_comp_atom.model_Cartn_y\n_chem_comp_atom.
model_Cartn_z\n_chem_comp_atom.pdbx_model_Cartn_x_ideal\n_chem_comp_at
om.pdbx_model_Cartn_y_ideal\n_chem_comp_atom.pdbx_model_Cartn_z_ideal\
```

n_chem_comp_atom.pdbx_component_atom_id\n_chem_comp_atom.pdbx_componen
t_comp_id\n_chem_comp_atom.pdbx_ordinal\nSTE C1 C1 C 0 1 N N N N N N
19.059 33.240 52.247 0.160 0.001 -9.422 C1 STE 1\nSTE O1 O1 O 0 1 N N
N N N N 18.383 32.247 52.074 1.365 0.014 -9.335 O1 STE 2\nSTE C2 C2 C
0 1 N N N N N N 20.059 33.155 53.319 -0.684 -0.007 -8.174 C2 STE
4\nSTE C3 C3 C 0 1 N N N N N N 20.932 34.263 53.667 0.223 0.002 -6.943
C3 STE 5\nSTE C4 C4 C 0 1 N N N N N N 21.956 34.081 54.790 -0.634 -
0.006 -5.676 C4 STE 6\nSTE C5 C5 C 0 1 N N N N N N 22.334 35.382
55.440 0.274 0.003 -4.445 C5 STE 7\nSTE C6 C6 C 0 1 N N N N N N 23.534
35.332 56.339 -0.584 -0.006 -3.178 C6 STE 8\nSTE C7 C7 C 0 1 N N N N N
N 23.417 34.451 57.545 0.324 0.003 -1.947 C7 STE 9\nSTE C8 C8 C 0 1 N
N N N N 22.312 34.887 58.510 -0.533 -0.005 -0.680 C8 STE 10\nSTE C9
C9 C 0 1 N N N N N N 22.118 33.940 59.640 0.374 0.004 0.550 C9 STE
11\nSTE C10 C10 C 0 1 N N N N N N 21.237 34.529 60.741 -0.483 -0.005
1.817 C10 STE 12\nSTE C11 C11 C 0 1 N N N N N N 19.925 35.023 60.159
0.424 0.004 3.048 C11 STE 13\nSTE C12 C12 C 0 1 N N N N N N 19.184
34.008 59.369 -0.433 -0.004 4.315 C12 STE 14\nSTE C13 C13 C 0 1 N N N
N N N 17.705 34.094 59.394 0.475 0.005 5.546 C13 STE 15\nSTE C14 C14 C
0 1 N N N N N N 17.002 34.893 58.383 -0.382 -0.004 6.813 C14 STE
16\nSTE C15 C15 C 0 1 N N N N N N 17.078 34.335 56.971 0.525 0.005
8.044 C15 STE 17\nSTE C16 C16 C 0 1 N N N N N N 16.780 35.466 55.970 -
0.332 -0.003 9.311 C16 STE 18\nSTE C17 C17 C 0 1 N N N N N N 16.099
36.593 56.719 0.575 0.006 10.542 C17 STE 19\nSTE C18 C18 C 0 1 N N N N
N N 16.704 37.939 56.339 -0.282 -0.003 11.809 C18 STE 20\nSTE H21 1H2
H 0 1 N N N N N N 19.528 32.840 54.248 -1.304 -0.904 -8.162 H21 STE
22\nSTE H22 2H2 H 0 1 N N N N N N 20.705 32.272 53.105 -1.323 0.875 -
8.162 H22 STE 23\nSTE H31 1H3 H 0 1 N N N N N N 21.464 34.601 52.747
0.843 0.899 -6.956 H31 STE 24\nSTE H32 2H3 H 0 1 N N N N N N 20.304
35.155 53.896 0.862 -0.880 -6.956 H32 STE 25\nSTE H41 1H4 H 0 1 N N N
N N N 21.596 33.344 55.545 -1.253 -0.903 -5.664 H41 STE 26\nSTE H42
2H4 H 0 1 N N N N N N 22.858 33.537 54.425 -1.273 0.876 -5.664 H42 STE
27\nSTE H51 1H5 H 0 1 N N N N N N 22.472 36.170 54.663 0.893 0.899 -
4.458 H51 STE 28\nSTE H52 2H5 H 0 1 N N N N N N 21.460 35.799 55.993
0.912 -0.880 -4.458 H52 STE 29\nSTE H61 1H6 H 0 1 N N N N N N 24.436
35.047 55.749 -1.203 -0.903 -3.166 H61 STE 30\nSTE H62 2H6 H 0 1 N N N
N N N 23.815 36.364 56.651 -1.222 0.876 -3.166 H62 STE 31\nSTE H71 1H7
H 0 1 N N N N N N 23.282 33.385 57.245 0.943 0.900 -1.960 H71 STE
32\nSTE H72 2H7 H 0 1 N N N N N N 24.395 34.371 58.073 0.963 -0.879 -
1.960 H72 STE 33\nSTE H81 1H8 H 0 1 N N N N N N 22.496 35.920 58.884 -
1.153 -0.902 -0.668 H81 STE 34\nSTE H82 2H8 H 0 1 N N N N N N 21.353
35.058 57.966 -1.172 0.877 -0.668 H82 STE 35\nSTE H91 1H9 H 0 1 N N N
N N N 21.718 32.962 59.283 0.993 0.900 0.537 H91 STE 36\nSTE H92 2H9 H
0 1 N N N N N N 23.095 33.592 60.048 1.013 -0.879 0.537 H92 STE
37\nSTE H101 1H10 H 0 0 N N N N N N 21.076 33.807 61.575 -1.102 -0.902
1.829 H101 STE 38\nSTE H102 2H10 H 0 0 N N N N N N 21.764 35.327
61.313 -1.122 0.877 1.829 H102 STE 39\nSTE H111 1H11 H 0 0 N N N N N N
19.275 35.439 60.963 1.044 0.901 3.035 H111 STE 40\nSTE H112 2H11 H 0
0 N N N N N N 20.091 35.943 59.551 1.063 -0.878 3.035 H112 STE 41\nSTE
H121 1H12 H 0 0 N N N N N N 19.541 34.022 58.312 -1.052 -0.901 4.327
H121 STE 42\nSTE H122 2H12 H 0 0 N N N N N N 19.504 32.986 59.679 -
1.072 0.878 4.327 H122 STE 43\nSTE H131 1H13 H 0 0 N N N N N N 17.290
33.059 59.377 1.094 0.902 5.533 H131 STE 44\nSTE H132 2H13 H 0 0 N N N

N N N 17.388 34.444 60.404 1.113 -0.877 5.533 H132 STE 45\nSTE H141 1H14 H 0 0 N N N N N N 15.939 35.048 58.681 -1.002 -0.901 6.825 H141 STE 46\nSTE H142 2H14 H 0 0 N N N N N N 17.362 35.947 58.402 -1.021 0.878 6.825 H142 STE 47\nSTE H151 1H15 H 0 0 N N N N N N 18.050 33.830 56.765 1.144 0.902 8.031 H151 STE 48\nSTE H152 2H15 H 0 0 N N N N N N 16.411 33.452 56.826 1.164 -0.877 8.031 H152 STE 49\nSTE H161 1H16 H 0 0 N N N N N N 17.688 35.803 55.419 -0.952 -0.900 9.323 H161 STE 50\nSTE H162 2H16 H 0 0 N N N N N N 16.191 35.117 55.089 -0.971 0.879 9.323 H162 STE 51\nSTE H171 1H17 H 0 0 N N N N N N 14.994 36.580 56.568 1.195 0.903 10.529 H171 STE 52\nSTE H172 2H17 H 0 0 N N N N N N 16.120 36.427 57.821 1.214 -0.876 10.529 H172 STE 53\nSTE H181 1H18 H 0 0 N N N N N N 16.202 38.768 56.890 0.364 0.004 12.686 H181 STE 54\nSTE H182 2H18 H 0 0 N N N N N N 17.808 37.952 56.489 -0.921 0.880 11.821 H182 STE 55\nSTE H183 3H18 H 0 0 N N N N N N 16.682 38.104 55.236 -0.901 -0.899 11.821 H183 STE 56\n#\nloop_\n_chem_comp_bond.comp_id\n_chem_comp_bond.atom_id_1\n_chem_comp_bond.atom_id_2\n_chem_comp_bond.value_order\n_chem_comp_bond.pdbx_aromatic_flag\n_chem_comp_bond.pdbx_stereo_config\n_chem_comp_bond.pdbx_ordinal\nSTE C1 O1 DOUB N N 1\nSTE C1 C2 SING N N 3\nSTE C2 C3 SING N N 5\nSTE C2 H21 SING N N 6\nSTE C2 H22 SING N N 7\nSTE C3 C4 SING N N 8\nSTE C3 H31 SING N N 9\nSTE C3 H32 SING N N 10\nSTE C4 C5 SING N N 11\nSTE C4 H41 SING N N 12\nSTE C4 H42 SING N N 13\nSTE C5 C6 SING N N 14\nSTE C5 H51 SING N N 15\nSTE C5 H52 SING N N 16\nSTE C6 C7 SING N N 17\nSTE C6 H61 SING N N 18\nSTE C6 H62 SING N N 19\nSTE C7 C8 SING N N 20\nSTE C7 H71 SING N N 21\nSTE C7 H72 SING N N 22\nSTE C8 C9 SING N N 23\nSTE C8 H81 SING N N 24\nSTE C8 H82 SING N N 25\nSTE C9 C10 SING N N 26\nSTE C9 H91 SING N N 27\nSTE C9 H92 SING N N 28\nSTE C10 C11 SING N N 29\nSTE C10 H101 SING N N 30\nSTE C10 H102 SING N N 31\nSTE C11 C12 SING N N 32\nSTE C11 H111 SING N N 33\nSTE C11 H112 SING N N 34\nSTE C12 C13 SING N N 35\nSTE C12 H121 SING N N 36\nSTE C12 H122 SING N N 37\nSTE C13 C14 SING N N 38\nSTE C13 H131 SING N N 39\nSTE C13 H132 SING N N 40\nSTE C14 C15 SING N N 41\nSTE C14 H141 SING N N 42\nSTE C14 H142 SING N N 43\nSTE C15 C16 SING N N 44\nSTE C15 H151 SING N N 45\nSTE C15 H152 SING N N 46\nSTE C16 C17 SING N N 47\nSTE C16 H161 SING N N 48\nSTE C16 H162 SING N N 49\nSTE C17 C18 SING N N 50\nSTE C17 H171 SING N N 51\nSTE C17 H172 SING N N 52\nSTE C18 H181 SING N N 53\nSTE C18 H182 SING N N 54\nSTE C18 H183 SING N N 55\n#\nloop_\n_pdbx_chem_comp_descriptor.comp_id\n_pdbx_chem_comp_descriptor.type\n_pdbx_chem_comp_descriptor.program\n_pdbx_chem_comp_descriptor.program_version\n_pdbx_chem_comp_descriptor.descriptor\nSTE SMILES ACDLabs 10.04 'O=C(O)CCCCCCCCCCCCCCCCC'\nSTE SMILES_CANONICAL CACTVS 3.341 'CCCCCCCCCCCCCCCCCC(O)=O'\nSTE SMILES CACTVS 3.341 'CCCCCCCCCCCCCCCCCC(O)=O'\nSTE SMILES_CANONICAL 'OpenEye OEToolkits' 1.5.0 'CCCCCCCCCCCCCCCCCC(=O)O'\nSTE SMILES 'OpenEye OEToolkits' 1.5.0 'CCCCCCCCCCCCCCCCCC(=O)O'\nSTE InChI InChI 1.03 'InChI=1S/C18H36O2/c1-2-3-4-5-6-7-8-9-10-11-12-13-14-15-16-17-18(19)20/h2-17H2,1H3,(H,19,20)'\nSTE InChIKey InChI 1.03 QIQXTHQIDYTFRH-UHFFFAOYSA-N\n#\nloop_\n_pdbx_chem_comp_identifier.comp_id\n_pdbx_chem_comp_identifier.type\n_pdbx_chem_comp_identifier.program\n_pdbx_chem_comp_identifier.program_version\n_pdbx_chem_comp_identifier.identifier\nSTE 'SYSTEMATIC NAME' ACDLabs 10.04 'octadecanoic acid'\nSTE 'SYSTEMATIC

NAME' 'OpenEye OEToolkits' 1.5.0 'octadecanoic
acid'\n#\nloop_\n_pdbx_chem_comp_audit.comp_id\n_pdbx_chem_comp_audit.
action_type\n_pdbx_chem_comp_audit.date\n_pdbx_chem_comp_audit.process
ing_site\nSTE 'Create component' 1999-07-08 RCSB\nSTE 'Modify
descriptor' 2011-06-04 RCSB\nSTE 'Modify PCM' 2024-09-27
PDBE\n#\nloop_\n_pdbx_chem_comp_pcm.pcm_id\n_pdbx_chem_comp_pcm.comp_i
d\n_pdbx_chem_comp_pcm.modified_residue_id\n_pdbx_chem_comp_pcm.type\n
_pdbx_chem_comp_pcm.category\n_pdbx_chem_comp_pcm.position\n_pdbx_chem
_comp_pcm.polypeptide_position\n_pdbx_chem_comp_pcm.comp_id_linking_at
om\n_pdbx_chem_comp_pcm.modified_residue_id_linking_atom\n_pdbx_chem_c
omp_pcm.uniprot_specific_ptm_accession\n_pdbx_chem_comp_pcm.uniprot_ge
neric_ptm_accession\n1 STE CYS Stearoylation Lipid/lipid-like 'Amino-
acid side chain' 'Any position' C1 SG PTM-0283 ?\n2 STE LYS
Stearoylation Lipid/lipid-like 'Amino-acid side chain' 'Any position'
C1 NZ PTM-0717 ?\n3 STE 4HH Stearoylation Lipid/lipid-like 'Amino-acid
side chain' 'Any position' C1
SU ? ?\n#\n_pdbe_chem_comp_drugbank_details.comp_id
STE\n_pdbe_chem_comp_drugbank_details.drugbank_id
DB03193\n_pdbe_chem_comp_drugbank_details.type 'small
molecule'\n_pdbe_chem_comp_drugbank_details.name 'Stearic
acid'\n_pdbe_chem_comp_drugbank_details.description\n'Stearic acid
(IUPAC systematic name: octadecanoic acid) is one of the useful types
of saturated fatty acids that comes from many animal and vegetable
fats and oils. It is a waxy
solid.'\n_pdbe_chem_comp_drugbank_details.cas_number 57-11-
4\n_pdbe_chem_comp_drugbank_details.mechanism_of_action ?\n#\nloop_\n_
pdbe_chem_comp_synonyms.comp_id\n_pdbe_chem_comp_synonyms.name\n_pdbe_
chem_comp_synonyms.provenance\n_pdbe_chem_comp_synonyms.type\nSTE
C18:0 DrugBank ?\nSTE 'n-octadecanoic acid' DrugBank ?\nSTE
'Octadecanoic acid' DrugBank ?\nSTE 'Octadecoic acid' DrugBank ?\nSTE
'Stearic acid'
DrugBank ?\n#\n_pdbe_chem_comp_drugbank_classification.comp_id
STE\n_pdbe_chem_comp_drugbank_classification.drugbank_id
DB03193\n_pdbe_chem_comp_drugbank_classification.parent 'Long-chain
fatty acids'\n_pdbe_chem_comp_drugbank_classification.kingdom 'Organic
compounds'\n_pdbe_chem_comp_drugbank_classification.class 'Fatty
Acyls'\n_pdbe_chem_comp_drugbank_classification.superclass 'Lipids and
lipid-like
molecules'\n_pdbe_chem_comp_drugbank_classification.description\n'This
compound belongs to the class of organic compounds known as long-chain
fatty acids. These are fatty acids with an aliphatic tail that
contains between 13 and 21 carbon
atoms.'\n#\nloop_\n_pdbe_chem_comp_drugbank_targets.comp_id\n_pdbe_che
m_comp_drugbank_targets.drugbank_id\n_pdbe_chem_comp_drugbank_targets.
name\n_pdbe_chem_comp_drugbank_targets.organism\n_pdbe_chem_comp_drugb
ank_targets.uniprot_id\n_pdbe_chem_comp_drugbank_targets.pharmacologic
ally_active\n_pdbe_chem_comp_drugbank_targets.ordinal\nSTE DB03193
'Peroxisome proliferator-activated receptor alpha' Humans Q07869
unknown 1\nSTE DB03193 'Group IID secretory phospholipase A2' Humans
Q9UNK4 unknown
2\n#\nloop_\n_software.name\n_software.version\n_software.description\
nrdkit 2023.09.6 'Core functionality.'\npdbeccdutils 0.8.6 'Wrapper to

provide 2D templates and molecular
fragments.'\n#\nloop_\n_pdbe_chem_comp_atom_depiction.comp_id\n_pdbe_chem_comp_atom_depiction.atom_id\n_pdbe_chem_comp_atom_depiction.element\n_pdbe_chem_comp_atom_depiction.model_Cartn_x\n_pdbe_chem_comp_atom_depiction.model_Cartn_y\n_pdbe_chem_comp_atom_depiction.pdbx_ordinal\nSTE C1 C 5.104 0.375 1\nSTE O1 O 3.805 1.125 2\nSTE C2 C 6.404 1.125 4\nSTE C3 C 7.702 0.375 5\nSTE C4 C 9.002 1.125 6\nSTE C5 C 10.301 0.375 7\nSTE C6 C 11.600 1.125 8\nSTE C7 C 12.899 0.375 9\nSTE C8 C 14.198 1.125 10\nSTE C9 C 15.497 0.375 11\nSTE C10 C 16.796 1.125 12\nSTE C11 C 18.095 0.375 13\nSTE C12 C 19.394 1.125 14\nSTE C13 C 20.693 0.375 15\nSTE C14 C 21.992 1.125 16\nSTE C15 C 23.291 0.375 17\nSTE C16 C 24.590 1.125 18\nSTE C17 C 25.889 0.375 19\nSTE C18 C 27.188 1.125
20\n#\nloop_\n_pdbe_chem_comp_bond_depiction.comp_id\n_pdbe_chem_comp_bond_depiction.atom_id_1\n_pdbe_chem_comp_bond_depiction.atom_id_2\n_pdbe_chem_comp_bond_depiction.value_order\n_pdbe_chem_comp_bond_depiction.bond_dir\n_pdbe_chem_comp_bond_depiction.pdbx_ordinal\nSTE C1 O1 DOUBLE NONE 1\nSTE C1 C2 SINGLE NONE 3\nSTE C2 C3 SINGLE NONE 4\nSTE C3 C4 SINGLE NONE 5\nSTE C4 C5 SINGLE NONE 6\nSTE C5 C6 SINGLE NONE 7\nSTE C6 C7 SINGLE NONE 8\nSTE C7 C8 SINGLE NONE 9\nSTE C8 C9 SINGLE NONE 10\nSTE C9 C10 SINGLE NONE 11\nSTE C10 C11 SINGLE NONE 12\nSTE C11 C12 SINGLE NONE 13\nSTE C12 C13 SINGLE NONE 14\nSTE C13 C14 SINGLE NONE 15\nSTE C14 C15 SINGLE NONE 16\nSTE C15 C16 SINGLE NONE 17\nSTE C16 C17 SINGLE NONE 18\nSTE C17 C18 SINGLE NONE
19\n#\n#\n#\n_pdbe_chem_comp_rdkit_properties.comp_id
STE\n_pdbe_chem_comp_rdkit_properties.exactmw
284.272\n_pdbe_chem_comp_rdkit_properties.amw
284.484\n_pdbe_chem_comp_rdkit_properties.lipinskiHBA
2\n_pdbe_chem_comp_rdkit_properties.lipinskiHBD
1\n_pdbe_chem_comp_rdkit_properties.NumRotatableBonds
17\n_pdbe_chem_comp_rdkit_properties.NumHBD
1\n_pdbe_chem_comp_rdkit_properties.NumHBA
2\n_pdbe_chem_comp_rdkit_properties.NumHeavyAtoms
20\n_pdbe_chem_comp_rdkit_properties.NumAtoms
56\n_pdbe_chem_comp_rdkit_properties.NumHeteroatoms
2\n_pdbe_chem_comp_rdkit_properties.NumAmideBonds
0\n_pdbe_chem_comp_rdkit_properties.FractionCSP3
0.944\n_pdbe_chem_comp_rdkit_properties.NumRings
0\n_pdbe_chem_comp_rdkit_properties.NumAromaticRings
0\n_pdbe_chem_comp_rdkit_properties.NumAliphaticRings
0\n_pdbe_chem_comp_rdkit_properties.NumSaturatedRings
0\n_pdbe_chem_comp_rdkit_properties.NumHeterocycles
0\n_pdbe_chem_comp_rdkit_properties.NumAromaticHeterocycles
0\n_pdbe_chem_comp_rdkit_properties.NumSaturatedHeterocycles
0\n_pdbe_chem_comp_rdkit_properties.NumAliphaticHeterocycles
0\n_pdbe_chem_comp_rdkit_properties.NumSpiroAtoms
0\n_pdbe_chem_comp_rdkit_properties.NumBridgeheadAtoms
0\n_pdbe_chem_comp_rdkit_properties.NumAtomStereoCenters
0\n_pdbe_chem_comp_rdkit_properties.NumUnspecifiedAtomStereoCenters
0\n_pdbe_chem_comp_rdkit_properties.labuteASA
176.565\n_pdbe_chem_comp_rdkit_properties.tpsa
37.300\n_pdbe_chem_comp_rdkit_properties.CrippenClogP

6.333\n_pdbe_chem_comp_rdkit_properties.CrippenMR
87.182\n_pdbe_chem_comp_rdkit_properties.chi0v
9.816\n_pdbe_chem_comp_rdkit_properties.chi1v
4.658\n_pdbe_chem_comp_rdkit_properties.chi2v
1.040\n_pdbe_chem_comp_rdkit_properties.chi3v
1.040\n_pdbe_chem_comp_rdkit_properties.chi4v
0.489\n_pdbe_chem_comp_rdkit_properties.chi0n
45.816\n_pdbe_chem_comp_rdkit_properties.chi1n
22.566\n_pdbe_chem_comp_rdkit_properties.chi2n
1.040\n_pdbe_chem_comp_rdkit_properties.chi3n
1.040\n_pdbe_chem_comp_rdkit_properties.chi4n
0.489\n_pdbe_chem_comp_rdkit_properties.hallKierAlpha -
0.530\n_pdbe_chem_comp_rdkit_properties.kappa1
2.239\n_pdbe_chem_comp_rdkit_properties.kappa2
16.524\n_pdbe_chem_comp_rdkit_properties.kappa3
17.470\n_pdbe_chem_comp_rdkit_properties.Phi
1.850\n#\nloop_\n_pdbe_chem_comp_external_mappings.comp_id\n_pdbe_chem_comp_external_mappings.source\n_pdbe_chem_comp_external_mappings.resource\n_pdbe_chem_comp_external_mappings.resource_id\nSTE UniChem ChEMBL CHEMBL46403\nSTE UniChem DrugBank DB03193\nSTE UniChem 'Guide to Pharmacology' 3377\nSTE UniChem ChEBI 28842\nSTE UniChem ZINC ZINC000004978673\nSTE UniChem fdasrs 4ELV7Z65AP\nSTE UniChem HMDB HMDB0000827\nSTE UniChem NMRShiftDB 10016909\nSTE UniChem BindingDb 50240485\nSTE UniChem DrugCentral 4611\nSTE UniChem MetaboLights MTBLC28842\nSTE UniChem BRENDA 101697\nSTE UniChem BRENDA 10738\nSTE UniChem BRENDA 1359\nSTE UniChem BRENDA 180906\nSTE UniChem BRENDA 20881\nSTE UniChem BRENDA 2739\nSTE UniChem BRENDA 3687\nSTE UniChem BRENDA 6047\nSTE UniChem BRENDA 7263\nSTE UniChem BRENDA 833\nSTE UniChem BRENDA 97314\nSTE UniChem BRENDA 98336\nSTE UniChem ChemicalBook CB4853859\nSTE UniChem DailyMed 'STEARIC ACID'\nSTE UniChem ClinicalTrials 'STEARIC ACID'\nSTE UniChem rxnorm 'STEARIC ACID'\nSTE UniChem MedChemExpress HY-B2219\nSTE UniChem 'Probes And Drugs' PD007733\nSTE UniChem CCDC STARAC\nSTE UniChem 'EPA CompTox Dashboard' DTXSID8021642\nSTE UniChem eMolecules 26756663\nSTE UniChem eMolecules 477279\nSTE UniChem SureChEMBL SCHEMBL659\nSTE UniChem 'PubChem TPHARMA' 14824610\nSTE UniChem PubChem 139057051\nSTE UniChem PubChem 5281\nSTE UniChem Mcule MCULE-5127577640\nSTE UniChem ACTor 126539-56-8\nSTE UniChem ACTor 134503-33-6\nSTE UniChem ACTor 57-11-4\nSTE UniChem ACTor 609343-71-7\nSTE UniChem ACTor 68937-76-8\nSTE UniChem Nikkaji
J1.379J\n#\nloop_\n_pdbe_chem_comp_rdkit_conformer.comp_id\n_pdbe_chem_comp_rdkit_conformer.atom_id\n_pdbe_chem_comp_rdkit_conformer.Cartn_x_rdkit\n_pdbe_chem_comp_rdkit_conformer.Cartn_y_rdkit\n_pdbe_chem_comp_rdkit_conformer.Cartn_z_rdkit\n_pdbe_chem_comp_rdkit_conformer.rdkit_method\n_pdbe_chem_comp_rdkit_conformer.rdkit_ordinal\nSTE C1 5.848 2.083 -3.953 ETKDGv3 1\nSTE O1 6.394 0.973 -4.195 ETKDGv3 2\nSTE C2 4.518 2.131 -3.270 ETKDGv3 4\nSTE C3 4.702 2.228 -1.750 ETKDGv3 5\nSTE C4 3.370 2.154 -0.985 ETKDGv3 6\nSTE C5 2.547 3.448 -1.107 ETKDGv3 7\nSTE C6 1.208 3.367 -0.357 ETKDGv3 8\nSTE C7 1.382 3.386 1.172 ETKDGv3 9\nSTE C8 0.054 3.595 1.922 ETKDGv3 10\nSTE C9 -1.010 2.512 1.670 ETKDGv3 11\nSTE C10 -0.558 1.113 2.115 ETKDGv3 12\nSTE C11 -1.708 0.104 2.009 ETKDGv3 13\nSTE C12 -1.246 -1.301 2.424 ETKDGv3

14\nSTE C13 -2.399 -2.318 2.457 ETKDGv3 15\nSTE C14 -2.913 -2.676 1.054 ETKDGv3 16\nSTE C15 -3.949 -3.808 1.123 ETKDGv3 17\nSTE C16 -4.638 -4.075 -0.228 ETKDGv3 18\nSTE C17 -3.696 -4.540 -1.353 ETKDGv3 19\nSTE C18 -3.004 -5.867 -1.043 ETKDGv3 20\nSTE H21 3.940 1.216 -3.526 ETKDGv3 22\nSTE H22 3.960 3.012 -3.649 ETKDGv3 23\nSTE H31 5.337 1.378 -1.413 ETKDGv3 24\nSTE H32 5.235 3.169 -1.490 ETKDGv3 25\nSTE H41 3.609 1.975 0.084 ETKDGv3 26\nSTE H42 2.775 1.287 -1.347 ETKDGv3 27\nSTE H51 2.318 3.638 -2.177 ETKDGv3 28\nSTE H52 3.138 4.312 -0.731 ETKDGv3 29\nSTE H61 0.591 4.241 -0.660 ETKDGv3 30\nSTE H62 0.670 2.446 -0.670 ETKDGv3 31\nSTE H71 1.863 2.449 1.520 ETKDGv3 32\nSTE H72 2.060 4.223 1.449 ETKDGv3 33\nSTE H81 0.267 3.643 3.012 ETKDGv3 34\nSTE H82 -0.370 4.581 1.633 ETKDGv3 35\nSTE H91 -1.295 2.496 0.597 ETKDGv3 36\nSTE H92 -1.920 2.795 2.243 ETKDGv3 37\nSTE H101 0.278 0.768 1.469 ETKDGv3 38\nSTE H102 -0.202 1.156 3.168 ETKDGv3 39\nSTE H111 -2.543 0.428 2.668 ETKDGv3 40\nSTE H112 -2.070 0.084 0.959 ETKDGv3 41\nSTE H121 -0.450 -1.657 1.734 ETKDGv3 42\nSTE H122 -0.808 -1.248 3.444 ETKDGv3 43\nSTE H131 -2.025 -3.242 2.949 ETKDGv3 44\nSTE H132 -3.233 -1.921 3.076 ETKDGv3 45\nSTE H141 -3.383 -1.784 0.589 ETKDGv3 46\nSTE H142 -2.054 -2.998 0.428 ETKDGv3 47\nSTE H151 -3.473 -4.737 1.502 ETKDGv3 48\nSTE H152 -4.739 -3.527 1.853 ETKDGv3 49\nSTE H161 -5.425 -4.845 -0.077 ETKDGv3 50\nSTE H162 -5.153 -3.147 -0.558 ETKDGv3 51\nSTE H171 -2.935 -3.762 -1.574 ETKDGv3 52\nSTE H172 -4.301 -4.671 -2.276 ETKDGv3 53\nSTE H181 -2.469 -6.224 -1.948 ETKDGv3 54\nSTE H182 -3.750 -6.635 -0.747 ETKDGv3 55\nSTE H183 -2.258 -5.739 -0.232 ETKDGv3 56\n#\n"
}

Fig. 4h. Bc2L-C trimer in complex with three Globo H molecules on mock lipid layer


```
{
    "name": "globoh_px4_bc2lc",
    "modelSeeds": [
      1
    ],
    "sequences": [
      {
        "protein": {
          "sequence":
"MPLLSASIVSAPVVTSETYVDIPGLYLDVAKAGIRDGKLQVILNVPTPYATGNNFPGIYFAIATNQGVV
ADGCFTYSSKVPESTGRMPFTLVATIDVGSGVTFVKGQWKSVRGSAMHIDSYASLSAIWGTAAPSSQGSG
NQGAETGGTGAGNIGGGGERDGTFNLPPHIKFGVTALTHAANDQTIDIYIDDDPKPAATFKGAGAQDQNL
GTKVLDSGNGRVRVIVMANGRPSRLGSRQVDIFKKSYFGIIGSEDGADDDYNDGIVFLNWPLG",
          "id": ["LECA", "LECB", "LECC"]
        }
      },
      {
        "ligand": {
          "ccdCodes": [
            "SPH", "STE-2", "BGC", "GAL", "GLA", "NGA", "GAL", "FUC"
          ],
          "id": ["GSLA", "GSLB", "GSLC"]
        }
      },
      {
        "ligand": {
          "ccdCodes": [
            "PX4"
          ],
          "id": ["LAA", "LAB", "LAC", "LAD", "LAE", "LAF", "LAG",
"LAH", "LAI", "LAJ", "LAK", "LAL", "LAM", "LAN", "LAO", "LAP", "LAQ",
"LAR", "LAS", "LAT", "LAU", "LAV", "LAW", "LAX", "LAY", "LAZ", "LBA",
"LBB", "LBC", "LBD", "LBE", "LBF", "LBG", "LBH", "LBI", "LBJ", "LBK",
"LBL", "LBM", "LBN", "LBO", "LBP", "LBQ", "LBR", "LBS", "LBT", "LBU",
"LBV", "LBW", "LBX", "LBY", "LBZ"]
        }
      }
    ],
    "dialect": "alphafold3",
    "version": 2,
    "bondedAtomPairs": [
    [["GSLA",1,"N2"],["GSLA",2,"C1"]],
    [["GSLA",1,"O1"],["GSLA",3,"C1"]],
    [["GSLA",3,"O4"],["GSLA",4,"C1"]],
    [["GSLA",4,"O4"],["GSLA",5,"C1"]],
    [["GSLA",5,"O3"],["GSLA",6,"C1"]],
    [["GSLA",6,"O3"],["GSLA",7,"C1"]],
    [["GSLA",7,"O2"],["GSLA",8,"C1"]],

    [["GSLB",1,"N2"],["GSLB",2,"C1"]],
```

[["GSLB",1,"O1"],["GSLB",3,"C1"]],
[["GSLB",3,"O4"],["GSLB",4,"C1"]],
[["GSLB",4,"O4"],["GSLB",5,"C1"]],
[["GSLB",5,"O3"],["GSLB",6,"C1"]],
[["GSLB",6,"O3"],["GSLB",7,"C1"]],
[["GSLB",7,"O2"],["GSLB",8,"C1"]],

[["GSLC",1,"N2"],["GSLC",2,"C1"]],
[["GSLC",1,"O1"],["GSLC",3,"C1"]],
[["GSLC",3,"O4"],["GSLC",4,"C1"]],
[["GSLC",4,"O4"],["GSLC",5,"C1"]],
[["GSLC",5,"O3"],["GSLC",6,"C1"]],
[["GSLC",6,"O3"],["GSLC",7,"C1"]],
[["GSLC",7,"O2"],["GSLC",8,"C1"]]

],
"userCCD": "data_STE-2\n#\n_chem_comp.id STE\n_chem_comp.name 'STEARIC ACID'\n_chem_comp.type NON-POLYMER\n_chem_comp.pdbx_type HETAIN\n_chem_comp.formula 'C18 H36 O2'\n_chem_comp.mon_nstd_parent_comp_id ?\n_chem_comp.pdbx_synonyms ?\n_chem_comp.pdbx_formal_charge 0\n_chem_comp.pdbx_initial_date 1999-07-08\n_chem_comp.pdbx_modified_date 2024-09-27\n_chem_comp.pdbx_ambiguous_flag N\n_chem_comp.pdbx_release_status REL\n_chem_comp.pdbx_replaced_by ?\n_chem_comp.pdbx_replaces ?\n_chem_comp.formula_weight 284.477\n_chem_comp.one_letter_code ?\n_chem_comp.three_letter_code STE\n_chem_comp.pdbx_model_coordinates_details ?\n_chem_comp.pdbx_model_coordinates_missing_flag N\n_chem_comp.pdbx_ideal_coordinates_details ?\n_chem_comp.pdbx_ideal_coordinates_missing_flag N\n_chem_comp.pdbx_model_coordinates_db_code 1HMT\n_chem_comp.pdbx_subcomponent_list ?\n_chem_comp.pdbx_processing_site RCSB\n_chem_comp.pdbx_pcm Y\n#\nloop_\n_chem_comp_atom.comp_id\n_chem_comp_atom.atom_id\n_chem_comp_atom.alt_atom_id\n_chem_comp_atom.type_symbol\n_chem_comp_atom.charge\n_chem_comp_atom.pdbx_align\n_chem_comp_atom.pdbx_aromatic_flag\n_chem_comp_atom.pdbx_leaving_atom_flag\n_chem_comp_atom.pdbx_stereo_config\n_chem_comp_atom.pdbx_backbone_atom_flag\n_chem_comp_atom.pdbx_n_terminal_atom_flag\n_chem_comp_atom.pdbx_c_terminal_atom_flag\n_chem_comp_atom.model_Cartn_x\n_chem_comp_atom.model_Cartn_y\n_chem_comp_atom.model_Cartn_z\n_chem_comp_atom.pdbx_model_Cartn_x_ideal\n_chem_comp_atom.pdbx_model_Cartn_y_ideal\n_chem_comp_atom.pdbx_model_Cartn_z_ideal\n_chem_comp_atom.pdbx_component_atom_id\n_chem_comp_atom.pdbx_component_comp_id\n_chem_comp_atom.pdbx_ordinal\nSTE C1 C1 C 0 1 N N N N N N 19.059 33.240 52.247 0.160 0.001 -9.422 C1 STE 1\nSTE O1 O1 O 0 1 N N N N N N 18.383 32.247 52.074 1.365 0.014 -9.335 O1 STE 2\nSTE C2 C2 C 0 1 N N N N N N 20.059 33.155 53.319 -0.684 -0.007 -8.174 C2 STE 4\nSTE C3 C3 C 0 1 N N N N N N 20.932 34.263 53.667 0.223 0.002 -6.943 C3 STE 5\nSTE C4 C4 C 0 1 N N N N N N 21.956 34.081 54.790 -0.634 -0.006 -5.676 C4 STE 6\nSTE C5 C5 C 0 1 N N N N N N 22.334 35.382 55.440 0.274 0.003 -4.445 C5 STE 7\nSTE C6 C6 C 0 1 N N N N N N 23.534 35.332 56.339 -0.584 -0.006 -3.178 C6 STE 8\nSTE C7 C7 C 0 1 N N N N N N 23.417 34.451 57.545 0.324 0.003 -1.947 C7 STE 9\nSTE C8 C8 C 0 1 N

N N N N N 22.312 34.887 58.510 -0.533 -0.005 -0.680 C8 STE 10\nSTE C9
C9 C 0 1 N N N N N N 22.118 33.940 59.640 0.374 0.004 0.550 C9 STE
11\nSTE C10 C10 C 0 1 N N N N N N 21.237 34.529 60.741 -0.483 -0.005
1.817 C10 STE 12\nSTE C11 C11 C 0 1 N N N N N N 19.925 35.023 60.159
0.424 0.004 3.048 C11 STE 13\nSTE C12 C12 C 0 1 N N N N N N 19.184
34.008 59.369 -0.433 -0.004 4.315 C12 STE 14\nSTE C13 C13 C 0 1 N N N
N N N 17.705 34.094 59.394 0.475 0.005 5.546 C13 STE 15\nSTE C14 C14 C
0 1 N N N N N N 17.002 34.893 58.383 -0.382 -0.004 6.813 C14 STE
16\nSTE C15 C15 C 0 1 N N N N N N 17.078 34.335 56.971 0.525 0.005
8.044 C15 STE 17\nSTE C16 C16 C 0 1 N N N N N N 16.780 35.466 55.970 -
0.332 -0.003 9.311 C16 STE 18\nSTE C17 C17 C 0 1 N N N N N N 16.099
36.593 56.719 0.575 0.006 10.542 C17 STE 19\nSTE C18 C18 C 0 1 N N N N
N N 16.704 37.939 56.339 -0.282 -0.003 11.809 C18 STE 20\nSTE H21 1H2
H 0 1 N N N N N N 19.528 32.840 54.248 -1.304 -0.904 -8.162 H21 STE
22\nSTE H22 2H2 H 0 1 N N N N N N 20.705 32.272 53.105 -1.323 0.875 -
8.162 H22 STE 23\nSTE H31 1H3 H 0 1 N N N N N N 21.464 34.601 52.747
0.843 0.899 -6.956 H31 STE 24\nSTE H32 2H3 H 0 1 N N N N N N 20.304
35.155 53.896 0.862 -0.880 -6.956 H32 STE 25\nSTE H41 1H4 H 0 1 N N N
N N N 21.596 33.344 55.545 -1.253 -0.903 -5.664 H41 STE 26\nSTE H42
2H4 H 0 1 N N N N N N 22.858 33.537 54.425 -1.273 0.876 -5.664 H42 STE
27\nSTE H51 1H5 H 0 1 N N N N N N 22.472 36.170 54.663 0.893 0.899 -
4.458 H51 STE 28\nSTE H52 2H5 H 0 1 N N N N N N 21.460 35.799 55.993
0.912 -0.880 -4.458 H52 STE 29\nSTE H61 1H6 H 0 1 N N N N N N 24.436
35.047 55.749 -1.203 -0.903 -3.166 H61 STE 30\nSTE H62 2H6 H 0 1 N N N
N N N 23.815 36.364 56.651 -1.222 0.876 -3.166 H62 STE 31\nSTE H71 1H7
H 0 1 N N N N N N 23.282 33.385 57.245 0.943 0.900 -1.960 H71 STE
32\nSTE H72 2H7 H 0 1 N N N N N N 24.395 34.371 58.073 0.963 -0.879 -
1.960 H72 STE 33\nSTE H81 1H8 H 0 1 N N N N N N 22.496 35.920 58.884 -
1.153 -0.902 -0.668 H81 STE 34\nSTE H82 2H8 H 0 1 N N N N N N 21.353
35.058 57.966 -1.172 0.877 -0.668 H82 STE 35\nSTE H91 1H9 H 0 1 N N N
N N N 21.718 32.962 59.283 0.993 0.900 0.537 H91 STE 36\nSTE H92 2H9 H
0 1 N N N N N N 23.095 33.592 60.048 1.013 -0.879 0.537 H92 STE
37\nSTE H101 1H10 H 0 0 N N N N N N 21.076 33.807 61.575 -1.102 -0.902
1.829 H101 STE 38\nSTE H102 2H10 H 0 0 N N N N N N 21.764 35.327
61.313 -1.122 0.877 1.829 H102 STE 39\nSTE H111 1H11 H 0 0 N N N N N N
19.275 35.439 60.963 1.044 0.901 3.035 H111 STE 40\nSTE H112 2H11 H 0
0 N N N N N N 20.091 35.943 59.551 1.063 -0.878 3.035 H112 STE 41\nSTE
H121 1H12 H 0 0 N N N N N N 19.541 34.022 58.312 -1.052 -0.901 4.327
H121 STE 42\nSTE H122 2H12 H 0 0 N N N N N N 19.504 32.986 59.679 -
1.072 0.878 4.327 H122 STE 43\nSTE H131 1H13 H 0 0 N N N N N N 17.290
33.059 59.377 1.094 0.902 5.533 H131 STE 44\nSTE H132 2H13 H 0 0 N N N
N N N 17.388 34.444 60.404 1.113 -0.877 5.533 H132 STE 45\nSTE H141
1H14 H 0 0 N N N N N N 15.939 35.048 58.681 -1.002 -0.901 6.825 H141
STE 46\nSTE H142 2H14 H 0 0 N N N N N N 17.362 35.947 58.402 -1.021
0.878 6.825 H142 STE 47\nSTE H151 1H15 H 0 0 N N N N N N 18.050 33.830
56.765 1.144 0.902 8.031 H151 STE 48\nSTE H152 2H15 H 0 0 N N N N N N
16.411 33.452 56.826 1.164 -0.877 8.031 H152 STE 49\nSTE H161 1H16 H 0
0 N N N N N N 17.688 35.803 55.419 -0.952 -0.900 9.323 H161 STE
50\nSTE H162 2H16 H 0 0 N N N N N N 16.191 35.117 55.089 -0.971 0.879
9.323 H162 STE 51\nSTE H171 1H17 H 0 0 N N N N N N 14.994 36.580
56.568 1.195 0.903 10.529 H171 STE 52\nSTE H172 2H17 H 0 0 N N N N N N
16.120 36.427 57.821 1.214 -0.876 10.529 H172 STE 53\nSTE H181 1H18 H

0 0 N N N N N N 16.202 38.768 56.890 0.364 0.004 12.686 H181 STE 54\nSTE H182 2H18 H 0 0 N N N N N N 17.808 37.952 56.489 -0.921 0.880 11.821 H182 STE 55\nSTE H183 3H18 H 0 0 N N N N N N 16.682 38.104 55.236 -0.901 -0.899 11.821 H183 STE 56\n#\nloop_\n_chem_comp_bond.comp_id\n_chem_comp_bond.atom_id_1\n_chem_comp_bond.atom_id_2\n_chem_comp_bond.value_order\n_chem_comp_bond.pdbx_aromatic_flag\n_chem_comp_bond.pdbx_stereo_config\n_chem_comp_bond.pdbx_ordinal\nSTE C1 O1 DOUB N N 1\nSTE C1 C2 SING N N 3\nSTE C2 C3 SING N N 5\nSTE C2 H21 SING N N 6\nSTE C2 H22 SING N N 7\nSTE C3 C4 SING N N 8\nSTE C3 H31 SING N N 9\nSTE C3 H32 SING N N 10\nSTE C4 C5 SING N N 11\nSTE C4 H41 SING N N 12\nSTE C4 H42 SING N N 13\nSTE C5 C6 SING N N 14\nSTE C5 H51 SING N N 15\nSTE C5 H52 SING N N 16\nSTE C6 C7 SING N N 17\nSTE C6 H61 SING N N 18\nSTE C6 H62 SING N N 19\nSTE C7 C8 SING N N 20\nSTE C7 H71 SING N N 21\nSTE C7 H72 SING N N 22\nSTE C8 C9 SING N N 23\nSTE C8 H81 SING N N 24\nSTE C8 H82 SING N N 25\nSTE C9 C10 SING N N 26\nSTE C9 H91 SING N N 27\nSTE C9 H92 SING N N 28\nSTE C10 C11 SING N N 29\nSTE C10 H101 SING N N 30\nSTE C10 H102 SING N N 31\nSTE C11 C12 SING N N 32\nSTE C11 H111 SING N N 33\nSTE C11 H112 SING N N 34\nSTE C12 C13 SING N N 35\nSTE C12 H121 SING N N 36\nSTE C12 H122 SING N N 37\nSTE C13 C14 SING N N 38\nSTE C13 H131 SING N N 39\nSTE C13 H132 SING N N 40\nSTE C14 C15 SING N N 41\nSTE C14 H141 SING N N 42\nSTE C14 H142 SING N N 43\nSTE C15 C16 SING N N 44\nSTE C15 H151 SING N N 45\nSTE C15 H152 SING N N 46\nSTE C16 C17 SING N N 47\nSTE C16 H161 SING N N 48\nSTE C16 H162 SING N N 49\nSTE C17 C18 SING N N 50\nSTE C17 H171 SING N N 51\nSTE C17 H172 SING N N 52\nSTE C18 H181 SING N N 53\nSTE C18 H182 SING N N 54\nSTE C18 H183 SING N N 55\n#\nloop_\n_pdbx_chem_comp_descriptor.comp_id\n_pdbx_chem_comp_descriptor.type\n_pdbx_chem_comp_descriptor.program\n_pdbx_chem_comp_descriptor.program_version\n_pdbx_chem_comp_descriptor.descriptor\nSTE SMILES ACDLabs 10.04 'O=C(O)CCCCCCCCCCCCCCCCC'\nSTE SMILES_CANONICAL CACTVS 3.341 'CCCCCCCCCCCCCCCCCC(O)=O'\nSTE SMILES CACTVS 3.341 'CCCCCCCCCCCCCCCCCC(O)=O'\nSTE SMILES_CANONICAL 'OpenEye OEToolkits' 1.5.0 'CCCCCCCCCCCCCCCCCC(=O)O'\nSTE SMILES 'OpenEye OEToolkits' 1.5.0 'CCCCCCCCCCCCCCCCCC(=O)O'\nSTE InChI InChI 1.03 'InChI=1S/C18H36O2/c1-2-3-4-5-6-7-8-9-10-11-12-13-14-15-16-17-18(19)20/h2-17H2,1H3,(H,19,20)'\nSTE InChIKey InChI 1.03 QIQXTHQIDYTFRH-UHFFFAOYSA-N\n#\nloop_\n_pdbx_chem_comp_identifier.comp_id\n_pdbx_chem_comp_identifier.type\n_pdbx_chem_comp_identifier.program\n_pdbx_chem_comp_identifier.program_version\n_pdbx_chem_comp_identifier.identifier\nSTE 'SYSTEMATIC NAME' ACDLabs 10.04 'octadecanoic acid'\nSTE 'SYSTEMATIC NAME' 'OpenEye OEToolkits' 1.5.0 'octadecanoic acid'\n#\nloop_\n_pdbx_chem_comp_audit.comp_id\n_pdbx_chem_comp_audit.action_type\n_pdbx_chem_comp_audit.date\n_pdbx_chem_comp_audit.processing_site\nSTE 'Create component' 1999-07-08 RCSB\nSTE 'Modify descriptor' 2011-06-04 RCSB\nSTE 'Modify PCM' 2024-09-27 PDBE\n#\nloop_\n_pdbx_chem_comp_pcm.pcm_id\n_pdbx_chem_comp_pcm.comp_id\n_pdbx_chem_comp_pcm.modified_residue_id\n_pdbx_chem_comp_pcm.type\n_pdbx_chem_comp_pcm.category\n_pdbx_chem_comp_pcm.position\n_pdbx_chem_comp_pcm.polypeptide_position\n_pdbx_chem_comp_pcm.comp_id_linking_atom\n_pdbx_chem_comp_pcm.modified_residue_id_linking_atom\n_pdbx_chem_comp_pcm.uniprot_specific_ptm_accession\n_pdbx_chem_comp_pcm.uniprot_ge

neric_ptm_accession\n1 STE CYS Stearoylation Lipid/lipid-like 'Amino-acid side chain' 'Any position' C1 SG PTM-0283 ?\n2 STE LYS Stearoylation Lipid/lipid-like 'Amino-acid side chain' 'Any position' C1 NZ PTM-0717 ?\n3 STE 4HH Stearoylation Lipid/lipid-like 'Amino-acid side chain' 'Any position' C1
SU ? ?\n#\n_pdbe_chem_comp_drugbank_details.comp_id STE\n_pdbe_chem_comp_drugbank_details.drugbank_id DB03193\n_pdbe_chem_comp_drugbank_details.type 'small molecule'\n_pdbe_chem_comp_drugbank_details.name 'Stearic acid'\n_pdbe_chem_comp_drugbank_details.description\n'Stearic acid (IUPAC systematic name: octadecanoic acid) is one of the useful types of saturated fatty acids that comes from many animal and vegetable fats and oils. It is a waxy
solid.'\n_pdbe_chem_comp_drugbank_details.cas_number 57-11-4\n_pdbe_chem_comp_drugbank_details.mechanism_of_action ?\n#\nloop_\n_pdbe_chem_comp_synonyms.comp_id\n_pdbe_chem_comp_synonyms.name\n_pdbe_chem_comp_synonyms.provenance\n_pdbe_chem_comp_synonyms.type\nSTE C18:0 DrugBank ?\nSTE 'n-octadecanoic acid' DrugBank ?\nSTE 'Octadecanoic acid' DrugBank ?\nSTE 'Octadecoic acid' DrugBank ?\nSTE 'Stearic acid'
DrugBank ?\n#\n_pdbe_chem_comp_drugbank_classification.comp_id STE\n_pdbe_chem_comp_drugbank_classification.drugbank_id DB03193\n_pdbe_chem_comp_drugbank_classification.parent 'Long-chain fatty acids'\n_pdbe_chem_comp_drugbank_classification.kingdom 'Organic compounds'\n_pdbe_chem_comp_drugbank_classification.class 'Fatty Acyls'\n_pdbe_chem_comp_drugbank_classification.superclass 'Lipids and lipid-like
molecules'\n_pdbe_chem_comp_drugbank_classification.description\n'This compound belongs to the class of organic compounds known as long-chain fatty acids. These are fatty acids with an aliphatic tail that contains between 13 and 21 carbon
atoms.'\n#\nloop_\n_pdbe_chem_comp_drugbank_targets.comp_id\n_pdbe_chem_comp_drugbank_targets.drugbank_id\n_pdbe_chem_comp_drugbank_targets.name\n_pdbe_chem_comp_drugbank_targets.organism\n_pdbe_chem_comp_drugbank_targets.uniprot_id\n_pdbe_chem_comp_drugbank_targets.pharmacologically_active\n_pdbe_chem_comp_drugbank_targets.ordinal\nSTE DB03193 'Peroxisome proliferator-activated receptor alpha' Humans Q07869 unknown 1\nSTE DB03193 'Group IID secretory phospholipase A2' Humans Q9UNK4 unknown
2\n#\nloop_\n_software.name\n_software.version\n_software.description\nrdkit 2023.09.6 'Core functionality.'\npdbeccdutils 0.8.6 'Wrapper to provide 2D templates and molecular
fragments.'\n#\nloop_\n_pdbe_chem_comp_atom_depiction.comp_id\n_pdbe_chem_comp_atom_depiction.atom_id\n_pdbe_chem_comp_atom_depiction.element\n_pdbe_chem_comp_atom_depiction.model_Cartn_x\n_pdbe_chem_comp_atom_depiction.model_Cartn_y\n_pdbe_chem_comp_atom_depiction.pdbx_ordinal\nSTE C1 C 5.104 0.375 1\nSTE O1 O 3.805 1.125 2\nSTE C2 C 6.404 1.125 4\nSTE C3 C 7.702 0.375 5\nSTE C4 C 9.002 1.125 6\nSTE C5 C 10.301 0.375 7\nSTE C6 C 11.600 1.125 8\nSTE C7 C 12.899 0.375 9\nSTE C8 C 14.198 1.125 10\nSTE C9 C 15.497 0.375 11\nSTE C10 C 16.796 1.125 12\nSTE C11 C 18.095 0.375 13\nSTE C12 C 19.394 1.125 14\nSTE C13 C 20.693 0.375 15\nSTE C14 C 21.992 1.125 16\nSTE C15 C 23.291 0.375

17\nSTE C16 C 24.590 1.125 18\nSTE C17 C 25.889 0.375 19\nSTE C18 C
27.188 1.125
20\n#\nloop_\n_pdbe_chem_comp_bond_depiction.comp_id\n_pdbe_chem_comp_
bond_depiction.atom_id_1\n_pdbe_chem_comp_bond_depiction.atom_id_2\n_p
dbe_chem_comp_bond_depiction.value_order\n_pdbe_chem_comp_bond_depicti
on.bond_dir\n_pdbe_chem_comp_bond_depiction.pdbx_ordinal\nSTE C1 O1
DOUBLE NONE 1\nSTE C1 C2 SINGLE NONE 3\nSTE C2 C3 SINGLE NONE 4\nSTE
C3 C4 SINGLE NONE 5\nSTE C4 C5 SINGLE NONE 6\nSTE C5 C6 SINGLE NONE
7\nSTE C6 C7 SINGLE NONE 8\nSTE C7 C8 SINGLE NONE 9\nSTE C8 C9 SINGLE
NONE 10\nSTE C9 C10 SINGLE NONE 11\nSTE C10 C11 SINGLE NONE 12\nSTE
C11 C12 SINGLE NONE 13\nSTE C12 C13 SINGLE NONE 14\nSTE C13 C14 SINGLE
NONE 15\nSTE C14 C15 SINGLE NONE 16\nSTE C15 C16 SINGLE NONE 17\nSTE
C16 C17 SINGLE NONE 18\nSTE C17 C18 SINGLE NONE
19\n#\n#\n#\n_pdbe_chem_comp_rdkit_properties.comp_id
STE\n_pdbe_chem_comp_rdkit_properties.exactmw
284.272\n_pdbe_chem_comp_rdkit_properties.amw
284.484\n_pdbe_chem_comp_rdkit_properties.lipinskiHBA
2\n_pdbe_chem_comp_rdkit_properties.lipinskiHBD
1\n_pdbe_chem_comp_rdkit_properties.NumRotatableBonds
17\n_pdbe_chem_comp_rdkit_properties.NumHBD
1\n_pdbe_chem_comp_rdkit_properties.NumHBA
2\n_pdbe_chem_comp_rdkit_properties.NumHeavyAtoms
20\n_pdbe_chem_comp_rdkit_properties.NumAtoms
56\n_pdbe_chem_comp_rdkit_properties.NumHeteroatoms
2\n_pdbe_chem_comp_rdkit_properties.NumAmideBonds
0\n_pdbe_chem_comp_rdkit_properties.FractionCSP3
0.944\n_pdbe_chem_comp_rdkit_properties.NumRings
0\n_pdbe_chem_comp_rdkit_properties.NumAromaticRings
0\n_pdbe_chem_comp_rdkit_properties.NumAliphaticRings
0\n_pdbe_chem_comp_rdkit_properties.NumSaturatedRings
0\n_pdbe_chem_comp_rdkit_properties.NumHeterocycles
0\n_pdbe_chem_comp_rdkit_properties.NumAromaticHeterocycles
0\n_pdbe_chem_comp_rdkit_properties.NumSaturatedHeterocycles
0\n_pdbe_chem_comp_rdkit_properties.NumAliphaticHeterocycles
0\n_pdbe_chem_comp_rdkit_properties.NumSpiroAtoms
0\n_pdbe_chem_comp_rdkit_properties.NumBridgeheadAtoms
0\n_pdbe_chem_comp_rdkit_properties.NumAtomStereoCenters
0\n_pdbe_chem_comp_rdkit_properties.NumUnspecifiedAtomStereoCenters
0\n_pdbe_chem_comp_rdkit_properties.labuteASA
176.565\n_pdbe_chem_comp_rdkit_properties.tpsa
37.300\n_pdbe_chem_comp_rdkit_properties.CrippenClogP
6.333\n_pdbe_chem_comp_rdkit_properties.CrippenMR
87.182\n_pdbe_chem_comp_rdkit_properties.chi0v
9.816\n_pdbe_chem_comp_rdkit_properties.chi1v
4.658\n_pdbe_chem_comp_rdkit_properties.chi2v
1.040\n_pdbe_chem_comp_rdkit_properties.chi3v
1.040\n_pdbe_chem_comp_rdkit_properties.chi4v
0.489\n_pdbe_chem_comp_rdkit_properties.chi0n
45.816\n_pdbe_chem_comp_rdkit_properties.chi1n
22.566\n_pdbe_chem_comp_rdkit_properties.chi2n
1.040\n_pdbe_chem_comp_rdkit_properties.chi3n
1.040\n_pdbe_chem_comp_rdkit_properties.chi4n

0.489\n_pdbe_chem_comp_rdkit_properties.hallKierAlpha -
0.530\n_pdbe_chem_comp_rdkit_properties.kappa1
2.239\n_pdbe_chem_comp_rdkit_properties.kappa2
16.524\n_pdbe_chem_comp_rdkit_properties.kappa3
17.470\n_pdbe_chem_comp_rdkit_properties.Phi
1.850\n#\nloop_\n_pdbe_chem_comp_external_mappings.comp_id\n_pdbe_chem
_comp_external_mappings.source\n_pdbe_chem_comp_external_mappings.reso
urce\n_pdbe_chem_comp_external_mappings.resource_id\nSTE UniChem
ChEMBL CHEMBL46403\nSTE UniChem DrugBank DB03193\nSTE UniChem 'Guide
to Pharmacology' 3377\nSTE UniChem ChEBI 28842\nSTE UniChem ZINC
ZINC000004978673\nSTE UniChem fdasrs 4ELV7Z65AP\nSTE UniChem HMDB
HMDB0000827\nSTE UniChem NMRShiftDB 10016909\nSTE UniChem BindingDb
50240485\nSTE UniChem DrugCentral 4611\nSTE UniChem MetaboLights
MTBLC28842\nSTE UniChem BRENDA 101697\nSTE UniChem BRENDA 10738\nSTE
UniChem BRENDA 1359\nSTE UniChem BRENDA 180906\nSTE UniChem BRENDA
20881\nSTE UniChem BRENDA 2739\nSTE UniChem BRENDA 3687\nSTE UniChem
BRENDA 6047\nSTE UniChem BRENDA 7263\nSTE UniChem BRENDA 833\nSTE
UniChem BRENDA 97314\nSTE UniChem BRENDA 98336\nSTE UniChem
ChemicalBook CB4853859\nSTE UniChem DailyMed 'STEARIC ACID'\nSTE
UniChem ClinicalTrials 'STEARIC ACID'\nSTE UniChem rxnorm 'STEARIC
ACID'\nSTE UniChem MedChemExpress HY-B2219\nSTE UniChem 'Probes And
Drugs' PD007733\nSTE UniChem CCDC STARAC\nSTE UniChem 'EPA CompTox
Dashboard' DTXSID8021642\nSTE UniChem eMolecules 26756663\nSTE UniChem
eMolecules 477279\nSTE UniChem SureChEMBL SCHEMBL659\nSTE UniChem
'PubChem TPHARMA' 14824610\nSTE UniChem PubChem 139057051\nSTE UniChem
PubChem 5281\nSTE UniChem Mcule MCULE-5127577640\nSTE UniChem ACTor
126539-56-8\nSTE UniChem ACTor 134503-33-6\nSTE UniChem ACTor 57-11-
4\nSTE UniChem ACTor 609343-71-7\nSTE UniChem ACTor 68937-76-8\nSTE
UniChem Nikkaji
J1.379J\n#\nloop_\n_pdbe_chem_comp_rdkit_conformer.comp_id\n_pdbe_chem
_comp_rdkit_conformer.atom_id\n_pdbe_chem_comp_rdkit_conformer.Cartn_x
_rdkit\n_pdbe_chem_comp_rdkit_conformer.Cartn_y_rdkit\n_pdbe_chem_comp
_rdkit_conformer.Cartn_z_rdkit\n_pdbe_chem_comp_rdkit_conformer.rdkit_
method\n_pdbe_chem_comp_rdkit_conformer.rdkit_ordinal\nSTE C1 5.848
2.083 -3.953 ETKDGv3 1\nSTE O1 6.394 0.973 -4.195 ETKDGv3 2\nSTE C2
4.518 2.131 -3.270 ETKDGv3 4\nSTE C3 4.702 2.228 -1.750 ETKDGv3 5\nSTE
C4 3.370 2.154 -0.985 ETKDGv3 6\nSTE C5 2.547 3.448 -1.107 ETKDGv3
7\nSTE C6 1.208 3.367 -0.357 ETKDGv3 8\nSTE C7 1.382 3.386 1.172
ETKDGv3 9\nSTE C8 0.054 3.595 1.922 ETKDGv3 10\nSTE C9 -1.010 2.512
1.670 ETKDGv3 11\nSTE C10 -0.558 1.113 2.115 ETKDGv3 12\nSTE C11 -
1.708 0.104 2.009 ETKDGv3 13\nSTE C12 -1.246 -1.301 2.424 ETKDGv3
14\nSTE C13 -2.399 -2.318 2.457 ETKDGv3 15\nSTE C14 -2.913 -2.676
1.054 ETKDGv3 16\nSTE C15 -3.949 -3.808 1.123 ETKDGv3 17\nSTE C16 -
4.638 -4.075 -0.228 ETKDGv3 18\nSTE C17 -3.696 -4.540 -1.353 ETKDGv3
19\nSTE C18 -3.004 -5.867 -1.043 ETKDGv3 20\nSTE H21 3.940 1.216 -
3.526 ETKDGv3 22\nSTE H22 3.960 3.012 -3.649 ETKDGv3 23\nSTE H31 5.337
1.378 -1.413 ETKDGv3 24\nSTE H32 5.235 3.169 -1.490 ETKDGv3 25\nSTE
H41 3.609 1.975 0.084 ETKDGv3 26\nSTE H42 2.775 1.287 -1.347 ETKDGv3
27\nSTE H51 2.318 3.638 -2.177 ETKDGv3 28\nSTE H52 3.138 4.312 -0.731
ETKDGv3 29\nSTE H61 0.591 4.241 -0.660 ETKDGv3 30\nSTE H62 0.670 2.446
-0.670 ETKDGv3 31\nSTE H71 1.863 2.449 1.520 ETKDGv3 32\nSTE H72 2.060
4.223 1.449 ETKDGv3 33\nSTE H81 0.267 3.643 3.012 ETKDGv3 34\nSTE H82

-0.370 4.581 1.633 ETKDGv3 35\nSTE H91 -1.295 2.496 0.597 ETKDGv3 36\nSTE H92 -1.920 2.795 2.243 ETKDGv3 37\nSTE H101 0.278 0.768 1.469 ETKDGv3 38\nSTE H102 -0.202 1.156 3.168 ETKDGv3 39\nSTE H111 -2.543 0.428 2.668 ETKDGv3 40\nSTE H112 -2.070 0.084 0.959 ETKDGv3 41\nSTE H121 -0.450 -1.657 1.734 ETKDGv3 42\nSTE H122 -0.808 -1.248 3.444 ETKDGv3 43\nSTE H131 -2.025 -3.242 2.949 ETKDGv3 44\nSTE H132 -3.233 -1.921 3.076 ETKDGv3 45\nSTE H141 -3.383 -1.784 0.589 ETKDGv3 46\nSTE H142 -2.054 -2.998 0.428 ETKDGv3 47\nSTE H151 -3.473 -4.737 1.502 ETKDGv3 48\nSTE H152 -4.739 -3.527 1.853 ETKDGv3 49\nSTE H161 -5.425 -4.845 -0.077 ETKDGv3 50\nSTE H162 -5.153 -3.147 -0.558 ETKDGv3 51\nSTE H171 -2.935 -3.762 -1.574 ETKDGv3 52\nSTE H172 -4.301 -4.671 -2.276 ETKDGv3 53\nSTE H181 -2.469 -6.224 -1.948 ETKDGv3 54\nSTE H182 -3.750 -6.635 -0.747 ETKDGv3 55\nSTE H183 -2.258 -5.739 -0.232 ETKDGv3 56\n#\n"
}

Fig. 4i. HsALG1 in complex with GDP-Man, Mn^{2+} and chitobiose


```
{
    "name": "alg1_nnm_nag_nag",
    "modelSeeds": [
      1
    ],
    "sequences": [
      {
        "protein": {
          "sequence":
"MFLEIPRWLLALIILYLSIPLVVYYVIPYLFYGNKSTKKRIIIFVLGDVGHSPRICYHAISFSKLGWQV
ELCGYVEDTLPKIISSDPNITVHHMSNLKRKGGGTSVIFMVKKVLFQVLSIFKLLWELRGSDYILVQNPP
SIPILPIAVLYKLTGCKLIIDWHNLAYSILQLKFKGNFYHPLVLISYMVEMIFSKFADYNLTVTEAMRKY
LIQSFHLNPKRCAVLYDRPASQFQPLAGDISRQKALTTKAFIKNYIRDDFDTEKGDKIIVTSTSFTPDED
IGILLGALKIYENSYVKFDSSLPKILCFITGKGPLKEKYMKQVEEYDWKRCQIEFVWLSAEDYPKLLQLC
DYGVSLHTSSSGLDLPMKILDMFGSGLPVIAMNYPVLDELVQHNVNGLKFVDRRELHESLIFAMKDADLY
QKLKKNVTQEAENRWQSNWERTMRDLKLIH",
          "id": ["ALGONE"]
        }
      },
      {
        "ligand": {
          "ccdCodes": [
            "MN"
          ],
          "id": ["MN"]
        }
      },
      {
        "ligand": {
          "ccdCodes": [
            "GDD"
          ],
          "id": ["GDPMAN"]
        }
      },
      {
        "ligand": {
          "ccdCodes": [
            "NNM", "PO4-2", "NDG", "NAG"
          ],
          "id": ["NGA"]
        }
      }        ],
    "dialect": "alphafold3",
    "version": 2,
    "bondedAtomPairs": [
      [["NGA",1,"OP2"],["NGA",2,"P"]],
      [["NGA",2,"O4"],["NGA",3,"C1"]],
      [["NGA",3,"O4"],["NGA",4,"C1"]]
    ],
```

"userCCD": "data_PO4-2\n#\n_chem_comp.id PO4\n_chem_comp.name 'PHOSPHATE ION'\n_chem_comp.type NON-POLYMER\n_chem_comp.pdbx_type HETAI\n_chem_comp.formula 'O4 P'\n_chem_comp.mon_nstd_parent_comp_id ?\n_chem_comp.pdbx_synonyms ?\n_chem_comp.pdbx_formal_charge -3\n_chem_comp.pdbx_initial_date 1999-07-08\n_chem_comp.pdbx_modified_date 2011-06-04\n_chem_comp.pdbx_ambiguous_flag N\n_chem_comp.pdbx_release_status REL\n_chem_comp.pdbx_replaced_by ?\n_chem_comp.pdbx_replaces IPS\n_chem_comp.formula_weight 94.971\n_chem_comp.one_letter_code ?\n_chem_comp.three_letter_code PO4\n_chem_comp.pdbx_model_coordinates_details ?\n_chem_comp.pdbx_model_coordinates_missing_flag N\n_chem_comp.pdbx_ideal_coordinates_details ?\n_chem_comp.pdbx_ideal_coordinates_missing_flag N\n_chem_comp.pdbx_model_coordinates_db_code 1IXG\n_chem_comp.pdbx_subcomponent_list ?\n_chem_comp.pdbx_processing_site EBI\n#\nloop_\n_chem_comp_atom.comp_id\n_chem_comp_atom.atom_id\n_chem_comp_atom.alt_atom_id\n_chem_comp_atom.type_symbol\n_chem_comp_atom.charge\n_chem_comp_atom.pdbx_align\n_chem_comp_atom.pdbx_aromatic_flag\n_chem_comp_atom.pdbx_leaving_atom_flag\n_chem_comp_atom.pdbx_stereo_config\n_chem_comp_atom.pdbx_backbone_atom_flag\n_chem_comp_atom.pdbx_n_terminal_atom_flag\n_chem_comp_atom.pdbx_c_terminal_atom_flag\n_chem_comp_atom.model_Cartn_x\n_chem_comp_atom.model_Cartn_y\n_chem_comp_atom.model_Cartn_z\n_chem_comp_atom.pdbx_model_Cartn_x_ideal\n_chem_comp_atom.pdbx_model_Cartn_y_ideal\n_chem_comp_atom.pdbx_model_Cartn_z_ideal\n_chem_comp_atom.pdbx_component_atom_id\n_chem_comp_atom.pdbx_component_comp_id\n_chem_comp_atom.pdbx_ordinal\nPO4 P P P 0 1 N N N N N N 29.995 23.516 13.249 0.000 0.000 0.000 P PO4 1\nPO4 O1 O1 O 0 1 N N N N N N 31.092 22.988 14.164 0.000 -1.288 -0.911 O1 PO4 2\nPO4 O3 O3 O -1 1 N N N N N N 29.646 22.518 12.126 -1.288 0.000 0.911 O3 PO4 4\nPO4 O4 O4 O -1 1 N N N N N N 28.727 23.744 14.161 1.288 0.000 0.911 O4 PO4 5\n#\nloop_\n_chem_comp_bond.comp_id\n_chem_comp_bond.atom_id_1\n_chem_comp_bond.atom_id_2\n_chem_comp_bond.value_order\n_chem_comp_bond.pdbx_aromatic_flag\n_chem_comp_bond.pdbx_stereo_config\n_chem_comp_bond.pdbx_ordinal\nPO4 P O1 DOUB N N 1\nPO4 P O3 SING N N 3\nPO4 P O4 SING N N 4\n#\nloop_\n_pdbx_chem_comp_descriptor.comp_id\n_pdbx_chem_comp_descriptor.type\n_pdbx_chem_comp_descriptor.program\n_pdbx_chem_comp_descriptor.program_version\n_pdbx_chem_comp_descriptor.descriptor\nPO4 SMILES ACDLabs 10.04 '[O-]P([O-])([O-])=O'\nPO4 SMILES_CANONICAL CACTVS 3.341 '[O-][P]([O-])([O-])=O'\nPO4 SMILES CACTVS 3.341 '[O-][P]([O-])([O-])=O'\nPO4 SMILES_CANONICAL 'OpenEye OEToolkits' 1.5.0 '[O-]P(=O)([O-])[O-]'\nPO4 SMILES 'OpenEye OEToolkits' 1.5.0 '[O-]P(=O)([O-])[O-]'\nPO4 InChI InChI 1.03 InChI=1S/H3O4P/c1-5(2,3)4/h(H3,1,2,3,4)/p-3\nPO4 InChIKey InChI 1.03 NBIIXXVUZAFLBC-UHFFFAOYSA-K\n#\nloop_\n_pdbx_chem_comp_identifier.comp_id\n_pdbx_chem_comp_identifier.type\n_pdbx_chem_comp_identifier.program\n_pdbx_chem_comp_identifier.program_version\n_pdbx_chem_comp_identifier.identifier\nPO4 'SYSTEMATIC NAME' ACDLabs 10.04 phosphate\nPO4 'SYSTEMATIC NAME' 'OpenEye OEToolkits' 1.5.0 phosphate\n#\nloop_\n_pdbx_chem_comp_audit.comp_id\n_pdbx_chem_comp_au

dit.action_type\n_pdbx_chem_comp_audit.date\n_pdbx_chem_comp_audit.pro
cessing_site\nPO4 'Create component' 1999-07-08 EBI\nPO4 'Modify
descriptor' 2011-06-04
RCSB\n#\n_pdbe_chem_comp_drugbank_details.comp_id
PO4\n_pdbe_chem_comp_drugbank_details.drugbank_id
DB14523\n_pdbe_chem_comp_drugbank_details.type 'small
molecule'\n_pdbe_chem_comp_drugbank_details.name 'Phosphate
ion'\n_pdbe_chem_comp_drugbank_details.description ?\n_pdbe_chem_comp_
drugbank_details.cas_number 14265-44-
2\n_pdbe_chem_comp_drugbank_details.mechanism_of_action ?\n#\nloop_\n_
pdbe_chem_comp_synonyms.comp_id\n_pdbe_chem_comp_synonyms.name\n_pdbe_
chem_comp_synonyms.provenance\n_pdbe_chem_comp_synonyms.type\nPO4
Orthophosphate DrugBank ?\nPO4 Phosphate
DrugBank ?\n#\n_pdbe_chem_comp_drugbank_classification.comp_id
PO4\n_pdbe_chem_comp_drugbank_classification.drugbank_id
DB14523\n_pdbe_chem_comp_drugbank_classification.parent 'Non-metal
phosphates'\n_pdbe_chem_comp_drugbank_classification.kingdom
'Inorganic compounds'\n_pdbe_chem_comp_drugbank_classification.class
'Non-metal oxoanionic
compounds'\n_pdbe_chem_comp_drugbank_classification.superclass
'Homogeneous non-metal
compounds'\n_pdbe_chem_comp_drugbank_classification.description\n'This
compound belongs to the class of inorganic compounds known as non-
metal phosphates. These are inorganic non-metallic compounds
containing a phosphate as its largest
oxoanion.'\n#\nloop_\n_software.name\n_software.version\n_software.des
cription\nrdkit 2023.09.6 'Core functionality.'\npdbeccdutils 0.8.6
'Wrapper to provide 2D templates and molecular
fragments.'\n#\nloop_\n_pdbe_chem_comp_atom_depiction.comp_id\n_pdbe_c
hem_comp_atom_depiction.atom_id\n_pdbe_chem_comp_atom_depiction.elemen
t\n_pdbe_chem_comp_atom_depiction.model_Cartn_x\n_pdbe_chem_comp_atom_
depiction.model_Cartn_y\n_pdbe_chem_comp_atom_depiction.pdbx_ordinal\n
PO4 P P 4.299 0.000 1\nPO4 O1 O 5.598 0.750 2\nPO4 O3 O 3.549 1.299
4\nPO4 O4 O 5.049 -1.299
5\n#\nloop_\n_pdbe_chem_comp_bond_depiction.comp_id\n_pdbe_chem_comp_b
ond_depiction.atom_id_1\n_pdbe_chem_comp_bond_depiction.atom_id_2\n_pd
be_chem_comp_bond_depiction.value_order\n_pdbe_chem_comp_bond_depictio
n.bond_dir\n_pdbe_chem_comp_bond_depiction.pdbx_ordinal\nPO4 P O1
DOUBLE NONE 1\nPO4 P O3 SINGLE NONE 3\nPO4 P O4 SINGLE NONE
4\n#\n_pdbe_chem_comp_substructure.comp_id
PO4\n_pdbe_chem_comp_substructure.substructure_name
phosphate\n_pdbe_chem_comp_substructure.id
F1\n_pdbe_chem_comp_substructure.substructure_type
fragment\n_pdbe_chem_comp_substructure.substructure_smiles
O=P(O)(O)O\n_pdbe_chem_comp_substructure.substructure_inchis
InChI=1S/H3O4P/c1-
5(2,3)4/h(H3,1,2,3,4)\n_pdbe_chem_comp_substructure.substructure_inchi
keys NBIIXXVUZAFLBC-UHFFFAOYSA-
N\n#\nloop_\n_pdbe_chem_comp_substructure_mapping.comp_id\n_pdbe_chem_
comp_substructure_mapping.atom_id\n_pdbe_chem_comp_substructure_mappin
g.substructure_id\n_pdbe_chem_comp_substructure_mapping.substructure_o
rdinal\nPO4 P F1 1\nPO4 O1 F1 1\nPO4 O3 F1 1\nPO4 O4 F1

1\n#\n_pdbe_chem_comp_rdkit_properties.comp_id
PO4\n_pdbe_chem_comp_rdkit_properties.exactmw
94.955\n_pdbe_chem_comp_rdkit_properties.amw
94.970\n_pdbe_chem_comp_rdkit_properties.lipinskiHBA
4\n_pdbe_chem_comp_rdkit_properties.lipinskiHBD
0\n_pdbe_chem_comp_rdkit_properties.NumRotatableBonds
0\n_pdbe_chem_comp_rdkit_properties.NumHBD
0\n_pdbe_chem_comp_rdkit_properties.NumHBA
4\n_pdbe_chem_comp_rdkit_properties.NumHeavyAtoms
5\n_pdbe_chem_comp_rdkit_properties.NumAtoms
5\n_pdbe_chem_comp_rdkit_properties.NumHeteroatoms
5\n_pdbe_chem_comp_rdkit_properties.NumAmideBonds
0\n_pdbe_chem_comp_rdkit_properties.FractionCSP3
0\n_pdbe_chem_comp_rdkit_properties.NumRings
0\n_pdbe_chem_comp_rdkit_properties.NumAromaticRings
0\n_pdbe_chem_comp_rdkit_properties.NumAliphaticRings
0\n_pdbe_chem_comp_rdkit_properties.NumSaturatedRings
0\n_pdbe_chem_comp_rdkit_properties.NumHeterocycles
0\n_pdbe_chem_comp_rdkit_properties.NumAromaticHeterocycles
0\n_pdbe_chem_comp_rdkit_properties.NumSaturatedHeterocycles
0\n_pdbe_chem_comp_rdkit_properties.NumAliphaticHeterocycles
0\n_pdbe_chem_comp_rdkit_properties.NumSpiroAtoms
0\n_pdbe_chem_comp_rdkit_properties.NumBridgeheadAtoms
0\n_pdbe_chem_comp_rdkit_properties.NumAtomStereoCenters
0\n_pdbe_chem_comp_rdkit_properties.NumUnspecifiedAtomStereoCenters
0\n_pdbe_chem_comp_rdkit_properties.labuteASA
28.307\n_pdbe_chem_comp_rdkit_properties.tpsa
86.250\n_pdbe_chem_comp_rdkit_properties.CrippenClogP -
2.825\n_pdbe_chem_comp_rdkit_properties.CrippenMR
7.606\n_pdbe_chem_comp_rdkit_properties.chi0v
2.975\n_pdbe_chem_comp_rdkit_properties.chi1v
2.191\n_pdbe_chem_comp_rdkit_properties.chi2v
0\n_pdbe_chem_comp_rdkit_properties.chi3v
0\n_pdbe_chem_comp_rdkit_properties.chi4v
0\n_pdbe_chem_comp_rdkit_properties.chi0n
2.080\n_pdbe_chem_comp_rdkit_properties.chi1n
0.730\n_pdbe_chem_comp_rdkit_properties.chi2n
0\n_pdbe_chem_comp_rdkit_properties.chi3n
0\n_pdbe_chem_comp_rdkit_properties.chi4n
0\n_pdbe_chem_comp_rdkit_properties.hallKierAlpha
0.110\n_pdbe_chem_comp_rdkit_properties.kappa1
5.110\n_pdbe_chem_comp_rdkit_properties.kappa2
1.065\n_pdbe_chem_comp_rdkit_properties.kappa3
1512.242\n_pdbe_chem_comp_rdkit_properties.Phi
1.088\n#\nloop_\n_pdbe_chem_comp_external_mappings.comp_id\n_pdbe_chem
_comp_external_mappings.source\n_pdbe_chem_comp_external_mappings.reso
urce\n_pdbe_chem_comp_external_mappings.resource_id\nPO4 UniChem
DrugBank DB14523\nPO4 UniChem ChEBI 18367\nPO4 UniChem eMolecules
882178\nPO4 UniChem fdasrs NK08V8K8HR\nPO4 UniChem NMRShiftDB
30000818\nPO4 UniChem BindingDb 50155537\nPO4 UniChem MetaboLights
MTBLC18367\nPO4 UniChem BRENDA 21671\nPO4 UniChem BRENDA 867\nPO4
UniChem ChemicalBook CB3409949\nPO4 UniChem ChemicalBook

CB7191961\nPO4 UniChem rxnorm 'PHOSPHATE ION'\nPO4 UniChem 'Probes And Drugs' PD094531\nPO4 UniChem CCDC EXIZIX\nPO4 UniChem 'EPA CompTox Dashboard' DTXSID7039672\nPO4 UniChem 'PubChem TPHARMA' 15218698\nPO4 UniChem PubChem 1061\nPO4 UniChem ACTor 14265-44-2\nPO4 UniChem Nikkaji J215.970H\nPO4 UniChem Nikkaji J351.173A\n#\nloop_\n_pdbe_chem_comp_rdkit_conformer.comp_id\n_pdbe_chem_comp_rdkit_conformer.atom_id\n_pdbe_chem_comp_rdkit_conformer.Cartn_x_rdkit\n_pdbe_chem_comp_rdkit_conformer.Cartn_y_rdkit\n_pdbe_chem_comp_rdkit_conformer.Cartn_z_rdkit\n_pdbe_chem_comp_rdkit_conformer.rdkit_method\n_pdbe_chem_comp_rdkit_conformer.rdkit_ordinal\nPO4 P -0.003 0.002 0.036 ETKDGv3 1\nPO4 O1 -0.117 0.079 1.541 ETKDGv3 2\nPO4 O3 -1.075 -1.165 -0.552 ETKDGv3 4\nPO4 O4 -0.383 1.506 -0.636 ETKDGv3 5\n#\n"
}

Fig. 4j. HsGPC1 linked to GPI anchor


```
{
    "name": "gpi_0401userccd8_user_ipd_pa1",
    "modelSeeds": [
      1,2,3,4,5,6,7,8,9,10
    ],
    "sequences": [
      {
        "protein": {
          "sequence":
"DPASKSRSCGEVRQIYGAKGFSLSDVPQAEISGEHLRICPQGYTCCTSEMEENLANRSHAELETALRDS
SRVLQAMLATQLRSFDDHFQHLLNDSERTLQATFPGAFGELYTQNARAFRDLYSELRLYYRGANLHLEET
LAEFWARLLERLFKQLHPQLLLPDDYLDCLGKQAEALRPFGEAPRELRLRATRAFVAARSFVQGLGVASD
VVRKVAQVPLGPECSRAVMKLVYCAHCLGVPGARPCPDYCRNVLKGCLANQADLDAEWRNLLDSMVLITD
KFWGTSGVESVIGSVHTWLAEAINALQDNRDTLTAKVIQGCGNPKVNPQGPGPEEKRRRGKLAPRERPPS
GTLEKLVSEAKAQLRDVQDFWISLPGTLCSEKMALSTASDDRCWNGMARGRYLPEVMGDGLANQINNPEV
EVDITKPDMTIRQQIMQLKIMTNRLRSAYNGNDVDFQDASDDGSGSGSGDGCLDDLCSRKVSRKSSSSRT
PLTHALPGLSEQEGQKTS",
          "id": ["GPC"],
          "modifications":
          [
          {"ptmType": "SER-2", "ptmPosition": 507}
          ]
        }
      },
      {
        "ligand": {
          "ccdCodes": [
            "GOL-2", "PL3-2", "STE-2", "IPD-6", "PA1-0", "MAN", "MAN",
"MAN", "MAN", "NGA", "GAL", "SIA-2", "PO4-2-3", "ETA", "PO4-2-3",
"ETA"
          ],
          "id": "GPI"
        }
      }
    ],
    "dialect": "alphafold3",
    "version": 2,
    "bondedAtomPairs": [
    [["GPI",16,"N"],["GPC",507,"C"]],
    [["GPI",1,"O1"],["GPI",2,"C1"]],
    [["GPI",1,"O2"],["GPI",3,"C1"]],
    [["GPI",1,"C3"],["GPI",4,"O9"]],
    [["GPI",4,"C6"],["GPI",5,"O1"]],
    [["GPI",5,"O4"],["GPI",6,"C1"]],
    [["GPI",6,"O6"],["GPI",7,"C1"]],
    [["GPI",7,"O2"],["GPI",8,"C1"]],
    [["GPI",8,"O2"],["GPI",9,"C1"]],
    [["GPI",6,"O4"],["GPI",10,"C1"]],
    [["GPI",10,"O3"],["GPI",11,"C1"]],
    [["GPI",11,"O3"],["GPI",12,"C2"]],
    [["GPI",6,"O2"],["GPI",13,"P"]],
```

[["GPI",13,"P"],["GPI",14,"O"]],
[["GPI",8,"O6"],["GPI",15,"P"]],
[["GPI",15,"P"],["GPI",16,"O"]]
],
"userCCD": "data_SER-2\n#\n_chem_comp.id SER\n_chem_comp.name SERINE\n_chem_comp.type 'L-PEPTIDE LINKING'\n_chem_comp.pdbx_type ATOMP\n_chem_comp.formula 'C3 H7 N O3'\n_chem_comp.mon_nstd_parent_comp_id ?\n_chem_comp.pdbx_synonyms ?\n_chem_comp.pdbx_formal_charge 0\n_chem_comp.pdbx_initial_date 1999-07-08\n_chem_comp.pdbx_modified_date 2024-09-27\n_chem_comp.pdbx_ambiguous_flag N\n_chem_comp.pdbx_release_status REL\n_chem_comp.pdbx_replaced_by ?\n_chem_comp.pdbx_replaces SEG\n_chem_comp.formula_weight 105.093\n_chem_comp.one_letter_code S\n_chem_comp.three_letter_code SER\n_chem_comp.pdbx_model_coordinates_details ?\n_chem_comp.pdbx_model_coordinates_missing_flag N\n_chem_comp.pdbx_ideal_coordinates_details ?\n_chem_comp.pdbx_ideal_coordinates_missing_flag N\n_chem_comp.pdbx_model_coordinates_db_code ?\n_chem_comp.pdbx_subcomponent_list ?\n_chem_comp.pdbx_processing_site RCSB\n_chem_comp.pdbx_pcm Y\n#\nloop_\n_chem_comp_atom.comp_id\n_chem_comp_atom.atom_id\n_chem_comp_atom.alt_atom_id\n_chem_comp_atom.type_symbol\n_chem_comp_atom.charge\n_chem_comp_atom.pdbx_align\n_chem_comp_atom.pdbx_aromatic_flag\n_chem_comp_atom.pdbx_leaving_atom_flag\n_chem_comp_atom.pdbx_stereo_config\n_chem_comp_atom.pdbx_backbone_atom_flag\n_chem_comp_atom.pdbx_n_terminal_atom_flag\n_chem_comp_atom.pdbx_c_terminal_atom_flag\n_chem_comp_atom.model_Cartn_x\n_chem_comp_atom.model_Cartn_y\n_chem_comp_atom.model_Cartn_z\n_chem_comp_atom.pdbx_model_Cartn_x_ideal\n_chem_comp_atom.pdbx_model_Cartn_y_ideal\n_chem_comp_atom.pdbx_model_Cartn_z_ideal\n_chem_comp_atom.pdbx_component_atom_id\n_chem_comp_atom.pdbx_component_comp_id\n_chem_comp_atom.pdbx_ordinal\nSER N N N 0 1 N N N Y Y N 88.198 -7.658 -9.979 1.525 0.493 -0.608 N SER 1\nSER CA CA C 0 1 N N S Y N N 87.782 -7.276 -11.358 0.100 0.469 -0.252 CA SER 2\nSER C C C 0 1 N N N Y N Y 88.571 -6.062 -11.818 -0.053 0.004 1.173 C SER 3\nSER O O O 0 1 N N N Y N Y 89.008 -5.296 -10.944 0.751 -0.760 1.649 O SER 4\nSER CB CB C 0 1 N N N N N N 86.286 -6.966 -11.391 -0.642 -0.489 -1.184 CB SER 5\nSER OG OG O 0 1 N N N N N N 85.543 -8.096 -10.989 -0.496 -0.049 -2.535 OG SER 6\nSER H H H 0 1 N N N Y Y N 87.668 -8.473 -9.670 1.867 -0.449 -0.499 H SER 8\nSER H2 HN2 H 0 1 N Y N Y Y N 88.118 -6.879 -9.325 1.574 0.707 -1.593 H2 SER 9\nSER HA HA H 0 1 N N N Y N N 87.988 -8.129 -12.045 -0.316 1.471 -0.354 HA SER 10\nSER HB2 1HB H 0 1 N N N N N N 86.034 -6.065 -10.783 -0.225 -1.491 -1.081 HB2 SER 11\nSER HB3 2HB H 0 1 N N N N N N 85.961 -6.588 -12.388 -1.699 -0.507 -0.920 HB3 SER 12\nSER HG HG H 0 1 N N N N N N 84.613 -7.903 -11.009 -0.978 -0.679 -3.088 HG SER 13\n#\nloop_\n_chem_comp_bond.comp_id\n_chem_comp_bond.atom_id_1\n_chem_comp_bond.atom_id_2\n_chem_comp_bond.value_order\n_chem_comp_bond.pdbx_aromatic_flag\n_chem_comp_bond.pdbx_stereo_config\n_chem_comp_bond.pdbx_ordinal\nSER N CA SING N N 1\nSER N H SING N N 2\nSER N H2 SING N N 3\nSER CA C SING N N 4\nSER CA CB SING N N 5\nSER CA HA SING N N 6\nSER C O DOUB N N 7\nSER CB OG SING N N 9\nSER CB HB2 SING N N

10\nSER CB HB3 SING N N 11\nSER OG HG SING N N 12\n#\nloop_\n_pdbx_chem_comp_descriptor.comp_id\n_pdbx_chem_comp_descriptor.type\n_pdbx_chem_comp_descriptor.program\n_pdbx_chem_comp_descriptor.program_version\n_pdbx_chem_comp_descriptor.descriptor\nSER SMILES ACDLabs 10.04 'O=C(O)C(N)CO'\nSER SMILES_CANONICAL CACTVS 3.341 'N[C@@H](CO)C(O)=O'\nSER SMILES CACTVS 3.341 'N[CH](CO)C(O)=O'\nSER SMILES_CANONICAL 'OpenEye OEToolkits' 1.5.0 'C([C@@H](C(=O)O)N)O'\nSER SMILES 'OpenEye OEToolkits' 1.5.0 'C(C(C(=O)O)N)O'\nSER InChI InChI 1.03 'InChI=1S/C3H7NO3/c4-2(1-5)3(6)7/h2,5H,1,4H2,(H,6,7)/t2-/m0/s1'\nSER InChIKey InChI 1.03 MTCFGRXMJLQNBG-REOHCLBHSA-N\n#\nloop_\n_pdbx_chem_comp_identifier.comp_id\n_pdbx_chem_comp_identifier.type\n_pdbx_chem_comp_identifier.program\n_pdbx_chem_comp_identifier.program_version\n_pdbx_chem_comp_identifier.identifier\nSER 'SYSTEMATIC NAME' ACDLabs 10.04 L-serine\nSER 'SYSTEMATIC NAME' 'OpenEye OEToolkits' 1.5.0 '(2S)-2-amino-3-hydroxy-propanoic acid'\n#\nloop_\n_pdbx_chem_comp_audit.comp_id\n_pdbx_chem_comp_audit.action_type\n_pdbx_chem_comp_audit.date\n_pdbx_chem_comp_audit.processing_site\nSER 'Create component' 1999-07-08 RCSB\nSER 'Modify descriptor' 2011-06-04 RCSB\nSER 'Modify backbone' 2023-11-03 PDBE\nSER 'Modify PCM' 2024-09-27 PDBE\n#\n_pdbx_chem_comp_pcm.pcm_id 1\n_pdbx_chem_comp_pcm.comp_id SER\n_pdbx_chem_comp_pcm.modified_residue_id LLP\n_pdbx_chem_comp_pcm.type Serylation\n_pdbx_chem_comp_pcm.category 'Amino acid'\n_pdbx_chem_comp_pcm.position 'Amino-acid side chain'\n_pdbx_chem_comp_pcm.polypeptide_position 'Any position'\n_pdbx_chem_comp_pcm.comp_id_linking_atom N\n_pdbx_chem_comp_pcm.modified_residue_id_linking_atom 'C4''\n_pdbx_chem_comp_pcm.uniprot_specific_ptm_accession ?\n_pdbx_chem_comp_pcm.uniprot_generic_ptm_accession ?\n#\n_pdbe_chem_comp_drugbank_details.comp_id SER\n_pdbe_chem_comp_drugbank_details.drugbank_id DB00133\n_pdbe_chem_comp_drugbank_details.type 'small molecule'\n_pdbe_chem_comp_drugbank_details.name Serine\n_pdbe_chem_comp_drugbank_details.description\n'A non-essential amino acid occurring in natural form as the L-isomer. It is synthesized from glycine or threonine. It is involved in the biosynthesis of purines; pyrimidines; and other amino acids.'\n_pdbe_chem_comp_drugbank_details.cas_number 56-45-1\n_pdbe_chem_comp_drugbank_details.mechanism_of_action\n'L-Serine plays a role in cell growth and development (cellular proliferation). The conversion of L-serine to glycine by serine hydroxymethyltransferase results in the formation of the one-carbon units necessary for the synthesis of the purine bases, adenine and guanine. These bases when linked to the phosphate ester of pentose sugars are essential components of DNA and RNA and the end products of energy producing metabolic pathways, ATP and GTP. In addition, L-serine conversion to glycine via this same enzyme provides the one-carbon units necessary for production of the pyrimidine nucleotide, deoxythymidine monophosphate, also an essential component of DNA.'\n#\nloop_\n_pdbe_chem_comp_synonyms.comp_id\n_pdbe_chem_comp_synonyms.name\n_pdbe_chem_comp_synonyms.provenance\n_pdbe_chem_comp_synonyms.type\nSER '(S)-2-Amino-3-hydroxypropanoic acid' DrugBank ?\nSER (S)-Serine DrugBank ?\nSER 'alpha-Amino-beta-hydroxypropionic acid'

DrugBank ?\nSER beta-Hydroxyalanine DrugBank ?\nSER L-Serine DrugBank ?\nSER Ser DrugBank ?\nSER Serine DrugBank ?\nSER Serinum DrugBank ?\n#\n_pdbe_chem_comp_drugbank_classification.comp_id SER\n_pdbe_chem_comp_drugbank_classification.drugbank_id DB00133\n_pdbe_chem_comp_drugbank_classification.parent 'Serine and derivatives'\n_pdbe_chem_comp_drugbank_classification.kingdom 'Organic compounds'\n_pdbe_chem_comp_drugbank_classification.class 'Carboxylic acids and derivatives'\n_pdbe_chem_comp_drugbank_classification.superclass 'Organic acids and derivatives'\n_pdbe_chem_comp_drugbank_classification.description\n'This compound belongs to the class of organic compounds known as serine and derivatives. These are compounds containing serine or a derivative thereof resulting from reaction of serine at the amino group or the carboxy group, or from the replacement of any hydrogen of glycine by a heteroatom.'\n#\nloop_\n_pdbe_chem_comp_drugbank_targets.comp_id\n_pdbe_chem_comp_drugbank_targets.drugbank_id\n_pdbe_chem_comp_drugbank_targets.name\n_pdbe_chem_comp_drugbank_targets.organism\n_pdbe_chem_comp_drugbank_targets.uniprot_id\n_pdbe_chem_comp_drugbank_targets.pharmacologically_active\n_pdbe_chem_comp_drugbank_targets.ordinal\nSER DB00133 'Cystathionine beta-synthase' Humans P35520 unknown 1\nSER DB00133 'L-serine dehydratase/L-threonine deaminase' Humans P20132 unknown 2\nSER DB00133 'Serine racemase' Humans Q9GZT4 unknown 3\nSER DB00133 'Serine palmitoyltransferase 2' Humans O15270 unknown 4\nSER DB00133 'Serine palmitoyltransferase 1' Humans O15269 unknown 5\nSER DB00133 'Serine--tRNA ligase, cytoplasmic' Humans P49591 unknown 6\nSER DB00133 'Alanine--glyoxylate aminotransferase' Humans P21549 unknown 7\n#\nloop_\n_software.name\n_software.version\n_software.description\nrdkit 2023.09.6 'Core functionality.'\npdbeccdutils 0.8.6 'Wrapper to provide 2D templates and molecular fragments.'\n#\nloop_\n_pdbe_chem_comp_atom_depiction.comp_id\n_pdbe_chem_comp_atom_depiction.atom_id\n_pdbe_chem_comp_atom_depiction.element\n_pdbe_chem_comp_atom_depiction.model_Cartn_x\n_pdbe_chem_comp_atom_depiction.model_Cartn_y\n_pdbe_chem_comp_atom_depiction.pdbx_ordinal\nSER N N 3.805 1.215 1\nSER CA C 5.104 0.465 2\nSER C C 6.404 1.215 3\nSER O O 7.702 0.465 4\nSER CB C 5.104 -1.035 5\nSER OG O 3.805 -1.785 6\n#\nloop_\n_pdbe_chem_comp_bond_depiction.comp_id\n_pdbe_chem_comp_bond_depiction.atom_id_1\n_pdbe_chem_comp_bond_depiction.atom_id_2\n_pdbe_chem_comp_bond_depiction.value_order\n_pdbe_chem_comp_bond_depiction.bond_dir\n_pdbe_chem_comp_bond_depiction.pdbx_ordinal\nSER CA N SINGLE BEGINDASH 1\nSER CA C SINGLE NONE 2\nSER CA CB SINGLE NONE 3\nSER C O DOUBLE NONE 4\nSER CB OG SINGLE NONE 6\n#\n_pdbe_chem_comp_substructure.comp_id SER\n_pdbe_chem_comp_substructure.substructure_name peptide\n_pdbe_chem_comp_substructure.id F1\n_pdbe_chem_comp_substructure.substructure_type fragment\n_pdbe_chem_comp_substructure.substructure_smiles NCC=O\n_pdbe_chem_comp_substructure.substructure_inchis InChI=1S/C2H5NO/c3-1-2-4/h2H,1,3H2\n_pdbe_chem_comp_substructure.substructure_inchikeys

LYIIBVSRGJSHAV-UHFFFAOYSA-
N\n#\nloop_\n_pdbe_chem_comp_substructure_mapping.comp_id\n_pdbe_chem_
comp_substructure_mapping.atom_id\n_pdbe_chem_comp_substructure_mappin
g.substructure_id\n_pdbe_chem_comp_substructure_mapping.substructure_o
rdinal\nSER O F1 1\nSER C F1 1\nSER CA F1 1\nSER N F1
1\n#\n_pdbe_chem_comp_rdkit_properties.comp_id
SER\n_pdbe_chem_comp_rdkit_properties.exactmw
105.043\n_pdbe_chem_comp_rdkit_properties.amw
105.093\n_pdbe_chem_comp_rdkit_properties.lipinskiHBA
4\n_pdbe_chem_comp_rdkit_properties.lipinskiHBD
4\n_pdbe_chem_comp_rdkit_properties.NumRotatableBonds
4\n_pdbe_chem_comp_rdkit_properties.NumHBD
3\n_pdbe_chem_comp_rdkit_properties.NumHBA
4\n_pdbe_chem_comp_rdkit_properties.NumHeavyAtoms
7\n_pdbe_chem_comp_rdkit_properties.NumAtoms
14\n_pdbe_chem_comp_rdkit_properties.NumHeteroatoms
4\n_pdbe_chem_comp_rdkit_properties.NumAmideBonds
0\n_pdbe_chem_comp_rdkit_properties.FractionCSP3
0.667\n_pdbe_chem_comp_rdkit_properties.NumRings
0\n_pdbe_chem_comp_rdkit_properties.NumAromaticRings
0\n_pdbe_chem_comp_rdkit_properties.NumAliphaticRings
0\n_pdbe_chem_comp_rdkit_properties.NumSaturatedRings
0\n_pdbe_chem_comp_rdkit_properties.NumHeterocycles
0\n_pdbe_chem_comp_rdkit_properties.NumAromaticHeterocycles
0\n_pdbe_chem_comp_rdkit_properties.NumSaturatedHeterocycles
0\n_pdbe_chem_comp_rdkit_properties.NumAliphaticHeterocycles
0\n_pdbe_chem_comp_rdkit_properties.NumSpiroAtoms
0\n_pdbe_chem_comp_rdkit_properties.NumBridgeheadAtoms
0\n_pdbe_chem_comp_rdkit_properties.NumAtomStereoCenters
1\n_pdbe_chem_comp_rdkit_properties.NumUnspecifiedAtomStereoCenters
0\n_pdbe_chem_comp_rdkit_properties.labuteASA
50.683\n_pdbe_chem_comp_rdkit_properties.tpsa
83.550\n_pdbe_chem_comp_rdkit_properties.CrippenClogP -
1.609\n_pdbe_chem_comp_rdkit_properties.CrippenMR
22.697\n_pdbe_chem_comp_rdkit_properties.chi0v
3.172\n_pdbe_chem_comp_rdkit_properties.chi1v
1.336\n_pdbe_chem_comp_rdkit_properties.chi2v
0.290\n_pdbe_chem_comp_rdkit_properties.chi3v
0.290\n_pdbe_chem_comp_rdkit_properties.chi4v
0.042\n_pdbe_chem_comp_rdkit_properties.chi0n
10.172\n_pdbe_chem_comp_rdkit_properties.chi1n
4.547\n_pdbe_chem_comp_rdkit_properties.chi2n
0.290\n_pdbe_chem_comp_rdkit_properties.chi3n
0.290\n_pdbe_chem_comp_rdkit_properties.chi4n
0.042\n_pdbe_chem_comp_rdkit_properties.hallKierAlpha -
0.610\n_pdbe_chem_comp_rdkit_properties.kappa1
1.209\n_pdbe_chem_comp_rdkit_properties.kappa2
2.544\n_pdbe_chem_comp_rdkit_properties.kappa3
2.132\n_pdbe_chem_comp_rdkit_properties.Phi
0.439\n#\nloop_\n_pdbe_chem_comp_external_mappings.comp_id\n_pdbe_chem
_comp_external_mappings.source\n_pdbe_chem_comp_external_mappings.reso
urce\n_pdbe_chem_comp_external_mappings.resource_id\nSER UniChem

ChEMBL CHEMBL11298\nSER UniChem DrugBank DB00133\nSER UniChem 'Guide to Pharmacology' 726\nSER UniChem 'KEGG LIGAND' C00065\nSER UniChem ChEBI 17115\nSER UniChem ChEBI 33384\nSER UniChem ZINC ZINC000000895034\nSER UniChem fdasrs 452VLY9402\nSER UniChem PharmGKB PA451330\nSER UniChem HMDB HMDB0000187\nSER UniChem NMRShiftDB 60006048\nSER UniChem Recon 'ser_L'\nSER UniChem BindingDb 50357212\nSER UniChem 'EPA CompTox Dashboard' DTXSID60883230\nSER UniChem DrugCentral 4127\nSER UniChem MetaboLights MTBLC17115\nSER UniChem MetaboLights MTBLC33384\nSER UniChem BRENDA 145040\nSER UniChem BRENDA 145952\nSER UniChem BRENDA 262\nSER UniChem BRENDA 35658\nSER UniChem BRENDA 870\nSER UniChem BRENDA 930\nSER UniChem BRENDA 95\nSER UniChem Rhea 33384\nSER UniChem ChemicalBook CB5673304\nSER UniChem DailyMed SERINE\nSER UniChem ClinicalTrials L-SERINE\nSER UniChem ClinicalTrials SERINE\nSER UniChem rxnorm SERINE\nSER UniChem MedChemExpress HY-N0650\nSER UniChem 'Probes And Drugs' PD010212\nSER UniChem CCDC LSERIN\nSER UniChem Nikkaji J1.195I\nSER UniChem eMolecules 514205\nSER UniChem SureChEMBL SCHEMBL1775\nSER UniChem 'PubChem TPHARMA' 15119722\nSER UniChem 'PubChem TPHARMA' 16532479\nSER UniChem PubChem 5951\nSER UniChem PubChem 6857581\nSER UniChem Mcule MCULE-5604658333\nSER UniChem Mcule MCULE-6118038121\nSER UniChem ACTor 25821-52-7\n#\nloop_\n_pdbe_chem_comp_rdkit_conformer.comp_id\n_pdbe_chem_comp_rdkit_conformer.atom_id\n_pdbe_chem_comp_rdkit_conformer.Cartn_x_rdkit\n_pdbe_chem_comp_rdkit_conformer.Cartn_y_rdkit\n_pdbe_chem_comp_rdkit_conformer.Cartn_z_rdkit\n_pdbe_chem_comp_rdkit_conformer.rdkit_method\n_pdbe_chem_comp_rdkit_conformer.rdkit_ordinal\nSER N -0.454 1.499 0.875 ETKDGv3 1\nSER CA -0.308 0.145 0.337 ETKDGv3 2\nSER C 1.135 -0.288 0.359 ETKDGv3 3\nSER O 2.007 0.361 -0.280 ETKDGv3 4\nSER CB -0.885 0.080 -1.085 ETKDGv3 5\nSER OG -0.821 -1.228 -1.589 ETKDGv3 6\nSER H 0.139 2.162 0.324 ETKDGv3 8\nSER H2 -0.101 1.508 1.860 ETKDGv3 9\nSER HA -0.897 -0.547 0.979 ETKDGv3 10\nSER HB2 -0.310 0.750 -1.759 ETKDGv3 11\nSER HB3 -1.941 0.436 -1.081 ETKDGv3 12\nSER HG -1.541 -1.741 -1.138 ETKDGv3 13\n#\ndata_SIA-2\n#\n_chem_comp.id SIA\n_chem_comp.name 'N-acetyl-alpha-neuraminic acid'\n_chem_comp.type 'D-saccharide, alpha linking'\n_chem_comp.pdbx_type ATOMS\n_chem_comp.formula 'C11 H19 N O9'\n_chem_comp.mon_nstd_parent_comp_id ?\n_chem_comp.pdbx_synonyms 'N-acetylneuraminic acid; sialic acid; alpha-sialic acid; O-SIALIC ACID'\n_chem_comp.pdbx_formal_charge 0\n_chem_comp.pdbx_initial_date 1999-07-08\n_chem_comp.pdbx_modified_date 2024-09-27\n_chem_comp.pdbx_ambiguous_flag N\n_chem_comp.pdbx_release_status REL\n_chem_comp.pdbx_replaced_by ?\n_chem_comp.pdbx_replaces NAN\n_chem_comp.formula_weight 309.270\n_chem_comp.one_letter_code ?\n_chem_comp.three_letter_code SIA\n_chem_comp.pdbx_model_coordinates_details ?\n_chem_comp.pdbx_model_coordinates_missing_flag N\n_chem_comp.pdbx_ideal_coordinates_details ?\n_chem_comp.pdbx_ideal_coordinates_missing_flag N\n_chem_comp.pdbx_model_coordinates_db_code ?\n_chem_comp.pdbx_subcomponent_list ?\n_chem_comp.pdbx_processing_site EBI\n_chem_comp.pdbx_pcm Y\n#\nloop_\n_pdbx_chem_comp_synonyms.ordinal\n_pdbx_chem_comp_synonym

s.comp_id\n_pdbx_chem_comp_synonyms.name\n_pdbx_chem_comp_synonyms.pro venance\n_pdbx_chem_comp_synonyms.type\n1 SIA 'N-acetylneuraminic acid' PDB ?\n2 SIA 'sialic acid' PDB ?\n3 SIA 'alpha-sialic acid' PDB ?\n4 SIA 'O-SIALIC ACID'
PDB ?\n#\nloop_\n_chem_comp_atom.comp_id\n_chem_comp_atom.atom_id\n_ch em_comp_atom.alt_atom_id\n_chem_comp_atom.type_symbol\n_chem_comp_atom .charge\n_chem_comp_atom.pdbx_align\n_chem_comp_atom.pdbx_aromatic_fla g\n_chem_comp_atom.pdbx_leaving_atom_flag\n_chem_comp_atom.pdbx_stereo _config\n_chem_comp_atom.pdbx_backbone_atom_flag\n_chem_comp_atom.pdbx _n_terminal_atom_flag\n_chem_comp_atom.pdbx_c_terminal_atom_flag\n_che m_comp_atom.model_Cartn_x\n_chem_comp_atom.model_Cartn_y\n_chem_comp_a tom.model_Cartn_z\n_chem_comp_atom.pdbx_model_Cartn_x_ideal\n_chem_com p_atom.pdbx_model_Cartn_y_ideal\n_chem_comp_atom.pdbx_model_Cartn_z_id eal\n_chem_comp_atom.pdbx_component_atom_id\n_chem_comp_atom.pdbx_comp onent_comp_id\n_chem_comp_atom.pdbx_ordinal\nSIA C1 C1 C 0 1 N N N N N N -2.196 58.872 -5.981 -2.502 -0.832 0.174 C1 SIA 1\nSIA C2 C2 C 0 1 N N R N N N -1.870 58.021 -7.211 -2.171 0.628 0.342 C2 SIA 2\nSIA C3 C3 C 0 1 N N N N N N -0.844 56.899 -7.306 -1.789 0.898 1.800 C3 SIA 3\nSIA C4 C4 C 0 1 N N S N N N -1.157 55.904 -8.413 -0.586 0.023 2.171 C4 SIA 4\nSIA C5 C5 C 0 1 N N R N N N -2.015 56.516 -9.517 0.529 0.264 1.148 C5 SIA 5\nSIA C6 C6 C 0 1 N N R N N N -3.352 56.956 -8.912 -0.026 0.043 -0.259 C6 SIA 6\nSIA C7 C7 C 0 1 N N R N N N -4.224 57.698 -9.942 1.088 0.251 -1.286 C7 SIA 7\nSIA C8 C8 C 0 1 N N R N N N -5.571 58.131 -9.360 0.535 0.021 -2.694 C8 SIA 8\nSIA C9 C9 C 0 1 N N N N N N -6.601 58.674 -10.381 1.650 0.229 -3.721 C9 SIA 9\nSIA C10 C10 C 0 1 N N N N N N -1.897 55.374 -11.759 2.632 -0.329 2.226 C10 SIA 10\nSIA C11 C11 C 0 1 N N N N N N -2.200 54.057 -12.454 3.763 -1.292 2.478 C11 SIA 11\nSIA N5 N5 N 0 1 N N N N N N -2.202 55.444 -10.478 1.629 -0.671 1.394 N5 SIA 12\nSIA O1A O1A O 0 1 N N N N N N -1.289 58.815 -5.130 - 2.191 -1.408 -0.841 O1A SIA 13\nSIA O1B O1B O 0 1 N N N N N N -3.210 59.504 -5.631 -3.141 -1.493 1.152 O1B SIA 14\nSIA O4 O4 O 0 1 N N N N N N 0.072 55.523 -8.986 -0.123 0.370 3.478 O4 SIA 16\nSIA O6 O6 O 0 1 N N N N N N -3.149 57.908 -7.847 -1.082 0.968 -0.513 O6 SIA 17\nSIA O7 O7 O 0 1 N N N N N N -3.594 58.883 -10.402 1.588 1.586 -1.183 O7 SIA 18\nSIA O8 O8 O 0 1 N N N N N N -6.119 56.946 -8.828 0.035 -1.313 - 2.797 O8 SIA 19\nSIA O9 O9 O 0 1 N N N N N N -6.931 57.687 -11.346 1.133 0.014 -5.035 O9 SIA 20\nSIA O10 O10 O 0 1 N N N N N N -1.423 56.357 -12.331 2.624 0.753 2.772 O10 SIA 21\nSIA H32 H31 H 0 1 N N N N N N -0.702 56.484 -6.300 -2.631 0.655 2.448 H32 SIA 22\nSIA H31 H32 H 0 1 N N N N N N 0.120 57.408 -7.182 -1.526 1.949 1.919 H31 SIA 23\nSIA H4 H4 H 0 1 N N N N N N -1.651 55.060 -7.897 -0.878 -1.026 2.153 H4 SIA 24\nSIA H5 H5 H 0 1 N N N N N N -1.506 57.375 -9.979 0.893 1.287 1.240 H5 SIA 25\nSIA H6 H6 H 0 1 N N N N N N -3.850 56.075 -8.492 - 0.408 -0.973 -0.341 H6 SIA 26\nSIA H7 H7 H 0 1 N N N N N N -4.339 57.176 -10.907 1.896 -0.454 -1.093 H7 SIA 27\nSIA H8 H8 H 0 1 N N N N N N -5.473 58.871 -8.553 -0.272 0.728 -2.887 H8 SIA 28\nSIA H92 H91 H 0 1 N N N N N N -6.054 59.459 -10.925 2.031 1.247 -3.642 H92 SIA 29\nSIA H91 H92 H 0 1 N N N N N N -7.587 59.029 -10.055 2.457 -0.476 - 3.528 H91 SIA 30\nSIA H111 H111 H 0 0 N N N N N N -3.215 53.728 - 12.207 4.474 -0.844 3.172 H111 SIA 31\nSIA H113 H112 H 0 0 N N N N N N -1.550 53.279 -12.033 3.368 -2.213 2.907 H113 SIA 32\nSIA H112 H113 H 0 0 N N N N N N -2.005 54.041 -13.531 4.266 -1.516 1.537 H112 SIA

33\nSIA HN5 HN5 H 0 1 N N N N N N -2.566 54.658 -10.003 1.635 -1.538 0.957 HN5 SIA 34\nSIA HO1B HOB1 H 0 0 N N N N N N -3.412 60.032 -4.867 -3.353 -2.430 1.044 HO1B SIA 35\nSIA HO4 HO4 H 0 1 N Y N N N N 0.427 54.801 -8.430 -0.854 0.203 4.087 HO4 SIA 37\nSIA HO7 HO7 H 0 1 N Y N N N N -3.109 58.884 -9.548 0.844 2.177 -1.360 HO7 SIA 38\nSIA HO8 HO8 H 0 1 N Y N N N N -7.071 57.051 -8.962 0.779 -1.904 -2.620 HO8 SIA 39\nSIA HO9 HO9 H 0 1 N Y N N N N -6.783 56.885 -10.808 1.866 0.155 -5.650 HO9 SIA
40\n#\nloop_\n_chem_comp_bond.comp_id\n_chem_comp_bond.atom_id_1\n_chem_comp_bond.atom_id_2\n_chem_comp_bond.value_order\n_chem_comp_bond.pdbx_aromatic_flag\n_chem_comp_bond.pdbx_stereo_config\n_chem_comp_bond.pdbx_ordinal\nSIA C1 C2 SING N N 1\nSIA C1 O1A DOUB N N 2\nSIA C1 O1B SING N N 3\nSIA C2 C3 SING N N 4\nSIA C2 O6 SING N N 6\nSIA C3 C4 SING N N 7\nSIA C3 H32 SING N N 8\nSIA C3 H31 SING N N 9\nSIA C4 C5 SING N N 10\nSIA C4 O4 SING N N 11\nSIA C4 H4 SING N N 12\nSIA C5 C6 SING N N 13\nSIA C5 N5 SING N N 14\nSIA C5 H5 SING N N 15\nSIA C6 C7 SING N N 16\nSIA C6 O6 SING N N 17\nSIA C6 H6 SING N N 18\nSIA C7 C8 SING N N 19\nSIA C7 O7 SING N N 20\nSIA C7 H7 SING N N 21\nSIA C8 C9 SING N N 22\nSIA C8 O8 SING N N 23\nSIA C8 H8 SING N N 24\nSIA C9 O9 SING N N 25\nSIA C9 H92 SING N N 26\nSIA C9 H91 SING N N 27\nSIA C10 C11 SING N N 28\nSIA C10 N5 SING N N 29\nSIA C10 O10 DOUB N N 30\nSIA C11 H111 SING N N 31\nSIA C11 H113 SING N N 32\nSIA C11 H112 SING N N 33\nSIA N5 HN5 SING N N 34\nSIA O1B HO1B SING N N 35\nSIA O4 HO4 SING N N 37\nSIA O7 HO7 SING N N 38\nSIA O8 HO8 SING N N 39\nSIA O9 HO9 SING N N
40\n#\nloop_\n_pdbx_chem_comp_descriptor.comp_id\n_pdbx_chem_comp_descriptor.type\n_pdbx_chem_comp_descriptor.program\n_pdbx_chem_comp_descriptor.program_version\n_pdbx_chem_comp_descriptor.descriptor\nSIA SMILES ACDLabs 10.04 'O=C(O)C1(O)OC(C(O)C(O)CO)C(NC(=O)C)C(O)C1'\nSIA SMILES_CANONICAL CACTVS 3.341
'CC(=O)N[C@@H]1[C@@H](O)C[C@@](O)(O[C@H]1[C@H](O)[C@H](O)CO)C(O)=O'\nSIA SMILES CACTVS 3.341
'CC(=O)N[CH]1[CH](O)C[C](O)(O[CH]1[CH](O)[CH](O)CO)C(O)=O'\nSIA SMILES_CANONICAL 'OpenEye OEToolkits' 1.5.0
'CC(=O)N[C@@H]1[C@H](C[C@@](O[C@H]1[C@@H]([C@@H](CO)O)O)(C(=O)O)O)O'\nSIA SMILES 'OpenEye OEToolkits' 1.5.0
'CC(=O)NC1C(CC(OC1C(C(CO)O)O)(C(=O)O)O)O'\nSIA InChI InChI 1.03
'InChI=1S/C11H19NO9/c1-4(14)12-7-5(15)2-11(20,10(18)19)21-9(7)8(17)6(16)3-13/h5-9,13,15-17,20H,2-3H2,1H3,(H,12,14)(H,18,19)/t5-,6+,7+,8+,9+,11+/m0/s1'\nSIA InChIKey InChI 1.03 SQVRNKJHWKZAKO-YRMXFSIDSA-N\n#\nloop_\n_pdbx_chem_comp_identifier.comp_id\n_pdbx_chem_comp_identifier.type\n_pdbx_chem_comp_identifier.program\n_pdbx_chem_comp_identifier.program_version\n_pdbx_chem_comp_identifier.identifier\nSIA 'SYSTEMATIC NAME' ACDLabs 10.04 '5-(acetylamino)-3,5-dideoxy-D-glycero-alpha-D-galacto-non-2-ulopyranosonic acid'\nSIA 'SYSTEMATIC NAME' 'OpenEye OEToolkits' 1.5.0 '(2R,4S,5R,6R)-5-acetamido-2,4-dihydroxy-6-[(1R,2R)-1,2,3-trihydroxypropyl]oxane-2-carboxylic acid'\nSIA 'CONDENSED IUPAC CARBOHYDRATE SYMBOL' GMML 1.0 DNeup5Aca\nSIA 'COMMON NAME' GMML 1.0 'N-acetyl-a-D-neuraminic acid'\nSIA 'IUPAC CARBOHYDRATE SYMBOL' PDB-CARE 1.0 a-D-Neup5Ac\nSIA 'SNFG CARBOHYDRATE SYMBOL' GMML 1.0

Neu5Ac\n#\nloop_\n_pdbx_chem_comp_feature.comp_id\n_pdbx_chem_comp_feature.type\n_pdbx_chem_comp_feature.value\n_pdbx_chem_comp_feature.source\n_pdbx_chem_comp_feature.support\nSIA 'CARBOHYDRATE ISOMER' D PDB ?\nSIA 'CARBOHYDRATE RING' pyranose PDB ?\nSIA 'CARBOHYDRATE ANOMER' alpha PDB ?\nSIA 'CARBOHYDRATE PRIMARY CARBONYL GROUP' ketose PDB ?\n#\nloop_\n_pdbx_chem_comp_audit.comp_id\n_pdbx_chem_comp_audit.action_type\n_pdbx_chem_comp_audit.date\n_pdbx_chem_comp_audit.processing_site\nSIA 'Create component' 1999-07-08 EBI\nSIA 'Modify descriptor' 2011-06-04 RCSB\nSIA 'Other modification' 2019-08-12 RCSB\nSIA 'Other modification' 2019-12-19 RCSB\nSIA 'Other modification' 2020-07-03 RCSB\nSIA 'Modify name' 2020-07-17 RCSB\nSIA 'Modify synonyms' 2020-07-17 RCSB\nSIA 'Modify atom id' 2020-07-17 RCSB\nSIA 'Modify component atom id' 2020-07-17 RCSB\nSIA 'Modify PCM' 2024-09-27 PDBE\n#\n_pdbx_chem_comp_pcm.pcm_id 1\n_pdbx_chem_comp_pcm.comp_id SIA\n_pdbx_chem_comp_pcm.modified_residue_id THR\n_pdbx_chem_comp_pcm.type None\n_pdbx_chem_comp_pcm.category Carbohydrate\n_pdbx_chem_comp_pcm.position 'Amino-acid side chain'\n_pdbx_chem_comp_pcm.polypeptide_position 'Any position'\n_pdbx_chem_comp_pcm.comp_id_linking_atom C2\n_pdbx_chem_comp_pcm.modified_residue_id_linking_atom OG1\n_pdbx_chem_comp_pcm.uniprot_specific_ptm_accession ?\n_pdbx_chem_comp_pcm.uniprot_generic_ptm_accession ?\n#\n_pdbe_chem_comp_drugbank_details.comp_id SIA\n_pdbe_chem_comp_drugbank_details.drugbank_id DB03721\n_pdbe_chem_comp_drugbank_details.type 'small molecule'\n_pdbe_chem_comp_drugbank_details.name 'N-acetyl-alpha-neuraminic acid'\n_pdbe_chem_comp_drugbank_details.description\n'An N-acyl derivative of neuraminic acid. N-acetylneuraminic acid occurs in many polysaccharides, glycoproteins, and glycolipids in animals and bacteria. (From Dorland, 28th ed, p1518)'\n_pdbe_chem_comp_drugbank_details.cas_number 21646-00-4\n_pdbe_chem_comp_drugbank_details.mechanism_of_action ?\n#\nloop_\n_pdbe_chem_comp_synonyms.comp_id\n_pdbe_chem_comp_synonyms.name\n_pdbe_chem_comp_synonyms.provenance\n_pdbe_chem_comp_synonyms.type\nSIA 'N-acetylneuraminic acid' wwPDB ?\nSIA 'sialic acid' wwPDB ?\nSIA 'alpha-sialic acid' wwPDB ?\nSIA 'O-SIALIC ACID' wwPDB ?\nSIA 'N-Acetyl-alpha-D-neuraminic acid' DrugBank ?\nSIA 'O-sialic acid' DrugBank ?\nSIA 'α-Neu5Ac' DrugBank ?\n#\n_pdbe_chem_comp_drugbank_classification.comp_id SIA\n_pdbe_chem_comp_drugbank_classification.drugbank_id DB03721\n_pdbe_chem_comp_drugbank_classification.parent 'N-acylneuraminic acids'\n_pdbe_chem_comp_drugbank_classification.kingdom 'Organic compounds'\n_pdbe_chem_comp_drugbank_classification.class 'Organooxygen compounds'\n_pdbe_chem_comp_drugbank_classification.superclass 'Organic oxygen compounds'\n_pdbe_chem_comp_drugbank_classification.description\n'This compound belongs to the class of organic compounds known as n-acylneuraminic acids. These are neuraminic acids carrying an N-acyl substituent.'\n#\nloop_\n_pdbe_chem_comp_drugbank_targets.comp_id\n_pdbe_chem_comp_drugbank_targets.drugbank_id\n_pdbe_chem_comp_drugbank_targets.name\n_pdbe_chem_comp_drugbank_targets.organism\n_pdbe_chem_comp

_drugbank_targets.uniprot_id\n_pdbe_chem_comp_drugbank_targets.pharmac
ologically_active\n_pdbe_chem_comp_drugbank_targets.ordinal\nSIA
DB03721 P-selectin Humans P16109 yes 1\nSIA DB03721 E-selectin Humans
P16581 yes 2\nSIA DB03721 'Liver carboxylesterase 1' Humans P23141 yes
3\nSIA DB03721 '3-deoxy-manno-octulosonate cytidylyltransferase'
'Escherichia coli' P42216 unknown 4\nSIA DB03721 'Tetanus toxin'
'Clostridium tetani (strain Massachusetts / E88)' P04958 unknown
5\nSIA DB03721 'Cholera enterotoxin subunit B' 'Vibrio cholerae
serotype O1 (strain ATCC 39315 / El Tor Inaba N16961)' P01556 unknown
6\nSIA DB03721 'Botulinum neurotoxin type B' 'Clostridium botulinum'
P10844 unknown 7\nSIA DB03721 'Mannose-binding protein C' Humans
P11226 unknown 8\nSIA DB03721 Lithostathine-1-alpha Humans P05451
unknown 9\nSIA DB03721 Endo-N-acetylneuraminidase 'Enterobacteria
phage K1F' Q04830 unknown 10\nSIA DB03721 'Enterotoxin type B'
'Staphylococcus aureus' P01552 unknown 11\nSIA DB03721 Neuraminidase
'Influenza A virus (strain A/Tern/Australia/G70C/1975 H11N9)' P03472
unknown 12\nSIA DB03721 Hemagglutinin-neuraminidase NDV P32884 unknown
13\nSIA DB03721 Fiber 'Human adenovirus 19' Q64822 unknown 14\nSIA
DB03721 Sialoadhesin Humans Q9BZZ2 unknown 15\nSIA DB03721 Zinc-alpha-
2-glycoprotein Humans P25311 unknown 16\nSIA DB03721 'Capsid protein
VP1' MPyV P49302 unknown 17\nSIA DB03721 Fiber 'Human adenovirus D37'
Q64823 unknown
18\n#\nloop_\n_software.name\n_software.version\n_software.description
\nrdkit 2023.09.6 'Core functionality.'\npdbeccdutils 0.8.6 'Wrapper
to provide 2D templates and molecular
fragments.'\n#\nloop_\n_pdbe_chem_comp_atom_depiction.comp_id\n_pdbe_c
hem_comp_atom_depiction.atom_id\n_pdbe_chem_comp_atom_depiction.elemen
t\n_pdbe_chem_comp_atom_depiction.model_Cartn_x\n_pdbe_chem_comp_atom_
depiction.model_Cartn_y\n_pdbe_chem_comp_atom_depiction.pdbx_ordinal\n
SIA C1 C 5.654 -3.375 1\nSIA C2 C 6.404 -2.076 2\nSIA C3 C 5.104 -
1.326 3\nSIA C4 C 5.104 0.174 4\nSIA C5 C 6.404 0.924 5\nSIA C6 C
7.702 0.174 6\nSIA C7 C 9.002 0.924 7\nSIA C8 C 10.301 0.174 8\nSIA C9
C 11.600 0.924 9\nSIA C10 C 5.104 3.174 10\nSIA C11 C 3.805 2.424
11\nSIA N5 N 6.404 2.424 12\nSIA O1A O 4.154 -3.375 13\nSIA O1B O
6.404 -4.674 14\nSIA O4 O 3.805 0.924 16\nSIA O6 O 7.702 -1.326
17\nSIA O7 O 9.002 2.424 18\nSIA O8 O 10.301 -1.326 19\nSIA O9 O
12.899 0.174 20\nSIA O10 O 5.104 4.674
21\n#\nloop_\n_pdbe_chem_comp_bond_depiction.comp_id\n_pdbe_chem_comp_
bond_depiction.atom_id_1\n_pdbe_chem_comp_bond_depiction.atom_id_2\n_p
dbe_chem_comp_bond_depiction.value_order\n_pdbe_chem_comp_bond_depicti
on.bond_dir\n_pdbe_chem_comp_bond_depiction.pdbx_ordinal\nSIA C1 C2
SINGLE NONE 1\nSIA C1 O1A DOUBLE NONE 2\nSIA C1 O1B SINGLE NONE 3\nSIA
C2 C3 SINGLE NONE 4\nSIA C2 O6 SINGLE NONE 6\nSIA C3 C4 SINGLE NONE
7\nSIA C4 C5 SINGLE NONE 8\nSIA C4 O4 SINGLE BEGINDASH 9\nSIA C5 C6
SINGLE NONE 10\nSIA C5 N5 SINGLE BEGINWEDGE 11\nSIA C6 C7 SINGLE NONE
12\nSIA C6 O6 SINGLE BEGINDASH 13\nSIA C7 C8 SINGLE NONE 14\nSIA C7 O7
SINGLE BEGINDASH 15\nSIA C8 C9 SINGLE NONE 16\nSIA C8 O8 SINGLE
BEGINWEDGE 17\nSIA C9 O9 SINGLE NONE 18\nSIA C10 C11 SINGLE NONE
19\nSIA C10 N5 SINGLE NONE 20\nSIA C10 O10 DOUBLE NONE
21\n#\nloop_\n_pdbe_chem_comp_substructure.comp_id\n_pdbe_chem_comp_su
bstructure.substructure_name\n_pdbe_chem_comp_substructure.id\n_pdbe_c
hem_comp_substructure.substructure_type\n_pdbe_chem_comp_substructure.

substructure_smiles\n_pdbe_chem_comp_substructure.substructure_inchis\
n_pdbe_chem_comp_substructure.substructure_inchikeys\nSIA
MurckoScaffold S1 scaffold C1CCOCC1 InChI=1S/C5H10O/c1-2-4-6-5-3-1/h1-
5H2 DHXVGJBLRPWPCS-UHFFFAOYSA-N\nSIA amide F1 fragment CC(N)=O
InChI=1S/C2H5NO/c1-2(3)4/h1H3,(H2,3,4) DLFVBJFMPXGRIB-UHFFFAOYSA-
N\nSIA pyranose F2 fragment OC1CCCCO1 InChI=1S/C5H10O2/c6-5-3-1-2-4-7-
5/h5-6H,1-4H2 CELWCAITJAEQNL-UHFFFAOYSA-
N\n#\nloop_\n_pdbe_chem_comp_substructure_mapping.comp_id\n_pdbe_chem_
comp_substructure_mapping.atom_id\n_pdbe_chem_comp_substructure_mappin
g.substructure_id\n_pdbe_chem_comp_substructure_mapping.substructure_o
rdinal\nSIA C2 S1 1\nSIA C3 S1 1\nSIA C4 S1 1\nSIA C5 S1 1\nSIA C6 S1
1\nSIA O6 S1 1\nSIA N5 F1 1\nSIA C10 F1 1\nSIA O10 F1 1\nSIA C11 F1
1\nSIA C5 F2 1\nSIA C6 F2 1\nSIA O6 F2 1\nSIA C2 F2 1\nSIA C3 F2
1\nSIA C4 F2 1\n#\n_pdbe_chem_comp_rdkit_properties.comp_id
SIA\n_pdbe_chem_comp_rdkit_properties.exactmw
309.106\n_pdbe_chem_comp_rdkit_properties.amw
309.271\n_pdbe_chem_comp_rdkit_properties.lipinskiHBA
10\n_pdbe_chem_comp_rdkit_properties.lipinskiHBD
7\n_pdbe_chem_comp_rdkit_properties.NumRotatableBonds
11\n_pdbe_chem_comp_rdkit_properties.NumHBD
7\n_pdbe_chem_comp_rdkit_properties.NumHBA
9\n_pdbe_chem_comp_rdkit_properties.NumHeavyAtoms
21\n_pdbe_chem_comp_rdkit_properties.NumAtoms
40\n_pdbe_chem_comp_rdkit_properties.NumHeteroatoms
10\n_pdbe_chem_comp_rdkit_properties.NumAmideBonds
1\n_pdbe_chem_comp_rdkit_properties.FractionCSP3
0.818\n_pdbe_chem_comp_rdkit_properties.NumRings
1\n_pdbe_chem_comp_rdkit_properties.NumAromaticRings
0\n_pdbe_chem_comp_rdkit_properties.NumAliphaticRings
1\n_pdbe_chem_comp_rdkit_properties.NumSaturatedRings
1\n_pdbe_chem_comp_rdkit_properties.NumHeterocycles
1\n_pdbe_chem_comp_rdkit_properties.NumAromaticHeterocycles
0\n_pdbe_chem_comp_rdkit_properties.NumSaturatedHeterocycles
1\n_pdbe_chem_comp_rdkit_properties.NumAliphaticHeterocycles
1\n_pdbe_chem_comp_rdkit_properties.NumSpiroAtoms
0\n_pdbe_chem_comp_rdkit_properties.NumBridgeheadAtoms
0\n_pdbe_chem_comp_rdkit_properties.NumAtomStereoCenters
6\n_pdbe_chem_comp_rdkit_properties.NumUnspecifiedAtomStereoCenters
0\n_pdbe_chem_comp_rdkit_properties.labuteASA
146.407\n_pdbe_chem_comp_rdkit_properties.tpsa
176.780\n_pdbe_chem_comp_rdkit_properties.CrippenClogP -
3.872\n_pdbe_chem_comp_rdkit_properties.CrippenMR
64.787\n_pdbe_chem_comp_rdkit_properties.chi0v
9.621\n_pdbe_chem_comp_rdkit_properties.chi1v
4.738\n_pdbe_chem_comp_rdkit_properties.chi2v
1.941\n_pdbe_chem_comp_rdkit_properties.chi3v
1.941\n_pdbe_chem_comp_rdkit_properties.chi4v
1.128\n_pdbe_chem_comp_rdkit_properties.chi0n
28.621\n_pdbe_chem_comp_rdkit_properties.chi1n
13.635\n_pdbe_chem_comp_rdkit_properties.chi2n
1.941\n_pdbe_chem_comp_rdkit_properties.chi3n
1.941\n_pdbe_chem_comp_rdkit_properties.chi4n

1.128\n_pdbe_chem_comp_rdkit_properties.hallKierAlpha -
1.300\n_pdbe_chem_comp_rdkit_properties.kappa1
4.600\n_pdbe_chem_comp_rdkit_properties.kappa2
6.642\n_pdbe_chem_comp_rdkit_properties.kappa3
3.872\n_pdbe_chem_comp_rdkit_properties.Phi
1.455\n#\nloop_\n_pdbe_chem_comp_external_mappings.comp_id\n_pdbe_chem
_comp_external_mappings.source\n_pdbe_chem_comp_external_mappings.reso
urce\n_pdbe_chem_comp_external_mappings.resource_id\nSIA UniChem
ChEMBL CHEMBL1234621\nSIA UniChem DrugBank DB03721\nSIA UniChem ChEBI
49026\nSIA UniChem ZINC ZINC000004081651\nSIA UniChem fdasrs
04A90EXP8V\nSIA UniChem HMDB HMDB0000773\nSIA UniChem Nikkaji
J614.853K\nSIA UniChem MetaboLights MTBLC49026\nSIA UniChem BRENDA
141715\nSIA UniChem BRENDA 233672\nSIA UniChem BRENDA 6105\nSIA
UniChem BRENDA 84245\nSIA UniChem BRENDA 85625\nSIA UniChem 'Probes
And Drugs' PD041137\nSIA UniChem PubChem 444885\nSIA UniChem
eMolecules 474793\nSIA UniChem SureChEMBL SCHEMBL79085\nSIA UniChem
'PubChem TPHARMA' 14776495\nSIA UniChem 'PubChem TPHARMA'
15395566\n#\nloop_\n_pdbe_chem_comp_rdkit_conformer.comp_id\n_pdbe_che
m_comp_rdkit_conformer.atom_id\n_pdbe_chem_comp_rdkit_conformer.Cartn_
x_rdkit\n_pdbe_chem_comp_rdkit_conformer.Cartn_y_rdkit\n_pdbe_chem_com
p_rdkit_conformer.Cartn_z_rdkit\n_pdbe_chem_comp_rdkit_conformer.rdkit
_method\n_pdbe_chem_comp_rdkit_conformer.rdkit_ordinal\nSIA C1 -1.164
-2.811 1.058 ETKDGv3 1\nSIA C2 -1.666 -1.928 -0.070 ETKDGv3 2\nSIA C3
-2.639 -0.809 0.442 ETKDGv3 3\nSIA C4 -2.546 0.454 -0.428 ETKDGv3
4\nSIA C5 -1.079 0.960 -0.528 ETKDGv3 5\nSIA C6 -0.071 -0.181 -0.219
ETKDGv3 6\nSIA C7 1.341 0.133 -0.777 ETKDGv3 7\nSIA C8 2.378 -0.960 -
0.400 ETKDGv3 8\nSIA C9 3.769 -0.651 -0.979 ETKDGv3 9\nSIA C10 -0.223
3.295 0.052 ETKDGv3 10\nSIA C11 0.035 4.333 1.094 ETKDGv3 11\nSIA N5 -
0.868 2.066 0.414 ETKDGv3 12\nSIA O1A -0.936 -2.335 2.203 ETKDGv3
13\nSIA O1B -0.875 -4.149 0.806 ETKDGv3 14\nSIA O4 -3.104 0.224 -1.700
ETKDGv3 16\nSIA O6 -0.537 -1.404 -0.765 ETKDGv3 17\nSIA O7 1.298 0.320
-2.172 ETKDGv3 18\nSIA O8 2.484 -1.066 0.998 ETKDGv3 19\nSIA O9 4.246
0.587 -0.524 ETKDGv3 20\nSIA O10 0.157 3.497 -1.133 ETKDGv3 21\nSIA
H32 -2.392 -0.510 1.484 ETKDGv3 22\nSIA H31 -3.691 -1.171 0.465
ETKDGv3 23\nSIA H4 -3.171 1.239 0.053 ETKDGv3 24\nSIA H5 -0.925 1.324
-1.568 ETKDGv3 25\nSIA H6 0.030 -0.270 0.887 ETKDGv3 26\nSIA H7 1.678
1.089 -0.320 ETKDGv3 27\nSIA H8 2.053 -1.935 -0.838 ETKDGv3 28\nSIA
H92 3.719 -0.629 -2.088 ETKDGv3 29\nSIA H91 4.478 -1.464 -0.703
ETKDGv3 30\nSIA H111 0.843 3.984 1.770 ETKDGv3 31\nSIA H113 -0.888
4.508 1.686 ETKDGv3 32\nSIA H112 0.344 5.291 0.624 ETKDGv3 33\nSIA HN5
-1.113 1.913 1.419 ETKDGv3 34\nSIA HO1B -0.511 -4.749 1.536 ETKDGv3
35\nSIA HO4 -2.492 -0.372 -2.205 ETKDGv3 37\nSIA HO7 1.040 -0.545 -
2.585 ETKDGv3 38\nSIA HO8 1.886 -1.804 1.285 ETKDGv3 39\nSIA HO9 4.592
0.441 0.395 ETKDGv3 40\n#\ndata_STE-2\n#\n_chem_comp.id
STE\n_chem_comp.name 'STEARIC ACID'\n_chem_comp.type NON-
POLYMER\n_chem_comp.pdbx_type HETAIN\n_chem_comp.formula 'C18 H36
O2'\n_chem_comp.mon_nstd_parent_comp_id ?\n_chem_comp.pdbx_synonyms ?\
n_chem_comp.pdbx_formal_charge 0\n_chem_comp.pdbx_initial_date 1999-
07-08\n_chem_comp.pdbx_modified_date 2024-09-
27\n_chem_comp.pdbx_ambiguous_flag N\n_chem_comp.pdbx_release_status
REL\n_chem_comp.pdbx_replaced_by ?\n_chem_comp.pdbx_replaces ?\n_chem_
comp.formula_weight

284.477\n_chem_comp.one_letter_code ?\n_chem_comp.three_letter_code STE\n_chem_comp.pdbx_model_coordinates_details ?\n_chem_comp.pdbx_model_coordinates_missing_flag N\n_chem_comp.pdbx_ideal_coordinates_details ?\n_chem_comp.pdbx_ideal_coordinates_missing_flag N\n_chem_comp.pdbx_model_coordinates_db_code 1HMT\n_chem_comp.pdbx_subcomponent_list ?\n_chem_comp.pdbx_processing_site RCSB\n_chem_comp.pdbx_pcm Y\n#\nloop_\n_chem_comp_atom.comp_id\n_chem_comp_atom.atom_id\n_chem_comp_atom.alt_atom_id\n_chem_comp_atom.type_symbol\n_chem_comp_atom.charge\n_chem_comp_atom.pdbx_align\n_chem_comp_atom.pdbx_aromatic_flag\n_chem_comp_atom.pdbx_leaving_atom_flag\n_chem_comp_atom.pdbx_stereo_config\n_chem_comp_atom.pdbx_backbone_atom_flag\n_chem_comp_atom.pdbx_n_terminal_atom_flag\n_chem_comp_atom.pdbx_c_terminal_atom_flag\n_chem_comp_atom.model_Cartn_x\n_chem_comp_atom.model_Cartn_y\n_chem_comp_atom.model_Cartn_z\n_chem_comp_atom.pdbx_model_Cartn_x_ideal\n_chem_comp_atom.pdbx_model_Cartn_y_ideal\n_chem_comp_atom.pdbx_model_Cartn_z_ideal\n_chem_comp_atom.pdbx_component_atom_id\n_chem_comp_atom.pdbx_component_comp_id\n_chem_comp_atom.pdbx_ordinal\nSTE C1 C1 C 0 1 N N N N N N 19.059 33.240 52.247 0.160 0.001 -9.422 C1 STE 1\nSTE O1 O1 O 0 1 N N N N N N 18.383 32.247 52.074 1.365 0.014 -9.335 O1 STE 2\nSTE C2 C2 C 0 1 N N N N N N 20.059 33.155 53.319 -0.684 -0.007 -8.174 C2 STE 4\nSTE C3 C3 C 0 1 N N N N N N 20.932 34.263 53.667 0.223 0.002 -6.943 C3 STE 5\nSTE C4 C4 C 0 1 N N N N N N 21.956 34.081 54.790 -0.634 -0.006 -5.676 C4 STE 6\nSTE C5 C5 C 0 1 N N N N N N 22.334 35.382 55.440 0.274 0.003 -4.445 C5 STE 7\nSTE C6 C6 C 0 1 N N N N N N 23.534 35.332 56.339 -0.584 -0.006 -3.178 C6 STE 8\nSTE C7 C7 C 0 1 N N N N N N 23.417 34.451 57.545 0.324 0.003 -1.947 C7 STE 9\nSTE C8 C8 C 0 1 N N N N N N 22.312 34.887 58.510 -0.533 -0.005 -0.680 C8 STE 10\nSTE C9 C9 C 0 1 N N N N N N 22.118 33.940 59.640 0.374 0.004 0.550 C9 STE 11\nSTE C10 C10 C 0 1 N N N N N N 21.237 34.529 60.741 -0.483 -0.005 1.817 C10 STE 12\nSTE C11 C11 C 0 1 N N N N N N 19.925 35.023 60.159 0.424 0.004 3.048 C11 STE 13\nSTE C12 C12 C 0 1 N N N N N N 19.184 34.008 59.369 -0.433 -0.004 4.315 C12 STE 14\nSTE C13 C13 C 0 1 N N N N N N 17.705 34.094 59.394 0.475 0.005 5.546 C13 STE 15\nSTE C14 C14 C 0 1 N N N N N N 17.002 34.893 58.383 -0.382 -0.004 6.813 C14 STE 16\nSTE C15 C15 C 0 1 N N N N N N 17.078 34.335 56.971 0.525 0.005 8.044 C15 STE 17\nSTE C16 C16 C 0 1 N N N N N N 16.780 35.466 55.970 -0.332 -0.003 9.311 C16 STE 18\nSTE C17 C17 C 0 1 N N N N N N 16.099 36.593 56.719 0.575 0.006 10.542 C17 STE 19\nSTE C18 C18 C 0 1 N N N N N N 16.704 37.939 56.339 -0.282 -0.003 11.809 C18 STE 20\nSTE H21 1H2 H 0 1 N N N N N N 19.528 32.840 54.248 -1.304 -0.904 -8.162 H21 STE 22\nSTE H22 2H2 H 0 1 N N N N N N 20.705 32.272 53.105 -1.323 0.875 -8.162 H22 STE 23\nSTE H31 1H3 H 0 1 N N N N N N 21.464 34.601 52.747 0.843 0.899 -6.956 H31 STE 24\nSTE H32 2H3 H 0 1 N N N N N N 20.304 35.155 53.896 0.862 -0.880 -6.956 H32 STE 25\nSTE H41 1H4 H 0 1 N N N N N N 21.596 33.344 55.545 -1.253 -0.903 -5.664 H41 STE 26\nSTE H42 2H4 H 0 1 N N N N N N 22.858 33.537 54.425 -1.273 0.876 -5.664 H42 STE 27\nSTE H51 1H5 H 0 1 N N N N N N 22.472 36.170 54.663 0.893 0.899 -4.458 H51 STE 28\nSTE H52 2H5 H 0 1 N N N N N N 21.460 35.799 55.993 0.912 -0.880 -4.458 H52 STE 29\nSTE H61 1H6 H 0 1 N N N N N N 24.436 35.047 55.749 -1.203 -0.903 -3.166 H61 STE 30\nSTE H62 2H6 H 0 1 N N N N N N 23.815 36.364 56.651 -1.222 0.876 -3.166 H62 STE 31\nSTE H71 1H7

H 0 1 N N N N N N 23.282 33.385 57.245 0.943 0.900 -1.960 H71 STE 32\nSTE H72 2H7 H 0 1 N N N N N N 24.395 34.371 58.073 0.963 -0.879 -1.960 H72 STE 33\nSTE H81 1H8 H 0 1 N N N N N N 22.496 35.920 58.884 -1.153 -0.902 -0.668 H81 STE 34\nSTE H82 2H8 H 0 1 N N N N N N 21.353 35.058 57.966 -1.172 0.877 -0.668 H82 STE 35\nSTE H91 1H9 H 0 1 N N N N N N 21.718 32.962 59.283 0.993 0.900 0.537 H91 STE 36\nSTE H92 2H9 H 0 1 N N N N N N 23.095 33.592 60.048 1.013 -0.879 0.537 H92 STE 37\nSTE H101 1H10 H 0 0 N N N N N N 21.076 33.807 61.575 -1.102 -0.902 1.829 H101 STE 38\nSTE H102 2H10 H 0 0 N N N N N N 21.764 35.327 61.313 -1.122 0.877 1.829 H102 STE 39\nSTE H111 1H11 H 0 0 N N N N N N 19.275 35.439 60.963 1.044 0.901 3.035 H111 STE 40\nSTE H112 2H11 H 0 0 N N N N N N 20.091 35.943 59.551 1.063 -0.878 3.035 H112 STE 41\nSTE H121 1H12 H 0 0 N N N N N N 19.541 34.022 58.312 -1.052 -0.901 4.327 H121 STE 42\nSTE H122 2H12 H 0 0 N N N N N N 19.504 32.986 59.679 -1.072 0.878 4.327 H122 STE 43\nSTE H131 1H13 H 0 0 N N N N N N 17.290 33.059 59.377 1.094 0.902 5.533 H131 STE 44\nSTE H132 2H13 H 0 0 N N N N N N 17.388 34.444 60.404 1.113 -0.877 5.533 H132 STE 45\nSTE H141 1H14 H 0 0 N N N N N N 15.939 35.048 58.681 -1.002 -0.901 6.825 H141 STE 46\nSTE H142 2H14 H 0 0 N N N N N N 17.362 35.947 58.402 -1.021 0.878 6.825 H142 STE 47\nSTE H151 1H15 H 0 0 N N N N N N 18.050 33.830 56.765 1.144 0.902 8.031 H151 STE 48\nSTE H152 2H15 H 0 0 N N N N N N 16.411 33.452 56.826 1.164 -0.877 8.031 H152 STE 49\nSTE H161 1H16 H 0 0 N N N N N N 17.688 35.803 55.419 -0.952 -0.900 9.323 H161 STE 50\nSTE H162 2H16 H 0 0 N N N N N N 16.191 35.117 55.089 -0.971 0.879 9.323 H162 STE 51\nSTE H171 1H17 H 0 0 N N N N N N 14.994 36.580 56.568 1.195 0.903 10.529 H171 STE 52\nSTE H172 2H17 H 0 0 N N N N N N 16.120 36.427 57.821 1.214 -0.876 10.529 H172 STE 53\nSTE H181 1H18 H 0 0 N N N N N N 16.202 38.768 56.890 0.364 0.004 12.686 H181 STE 54\nSTE H182 2H18 H 0 0 N N N N N N 17.808 37.952 56.489 -0.921 0.880 11.821 H182 STE 55\nSTE H183 3H18 H 0 0 N N N N N N 16.682 38.104 55.236 -0.901 -0.899 11.821 H183 STE 56\n#\nloop_\n_chem_comp_bond.comp_id\n_chem_comp_bond.atom_id_1\n_chem_comp_bond.atom_id_2\n_chem_comp_bond.value_order\n_chem_comp_bond.pdbx_aromatic_flag\n_chem_comp_bond.pdbx_stereo_config\n_chem_comp_bond.pdbx_ordinal\nSTE C1 O1 DOUB N N 1\nSTE C1 C2 SING N N 3\nSTE C2 C3 SING N N 5\nSTE C2 H21 SING N N 6\nSTE C2 H22 SING N N 7\nSTE C3 C4 SING N N 8\nSTE C3 H31 SING N N 9\nSTE C3 H32 SING N N 10\nSTE C4 C5 SING N N 11\nSTE C4 H41 SING N N 12\nSTE C4 H42 SING N N 13\nSTE C5 C6 SING N N 14\nSTE C5 H51 SING N N 15\nSTE C5 H52 SING N N 16\nSTE C6 C7 SING N N 17\nSTE C6 H61 SING N N 18\nSTE C6 H62 SING N N 19\nSTE C7 C8 SING N N 20\nSTE C7 H71 SING N N 21\nSTE C7 H72 SING N N 22\nSTE C8 C9 SING N N 23\nSTE C8 H81 SING N N 24\nSTE C8 H82 SING N N 25\nSTE C9 C10 SING N N 26\nSTE C9 H91 SING N N 27\nSTE C9 H92 SING N N 28\nSTE C10 C11 SING N N 29\nSTE C10 H101 SING N N 30\nSTE C10 H102 SING N N 31\nSTE C11 C12 SING N N 32\nSTE C11 H111 SING N N 33\nSTE C11 H112 SING N N 34\nSTE C12 C13 SING N N 35\nSTE C12 H121 SING N N 36\nSTE C12 H122 SING N N 37\nSTE C13 C14 SING N N 38\nSTE C13 H131 SING N N 39\nSTE C13 H132 SING N N 40\nSTE C14 C15 SING N N 41\nSTE C14 H141 SING N N 42\nSTE C14 H142 SING N N 43\nSTE C15 C16 SING N N 44\nSTE C15 H151 SING N N 45\nSTE C15 H152 SING N N 46\nSTE C16 C17 SING N N 47\nSTE C16 H161 SING N N 48\nSTE C16 H162 SING N N 49\nSTE C17 C18 SING N N 50\nSTE C17 H171 SING N N 51\nSTE C17 H172 SING N N 52\nSTE

C18 H181 SING N N 53\nSTE C18 H182 SING N N 54\nSTE C18 H183 SING N N 55\n#\nloop_\n_pdbx_chem_comp_descriptor.comp_id\n_pdbx_chem_comp_descriptor.type\n_pdbx_chem_comp_descriptor.program\n_pdbx_chem_comp_descriptor.program_version\n_pdbx_chem_comp_descriptor.descriptor\nSTE SMILES ACDLabs 10.04 'O=C(O)CCCCCCCCCCCCCCCCC'\nSTE SMILES_CANONICAL CACTVS 3.341 'CCCCCCCCCCCCCCCCCC(O)=O'\nSTE SMILES CACTVS 3.341 'CCCCCCCCCCCCCCCCCC(O)=O'\nSTE SMILES_CANONICAL 'OpenEye OEToolkits' 1.5.0 'CCCCCCCCCCCCCCCCCC(=O)O'\nSTE SMILES 'OpenEye OEToolkits' 1.5.0 'CCCCCCCCCCCCCCCCCC(=O)O'\nSTE InChI InChI 1.03 'InChI=1S/C18H36O2/c1-2-3-4-5-6-7-8-9-10-11-12-13-14-15-16-17-18(19)20/h2-17H2,1H3,(H,19,20)'\nSTE InChIKey InChI 1.03 QIQXTHQIDYTFRH-UHFFFAOYSA-N\n#\nloop_\n_pdbx_chem_comp_identifier.comp_id\n_pdbx_chem_comp_identifier.type\n_pdbx_chem_comp_identifier.program\n_pdbx_chem_comp_identifier.program_version\n_pdbx_chem_comp_identifier.identifier\nSTE 'SYSTEMATIC NAME' ACDLabs 10.04 'octadecanoic acid'\nSTE 'SYSTEMATIC NAME' 'OpenEye OEToolkits' 1.5.0 'octadecanoic acid'\n#\nloop_\n_pdbx_chem_comp_audit.comp_id\n_pdbx_chem_comp_audit.action_type\n_pdbx_chem_comp_audit.date\n_pdbx_chem_comp_audit.processing_site\nSTE 'Create component' 1999-07-08 RCSB\nSTE 'Modify descriptor' 2011-06-04 RCSB\nSTE 'Modify PCM' 2024-09-27 PDBE\n#\nloop_\n_pdbx_chem_comp_pcm.pcm_id\n_pdbx_chem_comp_pcm.comp_id\n_pdbx_chem_comp_pcm.modified_residue_id\n_pdbx_chem_comp_pcm.type\n_pdbx_chem_comp_pcm.category\n_pdbx_chem_comp_pcm.position\n_pdbx_chem_comp_pcm.polypeptide_position\n_pdbx_chem_comp_pcm.comp_id_linking_atom\n_pdbx_chem_comp_pcm.modified_residue_id_linking_atom\n_pdbx_chem_comp_pcm.uniprot_specific_ptm_accession\n_pdbx_chem_comp_pcm.uniprot_generic_ptm_accession\n1 STE CYS Stearoylation Lipid/lipid-like 'Amino-acid side chain' 'Any position' C1 SG PTM-0283 ?\n2 STE LYS Stearoylation Lipid/lipid-like 'Amino-acid side chain' 'Any position' C1 NZ PTM-0717 ?\n3 STE 4HH Stearoylation Lipid/lipid-like 'Amino-acid side chain' 'Any position' C1 SU ? ?\n#\n_pdbe_chem_comp_drugbank_details.comp_id STE\n_pdbe_chem_comp_drugbank_details.drugbank_id DB03193\n_pdbe_chem_comp_drugbank_details.type 'small molecule'\n_pdbe_chem_comp_drugbank_details.name 'Stearic acid'\n_pdbe_chem_comp_drugbank_details.description\n'Stearic acid (IUPAC systematic name: octadecanoic acid) is one of the useful types of saturated fatty acids that comes from many animal and vegetable fats and oils. It is a waxy solid.'\n_pdbe_chem_comp_drugbank_details.cas_number 57-11-4\n_pdbe_chem_comp_drugbank_details.mechanism_of_action ?\n#\nloop_\n_pdbe_chem_comp_synonyms.comp_id\n_pdbe_chem_comp_synonyms.name\n_pdbe_chem_comp_synonyms.provenance\n_pdbe_chem_comp_synonyms.type\nSTE C18:0 DrugBank ?\nSTE 'n-octadecanoic acid' DrugBank ?\nSTE 'Octadecanoic acid' DrugBank ?\nSTE 'Octadecoic acid' DrugBank ?\nSTE 'Stearic acid' DrugBank ?\n#\n_pdbe_chem_comp_drugbank_classification.comp_id STE\n_pdbe_chem_comp_drugbank_classification.drugbank_id DB03193\n_pdbe_chem_comp_drugbank_classification.parent 'Long-chain fatty acids'\n_pdbe_chem_comp_drugbank_classification.kingdom 'Organic compounds'\n_pdbe_chem_comp_drugbank_classification.class 'Fatty

Acyls'\n_pdbe_chem_comp_drugbank_classification.superclass 'Lipids and lipid-like
molecules'\n_pdbe_chem_comp_drugbank_classification.description\n'This compound belongs to the class of organic compounds known as long-chain fatty acids. These are fatty acids with an aliphatic tail that contains between 13 and 21 carbon
atoms.'\n#\nloop_\n_pdbe_chem_comp_drugbank_targets.comp_id\n_pdbe_chem_comp_drugbank_targets.drugbank_id\n_pdbe_chem_comp_drugbank_targets.name\n_pdbe_chem_comp_drugbank_targets.organism\n_pdbe_chem_comp_drugbank_targets.uniprot_id\n_pdbe_chem_comp_drugbank_targets.pharmacologically_active\n_pdbe_chem_comp_drugbank_targets.ordinal\nSTE DB03193 'Peroxisome proliferator-activated receptor alpha' Humans Q07869 unknown 1\nSTE DB03193 'Group IID secretory phospholipase A2' Humans Q9UNK4 unknown
2\n#\nloop_\n_software.name\n_software.version\n_software.description\nrdkit 2023.09.6 'Core functionality.'\npdbeccdutils 0.8.6 'Wrapper to provide 2D templates and molecular
fragments.'\n#\nloop_\n_pdbe_chem_comp_atom_depiction.comp_id\n_pdbe_chem_comp_atom_depiction.atom_id\n_pdbe_chem_comp_atom_depiction.element\n_pdbe_chem_comp_atom_depiction.model_Cartn_x\n_pdbe_chem_comp_atom_depiction.model_Cartn_y\n_pdbe_chem_comp_atom_depiction.pdbx_ordinal\nSTE C1 C 5.104 0.375 1\nSTE O1 O 3.805 1.125 2\nSTE C2 C 6.404 1.125 4\nSTE C3 C 7.702 0.375 5\nSTE C4 C 9.002 1.125 6\nSTE C5 C 10.301 0.375 7\nSTE C6 C 11.600 1.125 8\nSTE C7 C 12.899 0.375 9\nSTE C8 C 14.198 1.125 10\nSTE C9 C 15.497 0.375 11\nSTE C10 C 16.796 1.125 12\nSTE C11 C 18.095 0.375 13\nSTE C12 C 19.394 1.125 14\nSTE C13 C 20.693 0.375 15\nSTE C14 C 21.992 1.125 16\nSTE C15 C 23.291 0.375 17\nSTE C16 C 24.590 1.125 18\nSTE C17 C 25.889 0.375 19\nSTE C18 C 27.188 1.125
20\n#\nloop_\n_pdbe_chem_comp_bond_depiction.comp_id\n_pdbe_chem_comp_bond_depiction.atom_id_1\n_pdbe_chem_comp_bond_depiction.atom_id_2\n_pdbe_chem_comp_bond_depiction.value_order\n_pdbe_chem_comp_bond_depiction.bond_dir\n_pdbe_chem_comp_bond_depiction.pdbx_ordinal\nSTE C1 O1 DOUBLE NONE 1\nSTE C1 C2 SINGLE NONE 3\nSTE C2 C3 SINGLE NONE 4\nSTE C3 C4 SINGLE NONE 5\nSTE C4 C5 SINGLE NONE 6\nSTE C5 C6 SINGLE NONE 7\nSTE C6 C7 SINGLE NONE 8\nSTE C7 C8 SINGLE NONE 9\nSTE C8 C9 SINGLE NONE 10\nSTE C9 C10 SINGLE NONE 11\nSTE C10 C11 SINGLE NONE 12\nSTE C11 C12 SINGLE NONE 13\nSTE C12 C13 SINGLE NONE 14\nSTE C13 C14 SINGLE NONE 15\nSTE C14 C15 SINGLE NONE 16\nSTE C15 C16 SINGLE NONE 17\nSTE C16 C17 SINGLE NONE 18\nSTE C17 C18 SINGLE NONE
19\n#\n#\n#\n_pdbe_chem_comp_rdkit_properties.comp_id
STE\n_pdbe_chem_comp_rdkit_properties.exactmw
284.272\n_pdbe_chem_comp_rdkit_properties.amw
284.484\n_pdbe_chem_comp_rdkit_properties.lipinskiHBA
2\n_pdbe_chem_comp_rdkit_properties.lipinskiHBD
1\n_pdbe_chem_comp_rdkit_properties.NumRotatableBonds
17\n_pdbe_chem_comp_rdkit_properties.NumHBD
1\n_pdbe_chem_comp_rdkit_properties.NumHBA
2\n_pdbe_chem_comp_rdkit_properties.NumHeavyAtoms
20\n_pdbe_chem_comp_rdkit_properties.NumAtoms
56\n_pdbe_chem_comp_rdkit_properties.NumHeteroatoms
2\n_pdbe_chem_comp_rdkit_properties.NumAmideBonds

0\n_pdbe_chem_comp_rdkit_properties.FractionCSP3
0.944\n_pdbe_chem_comp_rdkit_properties.NumRings
0\n_pdbe_chem_comp_rdkit_properties.NumAromaticRings
0\n_pdbe_chem_comp_rdkit_properties.NumAliphaticRings
0\n_pdbe_chem_comp_rdkit_properties.NumSaturatedRings
0\n_pdbe_chem_comp_rdkit_properties.NumHeterocycles
0\n_pdbe_chem_comp_rdkit_properties.NumAromaticHeterocycles
0\n_pdbe_chem_comp_rdkit_properties.NumSaturatedHeterocycles
0\n_pdbe_chem_comp_rdkit_properties.NumAliphaticHeterocycles
0\n_pdbe_chem_comp_rdkit_properties.NumSpiroAtoms
0\n_pdbe_chem_comp_rdkit_properties.NumBridgeheadAtoms
0\n_pdbe_chem_comp_rdkit_properties.NumAtomStereoCenters
0\n_pdbe_chem_comp_rdkit_properties.NumUnspecifiedAtomStereoCenters
0\n_pdbe_chem_comp_rdkit_properties.labuteASA
176.565\n_pdbe_chem_comp_rdkit_properties.tpsa
37.300\n_pdbe_chem_comp_rdkit_properties.CrippenClogP
6.333\n_pdbe_chem_comp_rdkit_properties.CrippenMR
87.182\n_pdbe_chem_comp_rdkit_properties.chi0v
9.816\n_pdbe_chem_comp_rdkit_properties.chi1v
4.658\n_pdbe_chem_comp_rdkit_properties.chi2v
1.040\n_pdbe_chem_comp_rdkit_properties.chi3v
1.040\n_pdbe_chem_comp_rdkit_properties.chi4v
0.489\n_pdbe_chem_comp_rdkit_properties.chi0n
45.816\n_pdbe_chem_comp_rdkit_properties.chi1n
22.566\n_pdbe_chem_comp_rdkit_properties.chi2n
1.040\n_pdbe_chem_comp_rdkit_properties.chi3n
1.040\n_pdbe_chem_comp_rdkit_properties.chi4n
0.489\n_pdbe_chem_comp_rdkit_properties.hallKierAlpha -
0.530\n_pdbe_chem_comp_rdkit_properties.kappa1
2.239\n_pdbe_chem_comp_rdkit_properties.kappa2
16.524\n_pdbe_chem_comp_rdkit_properties.kappa3
17.470\n_pdbe_chem_comp_rdkit_properties.Phi
1.850\n#\nloop_\n_pdbe_chem_comp_external_mappings.comp_id\n_pdbe_chem
_comp_external_mappings.source\n_pdbe_chem_comp_external_mappings.reso
urce\n_pdbe_chem_comp_external_mappings.resource_id\nSTE UniChem
ChEMBL CHEMBL46403\nSTE UniChem DrugBank DB03193\nSTE UniChem 'Guide
to Pharmacology' 3377\nSTE UniChem ChEBI 28842\nSTE UniChem ZINC
ZINC000004978673\nSTE UniChem fdasrs 4ELV7Z65AP\nSTE UniChem HMDB
HMDB0000827\nSTE UniChem NMRShiftDB 10016909\nSTE UniChem BindingDb
50240485\nSTE UniChem DrugCentral 4611\nSTE UniChem MetaboLights
MTBLC28842\nSTE UniChem BRENDA 101697\nSTE UniChem BRENDA 10738\nSTE
UniChem BRENDA 1359\nSTE UniChem BRENDA 180906\nSTE UniChem BRENDA
20881\nSTE UniChem BRENDA 2739\nSTE UniChem BRENDA 3687\nSTE UniChem
BRENDA 6047\nSTE UniChem BRENDA 7263\nSTE UniChem BRENDA 833\nSTE
UniChem BRENDA 97314\nSTE UniChem BRENDA 98336\nSTE UniChem
ChemicalBook CB4853859\nSTE UniChem DailyMed 'STEARIC ACID'\nSTE
UniChem ClinicalTrials 'STEARIC ACID'\nSTE UniChem rxnorm 'STEARIC
ACID'\nSTE UniChem MedChemExpress HY-B2219\nSTE UniChem 'Probes And
Drugs' PD007733\nSTE UniChem CCDC STARAC\nSTE UniChem 'EPA CompTox
Dashboard' DTXSID8021642\nSTE UniChem eMolecules 26756663\nSTE UniChem
eMolecules 477279\nSTE UniChem SureChEMBL SCHEMBL659\nSTE UniChem
'PubChem TPHARMA' 14824610\nSTE UniChem PubChem 139057051\nSTE UniChem

PubChem 5281\nSTE UniChem Mcule MCULE-5127577640\nSTE UniChem ACTor 126539-56-8\nSTE UniChem ACTor 134503-33-6\nSTE UniChem ACTor 57-11-4\nSTE UniChem ACTor 609343-71-7\nSTE UniChem ACTor 68937-76-8\nSTE UniChem Nikkaji J1.379J\n#\nloop_\n_pdbe_chem_comp_rdkit_conformer.comp_id\n_pdbe_chem_comp_rdkit_conformer.atom_id\n_pdbe_chem_comp_rdkit_conformer.Cartn_x_rdkit\n_pdbe_chem_comp_rdkit_conformer.Cartn_y_rdkit\n_pdbe_chem_comp_rdkit_conformer.Cartn_z_rdkit\n_pdbe_chem_comp_rdkit_conformer.rdkit_method\n_pdbe_chem_comp_rdkit_conformer.rdkit_ordinal\nSTE C1 5.848 2.083 -3.953 ETKDGv3 1\nSTE O1 6.394 0.973 -4.195 ETKDGv3 2\nSTE C2 4.518 2.131 -3.270 ETKDGv3 4\nSTE C3 4.702 2.228 -1.750 ETKDGv3 5\nSTE C4 3.370 2.154 -0.985 ETKDGv3 6\nSTE C5 2.547 3.448 -1.107 ETKDGv3 7\nSTE C6 1.208 3.367 -0.357 ETKDGv3 8\nSTE C7 1.382 3.386 1.172 ETKDGv3 9\nSTE C8 0.054 3.595 1.922 ETKDGv3 10\nSTE C9 -1.010 2.512 1.670 ETKDGv3 11\nSTE C10 -0.558 1.113 2.115 ETKDGv3 12\nSTE C11 -1.708 0.104 2.009 ETKDGv3 13\nSTE C12 -1.246 -1.301 2.424 ETKDGv3 14\nSTE C13 -2.399 -2.318 2.457 ETKDGv3 15\nSTE C14 -2.913 -2.676 1.054 ETKDGv3 16\nSTE C15 -3.949 -3.808 1.123 ETKDGv3 17\nSTE C16 -4.638 -4.075 -0.228 ETKDGv3 18\nSTE C17 -3.696 -4.540 -1.353 ETKDGv3 19\nSTE C18 -3.004 -5.867 -1.043 ETKDGv3 20\nSTE H21 3.940 1.216 -3.526 ETKDGv3 22\nSTE H22 3.960 3.012 -3.649 ETKDGv3 23\nSTE H31 5.337 1.378 -1.413 ETKDGv3 24\nSTE H32 5.235 3.169 -1.490 ETKDGv3 25\nSTE H41 3.609 1.975 0.084 ETKDGv3 26\nSTE H42 2.775 1.287 -1.347 ETKDGv3 27\nSTE H51 2.318 3.638 -2.177 ETKDGv3 28\nSTE H52 3.138 4.312 -0.731 ETKDGv3 29\nSTE H61 0.591 4.241 -0.660 ETKDGv3 30\nSTE H62 0.670 2.446 -0.670 ETKDGv3 31\nSTE H71 1.863 2.449 1.520 ETKDGv3 32\nSTE H72 2.060 4.223 1.449 ETKDGv3 33\nSTE H81 0.267 3.643 3.012 ETKDGv3 34\nSTE H82 -0.370 4.581 1.633 ETKDGv3 35\nSTE H91 -1.295 2.496 0.597 ETKDGv3 36\nSTE H92 -1.920 2.795 2.243 ETKDGv3 37\nSTE H101 0.278 0.768 1.469 ETKDGv3 38\nSTE H102 -0.202 1.156 3.168 ETKDGv3 39\nSTE H111 -2.543 0.428 2.668 ETKDGv3 40\nSTE H112 -2.070 0.084 0.959 ETKDGv3 41\nSTE H121 -0.450 -1.657 1.734 ETKDGv3 42\nSTE H122 -0.808 -1.248 3.444 ETKDGv3 43\nSTE H131 -2.025 -3.242 2.949 ETKDGv3 44\nSTE H132 -3.233 -1.921 3.076 ETKDGv3 45\nSTE H141 -3.383 -1.784 0.589 ETKDGv3 46\nSTE H142 -2.054 -2.998 0.428 ETKDGv3 47\nSTE H151 -3.473 -4.737 1.502 ETKDGv3 48\nSTE H152 -4.739 -3.527 1.853 ETKDGv3 49\nSTE H161 -5.425 -4.845 -0.077 ETKDGv3 50\nSTE H162 -5.153 -3.147 -0.558 ETKDGv3 51\nSTE H171 -2.935 -3.762 -1.574 ETKDGv3 52\nSTE H172 -4.301 -4.671 -2.276 ETKDGv3 53\nSTE H181 -2.469 -6.224 -1.948 ETKDGv3 54\nSTE H182 -3.750 -6.635 -0.747 ETKDGv3 55\nSTE H183 -2.258 -5.739 -0.232 ETKDGv3 56\n#\ndata_PL3-2\n#\n_chem_comp.id PL3\n_chem_comp.name HEXADECAN-1-OL\n_chem_comp.type NON-POLYMER\n_chem_comp.pdbx_type HETAIN\n_chem_comp.formula 'C16 H34 O'\n_chem_comp.mon_nstd_parent_comp_id ?\n_chem_comp.pdbx_synonyms ?\n_chem_comp.pdbx_formal_charge 0\n_chem_comp.pdbx_initial_date 2007-03-07\n_chem_comp.pdbx_modified_date 2011-06-04\n_chem_comp.pdbx_ambiguous_flag ?\n_chem_comp.pdbx_release_status REL\n_chem_comp.pdbx_replaced_by ?\n_chem_comp.pdbx_replaces ?\n_chem_comp.formula_weight 242.441\n_chem_comp.one_letter_code ?\n_chem_comp.three_letter_code PL3\n_chem_comp.pdbx_model_coordinates_details ?\n_chem_comp.pdbx_model_coordinates_missing_flag

N\n_chem_comp.pdbx_ideal_coordinates_details 'OpenEye OEToolkits'\n_chem_comp.pdbx_ideal_coordinates_missing_flag N\n_chem_comp.pdbx_model_coordinates_db_code ?\n_chem_comp.pdbx_subcomponent_list ?\n_chem_comp.pdbx_processing_site EBI\n#\nloop_\n_chem_comp_atom.comp_id\n_chem_comp_atom.atom_id\n_chem_comp_atom.alt_atom_id\n_chem_comp_atom.type_symbol\n_chem_comp_atom.charge\n_chem_comp_atom.pdbx_align\n_chem_comp_atom.pdbx_aromatic_flag\n_chem_comp_atom.pdbx_leaving_atom_flag\n_chem_comp_atom.pdbx_stereo_config\n_chem_comp_atom.pdbx_backbone_atom_flag\n_chem_comp_atom.pdbx_n_terminal_atom_flag\n_chem_comp_atom.pdbx_c_terminal_atom_flag\n_chem_comp_atom.model_Cartn_x\n_chem_comp_atom.model_Cartn_y\n_chem_comp_atom.model_Cartn_z\n_chem_comp_atom.pdbx_model_Cartn_x_ideal\n_chem_comp_atom.pdbx_model_Cartn_y_ideal\n_chem_comp_atom.pdbx_model_Cartn_z_ideal\n_chem_comp_atom.pdbx_component_atom_id\n_chem_comp_atom.pdbx_component_comp_id\n_chem_comp_atom.pdbx_ordinal\nPL3 C1 C1 C 0 1 N N N N N N 35.217 -22.177 8.964 11.542 8.497 9.204 C1 PL3 1\nPL3 C2 C2 C 0 1 N N N N N N 35.188 -23.426 9.798 10.902 8.332 7.832 C2 PL3 3\nPL3 C3 C3 C 0 1 N N N N N N 33.761 -23.973 9.879 10.239 6.962 7.662 C3 PL3 4\nPL3 C4 C4 C 0 1 N N N N N N 33.267 -23.997 11.314 9.633 6.807 6.266 C4 PL3 5\nPL3 C5 C5 C 0 1 N N N N N N 31.753 -24.039 11.342 8.996 5.428 6.085 C5 PL3 6\nPL3 C6 C6 C 0 1 N N N N N N 31.274 -25.250 10.574 8.361 5.287 4.700 C6 PL3 7\nPL3 C7 C7 C 0 1 N N N N N N 30.150 -25.921 11.343 7.754 3.895 4.509 C7 PL3 8\nPL3 C8 C8 C 0 1 N N N N N N 29.082 -26.425 10.398 7.082 3.703 3.145 C8 PL3 9\nPL3 C9 C9 C 0 1 N N N N N N 28.197 -27.389 11.165 6.409 2.337 2.988 C9 PL3 10\nPL3 CA CA C 0 1 N N N N N N 27.394 -28.306 10.273 5.675 2.171 1.653 CA PL3 11\nPL3 CB CB C 0 1 N N N N N N 27.553 -29.728 10.765 4.958 0.833 1.446 CB PL3 12\nPL3 CC CC C 0 1 N N N N N N 26.288 -30.523 10.496 4.170 0.697 0.140 CC PL3 13\nPL3 CD CD C 0 1 N N N N N N 26.341 -31.881 11.170 3.504 -0.662 -0.096 CD PL3 14\nPL3 CE CE C 0 1 N N N N N N 26.410 -33.014 10.150 2.800 -0.768 -1.450 CE PL3 15\nPL3 CF CF C 0 1 N N N N N N 25.249 -33.957 10.394 2.077 -2.108 -1.595 CF PL3 16\nPL3 CG CG C 0 1 N N N N N N 25.169 -35.038 9.338 1.343 -2.204 -2.923 CG PL3 17\nPL3 H1C1 1H1C H 0 0 N N N N N N 35.177 -22.507 7.915 12.315 7.740 9.370 H1C1 PL3 18\nPL3 H1C2 2H1C H 0 0 N N N N N N 36.123 -21.655 9.304 10.782 8.400 9.986 H1C2 PL3 19\nPL3 H2C1 1H2C H 0 0 N N N N N N 35.841 -24.183 9.340 10.157 9.122 7.687 H2C1 PL3 20\nPL3 H2C2 2H2C H 0 0 N N N N N N 35.541 -23.189 10.813 11.670 8.452 7.057 H2C2 PL3 21\nPL3 H3C1 1H3C H 0 0 N N N N N N 33.097 -23.329 9.283 9.454 6.839 8.418 H3C1 PL3 22\nPL3 H3C2 2H3C H 0 0 N N N N N N 33.757 -25.002 9.489 10.976 6.167 7.829 H3C2 PL3 23\nPL3 H4C1 1H4C H 0 0 N N N N N N 33.664 -24.889 11.820 10.413 6.954 5.509 H4C1 PL3 24\nPL3 H4C2 2H4C H 0 0 N N N N N N 33.612 -23.089 11.829 8.877 7.585 6.106 H4C2 PL3 25\nPL3 H5C1 1H5C H 0 0 N N N N N N 31.406 -24.103 12.384 8.233 5.272 6.857 H5C1 PL3 26\nPL3 H5C2 2H5C H 0 0 N N N N N N 31.350 -23.126 10.880 9.758 4.651 6.220 H5C2 PL3 27\nPL3 H6C1 1H6C H 0 0 N N N N N N 30.907 -24.937 9.586 9.125 5.472 3.936 H6C1 PL3 28\nPL3 H6C2 2H6C H 0 0 N N N N N N 32.106 -25.958 10.447 7.582 6.048 4.574 H6C2 PL3 29\nPL3 H7C1 1H7C H 0 0 N N N N N N 30.559 -26.771 11.909 8.527 3.130 4.650 H7C1 PL3 30\nPL3 H7C2 2H7C H 0 0 N N N N N N 29.700 -25.186 12.026 6.998 3.727 5.287 H7C2 PL3 31\nPL3 H8C1 1H8C H 0 0 N N N N N N 28.483 -25.582 10.023 7.820 3.847 2.347 H8C1 PL3 32\nPL3 H8C2 2H8C H 0 0 N N N N N N 29.542 -

26.931 9.537 6.333 4.494 3.032 H8C2 PL3 33\nPL3 H9C1 1H9C H 0 0 N N N N N N 28.840 -28.010 11.805 7.166 1.546 3.045 H9C1 PL3 34\nPL3 H9C2 2H9C H 0 0 N N N N N N 27.481 -26.786 11.743 5.719 2.164 3.822 H9C2 PL3 35\nPL3 HAC1 1HAC H 0 0 N N N N N N 26.333 -28.019 10.306 6.394 2.325 0.840 HAC1 PL3 36\nPL3 HAC2 2HAC H 0 0 N N N N N N 27.754 -28.227 9.236 4.935 2.975 1.555 HAC2 PL3 37\nPL3 HBC1 1HBC H 0 0 N N N N N N 28.396 -30.201 10.239 4.284 0.667 2.295 HBC1 PL3 38\nPL3 HBC2 2HBC H 0 0 N N N N N N 27.743 -29.713 11.848 5.690 0.018 1.495 HBC2 PL3 39\nPL3 HCC1 1HCC H 0 0 N N N N N N 25.425 -29.964 10.888 3.398 1.475 0.099 HCC1 PL3 40\nPL3 HCC2 2HCC H 0 0 N N N N N N 26.195 -30.675 9.411 4.851 0.906 -0.695 HCC2 PL3 41\nPL3 HDC1 1HDC H 0 0 N N N N N N 27.236 -31.924 11.808 4.242 -1.466 0.002 HDC1 PL3 42\nPL3 HDC2 2HDC H 0 0 N N N N N N 25.423 -32.007 11.763 2.768 -0.821 0.703 HDC2 PL3 43\nPL3 HEC1 1HEC H 0 0 N N N N N N 26.347 -32.602 9.132 3.528 -0.665 -2.262 HEC1 PL3 44\nPL3 HEC2 2HEC H 0 0 N N N N N N 27.362 -33.556 10.255 2.078 0.052 -1.554 HEC2 PL3 45\nPL3 HFC1 1HFC H 0 0 N N N N N N 25.383 -34.433 11.376 2.799 -2.929 -1.522 HFC1 PL3 46\nPL3 HFC2 2HFC H 0 0 N N N N N N 24.319 -33.372 10.356 1.358 -2.234 -0.777 HFC2 PL3 47\nPL3 HGC1 1HGC H 0 0 N N N N N N 25.150 -34.576 8.340 0.832 -3.169 -3.004 HGC1 PL3 48\nPL3 HGC2 2HGC H 0 0 N N N N N N 26.047 -35.696 9.420 0.592 -1.413 -3.014 HGC2 PL3 49\nPL3 HGC3 3HGC H 0 0 N N N N N N 24.253 -35.628 9.486 2.039 -2.116 -3.763 HGC3 PL3 50\n#\nloop_\n_chem_comp_bond.comp_id\n_chem_comp_bond.atom_id_1\n_chem_comp_bond.atom_id_2\n_chem_comp_bond.value_order\n_chem_comp_bond.pdbx_aromatic_flag\n_chem_comp_bond.pdbx_stereo_config\n_chem_comp_bond.pdbx_ordinal\nPL3 C1 C2 SING N N 2\nPL3 C2 C3 SING N N 3\nPL3 C3 C4 SING N N 4\nPL3 C4 C5 SING N N 5\nPL3 C5 C6 SING N N 6\nPL3 C6 C7 SING N N 7\nPL3 C7 C8 SING N N 8\nPL3 C8 C9 SING N N 9\nPL3 C9 CA SING N N 10\nPL3 CA CB SING N N 11\nPL3 CB CC SING N N 12\nPL3 CC CD SING N N 13\nPL3 CD CE SING N N 14\nPL3 CE CF SING N N 15\nPL3 CF CG SING N N 16\nPL3 C1 H1C1 SING N N 17\nPL3 C1 H1C2 SING N N 18\nPL3 C2 H2C1 SING N N 19\nPL3 C2 H2C2 SING N N 20\nPL3 C3 H3C1 SING N N 21\nPL3 C3 H3C2 SING N N 22\nPL3 C4 H4C1 SING N N 23\nPL3 C4 H4C2 SING N N 24\nPL3 C5 H5C1 SING N N 25\nPL3 C5 H5C2 SING N N 26\nPL3 C6 H6C1 SING N N 27\nPL3 C6 H6C2 SING N N 28\nPL3 C7 H7C1 SING N N 29\nPL3 C7 H7C2 SING N N 30\nPL3 C8 H8C1 SING N N 31\nPL3 C8 H8C2 SING N N 32\nPL3 C9 H9C1 SING N N 33\nPL3 C9 H9C2 SING N N 34\nPL3 CA HAC1 SING N N 35\nPL3 CA HAC2 SING N N 36\nPL3 CB HBC1 SING N N 37\nPL3 CB HBC2 SING N N 38\nPL3 CC HCC1 SING N N 39\nPL3 CC HCC2 SING N N 40\nPL3 CD HDC1 SING N N 41\nPL3 CD HDC2 SING N N 42\nPL3 CE HEC1 SING N N 43\nPL3 CE HEC2 SING N N 44\nPL3 CF HFC1 SING N N 45\nPL3 CF HFC2 SING N N 46\nPL3 CG HGC1 SING N N 47\nPL3 CG HGC2 SING N N 48\nPL3 CG HGC3 SING N N 49\n#\nloop_\n_pdbx_chem_comp_descriptor.comp_id\n_pdbx_chem_comp_descriptor.type\n_pdbx_chem_comp_descriptor.program\n_pdbx_chem_comp_descriptor.program_version\n_pdbx_chem_comp_descriptor.descriptor\nPL3 SMILES ACDLabs 10.04 OCCCCCCCCCCCCCCCC\nPL3 SMILES_CANONICAL CACTVS 3.341 CCCCCCCCCCCCCCCCO\nPL3 SMILES CACTVS 3.341 CCCCCCCCCCCCCCCCO\nPL3 SMILES_CANONICAL 'OpenEye OEToolkits' 1.5.0 CCCCCCCCCCCCCCCCO\nPL3 SMILES 'OpenEye OEToolkits' 1.5.0 CCCCCCCCCCCCCCCCO\nPL3 InChI InChI 1.03 InChI=1S/C16H34O/c1-2-3-4-5-6-7-8-9-10-11-12-13-14-15-16-17/h17H,2-16H2,1H3\nPL3 InChIKey InChI 1.03 BXWNKGSJHAJOGX-UHFFFAOYSA-

N\n#\nloop_\n_pdbx_chem_comp_identifier.comp_id\n_pdbx_chem_comp_identifier.type\n_pdbx_chem_comp_identifier.program\n_pdbx_chem_comp_identifier.program_version\n_pdbx_chem_comp_identifier.identifier\nPL3 'SYSTEMATIC NAME' ACDLabs 10.04 hexadecan-1-ol\nPL3 'SYSTEMATIC NAME' 'OpenEye OEToolkits' 1.5.0 hexadecan-1-ol\n#\nloop_\n_pdbx_chem_comp_audit.comp_id\n_pdbx_chem_comp_audit.action_type\n_pdbx_chem_comp_audit.date\n_pdbx_chem_comp_audit.processing_site\nPL3 'Create component' 2007-03-07 EBI\nPL3 'Modify descriptor' 2011-06-04 RCSB\n#\n_pdbe_chem_comp_drugbank_details.comp_id PL3\n_pdbe_chem_comp_drugbank_details.drugbank_id DB09494\n_pdbe_chem_comp_drugbank_details.type 'small molecule'\n_pdbe_chem_comp_drugbank_details.name 'Cetyl alcohol'\n_pdbe_chem_comp_drugbank_details.description\n'Cetyl alcohol, also known as 1-hexadecanol or n-hexadecyl alcohol, is a 16-C fatty alcohol with the chemical formula CH3(CH2)15OH. It can be produced from the reduction of palmitic acid. Cetyl alcohol is present in a waxy white powder or flake form at room temperature, and is insoluble in water and soluble in alcohols and oils [A32209]. Discovered by Chevrenl in 1913, cetyl alcohol is one of the oldest known long-chain alcohol [A32209]. It may be contained in cosmetic and personal care products such as shampoos, creams and lotions. Mainly it is used as an opacifier, emulsifier, and thickening agent that alter the thickness of the liquid, and increase and stabilize the foaming capacity. Due to its water-binding property, cetyl alcohol is commonly used as an emollient that prevents drying and chapping of the skin [A32209]. According to the FDA Code of Federal Regulations, cetyl alcohol is a safe synthetic fatty acid in food and in the synthesis of food components under the condition that it contain not less than 98 percent of total alcohols and not less than 94 percent of straight chain alcohols [L1824]. Cetyl alcohol is also listed in the OTC ingredient list as a skin protectant for skin irritations caused by poison ivy, oak, sumac, and insect bites or stings [L1074]. Cetyl alcohol is reported to be a mild skin or eye irritant.'\n_pdbe_chem_comp_drugbank_details.cas_number 36653-82-4\n_pdbe_chem_comp_drugbank_details.mechanism_of_action\n'Cetyl alcohol has hydrating properties that makes it a suitable emulsifier and stabilizer in pharmaceutical formulations. It is also present in washable ointment base due to its dispersant abilities and stabilizing properties [A32209]. Potential antimicrobial activity of cetyl alcohol may be due to a change in cell membrane permeability that either blocks absorption of essential nutrients and induction of outward diffusion vital cellular components [A32209]. This proposed mechanism of action is thought to be similar for other long-chain aliphatic alcohols with same antimicrobial activity, such as myristyl alcohol and behenyl alcohol [A32209].'\n#\nloop_\n_pdbe_chem_comp_synonyms.comp_id\n_pdbe_chem_comp_synonyms.name\n_pdbe_chem_comp_synonyms.provenance\n_pdbe_chem_comp_synonyms.type\nPL3 'Cetyl Alcohol' ChEMBL 'United States Accepted Names'\nPL3 hexadecan-1-ol DrugBank ?\nPL3 'palmityl alcohol' DrugBank ?\n#\n_pdbe_chem_comp_drugbank_classification.comp_id PL3\n_pdbe_chem_comp_drugbank_classification.drugbank_id DB09494\n_pdbe_chem_comp_drugbank_classification.parent 'Long-chain

fatty alcohols'\n_pdbe_chem_comp_drugbank_classification.kingdom
'Organic compounds'\n_pdbe_chem_comp_drugbank_classification.class
'Fatty Acyls'\n_pdbe_chem_comp_drugbank_classification.superclass
'Lipids and lipid-like
molecules'\n_pdbe_chem_comp_drugbank_classification.description\n'This
compound belongs to the class of organic compounds known as long-chain
fatty alcohols. These are fatty alcohols that have an aliphatic tail
of 13 to 21 carbon
atoms.'\n#\nloop_\n_software.name\n_software.version\n_software.descri
ption\nrdkit 2023.09.6 'Core functionality.'\npdbeccdutils 0.8.6
'Wrapper to provide 2D templates and molecular
fragments.'\n#\nloop_\n_pdbe_chem_comp_atom_depiction.comp_id\n_pdbe_c
hem_comp_atom_depiction.atom_id\n_pdbe_chem_comp_atom_depiction.elemen
t\n_pdbe_chem_comp_atom_depiction.model_Cartn_x\n_pdbe_chem_comp_atom_
depiction.model_Cartn_y\n_pdbe_chem_comp_atom_depiction.pdbx_ordinal\n
PL3 C1 C 5.104 0.375 1\nPL3 C2 C 6.404 -0.375 3\nPL3 C3 C 7.702 0.375
4\nPL3 C4 C 9.002 -0.375 5\nPL3 C5 C 10.301 0.375 6\nPL3 C6 C 11.600 -
0.375 7\nPL3 C7 C 12.899 0.375 8\nPL3 C8 C 14.198 -0.375 9\nPL3 C9 C
15.497 0.375 10\nPL3 CA C 16.796 -0.375 11\nPL3 CB C 18.095 0.375
12\nPL3 CC C 19.394 -0.375 13\nPL3 CD C 20.693 0.375 14\nPL3 CE C
21.992 -0.375 15\nPL3 CF C 23.291 0.375 16\nPL3 CG C 24.590 -0.375
17\n#\nloop_\n_pdbe_chem_comp_bond_depiction.comp_id\n_pdbe_chem_comp_
bond_depiction.atom_id_1\n_pdbe_chem_comp_bond_depiction.atom_id_2\n_p
dbe_chem_comp_bond_depiction.value_order\n_pdbe_chem_comp_bond_depicti
on.bond_dir\n_pdbe_chem_comp_bond_depiction.pdbx_ordinal\nPL3 C1 C2
SINGLE NONE 2\nPL3 C2 C3 SINGLE NONE 3\nPL3 C3 C4 SINGLE NONE 4\nPL3
C4 C5 SINGLE NONE 5\nPL3 C5 C6 SINGLE NONE 6\nPL3 C6 C7 SINGLE NONE
7\nPL3 C7 C8 SINGLE NONE 8\nPL3 C8 C9 SINGLE NONE 9\nPL3 C9 CA SINGLE
NONE 10\nPL3 CA CB SINGLE NONE 11\nPL3 CB CC SINGLE NONE 12\nPL3 CC CD
SINGLE NONE 13\nPL3 CD CE SINGLE NONE 14\nPL3 CE CF SINGLE NONE
15\nPL3 CF CG SINGLE NONE
16\n#\n#\n#\n_pdbe_chem_comp_rdkit_properties.comp_id
PL3\n_pdbe_chem_comp_rdkit_properties.exactmw
242.261\n_pdbe_chem_comp_rdkit_properties.amw
242.447\n_pdbe_chem_comp_rdkit_properties.lipinskiHBA
1\n_pdbe_chem_comp_rdkit_properties.lipinskiHBD
1\n_pdbe_chem_comp_rdkit_properties.NumRotatableBonds
16\n_pdbe_chem_comp_rdkit_properties.NumHBD
1\n_pdbe_chem_comp_rdkit_properties.NumHBA
1\n_pdbe_chem_comp_rdkit_properties.NumHeavyAtoms
17\n_pdbe_chem_comp_rdkit_properties.NumAtoms
51\n_pdbe_chem_comp_rdkit_properties.NumHeteroatoms
1\n_pdbe_chem_comp_rdkit_properties.NumAmideBonds
0\n_pdbe_chem_comp_rdkit_properties.FractionCSP3
1\n_pdbe_chem_comp_rdkit_properties.NumRings
0\n_pdbe_chem_comp_rdkit_properties.NumAromaticRings
0\n_pdbe_chem_comp_rdkit_properties.NumAliphaticRings
0\n_pdbe_chem_comp_rdkit_properties.NumSaturatedRings
0\n_pdbe_chem_comp_rdkit_properties.NumHeterocycles
0\n_pdbe_chem_comp_rdkit_properties.NumAromaticHeterocycles
0\n_pdbe_chem_comp_rdkit_properties.NumSaturatedHeterocycles
0\n_pdbe_chem_comp_rdkit_properties.NumAliphaticHeterocycles

0\n_pdbe_chem_comp_rdkit_properties.NumSpiroAtoms
0\n_pdbe_chem_comp_rdkit_properties.NumBridgeheadAtoms
0\n_pdbe_chem_comp_rdkit_properties.NumAtomStereoCenters
0\n_pdbe_chem_comp_rdkit_properties.NumUnspecifiedAtomStereoCenters
0\n_pdbe_chem_comp_rdkit_properties.labuteASA
156.863\n_pdbe_chem_comp_rdkit_properties.tpsa
20.230\n_pdbe_chem_comp_rdkit_properties.CrippenClogP
5.460\n_pdbe_chem_comp_rdkit_properties.CrippenMR
77.398\n_pdbe_chem_comp_rdkit_properties.chi0v
8.408\n_pdbe_chem_comp_rdkit_properties.chi1v
3.954\n_pdbe_chem_comp_rdkit_properties.chi2v
0.864\n_pdbe_chem_comp_rdkit_properties.chi3v
0.864\n_pdbe_chem_comp_rdkit_properties.chi4v
0.401\n_pdbe_chem_comp_rdkit_properties.chi0n
42.408\n_pdbe_chem_comp_rdkit_properties.chi1n
20.862\n_pdbe_chem_comp_rdkit_properties.chi2n
0.864\n_pdbe_chem_comp_rdkit_properties.chi3n
0.864\n_pdbe_chem_comp_rdkit_properties.chi4n
0.401\n_pdbe_chem_comp_rdkit_properties.hallKierAlpha -
0.040\n_pdbe_chem_comp_rdkit_properties.kappa1
1.731\n_pdbe_chem_comp_rdkit_properties.kappa2
15.960\n_pdbe_chem_comp_rdkit_properties.kappa3
15.960\n_pdbe_chem_comp_rdkit_properties.Phi
1.625\n#\nloop_\n_pdbe_chem_comp_external_mappings.comp_id\n_pdbe_chem_comp_external_mappings.source\n_pdbe_chem_comp_external_mappings.resource\n_pdbe_chem_comp_external_mappings.resource_id\nPL3 UniChem ChEMBL CHEMBL706\nPL3 UniChem DrugBank DB09494\nPL3 UniChem ChEBI 16125\nPL3 UniChem ZINC ZINC000008214519\nPL3 UniChem eMolecules 475733\nPL3 UniChem fdasrs 936JST6JCN\nPL3 UniChem SureChEMBL SCHEMBL3381\nPL3 UniChem HMDB HMDB0003424\nPL3 UniChem Selleck 1-hexadecanol\nPL3 UniChem NMRShiftDB 10008807\nPL3 UniChem ACTor 124-29-8\nPL3 UniChem DrugCentral 4750\nPL3 UniChem MetaboLights MTBLC16125\nPL3 UniChem BRENDA 12594\nPL3 UniChem BRENDA 12851\nPL3 UniChem BRENDA 12872\nPL3 UniChem BRENDA 1379\nPL3 UniChem BRENDA 149508\nPL3 UniChem BRENDA 211565\nPL3 UniChem BRENDA 49268\nPL3 UniChem BRENDA 5455\nPL3 UniChem Rhea 16125\nPL3 UniChem ChemicalBook CB4853561\nPL3 UniChem DailyMed 'CETYL ALCOHOL'\nPL3 UniChem ClinicalTrials CETANOL\nPL3 UniChem ClinicalTrials 'CETYL ALCOHOL'\nPL3 UniChem rxnorm 'CETYL ALCOHOL'\nPL3 UniChem MedChemExpress HY-B1465\nPL3 UniChem 'Probes And Drugs' PD010418\nPL3 UniChem CCDC HEXDEC\nPL3 UniChem SwissLipids SLM:000000202\nPL3 UniChem 'PubChem TPHARMA' 15189029\nPL3 UniChem PubChem 2682\nPL3 UniChem Mcule MCULE-9457426256\nPL3 UniChem ACTor 36653-82-4\nPL3 UniChem ACTor 67762-27-0\nPL3 UniChem Nikkaji J3.499A\nPL3 UniChem 'EPA CompTox Dashboard' DTXSID4027991\n#\nloop_\n_pdbe_chem_comp_rdkit_conformer.comp_id\n_pdbe_chem_comp_rdkit_conformer.atom_id\n_pdbe_chem_comp_rdkit_conformer.Cartn_x_rdkit\n_pdbe_chem_comp_rdkit_conformer.Cartn_y_rdkit\n_pdbe_chem_comp_rdkit_conformer.Cartn_z_rdkit\n_pdbe_chem_comp_rdkit_conformer.rdkit_method\n_pdbe_chem_comp_rdkit_conformer.rdkit_ordinal\nPL3 C1 6.628 -0.271 -2.212 ETKDGv3 1\nPL3 C2 6.001 -0.012 -0.839 ETKDGv3 3\nPL3 C3 4.693 -0.800 -0.673 ETKDGv3 4\nPL3 C4 4.102 -0.674 0.740

ETKDGv3 5\nPL3 C5 3.565 0.737 1.035 ETKDGv3 6\nPL3 C6 2.781 0.796 2.364 ETKDGv3 7\nPL3 C7 1.255 0.810 2.177 ETKDGv3 8\nPL3 C8 0.713 -0.511 1.605 ETKDGv3 9\nPL3 C9 -0.821 -0.600 1.668 ETKDGv3 10\nPL3 CA -1.509 0.363 0.685 ETKDGv3 11\nPL3 CB -3.040 0.222 0.697 ETKDGv3 12\nPL3 CC -3.526 -0.989 -0.119 ETKDGv3 13\nPL3 CD -5.044 -1.219 -0.003 ETKDGv3 14\nPL3 CE -5.921 -0.069 -0.530 ETKDGv3 15\nPL3 CF -5.712 0.206 -2.026 ETKDGv3 16\nPL3 CG -6.729 1.219 -2.546 ETKDGv3 17\nPL3 H1C1 6.850 -1.354 -2.321 ETKDGv3 18\nPL3 H1C2 5.921 0.020 -3.022 ETKDGv3 19\nPL3 H2C1 6.723 -0.314 -0.049 ETKDGv3 20\nPL3 H2C2 5.801 1.076 -0.740 ETKDGv3 21\nPL3 H3C1 3.948 -0.459 -1.424 ETKDGv3 22\nPL3 H3C2 4.895 -1.877 -0.865 ETKDGv3 23\nPL3 H4C1 4.871 -0.948 1.495 ETKDGv3 24\nPL3 H4C2 3.278 -1.413 0.829 ETKDGv3 25\nPL3 H5C1 2.933 1.093 0.192 ETKDGv3 26\nPL3 H5C2 4.429 1.432 1.113 ETKDGv3 27\nPL3 H6C1 3.057 1.739 2.887 ETKDGv3 28\nPL3 H6C2 3.075 -0.034 3.043 ETKDGv3 29\nPL3 H7C1 0.790 0.993 3.170 ETKDGv3 30\nPL3 H7C2 0.978 1.657 1.513 ETKDGv3 31\nPL3 H8C1 1.128 -1.356 2.195 ETKDGv3 32\nPL3 H8C2 1.043 -0.633 0.551 ETKDGv3 33\nPL3 H9C1 -1.104 -1.645 1.424 ETKDGv3 34\nPL3 H9C2 -1.166 -0.393 2.705 ETKDGv3 35\nPL3 HAC1 -1.122 0.202 -0.345 ETKDGv3 36\nPL3 HAC2 -1.266 1.407 0.976 ETKDGv3 37\nPL3 HBC1 -3.407 0.152 1.745 ETKDGv3 38\nPL3 HBC2 -3.463 1.146 0.251 ETKDGv3 39\nPL3 HCC1 -3.235 -0.870 -1.184 ETKDGv3 40\nPL3 HCC2 -3.027 -1.909 0.249 ETKDGv3 41\nPL3 HDC1 -5.299 -1.397 1.065 ETKDGv3 42\nPL3 HDC2 -5.303 -2.148 -0.556 ETKDGv3 43\nPL3 HEC1 -5.739 0.856 0.057 ETKDGv3 44\nPL3 HEC2 -6.983 -0.354 -0.362 ETKDGv3 45\nPL3 HFC1 -5.819 -0.738 -2.602 ETKDGv3 46\nPL3 HFC2 -4.695 0.616 -2.198 ETKDGv3 47\nPL3 HGC1 -6.546 1.410 -3.624 ETKDGv3 48\nPL3 HGC2 -7.761 0.827 -2.426 ETKDGv3 49\nPL3 HGC3 -6.634 2.178 -1.994 ETKDGv3 50\n#\ndata_ETA-2\n#\n_chem_comp.id ETA\n_chem_comp.name ETHANOLAMINE\n_chem_comp.type 'L-peptide COOH carboxy terminus'\n_chem_comp.pdbx_type ATOMP\n_chem_comp.formula 'C2 H7 N O'\n_chem_comp.mon_nstd_parent_comp_id ?\n_chem_comp.pdbx_synonyms ?\n_chem_comp.pdbx_formal_charge 0\n_chem_comp.pdbx_initial_date 1999-07-08\n_chem_comp.pdbx_modified_date 2024-09-27\n_chem_comp.pdbx_ambiguous_flag N\n_chem_comp.pdbx_release_status REL\n_chem_comp.pdbx_replaced_by ?\n_chem_comp.pdbx_replaces ?\n_chem_comp.formula_weight 61.083\n_chem_comp.one_letter_code ?\n_chem_comp.three_letter_code ETA\n_chem_comp.pdbx_model_coordinates_details ?\n_chem_comp.pdbx_model_coordinates_missing_flag N\n_chem_comp.pdbx_ideal_coordinates_details Corina\n_chem_comp.pdbx_ideal_coordinates_missing_flag N\n_chem_comp.pdbx_model_coordinates_db_code ?\n_chem_comp.pdbx_subcomponent_list ?\n_chem_comp.pdbx_processing_site RCSB\n_chem_comp.pdbx_pcm Y\n#\nloop_\n_chem_comp_atom.comp_id\n_chem_comp_atom.atom_id\n_chem_comp_atom.alt_atom_id\n_chem_comp_atom.type_symbol\n_chem_comp_atom.charge\n_chem_comp_atom.pdbx_align\n_chem_comp_atom.pdbx_aromatic_flag\n_chem_comp_atom.pdbx_leaving_atom_flag\n_chem_comp_atom.pdbx_stereo_config\n_chem_comp_atom.pdbx_backbone_atom_flag\n_chem_comp_atom.pdbx_n_terminal_atom_flag\n_chem_comp_atom.pdbx_c_terminal_atom_flag\n_chem_comp_atom.model_Cartn_x\n_chem_comp_atom.model_Cartn_y\n_chem_comp_atom.model_Cartn_z\n_chem_comp_atom.pdbx_model_Cartn_x_ideal\n_chem_comp_at

om.pdbx_model_Cartn_y_ideal\n_chem_comp_atom.pdbx_model_Cartn_z_ideal\n_chem_comp_atom.pdbx_component_atom_id\n_chem_comp_atom.pdbx_component_comp_id\n_chem_comp_atom.pdbx_ordinal\nETA CA CA C 0 1 N N N N N N -12.690 2.113 24.488 -0.711 0.612 -0.308 CA ETA 1\nETA N N N 0 1 N N N Y Y N -12.852 1.252 23.311 -1.468 -0.574 0.114 N ETA 2\nETA C CB C 0 1 N N N N N N -13.061 3.561 24.312 0.681 0.581 0.327 CB ETA 3\nETA HA1 HA1 H 0 1 N N N N N N -13.334 1.705 25.281 -0.615 0.615 -1.394 HA1 ETA 5\nETA HA2 HA2 H 0 1 N N N N N N -11.628 2.081 24.772 -1.236 1.511 0.013 HA2 ETA 6\nETA H HN1 H 0 1 N N N Y Y N -12.578 0.318 23.540 -1.515 -0.634 1.120 HN1 ETA 7\nETA H2 HN2 H 0 1 N Y N Y Y N -13.810 1.257 23.024 -2.392 -0.577 -0.293 HN2 ETA 8\nETA HB1 HB1 H 0 1 N N N N N N -12.470 4.203 24.982 1.211 1.502 0.086 HB1 ETA 9\nETA HB2 HB2 H 0 1 N N N N N N -12.879 3.891 23.278 0.585 0.488 1.408 HB2 ETA 10\n#\nloop_\n_chem_comp_bond.comp_id\n_chem_comp_bond.atom_id_1\n_chem_comp_bond.atom_id_2\n_chem_comp_bond.value_order\n_chem_comp_bond.pdbx_aromatic_flag\n_chem_comp_bond.pdbx_stereo_config\n_chem_comp_bond.pdbx_ordinal\nETA CA N SING N N 1\nETA CA C SING N N 2\nETA CA HA1 SING N N 3\nETA CA HA2 SING N N 4\nETA N H SING N N 5\nETA N H2 SING N N 6\nETA C HB1 SING N N 8\nETA C HB2 SING N N 9\n#\nloop_\n_pdbx_chem_comp_descriptor.comp_id\n_pdbx_chem_comp_descriptor.type\n_pdbx_chem_comp_descriptor.program\n_pdbx_chem_comp_descriptor.program_version\n_pdbx_chem_comp_descriptor.descriptor\nETA SMILES ACDLabs 12.01 OCCN\nETA SMILES_CANONICAL CACTVS 3.370 NCCO\nETA SMILES CACTVS 3.370 NCCO\nETA SMILES_CANONICAL 'OpenEye OEToolkits' 1.7.0 'C(CO)N'\nETA SMILES 'OpenEye OEToolkits' 1.7.0 'C(CO)N'\nETA InChI InChI 1.03 InChI=1S/C2H7NO/c3-1-2-4/h4H,1-3H2\nETA InChIKey InChI 1.03 HZAXFHJVJLSVMW-UHFFFAOYSA-N\n#\nloop_\n_pdbx_chem_comp_identifier.comp_id\n_pdbx_chem_comp_identifier.type\n_pdbx_chem_comp_identifier.program\n_pdbx_chem_comp_identifier.program_version\n_pdbx_chem_comp_identifier.identifier\nETA 'SYSTEMATIC NAME' ACDLabs 12.01 2-aminoethanol\nETA 'SYSTEMATIC NAME' 'OpenEye OEToolkits' 1.7.0 2-azanylethanol\n#\nloop_\n_pdbx_chem_comp_audit.comp_id\n_pdbx_chem_comp_audit.action_type\n_pdbx_chem_comp_audit.date\n_pdbx_chem_comp_audit.processing_site\nETA 'Create component' 1999-07-08 RCSB\nETA 'Modify descriptor' 2011-06-04 RCSB\nETA 'Modify backbone' 2023-11-03 PDBE\nETA 'Modify PCM' 2024-09-27 PDBE\n#\n_pdbx_chem_comp_pcm.pcm_id 1\n_pdbx_chem_comp_pcm.comp_id ETA\n_pdbx_chem_comp_pcm.modified_residue_id ?\n_pdbx_chem_comp_pcm.type None\n_pdbx_chem_comp_pcm.category 'Non-standard residue'\n_pdbx_chem_comp_pcm.position 'Amino-acid side chain and backbone'\n_pdbx_chem_comp_pcm.polypeptide_position C-terminal\n_pdbx_chem_comp_pcm.comp_id_linking_atom ?\n_pdbx_chem_comp_pcm.modified_residue_id_linking_atom ?\n_pdbx_chem_comp_pcm.uniprot_specific_ptm_accession ?\n_pdbx_chem_comp_pcm.uniprot_generic_ptm_accession ?\n#\n_pdbe_chem_comp_drugbank_details.comp_id ETA\n_pdbe_chem_comp_drugbank_details.drugbank_id DB03994\n_pdbe_chem_comp_drugbank_details.type 'small molecule'\n_pdbe_chem_comp_drugbank_details.name Ethanolamine\n_pdbe_chem_comp_drugbank_details.description\n'A viscous, hygroscopic amino alcohol with an ammoniacal odor. It is widely distributed in biological tissue and is a component of

lecithin. It is used as a surfactant, fluorimetric reagent, and to remove CO2 and H2S from natural gas and other gases.'\n_pdbe_chem_comp_drugbank_details.cas_number 141-43-5\n_pdbe_chem_comp_drugbank_details.mechanism_of_action ?\n#\n_pdbe_chem_comp_drugbank_classification.comp_id ETA\n_pdbe_chem_comp_drugbank_classification.drugbank_id DB03994\n_pdbe_chem_comp_drugbank_classification.parent 1,2-aminoalcohols\n_pdbe_chem_comp_drugbank_classification.kingdom 'Organic compounds'\n_pdbe_chem_comp_drugbank_classification.class 'Organonitrogen compounds'\n_pdbe_chem_comp_drugbank_classification.superclass 'Organic nitrogen compounds'\n_pdbe_chem_comp_drugbank_classification.description\n'This compound belongs to the class of organic compounds known as 1,2-aminoalcohols. These are organic compounds containing an alkyl chain with an amine group bound to the C1 atom and an alcohol group bound to the C2 atom.'\n#\nloop_\n_pdbe_chem_comp_drugbank_targets.comp_id\n_pdbe_chem_comp_drugbank_targets.drugbank_id\n_pdbe_chem_comp_drugbank_targets.name\n_pdbe_chem_comp_drugbank_targets.organism\n_pdbe_chem_comp_drugbank_targets.uniprot_id\n_pdbe_chem_comp_drugbank_targets.pharmacologically_active\n_pdbe_chem_comp_drugbank_targets.ordinal\nETA DB03994 'Surface protein A' 'Neisseria meningitidis' Q9RP17 unknown 1\nETA DB03994 'Annexin A3' Humans P12429 unknown 2\n#\nloop_\n_software.name\n_software.version\n_software.description\nrdkit 2023.09.6 'Core functionality.'\npdbeccdutils 0.8.6 'Wrapper to provide 2D templates and molecular fragments.'\n#\nloop_\n_pdbe_chem_comp_atom_depiction.comp_id\n_pdbe_chem_comp_atom_depiction.atom_id\n_pdbe_chem_comp_atom_depiction.element\n_pdbe_chem_comp_atom_depiction.model_Cartn_x\n_pdbe_chem_comp_atom_depiction.model_Cartn_y\n_pdbe_chem_comp_atom_depiction.pdbx_ordinal\nETA CA C 6.404 -0.090 1\nETA N N 7.702 -0.840 2\nETA C C 5.104 -0.840 3\n#\nloop_\n_pdbe_chem_comp_bond_depiction.comp_id\n_pdbe_chem_comp_bond_depiction.atom_id_1\n_pdbe_chem_comp_bond_depiction.atom_id_2\n_pdbe_chem_comp_bond_depiction.value_order\n_pdbe_chem_comp_bond_depiction.bond_dir\n_pdbe_chem_comp_bond_depiction.pdbx_ordinal\nETA CA N SINGLE NONE 1\nETA CA C SINGLE NONE 2\n#\n#\n#\n_pdbe_chem_comp_rdkit_properties.comp_id ETA\n_pdbe_chem_comp_rdkit_properties.exactmw 61.053\n_pdbe_chem_comp_rdkit_properties.amw 61.084\n_pdbe_chem_comp_rdkit_properties.lipinskiHBA 2\n_pdbe_chem_comp_rdkit_properties.lipinskiHBD 3\n_pdbe_chem_comp_rdkit_properties.NumRotatableBonds 3\n_pdbe_chem_comp_rdkit_properties.NumHBD 2\n_pdbe_chem_comp_rdkit_properties.NumHBA 2\n_pdbe_chem_comp_rdkit_properties.NumHeavyAtoms 4\n_pdbe_chem_comp_rdkit_properties.NumAtoms 11\n_pdbe_chem_comp_rdkit_properties.NumHeteroatoms 2\n_pdbe_chem_comp_rdkit_properties.NumAmideBonds 0\n_pdbe_chem_comp_rdkit_properties.FractionCSP3 1\n_pdbe_chem_comp_rdkit_properties.NumRings 0\n_pdbe_chem_comp_rdkit_properties.NumAromaticRings

0\n_pdbe_chem_comp_rdkit_properties.NumAliphaticRings
0\n_pdbe_chem_comp_rdkit_properties.NumSaturatedRings
0\n_pdbe_chem_comp_rdkit_properties.NumHeterocycles
0\n_pdbe_chem_comp_rdkit_properties.NumAromaticHeterocycles
0\n_pdbe_chem_comp_rdkit_properties.NumSaturatedHeterocycles
0\n_pdbe_chem_comp_rdkit_properties.NumAliphaticHeterocycles
0\n_pdbe_chem_comp_rdkit_properties.NumSpiroAtoms
0\n_pdbe_chem_comp_rdkit_properties.NumBridgeheadAtoms
0\n_pdbe_chem_comp_rdkit_properties.NumAtomStereoCenters
0\n_pdbe_chem_comp_rdkit_properties.NumUnspecifiedAtomStereoCenters
0\n_pdbe_chem_comp_rdkit_properties.labuteASA
35.276\n_pdbe_chem_comp_rdkit_properties.tpsa
46.250\n_pdbe_chem_comp_rdkit_properties.CrippenClogP -
1.063\n_pdbe_chem_comp_rdkit_properties.CrippenMR
16.140\n_pdbe_chem_comp_rdkit_properties.chi0v
1.855\n_pdbe_chem_comp_rdkit_properties.chi1v
0.678\n_pdbe_chem_comp_rdkit_properties.chi2v
0.046\n_pdbe_chem_comp_rdkit_properties.chi3v
0.046\n_pdbe_chem_comp_rdkit_properties.chi4v
0\n_pdbe_chem_comp_rdkit_properties.chi0n
8.855\n_pdbe_chem_comp_rdkit_properties.chi1n
3.980\n_pdbe_chem_comp_rdkit_properties.chi2n
0.046\n_pdbe_chem_comp_rdkit_properties.chi3n
0.046\n_pdbe_chem_comp_rdkit_properties.chi4n
0\n_pdbe_chem_comp_rdkit_properties.hallKierAlpha -
0.080\n_pdbe_chem_comp_rdkit_properties.kappa1
0.340\n_pdbe_chem_comp_rdkit_properties.kappa2
2.920\n_pdbe_chem_comp_rdkit_properties.kappa3
1.920\n_pdbe_chem_comp_rdkit_properties.Phi
0.248\n#\nloop_\n_pdbe_chem_comp_external_mappings.comp_id\n_pdbe_chem
_comp_external_mappings.source\n_pdbe_chem_comp_external_mappings.reso
urce\n_pdbe_chem_comp_external_mappings.resource_id\nETA UniChem
ChEMBL CHEMBL104943\nETA UniChem DrugBank DB03994\nETA UniChem 'KEGG
LIGAND' C00189\nETA UniChem ChEBI 16000\nETA UniChem ZINC
ZINC000008214617\nETA UniChem eMolecules 474362\nETA UniChem fdasrs
5KV86114PT\nETA UniChem SureChEMBL SCHEMBL1644\nETA UniChem HMDB
HMDB0000149\nETA UniChem NMRShiftDB 10008325\nETA UniChem ACTor 141-
43-5\nETA UniChem BindingDb 7973\nETA UniChem MetaboLights
MTBLC16000\nETA UniChem BRENDA 15628\nETA UniChem BRENDA 20384\nETA
UniChem BRENDA 456\nETA UniChem BRENDA 9009\nETA UniChem ChemicalBook
CB1218589\nETA UniChem DailyMed MONOETHANOLAMINE\nETA UniChem
ClinicalTrials ETHANOLAMINE\nETA UniChem ClinicalTrials
MONOETHANOLAMINE\nETA UniChem ClinicalTrials OLAMINE\nETA UniChem
rxnorm ETHANOLAMINE\nETA UniChem 'Probes And Drugs' PD006576\nETA
UniChem CCDC JAKKEL\nETA UniChem 'PubChem TPHARMA' 15218601\nETA
UniChem PubChem 700\nETA UniChem Mcule MCULE-5175116598\nETA UniChem
Nikkaji J2.536D\nETA UniChem 'EPA CompTox Dashboard'
DTXSID6022000\n#\nloop_\n_pdbe_chem_comp_rdkit_conformer.comp_id\n_pdb
e_chem_comp_rdkit_conformer.atom_id\n_pdbe_chem_comp_rdkit_conformer.C
artn_x_rdkit\n_pdbe_chem_comp_rdkit_conformer.Cartn_y_rdkit\n_pdbe_che
m_comp_rdkit_conformer.Cartn_z_rdkit\n_pdbe_chem_comp_rdkit_conformer.
rdkit_method\n_pdbe_chem_comp_rdkit_conformer.rdkit_ordinal\nETA CA

0.398 0.195 0.370 ETKDGv3 1\nETA N 1.488 0.291 -0.596 ETKDGv3 2\nETA C -0.867 -0.327 -0.310 ETKDGv3 3\nETA HA1 0.195 1.204 0.793 ETKDGv3 5\nETA HA2 0.687 -0.485 1.202 ETKDGv3 6\nETA H 1.780 -0.679 -0.857 ETKDGv3 7\nETA H2 2.306 0.726 -0.110 ETKDGv3 8\nETA HB1 -0.670 -1.337 -0.730 ETKDGv3 9\nETA HB2 -1.157 0.344 -1.150 ETKDGv3 10\n#\ndata_GOL-2\n#\n_chem_comp.id GOL\n_chem_comp.name GLYCEROL\n_chem_comp.type NON-POLYMER\n_chem_comp.pdbx_type HETAIN\n_chem_comp.formula 'C3 H8 O3'\n_chem_comp.mon_nstd_parent_comp_id ?\n_chem_comp.pdbx_synonyms 'GLYCERIN; PROPANE-1,2,3-TRIOL'\n_chem_comp.pdbx_formal_charge 0\n_chem_comp.pdbx_initial_date 1999-07-08\n_chem_comp.pdbx_modified_date 2024-09-27\n_chem_comp.pdbx_ambiguous_flag N\n_chem_comp.pdbx_release_status REL\n_chem_comp.pdbx_replaced_by ?\n_chem_comp.pdbx_replaces CRY\n_chem_comp.formula_weight 92.094\n_chem_comp.one_letter_code ?\n_chem_comp.three_letter_code GOL\n_chem_comp.pdbx_model_coordinates_details ?\n_chem_comp.pdbx_model_coordinates_missing_flag N\n_chem_comp.pdbx_ideal_coordinates_details Corina\n_chem_comp.pdbx_ideal_coordinates_missing_flag N\n_chem_comp.pdbx_model_coordinates_db_code 1BXO\n_chem_comp.pdbx_subcomponent_list ?\n_chem_comp.pdbx_processing_site EBI\n_chem_comp.pdbx_pcm Y\n#\nloop_\n_chem_comp_atom.comp_id\n_chem_comp_atom.atom_id\n_chem_comp_atom.alt_atom_id\n_chem_comp_atom.type_symbol\n_chem_comp_atom.charge\n_chem_comp_atom.pdbx_align\n_chem_comp_atom.pdbx_aromatic_flag\n_chem_comp_atom.pdbx_leaving_atom_flag\n_chem_comp_atom.pdbx_stereo_config\n_chem_comp_atom.pdbx_backbone_atom_flag\n_chem_comp_atom.pdbx_n_terminal_atom_flag\n_chem_comp_atom.pdbx_c_terminal_atom_flag\n_chem_comp_atom.model_Cartn_x\n_chem_comp_atom.model_Cartn_y\n_chem_comp_atom.model_Cartn_z\n_chem_comp_atom.pdbx_model_Cartn_x_ideal\n_chem_comp_atom.pdbx_model_Cartn_y_ideal\n_chem_comp_atom.pdbx_model_Cartn_z_ideal\n_chem_comp_atom.pdbx_component_atom_id\n_chem_comp_atom.pdbx_component_comp_id\n_chem_comp_atom.pdbx_ordinal\nGOL C1 C1 C 0 1 N N N N N N 29.490 2.376 31.160 -1.249 -0.665 0.295 C1 GOL 1\nGOL O1 O1 O 0 1 N N N N N N 28.524 1.609 30.767 -2.413 -0.087 -0.300 O1 GOL 2\nGOL C2 C2 C 0 1 N N N N N N 30.249 2.850 29.893 0.000 0.034 -0.245 C2 GOL 3\nGOL O2 O2 O 0 1 N N N N N N 29.526 4.021 29.804 0.000 1.400 0.174 O2 GOL 4\nGOL C3 C3 C 0 1 N N N N N N 31.780 2.780 29.785 1.249 -0.665 0.295 C3 GOL 5\nGOL H11 H11 H 0 1 N N N N N N 29.089 3.241 31.708 -1.295 -0.543 1.377 H11 GOL 7\nGOL H12 H12 H 0 1 N N N N N N 30.169 1.821 31.824 -1.206 -1.727 0.051 H12 GOL 8\nGOL HO1 HO1 H 0 1 N N N N N N 28.037 1.300 31.522 -3.244 -0.483 -0.003 HO1 GOL 9\nGOL H2 H2 H 0 1 N N N N N N 30.271 2.201 29.005 -0.000 -0.012 -1.334 H2 GOL 10\nGOL HO2 HO2 H 0 1 N N N N N N 29.825 4.522 29.054 0.000 1.517 1.134 HO2 GOL 11\nGOL H31 H31 H 0 1 N N N N N N 32.222 2.438 30.733 1.295 -0.543 1.377 H31 GOL 12\nGOL H32 H32 H 0 1 N N N N N N 32.083 2.080 28.993 1.206 -1.727 0.051 H32 GOL 13\n#\nloop_\n_chem_comp_bond.comp_id\n_chem_comp_bond.atom_id_1\n_chem_comp_bond.atom_id_2\n_chem_comp_bond.value_order\n_chem_comp_bond.pdbx_aromatic_flag\n_chem_comp_bond.pdbx_stereo_config\n_chem_comp_bond.pdbx_ordinal\nGOL C1 O1 SING N N 1\nGOL C1 C2 SING N N 2\nGOL C1 H11 SING N N 3\nGOL C1 H12 SING N N 4\nGOL O1 HO1 SING N N 5\nGOL C2 O2

SING N N 6\nGOL C2 C3 SING N N 7\nGOL C2 H2 SING N N 8\nGOL O2 HO2 SING N N 9\nGOL C3 H31 SING N N 11\nGOL C3 H32 SING N N 12\n#\nloop_\n_pdbx_chem_comp_descriptor.comp_id\n_pdbx_chem_comp_descriptor.type\n_pdbx_chem_comp_descriptor.program\n_pdbx_chem_comp_descriptor.program_version\n_pdbx_chem_comp_descriptor.descriptor\nGOL SMILES ACDLabs 12.01 'OCC(O)CO'\nGOL SMILES_CANONICAL CACTVS 3.370 'OCC(O)CO'\nGOL SMILES CACTVS 3.370 'OCC(O)CO'\nGOL SMILES_CANONICAL 'OpenEye OEToolkits' 1.7.0 'C(C(CO)O)O'\nGOL SMILES 'OpenEye OEToolkits' 1.7.0 'C(C(CO)O)O'\nGOL InChI InChI 1.03 'InChI=1S/C3H8O3/c4-1-3(6)2-5/h3-6H,1-2H2'\nGOL InChIKey InChI 1.03 PEDCQBHIVMGVHV-UHFFFAOYSA-N\n#\nloop_\n_pdbx_chem_comp_identifier.comp_id\n_pdbx_chem_comp_identifier.type\n_pdbx_chem_comp_identifier.program\n_pdbx_chem_comp_identifier.program_version\n_pdbx_chem_comp_identifier.identifier\nGOL 'SYSTEMATIC NAME' ACDLabs 12.01 propane-1,2,3-triol\nGOL 'SYSTEMATIC NAME' 'OpenEye OEToolkits' 1.7.0 propane-1,2,3-triol\n#\nloop_\n_pdbx_chem_comp_audit.comp_id\n_pdbx_chem_comp_audit.action_type\n_pdbx_chem_comp_audit.date\n_pdbx_chem_comp_audit.processing_site\nGOL 'Create component' 1999-07-08 EBI\nGOL 'Modify descriptor' 2011-06-04 RCSB\nGOL 'Modify synonyms' 2020-05-28 PDBE\nGOL 'Modify PCM' 2024-09-27 PDBE\n#\nloop_\n_pdbx_chem_comp_synonyms.ordinal\n_pdbx_chem_comp_synonyms.comp_id\n_pdbx_chem_comp_synonyms.name\n_pdbx_chem_comp_synonyms.provenance\n_pdbx_chem_comp_synonyms.type\n1 GOL GLYCERIN ? ?\n2 GOL PROPANE-1,2,3-TRIOL ? ?\n#\nloop_\n_pdbx_chem_comp_pcm.pcm_id\n_pdbx_chem_comp_pcm.comp_id\n_pdbx_chem_comp_pcm.modified_residue_id\n_pdbx_chem_comp_pcm.type\n_pdbx_chem_comp_pcm.category\n_pdbx_chem_comp_pcm.position\n_pdbx_chem_comp_pcm.polypeptide_position\n_pdbx_chem_comp_pcm.comp_id_linking_atom\n_pdbx_chem_comp_pcm.modified_residue_id_linking_atom\n_pdbx_chem_comp_pcm.uniprot_specific_ptm_accession\n_pdbx_chem_comp_pcm.uniprot_generic_ptm_accession\n1 GOL ASP None 'Covalent chemical modification' 'Amino-acid side chain' 'Any position' C1 OD1 ? ?\n2 GOL CYS None 'Covalent chemical modification' 'Amino-acid side chain' 'Any position' C1 SG ? ?\n3 GOL GLU None 'Covalent chemical modification' 'Amino-acid side chain' 'Any position' C1 OE1 ? ?\n4 GOL HIS None 'Covalent chemical modification' 'Amino-acid side chain' 'Any position' C1 NE2 ? ?\n5 GOL HIS None 'Covalent chemical modification' 'Amino-acid side chain' 'Any position' C2 NE2 ? ?\n6 GOL LYS None 'Covalent chemical modification' 'Amino-acid side chain' 'Any position' C1 NZ ? ?\n#\n_pdbe_chem_comp_drugbank_details.comp_id GOL\n_pdbe_chem_comp_drugbank_details.drugbank_id DB09462\n_pdbe_chem_comp_drugbank_details.type 'small molecule'\n_pdbe_chem_comp_drugbank_details.name Glycerin\n_pdbe_chem_comp_drugbank_details.description\n'A trihydroxy sugar alcohol that is an intermediate in carbohydrate and lipid metabolism.'\n_pdbe_chem_comp_drugbank_details.cas_number 56-81-5\n_pdbe_chem_comp_drugbank_details.mechanism_of_action\n'When administered rectally, glycerin exerts a hygroscopic and/or local irritant action, drawing water from the tissues into the feces and reflexively stimulating evacuation. Glycerin decreases intraocular pressure by creating an osmotic gradient between the blood and

intraocular fluid, causing fluid to move out of the aqueous and vitreous humors into the bloodstream.'\n#\nloop_\n_pdbe_chem_comp_synonyms.comp_id\n_pdbe_chem_comp_synonyms.name\n_pdbe_chem_comp_synonyms.provenance\n_pdbe_chem_comp_synonyms.type\nGOL Glycerin ChEMBL 'United States Accepted Names'\nGOL Glycerol ChEMBL Tradename\nGOL 'Monoctanoin Component D' ChEMBL 'United States Accepted Names'\nGOL Optim ChEMBL Tradename\nGOL Osmoglyn ChEMBL Tradename\nGOL GLYCERIN wwPDB ?\nGOL PROPANE-1,2,3-TRIOL wwPDB ?\nGOL 1,2,3-propanetriol DrugBank ?\nGOL 1,2,3-trihydroxypropane DrugBank ?\nGOL Glycerin DrugBank ?\nGOL 'Glycerin, anhydrous' DrugBank ?\nGOL Glycerin,anhydrous DrugBank ?\nGOL Glycerine DrugBank ?\nGOL Glycerinum DrugBank ?\nGOL Glycerol DrugBank ?\nGOL 'Monoctanoin component D' DrugBank ?\nGOL Propanetriol DrugBank ?\nGOL Trihydroxypropane DrugBank ?\n#\n_pdbe_chem_comp_drugbank_classification.comp_id GOL\n_pdbe_chem_comp_drugbank_classification.drugbank_id DB09462\n_pdbe_chem_comp_drugbank_classification.parent 'Sugar alcohols'\n_pdbe_chem_comp_drugbank_classification.kingdom 'Organic compounds'\n_pdbe_chem_comp_drugbank_classification.class 'Organooxygen compounds'\n_pdbe_chem_comp_drugbank_classification.superclass 'Organic oxygen compounds'\n_pdbe_chem_comp_drugbank_classification.description\n'This compound belongs to the class of organic compounds known as sugar alcohols. These are hydrogenated forms of carbohydrate in which the carbonyl group (aldehyde or ketone, reducing sugar) has been reduced to a primary or secondary hydroxyl group.'\n#\nloop_\n_pdbe_chem_comp_drugbank_targets.comp_id\n_pdbe_chem_comp_drugbank_targets.drugbank_id\n_pdbe_chem_comp_drugbank_targets.name\n_pdbe_chem_comp_drugbank_targets.organism\n_pdbe_chem_comp_drugbank_targets.uniprot_id\n_pdbe_chem_comp_drugbank_targets.pharmacologically_active\n_pdbe_chem_comp_drugbank_targets.ordinal\nGOL DB09462 'Peroxisome proliferator-activated receptor delta' Humans Q03181 yes 1\nGOL DB09462 'Group IIE secretory phospholipase A2' Humans Q9NZK7 unknown 2\nGOL DB09462 'Inositol-3-phosphate synthase 1' Humans Q9NPH2 unknown 3\nGOL DB09462 'All-trans-retinol dehydrogenase [NAD(+)] ADH1B' Humans P00325 unknown 4\nGOL DB09462 'Inositol 1,4,5-trisphosphate-gated calcium channel ITPR1' Humans Q14643 unknown 5\nGOL DB09462 Glycodelin Humans P09466 unknown 6\nGOL DB09462 Alpha-N-acetylgalactosaminidase Humans P17050 unknown 7\nGOL DB09462 'ADP-ribosylation factor 1' Humans P84077 unknown 8\nGOL DB09462 'Bifunctional 3'-phosphoadenosine 5'-phosphosulfate synthase 1' Humans O43252 unknown 9\nGOL DB09462 'DNA mismatch repair protein MutL' 'Escherichia coli (strain K12)' P23367 unknown 10\nGOL DB09462 'Ribonucleoside-diphosphate reductase 1 subunit beta' 'Escherichia coli (strain K12)' P69924 unknown 11\nGOL DB09462 'Adenine DNA glycosylase' 'Escherichia coli (strain K12)' P17802 unknown 12\nGOL DB09462 'tRNA (cytosine(38)-C(5))-methyltransferase' Humans O14717 unknown 13\nGOL DB09462 'Maleylacetoacetate isomerase' Humans O43708 unknown 14\n#\nloop_\n_software.name\n_software.version\n_software.description\nrdkit 2023.09.6 'Core functionality.'\npdbeccdutils 0.8.6 'Wrapper

to provide 2D templates and molecular
fragments.'\n#\nloop_\n_pdbe_chem_comp_atom_depiction.comp_id\n_pdbe_c
hem_comp_atom_depiction.atom_id\n_pdbe_chem_comp_atom_depiction.elemen
t\n_pdbe_chem_comp_atom_depiction.model_Cartn_x\n_pdbe_chem_comp_atom_
depiction.model_Cartn_y\n_pdbe_chem_comp_atom_depiction.pdbx_ordinal\n
GOL C1 C 7.702 1.357 1\nGOL O1 O 9.002 0.608 2\nGOL C2 C 6.404 0.608
3\nGOL O2 O 6.404 -0.892 4\nGOL C3 C 5.104 1.357
5\n#\nloop_\n_pdbe_chem_comp_bond_depiction.comp_id\n_pdbe_chem_comp_b
ond_depiction.atom_id_1\n_pdbe_chem_comp_bond_depiction.atom_id_2\n_pd
be_chem_comp_bond_depiction.value_order\n_pdbe_chem_comp_bond_depictio
n.bond_dir\n_pdbe_chem_comp_bond_depiction.pdbx_ordinal\nGOL C1 O1
SINGLE NONE 1\nGOL C1 C2 SINGLE NONE 2\nGOL C2 O2 SINGLE NONE 3\nGOL
C2 C3 SINGLE NONE 4\n#\n#\n#\n_pdbe_chem_comp_rdkit_properties.comp_id
GOL\n_pdbe_chem_comp_rdkit_properties.exactmw
92.047\n_pdbe_chem_comp_rdkit_properties.amw
92.094\n_pdbe_chem_comp_rdkit_properties.lipinskiHBA
3\n_pdbe_chem_comp_rdkit_properties.lipinskiHBD
3\n_pdbe_chem_comp_rdkit_properties.NumRotatableBonds
5\n_pdbe_chem_comp_rdkit_properties.NumHBD
3\n_pdbe_chem_comp_rdkit_properties.NumHBA
3\n_pdbe_chem_comp_rdkit_properties.NumHeavyAtoms
6\n_pdbe_chem_comp_rdkit_properties.NumAtoms
14\n_pdbe_chem_comp_rdkit_properties.NumHeteroatoms
3\n_pdbe_chem_comp_rdkit_properties.NumAmideBonds
0\n_pdbe_chem_comp_rdkit_properties.FractionCSP3
1\n_pdbe_chem_comp_rdkit_properties.NumRings
0\n_pdbe_chem_comp_rdkit_properties.NumAromaticRings
0\n_pdbe_chem_comp_rdkit_properties.NumAliphaticRings
0\n_pdbe_chem_comp_rdkit_properties.NumSaturatedRings
0\n_pdbe_chem_comp_rdkit_properties.NumHeterocycles
0\n_pdbe_chem_comp_rdkit_properties.NumAromaticHeterocycles
0\n_pdbe_chem_comp_rdkit_properties.NumSaturatedHeterocycles
0\n_pdbe_chem_comp_rdkit_properties.NumAliphaticHeterocycles
0\n_pdbe_chem_comp_rdkit_properties.NumSpiroAtoms
0\n_pdbe_chem_comp_rdkit_properties.NumBridgeheadAtoms
0\n_pdbe_chem_comp_rdkit_properties.NumAtomStereoCenters
0\n_pdbe_chem_comp_rdkit_properties.NumUnspecifiedAtomStereoCenters
0\n_pdbe_chem_comp_rdkit_properties.labuteASA
47.352\n_pdbe_chem_comp_rdkit_properties.tpsa
60.690\n_pdbe_chem_comp_rdkit_properties.CrippenClogP -
1.668\n_pdbe_chem_comp_rdkit_properties.CrippenMR
20.178\n_pdbe_chem_comp_rdkit_properties.chi0v
2.725\n_pdbe_chem_comp_rdkit_properties.chi1v
1.112\n_pdbe_chem_comp_rdkit_properties.chi2v
0.185\n_pdbe_chem_comp_rdkit_properties.chi3v
0.185\n_pdbe_chem_comp_rdkit_properties.chi4v
0.021\n_pdbe_chem_comp_rdkit_properties.chi0n
10.725\n_pdbe_chem_comp_rdkit_properties.chi1n
4.837\n_pdbe_chem_comp_rdkit_properties.chi2n
0.185\n_pdbe_chem_comp_rdkit_properties.chi3n
0.185\n_pdbe_chem_comp_rdkit_properties.chi4n
0.021\n_pdbe_chem_comp_rdkit_properties.hallKierAlpha -

0.120\n_pdbe_chem_comp_rdkit_properties.kappa1
0.844\n_pdbe_chem_comp_rdkit_properties.kappa2
3.085\n_pdbe_chem_comp_rdkit_properties.kappa3
2.138\n_pdbe_chem_comp_rdkit_properties.Phi
0.434\n#\nloop_\n_pdbe_chem_comp_external_mappings.comp_id\n_pdbe_chem_comp_external_mappings.source\n_pdbe_chem_comp_external_mappings.resource\n_pdbe_chem_comp_external_mappings.resource_id\nGOL UniChem ChEMBL CHEMBL692\nGOL UniChem DrugBank DB09462\nGOL UniChem 'Guide to Pharmacology' 5195\nGOL UniChem 'KEGG LIGAND' C00116\nGOL UniChem ChEBI 17754\nGOL UniChem ZINC ZINC000000895048\nGOL UniChem fdasrs PDC6A3C0OX\nGOL UniChem HMDB HMDB0000131\nGOL UniChem NMRShiftDB 10005613\nGOL UniChem LINCS LSM-37180\nGOL UniChem ACTor 8043-29-6\nGOL UniChem Recon glyc\nGOL UniChem Nikkaji J1.916J\nGOL UniChem 'EPA CompTox Dashboard' DTXSID9020663\nGOL UniChem DrugCentral 1316\nGOL UniChem MetaboLights MTBLC17754\nGOL UniChem BRENDA 106408\nGOL UniChem BRENDA 107129\nGOL UniChem BRENDA 135\nGOL UniChem BRENDA 161944\nGOL UniChem BRENDA 164880\nGOL UniChem BRENDA 166157\nGOL UniChem BRENDA 183683\nGOL UniChem BRENDA 234046\nGOL UniChem BRENDA 49999\nGOL UniChem Rhea 17754\nGOL UniChem ChemicalBook CB51261492\nGOL UniChem ChemicalBook CB5339206\nGOL UniChem ChemicalBook CB8398945\nGOL UniChem DailyMed GLYCERIN\nGOL UniChem rxnorm GLYCERIN\nGOL UniChem MedChemExpress HY-B1659\nGOL UniChem 'Probes And Drugs' PD006499\nGOL UniChem CCDC GLCROL\nGOL UniChem eMolecules 476493\nGOL UniChem SureChEMBL SCHEMBL797\nGOL UniChem 'PubChem TPHARMA' 14747384\nGOL UniChem PubChem 753\nGOL UniChem Mcule MCULE-6349111826\nGOL UniChem ACTor 175385-78-1\nGOL UniChem ACTor 29796-42-7\nGOL UniChem ACTor 56-81-5\n#\nloop_\n_pdbe_chem_comp_rdkit_conformer.comp_id\n_pdbe_chem_comp_rdkit_conformer.atom_id\n_pdbe_chem_comp_rdkit_conformer.Cartn_x_rdkit\n_pdbe_chem_comp_rdkit_conformer.Cartn_y_rdkit\n_pdbe_chem_comp_rdkit_conformer.Cartn_z_rdkit\n_pdbe_chem_comp_rdkit_conformer.rdkit_method\n_pdbe_chem_comp_rdkit_conformer.rdkit_ordinal\nGOL C1 1.314 0.182 -0.282 ETKDGv3 1\nGOL O1 1.700 -0.971 0.417 ETKDGv3 2\nGOL C2 -0.199 0.173 -0.551 ETKDGv3 3\nGOL O2 -0.552 -0.905 -1.382 ETKDGv3 4\nGOL C3 -1.016 0.158 0.754 ETKDGv3 5\nGOL H11 1.854 0.229 -1.253 ETKDGv3 7\nGOL H12 1.579 1.087 0.310 ETKDGv3 8\nGOL HO1 2.680 -0.894 0.553 ETKDGv3 9\nGOL H2 -0.443 1.112 -1.094 ETKDGv3 10\nGOL HO2 -0.391 -1.744 -0.877 ETKDGv3 11\nGOL H31 -0.735 1.026 1.393 ETKDGv3 12\nGOL H32 -0.798 -0.767 1.329 ETKDGv3 13\n#\ndata_PA1-2\n#\n_chem_comp.id PA1\n_chem_comp.name 2-amino-2-deoxy-alpha-D-glucopyranose\n_chem_comp.type 'D-saccharide, alpha linking'\n_chem_comp.pdbx_type ATOMS\n_chem_comp.formula 'C6 H13 N O5'\n_chem_comp.mon_nstd_parent_comp_id ?\n_chem_comp.pdbx_synonyms\n'alpah-D-glucosamine; 2-amino-2-deoxy-alpha-D-glucose; 2-amino-2-deoxy-D-glucose; 2-amino-2-deoxy-glucose'\n_chem_comp.pdbx_formal_charge 0\n_chem_comp.pdbx_initial_date 1999-07-08\n_chem_comp.pdbx_modified_date 2021-08-24\n_chem_comp.pdbx_ambiguous_flag N\n_chem_comp.pdbx_release_status REL\n_chem_comp.pdbx_replaced_by ?\n_chem_comp.pdbx_replaces ?\n_chem_comp.formula_weight
179.171\n_chem_comp.one_letter_code ?\n_chem_comp.three_letter_code ?\n_chem_comp.pdbx_model_coordinates_details ?\n_chem_comp.pdbx_model_co

ordinates_missing_flag
N\n_chem_comp.pdbx_ideal_coordinates_details ?\n_chem_comp.pdbx_ideal_
coordinates_missing_flag N\n_chem_comp.pdbx_model_coordinates_db_code
1PBR\n_chem_comp.pdbx_subcomponent_list ?\n_chem_comp.pdbx_processing_
site
RCSB\n#\nloop_\n_pdbx_chem_comp_synonyms.ordinal\n_pdbx_chem_comp_syno
nyms.comp_id\n_pdbx_chem_comp_synonyms.name\n_pdbx_chem_comp_synonyms.
provenance\n_pdbx_chem_comp_synonyms.type\n1 PA1 alpha-D-glucosamine
PDB ?\n2 PA1 2-amino-2-deoxy-alpha-D-glucose PDB ?\n3 PA1 2-amino-2-
deoxy-D-glucose PDB ?\n4 PA1 2-amino-2-deoxy-glucose
PDB ?\n#\nloop_\n_chem_comp_atom.comp_id\n_chem_comp_atom.atom_id\n_ch
em_comp_atom.alt_atom_id\n_chem_comp_atom.type_symbol\n_chem_comp_atom
.charge\n_chem_comp_atom.pdbx_align\n_chem_comp_atom.pdbx_aromatic_fla
g\n_chem_comp_atom.pdbx_leaving_atom_flag\n_chem_comp_atom.pdbx_stereo
_config\n_chem_comp_atom.pdbx_backbone_atom_flag\n_chem_comp_atom.pdbx
_n_terminal_atom_flag\n_chem_comp_atom.pdbx_c_terminal_atom_flag\n_che
m_comp_atom.model_Cartn_x\n_chem_comp_atom.model_Cartn_y\n_chem_comp_a
tom.model_Cartn_z\n_chem_comp_atom.pdbx_model_Cartn_x_ideal\n_chem_com
p_atom.pdbx_model_Cartn_y_ideal\n_chem_comp_atom.pdbx_model_Cartn_z_id
eal\n_chem_comp_atom.pdbx_component_atom_id\n_chem_comp_atom.pdbx_comp
onent_comp_id\n_chem_comp_atom.pdbx_ordinal\nPA1 C1 C1 C 0 1 N N S N N
N 1.980 -0.879 43.999 1.564 0.223 0.505 C1 PA1 1\nPA1 O1 O1 O 0 1 N Y
N N N N 1.764 -2.297 44.213 1.770 -1.188 0.422 O1 PA1 2\nPA1 C2 C2 C 0
1 N N R N N N 3.316 -0.407 44.685 0.529 0.524 1.591 C2 PA1 3\nPA1 N2
N2 N 0 1 N N N N N N 4.453 -1.352 44.535 1.026 0.047 2.889 N2 PA1
4\nPA1 C3 C3 C 0 1 N N R N N N 3.131 -0.192 46.199 -0.780 -0.193 1.249
C3 PA1 5\nPA1 O3 O3 O 0 1 N N N N N N 4.258 0.481 46.756 -1.802 0.195
2.169 O3 PA1 6\nPA1 C4 C4 C 0 1 N N S N N N 1.858 0.617 46.490 -1.194
0.194 -0.174 C4 PA1 7\nPA1 C5 C5 C 0 1 N N R N N N 0.618 -0.077 45.884
-0.029 -0.081 -1.127 C5 PA1 9\nPA1 O5 O5 O 0 1 N N N N N N 0.822 -
0.127 44.456 1.098 0.707 -0.753 O5 PA1 10\nPA1 C6 C6 C 0 1 N N N N N N
-0.699 0.652 46.145 -0.447 0.274 -2.556 C6 PA1 11\nPA1 O6 O6 O 0 1 N N
N N N N -1.035 0.630 47.526 0.636 0.011 -3.449 O6 PA1 12\nPA1 H1 H1 H
0 1 N N N N N N 2.009 -0.659 42.921 2.505 0.713 0.755 H1 PA1 13\nPA1
HO1 HO1 H 0 1 N Y N N N N 0.957 -2.581 43.799 2.427 -1.334 -0.271 HO1
PA1 14\nPA1 H2 H2 H 0 1 N N N N N N 3.604 0.559 44.233 0.354 1.599
1.640 H2 PA1 15\nPA1 HN21 HN21 H 0 0 N N N N N N 5.282 -0.986 45.017
1.886 0.543 3.070 HN21 PA1 16\nPA1 HN22 HN22 H 0 0 N Y N N N N 4.235 -
2.242 44.998 0.363 0.355 3.584 HN22 PA1 17\nPA1 H3 H3 H 0 1 N N N N N
N 3.044 -1.188 46.649 -0.629 -1.271 1.304 H3 PA1 18\nPA1 HO3 HO3 H 0 1
N Y N N N N 4.298 1.336 46.320 -1.501 -0.067 3.049 HO3 PA1 19\nPA1 H4
H4 H 0 1 N N N N N N 1.966 1.564 45.945 -1.446 1.255 -0.203 H4 PA1
20\nPA1 H5 H5 H 0 1 N N N N N N 0.519 -1.112 46.244 0.235 -1.137 -
1.080 H5 PA1 22\nPA1 H61 H61 H 0 1 N N N N N N -1.503 0.179 45.555 -
1.309 -0.327 -2.843 H61 PA1 23\nPA1 H62 H62 H 0 1 N N N N N N -0.639
1.691 45.797 -0.709 1.331 -2.605 H62 PA1 24\nPA1 HO6 HO6 H 0 1 N Y N N
N N -1.106 -0.297 47.767 0.332 0.248 -4.335 HO6 PA1
25\n#\nloop_\n_chem_comp_bond.comp_id\n_chem_comp_bond.atom_id_1\n_che
m_comp_bond.atom_id_2\n_chem_comp_bond.value_order\n_chem_comp_bond.pd
bx_aromatic_flag\n_chem_comp_bond.pdbx_stereo_config\n_chem_comp_bond.
pdbx_ordinal\nPA1 C1 O1 SING N N 1\nPA1 C1 C2 SING N N 2\nPA1 C1 O5
SING N N 3\nPA1 C1 H1 SING N N 4\nPA1 O1 HO1 SING N N 5\nPA1 C2 N2

SING N N 6\nPA1 C2 C3 SING N N 7\nPA1 C2 H2 SING N N 8\nPA1 N2 HN21 SING N N 9\nPA1 N2 HN22 SING N N 10\nPA1 C3 O3 SING N N 11\nPA1 C3 C4 SING N N 12\nPA1 C3 H3 SING N N 13\nPA1 O3 HO3 SING N N 14\nPA1 C4 C5 SING N N 16\nPA1 C4 H4 SING N N 17\nPA1 C5 O5 SING N N 19\nPA1 C5 C6 SING N N 20\nPA1 C5 H5 SING N N 21\nPA1 C6 O6 SING N N 22\nPA1 C6 H61 SING N N 23\nPA1 C6 H62 SING N N 24\nPA1 O6 HO6 SING N N 25\n#\nloop_\n_pdbx_chem_comp_descriptor.comp_id\n_pdbx_chem_comp_descriptor.type\n_pdbx_chem_comp_descriptor.program\n_pdbx_chem_comp_descriptor.program_version\n_pdbx_chem_comp_descriptor.descriptor\nPA1 SMILES ACDLabs 10.04 OC1C(O)C(OC(O)C1N)CO\nPA1 SMILES_CANONICAL CACTVS 3.341 N[C@H]1[C@@H](O)O[C@H](CO)[C@@H](O)[C@@H]1O\nPA1 SMILES CACTVS 3.341 N[CH]1[CH](O)O[CH](CO)[CH](O)[CH]1O\nPA1 SMILES_CANONICAL 'OpenEye OEToolkits' 1.5.0 C([C@@H]1[C@H]([C@@H]([C@H]([C@H](O1)O)N)O)O)O\nPA1 SMILES 'OpenEye OEToolkits' 1.5.0 C(C1C(C(C(C(O1)O)N)O)O)O\nPA1 InChI InChI 1.03 InChI=1S/C6H13NO5/c7-3-5(10)4(9)2(1-8)12-6(3)11/h2-6,8-11H,1,7H2/t2-,3-,4-,5-,6+/m1/s1\nPA1 InChIKey InChI 1.03 MSWZFWKMSRAUBD-UKFBFLRUSA-N\n#\nloop_\n_pdbx_chem_comp_identifier.comp_id\n_pdbx_chem_comp_identifier.type\n_pdbx_chem_comp_identifier.program\n_pdbx_chem_comp_identifier.program_version\n_pdbx_chem_comp_identifier.identifier\nPA1 'SYSTEMATIC NAME' ACDLabs 10.04 2-amino-2-deoxy-alpha-D-glucopyranose\nPA1 'SYSTEMATIC NAME' 'OpenEye OEToolkits' 1.5.0 (2S,3R,4R,5S,6R)-3-amino-6-(hydroxymethyl)oxane-2,4,5-triol\nPA1 'CONDENSED IUPAC CARBOHYDRATE SYMBOL' GMML 1.0 DGlcpNa\nPA1 'COMMON NAME' GMML 1.0 a-D-glucopyranosamine\nPA1 'IUPAC CARBOHYDRATE SYMBOL' PDB-CARE 1.0 a-D-GlcpN\nPA1 'SNFG CARBOHYDRATE SYMBOL' GMML 1.0 GlcN\n#\nloop_\n_pdbx_chem_comp_feature.comp_id\n_pdbx_chem_comp_feature.type\n_pdbx_chem_comp_feature.value\n_pdbx_chem_comp_feature.source\n_pdbx_chem_comp_feature.support\nPA1 'CARBOHYDRATE ISOMER' D PDB ?\nPA1 'CARBOHYDRATE RING' pyranose PDB ?\nPA1 'CARBOHYDRATE ANOMER' alpha PDB ?\nPA1 'CARBOHYDRATE PRIMARY CARBONYL GROUP' aldose PDB ?\n#\nloop_\n_pdbx_chem_comp_audit.comp_id\n_pdbx_chem_comp_audit.action_type\n_pdbx_chem_comp_audit.date\n_pdbx_chem_comp_audit.processing_site\nPA1 'Create component' 1999-07-08 RCSB\nPA1 'Modify descriptor' 2011-06-04 RCSB\nPA1 'Modify leaving atom flag' 2012-06-13 RCSB\nPA1 'Other modification' 2019-08-12 RCSB\nPA1 'Other modification' 2019-12-19 RCSB\nPA1 'Other modification' 2020-07-03 RCSB\nPA1 'Modify synonyms' 2020-07-17 RCSB\nPA1 'Modify synonyms' 2021-08-24 RCSB\n#\nloop_\n_pdbe_chem_comp_synonyms.comp_id\n_pdbe_chem_comp_synonyms.name\n_pdbe_chem_comp_synonyms.provenance\n_pdbe_chem_comp_synonyms.type\nPA1 alpha-D-glucosamine wwPDB ?\nPA1 2-amino-2-deoxy-alpha-D-glucose wwPDB ?\nPA1 2-amino-2-deoxy-D-glucose wwPDB ?\nPA1 2-amino-2-deoxy-glucose wwPDB ?\n#\nloop_\n_software.name\n_software.version\n_software.description\nrdkit 2023.09.6 'Core functionality.'\npdbeccdutils 0.8.6 'Wrapper to provide 2D templates and molecular fragments.'\n#\nloop_\n_pdbe_chem_comp_atom_depiction.comp_id\n_pdbe_chem_comp_atom_depiction.atom_id\n_pdbe_chem_comp_atom_depiction.element\n_pdbe_chem_comp_atom_depiction.model_Cartn_x\n_pdbe_chem_comp_atom_depiction.model_Cartn_y\n_pdbe_chem_comp_atom_depiction.pdbx_ordinal\n

PA1 C1 C 7.702 0.750 1\nPA1 O1 O 9.002 1.500 2\nPA1 C2 C 7.702 -0.750
3\nPA1 N2 N 9.002 -1.500 4\nPA1 C3 C 6.404 -1.500 5\nPA1 O3 O 6.404 -
3.000 6\nPA1 C4 C 5.104 -0.750 7\nPA1 C5 C 5.104 0.750 9\nPA1 O5 O
6.404 1.500 10\nPA1 C6 C 3.805 1.500 11\nPA1 O6 O 3.805 3.000
12\n#\nloop_\n_pdbe_chem_comp_bond_depiction.comp_id\n_pdbe_chem_comp_
bond_depiction.atom_id_1\n_pdbe_chem_comp_bond_depiction.atom_id_2\n_p
dbe_chem_comp_bond_depiction.value_order\n_pdbe_chem_comp_bond_depicti
on.bond_dir\n_pdbe_chem_comp_bond_depiction.pdbx_ordinal\nPA1 C1 O1
SINGLE BEGINDASH 1\nPA1 C1 C2 SINGLE NONE 2\nPA1 C1 O5 SINGLE NONE
3\nPA1 C2 N2 SINGLE BEGINDASH 4\nPA1 C2 C3 SINGLE NONE 5\nPA1 C3 O3
SINGLE BEGINWEDGE 6\nPA1 C3 C4 SINGLE NONE 7\nPA1 C4 C5 SINGLE NONE
9\nPA1 C5 O5 SINGLE NONE 10\nPA1 C5 C6 SINGLE BEGINWEDGE 11\nPA1 C6 O6
SINGLE NONE
12\n#\nloop_\n_pdbe_chem_comp_substructure.comp_id\n_pdbe_chem_comp_su
bstructure.substructure_name\n_pdbe_chem_comp_substructure.id\n_pdbe_c
hem_comp_substructure.substructure_type\n_pdbe_chem_comp_substructure.
substructure_smiles\n_pdbe_chem_comp_substructure.substructure_inchis\
n_pdbe_chem_comp_substructure.substructure_inchikeys\nPA1
MurckoScaffold S1 scaffold C1CCOCC1 InChI=1S/C5H10O/c1-2-4-6-5-3-1/h1-
5H2 DHXVGJBLRPWPCS-UHFFFAOYSA-N\nPA1 pyranose F1 fragment OC1CCCCO1
InChI=1S/C5H10O2/c6-5-3-1-2-4-7-5/h5-6H,1-4H2 CELWCAITJAEQNL-
UHFFFAOYSA-
N\n#\nloop_\n_pdbe_chem_comp_substructure_mapping.comp_id\n_pdbe_chem_
comp_substructure_mapping.atom_id\n_pdbe_chem_comp_substructure_mappin
g.substructure_id\n_pdbe_chem_comp_substructure_mapping.substructure_o
rdinal\nPA1 C1 S1 1\nPA1 C2 S1 1\nPA1 C3 S1 1\nPA1 C4 S1 1\nPA1 C5 S1
1\nPA1 O5 S1 1\nPA1 C4 F1 1\nPA1 C5 F1 1\nPA1 O5 F1 1\nPA1 C1 F1
1\nPA1 C2 F1 1\nPA1 C3 F1 1\nPA1 O1 F1
1\n#\n_pdbe_chem_comp_rdkit_properties.comp_id
PA1\n_pdbe_chem_comp_rdkit_properties.exactmw
179.079\n_pdbe_chem_comp_rdkit_properties.amw
179.172\n_pdbe_chem_comp_rdkit_properties.lipinskiHBA
6\n_pdbe_chem_comp_rdkit_properties.lipinskiHBD
6\n_pdbe_chem_comp_rdkit_properties.NumRotatableBonds
6\n_pdbe_chem_comp_rdkit_properties.NumHBD
5\n_pdbe_chem_comp_rdkit_properties.NumHBA
6\n_pdbe_chem_comp_rdkit_properties.NumHeavyAtoms
12\n_pdbe_chem_comp_rdkit_properties.NumAtoms
25\n_pdbe_chem_comp_rdkit_properties.NumHeteroatoms
6\n_pdbe_chem_comp_rdkit_properties.NumAmideBonds
0\n_pdbe_chem_comp_rdkit_properties.FractionCSP3
1\n_pdbe_chem_comp_rdkit_properties.NumRings
1\n_pdbe_chem_comp_rdkit_properties.NumAromaticRings
0\n_pdbe_chem_comp_rdkit_properties.NumAliphaticRings
1\n_pdbe_chem_comp_rdkit_properties.NumSaturatedRings
1\n_pdbe_chem_comp_rdkit_properties.NumHeterocycles
1\n_pdbe_chem_comp_rdkit_properties.NumAromaticHeterocycles
0\n_pdbe_chem_comp_rdkit_properties.NumSaturatedHeterocycles
1\n_pdbe_chem_comp_rdkit_properties.NumAliphaticHeterocycles
1\n_pdbe_chem_comp_rdkit_properties.NumSpiroAtoms
0\n_pdbe_chem_comp_rdkit_properties.NumBridgeheadAtoms
0\n_pdbe_chem_comp_rdkit_properties.NumAtomStereoCenters

5\n_pdbe_chem_comp_rdkit_properties.NumUnspecifiedAtomStereoCenters
0\n_pdbe_chem_comp_rdkit_properties.labuteASA
87.916\n_pdbe_chem_comp_rdkit_properties.tpsa
116.170\n_pdbe_chem_comp_rdkit_properties.CrippenClogP -
3.255\n_pdbe_chem_comp_rdkit_properties.CrippenMR
37.955\n_pdbe_chem_comp_rdkit_properties.chi0v
5.488\n_pdbe_chem_comp_rdkit_properties.chi1v
2.698\n_pdbe_chem_comp_rdkit_properties.chi2v
1.108\n_pdbe_chem_comp_rdkit_properties.chi3v
1.108\n_pdbe_chem_comp_rdkit_properties.chi4v
0.558\n_pdbe_chem_comp_rdkit_properties.chi0n
18.488\n_pdbe_chem_comp_rdkit_properties.chi1n
8.726\n_pdbe_chem_comp_rdkit_properties.chi2n
1.108\n_pdbe_chem_comp_rdkit_properties.chi3n
1.108\n_pdbe_chem_comp_rdkit_properties.chi4n
0.558\n_pdbe_chem_comp_rdkit_properties.hallKierAlpha -
0.240\n_pdbe_chem_comp_rdkit_properties.kappa1
2.221\n_pdbe_chem_comp_rdkit_properties.kappa2
3.649\n_pdbe_chem_comp_rdkit_properties.kappa3
1.582\n_pdbe_chem_comp_rdkit_properties.Phi
0.675\n#\nloop_\n_pdbe_chem_comp_external_mappings.comp_id\n_pdbe_chem
_comp_external_mappings.source\n_pdbe_chem_comp_external_mappings.reso
urce\n_pdbe_chem_comp_external_mappings.resource_id\nPA1 UniChem ChEBI
44678\nPA1 UniChem ZINC ZINC000003860469\nPA1 UniChem atlas
glucosamine\nPA1 UniChem atlas glucosamine,\nPA1 UniChem atlas
'glucosamine, steady state'\nPA1 UniChem fdasrs S3BL0640UV\nPA1
UniChem NMRShiftDB 20212431\nPA1 UniChem LINCS LSM-36429\nPA1 UniChem
MetaboLights MTBLC44678\nPA1 UniChem 'Probes And Drugs' PD057482\nPA1
UniChem Nikkaji J361.550B\nPA1 UniChem SureChEMBL SCHEMBL429222\nPA1
UniChem 'PubChem TPHARMA' 15219649\nPA1 UniChem PubChem 445621\nPA1
UniChem Mcule MCULE-
9890197257\n#\nloop_\n_pdbe_chem_comp_rdkit_conformer.comp_id\n_pdbe_c
hem_comp_rdkit_conformer.atom_id\n_pdbe_chem_comp_rdkit_conformer.Cart
n_x_rdkit\n_pdbe_chem_comp_rdkit_conformer.Cartn_y_rdkit\n_pdbe_chem_c
omp_rdkit_conformer.Cartn_z_rdkit\n_pdbe_chem_comp_rdkit_conformer.rdk
it_method\n_pdbe_chem_comp_rdkit_conformer.rdkit_ordinal\nPA1 C1 0.783
1.254 0.836 ETKDGv3 1\nPA1 O1 1.033 2.393 0.052 ETKDGv3 2\nPA1 C2
1.679 0.047 0.399 ETKDGv3 3\nPA1 N2 2.118 0.190 -0.996 ETKDGv3 4\nPA1
C3 0.912 -1.285 0.576 ETKDGv3 5\nPA1 O3 0.779 -1.600 1.943 ETKDGv3
6\nPA1 C4 -0.473 -1.252 -0.130 ETKDGv3 7\nPA1 C5 -0.987 0.201 -0.283
ETKDGv3 9\nPA1 O5 -0.612 0.968 0.844 ETKDGv3 10\nPA1 C6 -2.516 0.228 -
0.420 ETKDGv3 11\nPA1 O6 -2.967 1.543 -0.600 ETKDGv3 12\nPA1 H1 1.065
1.492 1.887 ETKDGv3 13\nPA1 HO1 0.533 3.140 0.472 ETKDGv3 14\nPA1 H2
2.585 0.019 1.048 ETKDGv3 15\nPA1 HN21 2.686 -0.649 -1.257 ETKDGv3
16\nPA1 HN22 2.782 0.996 -1.052 ETKDGv3 17\nPA1 H3 1.522 -2.103 0.132
ETKDGv3 18\nPA1 HO3 0.163 -0.942 2.356 ETKDGv3 19\nPA1 H4 -1.198 -
1.835 0.487 ETKDGv3 20\nPA1 H5 -0.569 0.651 -1.215 ETKDGv3 22\nPA1 H61
-2.982 -0.180 0.502 ETKDGv3 23\nPA1 H62 -2.838 -0.408 -1.276 ETKDGv3
24\nPA1 HO6 -2.766 1.787 -1.542 ETKDGv3 25\n#\ndata_MAN-
2\n#\n_chem_comp.id MAN\n_chem_comp.name alpha-D-
mannopyranose\n_chem_comp.type 'D-saccharide, alpha
linking'\n_chem_comp.pdbx_type ATOMS\n_chem_comp.formula 'C6 H12

O6'\n_chem_comp.mon_nstd_parent_comp_id ?\n_chem_comp.pdbx_synonyms 'alpha-D-mannose; D-mannose; mannose'\n_chem_comp.pdbx_formal_charge 0\n_chem_comp.pdbx_initial_date 1999-07-
08\n_chem_comp.pdbx_modified_date 2024-09-
27\n_chem_comp.pdbx_ambiguous_flag N\n_chem_comp.pdbx_release_status REL\n_chem_comp.pdbx_replaced_by ?\n_chem_comp.pdbx_replaces ?\n_chem_comp.formula_weight
180.156\n_chem_comp.one_letter_code ?\n_chem_comp.three_letter_code MAN\n_chem_comp.pdbx_model_coordinates_details ?\n_chem_comp.pdbx_model_coordinates_missing_flag
N\n_chem_comp.pdbx_ideal_coordinates_details ?\n_chem_comp.pdbx_ideal_coordinates_missing_flag N\n_chem_comp.pdbx_model_coordinates_db_code 1GPZ\n_chem_comp.pdbx_subcomponent_list ?\n_chem_comp.pdbx_processing_site RCSB\n_chem_comp.pdbx_pcm
Y\n#\nloop_\n_pdbx_chem_comp_synonyms.ordinal\n_pdbx_chem_comp_synonyms.comp_id\n_pdbx_chem_comp_synonyms.name\n_pdbx_chem_comp_synonyms.provenance\n_pdbx_chem_comp_synonyms.type\n1 MAN alpha-D-mannose PDB ?\n2 MAN D-mannose PDB ?\n3 MAN mannose
PDB ?\n#\nloop_\n_chem_comp_atom.comp_id\n_chem_comp_atom.atom_id\n_chem_comp_atom.alt_atom_id\n_chem_comp_atom.type_symbol\n_chem_comp_atom.charge\n_chem_comp_atom.pdbx_align\n_chem_comp_atom.pdbx_aromatic_flag\n_chem_comp_atom.pdbx_leaving_atom_flag\n_chem_comp_atom.pdbx_stereo_config\n_chem_comp_atom.pdbx_backbone_atom_flag\n_chem_comp_atom.pdbx_n_terminal_atom_flag\n_chem_comp_atom.pdbx_c_terminal_atom_flag\n_chem_comp_atom.model_Cartn_x\n_chem_comp_atom.model_Cartn_y\n_chem_comp_atom.model_Cartn_z\n_chem_comp_atom.pdbx_model_Cartn_x_ideal\n_chem_comp_atom.pdbx_model_Cartn_y_ideal\n_chem_comp_atom.pdbx_model_Cartn_z_ideal\n_chem_comp_atom.pdbx_component_atom_id\n_chem_comp_atom.pdbx_component_comp_id\n_chem_comp_atom.pdbx_ordinal\nMAN C1 C1 C 0 1 N N S N N N 99.738 -29.415 24.222 -1.692 -0.156 -0.316 C1 MAN 1\nMAN C2 C2 C 0 1 N N S N N N 101.239 -29.305 24.564 -0.878 0.091 -1.588 C2 MAN 2\nMAN C3 C3 C 0 1 N N S N N N 102.016 -28.461 23.551 0.535 -0.467 -1.391 C3 MAN 3\nMAN C4 C4 C 0 1 N N S N N N 101.699 -28.940 22.129 1.126 0.134 -0.111 C4 MAN 4\nMAN C5 C5 C 0 1 N N R N N N 100.197 -28.798 21.881 0.160 -0.117 1.048 C5 MAN 5\nMAN C6 C6 C 0 1 N N N N N N 99.829 -29.254 20.463 0.757 0.448 2.339 C6 MAN 6\nMAN O1 O1 O 0 1 N Y N N N N 99.000 -28.349 24.713 -1.735 -1.558 -0.046 O1 MAN 7\nMAN O3 O3 O 0 1 N N N N N N 103.406 -28.578 23.812 1.350 -0.113 -2.511 O3 MAN 9\nMAN O4 O4 O 0 1 N N N N N N 102.419 -28.180 21.167 2.384 -0.482 0.170 O4 MAN 10\nMAN O5 O5 O 0 1 N N N N N N 99.454 -29.636 22.812 -1.087 0.520 0.784 O5 MAN 11\nMAN O6 O6 O 0 1 N N N N N N 98.821 -28.437 19.876 -0.142 0.211 3.423 O6 MAN 12\nMAN H1 H1 H 0 1 N N N N N N 99.408 -30.340 24.750 -2.707 0.216 -0.457 H1 MAN 13\nMAN H2 H2 H 0 1 N N N N N N 101.314 -28.790 25.550 -1.354 -0.410 -2.430 H2 MAN 14\nMAN H3 H3 H 0 1 N N N N N N 101.716 -27.391 23.643 0.491 -1.552 -1.300 H3 MAN 15\nMAN H4 H4 H 0 1 N N N N N N 102.004 -30.007 22.028 1.267 1.207 -0.244 H4 MAN 16\nMAN H5 H5 H 0 1 N N N N N N 99.938 -27.722 22.018 0.002 -1.189 1.162 H5 MAN 17\nMAN H61 H61 H 0 1 N N N N N N 100.731 -29.309 19.811 0.915 1.521 2.226 H61 MAN 18\nMAN H62 H62 H 0 1 N N N N N N 99.533 -30.329 20.450 1.710 -0.039 2.543 H62 MAN 19\nMAN HO1 HO1 H 0 1 N Y N N N N 98.076 -28.416 24.502 -2.260 -1.672 0.757 HO1 MAN 20\nMAN HO3 HO3 H 0 1 N Y N N N N 103.888 -28.054 23.183 0.934 -0.501

-3.293 HO3 MAN 22\nMAN HO4 HO4 H 0 1 N Y N N N N 102.222 -28.476 20.286 2.958 -0.305 -0.587 HO4 MAN 23\nMAN HO6 HO6 H 0 1 N Y N N N N 98.593 -28.719 18.998 0.270 0.582 4.215 HO6 MAN 24\n#\nloop_\n_chem_comp_bond.comp_id\n_chem_comp_bond.atom_id_1\n_chem_comp_bond.atom_id_2\n_chem_comp_bond.value_order\n_chem_comp_bond.pdbx_aromatic_flag\n_chem_comp_bond.pdbx_stereo_config\n_chem_comp_bond.pdbx_ordinal\nMAN C1 C2 SING N N 1\nMAN C1 O1 SING N N 2\nMAN C1 O5 SING N N 3\nMAN C1 H1 SING N N 4\nMAN C2 C3 SING N N 5\nMAN C2 H2 SING N N 7\nMAN C3 C4 SING N N 8\nMAN C3 O3 SING N N 9\nMAN C3 H3 SING N N 10\nMAN C4 C5 SING N N 11\nMAN C4 O4 SING N N 12\nMAN C4 H4 SING N N 13\nMAN C5 C6 SING N N 14\nMAN C5 O5 SING N N 15\nMAN C5 H5 SING N N 16\nMAN C6 O6 SING N N 17\nMAN C6 H61 SING N N 18\nMAN C6 H62 SING N N 19\nMAN O1 HO1 SING N N 20\nMAN O3 HO3 SING N N 22\nMAN O4 HO4 SING N N 23\nMAN O6 HO6 SING N N 24\n#\nloop_\n_pdbx_chem_comp_descriptor.comp_id\n_pdbx_chem_comp_descriptor.type\n_pdbx_chem_comp_descriptor.program\n_pdbx_chem_comp_descriptor.program_version\n_pdbx_chem_comp_descriptor.descriptor\nMAN SMILES ACDLabs 10.04 'OC1C(O)C(OC(O)C1O)CO'\nMAN SMILES_CANONICAL CACTVS 3.341 'OC[C@H]1O[C@H](O)[C@@H](O)[C@@H](O)[C@@H]1O'\nMAN SMILES CACTVS 3.341 'OC[CH]1O[CH](O)[CH](O)[CH](O)[CH]1O'\nMAN SMILES_CANONICAL 'OpenEye OEToolkits' 1.5.0 'C([C@@H]1[C@H]([C@@H]([C@@H]([C@H](O1)O)O)O)O)O'\nMAN SMILES 'OpenEye OEToolkits' 1.5.0 'C(C1C(C(C(C(O1)O)O)O)O)O'\nMAN InChI InChI 1.03 'InChI=1S/C6H12O6/c7-1-2-3(8)4(9)5(10)6(11)12-2/h2-11H,1H2/t2-,3-,4+,5+,6+/m1/s1'\nMAN InChIKey InChI 1.03 WQZGKKKJIJFFOK-PQMKYFCFSA-N\n#\nloop_\n_pdbx_chem_comp_identifier.comp_id\n_pdbx_chem_comp_identifier.type\n_pdbx_chem_comp_identifier.program\n_pdbx_chem_comp_identifier.program_version\n_pdbx_chem_comp_identifier.identifier\nMAN 'SYSTEMATIC NAME' ACDLabs 10.04 alpha-D-mannopyranose\nMAN 'SYSTEMATIC NAME' 'OpenEye OEToolkits' 1.5.0 '(2S,3S,4S,5S,6R)-6-(hydroxymethyl)oxane-2,3,4,5-tetrol'\nMAN 'CONDENSED IUPAC CARBOHYDRATE SYMBOL' GMML 1.0 DManpa\nMAN 'COMMON NAME' GMML 1.0 a-D-mannopyranose\nMAN 'IUPAC CARBOHYDRATE SYMBOL' PDB-CARE 1.0 a-D-Manp\nMAN 'SNFG CARBOHYDRATE SYMBOL' GMML 1.0 Man\n#\nloop_\n_pdbx_chem_comp_feature.comp_id\n_pdbx_chem_comp_feature.type\n_pdbx_chem_comp_feature.value\n_pdbx_chem_comp_feature.source\n_pdbx_chem_comp_feature.support\nMAN 'CARBOHYDRATE ISOMER' D PDB ?\nMAN 'CARBOHYDRATE RING' pyranose PDB ?\nMAN 'CARBOHYDRATE ANOMER' alpha PDB ?\nMAN 'CARBOHYDRATE PRIMARY CARBONYL GROUP' aldose PDB ?\n#\nloop_\n_pdbx_chem_comp_audit.comp_id\n_pdbx_chem_comp_audit.action_type\n_pdbx_chem_comp_audit.date\n_pdbx_chem_comp_audit.processing_site\nMAN 'Create component' 1999-07-08 RCSB\nMAN 'Modify descriptor' 2011-06-04 RCSB\nMAN 'Other modification' 2019-08-12 RCSB\nMAN 'Other modification' 2019-12-19 RCSB\nMAN 'Other modification' 2020-07-03 RCSB\nMAN 'Modify name' 2020-07-17 RCSB\nMAN 'Modify synonyms' 2020-07-17 RCSB\nMAN 'Modify PCM' 2024-09-27 PDBE\n#\nloop_\n_pdbx_chem_comp_pcm.pcm_id\n_pdbx_chem_comp_pcm.comp_id\n_pdbx_chem_comp_pcm.modified_residue_id\n_pdbx_chem_comp_pcm.type\n_pdbx_chem_comp_pcm.category\n_pdbx_chem_comp_pcm.position\n_pdbx_chem_comp_pcm.polypeptide_position\n_pdbx_chem_comp_pcm.comp_id_linking_atom\n_pdbx_chem_comp_pcm.modified_residue_id_linking_atom\n_pdbx_chem_c

omp_pcm.uniprot_specific_ptm_accession\n_pdbx_chem_comp_pcm.uniprot_generic_ptm_accession\n1 MAN SER O-Glycosylation Carbohydrate 'Amino-acid side chain' 'Any position' C1 OG ? ?\n2 MAN THR O-Glycosylation Carbohydrate 'Amino-acid side chain' 'Any position' C1 OG1 ? ?\n3 MAN TRP C-Mannosylation Carbohydrate 'Amino-acid side chain' 'Any position' C1 CD1 ? ?\n4 MAN 3FG None Carbohydrate 'Amino-acid side chain' 'Any position' C1 OD1 ? ?\n5 MAN ARG None Carbohydrate 'Amino-acid side chain' 'Any position' C4 NH2 ? ?\n6 MAN ASN None Carbohydrate 'Amino-acid side chain' 'Any position' C1 ND2 ? ?\n7 MAN D4P None Carbohydrate 'Amino-acid side chain' 'Any position' C1 O4 ? ?\n8 MAN GLU None Carbohydrate 'Amino-acid side chain' 'Any position' C1 OE1 ? ?\n9 MAN HG7 None Carbohydrate 'Amino-acid side chain' 'Any position' O1 C5 ? ?\n10 MAN MDF None Carbohydrate 'Amino-acid side chain' 'Any position' C1 OH2 ? ?\n11 MAN SER None Carbohydrate 'Amino-acid side chain' 'Any position' O1 CB ? ?\n12 MAN THR None Carbohydrate 'Amino-acid side chain' 'Any position' O1 CB ? ?\n#\nloop_\n_pdbe_chem_comp_synonyms.comp_id\n_pdbe_chem_comp_synonyms.name\n_pdbe_chem_comp_synonyms.provenance\n_pdbe_chem_comp_synonyms.type\nMAN alpha-D-mannose wwPDB ?\nMAN D-mannose wwPDB ?\nMAN mannose
wwPDB ?\n#\nloop_\n_software.name\n_software.version\n_software.description\nrdkit 2023.09.6 'Core functionality.'\npdbeccdutils 0.8.6 'Wrapper to provide 2D templates and molecular fragments.'\n#\nloop_\n_pdbe_chem_comp_atom_depiction.comp_id\n_pdbe_chem_comp_atom_depiction.atom_id\n_pdbe_chem_comp_atom_depiction.element\n_pdbe_chem_comp_atom_depiction.model_Cartn_x\n_pdbe_chem_comp_atom_depiction.model_Cartn_y\n_pdbe_chem_comp_atom_depiction.pdbx_ordinal\nMAN C1 C 7.702 0.750 1\nMAN C2 C 7.702 -0.750 2\nMAN C3 C 6.404 -1.500 3\nMAN C4 C 5.104 -0.750 4\nMAN C5 C 5.104 0.750 5\nMAN C6 C 3.805 1.500 6\nMAN O1 O 9.002 1.500 7\nMAN O3 O 6.404 -3.000 9\nMAN O4 O 3.805 -1.500 10\nMAN O5 O 6.404 1.500 11\nMAN O6 O 3.805 3.000 12\n#\nloop_\n_pdbe_chem_comp_bond_depiction.comp_id\n_pdbe_chem_comp_bond_depiction.atom_id_1\n_pdbe_chem_comp_bond_depiction.atom_id_2\n_pdbe_chem_comp_bond_depiction.value_order\n_pdbe_chem_comp_bond_depiction.bond_dir\n_pdbe_chem_comp_bond_depiction.pdbx_ordinal\nMAN C1 C2 SINGLE NONE 1\nMAN C1 O1 SINGLE BEGINDASH 2\nMAN C1 O5 SINGLE NONE 3\nMAN C2 C3 SINGLE NONE 4\nMAN C3 C4 SINGLE NONE 6\nMAN C3 O3 SINGLE BEGINWEDGE 7\nMAN C4 C5 SINGLE NONE 8\nMAN C4 O4 SINGLE BEGINDASH 9\nMAN C5 C6 SINGLE BEGINWEDGE 10\nMAN C5 O5 SINGLE NONE 11\nMAN C6 O6 SINGLE NONE
12\n#\nloop_\n_pdbe_chem_comp_substructure.comp_id\n_pdbe_chem_comp_substructure.substructure_name\n_pdbe_chem_comp_substructure.id\n_pdbe_chem_comp_substructure.substructure_type\n_pdbe_chem_comp_substructure.substructure_smiles\n_pdbe_chem_comp_substructure.substructure_inchis\n_pdbe_chem_comp_substructure.substructure_inchikeys\nMAN MurckoScaffold S1 scaffold C1CCOCC1 InChI=1S/C5H10O/c1-2-4-6-5-3-1/h1-5H2 DHXVGJBLRPWPCS-UHFFFAOYSA-N\nMAN pyranose F1 fragment OC1CCCCO1 InChI=1S/C5H10O2/c6-5-3-1-2-4-7-5/h5-6H,1-4H2 CELWCAITJAEQNL-UHFFFAOYSA-
N\n#\nloop_\n_pdbe_chem_comp_substructure_mapping.comp_id\n_pdbe_chem_comp_substructure_mapping.atom_id\n_pdbe_chem_comp_substructure_mapping.substructure_id\n_pdbe_chem_comp_substructure_mapping.substructure_o

rdinal\nMAN C1 S1 1\nMAN C2 S1 1\nMAN C3 S1 1\nMAN C4 S1 1\nMAN C5 S1
1\nMAN O5 S1 1\nMAN C4 F1 1\nMAN C5 F1 1\nMAN O5 F1 1\nMAN C1 F1
1\nMAN C2 F1 1\nMAN C3 F1 1\nMAN O1 F1
1\n#\n_pdbe_chem_comp_rdkit_properties.comp_id
MAN\n_pdbe_chem_comp_rdkit_properties.exactmw
180.063\n_pdbe_chem_comp_rdkit_properties.amw
180.156\n_pdbe_chem_comp_rdkit_properties.lipinskiHBA
6\n_pdbe_chem_comp_rdkit_properties.lipinskiHBD
5\n_pdbe_chem_comp_rdkit_properties.NumRotatableBonds
6\n_pdbe_chem_comp_rdkit_properties.NumHBD
5\n_pdbe_chem_comp_rdkit_properties.NumHBA
6\n_pdbe_chem_comp_rdkit_properties.NumHeavyAtoms
12\n_pdbe_chem_comp_rdkit_properties.NumAtoms
24\n_pdbe_chem_comp_rdkit_properties.NumHeteroatoms
6\n_pdbe_chem_comp_rdkit_properties.NumAmideBonds
0\n_pdbe_chem_comp_rdkit_properties.FractionCSP3
1\n_pdbe_chem_comp_rdkit_properties.NumRings
1\n_pdbe_chem_comp_rdkit_properties.NumAromaticRings
0\n_pdbe_chem_comp_rdkit_properties.NumAliphaticRings
1\n_pdbe_chem_comp_rdkit_properties.NumSaturatedRings
1\n_pdbe_chem_comp_rdkit_properties.NumHeterocycles
1\n_pdbe_chem_comp_rdkit_properties.NumAromaticHeterocycles
0\n_pdbe_chem_comp_rdkit_properties.NumSaturatedHeterocycles
1\n_pdbe_chem_comp_rdkit_properties.NumAliphaticHeterocycles
1\n_pdbe_chem_comp_rdkit_properties.NumSpiroAtoms
0\n_pdbe_chem_comp_rdkit_properties.NumBridgeheadAtoms
0\n_pdbe_chem_comp_rdkit_properties.NumAtomStereoCenters
5\n_pdbe_chem_comp_rdkit_properties.NumUnspecifiedAtomStereoCenters
0\n_pdbe_chem_comp_rdkit_properties.labuteASA
85.936\n_pdbe_chem_comp_rdkit_properties.tpsa
110.380\n_pdbe_chem_comp_rdkit_properties.CrippenClogP -
3.221\n_pdbe_chem_comp_rdkit_properties.CrippenMR
35.986\n_pdbe_chem_comp_rdkit_properties.chi0v
5.449\n_pdbe_chem_comp_rdkit_properties.chi1v
2.679\n_pdbe_chem_comp_rdkit_properties.chi2v
1.092\n_pdbe_chem_comp_rdkit_properties.chi3v
1.092\n_pdbe_chem_comp_rdkit_properties.chi4v
0.552\n_pdbe_chem_comp_rdkit_properties.chi0n
17.449\n_pdbe_chem_comp_rdkit_properties.chi1n
8.220\n_pdbe_chem_comp_rdkit_properties.chi2n
1.092\n_pdbe_chem_comp_rdkit_properties.chi3n
1.092\n_pdbe_chem_comp_rdkit_properties.chi4n
0.552\n_pdbe_chem_comp_rdkit_properties.hallKierAlpha -
0.240\n_pdbe_chem_comp_rdkit_properties.kappa1
2.412\n_pdbe_chem_comp_rdkit_properties.kappa2
3.649\n_pdbe_chem_comp_rdkit_properties.kappa3
1.582\n_pdbe_chem_comp_rdkit_properties.Phi
0.733\n#\nloop_\n_pdbe_chem_comp_external_mappings.comp_id\n_pdbe_chem
_comp_external_mappings.source\n_pdbe_chem_comp_external_mappings.reso
urce\n_pdbe_chem_comp_external_mappings.resource_id\nMAN UniChem
ChEMBL CHEMBL365590\nMAN UniChem 'KEGG LIGAND' C00936\nMAN UniChem
ChEBI 28729\nMAN UniChem ZINC ZINC000003860903\nMAN UniChem fdasrs

W3F28J9G0W\nMAN UniChem SureChEMBL SCHEMBL76882\nMAN UniChem HMDB HMDB0000169\nMAN UniChem 'PubChem TPHARMA' 14916352\nMAN UniChem NMRShiftDB 60078611\nMAN UniChem ACTor 29696-75-1\nMAN UniChem ACTor 7296-15-3\nMAN UniChem Nikkaji J15.393A\nMAN UniChem BindingDb 50467903\nMAN UniChem MetaboLights MTBLC28729\nMAN UniChem BRENDA 102433\nMAN UniChem BRENDA 16456\nMAN UniChem BRENDA 29727\nMAN UniChem BRENDA 47808\nMAN UniChem BRENDA 756\nMAN UniChem BRENDA 993\nMAN UniChem Rhea 28729\nMAN UniChem CCDC ADMANN\nMAN UniChem PubChem
185698\n#\nloop_\n_pdbe_chem_comp_rdkit_conformer.comp_id\n_pdbe_chem_comp_rdkit_conformer.atom_id\n_pdbe_chem_comp_rdkit_conformer.Cartn_x_rdkit\n_pdbe_chem_comp_rdkit_conformer.Cartn_y_rdkit\n_pdbe_chem_comp_rdkit_conformer.Cartn_z_rdkit\n_pdbe_chem_comp_rdkit_conformer.rdkit_method\n_pdbe_chem_comp_rdkit_conformer.rdkit_ordinal\nMAN C1 -1.033 1.513 -0.341 ETKDGv3 1\nMAN C2 -1.809 0.174 -0.462 ETKDGv3 2\nMAN C3 -1.145 -0.938 0.369 ETKDGv3 3\nMAN C4 0.371 -0.974 0.096 ETKDGv3 4\nMAN C5 0.975 0.443 0.267 ETKDGv3 5\nMAN C6 2.483 0.444 -0.020 ETKDGv3 6\nMAN O1 -1.251 2.186 0.879 ETKDGv3 7\nMAN O3 -1.726 -2.175 0.042 ETKDGv3 9\nMAN O4 1.003 -1.904 0.941 ETKDGv3 10\nMAN O5 0.353 1.367 -0.610 ETKDGv3 11\nMAN O6 2.728 0.077 -1.350 ETKDGv3 12\nMAN H1 -1.437 2.184 -1.129 ETKDGv3 13\nMAN H2 -2.848 0.318 -0.083 ETKDGv3 14\nMAN H3 -1.322 -0.725 1.449 ETKDGv3 15\nMAN H4 0.528 -1.305 -0.955 ETKDGv3 16\nMAN H5 0.858 0.774 1.324 ETKDGv3 17\nMAN H61 2.883 1.466 0.162 ETKDGv3 18\nMAN H62 3.004 -0.257 0.670 ETKDGv3 19\nMAN HO1 -1.279 1.524 1.614 ETKDGv3 20\nMAN HO3 -1.516 -2.799 0.786 ETKDGv3 22\nMAN HO4 0.879 -1.594 1.877 ETKDGv3 23\nMAN HO6 3.713 0.097 -1.464 ETKDGv3 24\n#\ndata_MAN-6\n#\n_chem_comp.id MAN\n_chem_comp.name alpha-D-mannopyranose\n_chem_comp.type 'D-saccharide, alpha linking'\n_chem_comp.pdbx_type ATOMS\n_chem_comp.formula 'C6 H12 O6'\n_chem_comp.mon_nstd_parent_comp_id ?\n_chem_comp.pdbx_synonyms 'alpha-D-mannose; D-mannose; mannose'\n_chem_comp.pdbx_formal_charge 0\n_chem_comp.pdbx_initial_date 1999-07-08\n_chem_comp.pdbx_modified_date 2024-09-27\n_chem_comp.pdbx_ambiguous_flag N\n_chem_comp.pdbx_release_status REL\n_chem_comp.pdbx_replaced_by ?\n_chem_comp.pdbx_replaces ?\n_chem_comp.formula_weight 180.156\n_chem_comp.one_letter_code ?\n_chem_comp.three_letter_code MAN\n_chem_comp.pdbx_model_coordinates_details ?\n_chem_comp.pdbx_model_coordinates_missing_flag N\n_chem_comp.pdbx_ideal_coordinates_details ?\n_chem_comp.pdbx_ideal_coordinates_missing_flag N\n_chem_comp.pdbx_model_coordinates_db_code 1GPZ\n_chem_comp.pdbx_subcomponent_list ?\n_chem_comp.pdbx_processing_site RCSB\n_chem_comp.pdbx_pcm Y\n#\nloop_\n_pdbx_chem_comp_synonyms.ordinal\n_pdbx_chem_comp_synonyms.comp_id\n_pdbx_chem_comp_synonyms.name\n_pdbx_chem_comp_synonyms.provenance\n_pdbx_chem_comp_synonyms.type\n1 MAN alpha-D-mannose PDB ?\n2 MAN D-mannose PDB ?\n3 MAN mannose PDB ?\n#\nloop_\n_chem_comp_atom.comp_id\n_chem_comp_atom.atom_id\n_chem_comp_atom.alt_atom_id\n_chem_comp_atom.type_symbol\n_chem_comp_atom.charge\n_chem_comp_atom.pdbx_align\n_chem_comp_atom.pdbx_aromatic_flag\n_chem_comp_atom.pdbx_leaving_atom_flag\n_chem_comp_atom.pdbx_stereo_config\n_chem_comp_atom.pdbx_backbone_atom_flag\n_chem_comp_atom.pdbx

_n_terminal_atom_flag\n_chem_comp_atom.pdbx_c_terminal_atom_flag\n_che
m_comp_atom.model_Cartn_x\n_chem_comp_atom.model_Cartn_y\n_chem_comp_a
tom.model_Cartn_z\n_chem_comp_atom.pdbx_model_Cartn_x_ideal\n_chem_com
p_atom.pdbx_model_Cartn_y_ideal\n_chem_comp_atom.pdbx_model_Cartn_z_id
eal\n_chem_comp_atom.pdbx_component_atom_id\n_chem_comp_atom.pdbx_comp
onent_comp_id\n_chem_comp_atom.pdbx_ordinal\nMAN C1 C1 C 0 1 N N S N N
N 99.738 -29.415 24.222 -1.692 -0.156 -0.316 C1 MAN 1\nMAN C2 C2 C 0 1
N N S N N N 101.239 -29.305 24.564 -0.878 0.091 -1.588 C2 MAN 2\nMAN
C3 C3 C 0 1 N N S N N N 102.016 -28.461 23.551 0.535 -0.467 -1.391 C3
MAN 3\nMAN C4 C4 C 0 1 N N S N N N 101.699 -28.940 22.129 1.126 0.134
-0.111 C4 MAN 4\nMAN C5 C5 C 0 1 N N R N N N 100.197 -28.798 21.881
0.160 -0.117 1.048 C5 MAN 5\nMAN C6 C6 C 0 1 N N N N N N 99.829 -
29.254 20.463 0.757 0.448 2.339 C6 MAN 6\nMAN O1 O1 O 0 1 N Y N N N N
99.000 -28.349 24.713 -1.735 -1.558 -0.046 O1 MAN 7\nMAN O2 O2 O 0 1 N
N N N N N 101.809 -30.606 24.635 -0.808 1.494 -1.845 O2 MAN 8\nMAN O3
O3 O 0 1 N N N N N N 103.406 -28.578 23.812 1.350 -0.113 -2.511 O3 MAN
9\nMAN O4 O4 O 0 1 N N N N N N 102.419 -28.180 21.167 2.384 -0.482
0.170 O4 MAN 10\nMAN O5 O5 O 0 1 N N N N N N 99.454 -29.636 22.812 -
1.087 0.520 0.784 O5 MAN 11\nMAN H1 H1 H 0 1 N N N N N N 99.408 -
30.340 24.750 -2.707 0.216 -0.457 H1 MAN 13\nMAN H2 H2 H 0 1 N N N N N
N 101.314 -28.790 25.550 -1.354 -0.410 -2.430 H2 MAN 14\nMAN H3 H3 H 0
1 N N N N N N 101.716 -27.391 23.643 0.491 -1.552 -1.300 H3 MAN
15\nMAN H4 H4 H 0 1 N N N N N N 102.004 -30.007 22.028 1.267 1.207 -
0.244 H4 MAN 16\nMAN H5 H5 H 0 1 N N N N N N 99.938 -27.722 22.018
0.002 -1.189 1.162 H5 MAN 17\nMAN H61 H61 H 0 1 N N N N N N 100.731 -
29.309 19.811 0.915 1.521 2.226 H61 MAN 18\nMAN H62 H62 H 0 1 N N N N
N N 99.533 -30.329 20.450 1.710 -0.039 2.543 H62 MAN 19\nMAN HO1 HO1 H
0 1 N Y N N N N 98.076 -28.416 24.502 -2.260 -1.672 0.757 HO1 MAN
20\nMAN HO2 HO2 H 0 1 N Y N N N N 102.732 -30.538 24.845 -1.717 1.804
-1.955 HO2 MAN 21\nMAN HO3 HO3 H 0 1 N Y N N N N 103.888 -28.054
23.183 0.934 -0.501 -3.293 HO3 MAN 22\nMAN HO4 HO4 H 0 1 N Y N N N N
102.222 -28.476 20.286 2.958 -0.305 -0.587 HO4 MAN
23\n#\nloop_\n_chem_comp_bond.comp_id\n_chem_comp_bond.atom_id_1\n_che
m_comp_bond.atom_id_2\n_chem_comp_bond.value_order\n_chem_comp_bond.pd
bx_aromatic_flag\n_chem_comp_bond.pdbx_stereo_config\n_chem_comp_bond.
pdbx_ordinal\nMAN C1 C2 SING N N 1\nMAN C1 O1 SING N N 2\nMAN C1 O5
SING N N 3\nMAN C1 H1 SING N N 4\nMAN C2 C3 SING N N 5\nMAN C2 O2 SING
N N 6\nMAN C2 H2 SING N N 7\nMAN C3 C4 SING N N 8\nMAN C3 O3 SING N N
9\nMAN C3 H3 SING N N 10\nMAN C4 C5 SING N N 11\nMAN C4 O4 SING N N
12\nMAN C4 H4 SING N N 13\nMAN C5 C6 SING N N 14\nMAN C5 O5 SING N N
15\nMAN C5 H5 SING N N 16\nMAN C6 H61 SING N N 18\nMAN C6 H62 SING N N
19\nMAN O1 HO1 SING N N 20\nMAN O2 HO2 SING N N 21\nMAN O3 HO3 SING N
N 22\nMAN O4 HO4 SING N N
23\n#\nloop_\n_pdbx_chem_comp_descriptor.comp_id\n_pdbx_chem_comp_desc
riptor.type\n_pdbx_chem_comp_descriptor.program\n_pdbx_chem_comp_descr
iptor.program_version\n_pdbx_chem_comp_descriptor.descriptor\nMAN
SMILES ACDLabs 10.04 'OC1C(O)C(OC(O)C1O)CO'\nMAN SMILES_CANONICAL
CACTVS 3.341 'OC[C@H]1O[C@H](O)[C@@H](O)[C@@H](O)[C@@H]1O'\nMAN SMILES
CACTVS 3.341 'OC[CH]1O[CH](O)[CH](O)[CH](O)[CH]1O'\nMAN
SMILES_CANONICAL 'OpenEye OEToolkits' 1.5.0
'C([C@@H]1[C@H]([C@@H]([C@@H]([C@H](O1)O)O)O)O)O'\nMAN SMILES 'OpenEye
OEToolkits' 1.5.0 'C(C1C(C(C(C(O1)O)O)O)O)O'\nMAN InChI InChI 1.03

'InChI=1S/C6H12O6/c7-1-2-3(8)4(9)5(10)6(11)12-2/h2-11H,1H2/t2-,3-,4+,5+,6+/m1/s1'\nMAN InChIKey InChI 1.03 WQZGKKKJIJFFOK-PQMKYFCFSA-N\n#\nloop_\n_pdbx_chem_comp_identifier.comp_id\n_pdbx_chem_comp_identifier.type\n_pdbx_chem_comp_identifier.program\n_pdbx_chem_comp_identifier.program_version\n_pdbx_chem_comp_identifier.identifier\nMAN 'SYSTEMATIC NAME' ACDLabs 10.04 alpha-D-mannopyranose\nMAN 'SYSTEMATIC NAME' 'OpenEye OEToolkits' 1.5.0 '(2S,3S,4S,5S,6R)-6-(hydroxymethyl)oxane-2,3,4,5-tetrol'\nMAN 'CONDENSED IUPAC CARBOHYDRATE SYMBOL' GMML 1.0 DManpa\nMAN 'COMMON NAME' GMML 1.0 a-D-mannopyranose\nMAN 'IUPAC CARBOHYDRATE SYMBOL' PDB-CARE 1.0 a-D-Manp\nMAN 'SNFG CARBOHYDRATE SYMBOL' GMML 1.0 Man\n#\nloop_\n_pdbx_chem_comp_feature.comp_id\n_pdbx_chem_comp_feature.type\n_pdbx_chem_comp_feature.value\n_pdbx_chem_comp_feature.source\n_pdbx_chem_comp_feature.support\nMAN 'CARBOHYDRATE ISOMER' D PDB ?\nMAN 'CARBOHYDRATE RING' pyranose PDB ?\nMAN 'CARBOHYDRATE ANOMER' alpha PDB ?\nMAN 'CARBOHYDRATE PRIMARY CARBONYL GROUP' aldose PDB ?\n#\nloop_\n_pdbx_chem_comp_audit.comp_id\n_pdbx_chem_comp_audit.action_type\n_pdbx_chem_comp_audit.date\n_pdbx_chem_comp_audit.processing_site\nMAN 'Create component' 1999-07-08 RCSB\nMAN 'Modify descriptor' 2011-06-04 RCSB\nMAN 'Other modification' 2019-08-12 RCSB\nMAN 'Other modification' 2019-12-19 RCSB\nMAN 'Other modification' 2020-07-03 RCSB\nMAN 'Modify name' 2020-07-17 RCSB\nMAN 'Modify synonyms' 2020-07-17 RCSB\nMAN 'Modify PCM' 2024-09-27 PDBE\n#\nloop_\n_pdbx_chem_comp_pcm.pcm_id\n_pdbx_chem_comp_pcm.comp_id\n_pdbx_chem_comp_pcm.modified_residue_id\n_pdbx_chem_comp_pcm.type\n_pdbx_chem_comp_pcm.category\n_pdbx_chem_comp_pcm.position\n_pdbx_chem_comp_pcm.polypeptide_position\n_pdbx_chem_comp_pcm.comp_id_linking_atom\n_pdbx_chem_comp_pcm.modified_residue_id_linking_atom\n_pdbx_chem_comp_pcm.uniprot_specific_ptm_accession\n_pdbx_chem_comp_pcm.uniprot_generic_ptm_accession\n1 MAN SER O-Glycosylation Carbohydrate 'Amino-acid side chain' 'Any position' C1 OG ? ?\n2 MAN THR O-Glycosylation Carbohydrate 'Amino-acid side chain' 'Any position' C1 OG1 ? ?\n3 MAN TRP C-Mannosylation Carbohydrate 'Amino-acid side chain' 'Any position' C1 CD1 ? ?\n4 MAN 3FG None Carbohydrate 'Amino-acid side chain' 'Any position' C1 OD1 ? ?\n5 MAN ARG None Carbohydrate 'Amino-acid side chain' 'Any position' C4 NH2 ? ?\n6 MAN ASN None Carbohydrate 'Amino-acid side chain' 'Any position' C1 ND2 ? ?\n7 MAN D4P None Carbohydrate 'Amino-acid side chain' 'Any position' C1 O4 ? ?\n8 MAN GLU None Carbohydrate 'Amino-acid side chain' 'Any position' C1 OE1 ? ?\n9 MAN HG7 None Carbohydrate 'Amino-acid side chain' 'Any position' O1 C5 ? ?\n10 MAN MDF None Carbohydrate 'Amino-acid side chain' 'Any position' C1 OH2 ? ?\n11 MAN SER None Carbohydrate 'Amino-acid side chain' 'Any position' O1 CB ? ?\n12 MAN THR None Carbohydrate 'Amino-acid side chain' 'Any position' O1 CB ? ?\n#\nloop_\n_pdbe_chem_comp_synonyms.comp_id\n_pdbe_chem_comp_synonyms.name\n_pdbe_chem_comp_synonyms.provenance\n_pdbe_chem_comp_synonyms.type\nMAN alpha-D-mannose wwPDB ?\nMAN D-mannose wwPDB ?\nMAN mannose wwPDB ?\n#\nloop_\n_software.name\n_software.version\n_software.description\nrdkit 2023.09.6 'Core functionality.'\npdbeccdutils 0.8.6 'Wrapper to provide 2D templates and molecular

fragments.'\n#\nloop_\n_pdbe_chem_comp_atom_depiction.comp_id\n_pdbe_chem_comp_atom_depiction.atom_id\n_pdbe_chem_comp_atom_depiction.element\n_pdbe_chem_comp_atom_depiction.model_Cartn_x\n_pdbe_chem_comp_atom_depiction.model_Cartn_y\n_pdbe_chem_comp_atom_depiction.pdbx_ordinal\nMAN C1 C 7.702 0.750 1\nMAN C2 C 7.702 -0.750 2\nMAN C3 C 6.404 -1.500 3\nMAN C4 C 5.104 -0.750 4\nMAN C5 C 5.104 0.750 5\nMAN C6 C 3.805 1.500 6\nMAN O1 O 9.002 1.500 7\nMAN O2 O 9.002 -1.500 8\nMAN O3 O 6.404 -3.000 9\nMAN O4 O 3.805 -1.500 10\nMAN O5 O 6.404 1.500 11\n#\nloop_\n_pdbe_chem_comp_bond_depiction.comp_id\n_pdbe_chem_comp_bond_depiction.atom_id_1\n_pdbe_chem_comp_bond_depiction.atom_id_2\n_pdbe_chem_comp_bond_depiction.value_order\n_pdbe_chem_comp_bond_depiction.bond_dir\n_pdbe_chem_comp_bond_depiction.pdbx_ordinal\nMAN C1 C2 SINGLE NONE 1\nMAN C1 O1 SINGLE BEGINDASH 2\nMAN C1 O5 SINGLE NONE 3\nMAN C2 C3 SINGLE NONE 4\nMAN C2 O2 SINGLE BEGINWEDGE 5\nMAN C3 C4 SINGLE NONE 6\nMAN C3 O3 SINGLE BEGINWEDGE 7\nMAN C4 C5 SINGLE NONE 8\nMAN C4 O4 SINGLE BEGINDASH 9\nMAN C5 C6 SINGLE BEGINWEDGE 10\nMAN C5 O5 SINGLE NONE
11\n#\nloop_\n_pdbe_chem_comp_substructure.comp_id\n_pdbe_chem_comp_substructure.substructure_name\n_pdbe_chem_comp_substructure.id\n_pdbe_chem_comp_substructure.substructure_type\n_pdbe_chem_comp_substructure.substructure_smiles\n_pdbe_chem_comp_substructure.substructure_inchis\n_pdbe_chem_comp_substructure.substructure_inchikeys\nMAN MurckoScaffold S1 scaffold C1CCOCC1 InChI=1S/C5H10O/c1-2-4-6-5-3-1/h1-5H2 DHXVGJBLRPWPCS-UHFFFAOYSA-N\nMAN pyranose F1 fragment OC1CCCCO1 InChI=1S/C5H10O2/c6-5-3-1-2-4-7-5/h5-6H,1-4H2 CELWCAITJAEQNL-UHFFFAOYSA-
N\n#\nloop_\n_pdbe_chem_comp_substructure_mapping.comp_id\n_pdbe_chem_comp_substructure_mapping.atom_id\n_pdbe_chem_comp_substructure_mapping.substructure_id\n_pdbe_chem_comp_substructure_mapping.substructure_ordinal\nMAN C1 S1 1\nMAN C2 S1 1\nMAN C3 S1 1\nMAN C4 S1 1\nMAN C5 S1 1\nMAN O5 S1 1\nMAN C4 F1 1\nMAN C5 F1 1\nMAN O5 F1 1\nMAN C1 F1 1\nMAN C2 F1 1\nMAN C3 F1 1\nMAN O1 F1
1\n#\n_pdbe_chem_comp_rdkit_properties.comp_id
MAN\n_pdbe_chem_comp_rdkit_properties.exactmw
180.063\n_pdbe_chem_comp_rdkit_properties.amw
180.156\n_pdbe_chem_comp_rdkit_properties.lipinskiHBA
6\n_pdbe_chem_comp_rdkit_properties.lipinskiHBD
5\n_pdbe_chem_comp_rdkit_properties.NumRotatableBonds
6\n_pdbe_chem_comp_rdkit_properties.NumHBD
5\n_pdbe_chem_comp_rdkit_properties.NumHBA
6\n_pdbe_chem_comp_rdkit_properties.NumHeavyAtoms
12\n_pdbe_chem_comp_rdkit_properties.NumAtoms
24\n_pdbe_chem_comp_rdkit_properties.NumHeteroatoms
6\n_pdbe_chem_comp_rdkit_properties.NumAmideBonds
0\n_pdbe_chem_comp_rdkit_properties.FractionCSP3
1\n_pdbe_chem_comp_rdkit_properties.NumRings
1\n_pdbe_chem_comp_rdkit_properties.NumAromaticRings
0\n_pdbe_chem_comp_rdkit_properties.NumAliphaticRings
1\n_pdbe_chem_comp_rdkit_properties.NumSaturatedRings
1\n_pdbe_chem_comp_rdkit_properties.NumHeterocycles
1\n_pdbe_chem_comp_rdkit_properties.NumAromaticHeterocycles
0\n_pdbe_chem_comp_rdkit_properties.NumSaturatedHeterocycles

1\n_pdbe_chem_comp_rdkit_properties.NumAliphaticHeterocycles
1\n_pdbe_chem_comp_rdkit_properties.NumSpiroAtoms
0\n_pdbe_chem_comp_rdkit_properties.NumBridgeheadAtoms
0\n_pdbe_chem_comp_rdkit_properties.NumAtomStereoCenters
5\n_pdbe_chem_comp_rdkit_properties.NumUnspecifiedAtomStereoCenters
0\n_pdbe_chem_comp_rdkit_properties.labuteASA
85.936\n_pdbe_chem_comp_rdkit_properties.tpsa
110.380\n_pdbe_chem_comp_rdkit_properties.CrippenClogP -
3.221\n_pdbe_chem_comp_rdkit_properties.CrippenMR
35.986\n_pdbe_chem_comp_rdkit_properties.chi0v
5.449\n_pdbe_chem_comp_rdkit_properties.chi1v
2.679\n_pdbe_chem_comp_rdkit_properties.chi2v
1.092\n_pdbe_chem_comp_rdkit_properties.chi3v
1.092\n_pdbe_chem_comp_rdkit_properties.chi4v
0.552\n_pdbe_chem_comp_rdkit_properties.chi0n
17.449\n_pdbe_chem_comp_rdkit_properties.chi1n
8.220\n_pdbe_chem_comp_rdkit_properties.chi2n
1.092\n_pdbe_chem_comp_rdkit_properties.chi3n
1.092\n_pdbe_chem_comp_rdkit_properties.chi4n
0.552\n_pdbe_chem_comp_rdkit_properties.hallKierAlpha -
0.240\n_pdbe_chem_comp_rdkit_properties.kappa1
2.412\n_pdbe_chem_comp_rdkit_properties.kappa2
3.649\n_pdbe_chem_comp_rdkit_properties.kappa3
1.582\n_pdbe_chem_comp_rdkit_properties.Phi
0.733\n#\nloop_\n_pdbe_chem_comp_external_mappings.comp_id\n_pdbe_chem_comp_external_mappings.source\n_pdbe_chem_comp_external_mappings.resource\n_pdbe_chem_comp_external_mappings.resource_id\nMAN UniChem ChEMBL CHEMBL365590\nMAN UniChem 'KEGG LIGAND' C00936\nMAN UniChem ChEBI 28729\nMAN UniChem ZINC ZINC000003860903\nMAN UniChem fdasrs W3F28J9G0W\nMAN UniChem SureChEMBL SCHEMBL76882\nMAN UniChem HMDB HMDB0000169\nMAN UniChem 'PubChem TPHARMA' 14916352\nMAN UniChem NMRShiftDB 60078611\nMAN UniChem ACTor 29696-75-1\nMAN UniChem ACTor 7296-15-3\nMAN UniChem Nikkaji J15.393A\nMAN UniChem BindingDb 50467903\nMAN UniChem MetaboLights MTBLC28729\nMAN UniChem BRENDA 102433\nMAN UniChem BRENDA 16456\nMAN UniChem BRENDA 29727\nMAN UniChem BRENDA 47808\nMAN UniChem BRENDA 756\nMAN UniChem BRENDA 993\nMAN UniChem Rhea 28729\nMAN UniChem CCDC ADMANN\nMAN UniChem PubChem
185698\n#\nloop_\n_pdbe_chem_comp_rdkit_conformer.comp_id\n_pdbe_chem_comp_rdkit_conformer.atom_id\n_pdbe_chem_comp_rdkit_conformer.Cartn_x_rdkit\n_pdbe_chem_comp_rdkit_conformer.Cartn_y_rdkit\n_pdbe_chem_comp_rdkit_conformer.Cartn_z_rdkit\n_pdbe_chem_comp_rdkit_conformer.rdkit_method\n_pdbe_chem_comp_rdkit_conformer.rdkit_ordinal\nMAN C1 -1.033 1.513 -0.341 ETKDGv3 1\nMAN C2 -1.809 0.174 -0.462 ETKDGv3 2\nMAN C3 -1.145 -0.938 0.369 ETKDGv3 3\nMAN C4 0.371 -0.974 0.096 ETKDGv3 4\nMAN C5 0.975 0.443 0.267 ETKDGv3 5\nMAN C6 2.483 0.444 -0.020 ETKDGv3 6\nMAN O1 -1.251 2.186 0.879 ETKDGv3 7\nMAN O2 -1.853 -0.220 -1.812 ETKDGv3 8\nMAN O3 -1.726 -2.175 0.042 ETKDGv3 9\nMAN O4 1.003 -1.904 0.941 ETKDGv3 10\nMAN O5 0.353 1.367 -0.610 ETKDGv3 11\nMAN H1 -1.437 2.184 -1.129 ETKDGv3 13\nMAN H2 -2.848 0.318 -0.083 ETKDGv3 14\nMAN H3 -1.322 -0.725 1.449 ETKDGv3 15\nMAN H4 0.528 -1.305 -0.955 ETKDGv3 16\nMAN H5 0.858 0.774 1.324 ETKDGv3 17\nMAN H61 2.883 1.466 0.162

ETKDGv3 18\nMAN H62 3.004 -0.257 0.670 ETKDGv3 19\nMAN HO1 -1.279 1.524 1.614 ETKDGv3 20\nMAN HO2 -2.558 0.327 -2.249 ETKDGv3 21\nMAN HO3 -1.516 -2.799 0.786 ETKDGv3 22\nMAN HO4 0.879 -1.594 1.877 ETKDGv3 23\n#\ndata_SER-2\n#\n_chem_comp.id SER\n_chem_comp.name SERINE\n_chem_comp.type 'L-PEPTIDE LINKING'\n_chem_comp.pdbx_type ATOMP\n_chem_comp.formula 'C3 H7 N O3'\n_chem_comp.mon_nstd_parent_comp_id ?\n_chem_comp.pdbx_synonyms ?\n_chem_comp.pdbx_formal_charge 0\n_chem_comp.pdbx_initial_date 1999-07-08\n_chem_comp.pdbx_modified_date 2024-09-27\n_chem_comp.pdbx_ambiguous_flag N\n_chem_comp.pdbx_release_status REL\n_chem_comp.pdbx_replaced_by ?\n_chem_comp.pdbx_replaces SEG\n_chem_comp.formula_weight 105.093\n_chem_comp.one_letter_code S\n_chem_comp.three_letter_code SER\n_chem_comp.pdbx_model_coordinates_details ?\n_chem_comp.pdbx_model_coordinates_missing_flag N\n_chem_comp.pdbx_ideal_coordinates_details ?\n_chem_comp.pdbx_ideal_coordinates_missing_flag N\n_chem_comp.pdbx_model_coordinates_db_code ?\n_chem_comp.pdbx_subcomponent_list ?\n_chem_comp.pdbx_processing_site RCSB\n_chem_comp.pdbx_pcm Y\n#\nloop_\n_chem_comp_atom.comp_id\n_chem_comp_atom.atom_id\n_chem_comp_atom.alt_atom_id\n_chem_comp_atom.type_symbol\n_chem_comp_atom.charge\n_chem_comp_atom.pdbx_align\n_chem_comp_atom.pdbx_aromatic_flag\n_chem_comp_atom.pdbx_leaving_atom_flag\n_chem_comp_atom.pdbx_stereo_config\n_chem_comp_atom.pdbx_backbone_atom_flag\n_chem_comp_atom.pdbx_n_terminal_atom_flag\n_chem_comp_atom.pdbx_c_terminal_atom_flag\n_chem_comp_atom.model_Cartn_x\n_chem_comp_atom.model_Cartn_y\n_chem_comp_atom.model_Cartn_z\n_chem_comp_atom.pdbx_model_Cartn_x_ideal\n_chem_comp_atom.pdbx_model_Cartn_y_ideal\n_chem_comp_atom.pdbx_model_Cartn_z_ideal\n_chem_comp_atom.pdbx_component_atom_id\n_chem_comp_atom.pdbx_component_comp_id\n_chem_comp_atom.pdbx_ordinal\nSER N N N 0 1 N N N Y Y N 88.198 -7.658 -9.979 1.525 0.493 -0.608 N SER 1\nSER CA CA C 0 1 N N S Y N N 87.782 -7.276 -11.358 0.100 0.469 -0.252 CA SER 2\nSER C C C 0 1 N N N Y N Y 88.571 -6.062 -11.818 -0.053 0.004 1.173 C SER 3\nSER O O O 0 1 N N N Y N Y 89.008 -5.296 -10.944 0.751 -0.760 1.649 O SER 4\nSER CB CB C 0 1 N N N N N N 86.286 -6.966 -11.391 -0.642 -0.489 -1.184 CB SER 5\nSER OG OG O 0 1 N N N N N N 85.543 -8.096 -10.989 -0.496 -0.049 -2.535 OG SER 6\nSER H H H 0 1 N N N Y Y N 87.668 -8.473 -9.670 1.867 -0.449 -0.499 H SER 8\nSER H2 HN2 H 0 1 N Y N Y Y N 88.118 -6.879 -9.325 1.574 0.707 -1.593 H2 SER 9\nSER HA HA H 0 1 N N N Y N N 87.988 -8.129 -12.045 -0.316 1.471 -0.354 HA SER 10\nSER HB2 1HB H 0 1 N N N N N N 86.034 -6.065 -10.783 -0.225 -1.491 -1.081 HB2 SER 11\nSER HB3 2HB H 0 1 N N N N N N 85.961 -6.588 -12.388 -1.699 -0.507 -0.920 HB3 SER 12\nSER HG HG H 0 1 N N N N N N 84.613 -7.903 -11.009 -0.978 -0.679 -3.088 HG SER 13\n#\nloop_\n_chem_comp_bond.comp_id\n_chem_comp_bond.atom_id_1\n_chem_comp_bond.atom_id_2\n_chem_comp_bond.value_order\n_chem_comp_bond.pdbx_aromatic_flag\n_chem_comp_bond.pdbx_stereo_config\n_chem_comp_bond.pdbx_ordinal\nSER N CA SING N N 1\nSER N H SING N N 2\nSER N H2 SING N N 3\nSER CA C SING N N 4\nSER CA CB SING N N 5\nSER CA HA SING N N 6\nSER C O DOUB N N 7\nSER CB OG SING N N 9\nSER CB HB2 SING N N 10\nSER CB HB3 SING N N 11\nSER OG HG SING N N

12\n#\nloop_\n_pdbx_chem_comp_descriptor.comp_id\n_pdbx_chem_comp_descriptor.type\n_pdbx_chem_comp_descriptor.program\n_pdbx_chem_comp_descriptor.program_version\n_pdbx_chem_comp_descriptor.descriptor\nSER SMILES ACDLabs 10.04 'O=C(O)C(N)CO'\nSER SMILES_CANONICAL CACTVS 3.341 'N[C@@H](CO)C(O)=O'\nSER SMILES CACTVS 3.341 'N[CH](CO)C(O)=O'\nSER SMILES_CANONICAL 'OpenEye OEToolkits' 1.5.0 'C([C@@H](C(=O)O)N)O'\nSER SMILES 'OpenEye OEToolkits' 1.5.0 'C(C(C(=O)O)N)O'\nSER InChI InChI 1.03 'InChI=1S/C3H7NO3/c4-2(1-5)3(6)7/h2,5H,1,4H2,(H,6,7)/t2-/m0/s1'\nSER InChIKey InChI 1.03 MTCFGRXMJLQNBG-REOHCLBHSA-N\n#\nloop_\n_pdbx_chem_comp_identifier.comp_id\n_pdbx_chem_comp_identifier.type\n_pdbx_chem_comp_identifier.program\n_pdbx_chem_comp_identifier.program_version\n_pdbx_chem_comp_identifier.identifier\nSER 'SYSTEMATIC NAME' ACDLabs 10.04 L-serine\nSER 'SYSTEMATIC NAME' 'OpenEye OEToolkits' 1.5.0 '(2S)-2-amino-3-hydroxy-propanoic acid'\n#\nloop_\n_pdbx_chem_comp_audit.comp_id\n_pdbx_chem_comp_audit.action_type\n_pdbx_chem_comp_audit.date\n_pdbx_chem_comp_audit.processing_site\nSER 'Create component' 1999-07-08 RCSB\nSER 'Modify descriptor' 2011-06-04 RCSB\nSER 'Modify backbone' 2023-11-03 PDBE\nSER 'Modify PCM' 2024-09-27 PDBE\n#\n_pdbx_chem_comp_pcm.pcm_id 1\n_pdbx_chem_comp_pcm.comp_id SER\n_pdbx_chem_comp_pcm.modified_residue_id LLP\n_pdbx_chem_comp_pcm.type Serylation\n_pdbx_chem_comp_pcm.category 'Amino acid'\n_pdbx_chem_comp_pcm.position 'Amino-acid side chain'\n_pdbx_chem_comp_pcm.polypeptide_position 'Any position'\n_pdbx_chem_comp_pcm.comp_id_linking_atom N\n_pdbx_chem_comp_pcm.modified_residue_id_linking_atom 'C4''\n_pdbx_chem_comp_pcm.uniprot_specific_ptm_accession ?\n_pdbx_chem_comp_pcm.uniprot_generic_ptm_accession ?\n#\n_pdbe_chem_comp_drugbank_details.comp_id SER\n_pdbe_chem_comp_drugbank_details.drugbank_id DB00133\n_pdbe_chem_comp_drugbank_details.type 'small molecule'\n_pdbe_chem_comp_drugbank_details.name Serine\n_pdbe_chem_comp_drugbank_details.description\n'A non-essential amino acid occurring in natural form as the L-isomer. It is synthesized from glycine or threonine. It is involved in the biosynthesis of purines; pyrimidines; and other amino acids.'\n_pdbe_chem_comp_drugbank_details.cas_number 56-45-1\n_pdbe_chem_comp_drugbank_details.mechanism_of_action\n'L-Serine plays a role in cell growth and development (cellular proliferation). The conversion of L-serine to glycine by serine hydroxymethyltransferase results in the formation of the one-carbon units necessary for the synthesis of the purine bases, adenine and guanine. These bases when linked to the phosphate ester of pentose sugars are essential components of DNA and RNA and the end products of energy producing metabolic pathways, ATP and GTP. In addition, L-serine conversion to glycine via this same enzyme provides the one-carbon units necessary for production of the pyrimidine nucleotide, deoxythymidine monophosphate, also an essential component of DNA.'\n#\nloop_\n_pdbe_chem_comp_synonyms.comp_id\n_pdbe_chem_comp_synonyms.name\n_pdbe_chem_comp_synonyms.provenance\n_pdbe_chem_comp_synonyms.type\nSER '(S)-2-Amino-3-hydroxypropanoic acid' DrugBank ?\nSER (S)-Serine DrugBank ?\nSER 'alpha-Amino-beta-hydroxypropionic acid' DrugBank ?\nSER beta-Hydroxyalanine DrugBank ?\nSER L-Serine

DrugBank ?\nSER Ser DrugBank ?\nSER Serine DrugBank ?\nSER Serinum DrugBank ?\n#\n_pdbe_chem_comp_drugbank_classification.comp_id SER\n_pdbe_chem_comp_drugbank_classification.drugbank_id DB00133\n_pdbe_chem_comp_drugbank_classification.parent 'Serine and derivatives'\n_pdbe_chem_comp_drugbank_classification.kingdom 'Organic compounds'\n_pdbe_chem_comp_drugbank_classification.class 'Carboxylic acids and derivatives'\n_pdbe_chem_comp_drugbank_classification.superclass 'Organic acids and derivatives'\n_pdbe_chem_comp_drugbank_classification.description\n'This compound belongs to the class of organic compounds known as serine and derivatives. These are compounds containing serine or a derivative thereof resulting from reaction of serine at the amino group or the carboxy group, or from the replacement of any hydrogen of glycine by a heteroatom.'\n#\nloop_\n_pdbe_chem_comp_drugbank_targets.comp_id\n_pdbe_chem_comp_drugbank_targets.drugbank_id\n_pdbe_chem_comp_drugbank_targets.name\n_pdbe_chem_comp_drugbank_targets.organism\n_pdbe_chem_comp_drugbank_targets.uniprot_id\n_pdbe_chem_comp_drugbank_targets.pharmacologically_active\n_pdbe_chem_comp_drugbank_targets.ordinal\nSER DB00133 'Cystathionine beta-synthase' Humans P35520 unknown 1\nSER DB00133 'L-serine dehydratase/L-threonine deaminase' Humans P20132 unknown 2\nSER DB00133 'Serine racemase' Humans Q9GZT4 unknown 3\nSER DB00133 'Serine palmitoyltransferase 2' Humans O15270 unknown 4\nSER DB00133 'Serine palmitoyltransferase 1' Humans O15269 unknown 5\nSER DB00133 'Serine--tRNA ligase, cytoplasmic' Humans P49591 unknown 6\nSER DB00133 'Alanine--glyoxylate aminotransferase' Humans P21549 unknown 7\n#\nloop_\n_software.name\n_software.version\n_software.description\nrdkit 2023.09.6 'Core functionality.'\npdbeccdutils 0.8.6 'Wrapper to provide 2D templates and molecular fragments.'\n#\nloop_\n_pdbe_chem_comp_atom_depiction.comp_id\n_pdbe_chem_comp_atom_depiction.atom_id\n_pdbe_chem_comp_atom_depiction.element\n_pdbe_chem_comp_atom_depiction.model_Cartn_x\n_pdbe_chem_comp_atom_depiction.model_Cartn_y\n_pdbe_chem_comp_atom_depiction.pdbx_ordinal\nSER N N 3.805 1.215 1\nSER CA C 5.104 0.465 2\nSER C C 6.404 1.215 3\nSER O O 7.702 0.465 4\nSER CB C 5.104 -1.035 5\nSER OG O 3.805 -1.785 6\n#\nloop_\n_pdbe_chem_comp_bond_depiction.comp_id\n_pdbe_chem_comp_bond_depiction.atom_id_1\n_pdbe_chem_comp_bond_depiction.atom_id_2\n_pdbe_chem_comp_bond_depiction.value_order\n_pdbe_chem_comp_bond_depiction.bond_dir\n_pdbe_chem_comp_bond_depiction.pdbx_ordinal\nSER CA N SINGLE BEGINDASH 1\nSER CA C SINGLE NONE 2\nSER CA CB SINGLE NONE 3\nSER C O DOUBLE NONE 4\nSER CB OG SINGLE NONE 6\n#\n_pdbe_chem_comp_substructure.comp_id SER\n_pdbe_chem_comp_substructure.substructure_name peptide\n_pdbe_chem_comp_substructure.id F1\n_pdbe_chem_comp_substructure.substructure_type fragment\n_pdbe_chem_comp_substructure.substructure_smiles NCC=O\n_pdbe_chem_comp_substructure.substructure_inchis InChI=1S/C2H5NO/c3-1-2-4/h2H,1,3H2\n_pdbe_chem_comp_substructure.substructure_inchikeys LYIIBVSRGJSHAV-UHFFFAOYSA-

N\n#\nloop_\n_pdbe_chem_comp_substructure_mapping.comp_id\n_pdbe_chem_
comp_substructure_mapping.atom_id\n_pdbe_chem_comp_substructure_mappin
g.substructure_id\n_pdbe_chem_comp_substructure_mapping.substructure_o
rdinal\nSER O F1 1\nSER C F1 1\nSER CA F1 1\nSER N F1
1\n#\n_pdbe_chem_comp_rdkit_properties.comp_id
SER\n_pdbe_chem_comp_rdkit_properties.exactmw
105.043\n_pdbe_chem_comp_rdkit_properties.amw
105.093\n_pdbe_chem_comp_rdkit_properties.lipinskiHBA
4\n_pdbe_chem_comp_rdkit_properties.lipinskiHBD
4\n_pdbe_chem_comp_rdkit_properties.NumRotatableBonds
4\n_pdbe_chem_comp_rdkit_properties.NumHBD
3\n_pdbe_chem_comp_rdkit_properties.NumHBA
4\n_pdbe_chem_comp_rdkit_properties.NumHeavyAtoms
7\n_pdbe_chem_comp_rdkit_properties.NumAtoms
14\n_pdbe_chem_comp_rdkit_properties.NumHeteroatoms
4\n_pdbe_chem_comp_rdkit_properties.NumAmideBonds
0\n_pdbe_chem_comp_rdkit_properties.FractionCSP3
0.667\n_pdbe_chem_comp_rdkit_properties.NumRings
0\n_pdbe_chem_comp_rdkit_properties.NumAromaticRings
0\n_pdbe_chem_comp_rdkit_properties.NumAliphaticRings
0\n_pdbe_chem_comp_rdkit_properties.NumSaturatedRings
0\n_pdbe_chem_comp_rdkit_properties.NumHeterocycles
0\n_pdbe_chem_comp_rdkit_properties.NumAromaticHeterocycles
0\n_pdbe_chem_comp_rdkit_properties.NumSaturatedHeterocycles
0\n_pdbe_chem_comp_rdkit_properties.NumAliphaticHeterocycles
0\n_pdbe_chem_comp_rdkit_properties.NumSpiroAtoms
0\n_pdbe_chem_comp_rdkit_properties.NumBridgeheadAtoms
0\n_pdbe_chem_comp_rdkit_properties.NumAtomStereoCenters
1\n_pdbe_chem_comp_rdkit_properties.NumUnspecifiedAtomStereoCenters
0\n_pdbe_chem_comp_rdkit_properties.labuteASA
50.683\n_pdbe_chem_comp_rdkit_properties.tpsa
83.550\n_pdbe_chem_comp_rdkit_properties.CrippenClogP -
1.609\n_pdbe_chem_comp_rdkit_properties.CrippenMR
22.697\n_pdbe_chem_comp_rdkit_properties.chi0v
3.172\n_pdbe_chem_comp_rdkit_properties.chi1v
1.336\n_pdbe_chem_comp_rdkit_properties.chi2v
0.290\n_pdbe_chem_comp_rdkit_properties.chi3v
0.290\n_pdbe_chem_comp_rdkit_properties.chi4v
0.042\n_pdbe_chem_comp_rdkit_properties.chi0n
10.172\n_pdbe_chem_comp_rdkit_properties.chi1n
4.547\n_pdbe_chem_comp_rdkit_properties.chi2n
0.290\n_pdbe_chem_comp_rdkit_properties.chi3n
0.290\n_pdbe_chem_comp_rdkit_properties.chi4n
0.042\n_pdbe_chem_comp_rdkit_properties.hallKierAlpha -
0.610\n_pdbe_chem_comp_rdkit_properties.kappa1
1.209\n_pdbe_chem_comp_rdkit_properties.kappa2
2.544\n_pdbe_chem_comp_rdkit_properties.kappa3
2.132\n_pdbe_chem_comp_rdkit_properties.Phi
0.439\n#\nloop_\n_pdbe_chem_comp_external_mappings.comp_id\n_pdbe_chem
_comp_external_mappings.source\n_pdbe_chem_comp_external_mappings.reso
urce\n_pdbe_chem_comp_external_mappings.resource_id\nSER UniChem
ChEMBL CHEMBL11298\nSER UniChem DrugBank DB00133\nSER UniChem 'Guide

to Pharmacology' 726\nSER UniChem 'KEGG LIGAND' C00065\nSER UniChem ChEBI 17115\nSER UniChem ChEBI 33384\nSER UniChem ZINC ZINC000000895034\nSER UniChem fdasrs 452VLY9402\nSER UniChem PharmGKB PA451330\nSER UniChem HMDB HMDB0000187\nSER UniChem NMRShiftDB 60006048\nSER UniChem Recon 'ser_L'\nSER UniChem BindingDb 50357212\nSER UniChem 'EPA CompTox Dashboard' DTXSID60883230\nSER UniChem DrugCentral 4127\nSER UniChem MetaboLights MTBLC17115\nSER UniChem MetaboLights MTBLC33384\nSER UniChem BRENDA 145040\nSER UniChem BRENDA 145952\nSER UniChem BRENDA 262\nSER UniChem BRENDA 35658\nSER UniChem BRENDA 870\nSER UniChem BRENDA 930\nSER UniChem BRENDA 95\nSER UniChem Rhea 33384\nSER UniChem ChemicalBook CB5673304\nSER UniChem DailyMed SERINE\nSER UniChem ClinicalTrials L-SERINE\nSER UniChem ClinicalTrials SERINE\nSER UniChem rxnorm SERINE\nSER UniChem MedChemExpress HY-N0650\nSER UniChem 'Probes And Drugs' PD010212\nSER UniChem CCDC LSERIN\nSER UniChem Nikkaji J1.195I\nSER UniChem eMolecules 514205\nSER UniChem SureChEMBL SCHEMBL1775\nSER UniChem 'PubChem TPHARMA' 15119722\nSER UniChem 'PubChem TPHARMA' 16532479\nSER UniChem PubChem 5951\nSER UniChem PubChem 6857581\nSER UniChem Mcule MCULE-5604658333\nSER UniChem Mcule MCULE-6118038121\nSER UniChem ACTor 25821-52-7\n#\nloop_\n_pdbe_chem_comp_rdkit_conformer.comp_id\n_pdbe_chem_comp_rdkit_conformer.atom_id\n_pdbe_chem_comp_rdkit_conformer.Cartn_x_rdkit\n_pdbe_chem_comp_rdkit_conformer.Cartn_y_rdkit\n_pdbe_chem_comp_rdkit_conformer.Cartn_z_rdkit\n_pdbe_chem_comp_rdkit_conformer.rdkit_method\n_pdbe_chem_comp_rdkit_conformer.rdkit_ordinal\nSER N -0.454 1.499 0.875 ETKDGv3 1\nSER CA -0.308 0.145 0.337 ETKDGv3 2\nSER C 1.135 -0.288 0.359 ETKDGv3 3\nSER O 2.007 0.361 -0.280 ETKDGv3 4\nSER CB -0.885 0.080 -1.085 ETKDGv3 5\nSER OG -0.821 -1.228 -1.589 ETKDGv3 6\nSER H 0.139 2.162 0.324 ETKDGv3 8\nSER H2 -0.101 1.508 1.860 ETKDGv3 9\nSER HA -0.897 -0.547 0.979 ETKDGv3 10\nSER HB2 -0.310 0.750 -1.759 ETKDGv3 11\nSER HB3 -1.941 0.436 -1.081 ETKDGv3 12\nSER HG -1.541 -1.741 -1.138 ETKDGv3 13\n#\ndata_MAN-1-2\n#\n_chem_comp.id MAN\n_chem_comp.name alpha-D-mannopyranose\n_chem_comp.type 'D-saccharide, alpha linking'\n_chem_comp.pdbx_type ATOMS\n_chem_comp.formula 'C6 H12 O6'\n_chem_comp.mon_nstd_parent_comp_id ?\n_chem_comp.pdbx_synonyms 'alpha-D-mannose; D-mannose; mannose'\n_chem_comp.pdbx_formal_charge 0\n_chem_comp.pdbx_initial_date 1999-07-08\n_chem_comp.pdbx_modified_date 2024-09-27\n_chem_comp.pdbx_ambiguous_flag N\n_chem_comp.pdbx_release_status REL\n_chem_comp.pdbx_replaced_by ?\n_chem_comp.pdbx_replaces ?\n_chem_comp.formula_weight 180.156\n_chem_comp.one_letter_code ?\n_chem_comp.three_letter_code MAN\n_chem_comp.pdbx_model_coordinates_details ?\n_chem_comp.pdbx_model_coordinates_missing_flag N\n_chem_comp.pdbx_ideal_coordinates_details ?\n_chem_comp.pdbx_ideal_coordinates_missing_flag N\n_chem_comp.pdbx_model_coordinates_db_code 1GPZ\n_chem_comp.pdbx_subcomponent_list ?\n_chem_comp.pdbx_processing_site RCSB\n_chem_comp.pdbx_pcm Y\n#\nloop_\n_pdbx_chem_comp_synonyms.ordinal\n_pdbx_chem_comp_synonyms.comp_id\n_pdbx_chem_comp_synonyms.name\n_pdbx_chem_comp_synonyms.provenance\n_pdbx_chem_comp_synonyms.type\n1 MAN alpha-D-mannose PDB ?\n2

MAN D-mannose PDB ?\n3 MAN mannose
PDB ?\n#\nloop_\n_chem_comp_atom.comp_id\n_chem_comp_atom.atom_id\n_chem_comp_atom.alt_atom_id\n_chem_comp_atom.type_symbol\n_chem_comp_atom.charge\n_chem_comp_atom.pdbx_align\n_chem_comp_atom.pdbx_aromatic_flag\n_chem_comp_atom.pdbx_leaving_atom_flag\n_chem_comp_atom.pdbx_stereo_config\n_chem_comp_atom.pdbx_backbone_atom_flag\n_chem_comp_atom.pdbx_n_terminal_atom_flag\n_chem_comp_atom.pdbx_c_terminal_atom_flag\n_chem_comp_atom.model_Cartn_x\n_chem_comp_atom.model_Cartn_y\n_chem_comp_atom.model_Cartn_z\n_chem_comp_atom.pdbx_model_Cartn_x_ideal\n_chem_comp_atom.pdbx_model_Cartn_y_ideal\n_chem_comp_atom.pdbx_model_Cartn_z_ideal\n_chem_comp_atom.pdbx_component_atom_id\n_chem_comp_atom.pdbx_component_comp_id\n_chem_comp_atom.pdbx_ordinal\nMAN C1 C1 C 0 1 N N S N N N 99.738 -29.415 24.222 -1.692 -0.156 -0.316 C1 MAN 1\nMAN C2 C2 C 0 1 N N S N N N 101.239 -29.305 24.564 -0.878 0.091 -1.588 C2 MAN 2\nMAN C3 C3 C 0 1 N N S N N N 102.016 -28.461 23.551 0.535 -0.467 -1.391 C3 MAN 3\nMAN C4 C4 C 0 1 N N S N N N 101.699 -28.940 22.129 1.126 0.134 -0.111 C4 MAN 4\nMAN C5 C5 C 0 1 N N R N N N 100.197 -28.798 21.881 0.160 -0.117 1.048 C5 MAN 5\nMAN C6 C6 C 0 1 N N N N N N 99.829 -29.254 20.463 0.757 0.448 2.339 C6 MAN 6\nMAN O3 O3 O 0 1 N N N N N N 103.406 -28.578 23.812 1.350 -0.113 -2.511 O3 MAN 9\nMAN O4 O4 O 0 1 N N N N N N 102.419 -28.180 21.167 2.384 -0.482 0.170 O4 MAN 10\nMAN O5 O5 O 0 1 N N N N N N 99.454 -29.636 22.812 -1.087 0.520 0.784 O5 MAN 11\nMAN O6 O6 O 0 1 N N N N N N 98.821 -28.437 19.876 -0.142 0.211 3.423 O6 MAN 12\nMAN H1 H1 H 0 1 N N N N N N 99.408 -30.340 24.750 -2.707 0.216 -0.457 H1 MAN 13\nMAN H2 H2 H 0 1 N N N N N N 101.314 -28.790 25.550 -1.354 -0.410 -2.430 H2 MAN 14\nMAN H3 H3 H 0 1 N N N N N N 101.716 -27.391 23.643 0.491 -1.552 -1.300 H3 MAN 15\nMAN H4 H4 H 0 1 N N N N N N 102.004 -30.007 22.028 1.267 1.207 -0.244 H4 MAN 16\nMAN H5 H5 H 0 1 N N N N N N 99.938 -27.722 22.018 0.002 -1.189 1.162 H5 MAN 17\nMAN H61 H61 H 0 1 N N N N N N 100.731 -29.309 19.811 0.915 1.521 2.226 H61 MAN 18\nMAN H62 H62 H 0 1 N N N N N N 99.533 -30.329 20.450 1.710 -0.039 2.543 H62 MAN 19\nMAN HO3 HO3 H 0 1 N Y N N N N 103.888 -28.054 23.183 0.934 -0.501 -3.293 HO3 MAN 22\nMAN HO4 HO4 H 0 1 N Y N N N N 102.222 -28.476 20.286 2.958 -0.305 -0.587 HO4 MAN 23\nMAN HO6 HO6 H 0 1 N Y N N N N 98.593 -28.719 18.998 0.270 0.582 4.215 HO6 MAN
24\n#\nloop_\n_chem_comp_bond.comp_id\n_chem_comp_bond.atom_id_1\n_chem_comp_bond.atom_id_2\n_chem_comp_bond.value_order\n_chem_comp_bond.pdbx_aromatic_flag\n_chem_comp_bond.pdbx_stereo_config\n_chem_comp_bond.pdbx_ordinal\nMAN C1 C2 SING N N 1\nMAN C1 O5 SING N N 3\nMAN C1 H1 SING N N 4\nMAN C2 C3 SING N N 5\nMAN C2 H2 SING N N 7\nMAN C3 C4 SING N N 8\nMAN C3 O3 SING N N 9\nMAN C3 H3 SING N N 10\nMAN C4 C5 SING N N 11\nMAN C4 O4 SING N N 12\nMAN C4 H4 SING N N 13\nMAN C5 C6 SING N N 14\nMAN C5 O5 SING N N 15\nMAN C5 H5 SING N N 16\nMAN C6 O6 SING N N 17\nMAN C6 H61 SING N N 18\nMAN C6 H62 SING N N 19\nMAN O3 HO3 SING N N 22\nMAN O4 HO4 SING N N 23\nMAN O6 HO6 SING N N
24\n#\nloop_\n_pdbx_chem_comp_descriptor.comp_id\n_pdbx_chem_comp_descriptor.type\n_pdbx_chem_comp_descriptor.program\n_pdbx_chem_comp_descriptor.program_version\n_pdbx_chem_comp_descriptor.descriptor\nMAN SMILES ACDLabs 10.04 'OC1C(O)C(OC(O)C1O)CO'\nMAN SMILES_CANONICAL CACTVS 3.341 'OC[C@H]1O[C@H](O)[C@@H](O)[C@@H](O)[C@@H]1O'\nMAN SMILES CACTVS 3.341 'OC[CH]1O[CH](O)[CH](O)[CH](O)[CH]1O'\nMAN

SMILES_CANONICAL 'OpenEye OEToolkits' 1.5.0
'C([C@@H]1[C@H]([C@@H]([C@@H]([C@H](O1)O)O)O)O)O'\nMAN SMILES 'OpenEye
OEToolkits' 1.5.0 'C(C1C(C(C(C(O1)O)O)O)O)O'\nMAN InChI InChI 1.03
'InChI=1S/C6H12O6/c7-1-2-3(8)4(9)5(10)6(11)12-2/h2-
11H,1H2/t2-,3-,4+,5+,6+/m1/s1'\nMAN InChIKey InChI 1.03
WQZGKKKJIJFFOK-PQMKYFCFSA-
N\n#\nloop_\n_pdbx_chem_comp_identifier.comp_id\n_pdbx_chem_comp_ident
ifier.type\n_pdbx_chem_comp_identifier.program\n_pdbx_chem_comp_identi
fier.program_version\n_pdbx_chem_comp_identifier.identifier\nMAN
'SYSTEMATIC NAME' ACDLabs 10.04 alpha-D-mannopyranose\nMAN 'SYSTEMATIC
NAME' 'OpenEye OEToolkits' 1.5.0 '(2S,3S,4S,5S,6R)-6-
(hydroxymethyl)oxane-2,3,4,5-tetrol'\nMAN 'CONDENSED IUPAC
CARBOHYDRATE SYMBOL' GMML 1.0 DManpa\nMAN 'COMMON NAME' GMML 1.0 a-D-
mannopyranose\nMAN 'IUPAC CARBOHYDRATE SYMBOL' PDB-CARE 1.0 a-D-
Manp\nMAN 'SNFG CARBOHYDRATE SYMBOL' GMML 1.0
Man\n#\nloop_\n_pdbx_chem_comp_feature.comp_id\n_pdbx_chem_comp_featur
e.type\n_pdbx_chem_comp_feature.value\n_pdbx_chem_comp_feature.source\
n_pdbx_chem_comp_feature.support\nMAN 'CARBOHYDRATE ISOMER' D
PDB ?\nMAN 'CARBOHYDRATE RING' pyranose PDB ?\nMAN 'CARBOHYDRATE
ANOMER' alpha PDB ?\nMAN 'CARBOHYDRATE PRIMARY CARBONYL GROUP' aldose
PDB ?\n#\nloop_\n_pdbx_chem_comp_audit.comp_id\n_pdbx_chem_comp_audit.
action_type\n_pdbx_chem_comp_audit.date\n_pdbx_chem_comp_audit.process
ing_site\nMAN 'Create component' 1999-07-08 RCSB\nMAN 'Modify
descriptor' 2011-06-04 RCSB\nMAN 'Other modification' 2019-08-12
RCSB\nMAN 'Other modification' 2019-12-19 RCSB\nMAN 'Other
modification' 2020-07-03 RCSB\nMAN 'Modify name' 2020-07-17 RCSB\nMAN
'Modify synonyms' 2020-07-17 RCSB\nMAN 'Modify PCM' 2024-09-27
PDBE\n#\nloop_\n_pdbx_chem_comp_pcm.pcm_id\n_pdbx_chem_comp_pcm.comp_i
d\n_pdbx_chem_comp_pcm.modified_residue_id\n_pdbx_chem_comp_pcm.type\n
_pdbx_chem_comp_pcm.category\n_pdbx_chem_comp_pcm.position\n_pdbx_chem
_comp_pcm.polypeptide_position\n_pdbx_chem_comp_pcm.comp_id_linking_at
om\n_pdbx_chem_comp_pcm.modified_residue_id_linking_atom\n_pdbx_chem_c
omp_pcm.uniprot_specific_ptm_accession\n_pdbx_chem_comp_pcm.uniprot_ge
neric_ptm_accession\n1 MAN SER O-Glycosylation Carbohydrate 'Amino-
acid side chain' 'Any position' C1 OG ? ?\n2 MAN THR O-Glycosylation
Carbohydrate 'Amino-acid side chain' 'Any position' C1 OG1 ? ?\n3 MAN
TRP C-Mannosylation Carbohydrate 'Amino-acid side chain' 'Any
position' C1 CD1 ? ?\n4 MAN 3FG None Carbohydrate 'Amino-acid side
chain' 'Any position' C1 OD1 ? ?\n5 MAN ARG None Carbohydrate 'Amino-
acid side chain' 'Any position' C4 NH2 ? ?\n6 MAN ASN None
Carbohydrate 'Amino-acid side chain' 'Any position' C1 ND2 ? ?\n7 MAN
D4P None Carbohydrate 'Amino-acid side chain' 'Any position' C1
O4 ? ?\n8 MAN GLU None Carbohydrate 'Amino-acid side chain' 'Any
position' C1 OE1 ? ?\n10 MAN MDF None Carbohydrate 'Amino-acid side
chain' 'Any position' C1
OH2 ? ?\n#\nloop_\n_pdbe_chem_comp_synonyms.comp_id\n_pdbe_chem_comp_s
ynonyms.name\n_pdbe_chem_comp_synonyms.provenance\n_pdbe_chem_comp_syn
onyms.type\nMAN alpha-D-mannose wwPDB ?\nMAN D-mannose wwPDB ?\nMAN
mannose
wwPDB ?\n#\nloop_\n_software.name\n_software.version\n_software.descri
ption\nrdkit 2023.09.6 'Core functionality.'\npdbeccdutils 0.8.6
'Wrapper to provide 2D templates and molecular

fragments.'\n#\nloop_\n_pdbe_chem_comp_atom_depiction.comp_id\n_pdbe_c
hem_comp_atom_depiction.atom_id\n_pdbe_chem_comp_atom_depiction.elemen
t\n_pdbe_chem_comp_atom_depiction.model_Cartn_x\n_pdbe_chem_comp_atom_
depiction.model_Cartn_y\n_pdbe_chem_comp_atom_depiction.pdbx_ordinal\n
MAN C1 C 7.702 0.750 1\nMAN C2 C 7.702 -0.750 2\nMAN C3 C 6.404 -1.500
3\nMAN C4 C 5.104 -0.750 4\nMAN C5 C 5.104 0.750 5\nMAN C6 C 3.805
1.500 6\nMAN O3 O 6.404 -3.000 9\nMAN O4 O 3.805 -1.500 10\nMAN O5 O
6.404 1.500 11\nMAN O6 O 3.805 3.000
12\n#\nloop_\n_pdbe_chem_comp_bond_depiction.comp_id\n_pdbe_chem_comp_
bond_depiction.atom_id_1\n_pdbe_chem_comp_bond_depiction.atom_id_2\n_p
dbe_chem_comp_bond_depiction.value_order\n_pdbe_chem_comp_bond_depicti
on.bond_dir\n_pdbe_chem_comp_bond_depiction.pdbx_ordinal\nMAN C1 C2
SINGLE NONE 1\nMAN C1 O5 SINGLE NONE 3\nMAN C2 C3 SINGLE NONE 4\nMAN
C3 C4 SINGLE NONE 6\nMAN C3 O3 SINGLE BEGINWEDGE 7\nMAN C4 C5 SINGLE
NONE 8\nMAN C4 O4 SINGLE BEGINDASH 9\nMAN C5 C6 SINGLE BEGINWEDGE
10\nMAN C5 O5 SINGLE NONE 11\nMAN C6 O6 SINGLE NONE
12\n#\nloop_\n_pdbe_chem_comp_substructure.comp_id\n_pdbe_chem_comp_su
bstructure.substructure_name\n_pdbe_chem_comp_substructure.id\n_pdbe_c
hem_comp_substructure.substructure_type\n_pdbe_chem_comp_substructure.
substructure_smiles\n_pdbe_chem_comp_substructure.substructure_inchis\
n_pdbe_chem_comp_substructure.substructure_inchikeys\nMAN
MurckoScaffold S1 scaffold C1CCOCC1 InChI=1S/C5H10O/c1-2-4-6-5-3-1/h1-
5H2 DHXVGJBLRPWPCS-UHFFFAOYSA-N\nMAN pyranose F1 fragment OC1CCCCO1
InChI=1S/C5H10O2/c6-5-3-1-2-4-7-5/h5-6H,1-4H2 CELWCAITJAEQNL-
UHFFFAOYSA-
N\n#\nloop_\n_pdbe_chem_comp_substructure_mapping.comp_id\n_pdbe_chem_
comp_substructure_mapping.atom_id\n_pdbe_chem_comp_substructure_mappin
g.substructure_id\n_pdbe_chem_comp_substructure_mapping.substructure_o
rdinal\nMAN C1 S1 1\nMAN C2 S1 1\nMAN C3 S1 1\nMAN C4 S1 1\nMAN C5 S1
1\nMAN O5 S1 1\nMAN C4 F1 1\nMAN C5 F1 1\nMAN O5 F1 1\nMAN C1 F1
1\nMAN C2 F1 1\nMAN C3 F1
1\n#\n_pdbe_chem_comp_rdkit_properties.comp_id
MAN\n_pdbe_chem_comp_rdkit_properties.exactmw
180.063\n_pdbe_chem_comp_rdkit_properties.amw
180.156\n_pdbe_chem_comp_rdkit_properties.lipinskiHBA
6\n_pdbe_chem_comp_rdkit_properties.lipinskiHBD
5\n_pdbe_chem_comp_rdkit_properties.NumRotatableBonds
6\n_pdbe_chem_comp_rdkit_properties.NumHBD
5\n_pdbe_chem_comp_rdkit_properties.NumHBA
6\n_pdbe_chem_comp_rdkit_properties.NumHeavyAtoms
12\n_pdbe_chem_comp_rdkit_properties.NumAtoms
24\n_pdbe_chem_comp_rdkit_properties.NumHeteroatoms
6\n_pdbe_chem_comp_rdkit_properties.NumAmideBonds
0\n_pdbe_chem_comp_rdkit_properties.FractionCSP3
1\n_pdbe_chem_comp_rdkit_properties.NumRings
1\n_pdbe_chem_comp_rdkit_properties.NumAromaticRings
0\n_pdbe_chem_comp_rdkit_properties.NumAliphaticRings
1\n_pdbe_chem_comp_rdkit_properties.NumSaturatedRings
1\n_pdbe_chem_comp_rdkit_properties.NumHeterocycles
1\n_pdbe_chem_comp_rdkit_properties.NumAromaticHeterocycles
0\n_pdbe_chem_comp_rdkit_properties.NumSaturatedHeterocycles
1\n_pdbe_chem_comp_rdkit_properties.NumAliphaticHeterocycles

1\n_pdbe_chem_comp_rdkit_properties.NumSpiroAtoms
0\n_pdbe_chem_comp_rdkit_properties.NumBridgeheadAtoms
0\n_pdbe_chem_comp_rdkit_properties.NumAtomStereoCenters
5\n_pdbe_chem_comp_rdkit_properties.NumUnspecifiedAtomStereoCenters
0\n_pdbe_chem_comp_rdkit_properties.labuteASA
85.936\n_pdbe_chem_comp_rdkit_properties.tpsa
110.380\n_pdbe_chem_comp_rdkit_properties.CrippenClogP -
3.221\n_pdbe_chem_comp_rdkit_properties.CrippenMR
35.986\n_pdbe_chem_comp_rdkit_properties.chi0v
5.449\n_pdbe_chem_comp_rdkit_properties.chi1v
2.679\n_pdbe_chem_comp_rdkit_properties.chi2v
1.092\n_pdbe_chem_comp_rdkit_properties.chi3v
1.092\n_pdbe_chem_comp_rdkit_properties.chi4v
0.552\n_pdbe_chem_comp_rdkit_properties.chi0n
17.449\n_pdbe_chem_comp_rdkit_properties.chi1n
8.220\n_pdbe_chem_comp_rdkit_properties.chi2n
1.092\n_pdbe_chem_comp_rdkit_properties.chi3n
1.092\n_pdbe_chem_comp_rdkit_properties.chi4n
0.552\n_pdbe_chem_comp_rdkit_properties.hallKierAlpha -
0.240\n_pdbe_chem_comp_rdkit_properties.kappa1
2.412\n_pdbe_chem_comp_rdkit_properties.kappa2
3.649\n_pdbe_chem_comp_rdkit_properties.kappa3
1.582\n_pdbe_chem_comp_rdkit_properties.Phi
0.733\n#\nloop_\n_pdbe_chem_comp_external_mappings.comp_id\n_pdbe_chem_comp_external_mappings.source\n_pdbe_chem_comp_external_mappings.resource\n_pdbe_chem_comp_external_mappings.resource_id\nMAN UniChem ChEMBL CHEMBL365590\nMAN UniChem 'KEGG LIGAND' C00936\nMAN UniChem ChEBI 28729\nMAN UniChem ZINC ZINC000003860903\nMAN UniChem fdasrs W3F28J9G0W\nMAN UniChem SureChEMBL SCHEMBL76882\nMAN UniChem HMDB HMDB0000169\nMAN UniChem 'PubChem TPHARMA' 14916352\nMAN UniChem NMRShiftDB 60078611\nMAN UniChem ACTor 29696-75-1\nMAN UniChem ACTor 7296-15-3\nMAN UniChem Nikkaji J15.393A\nMAN UniChem BindingDb 50467903\nMAN UniChem MetaboLights MTBLC28729\nMAN UniChem BRENDA 102433\nMAN UniChem BRENDA 16456\nMAN UniChem BRENDA 29727\nMAN UniChem BRENDA 47808\nMAN UniChem BRENDA 756\nMAN UniChem BRENDA 993\nMAN UniChem Rhea 28729\nMAN UniChem CCDC ADMANN\nMAN UniChem PubChem
185698\n#\nloop_\n_pdbe_chem_comp_rdkit_conformer.comp_id\n_pdbe_chem_comp_rdkit_conformer.atom_id\n_pdbe_chem_comp_rdkit_conformer.Cartn_x_rdkit\n_pdbe_chem_comp_rdkit_conformer.Cartn_y_rdkit\n_pdbe_chem_comp_rdkit_conformer.Cartn_z_rdkit\n_pdbe_chem_comp_rdkit_conformer.rdkit_method\n_pdbe_chem_comp_rdkit_conformer.rdkit_ordinal\nMAN C1 -1.033 1.513 -0.341 ETKDGv3 1\nMAN C2 -1.809 0.174 -0.462 ETKDGv3 2\nMAN C3 -1.145 -0.938 0.369 ETKDGv3 3\nMAN C4 0.371 -0.974 0.096 ETKDGv3 4\nMAN C5 0.975 0.443 0.267 ETKDGv3 5\nMAN C6 2.483 0.444 -0.020 ETKDGv3 6\nMAN O3 -1.726 -2.175 0.042 ETKDGv3 9\nMAN O4 1.003 -1.904 0.941 ETKDGv3 10\nMAN O5 0.353 1.367 -0.610 ETKDGv3 11\nMAN O6 2.728 0.077 -1.350 ETKDGv3 12\nMAN H1 -1.437 2.184 -1.129 ETKDGv3 13\nMAN H2 -2.848 0.318 -0.083 ETKDGv3 14\nMAN H3 -1.322 -0.725 1.449 ETKDGv3 15\nMAN H4 0.528 -1.305 -0.955 ETKDGv3 16\nMAN H5 0.858 0.774 1.324 ETKDGv3 17\nMAN H61 2.883 1.466 0.162 ETKDGv3 18\nMAN H62 3.004 -0.257 0.670 ETKDGv3 19\nMAN HO3 -1.516 -2.799 0.786 ETKDGv3 22\nMAN HO4 0.879 -

1.594 1.877 ETKDGv3 23\nMAN HO6 3.713 0.097 -1.464 ETKDGv3 24\n#\ndata_PO4-2\n#\n_chem_comp.id PO4\n_chem_comp.name 'PHOSPHATE ION'\n_chem_comp.type NON-POLYMER\n_chem_comp.pdbx_type HETAI\n_chem_comp.formula 'O4 P'\n_chem_comp.mon_nstd_parent_comp_id ?\n_chem_comp.pdbx_synonyms ?\n_chem_comp.pdbx_formal_charge -3\n_chem_comp.pdbx_initial_date 1999-07-08\n_chem_comp.pdbx_modified_date 2011-06-04\n_chem_comp.pdbx_ambiguous_flag N\n_chem_comp.pdbx_release_status REL\n_chem_comp.pdbx_replaced_by ?\n_chem_comp.pdbx_replaces IPS\n_chem_comp.formula_weight 94.971\n_chem_comp.one_letter_code ?\n_chem_comp.three_letter_code PO4\n_chem_comp.pdbx_model_coordinates_details ?\n_chem_comp.pdbx_model_coordinates_missing_flag N\n_chem_comp.pdbx_ideal_coordinates_details ?\n_chem_comp.pdbx_ideal_coordinates_missing_flag N\n_chem_comp.pdbx_model_coordinates_db_code 1IXG\n_chem_comp.pdbx_subcomponent_list ?\n_chem_comp.pdbx_processing_site EBI\n#\nloop_\n_chem_comp_atom.comp_id\n_chem_comp_atom.atom_id\n_chem_comp_atom.alt_atom_id\n_chem_comp_atom.type_symbol\n_chem_comp_atom.charge\n_chem_comp_atom.pdbx_align\n_chem_comp_atom.pdbx_aromatic_flag\n_chem_comp_atom.pdbx_leaving_atom_flag\n_chem_comp_atom.pdbx_stereo_config\n_chem_comp_atom.pdbx_backbone_atom_flag\n_chem_comp_atom.pdbx_n_terminal_atom_flag\n_chem_comp_atom.pdbx_c_terminal_atom_flag\n_chem_comp_atom.model_Cartn_x\n_chem_comp_atom.model_Cartn_y\n_chem_comp_atom.model_Cartn_z\n_chem_comp_atom.pdbx_model_Cartn_x_ideal\n_chem_comp_atom.pdbx_model_Cartn_y_ideal\n_chem_comp_atom.pdbx_model_Cartn_z_ideal\n_chem_comp_atom.pdbx_component_atom_id\n_chem_comp_atom.pdbx_component_comp_id\n_chem_comp_atom.pdbx_ordinal\nPO4 P P P 0 1 N N N N N N 29.995 23.516 13.249 0.000 0.000 0.000 P PO4 1\nPO4 O1 O1 O 0 1 N N N N N N 31.092 22.988 14.164 0.000 -1.288 -0.911 O1 PO4 2\nPO4 O3 O3 O -1 1 N N N N N N 29.646 22.518 12.126 -1.288 0.000 0.911 O3 PO4 4\nPO4 O4 O4 O -1 1 N N N N N N 28.727 23.744 14.161 1.288 0.000 0.911 O4 PO4 5\n#\nloop_\n_chem_comp_bond.comp_id\n_chem_comp_bond.atom_id_1\n_chem_comp_bond.atom_id_2\n_chem_comp_bond.value_order\n_chem_comp_bond.pdbx_aromatic_flag\n_chem_comp_bond.pdbx_stereo_config\n_chem_comp_bond.pdbx_ordinal\nPO4 P O1 DOUB N N 1\nPO4 P O3 SING N N 3\nPO4 P O4 SING N N 4\n#\nloop_\n_pdbx_chem_comp_descriptor.comp_id\n_pdbx_chem_comp_descriptor.type\n_pdbx_chem_comp_descriptor.program\n_pdbx_chem_comp_descriptor.program_version\n_pdbx_chem_comp_descriptor.descriptor\nPO4 SMILES ACDLabs 10.04 '[O-]P([O-])([O-])=O'\nPO4 SMILES_CANONICAL CACTVS 3.341 '[O-][P]([O-])([O-])=O'\nPO4 SMILES CACTVS 3.341 '[O-][P]([O-])([O-])=O'\nPO4 SMILES_CANONICAL 'OpenEye OEToolkits' 1.5.0 '[O-]P(=O)([O-])[O-]'\nPO4 SMILES 'OpenEye OEToolkits' 1.5.0 '[O-]P(=O)([O-])[O-]'\nPO4 InChI InChI 1.03 InChI=1S/H3O4P/c1-5(2,3)4/h(H3,1,2,3,4)/p-3\nPO4 InChIKey InChI 1.03 NBIIXXVUZAFLBC-UHFFFAOYSA-K\n#\nloop_\n_pdbx_chem_comp_identifier.comp_id\n_pdbx_chem_comp_identifier.type\n_pdbx_chem_comp_identifier.program\n_pdbx_chem_comp_identifier.program_version\n_pdbx_chem_comp_identifier.identifier\nPO4 'SYSTEMATIC NAME' ACDLabs 10.04 phosphate\nPO4 'SYSTEMATIC NAME' 'OpenEye OEToolkits' 1.5.0

phosphate\n#\nloop_\n_pdbx_chem_comp_audit.comp_id\n_pdbx_chem_comp_au
dit.action_type\n_pdbx_chem_comp_audit.date\n_pdbx_chem_comp_audit.pro
cessing_site\nPO4 'Create component' 1999-07-08 EBI\nPO4 'Modify
descriptor' 2011-06-04
RCSB\n#\n_pdbe_chem_comp_drugbank_details.comp_id
PO4\n_pdbe_chem_comp_drugbank_details.drugbank_id
DB14523\n_pdbe_chem_comp_drugbank_details.type 'small
molecule'\n_pdbe_chem_comp_drugbank_details.name 'Phosphate
ion'\n_pdbe_chem_comp_drugbank_details.description ?\n_pdbe_chem_comp_
drugbank_details.cas_number 14265-44-
2\n_pdbe_chem_comp_drugbank_details.mechanism_of_action ?\n#\nloop_\n_
pdbe_chem_comp_synonyms.comp_id\n_pdbe_chem_comp_synonyms.name\n_pdbe_
chem_comp_synonyms.provenance\n_pdbe_chem_comp_synonyms.type\nPO4
Orthophosphate DrugBank ?\nPO4 Phosphate
DrugBank ?\n#\n_pdbe_chem_comp_drugbank_classification.comp_id
PO4\n_pdbe_chem_comp_drugbank_classification.drugbank_id
DB14523\n_pdbe_chem_comp_drugbank_classification.parent 'Non-metal
phosphates'\n_pdbe_chem_comp_drugbank_classification.kingdom
'Inorganic compounds'\n_pdbe_chem_comp_drugbank_classification.class
'Non-metal oxoanionic
compounds'\n_pdbe_chem_comp_drugbank_classification.superclass
'Homogeneous non-metal
compounds'\n_pdbe_chem_comp_drugbank_classification.description\n'This
compound belongs to the class of inorganic compounds known as non-
metal phosphates. These are inorganic non-metallic compounds
containing a phosphate as its largest
oxoanion.'\n#\nloop_\n_software.name\n_software.version\n_software.des
cription\nrdkit 2023.09.6 'Core functionality.'\npdbeccdutils 0.8.6
'Wrapper to provide 2D templates and molecular
fragments.'\n#\nloop_\n_pdbe_chem_comp_atom_depiction.comp_id\n_pdbe_c
hem_comp_atom_depiction.atom_id\n_pdbe_chem_comp_atom_depiction.elemen
t\n_pdbe_chem_comp_atom_depiction.model_Cartn_x\n_pdbe_chem_comp_atom_
depiction.model_Cartn_y\n_pdbe_chem_comp_atom_depiction.pdbx_ordinal\n
PO4 P P 4.299 0.000 1\nPO4 O1 O 5.598 0.750 2\nPO4 O3 O 3.549 1.299
4\nPO4 O4 O 5.049 -1.299
5\n#\nloop_\n_pdbe_chem_comp_bond_depiction.comp_id\n_pdbe_chem_comp_b
ond_depiction.atom_id_1\n_pdbe_chem_comp_bond_depiction.atom_id_2\n_pd
be_chem_comp_bond_depiction.value_order\n_pdbe_chem_comp_bond_depictio
n.bond_dir\n_pdbe_chem_comp_bond_depiction.pdbx_ordinal\nPO4 P O1
DOUBLE NONE 1\nPO4 P O3 SINGLE NONE 3\nPO4 P O4 SINGLE NONE
4\n#\n_pdbe_chem_comp_substructure.comp_id
PO4\n_pdbe_chem_comp_substructure.substructure_name
phosphate\n_pdbe_chem_comp_substructure.id
F1\n_pdbe_chem_comp_substructure.substructure_type
fragment\n_pdbe_chem_comp_substructure.substructure_smiles
O=P(O)(O)O\n_pdbe_chem_comp_substructure.substructure_inchis
InChI=1S/H3O4P/c1-
5(2,3)4/h(H3,1,2,3,4)\n_pdbe_chem_comp_substructure.substructure_inchi
keys NBIIXXVUZAFLBC-UHFFFAOYSA-
N\n#\nloop_\n_pdbe_chem_comp_substructure_mapping.comp_id\n_pdbe_chem_
comp_substructure_mapping.atom_id\n_pdbe_chem_comp_substructure_mappin
g.substructure_id\n_pdbe_chem_comp_substructure_mapping.substructure_o

rdinal\nPO4 P F1 1\nPO4 O1 F1 1\nPO4 O3 F1 1\nPO4 O4 F1
1\n#\n_pdbe_chem_comp_rdkit_properties.comp_id
PO4\n_pdbe_chem_comp_rdkit_properties.exactmw
94.955\n_pdbe_chem_comp_rdkit_properties.amw
94.970\n_pdbe_chem_comp_rdkit_properties.lipinskiHBA
4\n_pdbe_chem_comp_rdkit_properties.lipinskiHBD
0\n_pdbe_chem_comp_rdkit_properties.NumRotatableBonds
0\n_pdbe_chem_comp_rdkit_properties.NumHBD
0\n_pdbe_chem_comp_rdkit_properties.NumHBA
4\n_pdbe_chem_comp_rdkit_properties.NumHeavyAtoms
5\n_pdbe_chem_comp_rdkit_properties.NumAtoms
5\n_pdbe_chem_comp_rdkit_properties.NumHeteroatoms
5\n_pdbe_chem_comp_rdkit_properties.NumAmideBonds
0\n_pdbe_chem_comp_rdkit_properties.FractionCSP3
0\n_pdbe_chem_comp_rdkit_properties.NumRings
0\n_pdbe_chem_comp_rdkit_properties.NumAromaticRings
0\n_pdbe_chem_comp_rdkit_properties.NumAliphaticRings
0\n_pdbe_chem_comp_rdkit_properties.NumSaturatedRings
0\n_pdbe_chem_comp_rdkit_properties.NumHeterocycles
0\n_pdbe_chem_comp_rdkit_properties.NumAromaticHeterocycles
0\n_pdbe_chem_comp_rdkit_properties.NumSaturatedHeterocycles
0\n_pdbe_chem_comp_rdkit_properties.NumAliphaticHeterocycles
0\n_pdbe_chem_comp_rdkit_properties.NumSpiroAtoms
0\n_pdbe_chem_comp_rdkit_properties.NumBridgeheadAtoms
0\n_pdbe_chem_comp_rdkit_properties.NumAtomStereoCenters
0\n_pdbe_chem_comp_rdkit_properties.NumUnspecifiedAtomStereoCenters
0\n_pdbe_chem_comp_rdkit_properties.labuteASA
28.307\n_pdbe_chem_comp_rdkit_properties.tpsa
86.250\n_pdbe_chem_comp_rdkit_properties.CrippenClogP -
2.825\n_pdbe_chem_comp_rdkit_properties.CrippenMR
7.606\n_pdbe_chem_comp_rdkit_properties.chi0v
2.975\n_pdbe_chem_comp_rdkit_properties.chi1v
2.191\n_pdbe_chem_comp_rdkit_properties.chi2v
0\n_pdbe_chem_comp_rdkit_properties.chi3v
0\n_pdbe_chem_comp_rdkit_properties.chi4v
0\n_pdbe_chem_comp_rdkit_properties.chi0n
2.080\n_pdbe_chem_comp_rdkit_properties.chi1n
0.730\n_pdbe_chem_comp_rdkit_properties.chi2n
0\n_pdbe_chem_comp_rdkit_properties.chi3n
0\n_pdbe_chem_comp_rdkit_properties.chi4n
0\n_pdbe_chem_comp_rdkit_properties.hallKierAlpha
0.110\n_pdbe_chem_comp_rdkit_properties.kappa1
5.110\n_pdbe_chem_comp_rdkit_properties.kappa2
1.065\n_pdbe_chem_comp_rdkit_properties.kappa3
1512.242\n_pdbe_chem_comp_rdkit_properties.Phi
1.088\n#\nloop_\n_pdbe_chem_comp_external_mappings.comp_id\n_pdbe_chem
_comp_external_mappings.source\n_pdbe_chem_comp_external_mappings.reso
urce\n_pdbe_chem_comp_external_mappings.resource_id\nPO4 UniChem
DrugBank DB14523\nPO4 UniChem ChEBI 18367\nPO4 UniChem eMolecules
882178\nPO4 UniChem fdasrs NK08V8K8HR\nPO4 UniChem NMRShiftDB
30000818\nPO4 UniChem BindingDb 50155537\nPO4 UniChem MetaboLights
MTBLC18367\nPO4 UniChem BRENDA 21671\nPO4 UniChem BRENDA 867\nPO4

UniChem ChemicalBook CB3409949\nPO4 UniChem ChemicalBook CB7191961\nPO4 UniChem rxnorm 'PHOSPHATE ION'\nPO4 UniChem 'Probes And Drugs' PD094531\nPO4 UniChem CCDC EXIZIX\nPO4 UniChem 'EPA CompTox Dashboard' DTXSID7039672\nPO4 UniChem 'PubChem TPHARMA' 15218698\nPO4 UniChem PubChem 1061\nPO4 UniChem ACTor 14265-44-2\nPO4 UniChem Nikkaji J215.970H\nPO4 UniChem Nikkaji J351.173A\n#\nloop_\n_pdbe_chem_comp_rdkit_conformer.comp_id\n_pdbe_chem_comp_rdkit_conformer.atom_id\n_pdbe_chem_comp_rdkit_conformer.Cartn_x_rdkit\n_pdbe_chem_comp_rdkit_conformer.Cartn_y_rdkit\n_pdbe_chem_comp_rdkit_conformer.Cartn_z_rdkit\n_pdbe_chem_comp_rdkit_conformer.rdkit_method\n_pdbe_chem_comp_rdkit_conformer.rdkit_ordinal\nPO4 P -0.003 0.002 0.036 ETKDGv3 1\nPO4 O1 -0.117 0.079 1.541 ETKDGv3 2\nPO4 O3 -1.075 -1.165 -0.552 ETKDGv3 4\nPO4 O4 -0.383 1.506 -0.636 ETKDGv3 5\n#\ndata_PO4-2-3\n#\n_chem_comp.id PO4\n_chem_comp.name 'PHOSPHATE ION'\n_chem_comp.type NON-POLYMER\n_chem_comp.pdbx_type HETAI\n_chem_comp.formula 'O4 P'\n_chem_comp.mon_nstd_parent_comp_id ?\n_chem_comp.pdbx_synonyms ?\n_chem_comp.pdbx_formal_charge -3\n_chem_comp.pdbx_initial_date 1999-07-08\n_chem_comp.pdbx_modified_date 2011-06-04\n_chem_comp.pdbx_ambiguous_flag N\n_chem_comp.pdbx_release_status REL\n_chem_comp.pdbx_replaced_by ?\n_chem_comp.pdbx_replaces IPS\n_chem_comp.formula_weight 94.971\n_chem_comp.one_letter_code ?\n_chem_comp.three_letter_code PO4\n_chem_comp.pdbx_model_coordinates_details ?\n_chem_comp.pdbx_model_coordinates_missing_flag N\n_chem_comp.pdbx_ideal_coordinates_details ?\n_chem_comp.pdbx_ideal_coordinates_missing_flag N\n_chem_comp.pdbx_model_coordinates_db_code 1IXG\n_chem_comp.pdbx_subcomponent_list ?\n_chem_comp.pdbx_processing_site EBI\n#\nloop_\n_chem_comp_atom.comp_id\n_chem_comp_atom.atom_id\n_chem_comp_atom.alt_atom_id\n_chem_comp_atom.type_symbol\n_chem_comp_atom.charge\n_chem_comp_atom.pdbx_align\n_chem_comp_atom.pdbx_aromatic_flag\n_chem_comp_atom.pdbx_leaving_atom_flag\n_chem_comp_atom.pdbx_stereo_config\n_chem_comp_atom.pdbx_backbone_atom_flag\n_chem_comp_atom.pdbx_n_terminal_atom_flag\n_chem_comp_atom.pdbx_c_terminal_atom_flag\n_chem_comp_atom.model_Cartn_x\n_chem_comp_atom.model_Cartn_y\n_chem_comp_atom.model_Cartn_z\n_chem_comp_atom.pdbx_model_Cartn_x_ideal\n_chem_comp_atom.pdbx_model_Cartn_y_ideal\n_chem_comp_atom.pdbx_model_Cartn_z_ideal\n_chem_comp_atom.pdbx_component_atom_id\n_chem_comp_atom.pdbx_component_comp_id\n_chem_comp_atom.pdbx_ordinal\nPO4 P P P 0 1 N N N N N N 29.995 23.516 13.249 0.000 0.000 0.000 P PO4 1\nPO4 O1 O1 O 0 1 N N N N N N 31.092 22.988 14.164 0.000 -1.288 -0.911 O1 PO4 2\nPO4 O4 O4 O -1 1 N N N N N N 28.727 23.744 14.161 1.288 0.000 0.911 O4 PO4 5\n#\nloop_\n_chem_comp_bond.comp_id\n_chem_comp_bond.atom_id_1\n_chem_comp_bond.atom_id_2\n_chem_comp_bond.value_order\n_chem_comp_bond.pdbx_aromatic_flag\n_chem_comp_bond.pdbx_stereo_config\n_chem_comp_bond.pdbx_ordinal\nPO4 P O1 DOUB N N 1\nPO4 P O4 SING N N 4\n#\nloop_\n_pdbx_chem_comp_descriptor.comp_id\n_pdbx_chem_comp_descriptor.type\n_pdbx_chem_comp_descriptor.program\n_pdbx_chem_comp_descriptor.program_version\n_pdbx_chem_comp_descriptor.descriptor\nPO4 SMILES ACDLabs 10.04 '[O-]P([O-])([O-])=O'\nPO4 SMILES_CANONICAL CACTVS 3.341 '[O-][P]([O-])([O-])=O'\nPO4 SMILES CACTVS 3.341

'[O-][P]([O-])([O-])=O'\nPO4 SMILES_CANONICAL 'OpenEye OEToolkits' 1.5.0 '[O-]P(=O)([O-])[O-]'\nPO4 SMILES 'OpenEye OEToolkits' 1.5.0 '[O-]P(=O)([O-])[O-]'\nPO4 InChI InChI 1.03 InChI=1S/H3O4P/c1-5(2,3)4/h(H3,1,2,3,4)/p-3\nPO4 InChIKey InChI 1.03 NBIIXXVUZAFLBC-UHFFFAOYSA-K\n#\nloop_\n_pdbx_chem_comp_identifier.comp_id\n_pdbx_chem_comp_identifier.type\n_pdbx_chem_comp_identifier.program\n_pdbx_chem_comp_identifier.program_version\n_pdbx_chem_comp_identifier.identifier\nPO4 'SYSTEMATIC NAME' ACDLabs 10.04 phosphate\nPO4 'SYSTEMATIC NAME' 'OpenEye OEToolkits' 1.5.0 phosphate\n#\nloop_\n_pdbx_chem_comp_audit.comp_id\n_pdbx_chem_comp_audit.action_type\n_pdbx_chem_comp_audit.date\n_pdbx_chem_comp_audit.processing_site\nPO4 'Create component' 1999-07-08 EBI\nPO4 'Modify descriptor' 2011-06-04 RCSB\n#\n_pdbe_chem_comp_drugbank_details.comp_id PO4\n_pdbe_chem_comp_drugbank_details.drugbank_id DB14523\n_pdbe_chem_comp_drugbank_details.type 'small molecule'\n_pdbe_chem_comp_drugbank_details.name 'Phosphate ion'\n_pdbe_chem_comp_drugbank_details.description ?\n_pdbe_chem_comp_drugbank_details.cas_number 14265-44-2\n_pdbe_chem_comp_drugbank_details.mechanism_of_action ?\n#\nloop_\n_pdbe_chem_comp_synonyms.comp_id\n_pdbe_chem_comp_synonyms.name\n_pdbe_chem_comp_synonyms.provenance\n_pdbe_chem_comp_synonyms.type\nPO4 Orthophosphate DrugBank ?\nPO4 Phosphate DrugBank ?\n#\n_pdbe_chem_comp_drugbank_classification.comp_id PO4\n_pdbe_chem_comp_drugbank_classification.drugbank_id DB14523\n_pdbe_chem_comp_drugbank_classification.parent 'Non-metal phosphates'\n_pdbe_chem_comp_drugbank_classification.kingdom 'Inorganic compounds'\n_pdbe_chem_comp_drugbank_classification.class 'Non-metal oxoanionic compounds'\n_pdbe_chem_comp_drugbank_classification.superclass 'Homogeneous non-metal compounds'\n_pdbe_chem_comp_drugbank_classification.description\n'This compound belongs to the class of inorganic compounds known as non-metal phosphates. These are inorganic non-metallic compounds containing a phosphate as its largest oxoanion.'\n#\nloop_\n_software.name\n_software.version\n_software.description\nrdkit 2023.09.6 'Core functionality.'\npdbeccdutils 0.8.6 'Wrapper to provide 2D templates and molecular fragments.'\n#\nloop_\n_pdbe_chem_comp_atom_depiction.comp_id\n_pdbe_chem_comp_atom_depiction.atom_id\n_pdbe_chem_comp_atom_depiction.element\n_pdbe_chem_comp_atom_depiction.model_Cartn_x\n_pdbe_chem_comp_atom_depiction.model_Cartn_y\n_pdbe_chem_comp_atom_depiction.pdbx_ordinal\nPO4 P P 4.299 0.000 1\nPO4 O1 O 5.598 0.750 2\nPO4 O4 O 5.049 -1.299 5\n#\nloop_\n_pdbe_chem_comp_bond_depiction.comp_id\n_pdbe_chem_comp_bond_depiction.atom_id_1\n_pdbe_chem_comp_bond_depiction.atom_id_2\n_pdbe_chem_comp_bond_depiction.value_order\n_pdbe_chem_comp_bond_depiction.bond_dir\n_pdbe_chem_comp_bond_depiction.pdbx_ordinal\nPO4 P O1 DOUBLE NONE 1\nPO4 P O4 SINGLE NONE 4\n#\n_pdbe_chem_comp_substructure.comp_id PO4\n_pdbe_chem_comp_substructure.substructure_name phosphate\n_pdbe_chem_comp_substructure.id

F1\n_pdbe_chem_comp_substructure.substructure_type
fragment\n_pdbe_chem_comp_substructure.substructure_smiles
O=P(O)(O)O\n_pdbe_chem_comp_substructure.substructure_inchis
InChI=1S/H3O4P/c1-
5(2,3)4/h(H3,1,2,3,4)\n_pdbe_chem_comp_substructure.substructure_inchi
keys NBIIXXVUZAFLBC-UHFFFAOYSA-
N\n#\nloop_\n_pdbe_chem_comp_substructure_mapping.comp_id\n_pdbe_chem_
comp_substructure_mapping.atom_id\n_pdbe_chem_comp_substructure_mappin
g.substructure_id\n_pdbe_chem_comp_substructure_mapping.substructure_o
rdinal\nPO4 P F1 1\nPO4 O1 F1 1\nPO4 O4 F1
1\n#\n_pdbe_chem_comp_rdkit_properties.comp_id
PO4\n_pdbe_chem_comp_rdkit_properties.exactmw
94.955\n_pdbe_chem_comp_rdkit_properties.amw
94.970\n_pdbe_chem_comp_rdkit_properties.lipinskiHBA
4\n_pdbe_chem_comp_rdkit_properties.lipinskiHBD
0\n_pdbe_chem_comp_rdkit_properties.NumRotatableBonds
0\n_pdbe_chem_comp_rdkit_properties.NumHBD
0\n_pdbe_chem_comp_rdkit_properties.NumHBA
4\n_pdbe_chem_comp_rdkit_properties.NumHeavyAtoms
5\n_pdbe_chem_comp_rdkit_properties.NumAtoms
5\n_pdbe_chem_comp_rdkit_properties.NumHeteroatoms
5\n_pdbe_chem_comp_rdkit_properties.NumAmideBonds
0\n_pdbe_chem_comp_rdkit_properties.FractionCSP3
0\n_pdbe_chem_comp_rdkit_properties.NumRings
0\n_pdbe_chem_comp_rdkit_properties.NumAromaticRings
0\n_pdbe_chem_comp_rdkit_properties.NumAliphaticRings
0\n_pdbe_chem_comp_rdkit_properties.NumSaturatedRings
0\n_pdbe_chem_comp_rdkit_properties.NumHeterocycles
0\n_pdbe_chem_comp_rdkit_properties.NumAromaticHeterocycles
0\n_pdbe_chem_comp_rdkit_properties.NumSaturatedHeterocycles
0\n_pdbe_chem_comp_rdkit_properties.NumAliphaticHeterocycles
0\n_pdbe_chem_comp_rdkit_properties.NumSpiroAtoms
0\n_pdbe_chem_comp_rdkit_properties.NumBridgeheadAtoms
0\n_pdbe_chem_comp_rdkit_properties.NumAtomStereoCenters
0\n_pdbe_chem_comp_rdkit_properties.NumUnspecifiedAtomStereoCenters
0\n_pdbe_chem_comp_rdkit_properties.labuteASA
28.307\n_pdbe_chem_comp_rdkit_properties.tpsa
86.250\n_pdbe_chem_comp_rdkit_properties.CrippenClogP -
2.825\n_pdbe_chem_comp_rdkit_properties.CrippenMR
7.606\n_pdbe_chem_comp_rdkit_properties.chi0v
2.975\n_pdbe_chem_comp_rdkit_properties.chi1v
2.191\n_pdbe_chem_comp_rdkit_properties.chi2v
0\n_pdbe_chem_comp_rdkit_properties.chi3v
0\n_pdbe_chem_comp_rdkit_properties.chi4v
0\n_pdbe_chem_comp_rdkit_properties.chi0n
2.080\n_pdbe_chem_comp_rdkit_properties.chi1n
0.730\n_pdbe_chem_comp_rdkit_properties.chi2n
0\n_pdbe_chem_comp_rdkit_properties.chi3n
0\n_pdbe_chem_comp_rdkit_properties.chi4n
0\n_pdbe_chem_comp_rdkit_properties.hallKierAlpha
0.110\n_pdbe_chem_comp_rdkit_properties.kappa1
5.110\n_pdbe_chem_comp_rdkit_properties.kappa2

1.065\n_pdbe_chem_comp_rdkit_properties.kappa3
1512.242\n_pdbe_chem_comp_rdkit_properties.Phi
1.088\n#\nloop_\n_pdbe_chem_comp_external_mappings.comp_id\n_pdbe_chem_comp_external_mappings.source\n_pdbe_chem_comp_external_mappings.resource\n_pdbe_chem_comp_external_mappings.resource_id\nPO4 UniChem DrugBank DB14523\nPO4 UniChem ChEBI 18367\nPO4 UniChem eMolecules 882178\nPO4 UniChem fdasrs NK08V8K8HR\nPO4 UniChem NMRShiftDB 30000818\nPO4 UniChem BindingDb 50155537\nPO4 UniChem MetaboLights MTBLC18367\nPO4 UniChem BRENDA 21671\nPO4 UniChem BRENDA 867\nPO4 UniChem ChemicalBook CB3409949\nPO4 UniChem ChemicalBook CB7191961\nPO4 UniChem rxnorm 'PHOSPHATE ION'\nPO4 UniChem 'Probes And Drugs' PD094531\nPO4 UniChem CCDC EXIZIX\nPO4 UniChem 'EPA CompTox Dashboard' DTXSID7039672\nPO4 UniChem 'PubChem TPHARMA' 15218698\nPO4 UniChem PubChem 1061\nPO4 UniChem ACTor 14265-44-2\nPO4 UniChem Nikkaji J215.970H\nPO4 UniChem Nikkaji
J351.173A\n#\nloop_\n_pdbe_chem_comp_rdkit_conformer.comp_id\n_pdbe_chem_comp_rdkit_conformer.atom_id\n_pdbe_chem_comp_rdkit_conformer.Cartn_x_rdkit\n_pdbe_chem_comp_rdkit_conformer.Cartn_y_rdkit\n_pdbe_chem_comp_rdkit_conformer.Cartn_z_rdkit\n_pdbe_chem_comp_rdkit_conformer.rdkit_method\n_pdbe_chem_comp_rdkit_conformer.rdkit_ordinal\nPO4 P -0.003 0.002 0.036 ETKDGv3 1\nPO4 O1 -0.117 0.079 1.541 ETKDGv3 2\nPO4 O4 -0.383 1.506 -0.636 ETKDGv3 5\n#\ndata_PA1-0\n#\n_chem_comp.id
PA1\n_chem_comp.name 2-amino-2-deoxy-alpha-D-glucopyranose\n_chem_comp.type 'D-saccharide, alpha linking'\n_chem_comp.pdbx_type ATOMS\n_chem_comp.formula 'C6 H13 N O5'\n_chem_comp.mon_nstd_parent_comp_id ?\n_chem_comp.pdbx_synonyms\n'alpah-D-glucosamine; 2-amino-2-deoxy-alpha-D-glucose; 2-amino-2-deoxy-D-glucose; 2-amino-2-deoxy-glucose'\n_chem_comp.pdbx_formal_charge 0\n_chem_comp.pdbx_initial_date 1999-07-08\n_chem_comp.pdbx_modified_date 2021-08-24\n_chem_comp.pdbx_ambiguous_flag N\n_chem_comp.pdbx_release_status REL\n_chem_comp.pdbx_replaced_by ?\n_chem_comp.pdbx_replaces ?\n_chem_comp.formula_weight
179.171\n_chem_comp.one_letter_code ?\n_chem_comp.three_letter_code ?\n_chem_comp.pdbx_model_coordinates_details ?\n_chem_comp.pdbx_model_coordinates_missing_flag
N\n_chem_comp.pdbx_ideal_coordinates_details ?\n_chem_comp.pdbx_ideal_coordinates_missing_flag N\n_chem_comp.pdbx_model_coordinates_db_code 1PBR\n_chem_comp.pdbx_subcomponent_list ?\n_chem_comp.pdbx_processing_site
RCSB\n#\nloop_\n_pdbx_chem_comp_synonyms.ordinal\n_pdbx_chem_comp_synonyms.comp_id\n_pdbx_chem_comp_synonyms.name\n_pdbx_chem_comp_synonyms.provenance\n_pdbx_chem_comp_synonyms.type\n1 PA1 alpha-D-glucosamine PDB ?\n2 PA1 2-amino-2-deoxy-alpha-D-glucose PDB ?\n3 PA1 2-amino-2-deoxy-D-glucose PDB ?\n4 PA1 2-amino-2-deoxy-glucose
PDB ?\n#\nloop_\n_chem_comp_atom.comp_id\n_chem_comp_atom.atom_id\n_chem_comp_atom.alt_atom_id\n_chem_comp_atom.type_symbol\n_chem_comp_atom.charge\n_chem_comp_atom.pdbx_align\n_chem_comp_atom.pdbx_aromatic_flag\n_chem_comp_atom.pdbx_leaving_atom_flag\n_chem_comp_atom.pdbx_stereo_config\n_chem_comp_atom.pdbx_backbone_atom_flag\n_chem_comp_atom.pdbx_n_terminal_atom_flag\n_chem_comp_atom.pdbx_c_terminal_atom_flag\n_chem_comp_atom.model_Cartn_x\n_chem_comp_atom.model_Cartn_y\n_chem_comp_a

tom.model_Cartn_z\n_chem_comp_atom.pdbx_model_Cartn_x_ideal\n_chem_comp_atom.pdbx_model_Cartn_y_ideal\n_chem_comp_atom.pdbx_model_Cartn_z_ideal\n_chem_comp_atom.pdbx_component_atom_id\n_chem_comp_atom.pdbx_component_comp_id\n_chem_comp_atom.pdbx_ordinal\nPA1 C1 C1 C 0 1 N N S N N N 1.980 -0.879 43.999 1.564 0.223 0.505 C1 PA1 1\nPA1 O1 O1 O 0 1 N Y N N N N 1.764 -2.297 44.213 1.770 -1.188 0.422 O1 PA1 2\nPA1 C2 C2 C 0 1 N N R N N N 3.316 -0.407 44.685 0.529 0.524 1.591 C2 PA1 3\nPA1 N2 N2 N 0 1 N N N N N N 4.453 -1.352 44.535 1.026 0.047 2.889 N2 PA1 4\nPA1 C3 C3 C 0 1 N N R N N N 3.131 -0.192 46.199 -0.780 -0.193 1.249 C3 PA1 5\nPA1 O3 O3 O 0 1 N N N N N N 4.258 0.481 46.756 -1.802 0.195 2.169 O3 PA1 6\nPA1 C4 C4 C 0 1 N N S N N N 1.858 0.617 46.490 -1.194 0.194 -0.174 C4 PA1 7\nPA1 O4 O4 O 0 1 N N N N N N 1.727 0.843 47.890 -2.330 -0.576 -0.571 O4 PA1 8\nPA1 C5 C5 C 0 1 N N R N N N 0.618 -0.077 45.884 -0.029 -0.081 -1.127 C5 PA1 9\nPA1 O5 O5 O 0 1 N N N N N N 0.822 -0.127 44.456 1.098 0.707 -0.753 O5 PA1 10\nPA1 C6 C6 C 0 1 N N N N N N -0.699 0.652 46.145 -0.447 0.274 -2.556 C6 PA1 11\nPA1 O6 O6 O 0 1 N N N N N N -1.035 0.630 47.526 0.636 0.011 -3.449 O6 PA1 12\nPA1 H1 H1 H 0 1 N N N N N N 2.009 -0.659 42.921 2.505 0.713 0.755 H1 PA1 13\nPA1 HO1 HO1 H 0 1 N Y N N N N 0.957 -2.581 43.799 2.427 -1.334 -0.271 HO1 PA1 14\nPA1 H2 H2 H 0 1 N N N N N N 3.604 0.559 44.233 0.354 1.599 1.640 H2 PA1 15\nPA1 HN21 HN21 H 0 0 N N N N N N 5.282 -0.986 45.017 1.886 0.543 3.070 HN21 PA1 16\nPA1 HN22 HN22 H 0 0 N Y N N N N 4.235 -2.242 44.998 0.363 0.355 3.584 HN22 PA1 17\nPA1 H3 H3 H 0 1 N N N N N N 3.044 -1.188 46.649 -0.629 -1.271 1.304 H3 PA1 18\nPA1 HO3 HO3 H 0 1 N Y N N N N 4.298 1.336 46.320 -1.501 -0.067 3.049 HO3 PA1 19\nPA1 H4 H4 H 0 1 N N N N N N 1.966 1.564 45.945 -1.446 1.255 -0.203 H4 PA1 20\nPA1 HO4 HO4 H 0 1 N Y N N N N 1.644 -0.026 48.291 -2.552 -0.304 -1.472 HO4 PA1 21\nPA1 H5 H5 H 0 1 N N N N N N 0.519 -1.112 46.244 0.235 -1.137 -1.080 H5 PA1 22\nPA1 H61 H61 H 0 1 N N N N N N -1.503 0.179 45.555 -1.309 -0.327 -2.843 H61 PA1 23\nPA1 H62 H62 H 0 1 N N N N N N -0.639 1.691 45.797 -0.709 1.331 -2.605 H62 PA1 24\nPA1 HO6 HO6 H 0 1 N Y N N N N -1.106 -0.297 47.767 0.332 0.248 -4.335 HO6 PA1
25\n#\nloop_\n_chem_comp_bond.comp_id\n_chem_comp_bond.atom_id_1\n_chem_comp_bond.atom_id_2\n_chem_comp_bond.value_order\n_chem_comp_bond.pdbx_aromatic_flag\n_chem_comp_bond.pdbx_stereo_config\n_chem_comp_bond.pdbx_ordinal\nPA1 C1 O1 SING N N 1\nPA1 C1 C2 SING N N 2\nPA1 C1 O5 SING N N 3\nPA1 C1 H1 SING N N 4\nPA1 O1 HO1 SING N N 5\nPA1 C2 N2 SING N N 6\nPA1 C2 C3 SING N N 7\nPA1 C2 H2 SING N N 8\nPA1 N2 HN21 SING N N 9\nPA1 N2 HN22 SING N N 10\nPA1 C3 O3 SING N N 11\nPA1 C3 C4 SING N N 12\nPA1 C3 H3 SING N N 13\nPA1 O3 HO3 SING N N 14\nPA1 C4 O4 SING N N 15\nPA1 C4 C5 SING N N 16\nPA1 C4 H4 SING N N 17\nPA1 O4 HO4 SING N N 18\nPA1 C5 O5 SING N N 19\nPA1 C5 C6 SING N N 20\nPA1 C5 H5 SING N N 21\nPA1 C6 O6 SING N N 22\nPA1 C6 H61 SING N N 23\nPA1 C6 H62 SING N N 24\nPA1 O6 HO6 SING N N
25\n#\nloop_\n_pdbx_chem_comp_descriptor.comp_id\n_pdbx_chem_comp_descriptor.type\n_pdbx_chem_comp_descriptor.program\n_pdbx_chem_comp_descriptor.program_version\n_pdbx_chem_comp_descriptor.descriptor\nPA1 SMILES ACDLabs 10.04 OC1C(O)C(OC(O)C1N)CO\nPA1 SMILES_CANONICAL CACTVS 3.341 N[C@H]1[C@@H](O)O[C@H](CO)[C@@H](O)[C@@H]1O\nPA1 SMILES CACTVS 3.341 N[CH]1[CH](O)O[CH](CO)[CH](O)[CH]1O\nPA1 SMILES_CANONICAL 'OpenEye OEToolkits' 1.5.0

C([C@@H]1[C@H]([C@@H]([C@H]([C@H](O1)O)N)O)O)O\nPA1 SMILES 'OpenEye OEToolkits' 1.5.0 C(C1C(C(C(C(O1)O)N)O)O)O\nPA1 InChI InChI 1.03 InChI=1S/C6H13NO5/c7-3-5(10)4(9)2(1-8)12-6(3)11/h2-6,8-11H,1,7H2/t2-,3-,4-,5-,6+/m1/s1\nPA1 InChIKey InChI 1.03 MSWZFWKMSRAUBD-UKFBFLRUSA-N\n#\nloop_\n_pdbx_chem_comp_identifier.comp_id\n_pdbx_chem_comp_identifier.type\n_pdbx_chem_comp_identifier.program\n_pdbx_chem_comp_identifier.program_version\n_pdbx_chem_comp_identifier.identifier\nPA1 'SYSTEMATIC NAME' ACDLabs 10.04 2-amino-2-deoxy-alpha-D-glucopyranose\nPA1 'SYSTEMATIC NAME' 'OpenEye OEToolkits' 1.5.0 (2S,3R,4R,5S,6R)-3-amino-6-(hydroxymethyl)oxane-2,4,5-triol\nPA1 'CONDENSED IUPAC CARBOHYDRATE SYMBOL' GMML 1.0 DGlcpNa\nPA1 'COMMON NAME' GMML 1.0 a-D-glucopyranosamine\nPA1 'IUPAC CARBOHYDRATE SYMBOL' PDB-CARE 1.0 a-D-GlcpN\nPA1 'SNFG CARBOHYDRATE SYMBOL' GMML 1.0 GlcN\n#\nloop_\n_pdbx_chem_comp_feature.comp_id\n_pdbx_chem_comp_feature.type\n_pdbx_chem_comp_feature.value\n_pdbx_chem_comp_feature.source\n_pdbx_chem_comp_feature.support\nPA1 'CARBOHYDRATE ISOMER' D PDB ?\nPA1 'CARBOHYDRATE RING' pyranose PDB ?\nPA1 'CARBOHYDRATE ANOMER' alpha PDB ?\nPA1 'CARBOHYDRATE PRIMARY CARBONYL GROUP' aldose PDB ?\n#\nloop_\n_pdbx_chem_comp_audit.comp_id\n_pdbx_chem_comp_audit.action_type\n_pdbx_chem_comp_audit.date\n_pdbx_chem_comp_audit.processing_site\nPA1 'Create component' 1999-07-08 RCSB\nPA1 'Modify descriptor' 2011-06-04 RCSB\nPA1 'Modify leaving atom flag' 2012-06-13 RCSB\nPA1 'Other modification' 2019-08-12 RCSB\nPA1 'Other modification' 2019-12-19 RCSB\nPA1 'Other modification' 2020-07-03 RCSB\nPA1 'Modify synonyms' 2020-07-17 RCSB\nPA1 'Modify synonyms' 2021-08-24 RCSB\n#\nloop_\n_pdbe_chem_comp_synonyms.comp_id\n_pdbe_chem_comp_synonyms.name\n_pdbe_chem_comp_synonyms.provenance\n_pdbe_chem_comp_synonyms.type\nPA1 alpha-D-glucosamine wwPDB ?\nPA1 2-amino-2-deoxy-alpha-D-glucose wwPDB ?\nPA1 2-amino-2-deoxy-D-glucose wwPDB ?\nPA1 2-amino-2-deoxy-glucose wwPDB ?\n#\nloop_\n_software.name\n_software.version\n_software.description\nrdkit 2023.09.6 'Core functionality.'\npdbeccdutils 0.8.6 'Wrapper to provide 2D templates and molecular fragments.'\n#\nloop_\n_pdbe_chem_comp_atom_depiction.comp_id\n_pdbe_chem_comp_atom_depiction.atom_id\n_pdbe_chem_comp_atom_depiction.element\n_pdbe_chem_comp_atom_depiction.model_Cartn_x\n_pdbe_chem_comp_atom_depiction.model_Cartn_y\n_pdbe_chem_comp_atom_depiction.pdbx_ordinal\nPA1 C1 C 7.702 0.750 1\nPA1 O1 O 9.002 1.500 2\nPA1 C2 C 7.702 -0.750 3\nPA1 N2 N 9.002 -1.500 4\nPA1 C3 C 6.404 -1.500 5\nPA1 O3 O 6.404 -3.000 6\nPA1 C4 C 5.104 -0.750 7\nPA1 O4 O 3.805 -1.500 8\nPA1 C5 C 5.104 0.750 9\nPA1 O5 O 6.404 1.500 10\nPA1 C6 C 3.805 1.500 11\nPA1 O6 O 3.805 3.000 12\n#\nloop_\n_pdbe_chem_comp_bond_depiction.comp_id\n_pdbe_chem_comp_bond_depiction.atom_id_1\n_pdbe_chem_comp_bond_depiction.atom_id_2\n_pdbe_chem_comp_bond_depiction.value_order\n_pdbe_chem_comp_bond_depiction.bond_dir\n_pdbe_chem_comp_bond_depiction.pdbx_ordinal\nPA1 C1 O1 SINGLE BEGINDASH 1\nPA1 C1 C2 SINGLE NONE 2\nPA1 C1 O5 SINGLE NONE 3\nPA1 C2 N2 SINGLE BEGINDASH 4\nPA1 C2 C3 SINGLE NONE 5\nPA1 C3 O3 SINGLE BEGINWEDGE 6\nPA1 C3 C4 SINGLE NONE 7\nPA1 C4 O4 SINGLE BEGINDASH 8\nPA1 C4 C5 SINGLE NONE 9\nPA1 C5 O5 SINGLE NONE 10\nPA1 C5

C6 SINGLE BEGINWEDGE 11\nPA1 C6 O6 SINGLE NONE
12\n#\nloop_\n_pdbe_chem_comp_substructure.comp_id\n_pdbe_chem_comp_su
bstructure.substructure_name\n_pdbe_chem_comp_substructure.id\n_pdbe_c
hem_comp_substructure.substructure_type\n_pdbe_chem_comp_substructure.
substructure_smiles\n_pdbe_chem_comp_substructure.substructure_inchis\
n_pdbe_chem_comp_substructure.substructure_inchikeys\nPA1
MurckoScaffold S1 scaffold C1CCOCC1 InChI=1S/C5H10O/c1-2-4-6-5-3-1/h1-
5H2 DHXVGJBLRPWPCS-UHFFFAOYSA-N\nPA1 pyranose F1 fragment OC1CCCCO1
InChI=1S/C5H10O2/c6-5-3-1-2-4-7-5/h5-6H,1-4H2 CELWCAITJAEQNL-
UHFFFAOYSA-
N\n#\nloop_\n_pdbe_chem_comp_substructure_mapping.comp_id\n_pdbe_chem_
comp_substructure_mapping.atom_id\n_pdbe_chem_comp_substructure_mappin
g.substructure_id\n_pdbe_chem_comp_substructure_mapping.substructure_o
rdinal\nPA1 C1 S1 1\nPA1 C2 S1 1\nPA1 C3 S1 1\nPA1 C4 S1 1\nPA1 C5 S1
1\nPA1 O5 S1 1\nPA1 C4 F1 1\nPA1 C5 F1 1\nPA1 O5 F1 1\nPA1 C1 F1
1\nPA1 C2 F1 1\nPA1 C3 F1 1\nPA1 O1 F1
1\n#\n_pdbe_chem_comp_rdkit_properties.comp_id
PA1\n_pdbe_chem_comp_rdkit_properties.exactmw
179.079\n_pdbe_chem_comp_rdkit_properties.amw
179.172\n_pdbe_chem_comp_rdkit_properties.lipinskiHBA
6\n_pdbe_chem_comp_rdkit_properties.lipinskiHBD
6\n_pdbe_chem_comp_rdkit_properties.NumRotatableBonds
6\n_pdbe_chem_comp_rdkit_properties.NumHBD
5\n_pdbe_chem_comp_rdkit_properties.NumHBA
6\n_pdbe_chem_comp_rdkit_properties.NumHeavyAtoms
12\n_pdbe_chem_comp_rdkit_properties.NumAtoms
25\n_pdbe_chem_comp_rdkit_properties.NumHeteroatoms
6\n_pdbe_chem_comp_rdkit_properties.NumAmideBonds
0\n_pdbe_chem_comp_rdkit_properties.FractionCSP3
1\n_pdbe_chem_comp_rdkit_properties.NumRings
1\n_pdbe_chem_comp_rdkit_properties.NumAromaticRings
0\n_pdbe_chem_comp_rdkit_properties.NumAliphaticRings
1\n_pdbe_chem_comp_rdkit_properties.NumSaturatedRings
1\n_pdbe_chem_comp_rdkit_properties.NumHeterocycles
1\n_pdbe_chem_comp_rdkit_properties.NumAromaticHeterocycles
0\n_pdbe_chem_comp_rdkit_properties.NumSaturatedHeterocycles
1\n_pdbe_chem_comp_rdkit_properties.NumAliphaticHeterocycles
1\n_pdbe_chem_comp_rdkit_properties.NumSpiroAtoms
0\n_pdbe_chem_comp_rdkit_properties.NumBridgeheadAtoms
0\n_pdbe_chem_comp_rdkit_properties.NumAtomStereoCenters
5\n_pdbe_chem_comp_rdkit_properties.NumUnspecifiedAtomStereoCenters
0\n_pdbe_chem_comp_rdkit_properties.labuteASA
87.916\n_pdbe_chem_comp_rdkit_properties.tpsa
116.170\n_pdbe_chem_comp_rdkit_properties.CrippenClogP -
3.255\n_pdbe_chem_comp_rdkit_properties.CrippenMR
37.955\n_pdbe_chem_comp_rdkit_properties.chi0v
5.488\n_pdbe_chem_comp_rdkit_properties.chi1v
2.698\n_pdbe_chem_comp_rdkit_properties.chi2v
1.108\n_pdbe_chem_comp_rdkit_properties.chi3v
1.108\n_pdbe_chem_comp_rdkit_properties.chi4v
0.558\n_pdbe_chem_comp_rdkit_properties.chi0n
18.488\n_pdbe_chem_comp_rdkit_properties.chi1n

8.726\n_pdbe_chem_comp_rdkit_properties.chi2n
1.108\n_pdbe_chem_comp_rdkit_properties.chi3n
1.108\n_pdbe_chem_comp_rdkit_properties.chi4n
0.558\n_pdbe_chem_comp_rdkit_properties.hallKierAlpha -
0.240\n_pdbe_chem_comp_rdkit_properties.kappa1
2.221\n_pdbe_chem_comp_rdkit_properties.kappa2
3.649\n_pdbe_chem_comp_rdkit_properties.kappa3
1.582\n_pdbe_chem_comp_rdkit_properties.Phi
0.675\n#\nloop_\n_pdbe_chem_comp_external_mappings.comp_id\n_pdbe_chem_comp_external_mappings.source\n_pdbe_chem_comp_external_mappings.resource\n_pdbe_chem_comp_external_mappings.resource_id\nPA1 UniChem ChEBI 44678\nPA1 UniChem ZINC ZINC000003860469\nPA1 UniChem atlas glucosamine\nPA1 UniChem atlas glucosamine,\nPA1 UniChem atlas 'glucosamine, steady state'\nPA1 UniChem fdasrs S3BL0640UV\nPA1 UniChem NMRShiftDB 20212431\nPA1 UniChem LINCS LSM-36429\nPA1 UniChem MetaboLights MTBLC44678\nPA1 UniChem 'Probes And Drugs' PD057482\nPA1 UniChem Nikkaji J361.550B\nPA1 UniChem SureChEMBL SCHEMBL429222\nPA1 UniChem 'PubChem TPHARMA' 15219649\nPA1 UniChem PubChem 445621\nPA1 UniChem Mcule MCULE-
9890197257\n#\nloop_\n_pdbe_chem_comp_rdkit_conformer.comp_id\n_pdbe_chem_comp_rdkit_conformer.atom_id\n_pdbe_chem_comp_rdkit_conformer.Cartn_x_rdkit\n_pdbe_chem_comp_rdkit_conformer.Cartn_y_rdkit\n_pdbe_chem_comp_rdkit_conformer.Cartn_z_rdkit\n_pdbe_chem_comp_rdkit_conformer.rdkit_method\n_pdbe_chem_comp_rdkit_conformer.rdkit_ordinal\nPA1 C1 -0.729 1.112 -0.639 ETKDGv3 1\nPA1 O1 -1.440 2.298 -0.895 ETKDGv3 2\nPA1 C2 -1.674 -0.028 -0.166 ETKDGv3 3\nPA1 N2 -2.758 0.490 0.676 ETKDGv3 4\nPA1 C3 -0.873 -1.104 0.617 ETKDGv3 5\nPA1 O3 -1.467 -2.371 0.479 ETKDGv3 6\nPA1 C4 0.600 -1.130 0.174 ETKDGv3 7\nPA1 O4 1.294 -2.094 0.925 ETKDGv3 8\nPA1 C5 1.228 0.280 0.391 ETKDGv3 9\nPA1 O5 0.291 1.354 0.318 ETKDGv3 10\nPA1 C6 2.409 0.533 -0.563 ETKDGv3 11\nPA1 O6 2.969 1.797 -0.326 ETKDGv3 12\nPA1 H1 -0.273 0.810 -1.609 ETKDGv3 13\nPA1 HO1 -1.578 2.756 -0.025 ETKDGv3 14\nPA1 H2 -2.121 -0.490 -1.077 ETKDGv3 15\nPA1 HN21 -3.322 -0.319 1.025 ETKDGv3 16\nPA1 HN22 -3.412 1.040 0.073 ETKDGv3 17\nPA1 H3 -0.886 -0.843 1.699 ETKDGv3 18\nPA1 HO3 -1.394 -2.637 -0.475 ETKDGv3 19\nPA1 H4 0.631 -1.403 -0.908 ETKDGv3 20\nPA1 HO4 2.123 -2.307 0.422 ETKDGv3 21\nPA1 H5 1.637 0.299 1.425 ETKDGv3 22\nPA1 H61 3.184 -0.257 -0.435 ETKDGv3 23\nPA1 H62 2.064 0.494 -1.617 ETKDGv3 24\nPA1 HO6 3.500 1.720 0.509 ETKDGv3 25\n#\ndata_IPD-6\n#\n_chem_comp.id IPD\n_chem_comp.name D-MYO-INOSITOL-1-PHOSPHATE\n_chem_comp.type NON-POLYMER\n_chem_comp.pdbx_type HETAIN\n_chem_comp.formula 'C6 H11 O9 P'\n_chem_comp.mon_nstd_parent_comp_id ?\n_chem_comp.pdbx_synonyms ?\n_chem_comp.pdbx_formal_charge -2\n_chem_comp.pdbx_initial_date 1999-07-08\n_chem_comp.pdbx_modified_date 2024-09-27\n_chem_comp.pdbx_ambiguous_flag N\n_chem_comp.pdbx_release_status REL\n_chem_comp.pdbx_replaced_by ?\n_chem_comp.pdbx_replaces ?\n_chem_comp.formula_weight
258.120\n_chem_comp.one_letter_code ?\n_chem_comp.three_letter_code IPD\n_chem_comp.pdbx_model_coordinates_details ?\n_chem_comp.pdbx_model_coordinates_missing_flag
N\n_chem_comp.pdbx_ideal_coordinates_details
Corina\n_chem_comp.pdbx_ideal_coordinates_missing_flag

N\n_chem_comp.pdbx_model_coordinates_db_code 1AWB\n_chem_comp.pdbx_subcomponent_list ?\n_chem_comp.pdbx_processing_site RCSB\n_chem_comp.pdbx_pcm Y\n#\nloop_\n_chem_comp_atom.comp_id\n_chem_comp_atom.atom_id\n_chem_comp_atom.alt_atom_id\n_chem_comp_atom.type_symbol\n_chem_comp_atom.charge\n_chem_comp_atom.pdbx_align\n_chem_comp_atom.pdbx_aromatic_flag\n_chem_comp_atom.pdbx_leaving_atom_flag\n_chem_comp_atom.pdbx_stereo_config\n_chem_comp_atom.pdbx_backbone_atom_flag\n_chem_comp_atom.pdbx_n_terminal_atom_flag\n_chem_comp_atom.pdbx_c_terminal_atom_flag\n_chem_comp_atom.model_Cartn_x\n_chem_comp_atom.model_Cartn_y\n_chem_comp_atom.model_Cartn_z\n_chem_comp_atom.pdbx_model_Cartn_x_ideal\n_chem_comp_atom.pdbx_model_Cartn_y_ideal\n_chem_comp_atom.pdbx_model_Cartn_z_ideal\n_chem_comp_atom.pdbx_component_atom_id\n_chem_comp_atom.pdbx_component_comp_id\n_chem_comp_atom.pdbx_ordinal\nIPD C1 C1 C 0 1 N N N N N N 5.269 52.561 12.799 -0.197 -0.074 -0.062 C1 IPD 1\nIPD C2 C2 C 0 1 N N R N N N 4.996 53.056 14.225 0.617 -1.345 0.188 C2 IPD 2\nIPD C3 C3 C 0 1 N N R N N N 4.405 51.889 15.041 1.962 -1.240 -0.534 C3 IPD 3\nIPD C4 C4 C 0 1 N N N N N N 5.413 50.728 15.130 2.732 -0.029 -0.004 C4 IPD 4\nIPD C5 C5 C 0 1 N N S N N N 5.751 50.225 13.715 1.919 1.242 -0.254 C5 IPD 5\nIPD C6 C6 C 0 1 N N R N N N 6.280 51.405 12.876 0.574 1.138 0.468 C6 IPD 6\nIPD P1 P1 P 0 1 N N N N N N 5.242 54.263 10.752 -2.867 -0.110 -0.156 P1 IPD 7\nIPD O1 O1 O 0 1 N N N N N N 5.864 53.675 12.063 -1.453 -0.171 0.612 O1 IPD 8\nIPD O2 O2 O 0 1 N N N N N N 6.230 53.533 14.822 0.840 -1.499 1.591 O2 IPD 9\nIPD O3 O3 O 0 1 N N N N N N 4.111 52.348 16.358 2.722 -2.428 -0.300 O3 IPD 10\nIPD O4 O4 O 0 1 N N N N N N 4.869 49.645 15.912 3.988 0.069 -0.678 O4 IPD 11\nIPD O5 O5 O 0 1 N N N N N N 6.780 49.213 13.826 2.638 2.373 0.241 O5 IPD 12\nIPD O7 O7 O 0 1 N N N N N N 6.119 55.318 10.238 -2.942 1.203 -1.008 O7 IPD 14\nIPD O8 O8 O -1 1 N N N N N N 5.035 53.142 9.799 -3.004 -1.353 -1.098 O8 IPD 15\nIPD O9 O9 O -1 1 N N N N N N 3.877 54.737 11.108 -4.031 -0.118 0.893 O9 IPD 16\nIPD H1 H1 H 0 1 N N N N N N 4.336 52.221 12.327 -0.367 0.044 -1.133 H1 IPD 17\nIPD H2 H2 H 0 1 N N N N N N 4.258 53.870 14.185 0.068 -2.208 -0.189 H2 IPD 18\nIPD H3 H3 H 0 1 N N N N N N 3.491 51.532 14.543 1.792 -1.123 -1.604 H3 IPD 19\nIPD H4 H4 H 0 1 N N N N N N 6.336 51.101 15.599 2.902 -0.147 1.066 H4 IPD 20\nIPD H5 H5 H 0 1 N N N N N N 4.846 49.814 13.244 1.748 1.360 -1.324 H5 IPD 21\nIPD H6 H6 H 0 1 N N N N N N 7.195 51.782 13.355 0.744 1.020 1.538 H6 IPD 22\nIPD HO2 HO2 H 0 1 N N N N N N 6.058 53.839 15.705 1.351 -2.286 1.824 HO2 IPD 23\nIPD HO3 HO3 H 0 1 N N N N N N 3.746 51.635 16.868 3.589 -2.431 -0.728 HO3 IPD 24\nIPD HO4 HO4 H 0 1 N N N N N N 5.502 48.938 15.957 4.531 0.816 -0.392 HO4 IPD 25\nIPD HO5 HO5 H 0 1 N N N N N N 7.002 48.890 12.961 3.503 2.502 -0.173 HO5 IPD 26\n#\nloop_\n_chem_comp_bond.comp_id\n_chem_comp_bond.atom_id_1\n_chem_comp_bond.atom_id_2\n_chem_comp_bond.value_order\n_chem_comp_bond.pdbx_aromatic_flag\n_chem_comp_bond.pdbx_stereo_config\n_chem_comp_bond.pdbx_ordinal\nIPD C1 C2 SING N N 1\nIPD C1 C6 SING N N 2\nIPD C1 O1 SING N N 3\nIPD C1 H1 SING N N 4\nIPD C2 C3 SING N N 5\nIPD C2 O2 SING N N 6\nIPD C2 H2 SING N N 7\nIPD C3 C4 SING N N 8\nIPD C3 O3 SING N N 9\nIPD C3 H3 SING N N 10\nIPD C4 C5 SING N N 11\nIPD C4 O4 SING N N 12\nIPD C4 H4 SING N N 13\nIPD C5 C6 SING N N 14\nIPD C5 O5 SING N N 15\nIPD C5 H5 SING N N 16\nIPD C6 H6 SING N N 18\nIPD P1 O1 SING N N 19\nIPD P1 O7 DOUB N N 20\nIPD P1 O8 SING N N 21\nIPD P1 O9 SING N N

22\nIPD O2 HO2 SING N N 23\nIPD O3 HO3 SING N N 24\nIPD O4 HO4 SING N N 25\nIPD O5 HO5 SING N N 26\n#\nloop_\n_pdbx_chem_comp_descriptor.comp_id\n_pdbx_chem_comp_descriptor.type\n_pdbx_chem_comp_descriptor.program\n_pdbx_chem_comp_descriptor.program_version\n_pdbx_chem_comp_descriptor.descriptor\nIPD SMILES ACDLabs 12.01 'O=P([O-])([O-])OC1C(O)C(O)C(O)C(O)C1O'\nIPD InChI InChI 1.03 'InChI=1S/C6H13O9P/c7-1-2(8)4(10)6(5(11)3(1)9)15-16(12,13)14/h1-11H,(H2,12,13,14)/p-2/t1-,2-,3+,4-,5-,6-/m1/s1'\nIPD InChIKey InChI 1.03 INAPMGSXUVUWAF-UOTPTPDRSA-L\nIPD SMILES_CANONICAL CACTVS 3.370 'O[C@@H]1[C@@H](O)[C@@H](O)[C@@H](O[P]([O-])([O-])=O)[C@H](O)[C@H]1O'\nIPD SMILES CACTVS 3.370 'O[CH]1[CH](O)[CH](O)[CH](O[P]([O-])([O-])=O)[CH](O)[CH]1O'\nIPD SMILES_CANONICAL 'OpenEye OEToolkits' 1.7.6 '[C@H]1([C@H](C([C@@H]([C@@H](C1O)O)O)OP(=O)([O-])[O-])O)O'\nIPD SMILES 'OpenEye OEToolkits' 1.7.6 'C1(C(C(C(C(C1O)O)OP(=O)([O-])[O-])O)O)O'\n#\nloop_\n_pdbx_chem_comp_identifier.comp_id\n_pdbx_chem_comp_identifier.type\n_pdbx_chem_comp_identifier.program\n_pdbx_chem_comp_identifier.program_version\n_pdbx_chem_comp_identifier.identifier\nIPD 'SYSTEMATIC NAME' ACDLabs 12.01 '(1S,2R,3R,4S,5S,6R)-2,3,4,5,6-pentahydroxycyclohexyl phosphate'\nIPD 'SYSTEMATIC NAME' 'OpenEye OEToolkits' 1.7.6 '[(2R,3R,5S,6R)-2,3,4,5,6-pentakis(oxidanyl)cyclohexyl] phosphate'\n#\nloop_\n_pdbx_chem_comp_audit.comp_id\n_pdbx_chem_comp_audit.action_type\n_pdbx_chem_comp_audit.date\n_pdbx_chem_comp_audit.processing_site\nIPD 'Create component' 1999-07-08 RCSB\nIPD 'Modify descriptor' 2011-06-04 RCSB\nIPD 'Other modification' 2013-03-12 RCSB\nIPD 'Modify PCM' 2024-09-27 PDBE\n#\n_pdbx_chem_comp_pcm.pcm_id 1\n_pdbx_chem_comp_pcm.comp_id IPD\n_pdbx_chem_comp_pcm.modified_residue_id HIS\n_pdbx_chem_comp_pcm.type None\n_pdbx_chem_comp_pcm.category 'Covalent chemical modification'\n_pdbx_chem_comp_pcm.position 'Amino-acid side chain'\n_pdbx_chem_comp_pcm.polypeptide_position 'Any position'\n_pdbx_chem_comp_pcm.comp_id_linking_atom P1\n_pdbx_chem_comp_pcm.modified_residue_id_linking_atom NE2\n_pdbx_chem_comp_pcm.uniprot_specific_ptm_accession ?\n_pdbx_chem_comp_pcm.uniprot_generic_ptm_accession ?\n#\nloop_\n_software.name\n_software.version\n_software.description\nrdkit 2023.09.6 'Core functionality.'\npdbeccdutils 0.8.6 'Wrapper to provide 2D templates and molecular fragments.'\n#\nloop_\n_pdbe_chem_comp_atom_depiction.comp_id\n_pdbe_chem_comp_atom_depiction.atom_id\n_pdbe_chem_comp_atom_depiction.element\n_pdbe_chem_comp_atom_depiction.model_Cartn_x\n_pdbe_chem_comp_atom_depiction.model_Cartn_y\n_pdbe_chem_comp_atom_depiction.pdbx_ordinal\nIPD C1 C -0.631 -0.084 1\nIPD C2 C 0.672 0.662 2\nIPD C3 C 1.968 -0.087 3\nIPD C4 C 1.959 -1.586 4\nIPD C5 C 0.658 -2.337 5\nIPD C6 C -0.635 -1.584 6\nIPD P1 P -1.983 2.137 7\nIPD O1 O -1.948 0.651 8\nIPD O2 O 0.689 2.175 9\nIPD O3 O 3.269 0.658 10\nIPD O4 O 3.257 -2.341 11\nIPD O5 O 0.655 -3.836 12\nIPD O7 O -2.001 3.635 14\nIPD O8 O -0.511 2.156 15\nIPD O9 O -3.480 2.111 16\n#\nloop_\n_pdbe_chem_comp_bond_depiction.comp_id\n_pdbe_chem_comp_bond_depiction.atom_id_1\n_pdbe_chem_comp_bond_depiction.atom_id_2\n_p

dbe_chem_comp_bond_depiction.value_order\n_pdbe_chem_comp_bond_depiction.bond_dir\n_pdbe_chem_comp_bond_depiction.pdbx_ordinal\nIPD C1 C2 SINGLE NONE 1\nIPD C1 C6 SINGLE NONE 2\nIPD C1 O1 SINGLE BEGINDASH 3\nIPD C2 C3 SINGLE NONE 4\nIPD C2 O2 SINGLE BEGINDASH 5\nIPD C3 C4 SINGLE NONE 6\nIPD C3 O3 SINGLE BEGINDASH 7\nIPD C4 C5 SINGLE NONE 8\nIPD C4 O4 SINGLE BEGINWEDGE 9\nIPD C5 C6 SINGLE NONE 10\nIPD C5 O5 SINGLE BEGINDASH 11\nIPD P1 O1 SINGLE NONE 13\nIPD P1 O7 DOUBLE NONE 14\nIPD P1 O8 SINGLE NONE 15\nIPD P1 O9 SINGLE NONE 16\n#\nloop_\n_pdbe_chem_comp_substructure.comp_id\n_pdbe_chem_comp_substructure.substructure_name\n_pdbe_chem_comp_substructure.id\n_pdbe_chem_comp_substructure.substructure_type\n_pdbe_chem_comp_substructure.substructure_smiles\n_pdbe_chem_comp_substructure.substructure_inchis\n_pdbe_chem_comp_substructure.substructure_inchikeys\nIPD MurckoScaffold S1 scaffold C1CCCCC1 InChI=1S/C6H12/c1-2-4-6-5-3-1/h1-6H2 XDTMQSROBMDMFD-UHFFFAOYSA-N\nIPD cyclohexane F1 fragment C1CCCCC1 InChI=1S/C6H12/c1-2-4-6-5-3-1/h1-6H2 XDTMQSROBMDMFD-UHFFFAOYSA-N\nIPD phosphate F2 fragment O=P(O)(O)O InChI=1S/H3O4P/c1-5(2,3)4/h(H3,1,2,3,4) NBIIXXVUZAFLBC-UHFFFAOYSA-N\n#\nloop_\n_pdbe_chem_comp_substructure_mapping.comp_id\n_pdbe_chem_comp_substructure_mapping.atom_id\n_pdbe_chem_comp_substructure_mapping.substructure_id\n_pdbe_chem_comp_substructure_mapping.substructure_ordinal\nIPD C1 S1 1\nIPD C2 S1 1\nIPD C3 S1 1\nIPD C4 S1 1\nIPD C5 S1 1\nIPD C6 S1 1\nIPD C1 F1 1\nIPD C2 F1 1\nIPD C3 F1 1\nIPD C4 F1 1\nIPD C5 F1 1\nIPD C6 F1 1\nIPD O1 F2 1\nIPD P1 F2 1\nIPD O7 F2 1\nIPD O8 F2 1\nIPD O9 F2 1\n#\n_pdbe_chem_comp_rdkit_properties.comp_id IPD\n_pdbe_chem_comp_rdkit_properties.exactmw 258.015\n_pdbe_chem_comp_rdkit_properties.amw 258.119\n_pdbe_chem_comp_rdkit_properties.lipinskiHBA 9\n_pdbe_chem_comp_rdkit_properties.lipinskiHBD 5\n_pdbe_chem_comp_rdkit_properties.NumRotatableBonds 7\n_pdbe_chem_comp_rdkit_properties.NumHBD 5\n_pdbe_chem_comp_rdkit_properties.NumHBA 9\n_pdbe_chem_comp_rdkit_properties.NumHeavyAtoms 16\n_pdbe_chem_comp_rdkit_properties.NumAtoms 27\n_pdbe_chem_comp_rdkit_properties.NumHeteroatoms 10\n_pdbe_chem_comp_rdkit_properties.NumAmideBonds 0\n_pdbe_chem_comp_rdkit_properties.FractionCSP3 1\n_pdbe_chem_comp_rdkit_properties.NumRings 1\n_pdbe_chem_comp_rdkit_properties.NumAromaticRings 0\n_pdbe_chem_comp_rdkit_properties.NumAliphaticRings 1\n_pdbe_chem_comp_rdkit_properties.NumSaturatedRings 1\n_pdbe_chem_comp_rdkit_properties.NumHeterocycles 0\n_pdbe_chem_comp_rdkit_properties.NumAromaticHeterocycles 0\n_pdbe_chem_comp_rdkit_properties.NumSaturatedHeterocycles 0\n_pdbe_chem_comp_rdkit_properties.NumAliphaticHeterocycles 0\n_pdbe_chem_comp_rdkit_properties.NumSpiroAtoms 0\n_pdbe_chem_comp_rdkit_properties.NumBridgeheadAtoms 0\n_pdbe_chem_comp_rdkit_properties.NumAtomStereoCenters 6\n_pdbe_chem_comp_rdkit_properties.NumUnspecifiedAtomStereoCenters 0\n_pdbe_chem_comp_rdkit_properties.labuteASA 105.669\n_pdbe_chem_comp_rdkit_properties.tpsa

173.570\n_pdbe_chem_comp_rdkit_properties.CrippenClogP -
4.982\n_pdbe_chem_comp_rdkit_properties.CrippenMR
42.514\n_pdbe_chem_comp_rdkit_properties.chi0v
8.016\n_pdbe_chem_comp_rdkit_properties.chi1v
4.916\n_pdbe_chem_comp_rdkit_properties.chi2v
1.847\n_pdbe_chem_comp_rdkit_properties.chi3v
1.847\n_pdbe_chem_comp_rdkit_properties.chi4v
1.203\n_pdbe_chem_comp_rdkit_properties.chi0n
18.121\n_pdbe_chem_comp_rdkit_properties.chi1n
8.496\n_pdbe_chem_comp_rdkit_properties.chi2n
1.440\n_pdbe_chem_comp_rdkit_properties.chi3n
1.440\n_pdbe_chem_comp_rdkit_properties.chi4n
0.813\n_pdbe_chem_comp_rdkit_properties.hallKierAlpha -
0.090\n_pdbe_chem_comp_rdkit_properties.kappa1
4.884\n_pdbe_chem_comp_rdkit_properties.kappa2
4.649\n_pdbe_chem_comp_rdkit_properties.kappa3
2.774\n_pdbe_chem_comp_rdkit_properties.Phi
1.419\n#\nloop_\n_pdbe_chem_comp_external_mappings.comp_id\n_pdbe_chem_comp_external_mappings.source\n_pdbe_chem_comp_external_mappings.resource\n_pdbe_chem_comp_external_mappings.resource_id\nIPD UniChem ChEBI
58433\nIPD UniChem Recon 'mi1p_D'\nIPD UniChem MetaboLights
MTBLC58433\nIPD UniChem Rhea
58433\n#\nloop_\n_pdbe_chem_comp_rdkit_conformer.comp_id\n_pdbe_chem_comp_rdkit_conformer.atom_id\n_pdbe_chem_comp_rdkit_conformer.Cartn_x_rdkit\n_pdbe_chem_comp_rdkit_conformer.Cartn_y_rdkit\n_pdbe_chem_comp_rdkit_conformer.Cartn_z_rdkit\n_pdbe_chem_comp_rdkit_conformer.rdkit_method\n_pdbe_chem_comp_rdkit_conformer.rdkit_ordinal\nIPD C1 -0.935 -
0.183 -0.287 ETKDGv3 1\nIPD C2 -0.321 1.219 -0.072 ETKDGv3 2\nIPD C3
0.902 1.142 0.866 ETKDGv3 3\nIPD C4 1.964 0.162 0.324 ETKDGv3 4\nIPD
C5 1.355 -1.238 0.101 ETKDGv3 5\nIPD C6 0.130 -1.158 -0.835 ETKDGv3
6\nIPD P1 -3.502 -0.155 -0.427 ETKDGv3 7\nIPD O1 -1.993 -0.100 -1.226
ETKDGv3 8\nIPD O2 0.059 1.789 -1.301 ETKDGv3 9\nIPD O3 1.475 2.417
1.006 ETKDGv3 10\nIPD O4 3.022 0.058 1.243 ETKDGv3 11\nIPD O5 2.314 -
2.087 -0.477 ETKDGv3 12\nIPD O7 -3.719 -1.531 0.159 ETKDGv3 14\nIPD O8
-4.728 0.168 -1.546 ETKDGv3 15\nIPD O9 -3.564 1.001 0.807 ETKDGv3
16\nIPD H1 -1.253 -0.577 0.709 ETKDGv3 17\nIPD H2 -1.081 1.869 0.421
ETKDGv3 18\nIPD H3 0.568 0.776 1.866 ETKDGv3 19\nIPD H4 2.350 0.537 -
0.654 ETKDGv3 20\nIPD H5 1.025 -1.652 1.084 ETKDGv3 21\nIPD H6 0.471 -
0.790 -1.829 ETKDGv3 22\nIPD HO2 -0.761 2.171 -1.710 ETKDGv3 23\nIPD
HO3 0.875 2.938 1.602 ETKDGv3 24\nIPD HO4 3.614 0.840 1.093 ETKDGv3
25\nIPD HO5 2.895 -2.411 0.260 ETKDGv3 26\n#\n"
}

Fig. 5a. HsEPO carrying core 1 *O*-GalNAc glycan


```
{
    "name": "epo_core1",
    "modelSeeds": [
      1
    ],
    "sequences": [
      {
        "protein": {
          "sequence":
"APPRLICDSRVLERYLLEAKEAENITTGCAEHCSLNENITVPDTKVNFYAWKRMEVGQQAVEVWQGLAL
LSEAVLRGQALLVNSSQPWEPLQLHVDKAVSGLRSLTTLLRALGAQKEAISPPDAASAAPLRTITADTFR
KLFRVYSNFLRGKLKLYTGEACRTGDR",
          "id": "EPO"
        }
      },
      {
        "ligand": {
          "ccdCodes": [
            "A2G", "GAL"
          ],
          "id": ["OGA"]
        }
      }
    ],
    "dialect": "alphafold3",
    "version": 2,
    "bondedAtomPairs": [
    [["EPO",126,"OG"],["OGA",1,"C1"]],
    [["OGA",1,"O3"],["OGA",2,"C1"]]
    ]
  }
```

Fig. 5b. HsEPO carrying core 2 *O*-GalNAc glycan


```
{
    "name": "epo_core2",
    "modelSeeds": [
      1
    ],
    "sequences": [
      {
        "protein": {
          "sequence":
"APPRLICDSRVLERYLLEAKEAENITTGCAEHCSLNENITVPDTKVNFYAWKRMEVGQQAVEVWQGLAL
LSEAVLRGQALLVNSSQPWEPLQLHVDKAVSGLRSLTTLLRALGAQKEAISPPDAASAAPLRTITADTFR
KLFRVYSNFLRGKLKLYTGEACRTGDR",
          "id": "EPO"
        }
      },
      {
        "ligand": {
          "ccdCodes": [
            "A2G", "GAL", "NAG"
          ],
          "id": ["OGA"]
        }
      }
    ],
    "dialect": "alphafold3",
    "version": 2,
    "bondedAtomPairs": [
    [["EPO",126,"OG"],["OGA",1,"C1"]],
    [["OGA",1,"O3"],["OGA",2,"C1"]],
    [["OGA",1,"O6"],["OGA",3,"C1"]]
    ]
  }
```

Fig. 5c. HsEPO carrying core 3 *O*-GalNAc glycan


```
{
    "name": "epo_core3",
    "modelSeeds": [
      1
    ],
    "sequences": [
      {
        "protein": {
          "sequence":
"APPRLICDSRVLERYLLEAKEAENITTGCAEHCSLNENITVPDTKVNFYAWKRMEVGQQAVEVWQGLAL
LSEAVLRGQALLVNSSQPWEPLQLHVDKAVSGLRSLTTLLRALGAQKEAISPPDAASAAPLRTITADTFR
KLFRVYSNFLRGKLKLYTGEACRTGDR",
          "id": "EPO"
        }
      },
      {
        "ligand": {
          "ccdCodes": [
            "A2G", "NAG"
          ],
          "id": ["OGA"]
        }
      }
    ],
    "dialect": "alphafold3",
    "version": 2,
    "bondedAtomPairs": [
    [["EPO",126,"OG"],["OGA",1,"C1"]],
    [["OGA",1,"O3"],["OGA",2,"C1"]]
    ]
  }
```

Fig. 5d. HsEPO carrying core 4 *O*-GalNAc glycan


```
{
    "name": "epo_core4",
    "modelSeeds": [
      1
    ],
    "sequences": [
      {
        "protein": {
          "sequence":
"APPRLICDSRVLERYLLEAKEAENITTGCAEHCSLNENITVPDTKVNFYAWKRMEVGQQAVEVWQGLAL
LSEAVLRGQALLVNSSQPWEPLQLHVDKAVSGLRSLTTLLRALGAQKEAISPPDAASAAPLRTITADTFR
KLFRVYSNFLRGKLKLYTGEACRTGDR",
          "id": "EPO"
        }
      },
      {
        "ligand": {
          "ccdCodes": [
            "A2G", "NAG", "NAG"
          ],
          "id": ["OGA"]
        }
      }
    ],
    "dialect": "alphafold3",
    "version": 2,
    "bondedAtomPairs": [
    [["EPO",126,"OG"],["OGA",1,"C1"]],
    [["OGA",1,"O3"],["OGA",2,"C1"]],
    [["OGA",1,"O6"],["OGA",3,"C1"]]
    ]
  }
```

Fig. 5e. HsEPO carrying extended disialyl core 1 *O*-GalNAc glycan

{
    "name": "epo_dscore1",
    "modelSeeds": [
      1
    ],
    "sequences": [
      {
        "protein": {
          "sequence":
"APPRLICDSRVLERYLLEAKEAENITTGCAEHCSLNENITVPDTKVNFYAWKRMEVGQQAVEVWQGLAL
LSEAVLRGQALLVNSSQPWEPLQLHVDKAVSGLRSLTTLLRALGAQKEAISPPDAASAAPLRTITADTFR
KLFRVYSNFLRGKLKLYTGEACRTGDR",
          "id": "EPO"
        }
      },
      {
        "ligand": {
          "ccdCodes": [
            "A2G", "GAL","SIA-2", "SIA-2"
          ],
          "id": ["OGA"]
        }
      }
    ],
    "dialect": "alphafold3",
    "version": 2,
    "bondedAtomPairs": [
    [["EPO",126,"OG"],["OGA",1,"C1"]],
    [["OGA",1,"O3"],["OGA",2,"C1"]],
    [["OGA",2,"O4"],["OGA",3,"C2"]],
    [["OGA",1,"O6"],["OGA",4,"C2"]]
    ],
    "userCCD": "data_SIA-2\n#\n_chem_comp.id SIA\n_chem_comp.name 'N-
acetyl-alpha-neuraminic acid'\n_chem_comp.type 'D-saccharide, alpha
linking'\n_chem_comp.pdbx_type ATOMS\n_chem_comp.formula 'C11 H19 N
O9'\n_chem_comp.mon_nstd_parent_comp_id ?\n_chem_comp.pdbx_synonyms
'N-acetylneuraminic acid; sialic acid; alpha-sialic acid; O-SIALIC
ACID'\n_chem_comp.pdbx_formal_charge 0\n_chem_comp.pdbx_initial_date
1999-07-08\n_chem_comp.pdbx_modified_date 2024-09-
27\n_chem_comp.pdbx_ambiguous_flag N\n_chem_comp.pdbx_release_status
REL\n_chem_comp.pdbx_replaced_by ?\n_chem_comp.pdbx_replaces
NAN\n_chem_comp.formula_weight
309.270\n_chem_comp.one_letter_code ?\n_chem_comp.three_letter_code
SIA\n_chem_comp.pdbx_model_coordinates_details ?\n_chem_comp.pdbx_mode
l_coordinates_missing_flag
N\n_chem_comp.pdbx_ideal_coordinates_details ?\n_chem_comp.pdbx_ideal_
coordinates_missing_flag
N\n_chem_comp.pdbx_model_coordinates_db_code ?\n_chem_comp.pdbx_subcom
ponent_list ?\n_chem_comp.pdbx_processing_site
EBI\n_chem_comp.pdbx_pcm
Y\n#\nloop_\n_pdbx_chem_comp_synonyms.ordinal\n_pdbx_chem_comp_synonym

s.comp_id\n_pdbx_chem_comp_synonyms.name\n_pdbx_chem_comp_synonyms.provenance\n_pdbx_chem_comp_synonyms.type\n1 SIA 'N-acetylneuraminic acid' PDB ?\n2 SIA 'sialic acid' PDB ?\n3 SIA 'alpha-sialic acid' PDB ?\n4 SIA 'O-SIALIC ACID' PDB ?\n#\nloop_\n_chem_comp_atom.comp_id\n_chem_comp_atom.atom_id\n_chem_comp_atom.alt_atom_id\n_chem_comp_atom.type_symbol\n_chem_comp_atom.charge\n_chem_comp_atom.pdbx_align\n_chem_comp_atom.pdbx_aromatic_flag\n_chem_comp_atom.pdbx_leaving_atom_flag\n_chem_comp_atom.pdbx_stereo_config\n_chem_comp_atom.pdbx_backbone_atom_flag\n_chem_comp_atom.pdbx_n_terminal_atom_flag\n_chem_comp_atom.pdbx_c_terminal_atom_flag\n_chem_comp_atom.model_Cartn_x\n_chem_comp_atom.model_Cartn_y\n_chem_comp_atom.model_Cartn_z\n_chem_comp_atom.pdbx_model_Cartn_x_ideal\n_chem_comp_atom.pdbx_model_Cartn_y_ideal\n_chem_comp_atom.pdbx_model_Cartn_z_ideal\n_chem_comp_atom.pdbx_component_atom_id\n_chem_comp_atom.pdbx_component_comp_id\n_chem_comp_atom.pdbx_ordinal\nSIA C1 C1 C 0 1 N N N N N N -2.196 58.872 -5.981 -2.502 -0.832 0.174 C1 SIA 1\nSIA C2 C2 C 0 1 N N R N N N -1.870 58.021 -7.211 -2.171 0.628 0.342 C2 SIA 2\nSIA C3 C3 C 0 1 N N N N N N -0.844 56.899 -7.306 -1.789 0.898 1.800 C3 SIA 3\nSIA C4 C4 C 0 1 N N S N N N -1.157 55.904 -8.413 -0.586 0.023 2.171 C4 SIA 4\nSIA C5 C5 C 0 1 N N R N N N -2.015 56.516 -9.517 0.529 0.264 1.148 C5 SIA 5\nSIA C6 C6 C 0 1 N N R N N N -3.352 56.956 -8.912 -0.026 0.043 -0.259 C6 SIA 6\nSIA C7 C7 C 0 1 N N R N N N -4.224 57.698 -9.942 1.088 0.251 -1.286 C7 SIA 7\nSIA C8 C8 C 0 1 N N R N N N -5.571 58.131 -9.360 0.535 0.021 -2.694 C8 SIA 8\nSIA C9 C9 C 0 1 N N N N N N -6.601 58.674 -10.381 1.650 0.229 -3.721 C9 SIA 9\nSIA C10 C10 C 0 1 N N N N N N -1.897 55.374 -11.759 2.632 -0.329 2.226 C10 SIA 10\nSIA C11 C11 C 0 1 N N N N N N -2.200 54.057 -12.454 3.763 -1.292 2.478 C11 SIA 11\nSIA N5 N5 N 0 1 N N N N N N -2.202 55.444 -10.478 1.629 -0.671 1.394 N5 SIA 12\nSIA O1A O1A O 0 1 N N N N N N -1.289 58.815 -5.130 -2.191 -1.408 -0.841 O1A SIA 13\nSIA O1B O1B O 0 1 N N N N N N -3.210 59.504 -5.631 -3.141 -1.493 1.152 O1B SIA 14\nSIA O4 O4 O 0 1 N N N N N N 0.072 55.523 -8.986 -0.123 0.370 3.478 O4 SIA 16\nSIA O6 O6 O 0 1 N N N N N N -3.149 57.908 -7.847 -1.082 0.968 -0.513 O6 SIA 17\nSIA O7 O7 O 0 1 N N N N N N -3.594 58.883 -10.402 1.588 1.586 -1.183 O7 SIA 18\nSIA O8 O8 O 0 1 N N N N N N -6.119 56.946 -8.828 0.035 -1.313 -2.797 O8 SIA 19\nSIA O9 O9 O 0 1 N N N N N N -6.931 57.687 -11.346 1.133 0.014 -5.035 O9 SIA 20\nSIA O10 O10 O 0 1 N N N N N N -1.423 56.357 -12.331 2.624 0.753 2.772 O10 SIA 21\nSIA H32 H31 H 0 1 N N N N N N -0.702 56.484 -6.300 -2.631 0.655 2.448 H32 SIA 22\nSIA H31 H32 H 0 1 N N N N N N 0.120 57.408 -7.182 -1.526 1.949 1.919 H31 SIA 23\nSIA H4 H4 H 0 1 N N N N N N -1.651 55.060 -7.897 -0.878 -1.026 2.153 H4 SIA 24\nSIA H5 H5 H 0 1 N N N N N N -1.506 57.375 -9.979 0.893 1.287 1.240 H5 SIA 25\nSIA H6 H6 H 0 1 N N N N N N -3.850 56.075 -8.492 -0.408 -0.973 -0.341 H6 SIA 26\nSIA H7 H7 H 0 1 N N N N N N -4.339 57.176 -10.907 1.896 -0.454 -1.093 H7 SIA 27\nSIA H8 H8 H 0 1 N N N N N N -5.473 58.871 -8.553 -0.272 0.728 -2.887 H8 SIA 28\nSIA H92 H91 H 0 1 N N N N N N -6.054 59.459 -10.925 2.031 1.247 -3.642 H92 SIA 29\nSIA H91 H92 H 0 1 N N N N N N -7.587 59.029 -10.055 2.457 -0.476 -3.528 H91 SIA 30\nSIA H111 H111 H 0 0 N N N N N N -3.215 53.728 -12.207 4.474 -0.844 3.172 H111 SIA 31\nSIA H113 H112 H 0 0 N N N N N N -1.550 53.279 -12.033 3.368 -2.213 2.907 H113 SIA 32\nSIA H112 H113 H 0 0 N N N N N N -2.005 54.041 -13.531 4.266 -1.516 1.537 H112 SIA

33\nSIA HN5 HN5 H 0 1 N N N N N N -2.566 54.658 -10.003 1.635 -1.538 0.957 HN5 SIA 34\nSIA HO1B HOB1 H 0 0 N N N N N N -3.412 60.032 -4.867 -3.353 -2.430 1.044 HO1B SIA 35\nSIA HO4 HO4 H 0 1 N Y N N N N 0.427 54.801 -8.430 -0.854 0.203 4.087 HO4 SIA 37\nSIA HO7 HO7 H 0 1 N Y N N N N -3.109 58.884 -9.548 0.844 2.177 -1.360 HO7 SIA 38\nSIA HO8 HO8 H 0 1 N Y N N N N -7.071 57.051 -8.962 0.779 -1.904 -2.620 HO8 SIA 39\nSIA HO9 HO9 H 0 1 N Y N N N N -6.783 56.885 -10.808 1.866 0.155 -5.650 HO9 SIA
40\n#\nloop_\n_chem_comp_bond.comp_id\n_chem_comp_bond.atom_id_1\n_chem_comp_bond.atom_id_2\n_chem_comp_bond.value_order\n_chem_comp_bond.pdbx_aromatic_flag\n_chem_comp_bond.pdbx_stereo_config\n_chem_comp_bond.pdbx_ordinal\nSIA C1 C2 SING N N 1\nSIA C1 O1A DOUB N N 2\nSIA C1 O1B SING N N 3\nSIA C2 C3 SING N N 4\nSIA C2 O6 SING N N 6\nSIA C3 C4 SING N N 7\nSIA C3 H32 SING N N 8\nSIA C3 H31 SING N N 9\nSIA C4 C5 SING N N 10\nSIA C4 O4 SING N N 11\nSIA C4 H4 SING N N 12\nSIA C5 C6 SING N N 13\nSIA C5 N5 SING N N 14\nSIA C5 H5 SING N N 15\nSIA C6 C7 SING N N 16\nSIA C6 O6 SING N N 17\nSIA C6 H6 SING N N 18\nSIA C7 C8 SING N N 19\nSIA C7 O7 SING N N 20\nSIA C7 H7 SING N N 21\nSIA C8 C9 SING N N 22\nSIA C8 O8 SING N N 23\nSIA C8 H8 SING N N 24\nSIA C9 O9 SING N N 25\nSIA C9 H92 SING N N 26\nSIA C9 H91 SING N N 27\nSIA C10 C11 SING N N 28\nSIA C10 N5 SING N N 29\nSIA C10 O10 DOUB N N 30\nSIA C11 H111 SING N N 31\nSIA C11 H113 SING N N 32\nSIA C11 H112 SING N N 33\nSIA N5 HN5 SING N N 34\nSIA O1B HO1B SING N N 35\nSIA O4 HO4 SING N N 37\nSIA O7 HO7 SING N N 38\nSIA O8 HO8 SING N N 39\nSIA O9 HO9 SING N N
40\n#\nloop_\n_pdbx_chem_comp_descriptor.comp_id\n_pdbx_chem_comp_descriptor.type\n_pdbx_chem_comp_descriptor.program\n_pdbx_chem_comp_descriptor.program_version\n_pdbx_chem_comp_descriptor.descriptor\nSIA SMILES ACDLabs 10.04 'O=C(O)C1(O)OC(C(O)C(O)CO)C(NC(=O)C)C(O)C1'\nSIA SMILES_CANONICAL CACTVS 3.341
'CC(=O)N[C@@H]1[C@@H](O)C[C@@](O)(O[C@H]1[C@H](O)[C@H](O)CO)C(O)=O'\nSIA SMILES CACTVS 3.341
'CC(=O)N[CH]1[CH](O)C[C](O)(O[CH]1[CH](O)[CH](O)CO)C(O)=O'\nSIA SMILES_CANONICAL 'OpenEye OEToolkits' 1.5.0
'CC(=O)N[C@@H]1[C@H](C[C@@](O[C@H]1[C@@H]([C@@H](CO)O)O)(C(=O)O)O)O'\nSIA SMILES 'OpenEye OEToolkits' 1.5.0
'CC(=O)NC1C(CC(OC1C(C(CO)O)O)(C(=O)O)O)O'\nSIA InChI InChI 1.03
'InChI=1S/C11H19NO9/c1-4(14)12-7-5(15)2-11(20,10(18)19)21-9(7)8(17)6(16)3-13/h5-9,13,15-17,20H,2-3H2,1H3,(H,12,14)(H,18,19)/t5-,6+,7+,8+,9+,11+/m0/s1'\nSIA InChIKey InChI 1.03 SQVRNKJHWKZAKO-YRMXFSIDSA-N\n#\nloop_\n_pdbx_chem_comp_identifier.comp_id\n_pdbx_chem_comp_identifier.type\n_pdbx_chem_comp_identifier.program\n_pdbx_chem_comp_identifier.program_version\n_pdbx_chem_comp_identifier.identifier\nSIA 'SYSTEMATIC NAME' ACDLabs 10.04 '5-(acetylamino)-3,5-dideoxy-D-glycero-alpha-D-galacto-non-2-ulopyranosonic acid'\nSIA 'SYSTEMATIC NAME' 'OpenEye OEToolkits' 1.5.0 '(2R,4S,5R,6R)-5-acetamido-2,4-dihydroxy-6-[(1R,2R)-1,2,3-trihydroxypropyl]oxane-2-carboxylic acid'\nSIA 'CONDENSED IUPAC CARBOHYDRATE SYMBOL' GMML 1.0 DNeup5Aca\nSIA 'COMMON NAME' GMML 1.0 'N-acetyl-a-D-neuraminic acid'\nSIA 'IUPAC CARBOHYDRATE SYMBOL' PDB-CARE 1.0 a-D-Neup5Ac\nSIA 'SNFG CARBOHYDRATE SYMBOL' GMML 1.0

Neu5Ac\n#\nloop_\n_pdbx_chem_comp_feature.comp_id\n_pdbx_chem_comp_feature.type\n_pdbx_chem_comp_feature.value\n_pdbx_chem_comp_feature.source\n_pdbx_chem_comp_feature.support\nSIA 'CARBOHYDRATE ISOMER' D
PDB ?\nSIA 'CARBOHYDRATE RING' pyranose PDB ?\nSIA 'CARBOHYDRATE
ANOMER' alpha PDB ?\nSIA 'CARBOHYDRATE PRIMARY CARBONYL GROUP' ketose
PDB ?\n#\nloop_\n_pdbx_chem_comp_audit.comp_id\n_pdbx_chem_comp_audit.action_type\n_pdbx_chem_comp_audit.date\n_pdbx_chem_comp_audit.processing_site\nSIA 'Create component' 1999-07-08 EBI\nSIA 'Modify
descriptor' 2011-06-04 RCSB\nSIA 'Other modification' 2019-08-12
RCSB\nSIA 'Other modification' 2019-12-19 RCSB\nSIA 'Other
modification' 2020-07-03 RCSB\nSIA 'Modify name' 2020-07-17 RCSB\nSIA
'Modify synonyms' 2020-07-17 RCSB\nSIA 'Modify atom id' 2020-07-17
RCSB\nSIA 'Modify component atom id' 2020-07-17 RCSB\nSIA 'Modify PCM'
2024-09-27 PDBE\n#\n_pdbx_chem_comp_pcm.pcm_id
1\n_pdbx_chem_comp_pcm.comp_id
SIA\n_pdbx_chem_comp_pcm.modified_residue_id
THR\n_pdbx_chem_comp_pcm.type None\n_pdbx_chem_comp_pcm.category
Carbohydrate\n_pdbx_chem_comp_pcm.position 'Amino-acid side
chain'\n_pdbx_chem_comp_pcm.polypeptide_position 'Any
position'\n_pdbx_chem_comp_pcm.comp_id_linking_atom
C2\n_pdbx_chem_comp_pcm.modified_residue_id_linking_atom
OG1\n_pdbx_chem_comp_pcm.uniprot_specific_ptm_accession ?\n_pdbx_chem_comp_pcm.uniprot_generic_ptm_accession ?\n#\n_pdbe_chem_comp_drugbank_details.comp_id SIA\n_pdbe_chem_comp_drugbank_details.drugbank_id
DB03721\n_pdbe_chem_comp_drugbank_details.type 'small
molecule'\n_pdbe_chem_comp_drugbank_details.name 'N-acetyl-alpha-
neuraminic acid'\n_pdbe_chem_comp_drugbank_details.description\n'An N-
acyl derivative of neuraminic acid. N-acetylneuraminic acid occurs in
many polysaccharides, glycoproteins, and glycolipids in animals and
bacteria. (From Dorland, 28th ed,
p1518)'\n_pdbe_chem_comp_drugbank_details.cas_number 21646-00-
4\n_pdbe_chem_comp_drugbank_details.mechanism_of_action ?\n#\nloop_\n_pdbe_chem_comp_synonyms.comp_id\n_pdbe_chem_comp_synonyms.name\n_pdbe_chem_comp_synonyms.provenance\n_pdbe_chem_comp_synonyms.type\nSIA 'N-
acetylneuraminic acid' wwPDB ?\nSIA 'sialic acid' wwPDB ?\nSIA 'alpha-
sialic acid' wwPDB ?\nSIA 'O-SIALIC ACID' wwPDB ?\nSIA 'N-Acetyl-
alpha-D-neuraminic acid' DrugBank ?\nSIA 'O-sialic acid'
DrugBank ?\nSIA 'α-Neu5Ac'
DrugBank ?\n#\n_pdbe_chem_comp_drugbank_classification.comp_id
SIA\n_pdbe_chem_comp_drugbank_classification.drugbank_id
DB03721\n_pdbe_chem_comp_drugbank_classification.parent 'N-
acylneuraminic acids'\n_pdbe_chem_comp_drugbank_classification.kingdom
'Organic compounds'\n_pdbe_chem_comp_drugbank_classification.class
'Organooxygen
compounds'\n_pdbe_chem_comp_drugbank_classification.superclass
'Organic oxygen
compounds'\n_pdbe_chem_comp_drugbank_classification.description\n'This
compound belongs to the class of organic compounds known as n-
acylneuraminic acids. These are neuraminic acids carrying an N-acyl
substituent.'\n#\nloop_\n_pdbe_chem_comp_drugbank_targets.comp_id\n_pdbe_chem_comp_drugbank_targets.drugbank_id\n_pdbe_chem_comp_drugbank_targets.name\n_pdbe_chem_comp_drugbank_targets.organism\n_pdbe_chem_comp

_drugbank_targets.uniprot_id\n_pdbe_chem_comp_drugbank_targets.pharmacologically_active\n_pdbe_chem_comp_drugbank_targets.ordinal\nSIA DB03721 P-selectin Humans P16109 yes 1\nSIA DB03721 E-selectin Humans P16581 yes 2\nSIA DB03721 'Liver carboxylesterase 1' Humans P23141 yes 3\nSIA DB03721 '3-deoxy-manno-octulosonate cytidylyltransferase' 'Escherichia coli' P42216 unknown 4\nSIA DB03721 'Tetanus toxin' 'Clostridium tetani (strain Massachusetts / E88)' P04958 unknown 5\nSIA DB03721 'Cholera enterotoxin subunit B' 'Vibrio cholerae serotype O1 (strain ATCC 39315 / El Tor Inaba N16961)' P01556 unknown 6\nSIA DB03721 'Botulinum neurotoxin type B' 'Clostridium botulinum' P10844 unknown 7\nSIA DB03721 'Mannose-binding protein C' Humans P11226 unknown 8\nSIA DB03721 Lithostathine-1-alpha Humans P05451 unknown 9\nSIA DB03721 Endo-N-acetylneuraminidase 'Enterobacteria phage K1F' Q04830 unknown 10\nSIA DB03721 'Enterotoxin type B' 'Staphylococcus aureus' P01552 unknown 11\nSIA DB03721 Neuraminidase 'Influenza A virus (strain A/Tern/Australia/G70C/1975 H11N9)' P03472 unknown 12\nSIA DB03721 Hemagglutinin-neuraminidase NDV P32884 unknown 13\nSIA DB03721 Fiber 'Human adenovirus 19' Q64822 unknown 14\nSIA DB03721 Sialoadhesin Humans Q9BZZ2 unknown 15\nSIA DB03721 Zinc-alpha-2-glycoprotein Humans P25311 unknown 16\nSIA DB03721 'Capsid protein VP1' MPyV P49302 unknown 17\nSIA DB03721 Fiber 'Human adenovirus D37' Q64823 unknown
18\n#\nloop_\n_software.name\n_software.version\n_software.description\nrdkit 2023.09.6 'Core functionality.'\npdbeccdutils 0.8.6 'Wrapper to provide 2D templates and molecular
fragments.'\n#\nloop_\n_pdbe_chem_comp_atom_depiction.comp_id\n_pdbe_chem_comp_atom_depiction.atom_id\n_pdbe_chem_comp_atom_depiction.element\n_pdbe_chem_comp_atom_depiction.model_Cartn_x\n_pdbe_chem_comp_atom_depiction.model_Cartn_y\n_pdbe_chem_comp_atom_depiction.pdbx_ordinal\nSIA C1 C 5.654 -3.375 1\nSIA C2 C 6.404 -2.076 2\nSIA C3 C 5.104 -1.326 3\nSIA C4 C 5.104 0.174 4\nSIA C5 C 6.404 0.924 5\nSIA C6 C 7.702 0.174 6\nSIA C7 C 9.002 0.924 7\nSIA C8 C 10.301 0.174 8\nSIA C9 C 11.600 0.924 9\nSIA C10 C 5.104 3.174 10\nSIA C11 C 3.805 2.424 11\nSIA N5 N 6.404 2.424 12\nSIA O1A O 4.154 -3.375 13\nSIA O1B O 6.404 -4.674 14\nSIA O4 O 3.805 0.924 16\nSIA O6 O 7.702 -1.326 17\nSIA O7 O 9.002 2.424 18\nSIA O8 O 10.301 -1.326 19\nSIA O9 O 12.899 0.174 20\nSIA O10 O 5.104 4.674
21\n#\nloop_\n_pdbe_chem_comp_bond_depiction.comp_id\n_pdbe_chem_comp_bond_depiction.atom_id_1\n_pdbe_chem_comp_bond_depiction.atom_id_2\n_pdbe_chem_comp_bond_depiction.value_order\n_pdbe_chem_comp_bond_depiction.bond_dir\n_pdbe_chem_comp_bond_depiction.pdbx_ordinal\nSIA C1 C2 SINGLE NONE 1\nSIA C1 O1A DOUBLE NONE 2\nSIA C1 O1B SINGLE NONE 3\nSIA C2 C3 SINGLE NONE 4\nSIA C2 O6 SINGLE NONE 6\nSIA C3 C4 SINGLE NONE 7\nSIA C4 C5 SINGLE NONE 8\nSIA C4 O4 SINGLE BEGINDASH 9\nSIA C5 C6 SINGLE NONE 10\nSIA C5 N5 SINGLE BEGINWEDGE 11\nSIA C6 C7 SINGLE NONE 12\nSIA C6 O6 SINGLE BEGINDASH 13\nSIA C7 C8 SINGLE NONE 14\nSIA C7 O7 SINGLE BEGINDASH 15\nSIA C8 C9 SINGLE NONE 16\nSIA C8 O8 SINGLE BEGINWEDGE 17\nSIA C9 O9 SINGLE NONE 18\nSIA C10 C11 SINGLE NONE 19\nSIA C10 N5 SINGLE NONE 20\nSIA C10 O10 DOUBLE NONE
21\n#\nloop_\n_pdbe_chem_comp_substructure.comp_id\n_pdbe_chem_comp_substructure.substructure_name\n_pdbe_chem_comp_substructure.id\n_pdbe_chem_comp_substructure.substructure_type\n_pdbe_chem_comp_substructure.

substructure_smiles\n_pdbe_chem_comp_substructure.substructure_inchis\
n_pdbe_chem_comp_substructure.substructure_inchikeys\nSIA
MurckoScaffold S1 scaffold C1CCOCC1 InChI=1S/C5H10O/c1-2-4-6-5-3-1/h1-
5H2 DHXVGJBLRPWPCS-UHFFFAOYSA-N\nSIA amide F1 fragment CC(N)=O
InChI=1S/C2H5NO/c1-2(3)4/h1H3,(H2,3,4) DLFVBJFMPXGRIB-UHFFFAOYSA-
N\nSIA pyranose F2 fragment OC1CCCCO1 InChI=1S/C5H10O2/c6-5-3-1-2-4-7-
5/h5-6H,1-4H2 CELWCAITJAEQNL-UHFFFAOYSA-
N\n#\nloop_\n_pdbe_chem_comp_substructure_mapping.comp_id\n_pdbe_chem_
comp_substructure_mapping.atom_id\n_pdbe_chem_comp_substructure_mappin
g.substructure_id\n_pdbe_chem_comp_substructure_mapping.substructure_o
rdinal\nSIA C2 S1 1\nSIA C3 S1 1\nSIA C4 S1 1\nSIA C5 S1 1\nSIA C6 S1
1\nSIA O6 S1 1\nSIA N5 F1 1\nSIA C10 F1 1\nSIA O10 F1 1\nSIA C11 F1
1\nSIA C5 F2 1\nSIA C6 F2 1\nSIA O6 F2 1\nSIA C2 F2 1\nSIA C3 F2
1\nSIA C4 F2 1\n#\n_pdbe_chem_comp_rdkit_properties.comp_id
SIA\n_pdbe_chem_comp_rdkit_properties.exactmw
309.106\n_pdbe_chem_comp_rdkit_properties.amw
309.271\n_pdbe_chem_comp_rdkit_properties.lipinskiHBA
10\n_pdbe_chem_comp_rdkit_properties.lipinskiHBD
7\n_pdbe_chem_comp_rdkit_properties.NumRotatableBonds
11\n_pdbe_chem_comp_rdkit_properties.NumHBD
7\n_pdbe_chem_comp_rdkit_properties.NumHBA
9\n_pdbe_chem_comp_rdkit_properties.NumHeavyAtoms
21\n_pdbe_chem_comp_rdkit_properties.NumAtoms
40\n_pdbe_chem_comp_rdkit_properties.NumHeteroatoms
10\n_pdbe_chem_comp_rdkit_properties.NumAmideBonds
1\n_pdbe_chem_comp_rdkit_properties.FractionCSP3
0.818\n_pdbe_chem_comp_rdkit_properties.NumRings
1\n_pdbe_chem_comp_rdkit_properties.NumAromaticRings
0\n_pdbe_chem_comp_rdkit_properties.NumAliphaticRings
1\n_pdbe_chem_comp_rdkit_properties.NumSaturatedRings
1\n_pdbe_chem_comp_rdkit_properties.NumHeterocycles
1\n_pdbe_chem_comp_rdkit_properties.NumAromaticHeterocycles
0\n_pdbe_chem_comp_rdkit_properties.NumSaturatedHeterocycles
1\n_pdbe_chem_comp_rdkit_properties.NumAliphaticHeterocycles
1\n_pdbe_chem_comp_rdkit_properties.NumSpiroAtoms
0\n_pdbe_chem_comp_rdkit_properties.NumBridgeheadAtoms
0\n_pdbe_chem_comp_rdkit_properties.NumAtomStereoCenters
6\n_pdbe_chem_comp_rdkit_properties.NumUnspecifiedAtomStereoCenters
0\n_pdbe_chem_comp_rdkit_properties.labuteASA
146.407\n_pdbe_chem_comp_rdkit_properties.tpsa
176.780\n_pdbe_chem_comp_rdkit_properties.CrippenClogP -
3.872\n_pdbe_chem_comp_rdkit_properties.CrippenMR
64.787\n_pdbe_chem_comp_rdkit_properties.chi0v
9.621\n_pdbe_chem_comp_rdkit_properties.chi1v
4.738\n_pdbe_chem_comp_rdkit_properties.chi2v
1.941\n_pdbe_chem_comp_rdkit_properties.chi3v
1.941\n_pdbe_chem_comp_rdkit_properties.chi4v
1.128\n_pdbe_chem_comp_rdkit_properties.chi0n
28.621\n_pdbe_chem_comp_rdkit_properties.chi1n
13.635\n_pdbe_chem_comp_rdkit_properties.chi2n
1.941\n_pdbe_chem_comp_rdkit_properties.chi3n
1.941\n_pdbe_chem_comp_rdkit_properties.chi4n

1.128\n_pdbe_chem_comp_rdkit_properties.hallKierAlpha -
1.300\n_pdbe_chem_comp_rdkit_properties.kappa1
4.600\n_pdbe_chem_comp_rdkit_properties.kappa2
6.642\n_pdbe_chem_comp_rdkit_properties.kappa3
3.872\n_pdbe_chem_comp_rdkit_properties.Phi
1.455\n#\nloop_\n_pdbe_chem_comp_external_mappings.comp_id\n_pdbe_chem
_comp_external_mappings.source\n_pdbe_chem_comp_external_mappings.reso
urce\n_pdbe_chem_comp_external_mappings.resource_id\nSIA UniChem
ChEMBL CHEMBL1234621\nSIA UniChem DrugBank DB03721\nSIA UniChem ChEBI
49026\nSIA UniChem ZINC ZINC000004081651\nSIA UniChem fdasrs
04A90EXP8V\nSIA UniChem HMDB HMDB0000773\nSIA UniChem Nikkaji
J614.853K\nSIA UniChem MetaboLights MTBLC49026\nSIA UniChem BRENDA
141715\nSIA UniChem BRENDA 233672\nSIA UniChem BRENDA 6105\nSIA
UniChem BRENDA 84245\nSIA UniChem BRENDA 85625\nSIA UniChem 'Probes
And Drugs' PD041137\nSIA UniChem PubChem 444885\nSIA UniChem
eMolecules 474793\nSIA UniChem SureChEMBL SCHEMBL79085\nSIA UniChem
'PubChem TPHARMA' 14776495\nSIA UniChem 'PubChem TPHARMA'
15395566\n#\nloop_\n_pdbe_chem_comp_rdkit_conformer.comp_id\n_pdbe_che
m_comp_rdkit_conformer.atom_id\n_pdbe_chem_comp_rdkit_conformer.Cartn_
x_rdkit\n_pdbe_chem_comp_rdkit_conformer.Cartn_y_rdkit\n_pdbe_chem_com
p_rdkit_conformer.Cartn_z_rdkit\n_pdbe_chem_comp_rdkit_conformer.rdkit
_method\n_pdbe_chem_comp_rdkit_conformer.rdkit_ordinal\nSIA C1 -1.164
-2.811 1.058 ETKDGv3 1\nSIA C2 -1.666 -1.928 -0.070 ETKDGv3 2\nSIA C3
-2.639 -0.809 0.442 ETKDGv3 3\nSIA C4 -2.546 0.454 -0.428 ETKDGv3
4\nSIA C5 -1.079 0.960 -0.528 ETKDGv3 5\nSIA C6 -0.071 -0.181 -0.219
ETKDGv3 6\nSIA C7 1.341 0.133 -0.777 ETKDGv3 7\nSIA C8 2.378 -0.960 -
0.400 ETKDGv3 8\nSIA C9 3.769 -0.651 -0.979 ETKDGv3 9\nSIA C10 -0.223
3.295 0.052 ETKDGv3 10\nSIA C11 0.035 4.333 1.094 ETKDGv3 11\nSIA N5 -
0.868 2.066 0.414 ETKDGv3 12\nSIA O1A -0.936 -2.335 2.203 ETKDGv3
13\nSIA O1B -0.875 -4.149 0.806 ETKDGv3 14\nSIA O4 -3.104 0.224 -1.700
ETKDGv3 16\nSIA O6 -0.537 -1.404 -0.765 ETKDGv3 17\nSIA O7 1.298 0.320
-2.172 ETKDGv3 18\nSIA O8 2.484 -1.066 0.998 ETKDGv3 19\nSIA O9 4.246
0.587 -0.524 ETKDGv3 20\nSIA O10 0.157 3.497 -1.133 ETKDGv3 21\nSIA
H32 -2.392 -0.510 1.484 ETKDGv3 22\nSIA H31 -3.691 -1.171 0.465
ETKDGv3 23\nSIA H4 -3.171 1.239 0.053 ETKDGv3 24\nSIA H5 -0.925 1.324
-1.568 ETKDGv3 25\nSIA H6 0.030 -0.270 0.887 ETKDGv3 26\nSIA H7 1.678
1.089 -0.320 ETKDGv3 27\nSIA H8 2.053 -1.935 -0.838 ETKDGv3 28\nSIA
H92 3.719 -0.629 -2.088 ETKDGv3 29\nSIA H91 4.478 -1.464 -0.703
ETKDGv3 30\nSIA H111 0.843 3.984 1.770 ETKDGv3 31\nSIA H113 -0.888
4.508 1.686 ETKDGv3 32\nSIA H112 0.344 5.291 0.624 ETKDGv3 33\nSIA HN5
-1.113 1.913 1.419 ETKDGv3 34\nSIA HO1B -0.511 -4.749 1.536 ETKDGv3
35\nSIA HO4 -2.492 -0.372 -2.205 ETKDGv3 37\nSIA HO7 1.040 -0.545 -
2.585 ETKDGv3 38\nSIA HO8 1.886 -1.804 1.285 ETKDGv3 39\nSIA HO9 4.592
0.441 0.395 ETKDGv3 40\n#\n"
}

Fig. 5f. HsEPO carrying extended core 2 O-GalNAc glycan & KS


```
{
    "name": "epo_og_ks",
    "modelSeeds": [
      1
    ],
    "sequences": [
      {
        "protein": {
          "sequence":
"MGVHECPAWLWLLLSLLSLPLGLPVLGAPPRLICDSRVLERYLLEAKEAENITTGCAEHCSLNENITVP
DTKVNFYAWKRMEVGQQAVEVWQGLALLSEAVLRGQALLVNSSQPWEPLQLHVDKAVSGLRSLTTLLRAL
GAQKEAISPPDAASAAPLRTITADTFRKLFRVYSNFLRGKLKLYTGEACRTGDR",
          "id": "EPO"
        }
      },
      {
        "ligand": {
          "ccdCodes": [
            "A2G", "GAL", "SIA-2", "NAG", "GAL", "NGS", "GAL", "NGS",
"G6S", "SIA-2", "FUC"
          ],
          "id": ["OGA"]
        }
      }
    ],
    "dialect": "alphafold3",
    "version": 2,
    "bondedAtomPairs": [
    [["EPO",153,"OG"],["OGA",1,"C1"]],
    [["OGA",1,"O3"],["OGA",2,"C1"]],
    [["OGA",2,"O3"],["OGA",3,"C2"]],

    [["OGA",1,"O6"],["OGA",4,"C1"]],
    [["OGA",4,"O4"],["OGA",5,"C1"]],
    [["OGA",5,"O3"],["OGA",6,"C1"]],
    [["OGA",6,"O4"],["OGA",7,"C1"]],
    [["OGA",7,"O3"],["OGA",8,"C1"]],
    [["OGA",8,"O4"],["OGA",9,"C1"]],
    [["OGA",9,"O3"],["OGA",10,"C2"]],
    [["OGA",8,"O3"],["OGA",11,"C1"]]
    ],
    "userCCD": "data_SIA-2\n#\n_chem_comp.id SIA\n_chem_comp.name 'N-
acetyl-alpha-neuraminic acid'\n_chem_comp.type 'D-saccharide, alpha
linking'\n_chem_comp.pdbx_type ATOMS\n_chem_comp.formula 'C11 H19 N
O9'\n_chem_comp.mon_nstd_parent_comp_id ?\n_chem_comp.pdbx_synonyms
'N-acetylneuraminic acid; sialic acid; alpha-sialic acid; O-SIALIC
ACID'\n_chem_comp.pdbx_formal_charge 0\n_chem_comp.pdbx_initial_date
1999-07-08\n_chem_comp.pdbx_modified_date 2024-09-
27\n_chem_comp.pdbx_ambiguous_flag N\n_chem_comp.pdbx_release_status
REL\n_chem_comp.pdbx_replaced_by ?\n_chem_comp.pdbx_replaces
NAN\n_chem_comp.formula_weight
```

309.270\n_chem_comp.one_letter_code ?\n_chem_comp.three_letter_code SIA\n_chem_comp.pdbx_model_coordinates_details ?\n_chem_comp.pdbx_model_coordinates_missing_flag N\n_chem_comp.pdbx_ideal_coordinates_details ?\n_chem_comp.pdbx_ideal_coordinates_missing_flag N\n_chem_comp.pdbx_model_coordinates_db_code ?\n_chem_comp.pdbx_subcomponent_list ?\n_chem_comp.pdbx_processing_site EBI\n_chem_comp.pdbx_pcm Y\n#\nloop_\n_pdbx_chem_comp_synonyms.ordinal\n_pdbx_chem_comp_synonyms.comp_id\n_pdbx_chem_comp_synonyms.name\n_pdbx_chem_comp_synonyms.provenance\n_pdbx_chem_comp_synonyms.type\n1 SIA 'N-acetylneuraminic acid' PDB ?\n2 SIA 'sialic acid' PDB ?\n3 SIA 'alpha-sialic acid' PDB ?\n4 SIA 'O-SIALIC ACID' PDB ?\n#\nloop_\n_chem_comp_atom.comp_id\n_chem_comp_atom.atom_id\n_chem_comp_atom.alt_atom_id\n_chem_comp_atom.type_symbol\n_chem_comp_atom.charge\n_chem_comp_atom.pdbx_align\n_chem_comp_atom.pdbx_aromatic_flag\n_chem_comp_atom.pdbx_leaving_atom_flag\n_chem_comp_atom.pdbx_stereo_config\n_chem_comp_atom.pdbx_backbone_atom_flag\n_chem_comp_atom.pdbx_n_terminal_atom_flag\n_chem_comp_atom.pdbx_c_terminal_atom_flag\n_chem_comp_atom.model_Cartn_x\n_chem_comp_atom.model_Cartn_y\n_chem_comp_atom.model_Cartn_z\n_chem_comp_atom.pdbx_model_Cartn_x_ideal\n_chem_comp_atom.pdbx_model_Cartn_y_ideal\n_chem_comp_atom.pdbx_model_Cartn_z_ideal\n_chem_comp_atom.pdbx_component_atom_id\n_chem_comp_atom.pdbx_component_comp_id\n_chem_comp_atom.pdbx_ordinal\nSIA C1 C1 C 0 1 N N N N N N -2.196 58.872 -5.981 -2.502 -0.832 0.174 C1 SIA 1\nSIA C2 C2 C 0 1 N N R N N N -1.870 58.021 -7.211 -2.171 0.628 0.342 C2 SIA 2\nSIA C3 C3 C 0 1 N N N N N N -0.844 56.899 -7.306 -1.789 0.898 1.800 C3 SIA 3\nSIA C4 C4 C 0 1 N N S N N N -1.157 55.904 -8.413 -0.586 0.023 2.171 C4 SIA 4\nSIA C5 C5 C 0 1 N N R N N N -2.015 56.516 -9.517 0.529 0.264 1.148 C5 SIA 5\nSIA C6 C6 C 0 1 N N R N N N -3.352 56.956 -8.912 -0.026 0.043 -0.259 C6 SIA 6\nSIA C7 C7 C 0 1 N N R N N N -4.224 57.698 -9.942 1.088 0.251 -1.286 C7 SIA 7\nSIA C8 C8 C 0 1 N N R N N N -5.571 58.131 -9.360 0.535 0.021 -2.694 C8 SIA 8\nSIA C9 C9 C 0 1 N N N N N N -6.601 58.674 -10.381 1.650 0.229 -3.721 C9 SIA 9\nSIA C10 C10 C 0 1 N N N N N N -1.897 55.374 -11.759 2.632 -0.329 2.226 C10 SIA 10\nSIA C11 C11 C 0 1 N N N N N N -2.200 54.057 -12.454 3.763 -1.292 2.478 C11 SIA 11\nSIA N5 N5 N 0 1 N N N N N N -2.202 55.444 -10.478 1.629 -0.671 1.394 N5 SIA 12\nSIA O1A O1A O 0 1 N N N N N N -1.289 58.815 -5.130 -2.191 -1.408 -0.841 O1A SIA 13\nSIA O1B O1B O 0 1 N N N N N N -3.210 59.504 -5.631 -3.141 -1.493 1.152 O1B SIA 14\nSIA O4 O4 O 0 1 N N N N N N 0.072 55.523 -8.986 -0.123 0.370 3.478 O4 SIA 16\nSIA O6 O6 O 0 1 N N N N N N -3.149 57.908 -7.847 -1.082 0.968 -0.513 O6 SIA 17\nSIA O7 O7 O 0 1 N N N N N N -3.594 58.883 -10.402 1.588 1.586 -1.183 O7 SIA 18\nSIA O8 O8 O 0 1 N N N N N N -6.119 56.946 -8.828 0.035 -1.313 -2.797 O8 SIA 19\nSIA O9 O9 O 0 1 N N N N N N -6.931 57.687 -11.346 1.133 0.014 -5.035 O9 SIA 20\nSIA O10 O10 O 0 1 N N N N N N -1.423 56.357 -12.331 2.624 0.753 2.772 O10 SIA 21\nSIA H32 H31 H 0 1 N N N N N N -0.702 56.484 -6.300 -2.631 0.655 2.448 H32 SIA 22\nSIA H31 H32 H 0 1 N N N N N N 0.120 57.408 -7.182 -1.526 1.949 1.919 H31 SIA 23\nSIA H4 H4 H 0 1 N N N N N N -1.651 55.060 -7.897 -0.878 -1.026 2.153 H4 SIA 24\nSIA H5 H5 H 0 1 N N N N N N -1.506 57.375 -9.979 0.893 1.287 1.240 H5 SIA 25\nSIA H6 H6 H 0 1 N N N N N N -3.850 56.075 -8.492 -

0.408 -0.973 -0.341 H6 SIA 26\nSIA H7 H7 H 0 1 N N N N N N -4.339 57.176 -10.907 1.896 -0.454 -1.093 H7 SIA 27\nSIA H8 H8 H 0 1 N N N N N N -5.473 58.871 -8.553 -0.272 0.728 -2.887 H8 SIA 28\nSIA H92 H91 H 0 1 N N N N N N -6.054 59.459 -10.925 2.031 1.247 -3.642 H92 SIA 29\nSIA H91 H92 H 0 1 N N N N N N -7.587 59.029 -10.055 2.457 -0.476 -3.528 H91 SIA 30\nSIA H111 H111 H 0 0 N N N N N N -3.215 53.728 -12.207 4.474 -0.844 3.172 H111 SIA 31\nSIA H113 H112 H 0 0 N N N N N N -1.550 53.279 -12.033 3.368 -2.213 2.907 H113 SIA 32\nSIA H112 H113 H 0 0 N N N N N N -2.005 54.041 -13.531 4.266 -1.516 1.537 H112 SIA 33\nSIA HN5 HN5 H 0 1 N N N N N N -2.566 54.658 -10.003 1.635 -1.538 0.957 HN5 SIA 34\nSIA HO1B HOB1 H 0 0 N N N N N N -3.412 60.032 -4.867 -3.353 -2.430 1.044 HO1B SIA 35\nSIA HO4 HO4 H 0 1 N Y N N N N 0.427 54.801 -8.430 -0.854 0.203 4.087 HO4 SIA 37\nSIA HO7 HO7 H 0 1 N Y N N N N -3.109 58.884 -9.548 0.844 2.177 -1.360 HO7 SIA 38\nSIA HO8 HO8 H 0 1 N Y N N N N -7.071 57.051 -8.962 0.779 -1.904 -2.620 HO8 SIA 39\nSIA HO9 HO9 H 0 1 N Y N N N N -6.783 56.885 -10.808 1.866 0.155 -5.650 HO9 SIA
40\n#\nloop_\n_chem_comp_bond.comp_id\n_chem_comp_bond.atom_id_1\n_chem_comp_bond.atom_id_2\n_chem_comp_bond.value_order\n_chem_comp_bond.pdbx_aromatic_flag\n_chem_comp_bond.pdbx_stereo_config\n_chem_comp_bond.pdbx_ordinal\nSIA C1 C2 SING N N 1\nSIA C1 O1A DOUB N N 2\nSIA C1 O1B SING N N 3\nSIA C2 C3 SING N N 4\nSIA C2 O6 SING N N 6\nSIA C3 C4 SING N N 7\nSIA C3 H32 SING N N 8\nSIA C3 H31 SING N N 9\nSIA C4 C5 SING N N 10\nSIA C4 O4 SING N N 11\nSIA C4 H4 SING N N 12\nSIA C5 C6 SING N N 13\nSIA C5 N5 SING N N 14\nSIA C5 H5 SING N N 15\nSIA C6 C7 SING N N 16\nSIA C6 O6 SING N N 17\nSIA C6 H6 SING N N 18\nSIA C7 C8 SING N N 19\nSIA C7 O7 SING N N 20\nSIA C7 H7 SING N N 21\nSIA C8 C9 SING N N 22\nSIA C8 O8 SING N N 23\nSIA C8 H8 SING N N 24\nSIA C9 O9 SING N N 25\nSIA C9 H92 SING N N 26\nSIA C9 H91 SING N N 27\nSIA C10 C11 SING N N 28\nSIA C10 N5 SING N N 29\nSIA C10 O10 DOUB N N 30\nSIA C11 H111 SING N N 31\nSIA C11 H113 SING N N 32\nSIA C11 H112 SING N N 33\nSIA N5 HN5 SING N N 34\nSIA O1B HO1B SING N N 35\nSIA O4 HO4 SING N N 37\nSIA O7 HO7 SING N N 38\nSIA O8 HO8 SING N N 39\nSIA O9 HO9 SING N N
40\n#\nloop_\n_pdbx_chem_comp_descriptor.comp_id\n_pdbx_chem_comp_descriptor.type\n_pdbx_chem_comp_descriptor.program\n_pdbx_chem_comp_descriptor.program_version\n_pdbx_chem_comp_descriptor.descriptor\nSIA SMILES ACDLabs 10.04 'O=C(O)C1(O)OC(C(O)C(O)CO)C(NC(=O)C)C(O)C1'\nSIA SMILES_CANONICAL CACTVS 3.341
'CC(=O)N[C@@H]1[C@@H](O)C[C@@](O)(O[C@H]1[C@H](O)[C@H](O)CO)C(O)=O'\nSIA SMILES CACTVS 3.341
'CC(=O)N[CH]1[CH](O)C[C](O)(O[CH]1[CH](O)[CH](O)CO)C(O)=O'\nSIA SMILES_CANONICAL 'OpenEye OEToolkits' 1.5.0
'CC(=O)N[C@@H]1[C@H](C[C@@](O[C@H]1[C@@H]([C@@H](CO)O)O)(C(=O)O)O)O'\nSIA SMILES 'OpenEye OEToolkits' 1.5.0
'CC(=O)NC1C(CC(OC1C(C(CO)O)O)(C(=O)O)O)O'\nSIA InChI InChI 1.03
'InChI=1S/C11H19NO9/c1-4(14)12-7-5(15)2-11(20,10(18)19)21-9(7)8(17)6(16)3-13/h5-9,13,15-17,20H,2-3H2,1H3,(H,12,14)(H,18,19)/t5-,6+,7+,8+,9+,11+/m0/s1'\nSIA InChIKey InChI 1.03 SQVRNKJHWKZAKO-YRMXFSIDSA-N\n#\nloop_\n_pdbx_chem_comp_identifier.comp_id\n_pdbx_chem_comp_identifier.type\n_pdbx_chem_comp_identifier.program\n_pdbx_chem_comp_identi

fier.program_version\n_pdbx_chem_comp_identifier.identifier\nSIA 'SYSTEMATIC NAME' ACDLabs 10.04 '5-(acetylamino)-3,5-dideoxy-D-glycero-alpha-D-galacto-non-2-ulopyranosonic acid'\nSIA 'SYSTEMATIC NAME' 'OpenEye OEToolkits' 1.5.0 '(2R,4S,5R,6R)-5-acetamido-2,4-dihydroxy-6-[(1R,2R)-1,2,3-trihydroxypropyl]oxane-2-carboxylic acid'\nSIA 'CONDENSED IUPAC CARBOHYDRATE SYMBOL' GMML 1.0 DNeup5Aca\nSIA 'COMMON NAME' GMML 1.0 'N-acetyl-a-D-neuraminic acid'\nSIA 'IUPAC CARBOHYDRATE SYMBOL' PDB-CARE 1.0 a-D-Neup5Ac\nSIA 'SNFG CARBOHYDRATE SYMBOL' GMML 1.0 Neu5Ac\n#\nloop_\n_pdbx_chem_comp_feature.comp_id\n_pdbx_chem_comp_feature.type\n_pdbx_chem_comp_feature.value\n_pdbx_chem_comp_feature.source\n_pdbx_chem_comp_feature.support\nSIA 'CARBOHYDRATE ISOMER' D PDB ?\nSIA 'CARBOHYDRATE RING' pyranose PDB ?\nSIA 'CARBOHYDRATE ANOMER' alpha PDB ?\nSIA 'CARBOHYDRATE PRIMARY CARBONYL GROUP' ketose PDB ?\n#\nloop_\n_pdbx_chem_comp_audit.comp_id\n_pdbx_chem_comp_audit.action_type\n_pdbx_chem_comp_audit.date\n_pdbx_chem_comp_audit.processing_site\nSIA 'Create component' 1999-07-08 EBI\nSIA 'Modify descriptor' 2011-06-04 RCSB\nSIA 'Other modification' 2019-08-12 RCSB\nSIA 'Other modification' 2019-12-19 RCSB\nSIA 'Other modification' 2020-07-03 RCSB\nSIA 'Modify name' 2020-07-17 RCSB\nSIA 'Modify synonyms' 2020-07-17 RCSB\nSIA 'Modify atom id' 2020-07-17 RCSB\nSIA 'Modify component atom id' 2020-07-17 RCSB\nSIA 'Modify PCM' 2024-09-27 PDBE\n#\n_pdbx_chem_comp_pcm.pcm_id 1\n_pdbx_chem_comp_pcm.comp_id SIA\n_pdbx_chem_comp_pcm.modified_residue_id THR\n_pdbx_chem_comp_pcm.type None\n_pdbx_chem_comp_pcm.category Carbohydrate\n_pdbx_chem_comp_pcm.position 'Amino-acid side chain'\n_pdbx_chem_comp_pcm.polypeptide_position 'Any position'\n_pdbx_chem_comp_pcm.comp_id_linking_atom C2\n_pdbx_chem_comp_pcm.modified_residue_id_linking_atom OG1\n_pdbx_chem_comp_pcm.uniprot_specific_ptm_accession ?\n_pdbx_chem_comp_pcm.uniprot_generic_ptm_accession ?\n#\n_pdbe_chem_comp_drugbank_details.comp_id SIA\n_pdbe_chem_comp_drugbank_details.drugbank_id DB03721\n_pdbe_chem_comp_drugbank_details.type 'small molecule'\n_pdbe_chem_comp_drugbank_details.name 'N-acetyl-alpha-neuraminic acid'\n_pdbe_chem_comp_drugbank_details.description\n'An N-acyl derivative of neuraminic acid. N-acetylneuraminic acid occurs in many polysaccharides, glycoproteins, and glycolipids in animals and bacteria. (From Dorland, 28th ed, p1518)'\n_pdbe_chem_comp_drugbank_details.cas_number 21646-00-4\n_pdbe_chem_comp_drugbank_details.mechanism_of_action ?\n#\nloop_\n_pdbe_chem_comp_synonyms.comp_id\n_pdbe_chem_comp_synonyms.name\n_pdbe_chem_comp_synonyms.provenance\n_pdbe_chem_comp_synonyms.type\nSIA 'N-acetylneuraminic acid' wwPDB ?\nSIA 'sialic acid' wwPDB ?\nSIA 'alpha-sialic acid' wwPDB ?\nSIA 'O-SIALIC ACID' wwPDB ?\nSIA 'N-Acetyl-alpha-D-neuraminic acid' DrugBank ?\nSIA 'O-sialic acid' DrugBank ?\nSIA 'α-Neu5Ac' DrugBank ?\n#\n_pdbe_chem_comp_drugbank_classification.comp_id SIA\n_pdbe_chem_comp_drugbank_classification.drugbank_id DB03721\n_pdbe_chem_comp_drugbank_classification.parent 'N-acylneuraminic acids'\n_pdbe_chem_comp_drugbank_classification.kingdom 'Organic compounds'\n_pdbe_chem_comp_drugbank_classification.class

'Organooxygen
compounds'\n_pdbe_chem_comp_drugbank_classification.superclass
'Organic oxygen
compounds'\n_pdbe_chem_comp_drugbank_classification.description\n'This
compound belongs to the class of organic compounds known as n-
acylneuraminic acids. These are neuraminic acids carrying an N-acyl
substituent.'\n#\nloop_\n_pdbe_chem_comp_drugbank_targets.comp_id\n_pd
be_chem_comp_drugbank_targets.drugbank_id\n_pdbe_chem_comp_drugbank_ta
rgets.name\n_pdbe_chem_comp_drugbank_targets.organism\n_pdbe_chem_comp
_drugbank_targets.uniprot_id\n_pdbe_chem_comp_drugbank_targets.pharmac
ologically_active\n_pdbe_chem_comp_drugbank_targets.ordinal\nSIA
DB03721 P-selectin Humans P16109 yes 1\nSIA DB03721 E-selectin Humans
P16581 yes 2\nSIA DB03721 'Liver carboxylesterase 1' Humans P23141 yes
3\nSIA DB03721 '3-deoxy-manno-octulosonate cytidylyltransferase'
'Escherichia coli' P42216 unknown 4\nSIA DB03721 'Tetanus toxin'
'Clostridium tetani (strain Massachusetts / E88)' P04958 unknown
5\nSIA DB03721 'Cholera enterotoxin subunit B' 'Vibrio cholerae
serotype O1 (strain ATCC 39315 / El Tor Inaba N16961)' P01556 unknown
6\nSIA DB03721 'Botulinum neurotoxin type B' 'Clostridium botulinum'
P10844 unknown 7\nSIA DB03721 'Mannose-binding protein C' Humans
P11226 unknown 8\nSIA DB03721 Lithostathine-1-alpha Humans P05451
unknown 9\nSIA DB03721 Endo-N-acetylneuraminidase 'Enterobacteria
phage K1F' Q04830 unknown 10\nSIA DB03721 'Enterotoxin type B'
'Staphylococcus aureus' P01552 unknown 11\nSIA DB03721 Neuraminidase
'Influenza A virus (strain A/Tern/Australia/G70C/1975 H11N9)' P03472
unknown 12\nSIA DB03721 Hemagglutinin-neuraminidase NDV P32884 unknown
13\nSIA DB03721 Fiber 'Human adenovirus 19' Q64822 unknown 14\nSIA
DB03721 Sialoadhesin Humans Q9BZZ2 unknown 15\nSIA DB03721 Zinc-alpha-
2-glycoprotein Humans P25311 unknown 16\nSIA DB03721 'Capsid protein
VP1' MPyV P49302 unknown 17\nSIA DB03721 Fiber 'Human adenovirus D37'
Q64823 unknown
18\n#\nloop_\n_software.name\n_software.version\n_software.description
\nrdkit 2023.09.6 'Core functionality.'\npdbeccdutils 0.8.6 'Wrapper
to provide 2D templates and molecular
fragments.'\n#\nloop_\n_pdbe_chem_comp_atom_depiction.comp_id\n_pdbe_c
hem_comp_atom_depiction.atom_id\n_pdbe_chem_comp_atom_depiction.elemen
t\n_pdbe_chem_comp_atom_depiction.model_Cartn_x\n_pdbe_chem_comp_atom_
depiction.model_Cartn_y\n_pdbe_chem_comp_atom_depiction.pdbx_ordinal\n
SIA C1 C 5.654 -3.375 1\nSIA C2 C 6.404 -2.076 2\nSIA C3 C 5.104 -
1.326 3\nSIA C4 C 5.104 0.174 4\nSIA C5 C 6.404 0.924 5\nSIA C6 C
7.702 0.174 6\nSIA C7 C 9.002 0.924 7\nSIA C8 C 10.301 0.174 8\nSIA C9
C 11.600 0.924 9\nSIA C10 C 5.104 3.174 10\nSIA C11 C 3.805 2.424
11\nSIA N5 N 6.404 2.424 12\nSIA O1A O 4.154 -3.375 13\nSIA O1B O
6.404 -4.674 14\nSIA O4 O 3.805 0.924 16\nSIA O6 O 7.702 -1.326
17\nSIA O7 O 9.002 2.424 18\nSIA O8 O 10.301 -1.326 19\nSIA O9 O
12.899 0.174 20\nSIA O10 O 5.104 4.674
21\n#\nloop_\n_pdbe_chem_comp_bond_depiction.comp_id\n_pdbe_chem_comp_
bond_depiction.atom_id_1\n_pdbe_chem_comp_bond_depiction.atom_id_2\n_p
dbe_chem_comp_bond_depiction.value_order\n_pdbe_chem_comp_bond_depicti
on.bond_dir\n_pdbe_chem_comp_bond_depiction.pdbx_ordinal\nSIA C1 C2
SINGLE NONE 1\nSIA C1 O1A DOUBLE NONE 2\nSIA C1 O1B SINGLE NONE 3\nSIA
C2 C3 SINGLE NONE 4\nSIA C2 O6 SINGLE NONE 6\nSIA C3 C4 SINGLE NONE

7\nSIA C4 C5 SINGLE NONE 8\nSIA C4 O4 SINGLE BEGINDASH 9\nSIA C5 C6 SINGLE NONE 10\nSIA C5 N5 SINGLE BEGINWEDGE 11\nSIA C6 C7 SINGLE NONE 12\nSIA C6 O6 SINGLE BEGINDASH 13\nSIA C7 C8 SINGLE NONE 14\nSIA C7 O7 SINGLE BEGINDASH 15\nSIA C8 C9 SINGLE NONE 16\nSIA C8 O8 SINGLE BEGINWEDGE 17\nSIA C9 O9 SINGLE NONE 18\nSIA C10 C11 SINGLE NONE 19\nSIA C10 N5 SINGLE NONE 20\nSIA C10 O10 DOUBLE NONE 21\n#\nloop_\n_pdbe_chem_comp_substructure.comp_id\n_pdbe_chem_comp_substructure.substructure_name\n_pdbe_chem_comp_substructure.id\n_pdbe_chem_comp_substructure.substructure_type\n_pdbe_chem_comp_substructure.substructure_smiles\n_pdbe_chem_comp_substructure.substructure_inchis\n_pdbe_chem_comp_substructure.substructure_inchikeys\nSIA MurckoScaffold S1 scaffold C1CCOCC1 InChI=1S/C5H10O/c1-2-4-6-5-3-1/h1-5H2 DHXVGJBLRPWPCS-UHFFFAOYSA-N\nSIA amide F1 fragment CC(N)=O InChI=1S/C2H5NO/c1-2(3)4/h1H3,(H2,3,4) DLFVBJFMPXGRIB-UHFFFAOYSA-N\nSIA pyranose F2 fragment OC1CCCCO1 InChI=1S/C5H10O2/c6-5-3-1-2-4-7-5/h5-6H,1-4H2 CELWCAITJAEQNL-UHFFFAOYSA-N\n#\nloop_\n_pdbe_chem_comp_substructure_mapping.comp_id\n_pdbe_chem_comp_substructure_mapping.atom_id\n_pdbe_chem_comp_substructure_mapping.substructure_id\n_pdbe_chem_comp_substructure_mapping.substructure_ordinal\nSIA C2 S1 1\nSIA C3 S1 1\nSIA C4 S1 1\nSIA C5 S1 1\nSIA C6 S1 1\nSIA O6 S1 1\nSIA N5 F1 1\nSIA C10 F1 1\nSIA O10 F1 1\nSIA C11 F1 1\nSIA C5 F2 1\nSIA C6 F2 1\nSIA O6 F2 1\nSIA C2 F2 1\nSIA C3 F2 1\nSIA C4 F2 1\n#\n_pdbe_chem_comp_rdkit_properties.comp_id SIA\n_pdbe_chem_comp_rdkit_properties.exactmw 309.106\n_pdbe_chem_comp_rdkit_properties.amw 309.271\n_pdbe_chem_comp_rdkit_properties.lipinskiHBA 10\n_pdbe_chem_comp_rdkit_properties.lipinskiHBD 7\n_pdbe_chem_comp_rdkit_properties.NumRotatableBonds 11\n_pdbe_chem_comp_rdkit_properties.NumHBD 7\n_pdbe_chem_comp_rdkit_properties.NumHBA 9\n_pdbe_chem_comp_rdkit_properties.NumHeavyAtoms 21\n_pdbe_chem_comp_rdkit_properties.NumAtoms 40\n_pdbe_chem_comp_rdkit_properties.NumHeteroatoms 10\n_pdbe_chem_comp_rdkit_properties.NumAmideBonds 1\n_pdbe_chem_comp_rdkit_properties.FractionCSP3 0.818\n_pdbe_chem_comp_rdkit_properties.NumRings 1\n_pdbe_chem_comp_rdkit_properties.NumAromaticRings 0\n_pdbe_chem_comp_rdkit_properties.NumAliphaticRings 1\n_pdbe_chem_comp_rdkit_properties.NumSaturatedRings 1\n_pdbe_chem_comp_rdkit_properties.NumHeterocycles 1\n_pdbe_chem_comp_rdkit_properties.NumAromaticHeterocycles 0\n_pdbe_chem_comp_rdkit_properties.NumSaturatedHeterocycles 1\n_pdbe_chem_comp_rdkit_properties.NumAliphaticHeterocycles 1\n_pdbe_chem_comp_rdkit_properties.NumSpiroAtoms 0\n_pdbe_chem_comp_rdkit_properties.NumBridgeheadAtoms 0\n_pdbe_chem_comp_rdkit_properties.NumAtomStereoCenters 6\n_pdbe_chem_comp_rdkit_properties.NumUnspecifiedAtomStereoCenters 0\n_pdbe_chem_comp_rdkit_properties.labuteASA 146.407\n_pdbe_chem_comp_rdkit_properties.tpsa 176.780\n_pdbe_chem_comp_rdkit_properties.CrippenClogP -3.872\n_pdbe_chem_comp_rdkit_properties.CrippenMR 64.787\n_pdbe_chem_comp_rdkit_properties.chi0v

9.621\n_pdbe_chem_comp_rdkit_properties.chi1v
4.738\n_pdbe_chem_comp_rdkit_properties.chi2v
1.941\n_pdbe_chem_comp_rdkit_properties.chi3v
1.941\n_pdbe_chem_comp_rdkit_properties.chi4v
1.128\n_pdbe_chem_comp_rdkit_properties.chi0n
28.621\n_pdbe_chem_comp_rdkit_properties.chi1n
13.635\n_pdbe_chem_comp_rdkit_properties.chi2n
1.941\n_pdbe_chem_comp_rdkit_properties.chi3n
1.941\n_pdbe_chem_comp_rdkit_properties.chi4n
1.128\n_pdbe_chem_comp_rdkit_properties.hallKierAlpha -
1.300\n_pdbe_chem_comp_rdkit_properties.kappa1
4.600\n_pdbe_chem_comp_rdkit_properties.kappa2
6.642\n_pdbe_chem_comp_rdkit_properties.kappa3
3.872\n_pdbe_chem_comp_rdkit_properties.Phi
1.455\n#\nloop_\n_pdbe_chem_comp_external_mappings.comp_id\n_pdbe_chem
_comp_external_mappings.source\n_pdbe_chem_comp_external_mappings.reso
urce\n_pdbe_chem_comp_external_mappings.resource_id\nSIA UniChem
ChEMBL CHEMBL1234621\nSIA UniChem DrugBank DB03721\nSIA UniChem ChEBI
49026\nSIA UniChem ZINC ZINC000004081651\nSIA UniChem fdasrs
04A90EXP8V\nSIA UniChem HMDB HMDB0000773\nSIA UniChem Nikkaji
J614.853K\nSIA UniChem MetaboLights MTBLC49026\nSIA UniChem BRENDA
141715\nSIA UniChem BRENDA 233672\nSIA UniChem BRENDA 6105\nSIA
UniChem BRENDA 84245\nSIA UniChem BRENDA 85625\nSIA UniChem 'Probes
And Drugs' PD041137\nSIA UniChem PubChem 444885\nSIA UniChem
eMolecules 474793\nSIA UniChem SureChEMBL SCHEMBL79085\nSIA UniChem
'PubChem TPHARMA' 14776495\nSIA UniChem 'PubChem TPHARMA'
15395566\n#\nloop_\n_pdbe_chem_comp_rdkit_conformer.comp_id\n_pdbe_che
m_comp_rdkit_conformer.atom_id\n_pdbe_chem_comp_rdkit_conformer.Cartn_
x_rdkit\n_pdbe_chem_comp_rdkit_conformer.Cartn_y_rdkit\n_pdbe_chem_com
p_rdkit_conformer.Cartn_z_rdkit\n_pdbe_chem_comp_rdkit_conformer.rdkit
_method\n_pdbe_chem_comp_rdkit_conformer.rdkit_ordinal\nSIA C1 -1.164
-2.811 1.058 ETKDGv3 1\nSIA C2 -1.666 -1.928 -0.070 ETKDGv3 2\nSIA C3
-2.639 -0.809 0.442 ETKDGv3 3\nSIA C4 -2.546 0.454 -0.428 ETKDGv3
4\nSIA C5 -1.079 0.960 -0.528 ETKDGv3 5\nSIA C6 -0.071 -0.181 -0.219
ETKDGv3 6\nSIA C7 1.341 0.133 -0.777 ETKDGv3 7\nSIA C8 2.378 -0.960 -
0.400 ETKDGv3 8\nSIA C9 3.769 -0.651 -0.979 ETKDGv3 9\nSIA C10 -0.223
3.295 0.052 ETKDGv3 10\nSIA C11 0.035 4.333 1.094 ETKDGv3 11\nSIA N5 -
0.868 2.066 0.414 ETKDGv3 12\nSIA O1A -0.936 -2.335 2.203 ETKDGv3
13\nSIA O1B -0.875 -4.149 0.806 ETKDGv3 14\nSIA O4 -3.104 0.224 -1.700
ETKDGv3 16\nSIA O6 -0.537 -1.404 -0.765 ETKDGv3 17\nSIA O7 1.298 0.320
-2.172 ETKDGv3 18\nSIA O8 2.484 -1.066 0.998 ETKDGv3 19\nSIA O9 4.246
0.587 -0.524 ETKDGv3 20\nSIA O10 0.157 3.497 -1.133 ETKDGv3 21\nSIA
H32 -2.392 -0.510 1.484 ETKDGv3 22\nSIA H31 -3.691 -1.171 0.465
ETKDGv3 23\nSIA H4 -3.171 1.239 0.053 ETKDGv3 24\nSIA H5 -0.925 1.324
-1.568 ETKDGv3 25\nSIA H6 0.030 -0.270 0.887 ETKDGv3 26\nSIA H7 1.678
1.089 -0.320 ETKDGv3 27\nSIA H8 2.053 -1.935 -0.838 ETKDGv3 28\nSIA
H92 3.719 -0.629 -2.088 ETKDGv3 29\nSIA H91 4.478 -1.464 -0.703
ETKDGv3 30\nSIA H111 0.843 3.984 1.770 ETKDGv3 31\nSIA H113 -0.888
4.508 1.686 ETKDGv3 32\nSIA H112 0.344 5.291 0.624 ETKDGv3 33\nSIA HN5
-1.113 1.913 1.419 ETKDGv3 34\nSIA HO1B -0.511 -4.749 1.536 ETKDGv3
35\nSIA HO4 -2.492 -0.372 -2.205 ETKDGv3 37\nSIA HO7 1.040 -0.545 -

2.585 ETKDGv3 38\nSIA HO8 1.886 -1.804 1.285 ETKDGv3 39\nSIA HO9 4.592 0.441 0.395 ETKDGv3 40\n#\n"
}

Fig. 5g. HsGPC1 carrying HS


```
{
    "name": "gpc1_hs_9a_7",
    "modelSeeds": [
      1, 2, 3, 4, 5, 6, 7, 8, 9, 10
    ],
    "sequences": [
      {
        "protein": {
          "sequence":
"DPASKSRSCGEVRQIYGAKGFSLSDVPQAEISGEHLRICPQGYTCCTSEMEENLANRSHAELETALRDS
SRVLQAMLATQLRSFDDHFQHLLNDSERTLQATFPGAFGELYTQNARAFRDLYSELRLYYRGANLHLEET
LAEFWARLLERLFKQLHPQLLLPDDYLDCLGKQAEALRPFGEAPRELRLRATRAFVAARSFVQGLGVASD
VVRKVAQVPLGPECSRAVMKLVYCAHCLGVPGARPCPDYCRNVLKGCLANQADLDAEWRNLLDSMVLITD
KFWGTSGVESVIGSVHTWLAEAINALQDNRDTLTAKVIQGCGNPKVNPQGPGPEEKRRRGKLAPRERPPS
GTLEKLVSEAKAQLRDVQDFWISLPGTLCSEKMALSTASDDRCWNGMARGRYLPEVMGDGLANQINNPEV
EVDITKPDMTIRQQIMQLKIMTNRLRSAYNGNDVDFQDASDDGSGSGSGDGCLDDLCSRKVSRKSSSSRT
PLTHALPGLSEQEGQKTS",
          "id": ["GPC"]
        }
      },      {
        "ligand": {
          "ccdCodes": [
            "XYP", "PO4-2", "GAL", "GAL", "BDP", "NDG", "BDP"
          ],
          "id": "HS"
        }
      }
    ],
    "dialect": "alphafold3",
    "version": 2,
    "bondedAtomPairs": [
    [["GPC",465,"OG"],["HS",1,"C1"]],
    [["HS",1,"O2"],["HS",2,"P"]],
    [["HS",1,"O4"],["HS",3,"C1"]],
    [["HS",3,"O3"],["HS",4,"C1"]],
    [["HS",4,"O3"],["HS",5,"C1"]],
    [["HS",5,"O4"],["HS",6,"C1"]],
    [["HS",6,"O4"],["HS",7,"C1"]]
    ],
    "userCCD": "data_PO4-2\n#\n_chem_comp.id PO4\n_chem_comp.name
'PHOSPHATE ION'\n_chem_comp.type NON-POLYMER\n_chem_comp.pdbx_type
HETAI\n_chem_comp.formula 'O4
P'\n_chem_comp.mon_nstd_parent_comp_id ?\n_chem_comp.pdbx_synonyms ?\n
_chem_comp.pdbx_formal_charge -3\n_chem_comp.pdbx_initial_date 1999-
07-08\n_chem_comp.pdbx_modified_date 2011-06-
04\n_chem_comp.pdbx_ambiguous_flag N\n_chem_comp.pdbx_release_status
REL\n_chem_comp.pdbx_replaced_by ?\n_chem_comp.pdbx_replaces
IPS\n_chem_comp.formula_weight
94.971\n_chem_comp.one_letter_code ?\n_chem_comp.three_letter_code
PO4\n_chem_comp.pdbx_model_coordinates_details ?\n_chem_comp.pdbx_mode
l_coordinates_missing_flag
```

N\n_chem_comp.pdbx_ideal_coordinates_details ?\n_chem_comp.pdbx_ideal_
coordinates_missing_flag N\n_chem_comp.pdbx_model_coordinates_db_code
1IXG\n_chem_comp.pdbx_subcomponent_list ?\n_chem_comp.pdbx_processing_
site
EBI\n#\nloop_\n_chem_comp_atom.comp_id\n_chem_comp_atom.atom_id\n_chem
_comp_atom.alt_atom_id\n_chem_comp_atom.type_symbol\n_chem_comp_atom.c
harge\n_chem_comp_atom.pdbx_align\n_chem_comp_atom.pdbx_aromatic_flag\
n_chem_comp_atom.pdbx_leaving_atom_flag\n_chem_comp_atom.pdbx_stereo_c
onfig\n_chem_comp_atom.pdbx_backbone_atom_flag\n_chem_comp_atom.pdbx_n
_terminal_atom_flag\n_chem_comp_atom.pdbx_c_terminal_atom_flag\n_chem_
comp_atom.model_Cartn_x\n_chem_comp_atom.model_Cartn_y\n_chem_comp_ato
m.model_Cartn_z\n_chem_comp_atom.pdbx_model_Cartn_x_ideal\n_chem_comp_
atom.pdbx_model_Cartn_y_ideal\n_chem_comp_atom.pdbx_model_Cartn_z_idea
l\n_chem_comp_atom.pdbx_component_atom_id\n_chem_comp_atom.pdbx_compon
ent_comp_id\n_chem_comp_atom.pdbx_ordinal\nPO4 P P P 0 1 N N N N N N
29.995 23.516 13.249 0.000 0.000 0.000 P PO4 1\nPO4 O1 O1 O 0 1 N N N
N N N 31.092 22.988 14.164 0.000 -1.288 -0.911 O1 PO4 2\nPO4 O3 O3 O -
1 1 N N N N N N 29.646 22.518 12.126 -1.288 0.000 0.911 O3 PO4 4\nPO4
O4 O4 O -1 1 N N N N N N 28.727 23.744 14.161 1.288 0.000 0.911 O4 PO4
5\n#\nloop_\n_chem_comp_bond.comp_id\n_chem_comp_bond.atom_id_1\n_chem
_comp_bond.atom_id_2\n_chem_comp_bond.value_order\n_chem_comp_bond.pdb
x_aromatic_flag\n_chem_comp_bond.pdbx_stereo_config\n_chem_comp_bond.p
dbx_ordinal\nPO4 P O1 DOUB N N 1\nPO4 P O3 SING N N 3\nPO4 P O4 SING N
N
4\n#\nloop_\n_pdbx_chem_comp_descriptor.comp_id\n_pdbx_chem_comp_descr
iptor.type\n_pdbx_chem_comp_descriptor.program\n_pdbx_chem_comp_descri
ptor.program_version\n_pdbx_chem_comp_descriptor.descriptor\nPO4
SMILES ACDLabs 10.04 '[O-]P([O-])([O-])=O'\nPO4 SMILES_CANONICAL
CACTVS 3.341 '[O-][P]([O-])([O-])=O'\nPO4 SMILES CACTVS 3.341
'[O-][P]([O-])([O-])=O'\nPO4 SMILES_CANONICAL 'OpenEye OEToolkits'
1.5.0 '[O-]P(=O)([O-])[O-]'\nPO4 SMILES 'OpenEye OEToolkits' 1.5.0
'[O-]P(=O)([O-])[O-]'\nPO4 InChI InChI 1.03 InChI=1S/H3O4P/c1-
5(2,3)4/h(H3,1,2,3,4)/p-3\nPO4 InChIKey InChI 1.03 NBIIXXVUZAFLBC-
UHFFFAOYSA-
K\n#\nloop_\n_pdbx_chem_comp_identifier.comp_id\n_pdbx_chem_comp_ident
ifier.type\n_pdbx_chem_comp_identifier.program\n_pdbx_chem_comp_identi
fier.program_version\n_pdbx_chem_comp_identifier.identifier\nPO4
'SYSTEMATIC NAME' ACDLabs 10.04 phosphate\nPO4 'SYSTEMATIC NAME'
'OpenEye OEToolkits' 1.5.0
phosphate\n#\nloop_\n_pdbx_chem_comp_audit.comp_id\n_pdbx_chem_comp_au
dit.action_type\n_pdbx_chem_comp_audit.date\n_pdbx_chem_comp_audit.pro
cessing_site\nPO4 'Create component' 1999-07-08 EBI\nPO4 'Modify
descriptor' 2011-06-04
RCSB\n#\n_pdbe_chem_comp_drugbank_details.comp_id
PO4\n_pdbe_chem_comp_drugbank_details.drugbank_id
DB14523\n_pdbe_chem_comp_drugbank_details.type 'small
molecule'\n_pdbe_chem_comp_drugbank_details.name 'Phosphate
ion'\n_pdbe_chem_comp_drugbank_details.description ?\n_pdbe_chem_comp_
drugbank_details.cas_number 14265-44-
2\n_pdbe_chem_comp_drugbank_details.mechanism_of_action ?\n#\nloop_\n_
pdbe_chem_comp_synonyms.comp_id\n_pdbe_chem_comp_synonyms.name\n_pdbe_
chem_comp_synonyms.provenance\n_pdbe_chem_comp_synonyms.type\nPO4

Orthophosphate DrugBank ?\nPO4 Phosphate
DrugBank ?\n#\n_pdbe_chem_comp_drugbank_classification.comp_id
PO4\n_pdbe_chem_comp_drugbank_classification.drugbank_id
DB14523\n_pdbe_chem_comp_drugbank_classification.parent 'Non-metal
phosphates'\n_pdbe_chem_comp_drugbank_classification.kingdom
'Inorganic compounds'\n_pdbe_chem_comp_drugbank_classification.class
'Non-metal oxoanionic
compounds'\n_pdbe_chem_comp_drugbank_classification.superclass
'Homogeneous non-metal
compounds'\n_pdbe_chem_comp_drugbank_classification.description\n'This
compound belongs to the class of inorganic compounds known as non-
metal phosphates. These are inorganic non-metallic compounds
containing a phosphate as its largest
oxoanion.'\n#\nloop_\n_software.name\n_software.version\n_software.des
cription\nrdkit 2023.09.6 'Core functionality.'\npdbeccdutils 0.8.6
'Wrapper to provide 2D templates and molecular
fragments.'\n#\nloop_\n_pdbe_chem_comp_atom_depiction.comp_id\n_pdbe_c
hem_comp_atom_depiction.atom_id\n_pdbe_chem_comp_atom_depiction.elemen
t\n_pdbe_chem_comp_atom_depiction.model_Cartn_x\n_pdbe_chem_comp_atom_
depiction.model_Cartn_y\n_pdbe_chem_comp_atom_depiction.pdbx_ordinal\n
PO4 P P 4.299 0.000 1\nPO4 O1 O 5.598 0.750 2\nPO4 O3 O 3.549 1.299
4\nPO4 O4 O 5.049 -1.299
5\n#\nloop_\n_pdbe_chem_comp_bond_depiction.comp_id\n_pdbe_chem_comp_b
ond_depiction.atom_id_1\n_pdbe_chem_comp_bond_depiction.atom_id_2\n_pd
be_chem_comp_bond_depiction.value_order\n_pdbe_chem_comp_bond_depictio
n.bond_dir\n_pdbe_chem_comp_bond_depiction.pdbx_ordinal\nPO4 P O1
DOUBLE NONE 1\nPO4 P O3 SINGLE NONE 3\nPO4 P O4 SINGLE NONE
4\n#\n_pdbe_chem_comp_substructure.comp_id
PO4\n_pdbe_chem_comp_substructure.substructure_name
phosphate\n_pdbe_chem_comp_substructure.id
F1\n_pdbe_chem_comp_substructure.substructure_type
fragment\n_pdbe_chem_comp_substructure.substructure_smiles
O=P(O)(O)O\n_pdbe_chem_comp_substructure.substructure_inchis
InChI=1S/H3O4P/c1-
5(2,3)4/h(H3,1,2,3,4)\n_pdbe_chem_comp_substructure.substructure_inchi
keys NBIIXXVUZAFLBC-UHFFFAOYSA-
N\n#\nloop_\n_pdbe_chem_comp_substructure_mapping.comp_id\n_pdbe_chem_
comp_substructure_mapping.atom_id\n_pdbe_chem_comp_substructure_mappin
g.substructure_id\n_pdbe_chem_comp_substructure_mapping.substructure_o
rdinal\nPO4 P F1 1\nPO4 O1 F1 1\nPO4 O3 F1 1\nPO4 O4 F1
1\n#\n_pdbe_chem_comp_rdkit_properties.comp_id
PO4\n_pdbe_chem_comp_rdkit_properties.exactmw
94.955\n_pdbe_chem_comp_rdkit_properties.amw
94.970\n_pdbe_chem_comp_rdkit_properties.lipinskiHBA
4\n_pdbe_chem_comp_rdkit_properties.lipinskiHBD
0\n_pdbe_chem_comp_rdkit_properties.NumRotatableBonds
0\n_pdbe_chem_comp_rdkit_properties.NumHBD
0\n_pdbe_chem_comp_rdkit_properties.NumHBA
4\n_pdbe_chem_comp_rdkit_properties.NumHeavyAtoms
5\n_pdbe_chem_comp_rdkit_properties.NumAtoms
5\n_pdbe_chem_comp_rdkit_properties.NumHeteroatoms
5\n_pdbe_chem_comp_rdkit_properties.NumAmideBonds

0\n_pdbe_chem_comp_rdkit_properties.FractionCSP3
0\n_pdbe_chem_comp_rdkit_properties.NumRings
0\n_pdbe_chem_comp_rdkit_properties.NumAromaticRings
0\n_pdbe_chem_comp_rdkit_properties.NumAliphaticRings
0\n_pdbe_chem_comp_rdkit_properties.NumSaturatedRings
0\n_pdbe_chem_comp_rdkit_properties.NumHeterocycles
0\n_pdbe_chem_comp_rdkit_properties.NumAromaticHeterocycles
0\n_pdbe_chem_comp_rdkit_properties.NumSaturatedHeterocycles
0\n_pdbe_chem_comp_rdkit_properties.NumAliphaticHeterocycles
0\n_pdbe_chem_comp_rdkit_properties.NumSpiroAtoms
0\n_pdbe_chem_comp_rdkit_properties.NumBridgeheadAtoms
0\n_pdbe_chem_comp_rdkit_properties.NumAtomStereoCenters
0\n_pdbe_chem_comp_rdkit_properties.NumUnspecifiedAtomStereoCenters
0\n_pdbe_chem_comp_rdkit_properties.labuteASA
28.307\n_pdbe_chem_comp_rdkit_properties.tpsa
86.250\n_pdbe_chem_comp_rdkit_properties.CrippenClogP -
2.825\n_pdbe_chem_comp_rdkit_properties.CrippenMR
7.606\n_pdbe_chem_comp_rdkit_properties.chi0v
2.975\n_pdbe_chem_comp_rdkit_properties.chi1v
2.191\n_pdbe_chem_comp_rdkit_properties.chi2v
0\n_pdbe_chem_comp_rdkit_properties.chi3v
0\n_pdbe_chem_comp_rdkit_properties.chi4v
0\n_pdbe_chem_comp_rdkit_properties.chi0n
2.080\n_pdbe_chem_comp_rdkit_properties.chi1n
0.730\n_pdbe_chem_comp_rdkit_properties.chi2n
0\n_pdbe_chem_comp_rdkit_properties.chi3n
0\n_pdbe_chem_comp_rdkit_properties.chi4n
0\n_pdbe_chem_comp_rdkit_properties.hallKierAlpha
0.110\n_pdbe_chem_comp_rdkit_properties.kappa1
5.110\n_pdbe_chem_comp_rdkit_properties.kappa2
1.065\n_pdbe_chem_comp_rdkit_properties.kappa3
1512.242\n_pdbe_chem_comp_rdkit_properties.Phi
1.088\n#\nloop_\n_pdbe_chem_comp_external_mappings.comp_id\n_pdbe_chem_comp_external_mappings.source\n_pdbe_chem_comp_external_mappings.resource\n_pdbe_chem_comp_external_mappings.resource_id\nPO4 UniChem DrugBank DB14523\nPO4 UniChem ChEBI 18367\nPO4 UniChem eMolecules 882178\nPO4 UniChem fdasrs NK08V8K8HR\nPO4 UniChem NMRShiftDB 30000818\nPO4 UniChem BindingDb 50155537\nPO4 UniChem MetaboLights MTBLC18367\nPO4 UniChem BRENDA 21671\nPO4 UniChem BRENDA 867\nPO4 UniChem ChemicalBook CB3409949\nPO4 UniChem ChemicalBook CB7191961\nPO4 UniChem rxnorm 'PHOSPHATE ION'\nPO4 UniChem 'Probes And Drugs' PD094531\nPO4 UniChem CCDC EXIZIX\nPO4 UniChem 'EPA CompTox Dashboard' DTXSID7039672\nPO4 UniChem 'PubChem TPHARMA' 15218698\nPO4 UniChem PubChem 1061\nPO4 UniChem ACTor 14265-44-2\nPO4 UniChem Nikkaji J215.970H\nPO4 UniChem Nikkaji J351.173A\n#\nloop_\n_pdbe_chem_comp_rdkit_conformer.comp_id\n_pdbe_chem_comp_rdkit_conformer.atom_id\n_pdbe_chem_comp_rdkit_conformer.Cartn_x_rdkit\n_pdbe_chem_comp_rdkit_conformer.Cartn_y_rdkit\n_pdbe_chem_comp_rdkit_conformer.Cartn_z_rdkit\n_pdbe_chem_comp_rdkit_conformer.rdkit_method\n_pdbe_chem_comp_rdkit_conformer.rdkit_ordinal\nPO4 P -0.003 0.002 0.036 ETKDGv3 1\nPO4 O1 -0.117 0.079 1.541 ETKDGv3 2\nPO4 O3 -

```
1.075 -1.165 -0.552 ETKDGv3 4\nPO4 O4 -0.383 1.506 -0.636 ETKDGv3
5\n#\n"
  }
```

Fig. 5h. HsBikunin carrying CS


```
{
    "name": "ambp_light_cs_7",
    "modelSeeds": [
      1, 2, 3, 4, 5, 6, 7, 8, 9, 10
    ],
    "sequences": [
      {
        "protein": {
          "sequence":
"AVLPQEEEGSGGGQLVTEVTKKEDSCQLGYSAGPCMGMTSRYFYNGTSMACETFQYGGCMGNGNNFVTE
KECLQTCRTVAACNLPIVRGPCRAFIQLWAFDAVKGKCVLFPYGGCQGNGNKFYSEKECREYCGVPGDGD
EELLRFSN",
          "id": ["AMBP"]
        }
      },      {
        "ligand": {
          "ccdCodes": [
            "XYP", "PO4-2", "GAL", "GAL", "BDP", "NGA", "BDP"
          ],
          "id": "CS"
        }
      }
    ],
    "dialect": "alphafold3",
    "version": 2,
    "bondedAtomPairs": [
    [["AMBP",10,"OG"],["CS",1,"C1"]],
    [["CS",1,"O2"],["CS",2,"P"]],
    [["CS",1,"O4"],["CS",3,"C1"]],
    [["CS",3,"O3"],["CS",4,"C1"]],
    [["CS",4,"O3"],["CS",5,"C1"]],
    [["CS",5,"O4"],["CS",6,"C1"]],
    [["CS",6,"O3"],["CS",7,"C1"]]
    ],
    "userCCD": "data_PO4-2\n#\n_chem_comp.id PO4\n_chem_comp.name
'PHOSPHATE ION'\n_chem_comp.type NON-POLYMER\n_chem_comp.pdbx_type
HETAI\n_chem_comp.formula 'O4
P'\n_chem_comp.mon_nstd_parent_comp_id ?\n_chem_comp.pdbx_synonyms ?\n
_chem_comp.pdbx_formal_charge -3\n_chem_comp.pdbx_initial_date 1999-
07-08\n_chem_comp.pdbx_modified_date 2011-06-
04\n_chem_comp.pdbx_ambiguous_flag N\n_chem_comp.pdbx_release_status
REL\n_chem_comp.pdbx_replaced_by ?\n_chem_comp.pdbx_replaces
IPS\n_chem_comp.formula_weight
94.971\n_chem_comp.one_letter_code ?\n_chem_comp.three_letter_code
PO4\n_chem_comp.pdbx_model_coordinates_details ?\n_chem_comp.pdbx_mode
l_coordinates_missing_flag
N\n_chem_comp.pdbx_ideal_coordinates_details ?\n_chem_comp.pdbx_ideal_
coordinates_missing_flag N\n_chem_comp.pdbx_model_coordinates_db_code
1IXG\n_chem_comp.pdbx_subcomponent_list ?\n_chem_comp.pdbx_processing_
site
EBI\n#\nloop_\n_chem_comp_atom.comp_id\n_chem_comp_atom.atom_id\n_chem
```

_comp_atom.alt_atom_id\n_chem_comp_atom.type_symbol\n_chem_comp_atom.c
harge\n_chem_comp_atom.pdbx_align\n_chem_comp_atom.pdbx_aromatic_flag\
n_chem_comp_atom.pdbx_leaving_atom_flag\n_chem_comp_atom.pdbx_stereo_c
onfig\n_chem_comp_atom.pdbx_backbone_atom_flag\n_chem_comp_atom.pdbx_n
_terminal_atom_flag\n_chem_comp_atom.pdbx_c_terminal_atom_flag\n_chem_
comp_atom.model_Cartn_x\n_chem_comp_atom.model_Cartn_y\n_chem_comp_ato
m.model_Cartn_z\n_chem_comp_atom.pdbx_model_Cartn_x_ideal\n_chem_comp_
atom.pdbx_model_Cartn_y_ideal\n_chem_comp_atom.pdbx_model_Cartn_z_idea
l\n_chem_comp_atom.pdbx_component_atom_id\n_chem_comp_atom.pdbx_compon
ent_comp_id\n_chem_comp_atom.pdbx_ordinal\nPO4 P P P 0 1 N N N N N N
29.995 23.516 13.249 0.000 0.000 0.000 P PO4 1\nPO4 O1 O1 O 0 1 N N N
N N N 31.092 22.988 14.164 0.000 -1.288 -0.911 O1 PO4 2\nPO4 O3 O3 O -
1 1 N N N N N N 29.646 22.518 12.126 -1.288 0.000 0.911 O3 PO4 4\nPO4
O4 O4 O -1 1 N N N N N N 28.727 23.744 14.161 1.288 0.000 0.911 O4 PO4
5\n#\nloop_\n_chem_comp_bond.comp_id\n_chem_comp_bond.atom_id_1\n_chem
_comp_bond.atom_id_2\n_chem_comp_bond.value_order\n_chem_comp_bond.pdb
x_aromatic_flag\n_chem_comp_bond.pdbx_stereo_config\n_chem_comp_bond.p
dbx_ordinal\nPO4 P O1 DOUB N N 1\nPO4 P O3 SING N N 3\nPO4 P O4 SING N
N
4\n#\nloop_\n_pdbx_chem_comp_descriptor.comp_id\n_pdbx_chem_comp_descr
iptor.type\n_pdbx_chem_comp_descriptor.program\n_pdbx_chem_comp_descri
ptor.program_version\n_pdbx_chem_comp_descriptor.descriptor\nPO4
SMILES ACDLabs 10.04 '[O-]P([O-])([O-])=O'\nPO4 SMILES_CANONICAL
CACTVS 3.341 '[O-][P]([O-])([O-])=O'\nPO4 SMILES CACTVS 3.341
'[O-][P]([O-])([O-])=O'\nPO4 SMILES_CANONICAL 'OpenEye OEToolkits'
1.5.0 '[O-]P(=O)([O-])[O-]'\nPO4 SMILES 'OpenEye OEToolkits' 1.5.0
'[O-]P(=O)([O-])[O-]'\nPO4 InChI InChI 1.03 InChI=1S/H3O4P/c1-
5(2,3)4/h(H3,1,2,3,4)/p-3\nPO4 InChIKey InChI 1.03 NBIIXXVUZAFLBC-
UHFFFAOYSA-
K\n#\nloop_\n_pdbx_chem_comp_identifier.comp_id\n_pdbx_chem_comp_ident
ifier.type\n_pdbx_chem_comp_identifier.program\n_pdbx_chem_comp_identi
fier.program_version\n_pdbx_chem_comp_identifier.identifier\nPO4
'SYSTEMATIC NAME' ACDLabs 10.04 phosphate\nPO4 'SYSTEMATIC NAME'
'OpenEye OEToolkits' 1.5.0
phosphate\n#\nloop_\n_pdbx_chem_comp_audit.comp_id\n_pdbx_chem_comp_au
dit.action_type\n_pdbx_chem_comp_audit.date\n_pdbx_chem_comp_audit.pro
cessing_site\nPO4 'Create component' 1999-07-08 EBI\nPO4 'Modify
descriptor' 2011-06-04
RCSB\n#\n_pdbe_chem_comp_drugbank_details.comp_id
PO4\n_pdbe_chem_comp_drugbank_details.drugbank_id
DB14523\n_pdbe_chem_comp_drugbank_details.type 'small
molecule'\n_pdbe_chem_comp_drugbank_details.name 'Phosphate
ion'\n_pdbe_chem_comp_drugbank_details.description ?\n_pdbe_chem_comp_
drugbank_details.cas_number 14265-44-
2\n_pdbe_chem_comp_drugbank_details.mechanism_of_action ?\n#\nloop_\n_
pdbe_chem_comp_synonyms.comp_id\n_pdbe_chem_comp_synonyms.name\n_pdbe_
chem_comp_synonyms.provenance\n_pdbe_chem_comp_synonyms.type\nPO4
Orthophosphate DrugBank ?\nPO4 Phosphate
DrugBank ?\n#\n_pdbe_chem_comp_drugbank_classification.comp_id
PO4\n_pdbe_chem_comp_drugbank_classification.drugbank_id
DB14523\n_pdbe_chem_comp_drugbank_classification.parent 'Non-metal
phosphates'\n_pdbe_chem_comp_drugbank_classification.kingdom

'Inorganic compounds'\n_pdbe_chem_comp_drugbank_classification.class 'Non-metal oxoanionic compounds'\n_pdbe_chem_comp_drugbank_classification.superclass 'Homogeneous non-metal compounds'\n_pdbe_chem_comp_drugbank_classification.description\n'This compound belongs to the class of inorganic compounds known as non-metal phosphates. These are inorganic non-metallic compounds containing a phosphate as its largest oxoanion.'\n#\nloop_\n_software.name\n_software.version\n_software.description\nrdkit 2023.09.6 'Core functionality.'\npdbeccdutils 0.8.6 'Wrapper to provide 2D templates and molecular fragments.'\n#\nloop_\n_pdbe_chem_comp_atom_depiction.comp_id\n_pdbe_chem_comp_atom_depiction.atom_id\n_pdbe_chem_comp_atom_depiction.element\n_pdbe_chem_comp_atom_depiction.model_Cartn_x\n_pdbe_chem_comp_atom_depiction.model_Cartn_y\n_pdbe_chem_comp_atom_depiction.pdbx_ordinal\nPO4 P P 4.299 0.000 1\nPO4 O1 O 5.598 0.750 2\nPO4 O3 O 3.549 1.299 4\nPO4 O4 O 5.049 -1.299 5\n#\nloop_\n_pdbe_chem_comp_bond_depiction.comp_id\n_pdbe_chem_comp_bond_depiction.atom_id_1\n_pdbe_chem_comp_bond_depiction.atom_id_2\n_pdbe_chem_comp_bond_depiction.value_order\n_pdbe_chem_comp_bond_depiction.bond_dir\n_pdbe_chem_comp_bond_depiction.pdbx_ordinal\nPO4 P O1 DOUBLE NONE 1\nPO4 P O3 SINGLE NONE 3\nPO4 P O4 SINGLE NONE 4\n#\n_pdbe_chem_comp_substructure.comp_id PO4\n_pdbe_chem_comp_substructure.substructure_name phosphate\n_pdbe_chem_comp_substructure.id F1\n_pdbe_chem_comp_substructure.substructure_type fragment\n_pdbe_chem_comp_substructure.substructure_smiles O=P(O)(O)O\n_pdbe_chem_comp_substructure.substructure_inchis InChI=1S/H3O4P/c1-5(2,3)4/h(H3,1,2,3,4)\n_pdbe_chem_comp_substructure.substructure_inchikeys NBIIXXVUZAFLBC-UHFFFAOYSA-N\n#\nloop_\n_pdbe_chem_comp_substructure_mapping.comp_id\n_pdbe_chem_comp_substructure_mapping.atom_id\n_pdbe_chem_comp_substructure_mapping.substructure_id\n_pdbe_chem_comp_substructure_mapping.substructure_ordinal\nPO4 P F1 1\nPO4 O1 F1 1\nPO4 O3 F1 1\nPO4 O4 F1 1\n#\n_pdbe_chem_comp_rdkit_properties.comp_id PO4\n_pdbe_chem_comp_rdkit_properties.exactmw 94.955\n_pdbe_chem_comp_rdkit_properties.amw 94.970\n_pdbe_chem_comp_rdkit_properties.lipinskiHBA 4\n_pdbe_chem_comp_rdkit_properties.lipinskiHBD 0\n_pdbe_chem_comp_rdkit_properties.NumRotatableBonds 0\n_pdbe_chem_comp_rdkit_properties.NumHBD 0\n_pdbe_chem_comp_rdkit_properties.NumHBA 4\n_pdbe_chem_comp_rdkit_properties.NumHeavyAtoms 5\n_pdbe_chem_comp_rdkit_properties.NumAtoms 5\n_pdbe_chem_comp_rdkit_properties.NumHeteroatoms 5\n_pdbe_chem_comp_rdkit_properties.NumAmideBonds 0\n_pdbe_chem_comp_rdkit_properties.FractionCSP3 0\n_pdbe_chem_comp_rdkit_properties.NumRings 0\n_pdbe_chem_comp_rdkit_properties.NumAromaticRings 0\n_pdbe_chem_comp_rdkit_properties.NumAliphaticRings 0\n_pdbe_chem_comp_rdkit_properties.NumSaturatedRings

0\n_pdbe_chem_comp_rdkit_properties.NumHeterocycles
0\n_pdbe_chem_comp_rdkit_properties.NumAromaticHeterocycles
0\n_pdbe_chem_comp_rdkit_properties.NumSaturatedHeterocycles
0\n_pdbe_chem_comp_rdkit_properties.NumAliphaticHeterocycles
0\n_pdbe_chem_comp_rdkit_properties.NumSpiroAtoms
0\n_pdbe_chem_comp_rdkit_properties.NumBridgeheadAtoms
0\n_pdbe_chem_comp_rdkit_properties.NumAtomStereoCenters
0\n_pdbe_chem_comp_rdkit_properties.NumUnspecifiedAtomStereoCenters
0\n_pdbe_chem_comp_rdkit_properties.labuteASA
28.307\n_pdbe_chem_comp_rdkit_properties.tpsa
86.250\n_pdbe_chem_comp_rdkit_properties.CrippenClogP -
2.825\n_pdbe_chem_comp_rdkit_properties.CrippenMR
7.606\n_pdbe_chem_comp_rdkit_properties.chi0v
2.975\n_pdbe_chem_comp_rdkit_properties.chi1v
2.191\n_pdbe_chem_comp_rdkit_properties.chi2v
0\n_pdbe_chem_comp_rdkit_properties.chi3v
0\n_pdbe_chem_comp_rdkit_properties.chi4v
0\n_pdbe_chem_comp_rdkit_properties.chi0n
2.080\n_pdbe_chem_comp_rdkit_properties.chi1n
0.730\n_pdbe_chem_comp_rdkit_properties.chi2n
0\n_pdbe_chem_comp_rdkit_properties.chi3n
0\n_pdbe_chem_comp_rdkit_properties.chi4n
0\n_pdbe_chem_comp_rdkit_properties.hallKierAlpha
0.110\n_pdbe_chem_comp_rdkit_properties.kappa1
5.110\n_pdbe_chem_comp_rdkit_properties.kappa2
1.065\n_pdbe_chem_comp_rdkit_properties.kappa3
1512.242\n_pdbe_chem_comp_rdkit_properties.Phi
1.088\n#\nloop_\n_pdbe_chem_comp_external_mappings.comp_id\n_pdbe_chem
_comp_external_mappings.source\n_pdbe_chem_comp_external_mappings.reso
urce\n_pdbe_chem_comp_external_mappings.resource_id\nPO4 UniChem
DrugBank DB14523\nPO4 UniChem ChEBI 18367\nPO4 UniChem eMolecules
882178\nPO4 UniChem fdasrs NK08V8K8HR\nPO4 UniChem NMRShiftDB
30000818\nPO4 UniChem BindingDb 50155537\nPO4 UniChem MetaboLights
MTBLC18367\nPO4 UniChem BRENDA 21671\nPO4 UniChem BRENDA 867\nPO4
UniChem ChemicalBook CB3409949\nPO4 UniChem ChemicalBook
CB7191961\nPO4 UniChem rxnorm 'PHOSPHATE ION'\nPO4 UniChem 'Probes And
Drugs' PD094531\nPO4 UniChem CCDC EXIZIX\nPO4 UniChem 'EPA CompTox
Dashboard' DTXSID7039672\nPO4 UniChem 'PubChem TPHARMA' 15218698\nPO4
UniChem PubChem 1061\nPO4 UniChem ACTor 14265-44-2\nPO4 UniChem
Nikkaji J215.970H\nPO4 UniChem Nikkaji
J351.173A\n#\nloop_\n_pdbe_chem_comp_rdkit_conformer.comp_id\n_pdbe_ch
em_comp_rdkit_conformer.atom_id\n_pdbe_chem_comp_rdkit_conformer.Cartn
_x_rdkit\n_pdbe_chem_comp_rdkit_conformer.Cartn_y_rdkit\n_pdbe_chem_co
mp_rdkit_conformer.Cartn_z_rdkit\n_pdbe_chem_comp_rdkit_conformer.rdki
t_method\n_pdbe_chem_comp_rdkit_conformer.rdkit_ordinal\nPO4 P -0.003
0.002 0.036 ETKDGv3 1\nPO4 O1 -0.117 0.079 1.541 ETKDGv3 2\nPO4 O3 -
1.075 -1.165 -0.552 ETKDGv3 4\nPO4 O4 -0.383 1.506 -0.636 ETKDGv3
5\n#\n"
}

Fig. 5i. Hsα-DG carrying core M3 *O*-Man glycan

{
    "name": "mg_7",
    "modelSeeds": [
      1, 2, 3, 4, 5, 6, 7, 8, 9, 10
    ],
    "sequences": [
      {
        "protein": {
          "sequence":
"RQIHATPTPVTAIGPPTTAIQEPPSRIVPTPTSPAIAPPTETMAPPVRDPVPGKPTVTIRTRGAIIQTP
TLGPIQPTRVSEAGTTVPGQIRPTMTIPGYVEPTAVATPPTTTTKKPRVSTPKPATPSTDSTTTTTRRPT
KKPRTPRPVPRVTTKVSITRLETASPPTRIRTTTSGVPRGGEPNQRPELKNHIDRVDAWVGTYFEVKIPS
DTFYDHEDTTTDKLKLTLKLREQQLVGEKSWVQFNSNSQLMYGLPDSSHVGKHEYFMHATDKGGLSAVDA
FEIHVHRRPQGDRAPARFKAKFVGDPALVLNDIHKKIALVKKLAFAFGDRNCSTITLQNIT",
          "id": "ADG"
        }
      },
      {
        "ligand": {
          "ccdCodes": [
            "M6P", "NAG", "NGA", "A1H0Z-O3P", "A1H0Z-O3P", "XYP",
"BDP"
          ],
          "id": "MGA"
        }
      }
        ],
    "dialect": "alphafold3",
    "version": 2,
    "bondedAtomPairs": [
    [["ADG",6,"OG1"],["MGA",1,"C1"]],
    [["MGA",1,"O4"],["MGA",2,"C1"]],
    [["MGA",2,"O3"],["MGA",3,"C1"]],
    [["MGA",3,"O3"],["MGA",4,"P"]],
    [["MGA",4,"O1"],["MGA",5,"P"]],
    [["MGA",5,"O4"],["MGA",6,"C1"]],
    [["MGA",6,"O4"],["MGA",7,"C1"]]
    ],
    "userCCD": "data_A1H0Z-O3P\n#\n_chem_comp.id
A1H0Z\n_chem_comp.name D-Ribitol-5-phosphate\n_chem_comp.type D-
saccharide\n_chem_comp.pdbx_type ATOMS\n_chem_comp.formula 'C5 H13 O8
P'\n_chem_comp.mon_nstd_parent_comp_id ?\n_chem_comp.pdbx_synonyms
'[(2R,3S,4S)-2,3,4,5-tetrakis(oxidanyl)pentyl] dihydrogen
phosphate'\n_chem_comp.pdbx_formal_charge
0\n_chem_comp.pdbx_initial_date 2023-12-
20\n_chem_comp.pdbx_modified_date 2024-05-
10\n_chem_comp.pdbx_ambiguous_flag N\n_chem_comp.pdbx_release_status
REL\n_chem_comp.pdbx_replaced_by ?\n_chem_comp.pdbx_replaces ?\n_chem_
comp.formula_weight
232.126\n_chem_comp.one_letter_code ?\n_chem_comp.three_letter_code
A1H0Z\n_chem_comp.pdbx_model_coordinates_details ?\n_chem_comp.pdbx_mo

del_coordinates_missing_flag
Y\n_chem_comp.pdbx_ideal_coordinates_details
Corina\n_chem_comp.pdbx_ideal_coordinates_missing_flag
N\n_chem_comp.pdbx_model_coordinates_db_code
8RDF\n_chem_comp.pdbx_subcomponent_list ?\n_chem_comp.pdbx_processing_
site
PDBE\n#\nloop_\n_chem_comp_atom.comp_id\n_chem_comp_atom.atom_id\n_che
m_comp_atom.alt_atom_id\n_chem_comp_atom.type_symbol\n_chem_comp_atom.
charge\n_chem_comp_atom.pdbx_align\n_chem_comp_atom.pdbx_aromatic_flag
\n_chem_comp_atom.pdbx_leaving_atom_flag\n_chem_comp_atom.pdbx_stereo_
config\n_chem_comp_atom.pdbx_n_terminal_atom_flag\n_chem_comp_atom.pdb
x_backbone_atom_flag\n_chem_comp_atom.pdbx_c_terminal_atom_flag\n_chem
_comp_atom.model_Cartn_x\n_chem_comp_atom.model_Cartn_y\n_chem_comp_at
om.model_Cartn_z\n_chem_comp_atom.pdbx_model_Cartn_x_ideal\n_chem_comp
_atom.pdbx_model_Cartn_y_ideal\n_chem_comp_atom.pdbx_model_Cartn_z_ide
al\n_chem_comp_atom.pdbx_component_atom_id\n_chem_comp_atom.pdbx_compo
nent_comp_id\n_chem_comp_atom.pdbx_ordinal\nA1H0Z O1 O1 O 0 1 N Y N N
N N -15.832 9.785 -36.304 -5.556 0.179 -0.308 O1 A1H0Z 1\nA1H0Z C1 C1
C 0 1 N N N N N N -16.593 9.662 -37.519 -4.354 0.626 0.324 C1 A1H0Z
2\nA1H0Z O2 O2 O 0 1 N N N N N N -16.641 9.014 -39.811 -3.251 -1.505
0.030 O2 A1H0Z 3\nA1H0Z C3 C2 C 0 1 N N S N N N -15.395 7.597 -38.321
-1.862 0.452 0.308 C3 A1H0Z 4\nA1H0Z O3 O3 O 0 1 N N N N N N -16.066
7.187 -37.125 -1.767 1.844 -0.001 O3 A1H0Z 5\nA1H0Z C4 C3 C 0 1 N N R
N N N -13.883 7.367 -38.194 -0.664 -0.286 -0.294 C4 A1H0Z 6\nA1H0Z O4
O4 O 0 1 N N N N N N -13.150 8.451 -38.775 -0.759 -1.678 0.014 O4
A1H0Z 7\nA1H0Z C5 C4 C 0 1 N N N N N N -13.429 6.050 -38.790 0.631
0.279 0.292 C5 A1H0Z 8\nA1H0Z C2 C5 C 0 1 N N S N N N -15.808 9.031 -
38.649 -3.157 -0.113 -0.278 C2 A1H0Z 9\nA1H0Z O5 O5 O 0 1 N N N N N N
-14.265 4.956 -38.315 1.751 -0.328 -0.354 O5 A1H0Z 10\nA1H0Z P P1 P 0
1 N N N N N N -14.039 3.490 -38.807 3.277 0.031 0.014 P A1H0Z
11\nA1H0Z O2P O6 O 0 1 N N N N N N -12.569 3.135 -38.464 3.574 1.566 -
0.370 O2P A1H0Z 12\nA1H0Z O1P O7 O 0 1 N N N N N N -14.982 2.499 -
38.205 3.494 -0.166 1.464 O1P A1H0Z 13\nA1H0Z HO1 H1 H 0 1 N Y N N N N
-16.371 10.185 -35.632 -6.357 0.606 0.025 HO1 A1H0Z 14\nA1H0Z H12 H2 H
0 1 N N N N N N -16.915 10.666 -37.834 -4.237 1.698 0.165 H12 A1H0Z
15\nA1H0Z H11 H3 H 0 1 N N N N N N -17.477 9.040 -37.317 -4.407 0.420
1.393 H11 A1H0Z 16\nA1H0Z HO2 H4 H 0 1 N N N N N N -16.905 9.901 -
40.025 -3.259 -1.698 0.977 HO2 A1H0Z 17\nA1H0Z HC3 H5 H 0 1 N N N N N
N -15.748 6.963 -39.148 -1.864 0.319 1.390 HC3 A1H0Z 18\nA1H0Z HO3 H6
H 0 1 N N N N N N -15.816 6.296 -36.911 -1.759 2.038 -0.948 HO3 A1H0Z
19\nA1H0Z HC4 H7 H 0 1 N N N N N N -13.653 7.334 -37.119 -0.662 -0.153
-1.376 HC4 A1H0Z 20\nA1H0Z HO4 H8 H 0 1 N N N N N N -13.442 9.271 -
38.395 -0.766 -1.872 0.961 HO4 A1H0Z 21\nA1H0Z H51 H9 H 0 1 N N N N N
N -12.386 5.861 -38.497 0.661 1.357 0.134 H51 A1H0Z 22\nA1H0Z H52 H10
H 0 1 N N N N N N -13.497 6.107 -39.886 0.669 0.067 1.360 H52 A1H0Z
23\nA1H0Z HC2 H11 H 0 1 N N N N N N -14.904 9.629 -38.837 -3.154 0.020
-1.360 HC2 A1H0Z 24\nA1H0Z HOP2 H12 H 0 0 N N N N N N -12.549 2.348 -
37.932 3.452 1.768 -1.308 HOP2 A1H0Z
25\n#\nloop_\n_chem_comp_bond.comp_id\n_chem_comp_bond.atom_id_1\n_che
m_comp_bond.atom_id_2\n_chem_comp_bond.value_order\n_chem_comp_bond.pd
bx_aromatic_flag\n_chem_comp_bond.pdbx_stereo_config\n_chem_comp_bond.
pdbx_ordinal\nA1H0Z O2 C2 SING N N 1\nA1H0Z P O2P SING N N 2\nA1H0Z P

O5 SING N N 3\nA1H0Z P O1P DOUB N N 4\nA1H0Z C5 O5 SING N N 5\nA1H0Z C5 C4 SING N N 6\nA1H0Z O4 C4 SING N N 7\nA1H0Z C2 C3 SING N N 8\nA1H0Z C2 C1 SING N N 9\nA1H0Z C3 C4 SING N N 10\nA1H0Z C3 O3 SING N N 11\nA1H0Z C1 O1 SING N N 12\nA1H0Z O1 HO1 SING N N 13\nA1H0Z C1 H12 SING N N 14\nA1H0Z C1 H11 SING N N 15\nA1H0Z O2 HO2 SING N N 16\nA1H0Z C3 HC3 SING N N 17\nA1H0Z O3 HO3 SING N N 18\nA1H0Z C4 HC4 SING N N 19\nA1H0Z O4 HO4 SING N N 20\nA1H0Z C5 H51 SING N N 21\nA1H0Z C5 H52 SING N N 22\nA1H0Z C2 HC2 SING N N 23\nA1H0Z O2P HOP2 SING N N 24\n#\nloop_\n_pdbx_chem_comp_descriptor.comp_id\n_pdbx_chem_comp_descriptor.type\n_pdbx_chem_comp_descriptor.program\n_pdbx_chem_comp_descriptor.program_version\n_pdbx_chem_comp_descriptor.descriptor\nA1H0Z InChI InChI 1.06 'InChI=1S/C5H13O8P/c6-1-3(7)5(9)4(8)2-13-14(10,11)12/h3-9H,1-2H2,(H2,10,11,12)/t3-,4+,5-/m0/s1'\nA1H0Z InChIKey InChI 1.06 VJDOAZKNBQCAGE-LMVFSUKVSA-N\nA1H0Z SMILES_CANONICAL CACTVS 3.385 'OC[C@H](O)[C@H](O)[C@H](O)CO[P](O)(O)=O'\nA1H0Z SMILES CACTVS 3.385 'OC[CH](O)[CH](O)[CH](O)CO[P](O)(O)=O'\nA1H0Z SMILES_CANONICAL 'OpenEye OEToolkits' 2.0.7 'C([C@@H]([C@@H]([C@@H](COP(=O)(O)O)O)O)O)O'\nA1H0Z SMILES 'OpenEye OEToolkits' 2.0.7 'C(C(C(C(COP(=O)(O)O)O)O)O)O'\n#\n_pdbx_chem_comp_identifier.comp_id A1H0Z\n_pdbx_chem_comp_identifier.type 'SYSTEMATIC NAME'\n_pdbx_chem_comp_identifier.program 'OpenEye OEToolkits'\n_pdbx_chem_comp_identifier.program_version 2.0.7\n_pdbx_chem_comp_identifier.identifier '[(2~{R},3~{S},4~{S})-2,3,4,5-tetrakis(oxidanyl)pentyl] dihydrogen phosphate'\n#\n_pdbx_chem_comp_synonyms.ordinal 1\n_pdbx_chem_comp_synonyms.comp_id A1H0Z\n_pdbx_chem_comp_synonyms.name '[(2R,3S,4S)-2,3,4,5-tetrakis(oxidanyl)pentyl] dihydrogen phosphate'\n_pdbx_chem_comp_synonyms.provenance PDB\n_pdbx_chem_comp_synonyms.type ?\n#\nloop_\n_pdbx_chem_comp_audit.comp_id\n_pdbx_chem_comp_audit.action_type\n_pdbx_chem_comp_audit.date\n_pdbx_chem_comp_audit.processing_site\nA1H0Z 'Create component' 2023-12-20 PDBE\nA1H0Z 'Initial release' 2024-05-15 RCSB\n#\n_pdbe_chem_comp_synonyms.comp_id A1H0Z\n_pdbe_chem_comp_synonyms.name '[(2R,3S,4S)-2,3,4,5-tetrakis(oxidanyl)pentyl] dihydrogen phosphate'\n_pdbe_chem_comp_synonyms.provenance wwPDB\n_pdbe_chem_comp_synonyms.type ?\n#\nloop_\n_software.name\n_software.version\n_software.description\nrdkit 2023.09.6 'Core functionality.'\npdbeccdutils 0.8.6 'Wrapper to provide 2D templates and molecular fragments.'\n#\nloop_\n_pdbe_chem_comp_atom_depiction.comp_id\n_pdbe_chem_comp_atom_depiction.atom_id\n_pdbe_chem_comp_atom_depiction.element\n_pdbe_chem_comp_atom_depiction.model_Cartn_x\n_pdbe_chem_comp_atom_depiction.model_Cartn_y\n_pdbe_chem_comp_atom_depiction.pdbx_ordinal\nA1H0Z O1 O 3.805 0.375 1\nA1H0Z C1 C 5.104 -0.375 2\nA1H0Z O2 O 6.404 1.875 3\nA1H0Z C3 C 7.702 -0.375 4\nA1H0Z O3 O 7.702 -1.875 5\nA1H0Z C4 C 9.002 0.375 6\nA1H0Z O4 O 9.002 1.875 7\nA1H0Z C5 C 10.301 -0.375 8\nA1H0Z C2 C 6.404 0.375 9\nA1H0Z O5 O 11.600 0.375 10\nA1H0Z P P 12.899 -0.375 11\nA1H0Z O2P O 14.198 -1.125 12\nA1H0Z O1P O 13.649 0.924

13\n#\nloop_\n_pdbe_chem_comp_bond_depiction.comp_id\n_pdbe_chem_comp_bond_depiction.atom_id_1\n_pdbe_chem_comp_bond_depiction.atom_id_2\n_pdbe_chem_comp_bond_depiction.value_order\n_pdbe_chem_comp_bond_depiction.bond_dir\n_pdbe_chem_comp_bond_depiction.pdbx_ordinal\nA1H0Z C2 O2 SINGLE BEGINDASH 1\nA1H0Z P O2P SINGLE NONE 2\nA1H0Z P O5 SINGLE NONE 3\nA1H0Z P O1P DOUBLE NONE 4\nA1H0Z C5 O5 SINGLE NONE 5\nA1H0Z C5 C4 SINGLE NONE 6\nA1H0Z C4 O4 SINGLE BEGINDASH 7\nA1H0Z C2 C3 SINGLE NONE 8\nA1H0Z C2 C1 SINGLE NONE 9\nA1H0Z C3 C4 SINGLE NONE 10\nA1H0Z C3 O3 SINGLE BEGINWEDGE 11\nA1H0Z C1 O1 SINGLE NONE 12\n#\n_pdbe_chem_comp_substructure.comp_id A1H0Z\n_pdbe_chem_comp_substructure.substructure_name phosphate\n_pdbe_chem_comp_substructure.id F1\n_pdbe_chem_comp_substructure.substructure_type fragment\n_pdbe_chem_comp_substructure.substructure_smiles O=P(O)(O)O\n_pdbe_chem_comp_substructure.substructure_inchis InChI=1S/H3O4P/c1-5(2,3)4/h(H3,1,2,3,4)\n_pdbe_chem_comp_substructure.substructure_inchikeys NBIIXXVUZAFLBC-UHFFFAOYSA-N\n#\nloop_\n_pdbe_chem_comp_substructure_mapping.comp_id\n_pdbe_chem_comp_substructure_mapping.atom_id\n_pdbe_chem_comp_substructure_mapping.substructure_id\n_pdbe_chem_comp_substructure_mapping.substructure_ordinal\nA1H0Z O5 F1 1\nA1H0Z P F1 1\nA1H0Z O1P F1 1\nA1H0Z O2P F1 1\nA1H0Z HO1 F1 1\n#\n_pdbe_chem_comp_rdkit_properties.comp_id A1H0Z\n_pdbe_chem_comp_rdkit_properties.exactmw 232.035\n_pdbe_chem_comp_rdkit_properties.amw 232.125\n_pdbe_chem_comp_rdkit_properties.lipinskiHBA 8\n_pdbe_chem_comp_rdkit_properties.lipinskiHBD 6\n_pdbe_chem_comp_rdkit_properties.NumRotatableBonds 12\n_pdbe_chem_comp_rdkit_properties.NumHBD 6\n_pdbe_chem_comp_rdkit_properties.NumHBA 8\n_pdbe_chem_comp_rdkit_properties.NumHeavyAtoms 14\n_pdbe_chem_comp_rdkit_properties.NumAtoms 27\n_pdbe_chem_comp_rdkit_properties.NumHeteroatoms 9\n_pdbe_chem_comp_rdkit_properties.NumAmideBonds 0\n_pdbe_chem_comp_rdkit_properties.FractionCSP3 1\n_pdbe_chem_comp_rdkit_properties.NumRings 0\n_pdbe_chem_comp_rdkit_properties.NumAromaticRings 0\n_pdbe_chem_comp_rdkit_properties.NumAliphaticRings 0\n_pdbe_chem_comp_rdkit_properties.NumSaturatedRings 0\n_pdbe_chem_comp_rdkit_properties.NumHeterocycles 0\n_pdbe_chem_comp_rdkit_properties.NumAromaticHeterocycles 0\n_pdbe_chem_comp_rdkit_properties.NumSaturatedHeterocycles 0\n_pdbe_chem_comp_rdkit_properties.NumAliphaticHeterocycles 0\n_pdbe_chem_comp_rdkit_properties.NumSpiroAtoms 0\n_pdbe_chem_comp_rdkit_properties.NumBridgeheadAtoms 0\n_pdbe_chem_comp_rdkit_properties.NumAtomStereoCenters 3\n_pdbe_chem_comp_rdkit_properties.NumUnspecifiedAtomStereoCenters 0\n_pdbe_chem_comp_rdkit_properties.labuteASA 98.412\n_pdbe_chem_comp_rdkit_properties.tpsa 147.680\n_pdbe_chem_comp_rdkit_properties.CrippenClogP -2.829\n_pdbe_chem_comp_rdkit_properties.CrippenMR 43.102\n_pdbe_chem_comp_rdkit_properties.chi0v

7.108\n_pdbe_chem_comp_rdkit_properties.chi1v
4.212\n_pdbe_chem_comp_rdkit_properties.chi2v
1.070\n_pdbe_chem_comp_rdkit_properties.chi3v
1.070\n_pdbe_chem_comp_rdkit_properties.chi4v
0.488\n_pdbe_chem_comp_rdkit_properties.chi0n
19.213\n_pdbe_chem_comp_rdkit_properties.chi1n
8.700\n_pdbe_chem_comp_rdkit_properties.chi2n
0.755\n_pdbe_chem_comp_rdkit_properties.chi3n
0.755\n_pdbe_chem_comp_rdkit_properties.chi4n
0.293\n_pdbe_chem_comp_rdkit_properties.hallKierAlpha -
0.050\n_pdbe_chem_comp_rdkit_properties.kappa1
3.474\n_pdbe_chem_comp_rdkit_properties.kappa2
5.740\n_pdbe_chem_comp_rdkit_properties.kappa3
5.632\n_pdbe_chem_comp_rdkit_properties.Phi
1.424\n#\nloop_\n_pdbe_chem_comp_external_mappings.comp_id\n_pdbe_chem_comp_external_mappings.source\n_pdbe_chem_comp_external_mappings.resource\n_pdbe_chem_comp_external_mappings.resource_id\nA1H0Z UniChem 'KEGG LIGAND' C01068\nA1H0Z UniChem ChEBI 16246\nA1H0Z UniChem ZINC ZINC000001529544\nA1H0Z UniChem fdasrs 0BA496N6TQ\nA1H0Z UniChem SureChEMBL SCHEMBL638724\nA1H0Z UniChem 'PubChem TPHARMA' 14773839\nA1H0Z UniChem ACTor 3506-18-1\nA1H0Z UniChem Nikkaji J1.612.794I\nA1H0Z UniChem 'EPA CompTox Dashboard' DTXSID30188562\nA1H0Z UniChem BRENDA 234532\nA1H0Z UniChem BRENDA 3458\nA1H0Z UniChem BRENDA 48290\nA1H0Z UniChem BRENDA 94916\nA1H0Z UniChem PubChem
151104\n#\nloop_\n_pdbe_chem_comp_rdkit_conformer.comp_id\n_pdbe_chem_comp_rdkit_conformer.atom_id\n_pdbe_chem_comp_rdkit_conformer.Cartn_x_rdkit\n_pdbe_chem_comp_rdkit_conformer.Cartn_y_rdkit\n_pdbe_chem_comp_rdkit_conformer.Cartn_z_rdkit\n_pdbe_chem_comp_rdkit_conformer.rdkit_method\n_pdbe_chem_comp_rdkit_conformer.rdkit_ordinal\nA1H0Z O1 -2.730 1.255 -1.432 ETKDGv3 1\nA1H0Z C1 -3.139 0.438 -0.370 ETKDGv3 2\nA1H0Z O2 -2.782 -1.741 0.657 ETKDGv3 3\nA1H0Z C3 -0.819 -0.765 -0.427 ETKDGv3 4\nA1H0Z O3 -0.288 -2.053 -0.630 ETKDGv3 5\nA1H0Z C4 -0.198 -0.140 0.853 ETKDGv3 6\nA1H0Z O4 -0.589 1.203 0.967 ETKDGv3 7\nA1H0Z C5 1.340 -0.206 0.838 ETKDGv3 8\nA1H0Z C2 -2.367 -0.897 -0.390 ETKDGv3 9\nA1H0Z O5 1.837 0.486 -0.285 ETKDGv3 10\nA1H0Z P 3.535 0.328 -0.310 ETKDGv3 11\nA1H0Z O2P 4.198 0.867 1.151 ETKDGv3 12\nA1H0Z O1P 3.917 -1.119 -0.532 ETKDGv3 13\nA1H0Z HO1 -3.257 2.093 -1.354 ETKDGv3 14\nA1H0Z H12 -4.225 0.219 -0.475 ETKDGv3 15\nA1H0Z H11 -2.998 0.965 0.598 ETKDGv3 16\nA1H0Z HO2 -2.764 -1.218 1.500 ETKDGv3 17\nA1H0Z HC3 -0.560 -0.113 -1.295 ETKDGv3 18\nA1H0Z HO3 0.473 -1.967 -1.262 ETKDGv3 19\nA1H0Z HC4 -0.546 -0.716 1.741 ETKDGv3 20\nA1H0Z HO4 -0.484 1.457 1.921 ETKDGv3 21\nA1H0Z H51 1.656 -1.274 0.831 ETKDGv3 22\nA1H0Z H52 1.713 0.262 1.777 ETKDGv3 23\nA1H0Z HC2 -2.659 -1.401 -1.339 ETKDGv3 24\nA1H0Z HOP2 3.916 1.814 1.221 ETKDGv3 25\n#\n"
}

Fig. 6c and 6e. Cis-interaction of two glycosylated HsSiglec-2


```
{
    "name": "siglec2_g2s2_66_dimer",
    "modelSeeds": [
      1
    ],
    "sequences": [
      {
        "protein": {
          "sequence":
"MHLLGPWLLLLVLEYLAFSDSSKWVFEHPETLYAWEGACVWIPCTYRALDGDLESFILFHNPEYNKNTS
KFDGTRLYESTKDGKVPSEQKRVQFLGDKNKNCTLSIHPVHLNDSGQLGLRMESKTEKWMERIHLNVSER
PFPPHIQLPPEIQESQEVTLTCLLNFSCYGYPIQLQWLLEGVPMRQAAVTSTSLTIKSVFTRSELKFSPQ
WSHHGKIVTCQLQDADGKFLSNDTVQLNVKHTPKLEIKVTPSDAIVREGDSVTMTCEVSSSNPEYTTVSW
LKDGTSLKKQNTFTLNLREVTKDQSGKYCCQVSNDVGPGRSEEVFLQVQYAPEPSTVQILHSPAVEGSQV
EFLCMSLANPLPTNYTWYHNGKEMQGRTEEKVHIPKILPWHAGTYSCVAENILGTGQRGPGAELDVQYPP
KKVTTVIQNPMPIREGDTVTLSCNYNSSNPSVTRYEWKPHGAWEEPSLGVLKIQNVGWDNTTIACAACNS
WCSWASPVALNVQYAPRDVRVRKIKPLSEIHSGNSVSLQCDFSSSHPKEVQFFWEKNGRLLGKESQLNFD
SISPEDAGSYSCWVNNSIGQTASKAWTLEVLYAPRRLRVSMSPGDQVMEGKSATLTCESDANPPVSHYTW
FDWNNQSLPYHSQKLRLEPVKVQHSGAYWCQGTNSVGKGRSPLSTLTVYYSPETIGRRVAVGLGSCLAIL
ILAICGLKLQRRWKRTQSQQGLQENSSGQSFFVRNKKVRRAPLSEGPHSLGCYNPMMEDGISYTTLRFPE
MNIPRTGDAESSEMQRPPPDCDDTVTYSALHKRQVGDYENVIPDFPEDEGIHYSELIQFGVGERPQAQEN
VDYVILKH",
          "id": ["SIGLEC", "SIG"]
        }
      },
      {
        "ligand": {
          "ccdCodes": [
            "NAG", "NAG", "BMA", "MAN", "MAN", "NAG", "NAG", "GAL",
"GAL", "SIA2", "SIA2"
          ],
          "id": ["NGA", "NGB", "NGC", "NGD", "NGE", "NGF", "NGG",
"NGH", "NGI", "NGJ", "NGK", "NA", "NB", "NC", "ND", "NE", "NF", "NG",
"NH", "NI", "NJ", "NK"]
        }
      }
    ],
    "dialect": "alphafold3",
    "version": 2,
    "bondedAtomPairs": [
    [["SIGLEC",67,"ND2"],["NGA",1,"C1"]],
    [["NGA",1,"O4"],["NGA",2,"C1"]],
    [["NGA",2,"O4"],["NGA",3,"C1"]],
    [["NGA",3,"O3"],["NGA",4,"C1"]],
    [["NGA",3,"O6"],["NGA",5,"C1"]],
    [["NGA",4,"O2"],["NGA",6,"C1"]],
    [["NGA",5,"O2"],["NGA",7,"C1"]],
    [["NGA",6,"O4"],["NGA",8,"C1"]],
    [["NGA",7,"O4"],["NGA",9,"C1"]],
    [["NGA",8,"O6"],["NGA",10,"C2"]],
    [["NGA",9,"O6"],["NGA",11,"C2"]],
```

[["SIGLEC",101,"ND2"],["NGB",1,"C1"]],
[["NGB",1,"O4"],["NGB",2,"C1"]],
[["NGB",2,"O4"],["NGB",3,"C1"]],
[["NGB",3,"O3"],["NGB",4,"C1"]],
[["NGB",3,"O6"],["NGB",5,"C1"]],
[["NGB",4,"O2"],["NGB",6,"C1"]],
[["NGB",5,"O2"],["NGB",7,"C1"]],
[["NGB",6,"O4"],["NGB",8,"C1"]],
[["NGB",7,"O4"],["NGB",9,"C1"]],
[["NGB",8,"O6"],["NGB",10,"C2"]],
[["NGB",9,"O6"],["NGB",11,"C2"]],
[["SIGLEC",112,"ND2"],["NGC",1,"C1"]],
[["NGC",1,"O4"],["NGC",2,"C1"]],
[["NGC",2,"O4"],["NGC",3,"C1"]],
[["NGC",3,"O3"],["NGC",4,"C1"]],
[["NGC",3,"O6"],["NGC",5,"C1"]],
[["NGC",4,"O2"],["NGC",6,"C1"]],
[["NGC",5,"O2"],["NGC",7,"C1"]],
[["NGC",6,"O4"],["NGC",8,"C1"]],
[["NGC",7,"O4"],["NGC",9,"C1"]],
[["NGC",8,"O6"],["NGC",10,"C2"]],
[["NGC",9,"O6"],["NGC",11,"C2"]],
[["SIGLEC",135,"ND2"],["NGD",1,"C1"]],
[["NGD",1,"O4"],["NGD",2,"C1"]],
[["NGD",2,"O4"],["NGD",3,"C1"]],
[["NGD",3,"O3"],["NGD",4,"C1"]],
[["NGD",3,"O6"],["NGD",5,"C1"]],
[["NGD",4,"O2"],["NGD",6,"C1"]],
[["NGD",5,"O2"],["NGD",7,"C1"]],
[["NGD",6,"O4"],["NGD",8,"C1"]],
[["NGD",7,"O4"],["NGD",9,"C1"]],
[["NGD",8,"O6"],["NGD",10,"C2"]],
[["NGD",9,"O6"],["NGD",11,"C2"]],
[["SIGLEC",164,"ND2"],["NGE",1,"C1"]],
[["NGE",1,"O4"],["NGE",2,"C1"]],
[["NGE",2,"O4"],["NGE",3,"C1"]],
[["NGE",3,"O3"],["NGE",4,"C1"]],
[["NGE",3,"O6"],["NGE",5,"C1"]],
[["NGE",4,"O2"],["NGE",6,"C1"]],
[["NGE",5,"O2"],["NGE",7,"C1"]],
[["NGE",6,"O4"],["NGE",8,"C1"]],
[["NGE",7,"O4"],["NGE",9,"C1"]],
[["NGE",8,"O6"],["NGE",10,"C2"]],
[["NGE",9,"O6"],["NGE",11,"C2"]],
[["SIGLEC",231,"ND2"],["NGF",1,"C1"]],
[["NGF",1,"O4"],["NGF",2,"C1"]],
[["NGF",2,"O4"],["NGF",3,"C1"]],
[["NGF",3,"O3"],["NGF",4,"C1"]],
[["NGF",3,"O6"],["NGF",5,"C1"]],
[["NGF",4,"O2"],["NGF",6,"C1"]],
[["NGF",5,"O2"],["NGF",7,"C1"]],
[["NGF",6,"O4"],["NGF",8,"C1"]],

[["NGF",7,"O4"],["NGF",9,"C1"]],
[["NGF",8,"O6"],["NGF",10,"C2"]],
[["NGF",9,"O6"],["NGF",11,"C2"]],
[["SIGLEC",363,"ND2"],["NGG",1,"C1"]],
[["NGG",1,"O4"],["NGG",2,"C1"]],
[["NGG",2,"O4"],["NGG",3,"C1"]],
[["NGG",3,"O3"],["NGG",4,"C1"]],
[["NGG",3,"O6"],["NGG",5,"C1"]],
[["NGG",4,"O2"],["NGG",6,"C1"]],
[["NGG",5,"O2"],["NGG",7,"C1"]],
[["NGG",6,"O4"],["NGG",8,"C1"]],
[["NGG",7,"O4"],["NGG",9,"C1"]],
[["NGG",8,"O6"],["NGG",10,"C2"]],
[["NGG",9,"O6"],["NGG",11,"C2"]],
[["SIGLEC",445,"ND2"],["NGH",1,"C1"]],
[["NGH",1,"O4"],["NGH",2,"C1"]],
[["NGH",2,"O4"],["NGH",3,"C1"]],
[["NGH",3,"O3"],["NGH",4,"C1"]],
[["NGH",3,"O6"],["NGH",5,"C1"]],
[["NGH",4,"O2"],["NGH",6,"C1"]],
[["NGH",5,"O2"],["NGH",7,"C1"]],
[["NGH",6,"O4"],["NGH",8,"C1"]],
[["NGH",7,"O4"],["NGH",9,"C1"]],
[["NGH",8,"O6"],["NGH",10,"C2"]],
[["NGH",9,"O6"],["NGH",11,"C2"]],
[["SIGLEC",479,"ND2"],["NGI",1,"C1"]],
[["NGI",1,"O4"],["NGI",2,"C1"]],
[["NGI",2,"O4"],["NGI",3,"C1"]],
[["NGI",3,"O3"],["NGI",4,"C1"]],
[["NGI",3,"O6"],["NGI",5,"C1"]],
[["NGI",4,"O2"],["NGI",6,"C1"]],
[["NGI",5,"O2"],["NGI",7,"C1"]],
[["NGI",6,"O4"],["NGI",8,"C1"]],
[["NGI",7,"O4"],["NGI",9,"C1"]],
[["NGI",8,"O6"],["NGI",10,"C2"]],
[["NGI",9,"O6"],["NGI",11,"C2"]],
[["SIGLEC",574,"ND2"],["NGJ",1,"C1"]],
[["NGJ",1,"O4"],["NGJ",2,"C1"]],
[["NGJ",2,"O4"],["NGJ",3,"C1"]],
[["NGJ",3,"O3"],["NGJ",4,"C1"]],
[["NGJ",3,"O6"],["NGJ",5,"C1"]],
[["NGJ",4,"O2"],["NGJ",6,"C1"]],
[["NGJ",5,"O2"],["NGJ",7,"C1"]],
[["NGJ",6,"O4"],["NGJ",8,"C1"]],
[["NGJ",7,"O4"],["NGJ",9,"C1"]],
[["NGJ",8,"O6"],["NGJ",10,"C2"]],
[["NGJ",9,"O6"],["NGJ",11,"C2"]],
[["SIGLEC",634,"ND2"],["NGK",1,"C1"]],
[["NGK",1,"O4"],["NGK",2,"C1"]],
[["NGK",2,"O4"],["NGK",3,"C1"]],
[["NGK",3,"O3"],["NGK",4,"C1"]],
[["NGK",3,"O6"],["NGK",5,"C1"]],

[["NGK",4,"O2"],["NGK",6,"C1"]],
[["NGK",5,"O2"],["NGK",7,"C1"]],
[["NGK",6,"O4"],["NGK",8,"C1"]],
[["NGK",7,"O4"],["NGK",9,"C1"]],
[["NGK",8,"O6"],["NGK",10,"C2"]],
[["NGK",9,"O6"],["NGK",11,"C2"]],
[["SIG",67,"ND2"],["NA",1,"C1"]],
[["NA",1,"O4"],["NA",2,"C1"]],
[["NA",2,"O4"],["NA",3,"C1"]],
[["NA",3,"O3"],["NA",4,"C1"]],
[["NA",3,"O6"],["NA",5,"C1"]],
[["NA",4,"O2"],["NA",6,"C1"]],
[["NA",5,"O2"],["NA",7,"C1"]],
[["NA",6,"O4"],["NA",8,"C1"]],
[["NA",7,"O4"],["NA",9,"C1"]],
[["NA",8,"O6"],["NA",10,"C2"]],
[["NA",9,"O6"],["NA",11,"C2"]],
[["SIG",101,"ND2"],["NB",1,"C1"]],
[["NB",1,"O4"],["NB",2,"C1"]],
[["NB",2,"O4"],["NB",3,"C1"]],
[["NB",3,"O3"],["NB",4,"C1"]],
[["NB",3,"O6"],["NB",5,"C1"]],
[["NB",4,"O2"],["NB",6,"C1"]],
[["NB",5,"O2"],["NB",7,"C1"]],
[["NB",6,"O4"],["NB",8,"C1"]],
[["NB",7,"O4"],["NB",9,"C1"]],
[["NB",8,"O6"],["NB",10,"C2"]],
[["NB",9,"O6"],["NB",11,"C2"]],
[["SIG",112,"ND2"],["NC",1,"C1"]],
[["NC",1,"O4"],["NC",2,"C1"]],
[["NC",2,"O4"],["NC",3,"C1"]],
[["NC",3,"O3"],["NC",4,"C1"]],
[["NC",3,"O6"],["NC",5,"C1"]],
[["NC",4,"O2"],["NC",6,"C1"]],
[["NC",5,"O2"],["NC",7,"C1"]],
[["NC",6,"O4"],["NC",8,"C1"]],
[["NC",7,"O4"],["NC",9,"C1"]],
[["NC",8,"O6"],["NC",10,"C2"]],
[["NC",9,"O6"],["NC",11,"C2"]],
[["SIG",135,"ND2"],["ND",1,"C1"]],
[["ND",1,"O4"],["ND",2,"C1"]],
[["ND",2,"O4"],["ND",3,"C1"]],
[["ND",3,"O3"],["ND",4,"C1"]],
[["ND",3,"O6"],["ND",5,"C1"]],
[["ND",4,"O2"],["ND",6,"C1"]],
[["ND",5,"O2"],["ND",7,"C1"]],
[["ND",6,"O4"],["ND",8,"C1"]],
[["ND",7,"O4"],["ND",9,"C1"]],
[["ND",8,"O6"],["ND",10,"C2"]],
[["ND",9,"O6"],["ND",11,"C2"]],
[["SIG",164,"ND2"],["NE",1,"C1"]],
[["NE",1,"O4"],["NE",2,"C1"]],

[["NE",2,"O4"],["NE",3,"C1"]],
[["NE",3,"O3"],["NE",4,"C1"]],
[["NE",3,"O6"],["NE",5,"C1"]],
[["NE",4,"O2"],["NE",6,"C1"]],
[["NE",5,"O2"],["NE",7,"C1"]],
[["NE",6,"O4"],["NE",8,"C1"]],
[["NE",7,"O4"],["NE",9,"C1"]],
[["NE",8,"O6"],["NE",10,"C2"]],
[["NE",9,"O6"],["NE",11,"C2"]],
[["SIG",231,"ND2"],["NF",1,"C1"]],
[["NF",1,"O4"],["NF",2,"C1"]],
[["NF",2,"O4"],["NF",3,"C1"]],
[["NF",3,"O3"],["NF",4,"C1"]],
[["NF",3,"O6"],["NF",5,"C1"]],
[["NF",4,"O2"],["NF",6,"C1"]],
[["NF",5,"O2"],["NF",7,"C1"]],
[["NF",6,"O4"],["NF",8,"C1"]],
[["NF",7,"O4"],["NF",9,"C1"]],
[["NF",8,"O6"],["NF",10,"C2"]],
[["NF",9,"O6"],["NF",11,"C2"]],
[["SIG",363,"ND2"],["NG",1,"C1"]],
[["NG",1,"O4"],["NG",2,"C1"]],
[["NG",2,"O4"],["NG",3,"C1"]],
[["NG",3,"O3"],["NG",4,"C1"]],
[["NG",3,"O6"],["NG",5,"C1"]],
[["NG",4,"O2"],["NG",6,"C1"]],
[["NG",5,"O2"],["NG",7,"C1"]],
[["NG",6,"O4"],["NG",8,"C1"]],
[["NG",7,"O4"],["NG",9,"C1"]],
[["NG",8,"O6"],["NG",10,"C2"]],
[["NG",9,"O6"],["NG",11,"C2"]],
[["SIG",445,"ND2"],["NH",1,"C1"]],
[["NH",1,"O4"],["NH",2,"C1"]],
[["NH",2,"O4"],["NH",3,"C1"]],
[["NH",3,"O3"],["NH",4,"C1"]],
[["NH",3,"O6"],["NH",5,"C1"]],
[["NH",4,"O2"],["NH",6,"C1"]],
[["NH",5,"O2"],["NH",7,"C1"]],
[["NH",6,"O4"],["NH",8,"C1"]],
[["NH",7,"O4"],["NH",9,"C1"]],
[["NH",8,"O6"],["NH",10,"C2"]],
[["NH",9,"O6"],["NH",11,"C2"]],
[["SIG",479,"ND2"],["NI",1,"C1"]],
[["NI",1,"O4"],["NI",2,"C1"]],
[["NI",2,"O4"],["NI",3,"C1"]],
[["NI",3,"O3"],["NI",4,"C1"]],
[["NI",3,"O6"],["NI",5,"C1"]],
[["NI",4,"O2"],["NI",6,"C1"]],
[["NI",5,"O2"],["NI",7,"C1"]],
[["NI",6,"O4"],["NI",8,"C1"]],
[["NI",7,"O4"],["NI",9,"C1"]],
[["NI",8,"O6"],["NI",10,"C2"]],

[["NI",9,"O6"],["NI",11,"C2"]],
[["SIG",574,"ND2"],["NJ",1,"C1"]],
[["NJ",1,"O4"],["NJ",2,"C1"]],
[["NJ",2,"O4"],["NJ",3,"C1"]],
[["NJ",3,"O3"],["NJ",4,"C1"]],
[["NJ",3,"O6"],["NJ",5,"C1"]],
[["NJ",4,"O2"],["NJ",6,"C1"]],
[["NJ",5,"O2"],["NJ",7,"C1"]],
[["NJ",6,"O4"],["NJ",8,"C1"]],
[["NJ",7,"O4"],["NJ",9,"C1"]],
[["NJ",8,"O6"],["NJ",10,"C2"]],
[["NJ",9,"O6"],["NJ",11,"C2"]],
[["SIG",634,"ND2"],["NK",1,"C1"]],
[["NK",1,"O4"],["NK",2,"C1"]],
[["NK",2,"O4"],["NK",3,"C1"]],
[["NK",3,"O3"],["NK",4,"C1"]],
[["NK",3,"O6"],["NK",5,"C1"]],
[["NK",4,"O2"],["NK",6,"C1"]],
[["NK",5,"O2"],["NK",7,"C1"]],
[["NK",6,"O4"],["NK",8,"C1"]],
[["NK",7,"O4"],["NK",9,"C1"]],
[["NK",8,"O6"],["NK",10,"C2"]],
[["NK",9,"O6"],["NK",11,"C2"]]
],
"userCCD": "data_SIA2\n#\n_chem_comp.id SIA\n_chem_comp.name 'N-acetyl-alpha-neuraminic acid'\n_chem_comp.type 'D-saccharide, alpha linking'\n_chem_comp.pdbx_type ATOMS\n_chem_comp.formula 'C11 H19 N O9'\n_chem_comp.mon_nstd_parent_comp_id ?\n_chem_comp.pdbx_synonyms 'N-acetylneuraminic acid; sialic acid; alpha-sialic acid; O-SIALIC ACID'\n_chem_comp.pdbx_formal_charge 0\n_chem_comp.pdbx_initial_date 1999-07-08\n_chem_comp.pdbx_modified_date 2024-09-27\n_chem_comp.pdbx_ambiguous_flag N\n_chem_comp.pdbx_release_status REL\n_chem_comp.pdbx_replaced_by ?\n_chem_comp.pdbx_replaces NAN\n_chem_comp.formula_weight 309.270\n_chem_comp.one_letter_code ?\n_chem_comp.three_letter_code SIA\n_chem_comp.pdbx_model_coordinates_details ?\n_chem_comp.pdbx_model_coordinates_missing_flag N\n_chem_comp.pdbx_ideal_coordinates_details ?\n_chem_comp.pdbx_ideal_coordinates_missing_flag N\n_chem_comp.pdbx_model_coordinates_db_code ?\n_chem_comp.pdbx_subcomponent_list ?\n_chem_comp.pdbx_processing_site EBI\n_chem_comp.pdbx_pcm Y\n#\nloop_\n_pdbx_chem_comp_synonyms.ordinal\n_pdbx_chem_comp_synonyms.comp_id\n_pdbx_chem_comp_synonyms.name\n_pdbx_chem_comp_synonyms.provenance\n_pdbx_chem_comp_synonyms.type\n1 SIA 'N-acetylneuraminic acid' PDB ?\n2 SIA 'sialic acid' PDB ?\n3 SIA 'alpha-sialic acid' PDB ?\n4 SIA 'O-SIALIC ACID' PDB ?\n#\nloop_\n_chem_comp_atom.comp_id\n_chem_comp_atom.atom_id\n_chem_comp_atom.alt_atom_id\n_chem_comp_atom.type_symbol\n_chem_comp_atom.charge\n_chem_comp_atom.pdbx_align\n_chem_comp_atom.pdbx_aromatic_flag\n_chem_comp_atom.pdbx_leaving_atom_flag\n_chem_comp_atom.pdbx_stereo_config\n_chem_comp_atom.pdbx_backbone_atom_flag\n_chem_comp_atom.pdbx

_n_terminal_atom_flag\n_chem_comp_atom.pdbx_c_terminal_atom_flag\n_chem_comp_atom.model_Cartn_x\n_chem_comp_atom.model_Cartn_y\n_chem_comp_atom.model_Cartn_z\n_chem_comp_atom.pdbx_model_Cartn_x_ideal\n_chem_comp_atom.pdbx_model_Cartn_y_ideal\n_chem_comp_atom.pdbx_model_Cartn_z_ideal\n_chem_comp_atom.pdbx_component_atom_id\n_chem_comp_atom.pdbx_component_comp_id\n_chem_comp_atom.pdbx_ordinal\nSIA C1 C1 C 0 1 N N N N N N -2.196 58.872 -5.981 -2.502 -0.832 0.174 C1 SIA 1\nSIA C2 C2 C 0 1 N N R N N N -1.870 58.021 -7.211 -2.171 0.628 0.342 C2 SIA 2\nSIA C3 C3 C 0 1 N N N N N N -0.844 56.899 -7.306 -1.789 0.898 1.800 C3 SIA 3\nSIA C4 C4 C 0 1 N N S N N N -1.157 55.904 -8.413 -0.586 0.023 2.171 C4 SIA 4\nSIA C5 C5 C 0 1 N N R N N N -2.015 56.516 -9.517 0.529 0.264 1.148 C5 SIA 5\nSIA C6 C6 C 0 1 N N R N N N -3.352 56.956 -8.912 -0.026 0.043 -0.259 C6 SIA 6\nSIA C7 C7 C 0 1 N N R N N N -4.224 57.698 -9.942 1.088 0.251 -1.286 C7 SIA 7\nSIA C8 C8 C 0 1 N N R N N N -5.571 58.131 -9.360 0.535 0.021 -2.694 C8 SIA 8\nSIA C9 C9 C 0 1 N N N N N N -6.601 58.674 -10.381 1.650 0.229 -3.721 C9 SIA 9\nSIA C10 C10 C 0 1 N N N N N N -1.897 55.374 -11.759 2.632 -0.329 2.226 C10 SIA 10\nSIA C11 C11 C 0 1 N N N N N N -2.200 54.057 -12.454 3.763 -1.292 2.478 C11 SIA 11\nSIA N5 N5 N 0 1 N N N N N N -2.202 55.444 -10.478 1.629 -0.671 1.394 N5 SIA 12\nSIA O1A O1A O 0 1 N N N N N N -1.289 58.815 -5.130 -2.191 -1.408 -0.841 O1A SIA 13\nSIA O1B O1B O 0 1 N N N N N N -3.210 59.504 -5.631 -3.141 -1.493 1.152 O1B SIA 14\nSIA O4 O4 O 0 1 N N N N N N 0.072 55.523 -8.986 -0.123 0.370 3.478 O4 SIA 16\nSIA O6 O6 O 0 1 N N N N N N -3.149 57.908 -7.847 -1.082 0.968 -0.513 O6 SIA 17\nSIA O7 O7 O 0 1 N N N N N N -3.594 58.883 -10.402 1.588 1.586 -1.183 O7 SIA 18\nSIA O8 O8 O 0 1 N N N N N N -6.119 56.946 -8.828 0.035 -1.313 -2.797 O8 SIA 19\nSIA O9 O9 O 0 1 N N N N N N -6.931 57.687 -11.346 1.133 0.014 -5.035 O9 SIA 20\nSIA O10 O10 O 0 1 N N N N N N -1.423 56.357 -12.331 2.624 0.753 2.772 O10 SIA 21\nSIA H32 H31 H 0 1 N N N N N N -0.702 56.484 -6.300 -2.631 0.655 2.448 H32 SIA 22\nSIA H31 H32 H 0 1 N N N N N N 0.120 57.408 -7.182 -1.526 1.949 1.919 H31 SIA 23\nSIA H4 H4 H 0 1 N N N N N N -1.651 55.060 -7.897 -0.878 -1.026 2.153 H4 SIA 24\nSIA H5 H5 H 0 1 N N N N N N -1.506 57.375 -9.979 0.893 1.287 1.240 H5 SIA 25\nSIA H6 H6 H 0 1 N N N N N N -3.850 56.075 -8.492 -0.408 -0.973 -0.341 H6 SIA 26\nSIA H7 H7 H 0 1 N N N N N N -4.339 57.176 -10.907 1.896 -0.454 -1.093 H7 SIA 27\nSIA H8 H8 H 0 1 N N N N N N -5.473 58.871 -8.553 -0.272 0.728 -2.887 H8 SIA 28\nSIA H92 H91 H 0 1 N N N N N N -6.054 59.459 -10.925 2.031 1.247 -3.642 H92 SIA 29\nSIA H91 H92 H 0 1 N N N N N N -7.587 59.029 -10.055 2.457 -0.476 -3.528 H91 SIA 30\nSIA H111 H111 H 0 0 N N N N N N -3.215 53.728 -12.207 4.474 -0.844 3.172 H111 SIA 31\nSIA H113 H112 H 0 0 N N N N N N -1.550 53.279 -12.033 3.368 -2.213 2.907 H113 SIA 32\nSIA H112 H113 H 0 0 N N N N N N -2.005 54.041 -13.531 4.266 -1.516 1.537 H112 SIA 33\nSIA HN5 HN5 H 0 1 N N N N N N -2.566 54.658 -10.003 1.635 -1.538 0.957 HN5 SIA 34\nSIA HO1B HOB1 H 0 0 N N N N N N -3.412 60.032 -4.867 -3.353 -2.430 1.044 HO1B SIA 35\nSIA HO4 HO4 H 0 1 N Y N N N N 0.427 54.801 -8.430 -0.854 0.203 4.087 HO4 SIA 37\nSIA HO7 HO7 H 0 1 N Y N N N N -3.109 58.884 -9.548 0.844 2.177 -1.360 HO7 SIA 38\nSIA HO8 HO8 H 0 1 N Y N N N N -7.071 57.051 -8.962 0.779 -1.904 -2.620 HO8 SIA 39\nSIA HO9 HO9 H 0 1 N Y N N N N -6.783 56.885 -10.808 1.866 0.155 -5.650 HO9 SIA 40\n#\nloop_\n_chem_comp_bond.comp_id\n_chem_comp_bond.atom_id_1\n_che

m_comp_bond.atom_id_2\n_chem_comp_bond.value_order\n_chem_comp_bond.pdbx_aromatic_flag\n_chem_comp_bond.pdbx_stereo_config\n_chem_comp_bond.pdbx_ordinal\nSIA C1 C2 SING N N 1\nSIA C1 O1A DOUB N N 2\nSIA C1 O1B SING N N 3\nSIA C2 C3 SING N N 4\nSIA C2 O6 SING N N 6\nSIA C3 C4 SING N N 7\nSIA C3 H32 SING N N 8\nSIA C3 H31 SING N N 9\nSIA C4 C5 SING N N 10\nSIA C4 O4 SING N N 11\nSIA C4 H4 SING N N 12\nSIA C5 C6 SING N N 13\nSIA C5 N5 SING N N 14\nSIA C5 H5 SING N N 15\nSIA C6 C7 SING N N 16\nSIA C6 O6 SING N N 17\nSIA C6 H6 SING N N 18\nSIA C7 C8 SING N N 19\nSIA C7 O7 SING N N 20\nSIA C7 H7 SING N N 21\nSIA C8 C9 SING N N 22\nSIA C8 O8 SING N N 23\nSIA C8 H8 SING N N 24\nSIA C9 O9 SING N N 25\nSIA C9 H92 SING N N 26\nSIA C9 H91 SING N N 27\nSIA C10 C11 SING N N 28\nSIA C10 N5 SING N N 29\nSIA C10 O10 DOUB N N 30\nSIA C11 H111 SING N N 31\nSIA C11 H113 SING N N 32\nSIA C11 H112 SING N N 33\nSIA N5 HN5 SING N N 34\nSIA O1B HO1B SING N N 35\nSIA O4 HO4 SING N N 37\nSIA O7 HO7 SING N N 38\nSIA O8 HO8 SING N N 39\nSIA O9 HO9 SING N N
40\n#\nloop_\n_pdbx_chem_comp_descriptor.comp_id\n_pdbx_chem_comp_descriptor.type\n_pdbx_chem_comp_descriptor.program\n_pdbx_chem_comp_descriptor.program_version\n_pdbx_chem_comp_descriptor.descriptor\nSIA SMILES ACDLabs 10.04 'O=C(O)C1(O)OC(C(O)C(O)CO)C(NC(=O)C)C(O)C1'\nSIA SMILES_CANONICAL CACTVS 3.341
'CC(=O)N[C@@H]1[C@@H](O)C[C@@](O)(O[C@H]1[C@H](O)[C@H](O)CO)C(O)=O'\nSIA SMILES CACTVS 3.341
'CC(=O)N[CH]1[CH](O)C[C](O)(O[CH]1[CH](O)[CH](O)CO)C(O)=O'\nSIA SMILES_CANONICAL 'OpenEye OEToolkits' 1.5.0
'CC(=O)N[C@@H]1[C@H](C[C@@](O[C@H]1[C@@H]([C@@H](CO)O)O)(C(=O)O)O)O'\nSIA SMILES 'OpenEye OEToolkits' 1.5.0
'CC(=O)NC1C(CC(OC1C(C(CO)O)O)(C(=O)O)O)O'\nSIA InChI InChI 1.03
'InChI=1S/C11H19NO9/c1-4(14)12-7-5(15)2-11(20,10(18)19)21-9(7)8(17)6(16)3-13/h5-9,13,15-17,20H,2-3H2,1H3,(H,12,14)(H,18,19)/t5-,6+,7+,8+,9+,11+/m0/s1'\nSIA InChIKey InChI 1.03 SQVRNKJHWKZAKO-YRMXFSIDSA-N\n#\nloop_\n_pdbx_chem_comp_identifier.comp_id\n_pdbx_chem_comp_identifier.type\n_pdbx_chem_comp_identifier.program\n_pdbx_chem_comp_identifier.program_version\n_pdbx_chem_comp_identifier.identifier\nSIA 'SYSTEMATIC NAME' ACDLabs 10.04 '5-(acetylamino)-3,5-dideoxy-D-glycero-alpha-D-galacto-non-2-ulopyranosonic acid'\nSIA 'SYSTEMATIC NAME' 'OpenEye OEToolkits' 1.5.0 '(2R,4S,5R,6R)-5-acetamido-2,4-dihydroxy-6-[(1R,2R)-1,2,3-trihydroxypropyl]oxane-2-carboxylic acid'\nSIA 'CONDENSED IUPAC CARBOHYDRATE SYMBOL' GMML 1.0 DNeup5Aca\nSIA 'COMMON NAME' GMML 1.0 'N-acetyl-a-D-neuraminic acid'\nSIA 'IUPAC CARBOHYDRATE SYMBOL' PDB-CARE 1.0 a-D-Neup5Ac\nSIA 'SNFG CARBOHYDRATE SYMBOL' GMML 1.0
Neu5Ac\n#\nloop_\n_pdbx_chem_comp_feature.comp_id\n_pdbx_chem_comp_feature.type\n_pdbx_chem_comp_feature.value\n_pdbx_chem_comp_feature.source\n_pdbx_chem_comp_feature.support\nSIA 'CARBOHYDRATE ISOMER' D PDB ?\nSIA 'CARBOHYDRATE RING' pyranose PDB ?\nSIA 'CARBOHYDRATE ANOMER' alpha PDB ?\nSIA 'CARBOHYDRATE PRIMARY CARBONYL GROUP' ketose PDB ?\n#\nloop_\n_pdbx_chem_comp_audit.comp_id\n_pdbx_chem_comp_audit.action_type\n_pdbx_chem_comp_audit.date\n_pdbx_chem_comp_audit.processing_site\nSIA 'Create component' 1999-07-08 EBI\nSIA 'Modify descriptor' 2011-06-04 RCSB\nSIA 'Other modification' 2019-08-12

RCSB\nSIA 'Other modification' 2019-12-19 RCSB\nSIA 'Other modification' 2020-07-03 RCSB\nSIA 'Modify name' 2020-07-17 RCSB\nSIA 'Modify synonyms' 2020-07-17 RCSB\nSIA 'Modify atom id' 2020-07-17 RCSB\nSIA 'Modify component atom id' 2020-07-17 RCSB\nSIA 'Modify PCM' 2024-09-27 PDBE\n#\n_pdbx_chem_comp_pcm.pcm_id 1\n_pdbx_chem_comp_pcm.comp_id SIA\n_pdbx_chem_comp_pcm.modified_residue_id THR\n_pdbx_chem_comp_pcm.type None\n_pdbx_chem_comp_pcm.category Carbohydrate\n_pdbx_chem_comp_pcm.position 'Amino-acid side chain'\n_pdbx_chem_comp_pcm.polypeptide_position 'Any position'\n_pdbx_chem_comp_pcm.comp_id_linking_atom C2\n_pdbx_chem_comp_pcm.modified_residue_id_linking_atom OG1\n_pdbx_chem_comp_pcm.uniprot_specific_ptm_accession ?\n_pdbx_chem_comp_pcm.uniprot_generic_ptm_accession ?\n#\n_pdbe_chem_comp_drugbank_details.comp_id SIA\n_pdbe_chem_comp_drugbank_details.drugbank_id DB03721\n_pdbe_chem_comp_drugbank_details.type 'small molecule'\n_pdbe_chem_comp_drugbank_details.name 'N-acetyl-alpha-neuraminic acid'\n_pdbe_chem_comp_drugbank_details.description\n'An N-acyl derivative of neuraminic acid. N-acetylneuraminic acid occurs in many polysaccharides, glycoproteins, and glycolipids in animals and bacteria. (From Dorland, 28th ed, p1518)'\n_pdbe_chem_comp_drugbank_details.cas_number 21646-00-4\n_pdbe_chem_comp_drugbank_details.mechanism_of_action ?\n#\nloop_\n_pdbe_chem_comp_synonyms.comp_id\n_pdbe_chem_comp_synonyms.name\n_pdbe_chem_comp_synonyms.provenance\n_pdbe_chem_comp_synonyms.type\nSIA 'N-acetylneuraminic acid' wwPDB ?\nSIA 'sialic acid' wwPDB ?\nSIA 'alpha-sialic acid' wwPDB ?\nSIA 'O-SIALIC ACID' wwPDB ?\nSIA 'N-Acetyl-alpha-D-neuraminic acid' DrugBank ?\nSIA 'O-sialic acid' DrugBank ?\nSIA 'α-Neu5Ac' DrugBank ?\n#\n_pdbe_chem_comp_drugbank_classification.comp_id SIA\n_pdbe_chem_comp_drugbank_classification.drugbank_id DB03721\n_pdbe_chem_comp_drugbank_classification.parent 'N-acylneuraminic acids'\n_pdbe_chem_comp_drugbank_classification.kingdom 'Organic compounds'\n_pdbe_chem_comp_drugbank_classification.class 'Organooxygen compounds'\n_pdbe_chem_comp_drugbank_classification.superclass 'Organic oxygen compounds'\n_pdbe_chem_comp_drugbank_classification.description\n'This compound belongs to the class of organic compounds known as n-acylneuraminic acids. These are neuraminic acids carrying an N-acyl substituent.'\n#\nloop_\n_pdbe_chem_comp_drugbank_targets.comp_id\n_pdbe_chem_comp_drugbank_targets.drugbank_id\n_pdbe_chem_comp_drugbank_targets.name\n_pdbe_chem_comp_drugbank_targets.organism\n_pdbe_chem_comp_drugbank_targets.uniprot_id\n_pdbe_chem_comp_drugbank_targets.pharmacologically_active\n_pdbe_chem_comp_drugbank_targets.ordinal\nSIA DB03721 P-selectin Humans P16109 yes 1\nSIA DB03721 E-selectin Humans P16581 yes 2\nSIA DB03721 'Liver carboxylesterase 1' Humans P23141 yes 3\nSIA DB03721 '3-deoxy-manno-octulosonate cytidylyltransferase' 'Escherichia coli' P42216 unknown 4\nSIA DB03721 'Tetanus toxin' 'Clostridium tetani (strain Massachusetts / E88)' P04958 unknown 5\nSIA DB03721 'Cholera enterotoxin subunit B' 'Vibrio cholerae serotype O1 (strain ATCC 39315 / El Tor Inaba N16961)' P01556 unknown

6\nSIA DB03721 'Botulinum neurotoxin type B' 'Clostridium botulinum' P10844 unknown 7\nSIA DB03721 'Mannose-binding protein C' Humans P11226 unknown 8\nSIA DB03721 Lithostathine-1-alpha Humans P05451 unknown 9\nSIA DB03721 Endo-N-acetylneuraminidase 'Enterobacteria phage K1F' Q04830 unknown 10\nSIA DB03721 'Enterotoxin type B' 'Staphylococcus aureus' P01552 unknown 11\nSIA DB03721 Neuraminidase 'Influenza A virus (strain A/Tern/Australia/G70C/1975 H11N9)' P03472 unknown 12\nSIA DB03721 Hemagglutinin-neuraminidase NDV P32884 unknown 13\nSIA DB03721 Fiber 'Human adenovirus 19' Q64822 unknown 14\nSIA DB03721 Sialoadhesin Humans Q9BZZ2 unknown 15\nSIA DB03721 Zinc-alpha-2-glycoprotein Humans P25311 unknown 16\nSIA DB03721 'Capsid protein VP1' MPyV P49302 unknown 17\nSIA DB03721 Fiber 'Human adenovirus D37' Q64823 unknown
18\n#\nloop_\n_software.name\n_software.version\n_software.description \nrdkit 2023.09.6 'Core functionality.'\npdbeccdutils 0.8.6 'Wrapper to provide 2D templates and molecular
fragments.'\n#\nloop_\n_pdbe_chem_comp_atom_depiction.comp_id\n_pdbe_chem_comp_atom_depiction.atom_id\n_pdbe_chem_comp_atom_depiction.element\n_pdbe_chem_comp_atom_depiction.model_Cartn_x\n_pdbe_chem_comp_atom_depiction.model_Cartn_y\n_pdbe_chem_comp_atom_depiction.pdbx_ordinal\nSIA C1 C 5.654 -3.375 1\nSIA C2 C 6.404 -2.076 2\nSIA C3 C 5.104 -1.326 3\nSIA C4 C 5.104 0.174 4\nSIA C5 C 6.404 0.924 5\nSIA C6 C 7.702 0.174 6\nSIA C7 C 9.002 0.924 7\nSIA C8 C 10.301 0.174 8\nSIA C9 C 11.600 0.924 9\nSIA C10 C 5.104 3.174 10\nSIA C11 C 3.805 2.424 11\nSIA N5 N 6.404 2.424 12\nSIA O1A O 4.154 -3.375 13\nSIA O1B O 6.404 -4.674 14\nSIA O4 O 3.805 0.924 16\nSIA O6 O 7.702 -1.326 17\nSIA O7 O 9.002 2.424 18\nSIA O8 O 10.301 -1.326 19\nSIA O9 O 12.899 0.174 20\nSIA O10 O 5.104 4.674
21\n#\nloop_\n_pdbe_chem_comp_bond_depiction.comp_id\n_pdbe_chem_comp_bond_depiction.atom_id_1\n_pdbe_chem_comp_bond_depiction.atom_id_2\n_pdbe_chem_comp_bond_depiction.value_order\n_pdbe_chem_comp_bond_depiction.bond_dir\n_pdbe_chem_comp_bond_depiction.pdbx_ordinal\nSIA C1 C2 SINGLE NONE 1\nSIA C1 O1A DOUBLE NONE 2\nSIA C1 O1B SINGLE NONE 3\nSIA C2 C3 SINGLE NONE 4\nSIA C2 O6 SINGLE NONE 6\nSIA C3 C4 SINGLE NONE 7\nSIA C4 C5 SINGLE NONE 8\nSIA C4 O4 SINGLE BEGINDASH 9\nSIA C5 C6 SINGLE NONE 10\nSIA C5 N5 SINGLE BEGINWEDGE 11\nSIA C6 C7 SINGLE NONE 12\nSIA C6 O6 SINGLE BEGINDASH 13\nSIA C7 C8 SINGLE NONE 14\nSIA C7 O7 SINGLE BEGINDASH 15\nSIA C8 C9 SINGLE NONE 16\nSIA C8 O8 SINGLE BEGINWEDGE 17\nSIA C9 O9 SINGLE NONE 18\nSIA C10 C11 SINGLE NONE 19\nSIA C10 N5 SINGLE NONE 20\nSIA C10 O10 DOUBLE NONE
21\n#\nloop_\n_pdbe_chem_comp_substructure.comp_id\n_pdbe_chem_comp_substructure.substructure_name\n_pdbe_chem_comp_substructure.id\n_pdbe_chem_comp_substructure.substructure_type\n_pdbe_chem_comp_substructure.substructure_smiles\n_pdbe_chem_comp_substructure.substructure_inchis\n_pdbe_chem_comp_substructure.substructure_inchikeys\nSIA MurckoScaffold S1 scaffold C1CCOCC1 InChI=1S/C5H10O/c1-2-4-6-5-3-1/h1-5H2 DHXVGJBLRPWPCS-UHFFFAOYSA-N\nSIA amide F1 fragment CC(N)=O InChI=1S/C2H5NO/c1-2(3)4/h1H3,(H2,3,4) DLFVBJFMPXGRIB-UHFFFAOYSA-N\nSIA pyranose F2 fragment OC1CCCCO1 InChI=1S/C5H10O2/c6-5-3-1-2-4-7-5/h5-6H,1-4H2 CELWCAITJAEQNL-UHFFFAOYSA-
N\n#\nloop_\n_pdbe_chem_comp_substructure_mapping.comp_id\n_pdbe_chem_comp_substructure_mapping.atom_id\n_pdbe_chem_comp_substructure_mappin

g.substructure_id\n_pdbe_chem_comp_substructure_mapping.substructure_o
rdinal\nSIA C2 S1 1\nSIA C3 S1 1\nSIA C4 S1 1\nSIA C5 S1 1\nSIA C6 S1
1\nSIA O6 S1 1\nSIA N5 F1 1\nSIA C10 F1 1\nSIA O10 F1 1\nSIA C11 F1
1\nSIA C5 F2 1\nSIA C6 F2 1\nSIA O6 F2 1\nSIA C2 F2 1\nSIA C3 F2
1\nSIA C4 F2 1\n#\n_pdbe_chem_comp_rdkit_properties.comp_id
SIA\n_pdbe_chem_comp_rdkit_properties.exactmw
309.106\n_pdbe_chem_comp_rdkit_properties.amw
309.271\n_pdbe_chem_comp_rdkit_properties.lipinskiHBA
10\n_pdbe_chem_comp_rdkit_properties.lipinskiHBD
7\n_pdbe_chem_comp_rdkit_properties.NumRotatableBonds
11\n_pdbe_chem_comp_rdkit_properties.NumHBD
7\n_pdbe_chem_comp_rdkit_properties.NumHBA
9\n_pdbe_chem_comp_rdkit_properties.NumHeavyAtoms
21\n_pdbe_chem_comp_rdkit_properties.NumAtoms
40\n_pdbe_chem_comp_rdkit_properties.NumHeteroatoms
10\n_pdbe_chem_comp_rdkit_properties.NumAmideBonds
1\n_pdbe_chem_comp_rdkit_properties.FractionCSP3
0.818\n_pdbe_chem_comp_rdkit_properties.NumRings
1\n_pdbe_chem_comp_rdkit_properties.NumAromaticRings
0\n_pdbe_chem_comp_rdkit_properties.NumAliphaticRings
1\n_pdbe_chem_comp_rdkit_properties.NumSaturatedRings
1\n_pdbe_chem_comp_rdkit_properties.NumHeterocycles
1\n_pdbe_chem_comp_rdkit_properties.NumAromaticHeterocycles
0\n_pdbe_chem_comp_rdkit_properties.NumSaturatedHeterocycles
1\n_pdbe_chem_comp_rdkit_properties.NumAliphaticHeterocycles
1\n_pdbe_chem_comp_rdkit_properties.NumSpiroAtoms
0\n_pdbe_chem_comp_rdkit_properties.NumBridgeheadAtoms
0\n_pdbe_chem_comp_rdkit_properties.NumAtomStereoCenters
6\n_pdbe_chem_comp_rdkit_properties.NumUnspecifiedAtomStereoCenters
0\n_pdbe_chem_comp_rdkit_properties.labuteASA
146.407\n_pdbe_chem_comp_rdkit_properties.tpsa
176.780\n_pdbe_chem_comp_rdkit_properties.CrippenClogP -
3.872\n_pdbe_chem_comp_rdkit_properties.CrippenMR
64.787\n_pdbe_chem_comp_rdkit_properties.chi0v
9.621\n_pdbe_chem_comp_rdkit_properties.chi1v
4.738\n_pdbe_chem_comp_rdkit_properties.chi2v
1.941\n_pdbe_chem_comp_rdkit_properties.chi3v
1.941\n_pdbe_chem_comp_rdkit_properties.chi4v
1.128\n_pdbe_chem_comp_rdkit_properties.chi0n
28.621\n_pdbe_chem_comp_rdkit_properties.chi1n
13.635\n_pdbe_chem_comp_rdkit_properties.chi2n
1.941\n_pdbe_chem_comp_rdkit_properties.chi3n
1.941\n_pdbe_chem_comp_rdkit_properties.chi4n
1.128\n_pdbe_chem_comp_rdkit_properties.hallKierAlpha -
1.300\n_pdbe_chem_comp_rdkit_properties.kappa1
4.600\n_pdbe_chem_comp_rdkit_properties.kappa2
6.642\n_pdbe_chem_comp_rdkit_properties.kappa3
3.872\n_pdbe_chem_comp_rdkit_properties.Phi
1.455\n#\nloop_\n_pdbe_chem_comp_external_mappings.comp_id\n_pdbe_chem
_comp_external_mappings.source\n_pdbe_chem_comp_external_mappings.reso
urce\n_pdbe_chem_comp_external_mappings.resource_id\nSIA UniChem
ChEMBL CHEMBL1234621\nSIA UniChem DrugBank DB03721\nSIA UniChem ChEBI

49026\nSIA UniChem ZINC ZINC000004081651\nSIA UniChem fdasrs 04A90EXP8V\nSIA UniChem HMDB HMDB0000773\nSIA UniChem Nikkaji J614.853K\nSIA UniChem MetaboLights MTBLC49026\nSIA UniChem BRENDA 141715\nSIA UniChem BRENDA 233672\nSIA UniChem BRENDA 6105\nSIA UniChem BRENDA 84245\nSIA UniChem BRENDA 85625\nSIA UniChem 'Probes And Drugs' PD041137\nSIA UniChem PubChem 444885\nSIA UniChem eMolecules 474793\nSIA UniChem SureChEMBL SCHEMBL79085\nSIA UniChem 'PubChem TPHARMA' 14776495\nSIA UniChem 'PubChem TPHARMA' 15395566\n#\nloop_\n_pdbe_chem_comp_rdkit_conformer.comp_id\n_pdbe_chem_comp_rdkit_conformer.atom_id\n_pdbe_chem_comp_rdkit_conformer.Cartn_x_rdkit\n_pdbe_chem_comp_rdkit_conformer.Cartn_y_rdkit\n_pdbe_chem_comp_rdkit_conformer.Cartn_z_rdkit\n_pdbe_chem_comp_rdkit_conformer.rdkit_method\n_pdbe_chem_comp_rdkit_conformer.rdkit_ordinal\nSIA C1 -1.164 -2.811 1.058 ETKDGv3 1\nSIA C2 -1.666 -1.928 -0.070 ETKDGv3 2\nSIA C3 -2.639 -0.809 0.442 ETKDGv3 3\nSIA C4 -2.546 0.454 -0.428 ETKDGv3 4\nSIA C5 -1.079 0.960 -0.528 ETKDGv3 5\nSIA C6 -0.071 -0.181 -0.219 ETKDGv3 6\nSIA C7 1.341 0.133 -0.777 ETKDGv3 7\nSIA C8 2.378 -0.960 -0.400 ETKDGv3 8\nSIA C9 3.769 -0.651 -0.979 ETKDGv3 9\nSIA C10 -0.223 3.295 0.052 ETKDGv3 10\nSIA C11 0.035 4.333 1.094 ETKDGv3 11\nSIA N5 -0.868 2.066 0.414 ETKDGv3 12\nSIA O1A -0.936 -2.335 2.203 ETKDGv3 13\nSIA O1B -0.875 -4.149 0.806 ETKDGv3 14\nSIA O4 -3.104 0.224 -1.700 ETKDGv3 16\nSIA O6 -0.537 -1.404 -0.765 ETKDGv3 17\nSIA O7 1.298 0.320 -2.172 ETKDGv3 18\nSIA O8 2.484 -1.066 0.998 ETKDGv3 19\nSIA O9 4.246 0.587 -0.524 ETKDGv3 20\nSIA O10 0.157 3.497 -1.133 ETKDGv3 21\nSIA H32 -2.392 -0.510 1.484 ETKDGv3 22\nSIA H31 -3.691 -1.171 0.465 ETKDGv3 23\nSIA H4 -3.171 1.239 0.053 ETKDGv3 24\nSIA H5 -0.925 1.324 -1.568 ETKDGv3 25\nSIA H6 0.030 -0.270 0.887 ETKDGv3 26\nSIA H7 1.678 1.089 -0.320 ETKDGv3 27\nSIA H8 2.053 -1.935 -0.838 ETKDGv3 28\nSIA H92 3.719 -0.629 -2.088 ETKDGv3 29\nSIA H91 4.478 -1.464 -0.703 ETKDGv3 30\nSIA H111 0.843 3.984 1.770 ETKDGv3 31\nSIA H113 -0.888 4.508 1.686 ETKDGv3 32\nSIA H112 0.344 5.291 0.624 ETKDGv3 33\nSIA HN5 -1.113 1.913 1.419 ETKDGv3 34\nSIA HO1B -0.511 -4.749 1.536 ETKDGv3 35\nSIA HO4 -2.492 -0.372 -2.205 ETKDGv3 37\nSIA HO7 1.040 -0.545 -2.585 ETKDGv3 38\nSIA HO8 1.886 -1.804 1.285 ETKDGv3 39\nSIA HO9 4.592 0.441 0.395 ETKDGv3 40\n#\n"
}

Fig. 6f. G2S2 (GlcNAc6S) acting as trans-ligand interrupts HsSiglec-2 cis-interaction


```
{
    "name": "siglec2_g2s2_dimer_sul",
    "modelSeeds": [
      1
    ],
    "sequences": [
      {
        "protein": {
          "sequence":
"MHLLGPWLLLLVLEYLAFSDSSKWVFEHPETLYAWEGACVWIPCTYRALDGDLESFILFHNPEYNKNTS
KFDGTRLYESTKDGKVPSEQKRVQFLGDKNKNCTLSIHPVHLNDSGQLGLRMESKTEKWMERIHLNVSER
PFPPHIQLPPEIQESQEVTLTCLLNFSCYGYPIQLQWLLEGVPMRQAAVTSTSLTIKSVFTRSELKFSPQ
WSHHGKIVTCQLQDADGKFLSNDTVQLNVKHTPKLEIKVTPSDAIVREGDSVTMTCEVSSSNPEYTTVSW
LKDGTSLKKQNTFTLNLREVTKDQSGKYCCQVSNDVGPGRSEEVFLQVQYAPEPSTVQILHSPAVEGSQV
EFLCMSLANPLPTNYTWYHNGKEMQGRTEEKVHIPKILPWHAGTYSCVAENILGTGQRGPGAELDVQYPP
KKVTTVIQNPMPIREGDTVTLSCNYNSSNPSVTRYEWKPHGAWEEPSLGVLKIQNVGWDNTTIACAACNS
WCSWASPVALNVQYAPRDVRVRKIKPLSEIHSGNSVSLQCDFSSSHPKEVQFFWEKNGRLLGKESQLNFD
SISPEDAGSYSCWVNNSIGQTASKAWTLEVLYAPRRLRVSMSPGDQVMEGKSATLTCESDANPPVSHYTW
FDWNNQSLPYHSQKLRLEPVKVQHSGAYWCQGTNSVGKGRSPLSTLTVYYSPETIGRRVAVGLGSCLAIL
ILAICGLKLQRRWKRTQSQQGLQENSSGQSFFVRNKKVRRAPLSEGPHSLGCYNPMMEDGISYTTLRFPE
MNIPRTGDAESSEMQRPPPDCDDTVTYSALHKRQVGDYENVIPDFPEDEGIHYSELIQFGVGERPQAQEN
VDYVILKH",
          "id": ["SIGLEC", "SIG"]
        }
      },
      {
        "ligand": {
          "ccdCodes": [
            "NAG", "NAG", "BMA", "MAN", "MAN", "NAG", "NAG", "GAL",
"GAL", "SIA-2", "SIA-2"
          ],
          "id": ["NGA", "NA"]
        }
      },
      {
        "ligand": {
          "ccdCodes": [
            "NAG", "NAG", "BMA", "MAN", "MAN", "NAG", "NAG", "GAL",
"GAL", "SIA-2", "SIA-2"
          ],
          "id": ["NGB", "NGC", "NGD", "NGE", "NGF", "NGG", "NGH",
"NGI", "NGJ", "NGK", "NB", "NC", "ND", "NE", "NF", "NG", "NH", "NI",
"NJ", "NK"]
        }
      },
      {
        "ligand": {
          "ccdCodes": [
            "NAG", "NAG", "BMA", "MAN", "MAN", "NGS", "NGS", "GAL",
"GAL", "SIA-2", "SIA-2"
```

```
      ],
      "id": "EPONG"
    }
  }
],
"dialect": "alphafold3",
"version": 2,
"bondedAtomPairs": [
[["EPONG",1,"O4"],["EPONG",2,"C1"]],
[["EPONG",2,"O4"],["EPONG",3,"C1"]],
[["EPONG",3,"O3"],["EPONG",4,"C1"]],
[["EPONG",3,"O6"],["EPONG",5,"C1"]],
[["EPONG",4,"O2"],["EPONG",6,"C1"]],
[["EPONG",5,"O2"],["EPONG",7,"C1"]],
[["EPONG",6,"O4"],["EPONG",8,"C1"]],
[["EPONG",7,"O4"],["EPONG",9,"C1"]],
[["EPONG",8,"O6"],["EPONG",10,"C2"]],
[["EPONG",9,"O6"],["EPONG",11,"C2"]],

[["SIGLEC",67,"ND2"],["NGA",1,"C1"]],
[["NGA",1,"O4"],["NGA",2,"C1"]],
[["NGA",2,"O4"],["NGA",3,"C1"]],
[["NGA",3,"O3"],["NGA",4,"C1"]],
[["NGA",3,"O6"],["NGA",5,"C1"]],
[["NGA",4,"O2"],["NGA",6,"C1"]],
[["NGA",5,"O2"],["NGA",7,"C1"]],
[["NGA",6,"O4"],["NGA",8,"C1"]],
[["NGA",7,"O4"],["NGA",9,"C1"]],
[["NGA",8,"O6"],["NGA",10,"C2"]],
[["NGA",9,"O6"],["NGA",11,"C2"]],
[["SIGLEC",101,"ND2"],["NGB",1,"C1"]],
[["NGB",1,"O4"],["NGB",2,"C1"]],
[["NGB",2,"O4"],["NGB",3,"C1"]],
[["NGB",3,"O3"],["NGB",4,"C1"]],
[["NGB",3,"O6"],["NGB",5,"C1"]],
[["NGB",4,"O2"],["NGB",6,"C1"]],
[["NGB",5,"O2"],["NGB",7,"C1"]],
[["NGB",6,"O4"],["NGB",8,"C1"]],
[["NGB",7,"O4"],["NGB",9,"C1"]],
[["NGB",8,"O6"],["NGB",10,"C2"]],
[["NGB",9,"O6"],["NGB",11,"C2"]],
[["SIGLEC",112,"ND2"],["NGC",1,"C1"]],
[["NGC",1,"O4"],["NGC",2,"C1"]],
[["NGC",2,"O4"],["NGC",3,"C1"]],
[["NGC",3,"O3"],["NGC",4,"C1"]],
[["NGC",3,"O6"],["NGC",5,"C1"]],
[["NGC",4,"O2"],["NGC",6,"C1"]],
[["NGC",5,"O2"],["NGC",7,"C1"]],
[["NGC",6,"O4"],["NGC",8,"C1"]],
[["NGC",7,"O4"],["NGC",9,"C1"]],
[["NGC",8,"O6"],["NGC",10,"C2"]],
[["NGC",9,"O6"],["NGC",11,"C2"]],
```

[["SIGLEC",135,"ND2"],["NGD",1,"C1"]],
[["NGD",1,"O4"],["NGD",2,"C1"]],
[["NGD",2,"O4"],["NGD",3,"C1"]],
[["NGD",3,"O3"],["NGD",4,"C1"]],
[["NGD",3,"O6"],["NGD",5,"C1"]],
[["NGD",4,"O2"],["NGD",6,"C1"]],
[["NGD",5,"O2"],["NGD",7,"C1"]],
[["NGD",6,"O4"],["NGD",8,"C1"]],
[["NGD",7,"O4"],["NGD",9,"C1"]],
[["NGD",8,"O6"],["NGD",10,"C2"]],
[["NGD",9,"O6"],["NGD",11,"C2"]],
[["SIGLEC",164,"ND2"],["NGE",1,"C1"]],
[["NGE",1,"O4"],["NGE",2,"C1"]],
[["NGE",2,"O4"],["NGE",3,"C1"]],
[["NGE",3,"O3"],["NGE",4,"C1"]],
[["NGE",3,"O6"],["NGE",5,"C1"]],
[["NGE",4,"O2"],["NGE",6,"C1"]],
[["NGE",5,"O2"],["NGE",7,"C1"]],
[["NGE",6,"O4"],["NGE",8,"C1"]],
[["NGE",7,"O4"],["NGE",9,"C1"]],
[["NGE",8,"O6"],["NGE",10,"C2"]],
[["NGE",9,"O6"],["NGE",11,"C2"]],
[["SIGLEC",231,"ND2"],["NGF",1,"C1"]],
[["NGF",1,"O4"],["NGF",2,"C1"]],
[["NGF",2,"O4"],["NGF",3,"C1"]],
[["NGF",3,"O3"],["NGF",4,"C1"]],
[["NGF",3,"O6"],["NGF",5,"C1"]],
[["NGF",4,"O2"],["NGF",6,"C1"]],
[["NGF",5,"O2"],["NGF",7,"C1"]],
[["NGF",6,"O4"],["NGF",8,"C1"]],
[["NGF",7,"O4"],["NGF",9,"C1"]],
[["NGF",8,"O6"],["NGF",10,"C2"]],
[["NGF",9,"O6"],["NGF",11,"C2"]],
[["SIGLEC",363,"ND2"],["NGG",1,"C1"]],
[["NGG",1,"O4"],["NGG",2,"C1"]],
[["NGG",2,"O4"],["NGG",3,"C1"]],
[["NGG",3,"O3"],["NGG",4,"C1"]],
[["NGG",3,"O6"],["NGG",5,"C1"]],
[["NGG",4,"O2"],["NGG",6,"C1"]],
[["NGG",5,"O2"],["NGG",7,"C1"]],
[["NGG",6,"O4"],["NGG",8,"C1"]],
[["NGG",7,"O4"],["NGG",9,"C1"]],
[["NGG",8,"O6"],["NGG",10,"C2"]],
[["NGG",9,"O6"],["NGG",11,"C2"]],
[["SIGLEC",445,"ND2"],["NGH",1,"C1"]],
[["NGH",1,"O4"],["NGH",2,"C1"]],
[["NGH",2,"O4"],["NGH",3,"C1"]],
[["NGH",3,"O3"],["NGH",4,"C1"]],
[["NGH",3,"O6"],["NGH",5,"C1"]],
[["NGH",4,"O2"],["NGH",6,"C1"]],
[["NGH",5,"O2"],["NGH",7,"C1"]],
[["NGH",6,"O4"],["NGH",8,"C1"]],

[["NGH",7,"O4"],["NGH",9,"C1"]],
[["NGH",8,"O6"],["NGH",10,"C2"]],
[["NGH",9,"O6"],["NGH",11,"C2"]],
[["SIGLEC",479,"ND2"],["NGI",1,"C1"]],
[["NGI",1,"O4"],["NGI",2,"C1"]],
[["NGI",2,"O4"],["NGI",3,"C1"]],
[["NGI",3,"O3"],["NGI",4,"C1"]],
[["NGI",3,"O6"],["NGI",5,"C1"]],
[["NGI",4,"O2"],["NGI",6,"C1"]],
[["NGI",5,"O2"],["NGI",7,"C1"]],
[["NGI",6,"O4"],["NGI",8,"C1"]],
[["NGI",7,"O4"],["NGI",9,"C1"]],
[["NGI",8,"O6"],["NGI",10,"C2"]],
[["NGI",9,"O6"],["NGI",11,"C2"]],
[["SIGLEC",574,"ND2"],["NGJ",1,"C1"]],
[["NGJ",1,"O4"],["NGJ",2,"C1"]],
[["NGJ",2,"O4"],["NGJ",3,"C1"]],
[["NGJ",3,"O3"],["NGJ",4,"C1"]],
[["NGJ",3,"O6"],["NGJ",5,"C1"]],
[["NGJ",4,"O2"],["NGJ",6,"C1"]],
[["NGJ",5,"O2"],["NGJ",7,"C1"]],
[["NGJ",6,"O4"],["NGJ",8,"C1"]],
[["NGJ",7,"O4"],["NGJ",9,"C1"]],
[["NGJ",8,"O6"],["NGJ",10,"C2"]],
[["NGJ",9,"O6"],["NGJ",11,"C2"]],
[["SIGLEC",634,"ND2"],["NGK",1,"C1"]],
[["NGK",1,"O4"],["NGK",2,"C1"]],
[["NGK",2,"O4"],["NGK",3,"C1"]],
[["NGK",3,"O3"],["NGK",4,"C1"]],
[["NGK",3,"O6"],["NGK",5,"C1"]],
[["NGK",4,"O2"],["NGK",6,"C1"]],
[["NGK",5,"O2"],["NGK",7,"C1"]],
[["NGK",6,"O4"],["NGK",8,"C1"]],
[["NGK",7,"O4"],["NGK",9,"C1"]],
[["NGK",8,"O6"],["NGK",10,"C2"]],
[["NGK",9,"O6"],["NGK",11,"C2"]],
[["SIG",67,"ND2"],["NA",1,"C1"]],
[["NA",1,"O4"],["NA",2,"C1"]],
[["NA",2,"O4"],["NA",3,"C1"]],
[["NA",3,"O3"],["NA",4,"C1"]],
[["NA",3,"O6"],["NA",5,"C1"]],
[["NA",4,"O2"],["NA",6,"C1"]],
[["NA",5,"O2"],["NA",7,"C1"]],
[["NA",6,"O4"],["NA",8,"C1"]],
[["NA",7,"O4"],["NA",9,"C1"]],
[["NA",8,"O6"],["NA",10,"C2"]],
[["NA",9,"O6"],["NA",11,"C2"]],
[["SIG",101,"ND2"],["NB",1,"C1"]],
[["NB",1,"O4"],["NB",2,"C1"]],
[["NB",2,"O4"],["NB",3,"C1"]],
[["NB",3,"O3"],["NB",4,"C1"]],
[["NB",3,"O6"],["NB",5,"C1"]],

[["NB",4,"O2"],["NB",6,"C1"]],
[["NB",5,"O2"],["NB",7,"C1"]],
[["NB",6,"O4"],["NB",8,"C1"]],
[["NB",7,"O4"],["NB",9,"C1"]],
[["NB",8,"O6"],["NB",10,"C2"]],
[["NB",9,"O6"],["NB",11,"C2"]],
[["SIG",112,"ND2"],["NC",1,"C1"]],
[["NC",1,"O4"],["NC",2,"C1"]],
[["NC",2,"O4"],["NC",3,"C1"]],
[["NC",3,"O3"],["NC",4,"C1"]],
[["NC",3,"O6"],["NC",5,"C1"]],
[["NC",4,"O2"],["NC",6,"C1"]],
[["NC",5,"O2"],["NC",7,"C1"]],
[["NC",6,"O4"],["NC",8,"C1"]],
[["NC",7,"O4"],["NC",9,"C1"]],
[["NC",8,"O6"],["NC",10,"C2"]],
[["NC",9,"O6"],["NC",11,"C2"]],
[["SIG",135,"ND2"],["ND",1,"C1"]],
[["ND",1,"O4"],["ND",2,"C1"]],
[["ND",2,"O4"],["ND",3,"C1"]],
[["ND",3,"O3"],["ND",4,"C1"]],
[["ND",3,"O6"],["ND",5,"C1"]],
[["ND",4,"O2"],["ND",6,"C1"]],
[["ND",5,"O2"],["ND",7,"C1"]],
[["ND",6,"O4"],["ND",8,"C1"]],
[["ND",7,"O4"],["ND",9,"C1"]],
[["ND",8,"O6"],["ND",10,"C2"]],
[["ND",9,"O6"],["ND",11,"C2"]],
[["SIG",164,"ND2"],["NE",1,"C1"]],
[["NE",1,"O4"],["NE",2,"C1"]],
[["NE",2,"O4"],["NE",3,"C1"]],
[["NE",3,"O3"],["NE",4,"C1"]],
[["NE",3,"O6"],["NE",5,"C1"]],
[["NE",4,"O2"],["NE",6,"C1"]],
[["NE",5,"O2"],["NE",7,"C1"]],
[["NE",6,"O4"],["NE",8,"C1"]],
[["NE",7,"O4"],["NE",9,"C1"]],
[["NE",8,"O6"],["NE",10,"C2"]],
[["NE",9,"O6"],["NE",11,"C2"]],
[["SIG",231,"ND2"],["NF",1,"C1"]],
[["NF",1,"O4"],["NF",2,"C1"]],
[["NF",2,"O4"],["NF",3,"C1"]],
[["NF",3,"O3"],["NF",4,"C1"]],
[["NF",3,"O6"],["NF",5,"C1"]],
[["NF",4,"O2"],["NF",6,"C1"]],
[["NF",5,"O2"],["NF",7,"C1"]],
[["NF",6,"O4"],["NF",8,"C1"]],
[["NF",7,"O4"],["NF",9,"C1"]],
[["NF",8,"O6"],["NF",10,"C2"]],
[["NF",9,"O6"],["NF",11,"C2"]],
[["SIG",363,"ND2"],["NG",1,"C1"]],
[["NG",1,"O4"],["NG",2,"C1"]],

[["NG",2,"O4"],["NG",3,"C1"]],
[["NG",3,"O3"],["NG",4,"C1"]],
[["NG",3,"O6"],["NG",5,"C1"]],
[["NG",4,"O2"],["NG",6,"C1"]],
[["NG",5,"O2"],["NG",7,"C1"]],
[["NG",6,"O4"],["NG",8,"C1"]],
[["NG",7,"O4"],["NG",9,"C1"]],
[["NG",8,"O6"],["NG",10,"C2"]],
[["NG",9,"O6"],["NG",11,"C2"]],
[["SIG",445,"ND2"],["NH",1,"C1"]],
[["NH",1,"O4"],["NH",2,"C1"]],
[["NH",2,"O4"],["NH",3,"C1"]],
[["NH",3,"O3"],["NH",4,"C1"]],
[["NH",3,"O6"],["NH",5,"C1"]],
[["NH",4,"O2"],["NH",6,"C1"]],
[["NH",5,"O2"],["NH",7,"C1"]],
[["NH",6,"O4"],["NH",8,"C1"]],
[["NH",7,"O4"],["NH",9,"C1"]],
[["NH",8,"O6"],["NH",10,"C2"]],
[["NH",9,"O6"],["NH",11,"C2"]],
[["SIG",479,"ND2"],["NI",1,"C1"]],
[["NI",1,"O4"],["NI",2,"C1"]],
[["NI",2,"O4"],["NI",3,"C1"]],
[["NI",3,"O3"],["NI",4,"C1"]],
[["NI",3,"O6"],["NI",5,"C1"]],
[["NI",4,"O2"],["NI",6,"C1"]],
[["NI",5,"O2"],["NI",7,"C1"]],
[["NI",6,"O4"],["NI",8,"C1"]],
[["NI",7,"O4"],["NI",9,"C1"]],
[["NI",8,"O6"],["NI",10,"C2"]],
[["NI",9,"O6"],["NI",11,"C2"]],
[["SIG",574,"ND2"],["NJ",1,"C1"]],
[["NJ",1,"O4"],["NJ",2,"C1"]],
[["NJ",2,"O4"],["NJ",3,"C1"]],
[["NJ",3,"O3"],["NJ",4,"C1"]],
[["NJ",3,"O6"],["NJ",5,"C1"]],
[["NJ",4,"O2"],["NJ",6,"C1"]],
[["NJ",5,"O2"],["NJ",7,"C1"]],
[["NJ",6,"O4"],["NJ",8,"C1"]],
[["NJ",7,"O4"],["NJ",9,"C1"]],
[["NJ",8,"O6"],["NJ",10,"C2"]],
[["NJ",9,"O6"],["NJ",11,"C2"]],
[["SIG",634,"ND2"],["NK",1,"C1"]],
[["NK",1,"O4"],["NK",2,"C1"]],
[["NK",2,"O4"],["NK",3,"C1"]],
[["NK",3,"O3"],["NK",4,"C1"]],
[["NK",3,"O6"],["NK",5,"C1"]],
[["NK",4,"O2"],["NK",6,"C1"]],
[["NK",5,"O2"],["NK",7,"C1"]],
[["NK",6,"O4"],["NK",8,"C1"]],
[["NK",7,"O4"],["NK",9,"C1"]],
[["NK",8,"O6"],["NK",10,"C2"]],

[["NK",9,"O6"],["NK",11,"C2"]]
],
"userCCD": "data_SIA-2\n#\n_chem_comp.id SIA\n_chem_comp.name 'N-acetyl-alpha-neuraminic acid'\n_chem_comp.type 'D-saccharide, alpha linking'\n_chem_comp.pdbx_type ATOMS\n_chem_comp.formula 'C11 H19 N O9'\n_chem_comp.mon_nstd_parent_comp_id ?\n_chem_comp.pdbx_synonyms 'N-acetylneuraminic acid; sialic acid; alpha-sialic acid; O-SIALIC ACID'\n_chem_comp.pdbx_formal_charge 0\n_chem_comp.pdbx_initial_date 1999-07-08\n_chem_comp.pdbx_modified_date 2024-09-27\n_chem_comp.pdbx_ambiguous_flag N\n_chem_comp.pdbx_release_status REL\n_chem_comp.pdbx_replaced_by ?\n_chem_comp.pdbx_replaces NAN\n_chem_comp.formula_weight 309.270\n_chem_comp.one_letter_code ?\n_chem_comp.three_letter_code SIA\n_chem_comp.pdbx_model_coordinates_details ?\n_chem_comp.pdbx_model_coordinates_missing_flag N\n_chem_comp.pdbx_ideal_coordinates_details ?\n_chem_comp.pdbx_ideal_coordinates_missing_flag N\n_chem_comp.pdbx_model_coordinates_db_code ?\n_chem_comp.pdbx_subcomponent_list ?\n_chem_comp.pdbx_processing_site EBI\n_chem_comp.pdbx_pcm Y\n#\nloop_\n_pdbx_chem_comp_synonyms.ordinal\n_pdbx_chem_comp_synonyms.comp_id\n_pdbx_chem_comp_synonyms.name\n_pdbx_chem_comp_synonyms.provenance\n_pdbx_chem_comp_synonyms.type\n1 SIA 'N-acetylneuraminic acid' PDB ?\n2 SIA 'sialic acid' PDB ?\n3 SIA 'alpha-sialic acid' PDB ?\n4 SIA 'O-SIALIC ACID' PDB ?\n#\nloop_\n_chem_comp_atom.comp_id\n_chem_comp_atom.atom_id\n_chem_comp_atom.alt_atom_id\n_chem_comp_atom.type_symbol\n_chem_comp_atom.charge\n_chem_comp_atom.pdbx_align\n_chem_comp_atom.pdbx_aromatic_flag\n_chem_comp_atom.pdbx_leaving_atom_flag\n_chem_comp_atom.pdbx_stereo_config\n_chem_comp_atom.pdbx_backbone_atom_flag\n_chem_comp_atom.pdbx_n_terminal_atom_flag\n_chem_comp_atom.pdbx_c_terminal_atom_flag\n_chem_comp_atom.model_Cartn_x\n_chem_comp_atom.model_Cartn_y\n_chem_comp_atom.model_Cartn_z\n_chem_comp_atom.pdbx_model_Cartn_x_ideal\n_chem_comp_atom.pdbx_model_Cartn_y_ideal\n_chem_comp_atom.pdbx_model_Cartn_z_ideal\n_chem_comp_atom.pdbx_component_atom_id\n_chem_comp_atom.pdbx_component_comp_id\n_chem_comp_atom.pdbx_ordinal\nSIA C1 C1 C 0 1 N N N N N N -2.196 58.872 -5.981 -2.502 -0.832 0.174 C1 SIA 1\nSIA C2 C2 C 0 1 N N R N N N -1.870 58.021 -7.211 -2.171 0.628 0.342 C2 SIA 2\nSIA C3 C3 C 0 1 N N N N N N -0.844 56.899 -7.306 -1.789 0.898 1.800 C3 SIA 3\nSIA C4 C4 C 0 1 N N S N N N -1.157 55.904 -8.413 -0.586 0.023 2.171 C4 SIA 4\nSIA C5 C5 C 0 1 N N R N N N -2.015 56.516 -9.517 0.529 0.264 1.148 C5 SIA 5\nSIA C6 C6 C 0 1 N N R N N N -3.352 56.956 -8.912 -0.026 0.043 -0.259 C6 SIA 6\nSIA C7 C7 C 0 1 N N R N N N -4.224 57.698 -9.942 1.088 0.251 -1.286 C7 SIA 7\nSIA C8 C8 C 0 1 N N R N N N -5.571 58.131 -9.360 0.535 0.021 -2.694 C8 SIA 8\nSIA C9 C9 C 0 1 N N N N N N -6.601 58.674 -10.381 1.650 0.229 -3.721 C9 SIA 9\nSIA C10 C10 C 0 1 N N N N N N -1.897 55.374 -11.759 2.632 -0.329 2.226 C10 SIA 10\nSIA C11 C11 C 0 1 N N N N N N -2.200 54.057 -12.454 3.763 -1.292 2.478 C11 SIA 11\nSIA N5 N5 N 0 1 N N N N N N -2.202 55.444 -10.478 1.629 -0.671 1.394 N5 SIA 12\nSIA O1A O1A O 0 1 N N N N N N -1.289 58.815 -5.130 -2.191 -1.408 -0.841 O1A SIA 13\nSIA O1B O1B O 0 1 N N N N N N -3.210 59.504 -5.631 -3.141 -1.493 1.152 O1B SIA 14\nSIA O4 O4 O 0 1 N N N N

N N 0.072 55.523 -8.986 -0.123 0.370 3.478 O4 SIA 16\nSIA O6 O6 O 0 1 N N N N N N -3.149 57.908 -7.847 -1.082 0.968 -0.513 O6 SIA 17\nSIA O7 O7 O 0 1 N N N N N N -3.594 58.883 -10.402 1.588 1.586 -1.183 O7 SIA 18\nSIA O8 O8 O 0 1 N N N N N N -6.119 56.946 -8.828 0.035 -1.313 -2.797 O8 SIA 19\nSIA O9 O9 O 0 1 N N N N N N -6.931 57.687 -11.346 1.133 0.014 -5.035 O9 SIA 20\nSIA O10 O10 O 0 1 N N N N N N -1.423 56.357 -12.331 2.624 0.753 2.772 O10 SIA 21\nSIA H32 H31 H 0 1 N N N N N N -0.702 56.484 -6.300 -2.631 0.655 2.448 H32 SIA 22\nSIA H31 H32 H 0 1 N N N N N N 0.120 57.408 -7.182 -1.526 1.949 1.919 H31 SIA 23\nSIA H4 H4 H 0 1 N N N N N N -1.651 55.060 -7.897 -0.878 -1.026 2.153 H4 SIA 24\nSIA H5 H5 H 0 1 N N N N N N -1.506 57.375 -9.979 0.893 1.287 1.240 H5 SIA 25\nSIA H6 H6 H 0 1 N N N N N N -3.850 56.075 -8.492 -0.408 -0.973 -0.341 H6 SIA 26\nSIA H7 H7 H 0 1 N N N N N N -4.339 57.176 -10.907 1.896 -0.454 -1.093 H7 SIA 27\nSIA H8 H8 H 0 1 N N N N N N -5.473 58.871 -8.553 -0.272 0.728 -2.887 H8 SIA 28\nSIA H92 H91 H 0 1 N N N N N N -6.054 59.459 -10.925 2.031 1.247 -3.642 H92 SIA 29\nSIA H91 H92 H 0 1 N N N N N N -7.587 59.029 -10.055 2.457 -0.476 -3.528 H91 SIA 30\nSIA H111 H111 H 0 0 N N N N N N -3.215 53.728 -12.207 4.474 -0.844 3.172 H111 SIA 31\nSIA H113 H112 H 0 0 N N N N N N -1.550 53.279 -12.033 3.368 -2.213 2.907 H113 SIA 32\nSIA H112 H113 H 0 0 N N N N N N -2.005 54.041 -13.531 4.266 -1.516 1.537 H112 SIA 33\nSIA HN5 HN5 H 0 1 N N N N N N -2.566 54.658 -10.003 1.635 -1.538 0.957 HN5 SIA 34\nSIA HO1B HOB1 H 0 0 N N N N N N -3.412 60.032 -4.867 -3.353 -2.430 1.044 HO1B SIA 35\nSIA HO4 HO4 H 0 1 N Y N N N N 0.427 54.801 -8.430 -0.854 0.203 4.087 HO4 SIA 37\nSIA HO7 HO7 H 0 1 N Y N N N N -3.109 58.884 -9.548 0.844 2.177 -1.360 HO7 SIA 38\nSIA HO8 HO8 H 0 1 N Y N N N N -7.071 57.051 -8.962 0.779 -1.904 -2.620 HO8 SIA 39\nSIA HO9 HO9 H 0 1 N Y N N N N -6.783 56.885 -10.808 1.866 0.155 -5.650 HO9 SIA
40\n#\nloop_\n_chem_comp_bond.comp_id\n_chem_comp_bond.atom_id_1\n_chem_comp_bond.atom_id_2\n_chem_comp_bond.value_order\n_chem_comp_bond.pdbx_aromatic_flag\n_chem_comp_bond.pdbx_stereo_config\n_chem_comp_bond.pdbx_ordinal\nSIA C1 C2 SING N N 1\nSIA C1 O1A DOUB N N 2\nSIA C1 O1B SING N N 3\nSIA C2 C3 SING N N 4\nSIA C2 O6 SING N N 6\nSIA C3 C4 SING N N 7\nSIA C3 H32 SING N N 8\nSIA C3 H31 SING N N 9\nSIA C4 C5 SING N N 10\nSIA C4 O4 SING N N 11\nSIA C4 H4 SING N N 12\nSIA C5 C6 SING N N 13\nSIA C5 N5 SING N N 14\nSIA C5 H5 SING N N 15\nSIA C6 C7 SING N N 16\nSIA C6 O6 SING N N 17\nSIA C6 H6 SING N N 18\nSIA C7 C8 SING N N 19\nSIA C7 O7 SING N N 20\nSIA C7 H7 SING N N 21\nSIA C8 C9 SING N N 22\nSIA C8 O8 SING N N 23\nSIA C8 H8 SING N N 24\nSIA C9 O9 SING N N 25\nSIA C9 H92 SING N N 26\nSIA C9 H91 SING N N 27\nSIA C10 C11 SING N N 28\nSIA C10 N5 SING N N 29\nSIA C10 O10 DOUB N N 30\nSIA C11 H111 SING N N 31\nSIA C11 H113 SING N N 32\nSIA C11 H112 SING N N 33\nSIA N5 HN5 SING N N 34\nSIA O1B HO1B SING N N 35\nSIA O4 HO4 SING N N 37\nSIA O7 HO7 SING N N 38\nSIA O8 HO8 SING N N 39\nSIA O9 HO9 SING N N
40\n#\nloop_\n_pdbx_chem_comp_descriptor.comp_id\n_pdbx_chem_comp_descriptor.type\n_pdbx_chem_comp_descriptor.program\n_pdbx_chem_comp_descriptor.program_version\n_pdbx_chem_comp_descriptor.descriptor\nSIA SMILES ACDLabs 10.04 'O=C(O)C1(O)OC(C(O)C(O)CO)C(NC(=O)C)C(O)C1'\nSIA SMILES_CANONICAL CACTVS 3.341
'CC(=O)N[C@@H]1[C@@H](O)C[C@@](O)(O[C@H]1[C@H](O)[C@H](O)CO)C(O)=O'\nS

IA SMILES CACTVS 3.341
'CC(=O)N[CH]1[CH](O)C[C](O)(O[CH]1[CH](O)[CH](O)CO)C(O)=O'\nSIA
SMILES_CANONICAL 'OpenEye OEToolkits' 1.5.0
'CC(=O)N[C@@H]1[C@H](C[C@@](O[C@H]1[C@@H]([C@@H](CO)O)O)(C(=O)O)O)O'\n
SIA SMILES 'OpenEye OEToolkits' 1.5.0
'CC(=O)NC1C(CC(OC1C(C(CO)O)O)(C(=O)O)O)O'\nSIA InChI InChI 1.03
'InChI=1S/C11H19NO9/c1-4(14)12-7-5(15)2-11(20,10(18)19)21-
9(7)8(17)6(16)3-13/h5-9,13,15-17,20H,2-
3H2,1H3,(H,12,14)(H,18,19)/t5-,6+,7+,8+,9+,11+/m0/s1'\nSIA InChIKey
InChI 1.03 SQVRNKJHWKZAKO-YRMXFSIDSA-
N\n#\nloop_\n_pdbx_chem_comp_identifier.comp_id\n_pdbx_chem_comp_ident
ifier.type\n_pdbx_chem_comp_identifier.program\n_pdbx_chem_comp_identi
fier.program_version\n_pdbx_chem_comp_identifier.identifier\nSIA
'SYSTEMATIC NAME' ACDLabs 10.04 '5-(acetylamino)-3,5-dideoxy-D-
glycero-alpha-D-galacto-non-2-ulopyranosonic acid'\nSIA 'SYSTEMATIC
NAME' 'OpenEye OEToolkits' 1.5.0 '(2R,4S,5R,6R)-5-acetamido-2,4-
dihydroxy-6-[(1R,2R)-1,2,3-trihydroxypropyl]oxane-2-carboxylic
acid'\nSIA 'CONDENSED IUPAC CARBOHYDRATE SYMBOL' GMML 1.0
DNeup5Aca\nSIA 'COMMON NAME' GMML 1.0 'N-acetyl-a-D-neuraminic
acid'\nSIA 'IUPAC CARBOHYDRATE SYMBOL' PDB-CARE 1.0 a-D-Neup5Ac\nSIA
'SNFG CARBOHYDRATE SYMBOL' GMML 1.0
Neu5Ac\n#\nloop_\n_pdbx_chem_comp_feature.comp_id\n_pdbx_chem_comp_fea
ture.type\n_pdbx_chem_comp_feature.value\n_pdbx_chem_comp_feature.sour
ce\n_pdbx_chem_comp_feature.support\nSIA 'CARBOHYDRATE ISOMER' D
PDB ?\nSIA 'CARBOHYDRATE RING' pyranose PDB ?\nSIA 'CARBOHYDRATE
ANOMER' alpha PDB ?\nSIA 'CARBOHYDRATE PRIMARY CARBONYL GROUP' ketose
PDB ?\n#\nloop_\n_pdbx_chem_comp_audit.comp_id\n_pdbx_chem_comp_audit.
action_type\n_pdbx_chem_comp_audit.date\n_pdbx_chem_comp_audit.process
ing_site\nSIA 'Create component' 1999-07-08 EBI\nSIA 'Modify
descriptor' 2011-06-04 RCSB\nSIA 'Other modification' 2019-08-12
RCSB\nSIA 'Other modification' 2019-12-19 RCSB\nSIA 'Other
modification' 2020-07-03 RCSB\nSIA 'Modify name' 2020-07-17 RCSB\nSIA
'Modify synonyms' 2020-07-17 RCSB\nSIA 'Modify atom id' 2020-07-17
RCSB\nSIA 'Modify component atom id' 2020-07-17 RCSB\nSIA 'Modify PCM'
2024-09-27 PDBE\n#\n_pdbx_chem_comp_pcm.pcm_id
1\n_pdbx_chem_comp_pcm.comp_id
SIA\n_pdbx_chem_comp_pcm.modified_residue_id
THR\n_pdbx_chem_comp_pcm.type None\n_pdbx_chem_comp_pcm.category
Carbohydrate\n_pdbx_chem_comp_pcm.position 'Amino-acid side
chain'\n_pdbx_chem_comp_pcm.polypeptide_position 'Any
position'\n_pdbx_chem_comp_pcm.comp_id_linking_atom
C2\n_pdbx_chem_comp_pcm.modified_residue_id_linking_atom
OG1\n_pdbx_chem_comp_pcm.uniprot_specific_ptm_accession ?\n_pdbx_chem_
comp_pcm.uniprot_generic_ptm_accession ?\n#\n_pdbe_chem_comp_drugbank_
details.comp_id SIA\n_pdbe_chem_comp_drugbank_details.drugbank_id
DB03721\n_pdbe_chem_comp_drugbank_details.type 'small
molecule'\n_pdbe_chem_comp_drugbank_details.name 'N-acetyl-alpha-
neuraminic acid'\n_pdbe_chem_comp_drugbank_details.description\n'An N-
acyl derivative of neuraminic acid. N-acetylneuraminic acid occurs in
many polysaccharides, glycoproteins, and glycolipids in animals and
bacteria. (From Dorland, 28th ed,
p1518)'\n_pdbe_chem_comp_drugbank_details.cas_number 21646-00-

4\n_pdbe_chem_comp_drugbank_details.mechanism_of_action ?\n#\nloop_\n_pdbe_chem_comp_synonyms.comp_id\n_pdbe_chem_comp_synonyms.name\n_pdbe_chem_comp_synonyms.provenance\n_pdbe_chem_comp_synonyms.type\nSIA 'N-acetylneuraminic acid' wwPDB ?\nSIA 'sialic acid' wwPDB ?\nSIA 'alpha-sialic acid' wwPDB ?\nSIA 'O-SIALIC ACID' wwPDB ?\nSIA 'N-Acetyl-alpha-D-neuraminic acid' DrugBank ?\nSIA 'O-sialic acid' DrugBank ?\nSIA 'α-Neu5Ac' DrugBank ?\n#\n_pdbe_chem_comp_drugbank_classification.comp_id SIA\n_pdbe_chem_comp_drugbank_classification.drugbank_id DB03721\n_pdbe_chem_comp_drugbank_classification.parent 'N-acylneuraminic acids'\n_pdbe_chem_comp_drugbank_classification.kingdom 'Organic compounds'\n_pdbe_chem_comp_drugbank_classification.class 'Organooxygen compounds'\n_pdbe_chem_comp_drugbank_classification.superclass 'Organic oxygen compounds'\n_pdbe_chem_comp_drugbank_classification.description\n'This compound belongs to the class of organic compounds known as n-acylneuraminic acids. These are neuraminic acids carrying an N-acyl substituent.'\n#\nloop_\n_pdbe_chem_comp_drugbank_targets.comp_id\n_pdbe_chem_comp_drugbank_targets.drugbank_id\n_pdbe_chem_comp_drugbank_targets.name\n_pdbe_chem_comp_drugbank_targets.organism\n_pdbe_chem_comp_drugbank_targets.uniprot_id\n_pdbe_chem_comp_drugbank_targets.pharmacologically_active\n_pdbe_chem_comp_drugbank_targets.ordinal\nSIA DB03721 P-selectin Humans P16109 yes 1\nSIA DB03721 E-selectin Humans P16581 yes 2\nSIA DB03721 'Liver carboxylesterase 1' Humans P23141 yes 3\nSIA DB03721 '3-deoxy-manno-octulosonate cytidylyltransferase' 'Escherichia coli' P42216 unknown 4\nSIA DB03721 'Tetanus toxin' 'Clostridium tetani (strain Massachusetts / E88)' P04958 unknown 5\nSIA DB03721 'Cholera enterotoxin subunit B' 'Vibrio cholerae serotype O1 (strain ATCC 39315 / El Tor Inaba N16961)' P01556 unknown 6\nSIA DB03721 'Botulinum neurotoxin type B' 'Clostridium botulinum' P10844 unknown 7\nSIA DB03721 'Mannose-binding protein C' Humans P11226 unknown 8\nSIA DB03721 Lithostathine-1-alpha Humans P05451 unknown 9\nSIA DB03721 Endo-N-acetylneuraminidase 'Enterobacteria phage K1F' Q04830 unknown 10\nSIA DB03721 'Enterotoxin type B' 'Staphylococcus aureus' P01552 unknown 11\nSIA DB03721 Neuraminidase 'Influenza A virus (strain A/Tern/Australia/G70C/1975 H11N9)' P03472 unknown 12\nSIA DB03721 Hemagglutinin-neuraminidase NDV P32884 unknown 13\nSIA DB03721 Fiber 'Human adenovirus 19' Q64822 unknown 14\nSIA DB03721 Sialoadhesin Humans Q9BZZ2 unknown 15\nSIA DB03721 Zinc-alpha-2-glycoprotein Humans P25311 unknown 16\nSIA DB03721 'Capsid protein VP1' MPyV P49302 unknown 17\nSIA DB03721 Fiber 'Human adenovirus D37' Q64823 unknown 18\n#\nloop_\n_software.name\n_software.version\n_software.description\nrdkit 2023.09.6 'Core functionality.'\npdbeccdutils 0.8.6 'Wrapper to provide 2D templates and molecular fragments.'\n#\nloop_\n_pdbe_chem_comp_atom_depiction.comp_id\n_pdbe_chem_comp_atom_depiction.atom_id\n_pdbe_chem_comp_atom_depiction.element\n_pdbe_chem_comp_atom_depiction.model_Cartn_x\n_pdbe_chem_comp_atom_depiction.model_Cartn_y\n_pdbe_chem_comp_atom_depiction.pdbx_ordinal\nSIA C1 C 5.654 -3.375 1\nSIA C2 C 6.404 -2.076 2\nSIA C3 C 5.104 -1.326 3\nSIA C4 C 5.104 0.174 4\nSIA C5 C 6.404 0.924 5\nSIA C6 C

7.702 0.174 6\nSIA C7 C 9.002 0.924 7\nSIA C8 C 10.301 0.174 8\nSIA C9
C 11.600 0.924 9\nSIA C10 C 5.104 3.174 10\nSIA C11 C 3.805 2.424
11\nSIA N5 N 6.404 2.424 12\nSIA O1A O 4.154 -3.375 13\nSIA O1B O
6.404 -4.674 14\nSIA O4 O 3.805 0.924 16\nSIA O6 O 7.702 -1.326
17\nSIA O7 O 9.002 2.424 18\nSIA O8 O 10.301 -1.326 19\nSIA O9 O
12.899 0.174 20\nSIA O10 O 5.104 4.674
21\n#\nloop_\n_pdbe_chem_comp_bond_depiction.comp_id\n_pdbe_chem_comp_
bond_depiction.atom_id_1\n_pdbe_chem_comp_bond_depiction.atom_id_2\n_p
dbe_chem_comp_bond_depiction.value_order\n_pdbe_chem_comp_bond_depicti
on.bond_dir\n_pdbe_chem_comp_bond_depiction.pdbx_ordinal\nSIA C1 C2
SINGLE NONE 1\nSIA C1 O1A DOUBLE NONE 2\nSIA C1 O1B SINGLE NONE 3\nSIA
C2 C3 SINGLE NONE 4\nSIA C2 O6 SINGLE NONE 6\nSIA C3 C4 SINGLE NONE
7\nSIA C4 C5 SINGLE NONE 8\nSIA C4 O4 SINGLE BEGINDASH 9\nSIA C5 C6
SINGLE NONE 10\nSIA C5 N5 SINGLE BEGINWEDGE 11\nSIA C6 C7 SINGLE NONE
12\nSIA C6 O6 SINGLE BEGINDASH 13\nSIA C7 C8 SINGLE NONE 14\nSIA C7 O7
SINGLE BEGINDASH 15\nSIA C8 C9 SINGLE NONE 16\nSIA C8 O8 SINGLE
BEGINWEDGE 17\nSIA C9 O9 SINGLE NONE 18\nSIA C10 C11 SINGLE NONE
19\nSIA C10 N5 SINGLE NONE 20\nSIA C10 O10 DOUBLE NONE
21\n#\nloop_\n_pdbe_chem_comp_substructure.comp_id\n_pdbe_chem_comp_su
bstructure.substructure_name\n_pdbe_chem_comp_substructure.id\n_pdbe_c
hem_comp_substructure.substructure_type\n_pdbe_chem_comp_substructure.
substructure_smiles\n_pdbe_chem_comp_substructure.substructure_inchis\
n_pdbe_chem_comp_substructure.substructure_inchikeys\nSIA
MurckoScaffold S1 scaffold C1CCOCC1 InChI=1S/C5H10O/c1-2-4-6-5-3-1/h1-
5H2 DHXVGJBLRPWPCS-UHFFFAOYSA-N\nSIA amide F1 fragment CC(N)=O
InChI=1S/C2H5NO/c1-2(3)4/h1H3,(H2,3,4) DLFVBJFMPXGRIB-UHFFFAOYSA-
N\nSIA pyranose F2 fragment OC1CCCCO1 InChI=1S/C5H10O2/c6-5-3-1-2-4-7-
5/h5-6H,1-4H2 CELWCAITJAEQNL-UHFFFAOYSA-
N\n#\nloop_\n_pdbe_chem_comp_substructure_mapping.comp_id\n_pdbe_chem_
comp_substructure_mapping.atom_id\n_pdbe_chem_comp_substructure_mappin
g.substructure_id\n_pdbe_chem_comp_substructure_mapping.substructure_o
rdinal\nSIA C2 S1 1\nSIA C3 S1 1\nSIA C4 S1 1\nSIA C5 S1 1\nSIA C6 S1
1\nSIA O6 S1 1\nSIA N5 F1 1\nSIA C10 F1 1\nSIA O10 F1 1\nSIA C11 F1
1\nSIA C5 F2 1\nSIA C6 F2 1\nSIA O6 F2 1\nSIA C2 F2 1\nSIA C3 F2
1\nSIA C4 F2 1\n#\n_pdbe_chem_comp_rdkit_properties.comp_id
SIA\n_pdbe_chem_comp_rdkit_properties.exactmw
309.106\n_pdbe_chem_comp_rdkit_properties.amw
309.271\n_pdbe_chem_comp_rdkit_properties.lipinskiHBA
10\n_pdbe_chem_comp_rdkit_properties.lipinskiHBD
7\n_pdbe_chem_comp_rdkit_properties.NumRotatableBonds
11\n_pdbe_chem_comp_rdkit_properties.NumHBD
7\n_pdbe_chem_comp_rdkit_properties.NumHBA
9\n_pdbe_chem_comp_rdkit_properties.NumHeavyAtoms
21\n_pdbe_chem_comp_rdkit_properties.NumAtoms
40\n_pdbe_chem_comp_rdkit_properties.NumHeteroatoms
10\n_pdbe_chem_comp_rdkit_properties.NumAmideBonds
1\n_pdbe_chem_comp_rdkit_properties.FractionCSP3
0.818\n_pdbe_chem_comp_rdkit_properties.NumRings
1\n_pdbe_chem_comp_rdkit_properties.NumAromaticRings
0\n_pdbe_chem_comp_rdkit_properties.NumAliphaticRings
1\n_pdbe_chem_comp_rdkit_properties.NumSaturatedRings
1\n_pdbe_chem_comp_rdkit_properties.NumHeterocycles

1\n_pdbe_chem_comp_rdkit_properties.NumAromaticHeterocycles
0\n_pdbe_chem_comp_rdkit_properties.NumSaturatedHeterocycles
1\n_pdbe_chem_comp_rdkit_properties.NumAliphaticHeterocycles
1\n_pdbe_chem_comp_rdkit_properties.NumSpiroAtoms
0\n_pdbe_chem_comp_rdkit_properties.NumBridgeheadAtoms
0\n_pdbe_chem_comp_rdkit_properties.NumAtomStereoCenters
6\n_pdbe_chem_comp_rdkit_properties.NumUnspecifiedAtomStereoCenters
0\n_pdbe_chem_comp_rdkit_properties.labuteASA
146.407\n_pdbe_chem_comp_rdkit_properties.tpsa
176.780\n_pdbe_chem_comp_rdkit_properties.CrippenClogP -
3.872\n_pdbe_chem_comp_rdkit_properties.CrippenMR
64.787\n_pdbe_chem_comp_rdkit_properties.chi0v
9.621\n_pdbe_chem_comp_rdkit_properties.chi1v
4.738\n_pdbe_chem_comp_rdkit_properties.chi2v
1.941\n_pdbe_chem_comp_rdkit_properties.chi3v
1.941\n_pdbe_chem_comp_rdkit_properties.chi4v
1.128\n_pdbe_chem_comp_rdkit_properties.chi0n
28.621\n_pdbe_chem_comp_rdkit_properties.chi1n
13.635\n_pdbe_chem_comp_rdkit_properties.chi2n
1.941\n_pdbe_chem_comp_rdkit_properties.chi3n
1.941\n_pdbe_chem_comp_rdkit_properties.chi4n
1.128\n_pdbe_chem_comp_rdkit_properties.hallKierAlpha -
1.300\n_pdbe_chem_comp_rdkit_properties.kappa1
4.600\n_pdbe_chem_comp_rdkit_properties.kappa2
6.642\n_pdbe_chem_comp_rdkit_properties.kappa3
3.872\n_pdbe_chem_comp_rdkit_properties.Phi
1.455\n#\nloop_\n_pdbe_chem_comp_external_mappings.comp_id\n_pdbe_chem
_comp_external_mappings.source\n_pdbe_chem_comp_external_mappings.reso
urce\n_pdbe_chem_comp_external_mappings.resource_id\nSIA UniChem
ChEMBL CHEMBL1234621\nSIA UniChem DrugBank DB03721\nSIA UniChem ChEBI
49026\nSIA UniChem ZINC ZINC000004081651\nSIA UniChem fdasrs
04A90EXP8V\nSIA UniChem HMDB HMDB0000773\nSIA UniChem Nikkaji
J614.853K\nSIA UniChem MetaboLights MTBLC49026\nSIA UniChem BRENDA
141715\nSIA UniChem BRENDA 233672\nSIA UniChem BRENDA 6105\nSIA
UniChem BRENDA 84245\nSIA UniChem BRENDA 85625\nSIA UniChem 'Probes
And Drugs' PD041137\nSIA UniChem PubChem 444885\nSIA UniChem
eMolecules 474793\nSIA UniChem SureChEMBL SCHEMBL79085\nSIA UniChem
'PubChem TPHARMA' 14776495\nSIA UniChem 'PubChem TPHARMA'
15395566\n#\nloop_\n_pdbe_chem_comp_rdkit_conformer.comp_id\n_pdbe_che
m_comp_rdkit_conformer.atom_id\n_pdbe_chem_comp_rdkit_conformer.Cartn_
x_rdkit\n_pdbe_chem_comp_rdkit_conformer.Cartn_y_rdkit\n_pdbe_chem_com
p_rdkit_conformer.Cartn_z_rdkit\n_pdbe_chem_comp_rdkit_conformer.rdkit
_method\n_pdbe_chem_comp_rdkit_conformer.rdkit_ordinal\nSIA C1 -1.164
-2.811 1.058 ETKDGv3 1\nSIA C2 -1.666 -1.928 -0.070 ETKDGv3 2\nSIA C3
-2.639 -0.809 0.442 ETKDGv3 3\nSIA C4 -2.546 0.454 -0.428 ETKDGv3
4\nSIA C5 -1.079 0.960 -0.528 ETKDGv3 5\nSIA C6 -0.071 -0.181 -0.219
ETKDGv3 6\nSIA C7 1.341 0.133 -0.777 ETKDGv3 7\nSIA C8 2.378 -0.960 -
0.400 ETKDGv3 8\nSIA C9 3.769 -0.651 -0.979 ETKDGv3 9\nSIA C10 -0.223
3.295 0.052 ETKDGv3 10\nSIA C11 0.035 4.333 1.094 ETKDGv3 11\nSIA N5 -
0.868 2.066 0.414 ETKDGv3 12\nSIA O1A -0.936 -2.335 2.203 ETKDGv3
13\nSIA O1B -0.875 -4.149 0.806 ETKDGv3 14\nSIA O4 -3.104 0.224 -1.700
ETKDGv3 16\nSIA O6 -0.537 -1.404 -0.765 ETKDGv3 17\nSIA O7 1.298 0.320

-2.172 ETKDGv3 18\nSIA O8 2.484 -1.066 0.998 ETKDGv3 19\nSIA O9 4.246 0.587 -0.524 ETKDGv3 20\nSIA O10 0.157 3.497 -1.133 ETKDGv3 21\nSIA H32 -2.392 -0.510 1.484 ETKDGv3 22\nSIA H31 -3.691 -1.171 0.465 ETKDGv3 23\nSIA H4 -3.171 1.239 0.053 ETKDGv3 24\nSIA H5 -0.925 1.324 -1.568 ETKDGv3 25\nSIA H6 0.030 -0.270 0.887 ETKDGv3 26\nSIA H7 1.678 1.089 -0.320 ETKDGv3 27\nSIA H8 2.053 -1.935 -0.838 ETKDGv3 28\nSIA H92 3.719 -0.629 -2.088 ETKDGv3 29\nSIA H91 4.478 -1.464 -0.703 ETKDGv3 30\nSIA H111 0.843 3.984 1.770 ETKDGv3 31\nSIA H113 -0.888 4.508 1.686 ETKDGv3 32\nSIA H112 0.344 5.291 0.624 ETKDGv3 33\nSIA HN5 -1.113 1.913 1.419 ETKDGv3 34\nSIA HO1B -0.511 -4.749 1.536 ETKDGv3 35\nSIA HO4 -2.492 -0.372 -2.205 ETKDGv3 37\nSIA HO7 1.040 -0.545 -2.585 ETKDGv3 38\nSIA HO8 1.886 -1.804 1.285 ETKDGv3 39\nSIA HO9 4.592 0.441 0.395 ETKDGv3 40\n#\n"
}

Sup. Fig. 1c. G2S2 (α2,6-sia)

{
    "name": "g2s2f_66_userccd",
    "modelSeeds": [
      1
    ],
    "sequences": [
      {
        "ligand": {
          "ccdCodes": [
            "NAG", "NAG", "BMA", "MAN", "MAN", "NAG", "NAG", "GAL",
"GAL", "SIA-2", "SIA-2", "FUC"
          ],
          "id": "NG"
        }
      }
    ],
    "dialect": "alphafold3",
    "version": 2,
    "bondedAtomPairs": [
    [["NG",1,"O4"],["NG",2,"C1"]],
    [["NG",2,"O4"],["NG",3,"C1"]],
    [["NG",3,"O3"],["NG",4,"C1"]],
    [["NG",3,"O6"],["NG",5,"C1"]],
    [["NG",4,"O2"],["NG",6,"C1"]],
    [["NG",5,"O2"],["NG",7,"C1"]],
    [["NG",6,"O4"],["NG",8,"C1"]],
    [["NG",7,"O4"],["NG",9,"C1"]],
    [["NG",8,"O6"],["NG",10,"C2"]],
    [["NG",9,"O6"],["NG",11,"C2"]],
    [["NG",1,"O6"],["NG",12,"C1"]]
    ],
    "userCCD":"data_SIA-2\n#\n_chem_comp.id SIA\n_chem_comp.name 'N-acetyl-alpha-neuraminic acid'\n_chem_comp.type 'D-saccharide, alpha linking'\n_chem_comp.pdbx_type ATOMS\n_chem_comp.formula 'C11 H19 N O9'\n_chem_comp.mon_nstd_parent_comp_id ?\n_chem_comp.pdbx_synonyms 'N-acetylneuraminic acid; sialic acid; alpha-sialic acid; O-SIALIC ACID'\n_chem_comp.pdbx_formal_charge 0\n_chem_comp.pdbx_initial_date 1999-07-08\n_chem_comp.pdbx_modified_date 2024-09-27\n_chem_comp.pdbx_ambiguous_flag N\n_chem_comp.pdbx_release_status REL\n_chem_comp.pdbx_replaced_by ?\n_chem_comp.pdbx_replaces NAN\n_chem_comp.formula_weight 309.270\n_chem_comp.one_letter_code ?\n_chem_comp.three_letter_code SIA\n_chem_comp.pdbx_model_coordinates_details ?\n_chem_comp.pdbx_model_coordinates_missing_flag N\n_chem_comp.pdbx_ideal_coordinates_details ?\n_chem_comp.pdbx_ideal_coordinates_missing_flag N\n_chem_comp.pdbx_model_coordinates_db_code ?\n_chem_comp.pdbx_subcomponent_list ?\n_chem_comp.pdbx_processing_site EBI\n_chem_comp.pdbx_pcm Y\n#\nloop_\n_pdbx_chem_comp_synonyms.ordinal\n_pdbx_chem_comp_synonym

s.comp_id\n_pdbx_chem_comp_synonyms.name\n_pdbx_chem_comp_synonyms.pro venance\n_pdbx_chem_comp_synonyms.type\n1 SIA 'N-acetylneuraminic acid' PDB ?\n2 SIA 'sialic acid' PDB ?\n3 SIA 'alpha-sialic acid' PDB ?\n4 SIA 'O-SIALIC ACID'
PDB ?\n#\nloop_\n_chem_comp_atom.comp_id\n_chem_comp_atom.atom_id\n_ch em_comp_atom.alt_atom_id\n_chem_comp_atom.type_symbol\n_chem_comp_atom .charge\n_chem_comp_atom.pdbx_align\n_chem_comp_atom.pdbx_aromatic_fla g\n_chem_comp_atom.pdbx_leaving_atom_flag\n_chem_comp_atom.pdbx_stereo _config\n_chem_comp_atom.pdbx_backbone_atom_flag\n_chem_comp_atom.pdbx _n_terminal_atom_flag\n_chem_comp_atom.pdbx_c_terminal_atom_flag\n_che m_comp_atom.model_Cartn_x\n_chem_comp_atom.model_Cartn_y\n_chem_comp_a tom.model_Cartn_z\n_chem_comp_atom.pdbx_model_Cartn_x_ideal\n_chem_com p_atom.pdbx_model_Cartn_y_ideal\n_chem_comp_atom.pdbx_model_Cartn_z_id eal\n_chem_comp_atom.pdbx_component_atom_id\n_chem_comp_atom.pdbx_comp onent_comp_id\n_chem_comp_atom.pdbx_ordinal\nSIA C1 C1 C 0 1 N N N N N N -2.196 58.872 -5.981 -2.502 -0.832 0.174 C1 SIA 1\nSIA C2 C2 C 0 1 N N R N N N -1.870 58.021 -7.211 -2.171 0.628 0.342 C2 SIA 2\nSIA C3 C3 C 0 1 N N N N N N -0.844 56.899 -7.306 -1.789 0.898 1.800 C3 SIA 3\nSIA C4 C4 C 0 1 N N S N N N -1.157 55.904 -8.413 -0.586 0.023 2.171 C4 SIA 4\nSIA C5 C5 C 0 1 N N R N N N -2.015 56.516 -9.517 0.529 0.264 1.148 C5 SIA 5\nSIA C6 C6 C 0 1 N N R N N N -3.352 56.956 -8.912 -0.026 0.043 -0.259 C6 SIA 6\nSIA C7 C7 C 0 1 N N R N N N -4.224 57.698 -9.942 1.088 0.251 -1.286 C7 SIA 7\nSIA C8 C8 C 0 1 N N R N N N -5.571 58.131 -9.360 0.535 0.021 -2.694 C8 SIA 8\nSIA C9 C9 C 0 1 N N N N N N -6.601 58.674 -10.381 1.650 0.229 -3.721 C9 SIA 9\nSIA C10 C10 C 0 1 N N N N N N -1.897 55.374 -11.759 2.632 -0.329 2.226 C10 SIA 10\nSIA C11 C11 C 0 1 N N N N N N -2.200 54.057 -12.454 3.763 -1.292 2.478 C11 SIA 11\nSIA N5 N5 N 0 1 N N N N N N -2.202 55.444 -10.478 1.629 -0.671 1.394 N5 SIA 12\nSIA O1A O1A O 0 1 N N N N N N -1.289 58.815 -5.130 -2.191 -1.408 -0.841 O1A SIA 13\nSIA O1B O1B O 0 1 N N N N N N -3.210 59.504 -5.631 -3.141 -1.493 1.152 O1B SIA 14\nSIA O4 O4 O 0 1 N N N N N N 0.072 55.523 -8.986 -0.123 0.370 3.478 O4 SIA 16\nSIA O6 O6 O 0 1 N N N N N N -3.149 57.908 -7.847 -1.082 0.968 -0.513 O6 SIA 17\nSIA O7 O7 O 0 1 N N N N N N -3.594 58.883 -10.402 1.588 1.586 -1.183 O7 SIA 18\nSIA O8 O8 O 0 1 N N N N N N -6.119 56.946 -8.828 0.035 -1.313 -2.797 O8 SIA 19\nSIA O9 O9 O 0 1 N N N N N N -6.931 57.687 -11.346 1.133 0.014 -5.035 O9 SIA 20\nSIA O10 O10 O 0 1 N N N N N N -1.423 56.357 -12.331 2.624 0.753 2.772 O10 SIA 21\nSIA H32 H31 H 0 1 N N N N N N -0.702 56.484 -6.300 -2.631 0.655 2.448 H32 SIA 22\nSIA H31 H32 H 0 1 N N N N N N 0.120 57.408 -7.182 -1.526 1.949 1.919 H31 SIA 23\nSIA H4 H4 H 0 1 N N N N N N -1.651 55.060 -7.897 -0.878 -1.026 2.153 H4 SIA 24\nSIA H5 H5 H 0 1 N N N N N N -1.506 57.375 -9.979 0.893 1.287 1.240 H5 SIA 25\nSIA H6 H6 H 0 1 N N N N N N -3.850 56.075 -8.492 -0.408 -0.973 -0.341 H6 SIA 26\nSIA H7 H7 H 0 1 N N N N N N -4.339 57.176 -10.907 1.896 -0.454 -1.093 H7 SIA 27\nSIA H8 H8 H 0 1 N N N N N N -5.473 58.871 -8.553 -0.272 0.728 -2.887 H8 SIA 28\nSIA H92 H91 H 0 1 N N N N N N -6.054 59.459 -10.925 2.031 1.247 -3.642 H92 SIA 29\nSIA H91 H92 H 0 1 N N N N N N -7.587 59.029 -10.055 2.457 -0.476 -3.528 H91 SIA 30\nSIA H111 H111 H 0 0 N N N N N N -3.215 53.728 -12.207 4.474 -0.844 3.172 H111 SIA 31\nSIA H113 H112 H 0 0 N N N N N N -1.550 53.279 -12.033 3.368 -2.213 2.907 H113 SIA 32\nSIA H112 H113 H 0 0 N N N N N N -2.005 54.041 -13.531 4.266 -1.516 1.537 H112 SIA

33\nSIA HN5 HN5 H 0 1 N N N N N N -2.566 54.658 -10.003 1.635 -1.538 0.957 HN5 SIA 34\nSIA HO1B HOB1 H 0 0 N N N N N N -3.412 60.032 -4.867 -3.353 -2.430 1.044 HO1B SIA 35\nSIA HO4 HO4 H 0 1 N Y N N N N 0.427 54.801 -8.430 -0.854 0.203 4.087 HO4 SIA 37\nSIA HO7 HO7 H 0 1 N Y N N N N -3.109 58.884 -9.548 0.844 2.177 -1.360 HO7 SIA 38\nSIA HO8 HO8 H 0 1 N Y N N N N -7.071 57.051 -8.962 0.779 -1.904 -2.620 HO8 SIA 39\nSIA HO9 HO9 H 0 1 N Y N N N N -6.783 56.885 -10.808 1.866 0.155 -5.650 HO9 SIA
40\n#\nloop_\n_chem_comp_bond.comp_id\n_chem_comp_bond.atom_id_1\n_chem_comp_bond.atom_id_2\n_chem_comp_bond.value_order\n_chem_comp_bond.pdbx_aromatic_flag\n_chem_comp_bond.pdbx_stereo_config\n_chem_comp_bond.pdbx_ordinal\nSIA C1 C2 SING N N 1\nSIA C1 O1A DOUB N N 2\nSIA C1 O1B SING N N 3\nSIA C2 C3 SING N N 4\nSIA C2 O6 SING N N 6\nSIA C3 C4 SING N N 7\nSIA C3 H32 SING N N 8\nSIA C3 H31 SING N N 9\nSIA C4 C5 SING N N 10\nSIA C4 O4 SING N N 11\nSIA C4 H4 SING N N 12\nSIA C5 C6 SING N N 13\nSIA C5 N5 SING N N 14\nSIA C5 H5 SING N N 15\nSIA C6 C7 SING N N 16\nSIA C6 O6 SING N N 17\nSIA C6 H6 SING N N 18\nSIA C7 C8 SING N N 19\nSIA C7 O7 SING N N 20\nSIA C7 H7 SING N N 21\nSIA C8 C9 SING N N 22\nSIA C8 O8 SING N N 23\nSIA C8 H8 SING N N 24\nSIA C9 O9 SING N N 25\nSIA C9 H92 SING N N 26\nSIA C9 H91 SING N N 27\nSIA C10 C11 SING N N 28\nSIA C10 N5 SING N N 29\nSIA C10 O10 DOUB N N 30\nSIA C11 H111 SING N N 31\nSIA C11 H113 SING N N 32\nSIA C11 H112 SING N N 33\nSIA N5 HN5 SING N N 34\nSIA O1B HO1B SING N N 35\nSIA O4 HO4 SING N N 37\nSIA O7 HO7 SING N N 38\nSIA O8 HO8 SING N N 39\nSIA O9 HO9 SING N N
40\n#\nloop_\n_pdbx_chem_comp_descriptor.comp_id\n_pdbx_chem_comp_descriptor.type\n_pdbx_chem_comp_descriptor.program\n_pdbx_chem_comp_descriptor.program_version\n_pdbx_chem_comp_descriptor.descriptor\nSIA SMILES ACDLabs 10.04 'O=C(O)C1(O)OC(C(O)C(O)CO)C(NC(=O)C)C(O)C1'\nSIA SMILES_CANONICAL CACTVS 3.341
'CC(=O)N[C@@H]1[C@@H](O)C[C@@](O)(O[C@H]1[C@H](O)[C@H](O)CO)C(O)=O'\nSIA SMILES CACTVS 3.341
'CC(=O)N[CH]1[CH](O)C[C](O)(O[CH]1[CH](O)[CH](O)CO)C(O)=O'\nSIA SMILES_CANONICAL 'OpenEye OEToolkits' 1.5.0
'CC(=O)N[C@@H]1[C@H](C[C@@](O[C@H]1[C@@H]([C@@H](CO)O)O)(C(=O)O)O)O'\nSIA SMILES 'OpenEye OEToolkits' 1.5.0
'CC(=O)NC1C(CC(OC1C(C(CO)O)O)(C(=O)O)O)O'\nSIA InChI InChI 1.03
'InChI=1S/C11H19NO9/c1-4(14)12-7-5(15)2-11(20,10(18)19)21-9(7)8(17)6(16)3-13/h5-9,13,15-17,20H,2-3H2,1H3,(H,12,14)(H,18,19)/t5-,6+,7+,8+,9+,11+/m0/s1'\nSIA InChIKey InChI 1.03 SQVRNKJHWKZAKO-YRMXFSIDSA-N\n#\nloop_\n_pdbx_chem_comp_identifier.comp_id\n_pdbx_chem_comp_identifier.type\n_pdbx_chem_comp_identifier.program\n_pdbx_chem_comp_identifier.program_version\n_pdbx_chem_comp_identifier.identifier\nSIA 'SYSTEMATIC NAME' ACDLabs 10.04 '5-(acetylamino)-3,5-dideoxy-D-glycero-alpha-D-galacto-non-2-ulopyranosonic acid'\nSIA 'SYSTEMATIC NAME' 'OpenEye OEToolkits' 1.5.0 '(2R,4S,5R,6R)-5-acetamido-2,4-dihydroxy-6-[(1R,2R)-1,2,3-trihydroxypropyl]oxane-2-carboxylic acid'\nSIA 'CONDENSED IUPAC CARBOHYDRATE SYMBOL' GMML 1.0 DNeup5Aca\nSIA 'COMMON NAME' GMML 1.0 'N-acetyl-a-D-neuraminic acid'\nSIA 'IUPAC CARBOHYDRATE SYMBOL' PDB-CARE 1.0 a-D-Neup5Ac\nSIA 'SNFG CARBOHYDRATE SYMBOL' GMML 1.0

Neu5Ac\n#\nloop_\n_pdbx_chem_comp_feature.comp_id\n_pdbx_chem_comp_feature.type\n_pdbx_chem_comp_feature.value\n_pdbx_chem_comp_feature.source\n_pdbx_chem_comp_feature.support\nSIA 'CARBOHYDRATE ISOMER' D PDB ?\nSIA 'CARBOHYDRATE RING' pyranose PDB ?\nSIA 'CARBOHYDRATE ANOMER' alpha PDB ?\nSIA 'CARBOHYDRATE PRIMARY CARBONYL GROUP' ketose PDB ?\n#\nloop_\n_pdbx_chem_comp_audit.comp_id\n_pdbx_chem_comp_audit.action_type\n_pdbx_chem_comp_audit.date\n_pdbx_chem_comp_audit.processing_site\nSIA 'Create component' 1999-07-08 EBI\nSIA 'Modify descriptor' 2011-06-04 RCSB\nSIA 'Other modification' 2019-08-12 RCSB\nSIA 'Other modification' 2019-12-19 RCSB\nSIA 'Other modification' 2020-07-03 RCSB\nSIA 'Modify name' 2020-07-17 RCSB\nSIA 'Modify synonyms' 2020-07-17 RCSB\nSIA 'Modify atom id' 2020-07-17 RCSB\nSIA 'Modify component atom id' 2020-07-17 RCSB\nSIA 'Modify PCM' 2024-09-27 PDBE\n#\n_pdbx_chem_comp_pcm.pcm_id 1\n_pdbx_chem_comp_pcm.comp_id SIA\n_pdbx_chem_comp_pcm.modified_residue_id THR\n_pdbx_chem_comp_pcm.type None\n_pdbx_chem_comp_pcm.category Carbohydrate\n_pdbx_chem_comp_pcm.position 'Amino-acid side chain'\n_pdbx_chem_comp_pcm.polypeptide_position 'Any position'\n_pdbx_chem_comp_pcm.comp_id_linking_atom C2\n_pdbx_chem_comp_pcm.modified_residue_id_linking_atom OG1\n_pdbx_chem_comp_pcm.uniprot_specific_ptm_accession ?\n_pdbx_chem_comp_pcm.uniprot_generic_ptm_accession ?\n#\n_pdbe_chem_comp_drugbank_details.comp_id SIA\n_pdbe_chem_comp_drugbank_details.drugbank_id DB03721\n_pdbe_chem_comp_drugbank_details.type 'small molecule'\n_pdbe_chem_comp_drugbank_details.name 'N-acetyl-alpha-neuraminic acid'\n_pdbe_chem_comp_drugbank_details.description\n'An N-acyl derivative of neuraminic acid. N-acetylneuraminic acid occurs in many polysaccharides, glycoproteins, and glycolipids in animals and bacteria. (From Dorland, 28th ed, p1518)'\n_pdbe_chem_comp_drugbank_details.cas_number 21646-00-4\n_pdbe_chem_comp_drugbank_details.mechanism_of_action ?\n#\nloop_\n_pdbe_chem_comp_synonyms.comp_id\n_pdbe_chem_comp_synonyms.name\n_pdbe_chem_comp_synonyms.provenance\n_pdbe_chem_comp_synonyms.type\nSIA 'N-acetylneuraminic acid' wwPDB ?\nSIA 'sialic acid' wwPDB ?\nSIA 'alpha-sialic acid' wwPDB ?\nSIA 'O-SIALIC ACID' wwPDB ?\nSIA 'N-Acetyl-alpha-D-neuraminic acid' DrugBank ?\nSIA 'O-sialic acid' DrugBank ?\nSIA 'α-Neu5Ac' DrugBank ?\n#\n_pdbe_chem_comp_drugbank_classification.comp_id SIA\n_pdbe_chem_comp_drugbank_classification.drugbank_id DB03721\n_pdbe_chem_comp_drugbank_classification.parent 'N-acylneuraminic acids'\n_pdbe_chem_comp_drugbank_classification.kingdom 'Organic compounds'\n_pdbe_chem_comp_drugbank_classification.class 'Organooxygen compounds'\n_pdbe_chem_comp_drugbank_classification.superclass 'Organic oxygen compounds'\n_pdbe_chem_comp_drugbank_classification.description\n'This compound belongs to the class of organic compounds known as n-acylneuraminic acids. These are neuraminic acids carrying an N-acyl substituent.'\n#\nloop_\n_pdbe_chem_comp_drugbank_targets.comp_id\n_pdbe_chem_comp_drugbank_targets.drugbank_id\n_pdbe_chem_comp_drugbank_targets.name\n_pdbe_chem_comp_drugbank_targets.organism\n_pdbe_chem_comp

_drugbank_targets.uniprot_id\n_pdbe_chem_comp_drugbank_targets.pharmacologically_active\n_pdbe_chem_comp_drugbank_targets.ordinal\nSIA DB03721 P-selectin Humans P16109 yes 1\nSIA DB03721 E-selectin Humans P16581 yes 2\nSIA DB03721 'Liver carboxylesterase 1' Humans P23141 yes 3\nSIA DB03721 '3-deoxy-manno-octulosonate cytidylyltransferase' 'Escherichia coli' P42216 unknown 4\nSIA DB03721 'Tetanus toxin' 'Clostridium tetani (strain Massachusetts / E88)' P04958 unknown 5\nSIA DB03721 'Cholera enterotoxin subunit B' 'Vibrio cholerae serotype O1 (strain ATCC 39315 / El Tor Inaba N16961)' P01556 unknown 6\nSIA DB03721 'Botulinum neurotoxin type B' 'Clostridium botulinum' P10844 unknown 7\nSIA DB03721 'Mannose-binding protein C' Humans P11226 unknown 8\nSIA DB03721 Lithostathine-1-alpha Humans P05451 unknown 9\nSIA DB03721 Endo-N-acetylneuraminidase 'Enterobacteria phage K1F' Q04830 unknown 10\nSIA DB03721 'Enterotoxin type B' 'Staphylococcus aureus' P01552 unknown 11\nSIA DB03721 Neuraminidase 'Influenza A virus (strain A/Tern/Australia/G70C/1975 H11N9)' P03472 unknown 12\nSIA DB03721 Hemagglutinin-neuraminidase NDV P32884 unknown 13\nSIA DB03721 Fiber 'Human adenovirus 19' Q64822 unknown 14\nSIA DB03721 Sialoadhesin Humans Q9BZZ2 unknown 15\nSIA DB03721 Zinc-alpha-2-glycoprotein Humans P25311 unknown 16\nSIA DB03721 'Capsid protein VP1' MPyV P49302 unknown 17\nSIA DB03721 Fiber 'Human adenovirus D37' Q64823 unknown 18\n#\nloop_\n_software.name\n_software.version\n_software.description\nrdkit 2023.09.6 'Core functionality.'\npdbeccdutils 0.8.6 'Wrapper to provide 2D templates and molecular fragments.'\n#\nloop_\n_pdbe_chem_comp_atom_depiction.comp_id\n_pdbe_chem_comp_atom_depiction.atom_id\n_pdbe_chem_comp_atom_depiction.element\n_pdbe_chem_comp_atom_depiction.model_Cartn_x\n_pdbe_chem_comp_atom_depiction.model_Cartn_y\n_pdbe_chem_comp_atom_depiction.pdbx_ordinal\nSIA C1 C 5.654 -3.375 1\nSIA C2 C 6.404 -2.076 2\nSIA C3 C 5.104 -1.326 3\nSIA C4 C 5.104 0.174 4\nSIA C5 C 6.404 0.924 5\nSIA C6 C 7.702 0.174 6\nSIA C7 C 9.002 0.924 7\nSIA C8 C 10.301 0.174 8\nSIA C9 C 11.600 0.924 9\nSIA C10 C 5.104 3.174 10\nSIA C11 C 3.805 2.424 11\nSIA N5 N 6.404 2.424 12\nSIA O1A O 4.154 -3.375 13\nSIA O1B O 6.404 -4.674 14\nSIA O4 O 3.805 0.924 16\nSIA O6 O 7.702 -1.326 17\nSIA O7 O 9.002 2.424 18\nSIA O8 O 10.301 -1.326 19\nSIA O9 O 12.899 0.174 20\nSIA O10 O 5.104 4.674 21\n#\nloop_\n_pdbe_chem_comp_bond_depiction.comp_id\n_pdbe_chem_comp_bond_depiction.atom_id_1\n_pdbe_chem_comp_bond_depiction.atom_id_2\n_pdbe_chem_comp_bond_depiction.value_order\n_pdbe_chem_comp_bond_depiction.bond_dir\n_pdbe_chem_comp_bond_depiction.pdbx_ordinal\nSIA C1 C2 SINGLE NONE 1\nSIA C1 O1A DOUBLE NONE 2\nSIA C1 O1B SINGLE NONE 3\nSIA C2 C3 SINGLE NONE 4\nSIA C2 O6 SINGLE NONE 6\nSIA C3 C4 SINGLE NONE 7\nSIA C4 C5 SINGLE NONE 8\nSIA C4 O4 SINGLE BEGINDASH 9\nSIA C5 C6 SINGLE NONE 10\nSIA C5 N5 SINGLE BEGINWEDGE 11\nSIA C6 C7 SINGLE NONE 12\nSIA C6 O6 SINGLE BEGINDASH 13\nSIA C7 C8 SINGLE NONE 14\nSIA C7 O7 SINGLE BEGINDASH 15\nSIA C8 C9 SINGLE NONE 16\nSIA C8 O8 SINGLE BEGINWEDGE 17\nSIA C9 O9 SINGLE NONE 18\nSIA C10 C11 SINGLE NONE 19\nSIA C10 N5 SINGLE NONE 20\nSIA C10 O10 DOUBLE NONE 21\n#\nloop_\n_pdbe_chem_comp_substructure.comp_id\n_pdbe_chem_comp_substructure.substructure_name\n_pdbe_chem_comp_substructure.id\n_pdbe_chem_comp_substructure.substructure_type\n_pdbe_chem_comp_substructure.

substructure_smiles\n_pdbe_chem_comp_substructure.substructure_inchis\
n_pdbe_chem_comp_substructure.substructure_inchikeys\nSIA
MurckoScaffold S1 scaffold C1CCOCC1 InChI=1S/C5H10O/c1-2-4-6-5-3-1/h1-
5H2 DHXVGJBLRPWPCS-UHFFFAOYSA-N\nSIA amide F1 fragment CC(N)=O
InChI=1S/C2H5NO/c1-2(3)4/h1H3,(H2,3,4) DLFVBJFMPXGRIB-UHFFFAOYSA-
N\nSIA pyranose F2 fragment OC1CCCCO1 InChI=1S/C5H10O2/c6-5-3-1-2-4-7-
5/h5-6H,1-4H2 CELWCAITJAEQNL-UHFFFAOYSA-
N\n#\nloop_\n_pdbe_chem_comp_substructure_mapping.comp_id\n_pdbe_chem_
comp_substructure_mapping.atom_id\n_pdbe_chem_comp_substructure_mappin
g.substructure_id\n_pdbe_chem_comp_substructure_mapping.substructure_o
rdinal\nSIA C2 S1 1\nSIA C3 S1 1\nSIA C4 S1 1\nSIA C5 S1 1\nSIA C6 S1
1\nSIA O6 S1 1\nSIA N5 F1 1\nSIA C10 F1 1\nSIA O10 F1 1\nSIA C11 F1
1\nSIA C5 F2 1\nSIA C6 F2 1\nSIA O6 F2 1\nSIA C2 F2 1\nSIA C3 F2
1\nSIA C4 F2 1\n#\n_pdbe_chem_comp_rdkit_properties.comp_id
SIA\n_pdbe_chem_comp_rdkit_properties.exactmw
309.106\n_pdbe_chem_comp_rdkit_properties.amw
309.271\n_pdbe_chem_comp_rdkit_properties.lipinskiHBA
10\n_pdbe_chem_comp_rdkit_properties.lipinskiHBD
7\n_pdbe_chem_comp_rdkit_properties.NumRotatableBonds
11\n_pdbe_chem_comp_rdkit_properties.NumHBD
7\n_pdbe_chem_comp_rdkit_properties.NumHBA
9\n_pdbe_chem_comp_rdkit_properties.NumHeavyAtoms
21\n_pdbe_chem_comp_rdkit_properties.NumAtoms
40\n_pdbe_chem_comp_rdkit_properties.NumHeteroatoms
10\n_pdbe_chem_comp_rdkit_properties.NumAmideBonds
1\n_pdbe_chem_comp_rdkit_properties.FractionCSP3
0.818\n_pdbe_chem_comp_rdkit_properties.NumRings
1\n_pdbe_chem_comp_rdkit_properties.NumAromaticRings
0\n_pdbe_chem_comp_rdkit_properties.NumAliphaticRings
1\n_pdbe_chem_comp_rdkit_properties.NumSaturatedRings
1\n_pdbe_chem_comp_rdkit_properties.NumHeterocycles
1\n_pdbe_chem_comp_rdkit_properties.NumAromaticHeterocycles
0\n_pdbe_chem_comp_rdkit_properties.NumSaturatedHeterocycles
1\n_pdbe_chem_comp_rdkit_properties.NumAliphaticHeterocycles
1\n_pdbe_chem_comp_rdkit_properties.NumSpiroAtoms
0\n_pdbe_chem_comp_rdkit_properties.NumBridgeheadAtoms
0\n_pdbe_chem_comp_rdkit_properties.NumAtomStereoCenters
6\n_pdbe_chem_comp_rdkit_properties.NumUnspecifiedAtomStereoCenters
0\n_pdbe_chem_comp_rdkit_properties.labuteASA
146.407\n_pdbe_chem_comp_rdkit_properties.tpsa
176.780\n_pdbe_chem_comp_rdkit_properties.CrippenClogP -
3.872\n_pdbe_chem_comp_rdkit_properties.CrippenMR
64.787\n_pdbe_chem_comp_rdkit_properties.chi0v
9.621\n_pdbe_chem_comp_rdkit_properties.chi1v
4.738\n_pdbe_chem_comp_rdkit_properties.chi2v
1.941\n_pdbe_chem_comp_rdkit_properties.chi3v
1.941\n_pdbe_chem_comp_rdkit_properties.chi4v
1.128\n_pdbe_chem_comp_rdkit_properties.chi0n
28.621\n_pdbe_chem_comp_rdkit_properties.chi1n
13.635\n_pdbe_chem_comp_rdkit_properties.chi2n
1.941\n_pdbe_chem_comp_rdkit_properties.chi3n
1.941\n_pdbe_chem_comp_rdkit_properties.chi4n

1.128\n_pdbe_chem_comp_rdkit_properties.hallKierAlpha -
1.300\n_pdbe_chem_comp_rdkit_properties.kappa1
4.600\n_pdbe_chem_comp_rdkit_properties.kappa2
6.642\n_pdbe_chem_comp_rdkit_properties.kappa3
3.872\n_pdbe_chem_comp_rdkit_properties.Phi
1.455\n#\nloop_\n_pdbe_chem_comp_external_mappings.comp_id\n_pdbe_chem
_comp_external_mappings.source\n_pdbe_chem_comp_external_mappings.reso
urce\n_pdbe_chem_comp_external_mappings.resource_id\nSIA UniChem
ChEMBL CHEMBL1234621\nSIA UniChem DrugBank DB03721\nSIA UniChem ChEBI
49026\nSIA UniChem ZINC ZINC000004081651\nSIA UniChem fdasrs
04A90EXP8V\nSIA UniChem HMDB HMDB0000773\nSIA UniChem Nikkaji
J614.853K\nSIA UniChem MetaboLights MTBLC49026\nSIA UniChem BRENDA
141715\nSIA UniChem BRENDA 233672\nSIA UniChem BRENDA 6105\nSIA
UniChem BRENDA 84245\nSIA UniChem BRENDA 85625\nSIA UniChem 'Probes
And Drugs' PD041137\nSIA UniChem eMolecules 474793\nSIA UniChem
SureChEMBL SCHEMBL79085\nSIA UniChem 'PubChem TPHARMA' 14776495\nSIA
UniChem 'PubChem TPHARMA' 15395566\nSIA UniChem PubChem
444885\n#\nloop_\n_pdbe_chem_comp_rdkit_conformer.comp_id\n_pdbe_chem_
comp_rdkit_conformer.atom_id\n_pdbe_chem_comp_rdkit_conformer.Cartn_x_
rdkit\n_pdbe_chem_comp_rdkit_conformer.Cartn_y_rdkit\n_pdbe_chem_comp_
rdkit_conformer.Cartn_z_rdkit\n_pdbe_chem_comp_rdkit_conformer.rdkit_m
ethod\n_pdbe_chem_comp_rdkit_conformer.rdkit_ordinal\nSIA C1 0.388
3.628 -0.366 ETKDGv3 1\nSIA C2 -0.221 2.365 0.190 ETKDGv3 2\nSIA C3 -
1.479 2.016 -0.650 ETKDGv3 3\nSIA C4 -2.132 0.712 -0.164 ETKDGv3
4\nSIA C5 -1.083 -0.261 0.426 ETKDGv3 5\nSIA C6 0.326 0.037 -0.160
ETKDGv3 6\nSIA C7 1.380 -0.998 0.336 ETKDGv3 7\nSIA C8 2.814 -0.780 -
0.221 ETKDGv3 8\nSIA C9 2.868 -0.877 -1.754 ETKDGv3 9\nSIA C10 -2.109
-2.492 1.110 ETKDGv3 10\nSIA C11 -2.547 -3.866 0.723 ETKDGv3 11\nSIA
N5 -1.485 -1.642 0.138 ETKDGv3 12\nSIA O1A 1.208 3.566 -1.321 ETKDGv3
13\nSIA O1B 0.005 4.872 0.130 ETKDGv3 14\nSIA O4 -3.107 1.009 0.803
ETKDGv3 16\nSIA O6 0.783 1.351 0.151 ETKDGv3 17\nSIA O7 1.411 -1.059
1.742 ETKDGv3 18\nSIA O8 3.333 0.464 0.180 ETKDGv3 19\nSIA O9 4.199 -
0.882 -2.194 ETKDGv3 20\nSIA O10 -2.315 -2.081 2.284 ETKDGv3 21\nSIA
H32 -2.218 2.848 -0.619 ETKDGv3 22\nSIA H31 -1.178 1.894 -1.715
ETKDGv3 23\nSIA H4 -2.627 0.248 -1.051 ETKDGv3 24\nSIA H5 -1.023 -
0.102 1.526 ETKDGv3 25\nSIA H6 0.256 -0.059 -1.265 ETKDGv3 26\nSIA H7
1.060 -2.002 -0.019 ETKDGv3 27\nSIA H8 3.455 -1.602 0.181 ETKDGv3
28\nSIA H92 2.346 -1.796 -2.106 ETKDGv3 29\nSIA H91 2.360 -0.000 -
2.207 ETKDGv3 30\nSIA H111 -2.829 -4.455 1.622 ETKDGv3 31\nSIA H113 -
1.720 -4.389 0.199 ETKDGv3 32\nSIA H112 -3.423 -3.797 0.045 ETKDGv3
33\nSIA HN5 -1.408 -1.987 -0.846 ETKDGv3 34\nSIA HO1B 0.393 5.726 -
0.253 ETKDGv3 35\nSIA HO4 -3.684 0.206 0.883 ETKDGv3 37\nSIA HO7 1.603
-0.147 2.082 ETKDGv3 38\nSIA HO8 3.744 0.336 1.075 ETKDGv3 39\nSIA HO9
4.550 -1.796 -2.032 ETKDGv3 40\n#\n"
}

Sup. Fig. 2b. A1


```
{
    "name": "a1",
    "modelSeeds": [
      1
    ],
    "sequences": [
      {
        "ligand": {
          "ccdCodes": [
            "NAG", "NAG", "BMA", "MAN", "MAN", "NAG"
          ],
          "id": "NG"
        }
      }
    ],
    "dialect": "alphafold3",
    "version": 2,
    "bondedAtomPairs": [
    [["NG",1,"O4"],["NG",2,"C1"]],
    [["NG",2,"O4"],["NG",3,"C1"]],
    [["NG",3,"O3"],["NG",4,"C1"]],
    [["NG",3,"O6"],["NG",5,"C1"]],
    [["NG",4,"O2"],["NG",6,"C1"]]
    ]
  }
```

Sup. Fig. 2c. HsMGAT2 complexed with Mn^{2+}, UDP-GlcNAc and A1


```
{
    "name": "mgat2_mn_ud1_a1",
    "modelSeeds": [
      1
    ],
    "sequences": [
      {
        "protein": {
          "sequence":
"MRFRIYKRKVLILTLVVAACGFVLWSSNGRQRKNEALAPPLLDAEPARGAGGRGGDHPSVAVGIRRVSN
VSAASLVPAVPQPEADNLTLRYRSLVYQLNFDQTLRNVDKAGTWAPRELVLVVQVHNRPEYLRLLLDSLR
KAQGIDNVLVIFSHDFWSTEINQLIAGVNFCPVLQVFFPFSIQLYPNEFPGSDPRDCPRDLPKNAALKLG
CINAEYPDSFGHYREAKFSQTKHHWWWKLHFVWERVKILRDYAGLILFLEEDHYLAPDFYHVFKKMWKLK
QQECPECDVLSLGTYSASRSFYGMADKVDVKTWKSTEHNMGLALTRNAYQKLIECTDTFCTYDDYNWDWT
LQYLTVSCLPKFWKVLVPQIPRIFHAGDCGMHHKKTCRPSTQSAQIESLLNNNKQYMFPETLTISEKFTV
VAISPPRKNGGWGDIRDHELCKSYRRLQ",
          "id": "MGAT"
        }
      },
      {
        "ligand": {
          "ccdCodes": [
            "MN"
          ],
          "id": "MN"
        }
      },
      {
        "ligand": {
          "ccdCodes": [
            "UD1"
          ],
          "id": "UDPGLCNAC"
        }
      },
      {
        "ligand": {
          "ccdCodes": [
            "NAG", "NAG", "BMA", "MAN", "MAN", "NAG"
          ],
          "id": "NG"
        }
      }
    ],
    "dialect": "alphafold3",
    "version": 2,
    "bondedAtomPairs": [
    [["NG",1,"O4"],["NG",2,"C1"]],
    [["NG",2,"O4"],["NG",3,"C1"]],
    [["NG",3,"O3"],["NG",4,"C1"]],
    [["NG",3,"O6"],["NG",5,"C1"]],
```

```
    [["NG",4,"O2"],["NG",6,"C1"]]
    ]
}
```

Sup. Fig. 2f. HsEPO carrying A1


```
{
    "name": "epo_a1",
    "modelSeeds": [
      1
    ],
    "sequences": [
      {
        "protein": {
          "sequence":
"MGVHECPAWLWLLLSLLSLPLGLPVLGAPPRLICDSRVLERYLLEAKEAENITTGCAEHCSLNENITVP
DTKVNFYAWKRMEVGQQAVEVWQGLALLSEAVLRGQALLVNSSQPWEPLQLHVDKAVSGLRSLTTLLRAL
GAQKEAISPPDAASAAPLRTITADTFRKLFRVYSNFLRGKLKLYTGEACRTGDR",
          "id": "EPO"
        }
      },
      {
        "ligand": {
          "ccdCodes": [
            "NAG", "NAG", "BMA", "MAN", "MAN", "NAG"
          ],
          "id": "NG"
        }
      }
    ],
    "dialect": "alphafold3",
    "version": 2,
    "bondedAtomPairs": [
    [["EPO",51,"ND2"],["NG",1,"C1"]],
    [["NG",1,"O4"],["NG",2,"C1"]],
    [["NG",2,"O4"],["NG",3,"C1"]],
    [["NG",3,"O3"],["NG",4,"C1"]],
    [["NG",3,"O6"],["NG",5,"C1"]],
    [["NG",4,"O2"],["NG",6,"C1"]]
    ]
  }
```

Sup. Fig. 2g. HsMGAT2 complexed with Mn^{2+}, UDP-GlcNAc and HsEPO carrying A1


```
{
    "name": "mgat2_mn_ud1_epo_a1",
    "modelSeeds": [
      1
    ],
    "sequences": [
      {
        "protein": {
          "sequence":
"MRFRIYKRKVLILTLVVAACGFVLWSSNGRQRKNEALAPPLLDAEPARGAGGRGGDHPSVAVGIRRVSN
VSAASLVPAVPQPEADNLTLRYRSLVYQLNFDQTLRNVDKAGTWAPRELVLVVQVHNRPEYLRLLLDSLR
KAQGIDNVLVIFSHDFWSTEINQLIAGVNFCPVLQVFFPFSIQLYPNEFPGSDPRDCPRDLPKNAALKLG
CINAEYPDSFGHYREAKFSQTKHHWWWKLHFVWERVKILRDYAGLILFLEEDHYLAPDFYHVFKKMWKLK
QQECPECDVLSLGTYSASRSFYGMADKVDVKTWKSTEHNMGLALTRNAYQKLIECTDTFCTYDDYNWDWT
LQYLTVSCLPKFWKVLVPQIPRIFHAGDCGMHHKKTCRPSTQSAQIESLLNNNKQYMFPETLTISEKFTV
VAISPPRKNGGWGDIRDHELCKSYRRLQ",
          "id": "MGAT"
        }
      },
      {
        "ligand": {
          "ccdCodes": [
            "MN"
          ],
          "id": "MN"
        }
      },
      {
        "ligand": {
          "ccdCodes": [
            "UD1"
          ],
          "id": "UDPGLCNAC"
        }
      },
      {
        "protein": {
          "sequence":
"MGVHECPAWLWLLLSLLSLPLGLPVLGAPPRLICDSRVLERYLLEAKEAENITTGCAEHCSLNENITVP
DTKVNFYAWKRMEVGQQAVEVWQGLALLSEAVLRGQALLVNSSQPWEPLQLHVDKAVSGLRSLTTLLRAL
GAQKEAISPPDAASAAPLRTITADTFRKLFRVYSNFLRGKLKLYTGEACRTGDR",
          "id": "EPO"
        }
      },
      {
        "ligand": {
          "ccdCodes": [
            "NAG", "NAG", "BMA", "MAN", "MAN", "NAG"
          ],
          "id": "NG"
```

```
      }
    }
  ],
  "dialect": "alphafold3",
  "version": 2,
  "bondedAtomPairs": [
  [["EPO",51,"ND2"],["NG",1,"C1"]],
  [["NG",1,"O4"],["NG",2,"C1"]],
  [["NG",2,"O4"],["NG",3,"C1"]],
  [["NG",3,"O3"],["NG",4,"C1"]],
  [["NG",3,"O6"],["NG",5,"C1"]],
  [["NG",4,"O2"],["NG",6,"C1"]]
  ]
}
```

Sup. Fig. 3b. G2F


```
{
    "name": "g2f",
    "modelSeeds": [
      1
    ],
    "sequences": [
      {
        "ligand": {
          "ccdCodes": [
            "NAG", "NAG", "BMA", "MAN", "MAN", "NAG", "NAG", "GAL",
"GAL", "FUC"
          ],
          "id": "NG"
        }
      }
    ],
    "dialect": "alphafold3",
    "version": 2,
    "bondedAtomPairs": [
    [["NG",1,"O4"],["NG",2,"C1"]],
    [["NG",2,"O4"],["NG",3,"C1"]],
    [["NG",3,"O3"],["NG",4,"C1"]],
    [["NG",3,"O6"],["NG",5,"C1"]],
    [["NG",4,"O2"],["NG",6,"C1"]],
    [["NG",5,"O2"],["NG",7,"C1"]],
    [["NG",6,"O4"],["NG",8,"C1"]],
    [["NG",7,"O4"],["NG",9,"C1"]],
    [["NG",1,"O6"],["NG",10,"C1"]]
    ]
  }
```

Sup. Fig. 3c. *N*-glycosylated HsST6GAL1 complexed with CMP-Sia and G2F


```
{
    "name": "st6gal1_ng_ncc_g2f",
    "modelSeeds": [
      1
    ],
    "sequences": [
      {
        "protein": {
          "sequence":
"MIHTNLKKKFSCCVLVFLLFAVICVWKEKKKGSYYDSFKLQTKEFQVLKSLGKLAMGSDSQSVSSSSTQ
DPHRGRQTLGSLRGLAKAKPEASFQVWNKDSSSKNLIPRLQKIWKNYLSMNKYKVSYKGPGPGIKFSAEA
LRCHLRDHVNVSMVEVTDFPFNTSEWEGYLPKESIRTKAGPWGRCAVVSSAGSLKSSQLGREIDDHDAVL
RFNGAPTANFQQDVGTKTTIRLMNSQLVTTEKRFLKDSLYNEGILIVWDPSVYHSDIPKWYQNPDYNFFN
NYKTYRKLHPNQPFYILKPQMPWELWDILQEISPEEIQPNPPSSGMLGIIIMMTLCDQVDIYEFLPSKRK
TDVCYYYQKFFDSACTMGAYHPLLYEKNLVKHLNQGTDEDIYLLGKATLPGFRTIHC",
          "id": "STGAL"
        }
      },
      {
        "ligand": {
          "ccdCodes": [
            "NCC"
          ],
          "id": "CMPSIA"
        }
      },
      {
        "ligand": {
          "ccdCodes": [
            "NAG", "NAG", "BMA", "MAN", "MAN", "NAG", "NAG", "GAL",
"GAL", "FUC"
          ],
          "id": "NG"
        }
      },
      {
        "ligand": {
          "ccdCodes": [
            "NAG", "NAG", "BMA", "MAN", "MAN", "NAG", "NAG", "GAL",
"GAL", "FUC"
          ],
          "id": ["STGALNGA", "STGALNGB"]
        }
      }
    ],
    "dialect": "alphafold3",
    "version": 2,
    "bondedAtomPairs": [
    [["NG",1,"O4"],["NG",2,"C1"]],
    [["NG",2,"O4"],["NG",3,"C1"]],
    [["NG",3,"O3"],["NG",4,"C1"]],
```

```
  [["NG",3,"O6"],["NG",5,"C1"]],
  [["NG",4,"O2"],["NG",6,"C1"]],
  [["NG",5,"O2"],["NG",7,"C1"]],
  [["NG",6,"O4"],["NG",8,"C1"]],
  [["NG",7,"O4"],["NG",9,"C1"]],
  [["NG",1,"O6"],["NG",10,"C1"]],

  [["STGAL",149,"ND2"],["STGALNGA",1,"C1"]],
  [["STGALNGA",1,"O4"],["STGALNGA",2,"C1"]],
  [["STGALNGA",2,"O4"],["STGALNGA",3,"C1"]],
  [["STGALNGA",3,"O3"],["STGALNGA",4,"C1"]],
  [["STGALNGA",3,"O6"],["STGALNGA",5,"C1"]],
  [["STGALNGA",4,"O2"],["STGALNGA",6,"C1"]],
  [["STGALNGA",5,"O2"],["STGALNGA",7,"C1"]],
  [["STGALNGA",6,"O4"],["STGALNGA",8,"C1"]],
  [["STGALNGA",7,"O4"],["STGALNGA",9,"C1"]],
  [["STGALNGA",1,"O6"],["STGALNGA",10,"C1"]],

  [["STGAL",161,"ND2"],["STGALNGB",1,"C1"]],
  [["STGALNGB",1,"O4"],["STGALNGB",2,"C1"]],
  [["STGALNGB",2,"O4"],["STGALNGB",3,"C1"]],
  [["STGALNGB",3,"O3"],["STGALNGB",4,"C1"]],
  [["STGALNGB",3,"O6"],["STGALNGB",5,"C1"]],
  [["STGALNGB",4,"O2"],["STGALNGB",6,"C1"]],
  [["STGALNGB",5,"O2"],["STGALNGB",7,"C1"]],
  [["STGALNGB",6,"O4"],["STGALNGB",8,"C1"]],
  [["STGALNGB",7,"O4"],["STGALNGB",9,"C1"]],
  [["STGALNGB",1,"O6"],["STGALNGB",10,"C1"]]

  ]
}
```

Sup. Fig. 3f. HsEPO carrying G2F


```
{
    "name": "epo_g2f",
    "modelSeeds": [
      1
    ],
    "sequences": [
      {
        "protein": {
          "sequence":
"MGVHECPAWLWLLLSLLSLPLGLPVLGAPPRLICDSRVLERYLLEAKEAENITTGCAEHCSLNENITVP
DTKVNFYAWKRMEVGQQAVEVWQGLALLSEAVLRGQALLVNSSQPWEPLQLHVDKAVSGLRSLTTLLRAL
GAQKEAISPPDAASAAPLRTITADTFRKLFRVYSNFLRGKLKLYTGEACRTGDR",
          "id": "EPO"
        }
      },
      {
        "ligand": {
          "ccdCodes": [
            "NAG", "NAG", "BMA", "MAN", "MAN", "NAG", "NAG", "GAL",
"GAL", "FUC"
          ],
          "id": "NG"
        }
      }
    ],
    "dialect": "alphafold3",
    "version": 2,
    "bondedAtomPairs": [
    [["EPO",51,"ND2"],["NG",1,"C1"]],
    [["NG",1,"O4"],["NG",2,"C1"]],
    [["NG",2,"O4"],["NG",3,"C1"]],
    [["NG",3,"O3"],["NG",4,"C1"]],
    [["NG",3,"O6"],["NG",5,"C1"]],
    [["NG",4,"O2"],["NG",6,"C1"]],
    [["NG",5,"O2"],["NG",7,"C1"]],
    [["NG",6,"O4"],["NG",8,"C1"]],
    [["NG",7,"O4"],["NG",9,"C1"]],
    [["NG",1,"O6"],["NG",10,"C1"]]
    ]
  }
```

Sup. Fig. 3g. HsST6GAL1 complexed with CMP-Sia and HsEPO carrying G2F

```
{
    "name": "st6gal1_ncc_epo_g2f",
    "modelSeeds": [
      1
    ],
    "sequences": [
      {
        "protein": {
          "sequence":
"MIHTNLKKKFSCCVLVFLLFAVICVWKEKKKGSYYDSFKLQTKEFQVLKSLGKLAMGSDSQSVSSSSTQ
DPHRGRQTLGSLRGLAKAKPEASFQVWNKDSSSKNLIPRLQKIWKNYLSMNKYKVSYKGPGPGIKFSAEA
LRCHLRDHVNVSMVEVTDFPFNTSEWEGYLPKESIRTKAGPWGRCAVVSSAGSLKSSQLGREIDDHDAVL
RFNGAPTANFQQDVGTKTTIRLMNSQLVTTEKRFLKDSLYNEGILIVWDPSVYHSDIPKWYQNPDYNFFN
NYKTYRKLHPNQPFYILKPQMPWELWDILQEISPEEIQPNPPSSGMLGIIIMMTLCDQVDIYEFLPSKRK
TDVCYYYQKFFDSACTMGAYHPLLYEKNLVKHLNQGTDEDIYLLGKATLPGFRTIHC",
          "id": "STGAL"
        }
      },
      {
        "ligand": {
          "ccdCodes": [
            "NCC"
          ],
          "id": "CMPSIA"
        }
      },
      {
        "protein": {
          "sequence":
"MGVHECPAWLWLLLSLLSLPLGLPVLGAPPRLICDSRVLERYLLEAKEAENITTGCAEHCSLNENITVP
DTKVNFYAWKRMEVGQQAVEVWQGLALLSEAVLRGQALLVNSSQPWEPLQLHVDKAVSGLRSLTTLLRAL
GAQKEAISPPDAASAAPLRTITADTFRKLFRVYSNFLRGKLKLYTGEACRTGDR",
          "id": "EPO"
        }
      },
      {
        "ligand": {
          "ccdCodes": [
            "NAG", "NAG", "BMA", "MAN", "MAN", "NAG", "NAG", "GAL",
"GAL", "FUC"
          ],
          "id": "NG"
        }
      }
    ],
    "dialect": "alphafold3",
    "version": 2,
    "bondedAtomPairs": [
    [["EPO",51,"ND2"],["NG",1,"C1"]],
    [["NG",1,"O4"],["NG",2,"C1"]],
    [["NG",2,"O4"],["NG",3,"C1"]],
```

```
  [["NG",3,"O3"],["NG",4,"C1"]],
  [["NG",3,"O6"],["NG",5,"C1"]],
  [["NG",4,"O2"],["NG",6,"C1"]],
  [["NG",5,"O2"],["NG",7,"C1"]],
  [["NG",6,"O4"],["NG",8,"C1"]],
  [["NG",7,"O4"],["NG",9,"C1"]],
  [["NG",1,"O6"],["NG",10,"C1"]]
  ]
}
```

Sup. Fig. 4. HsB3GALT5 complexed with Core 3 O-GalNAc


```
{
    "name": "b3galt5_gdu_mn_type1lacdinac",
    "modelSeeds": [
      1
    ],
    "sequences": [

      {
        "protein": {
          "sequence":
"FKEQSFVYKKDGNFLKLPDTDCRQTPPFLVLLVTSSHKQLAERMAIRQTWGKERMVKGKQLKTFFLLGT
TSSAAETKEVDQESQRHGDIIQKDFLDVYYNLTLKTMMGIEWVHRFCPQAAFVMKTDSDMFINVDYLTEL
LLKKNRTTRFFTGFLKLNEFPIRQPFSKWFVSKSEYPWDRYPPFCSGTGYVFSGDVASQVYNVSKSVPYI
KLEDVFVGLCLERLNIRLEELHSQPTFFPGGLRFSVCLFRRIVACHFIKPRTLLDYWQALENSRGEDCP"
,
          "id": ["BGALT"]
        }
      },
      {
        "ligand": {
          "ccdCodes": [
            "A2G", "NAG"
          ],
          "id": "GLYCAN"
        }
      },
      {
        "ligand": {
          "ccdCodes": [
            "GDU"
          ],
          "id": "UDPGAL"
        }
      },
      {
        "ligand": {
          "ccdCodes": [
            "MN"
          ],
          "id": "MN"
        }
      }
    ],
    "dialect": "alphafold3",
    "version": 2,
    "bondedAtomPairs": [
    [["GLYCAN",1,"O3"],["GLYCAN",2,"C1"]]
    ]
  }
```

Sup. Fig. 5. PmHS2 complexed with HS 5-mer


```
{
    "name": "8viw_hs5_2ud1_2ugb_4mn",
    "modelSeeds": [
      1
    ],
    "sequences": [

      {
        "protein": {
          "sequence":
"GSAAADKQTTSITDLYNEVAKSDLGLVKETNSANPLVSIIMTSHNTAQFIEASINSLLLQTYKNIEIII
VDDDSSDNTFEIASRIANTTSKVRVFRLNSNLGTYFAKNTGILKSKGDIIFFQDSDDVCHHERIERCVNI
LLANKETIAVRCAYSRLAPETQHIIKVNNMDYRLGFITLGMHRKVFQEIGFFNCTTKGSDDEFFHRIAKY
YGKEKIKNLLLPLYYNTMRENSLFTDMVEWIDNHNIIQKMSDTRQHYATLFQAMHNETASHDFKNLFQFP
RIYDALPVPQEMSKLSNPKIPVYINICSIPSRIAQLRRIIGILKNQCDHFHIYLDGYVEIPDFIKNLGNK
ATVVHCKDKDNSIRDNGKFILLEELIEKNQDGYYITCDDDIIYPSDYINTMIKKLNEYDDKAVIGLHGIL
FPSRMTKYFSADRLVYSFYKPLEKDKAVNVLGTGTVSFRVSLFNQFSLSDFTHSGMADIYFSLLCKKNNI
LQICISRPANWLTEDNRDSETLYHQYRDNDEQQTQLIMENGPWGYSSIYPLVKNHPKFTDLIPCLPFYFL
",
          "id": ["PMHSA", "PMHSB"]
        }
      },
      {
        "ligand": {
          "ccdCodes": [
            "BDP", "GNS", "BDP", "GNS", "BDP"
          ],
          "id": ["GLYCANA", "GLYCANB"]
        }
      },
      {
        "ligand": {
          "ccdCodes": [
            "UD1"
          ],
          "id": ["UDPGLCNACA", "UDPGLCNACB"]
        }
      },
      {
        "ligand": {
          "ccdCodes": [
            "UGA"
          ],
          "id": ["UDPGLCAA", "UDPGLCAB"]
        }
      },
      {
        "ligand": {
          "ccdCodes": [
            "MN"
```

```
        ],
        "id": ["MNA", "MNB", "MNC", "MND"]
      }
    }
  ],
  "dialect": "alphafold3",
  "version": 2,
  "bondedAtomPairs": [
  [["GLYCANA",1,"O4"],["GLYCANA",2,"C1"]],
  [["GLYCANA",2,"O4"],["GLYCANA",3,"C1"]],
  [["GLYCANA",3,"O4"],["GLYCANA",4,"C1"]],
  [["GLYCANA",4,"O4"],["GLYCANA",5,"C1"]],

  [["GLYCANB",1,"O4"],["GLYCANB",2,"C1"]],
  [["GLYCANB",2,"O4"],["GLYCANB",3,"C1"]],
  [["GLYCANB",3,"O4"],["GLYCANB",4,"C1"]],
  [["GLYCANB",4,"O4"],["GLYCANB",5,"C1"]]
  ]
}
```

Sup. Fig. 6. HsAggrecan G1 complexed with HA 10-mer


```
{
    "name": "9dff_ha9mer",
    "modelSeeds": [
      1
    ],
    "sequences": [

      {
        "protein": {
          "sequence":
"GPGSETSDHDNSLSVSIPQPSPLRVLLGTSLTIPCYFIDPMHPVTTAPSTAPLAPRIKWSRVSKEKEVV
LLVATEGRVRVNSAYQDKVSLPNYPAIPSDATLEVQSLRSNDSGVYRCEVMHGIEDSEATLEVVVKGIVF
HYRAISTRYTLDFDRAQRACLQNSAIIATPEQLQAAYEDGFHQCDAGWLADQTVRYPIHTPREGCYGDKD
EFPGVRTYGIRDTNETYDVYCFAEEMEGEVFYATSPEKFTFQEAANECRRLGARLATTGQLYLAWQAGMD
MCSAGWLADRSVRYPISKARPNCGGNLLGVRTVYVHANQTGYPDPSSRYDAICYTG",
          "id": ["AGGRECAN"]
        }
      },

      {
        "ligand": {
          "ccdCodes": [
            "NAG", "BDP","NAG", "BDP","NAG", "BDP","NAG", "BDP","NAG"
          ],
          "id": ["GLYCAN"]
        }
      }
    ],
    "dialect": "alphafold3",
    "version": 2,
    "bondedAtomPairs": [
    [["GLYCAN",1,"O3"],["GLYCAN",2,"C1"]],
    [["GLYCAN",2,"O4"],["GLYCAN",3,"C1"]],
    [["GLYCAN",3,"O3"],["GLYCAN",4,"C1"]],
    [["GLYCAN",4,"O4"],["GLYCAN",5,"C1"]],
    [["GLYCAN",5,"O3"],["GLYCAN",6,"C1"]],
    [["GLYCAN",6,"O4"],["GLYCAN",7,"C1"]],
    [["GLYCAN",7,"O3"],["GLYCAN",8,"C1"]],
    [["GLYCAN",8,"O4"],["GLYCAN",9,"C1"]]
    ]
  }
```

Sup. Fig. 7. CsPP2 complexed with chitotriose


```
{
    "name": "7w4b_pp2_chitotriose",
    "modelSeeds": [
      1
    ],
    "sequences": [

      {
        "protein": {
          "sequence":
"STHYLAFPRASTITWGDDTRYWSWATVDFCSYAIEEARLLQVSWLDCRWSMDASDFKQDIWYNASVEVM
LTSNASGWNVPLHLEIELPDGSKQESQIVLAGRQPNVWFKIPIGKFILRGSLTSGTIRFGFYNHEGNWKR
GLNIRTLAIQA",
          "id": ["LECTIN"]
        }
      },

      {
        "ligand": {
          "ccdCodes": [
            "NAG", "NAG", "NAG"
          ],
          "id": ["GLYCAN"]
        }
      }
    ],
    "dialect": "alphafold3",
    "version": 2,
    "bondedAtomPairs": [
    [["GLYCAN",1,"O4"],["GLYCAN",2,"C1"]],
    [["GLYCAN",2,"O4"],["GLYCAN",3,"C1"]]
    ]
  }
```

Sup. Fig. 8. BT1258 D161A/E163A complexed with M9


```
{
    "name": "8u48_m9",
    "modelSeeds": [
      1
    ],
    "sequences": [

      {
        "protein": {
          "sequence":
"MPVNQSDNNQSEVVTRSATGIKNIVYIEVNDINPLNAGSYIMDDAPFFDYVILFAANIRGVGSDATLYN
NPNVQYILDHKDTLIKPLQDKGIKVLLGLLGDHTGLGFANMNSAQTEQFATAVANAVSQYGLDGVDFADA
WAEYGRNGYPSGSTGSFSNLITALHNKMPGKTITVFNYGYTSELTGVNSYIDYGIYAFFNSPSWSTGFGM
PNSKFAPYTINLNSAPSAASAQLYSGQVASKGYGAIGYYDLRANNIVSVLNGVAKGAFKSTCTYDGNSYP
KNY",
          "id": ["ENDO"]
        }
      },

      {
        "ligand": {
          "ccdCodes": [
            "NAG", "NAG", "BMA", "MAN", "MAN", "MAN", "MAN", "MAN",
"MAN", "MAN", "MAN"
          ],
          "id": ["GLYCAN"]
        }
      }
    ],
    "dialect": "alphafold3",
    "version": 2,
    "bondedAtomPairs": [
    [["GLYCAN",1,"O4"],["GLYCAN",2,"C1"]],
    [["GLYCAN",2,"O4"],["GLYCAN",3,"C1"]],
    [["GLYCAN",3,"O3"],["GLYCAN",4,"C1"]],
    [["GLYCAN",3,"O6"],["GLYCAN",5,"C1"]],
    [["GLYCAN",5,"O3"],["GLYCAN",6,"C1"]],
    [["GLYCAN",5,"O6"],["GLYCAN",7,"C1"]],
    [["GLYCAN",6,"O2"],["GLYCAN",8,"C1"]],
    [["GLYCAN",7,"O2"],["GLYCAN",9,"C1"]],
    [["GLYCAN",4,"O2"],["GLYCAN",10,"C1"]],
    [["GLYCAN",10,"O2"],["GLYCAN",11,"C1"]]
    ]
  }
```

Sup. Fig. 9. BoPL38 complexed with M4 alginate


```
{
    "name": "bopl38_tetramana_9fhu",
    "modelSeeds": [
      1
    ],
    "sequences": [

      {
        "protein": {
          "sequence":
"MGSSHHHHHHSSGLVPRGSHMASAPLGPFNATLLEQLKNDYQKGEKEVTRYIELQEKVAEKYIKMTPLS
VTAKKKLPPSKDPRDYMTLSPYWWPDSTKIDGLPYIRKDGERNPEVYEYPERENANRFGDAAYCLGVLYY
ITGKEVYAKACANHLRTWFTDPKLGMNPNMTYAQAVPGMKKMRGSGFIDSRRFSRALGVAKLIEGSKSWT
PSDKKKLDDWATAFCYWMENSTQGQRESHAANNHGLWYEAIHLMVLAYLDRTDRIREVAEQSILPKMGAQ
IADDGSLPQELKRTLSLHYSTFALEALMEANQITSQIGINLWSTPASNGKVASQAVDYLYPFYLNPEDWK
FKQIKPFDQSRAAILLYEAGTALGNQKYVDTAKRIGLKYSTSDVETIPYLVLKKK",
          "id": ["LYASE"]
        }
      },
      {
        "ligand": {
          "ccdCodes": [
            "BEM", "BEM", "BEM", "BEM"
          ],
          "id": "GLYCAN"
        }
      }
    ],
    "dialect": "alphafold3",
    "version": 2,
    "bondedAtomPairs": [
    [["GLYCAN",1,"O4"],["GLYCAN",2,"C1"]],
    [["GLYCAN",2,"O4"],["GLYCAN",3,"C1"]],
    [["GLYCAN",3,"O4"],["GLYCAN",4,"C1"]]
    ]
  }
```

Sup. Fig. 10. PtGlucanase complexed with laminarin


```
{
    "name": "7wwc_glucanase_laminaritriose",
    "modelSeeds": [
      1
    ],
    "sequences": [

      {
        "protein": {
          "sequence":
"FNWNKNQVIAHRGAWKKNNFPQNSIASLNEAVKLGCYGSEFDVWMTADHILVVNHDPEFQGLTIEKVNY
ADLLTKTMSNGEKIPTLEAYLLAGKKQKSTKLILEIKPSLISKERGIEVTNKCVEMVQKLKVTDWVEYIS
FDYDYCKRILTLLPNAKVAYLKGEVSAEQMKADKLTGVDYHYSVYQKDNWIENAQKLGLTVNAWTVNAVP
EMQWLLAHNVDYITTNEPELLFDEIKKAPVAQGWKLKWADEFDNSGLPLNKNWGYDVGGRGWGNNELQYY
TDADSANAIVKKGNLNIIALKAEKENRHYTSARLVTKNKFDFKYGRVEVRAMLPKGRGLWPAIWALPTDS
KYGSWPKSGEIDIMEHVGFDPDSVHGTVHTEKFNHVIHTQVGKALKVNNPYTEYHIYAIEWFTDHIDFFI
DDQKYLTFKNTQKGSGDWPFDQNFHILNLAVGGNWGGKKGVDDAIFPATMKVDYVRVFQK",
          "id": ["GLUCANASE"]
        }
      },
      {
        "ligand": {
          "ccdCodes": [
            "BGC", "BGC", "BGC"
          ],
          "id": ["GLYCAN"]
        }
      },
      {
        "ligand": {
          "ccdCodes": [
            "BGC", "BGC"
          ],
          "id": ["GLYCANB"]
        }
      }
    ],
    "dialect": "alphafold3",
    "version": 2,
    "bondedAtomPairs": [
    [["GLYCAN",1,"O3"],["GLYCAN",2,"C1"]],
    [["GLYCAN",2,"O3"],["GLYCAN",3,"C1"]],

    [["GLYCANB",1,"O3"],["GLYCANB",2,"C1"]]
    ]
  }
```

Sup. Fig. 11. BcXylanase E78Q complexed with xylotriose


```
{
    "name": "8qxy_xylanase_xylotriose",
    "modelSeeds": [
      1
    ],
    "sequences": [

      {
        "protein": {
          "sequence":
"ASTDYWQNWTDGGGIVNAVNGSGGNYSVNWSNTGNFVVGKGWTTGSPFRTINYNAGVWAPNGNGYLTLY
GWTRSPLIQYYVVDSWGTYRPTGTYKGTVKSDGGTYDIYTTTRYNAPSIDGDRTTFTQYWSVRQSKRPTG
SNATITFTNHVNAWKSHGMNLGSNWAYQVMATEGYQSSGSSNVTVW",
          "id": ["XYLANASE"]
        }
      },

      {
        "ligand": {
          "ccdCodes": [
            "XYP", "XYP", "XYP"
          ],
          "id": ["GLYCAN"]
        }
      }
    ],
    "dialect": "alphafold3",
    "version": 2,
    "bondedAtomPairs": [
    [["GLYCAN",1,"O4"],["GLYCAN",2,"C1"]],
    [["GLYCAN",2,"O4"],["GLYCAN",3,"C1"]]
    ]
  }
```

Sup. Fig. 12. EcFimH complexed with M3F


```
{
    "name": "fimh_monomer_m3f_7bhd_8bxy",
    "modelSeeds": [
      1
    ],
    "sequences": [

      {
        "protein": {
          "sequence":
"FACKTANGTAIPIGGGSANVYVNLAPVVNVGQNLVVDLSTQIFCHNDYPETITDYVTLQRGSAYGGVLS
NFSGTVKYSGSSYPFPTTSETPRVVYNSRTDKPWPVALYLTPVSSAGGVAIKAGSLIAVLILRQTNNYNS
DDFQFVWNIYANNDVVVPT",
          "id": ["LECTINA"]
        }
      },
      {
        "ligand": {
          "ccdCodes": [
            "NAG", "NAG", "BMA", "MAN", "MAN", "FUC"
          ],
          "id": "NG"
        }
      }
    ],
    "dialect": "alphafold3",
    "version": 2,
    "bondedAtomPairs": [
    [["NG",1,"O4"],["NG",2,"C1"]],
    [["NG",2,"O4"],["NG",3,"C1"]],
    [["NG",3,"O3"],["NG",4,"C1"]],
    [["NG",3,"O6"],["NG",5,"C1"]],
    [["NG",1,"O6"],["NG",6,"C1"]]
    ]
  }
```

Sup. Fig. 12. BoNT/A Y1117V complexed with GM1b


```
{
    "name": "8rvg_gd1a",
    "modelSeeds": [
      1
    ],
    "sequences": [

      {
        "protein": {
          "sequence":
"MGSSHHHHHHSSGLVPRGSHMDTSILNLRYESNHLIDLSRYASKINIGSKVNFDPIDKNQIQLFNLESS
KIEVILKNAIVYNSMYENFSTSFWIRIPKYFNSISLNNEYTIINCMENNSGWKVSLNYGEIIWTLQDTQE
IKQRVVFKYSQMINISDYINRWIFVTITNNRLNNSKIYINGRLIDQKPISNLGNIHASNNIMFKLDGCRD
THRYIWIKYFNLFDKELNEKEIKDLYDNQSNSGILKDFWGDYLQYDKPYYMLNLVDPNKYVDVNNVGIRG
YMYLKGPRGSVMTTNIYLNSSLYRGTKFIIKKYASGNKDNIVRNNDRVYINVVVKNKEYRLATNASQAGV
EKILSALEIPDVGNLSQVVVMKSKNDQGITNKCKMNLQDNNGNDIGFIGFHQFNNIAKLVASNWYNRQIE
RSSRTLGCSWEFIPVDDGWGERPLQ",
          "id": ["BONT"]
        }
      },

      {
        "ligand": {
          "ccdCodes": [
            "GAL", "NGA", "GAL", "SIA"
          ],
          "id": ["GLYCAN"]
        }
      }
    ],
    "dialect": "alphafold3",
    "version": 2,
    "bondedAtomPairs": [
    [["GLYCAN",1,"O4"],["GLYCAN",2,"C1"]],
    [["GLYCAN",2,"O3"],["GLYCAN",3,"C1"]],
    [["GLYCAN",3,"O3"],["GLYCAN",4,"C2"]]
    ]
  }
```

Template 1. A2

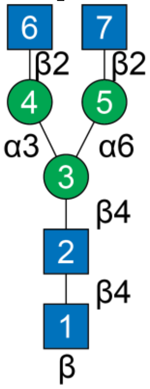


```
{
    "name": "a2",
    "modelSeeds": [
      1
    ],
    "sequences": [
      {
        "ligand": {
          "ccdCodes": [
            "NAG", "NAG", "BMA", "MAN", "MAN", "NAG", "NAG"
          ],
          "id": "NG"
        }
      }
    ],
    "dialect": "alphafold3",
    "version": 2,
    "bondedAtomPairs": [
    [["NG",1,"O4"],["NG",2,"C1"]],
    [["NG",2,"O4"],["NG",3,"C1"]],
    [["NG",3,"O3"],["NG",4,"C1"]],
    [["NG",3,"O6"],["NG",5,"C1"]],
    [["NG",4,"O2"],["NG",6,"C1"]],
    [["NG",5,"O2"],["NG",7,"C1"]]
    ]
  }
```

Template 2. A2F

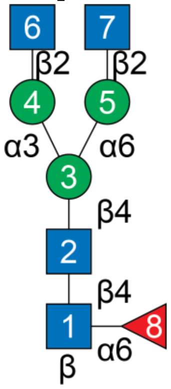


```
{
    "name": "a2f",
    "modelSeeds": [
      1
    ],
    "sequences": [
      {
        "ligand": {
          "ccdCodes": [
            "NAG", "NAG", "BMA", "MAN", "MAN", "NAG", "NAG", "FUC"
          ],
          "id": "NG"
        }
      }
    ],
    "dialect": "alphafold3",
    "version": 2,
    "bondedAtomPairs": [
    [["NG",1,"O4"],["NG",2,"C1"]],
    [["NG",2,"O4"],["NG",3,"C1"]],
    [["NG",3,"O3"],["NG",4,"C1"]],
    [["NG",3,"O6"],["NG",5,"C1"]],
    [["NG",4,"O2"],["NG",6,"C1"]],
    [["NG",5,"O2"],["NG",7,"C1"]],
    [["NG",1,"O6"],["NG",8,"C1"]]
    ]
  }
```

Template 3. A3 (3-arm)

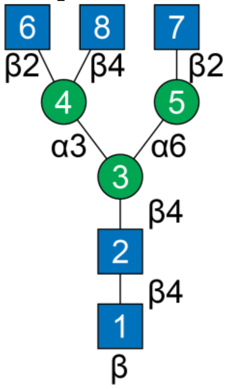


```
{
    "name": "a3_3arm",
    "modelSeeds": [
      1
    ],
    "sequences": [
      {
        "ligand": {
          "ccdCodes": [
            "NAG", "NAG", "BMA", "MAN", "MAN", "NAG", "NAG", "NAG"
          ],
          "id": "NG"
        }
      }
    ],
    "dialect": "alphafold3",
    "version": 2,
    "bondedAtomPairs": [
    [["NG",1,"O4"],["NG",2,"C1"]],
    [["NG",2,"O4"],["NG",3,"C1"]],
    [["NG",3,"O3"],["NG",4,"C1"]],
    [["NG",3,"O6"],["NG",5,"C1"]],
    [["NG",4,"O2"],["NG",6,"C1"]],
    [["NG",5,"O2"],["NG",7,"C1"]],
    [["NG",4,"O4"],["NG",8,"C1"]]
    ]
  }
```

Template 4. A3F (3-arm)

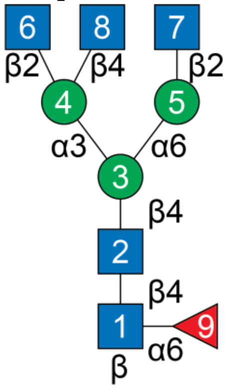


```
{
    "name": "a3f_3arm",
    "modelSeeds": [
      1
    ],
    "sequences": [
      {
        "ligand": {
          "ccdCodes": [
            "NAG", "NAG", "BMA", "MAN", "MAN", "NAG", "NAG", "NAG",
"FUC"
          ],
          "id": "NG"
        }
      }
    ],
    "dialect": "alphafold3",
    "version": 2,
    "bondedAtomPairs": [
    [["NG",1,"O4"],["NG",2,"C1"]],
    [["NG",2,"O4"],["NG",3,"C1"]],
    [["NG",3,"O3"],["NG",4,"C1"]],
    [["NG",3,"O6"],["NG",5,"C1"]],
    [["NG",4,"O2"],["NG",6,"C1"]],
    [["NG",5,"O2"],["NG",7,"C1"]],
    [["NG",4,"O4"],["NG",8,"C1"]],
    [["NG",1,"O6"],["NG",9,"C1"]]
    ]
  }
```

Template 5. A3 (6-arm)

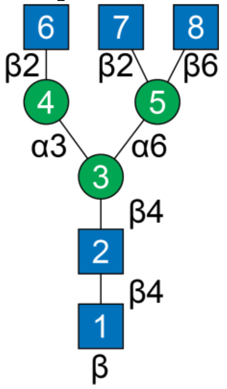


```
{
    "name": "a3_6arm",
    "modelSeeds": [
      1
    ],
    "sequences": [
      {
        "ligand": {
          "ccdCodes": [
            "NAG", "NAG", "BMA", "MAN", "MAN", "NAG", "NAG", "NAG"
          ],
          "id": "NG"
        }
      }
    ],
    "dialect": "alphafold3",
    "version": 2,
    "bondedAtomPairs": [
    [["NG",1,"O4"],["NG",2,"C1"]],
    [["NG",2,"O4"],["NG",3,"C1"]],
    [["NG",3,"O3"],["NG",4,"C1"]],
    [["NG",3,"O6"],["NG",5,"C1"]],
    [["NG",4,"O2"],["NG",6,"C1"]],
    [["NG",5,"O2"],["NG",7,"C1"]],
    [["NG",5,"O6"],["NG",8,"C1"]]
    ]
  }
```

Template 6. A3F (6-arm)

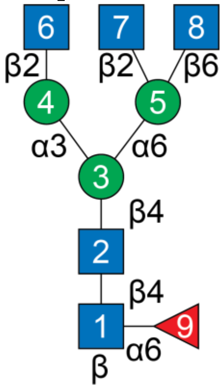


```
{
    "name": "a3f_6arm",
    "modelSeeds": [
      1
    ],
    "sequences": [
      {
        "ligand": {
          "ccdCodes": [
            "NAG", "NAG", "BMA", "MAN", "MAN", "NAG", "NAG", "NAG",
"FUC"
          ],
          "id": "NG"
        }
      }
    ],
    "dialect": "alphafold3",
    "version": 2,
    "bondedAtomPairs": [
    [["NG",1,"O4"],["NG",2,"C1"]],
    [["NG",2,"O4"],["NG",3,"C1"]],
    [["NG",3,"O3"],["NG",4,"C1"]],
    [["NG",3,"O6"],["NG",5,"C1"]],
    [["NG",4,"O2"],["NG",6,"C1"]],
    [["NG",5,"O2"],["NG",7,"C1"]],
    [["NG",5,"O6"],["NG",8,"C1"]],
    [["NG",1,"O6"],["NG",9,"C1"]]
    ]
  }
```

Template 7. A4 (3- and 6-arm)

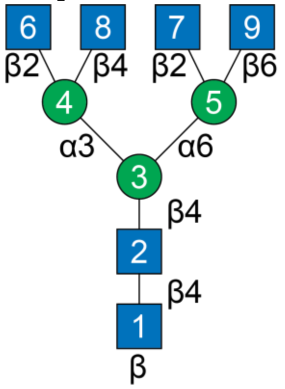


```
{
    "name": "a4_36arm",
    "modelSeeds": [
      1
    ],
    "sequences": [
      {
        "ligand": {
          "ccdCodes": [
            "NAG", "NAG", "BMA", "MAN", "MAN", "NAG", "NAG", "NAG",
"NAG"
          ],
          "id": "NG"
        }
      }
    ],
    "dialect": "alphafold3",
    "version": 2,
    "bondedAtomPairs": [
    [["NG",1,"O4"],["NG",2,"C1"]],
    [["NG",2,"O4"],["NG",3,"C1"]],
    [["NG",3,"O3"],["NG",4,"C1"]],
    [["NG",3,"O6"],["NG",5,"C1"]],
    [["NG",4,"O2"],["NG",6,"C1"]],
    [["NG",5,"O2"],["NG",7,"C1"]],
    [["NG",4,"O4"],["NG",8,"C1"]],
    [["NG",5,"O6"],["NG",9,"C1"]]
    ]
  }
```

Template 8. A4F (3- and 6-arm)

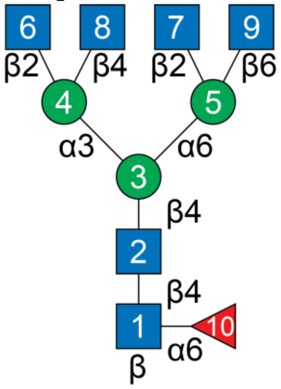


```
{
    "name": "a4f_36arm",
    "modelSeeds": [
      1
    ],
    "sequences": [
      {
        "ligand": {
          "ccdCodes": [
            "NAG", "NAG", "BMA", "MAN", "MAN", "NAG", "NAG", "NAG",
"NAG", "FUC"
          ],
          "id": "NG"
        }
      }
    ],
    "dialect": "alphafold3",
    "version": 2,
    "bondedAtomPairs": [
    [["NG",1,"O4"],["NG",2,"C1"]],
    [["NG",2,"O4"],["NG",3,"C1"]],
    [["NG",3,"O3"],["NG",4,"C1"]],
    [["NG",3,"O6"],["NG",5,"C1"]],
    [["NG",4,"O2"],["NG",6,"C1"]],
    [["NG",5,"O2"],["NG",7,"C1"]],
    [["NG",4,"O4"],["NG",8,"C1"]],
    [["NG",5,"O6"],["NG",9,"C1"]],
    [["NG",1,"O6"],["NG",10,"C1"]]
    ]
  }
```

Template 9. G2F

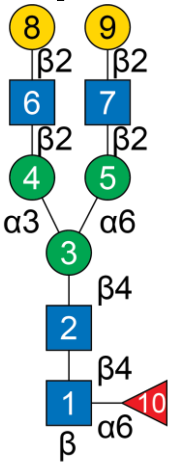


```
{
    "name": "g2f",
    "modelSeeds": [
      1
    ],
    "sequences": [
      {
        "ligand": {
          "ccdCodes": [
            "NAG", "NAG", "BMA", "MAN", "MAN", "NAG", "NAG", "GAL",
"GAL", "FUC"
          ],
          "id": "NG"
        }
      }
    ],
    "dialect": "alphafold3",
    "version": 2,
    "bondedAtomPairs": [
    [["NG",1,"O4"],["NG",2,"C1"]],
    [["NG",2,"O4"],["NG",3,"C1"]],
    [["NG",3,"O3"],["NG",4,"C1"]],
    [["NG",3,"O6"],["NG",5,"C1"]],
    [["NG",4,"O2"],["NG",6,"C1"]],
    [["NG",5,"O2"],["NG",7,"C1"]],
    [["NG",6,"O4"],["NG",8,"C1"]],
    [["NG",7,"O4"],["NG",9,"C1"]],
    [["NG",1,"O6"],["NG",10,"C1"]]
    ]
  }
```

Template 10. G2S2 (α2,3-sia)

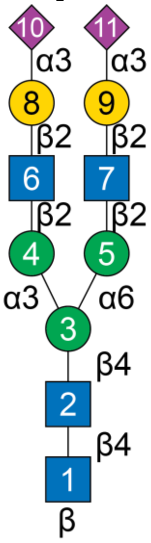


```
{
    "name": "g2s2_33_usersia",
    "modelSeeds": [
      1
    ],
    "sequences": [
      {
        "ligand": {
          "ccdCodes": [
            "NAG", "NAG", "BMA", "MAN", "MAN", "NAG", "NAG", "GAL",
"GAL", "SIA2", "SIA2"
          ],
          "id": "NG"
        }
      }
    ],
    "dialect": "alphafold3",
    "version": 2,
    "bondedAtomPairs": [
    [["NG",1,"O4"],["NG",2,"C1"]],
    [["NG",2,"O4"],["NG",3,"C1"]],
    [["NG",3,"O3"],["NG",4,"C1"]],
    [["NG",3,"O6"],["NG",5,"C1"]],
    [["NG",4,"O2"],["NG",6,"C1"]],
    [["NG",5,"O2"],["NG",7,"C1"]],
    [["NG",6,"O4"],["NG",8,"C1"]],
    [["NG",7,"O4"],["NG",9,"C1"]],
    [["NG",8,"O3"],["NG",10,"C2"]],
    [["NG",9,"O3"],["NG",11,"C2"]]
    ],
    "userCCD": "data_SIA2\n#\n_chem_comp.id SIA\n_chem_comp.name 'N-
acetyl-alpha-neuraminic acid'\n_chem_comp.type 'D-saccharide, alpha
linking'\n_chem_comp.pdbx_type ATOMS\n_chem_comp.formula 'C11 H19 N
O9'\n_chem_comp.mon_nstd_parent_comp_id ?\n_chem_comp.pdbx_synonyms
```

'N-acetylneuraminic acid; sialic acid; alpha-sialic acid; O-SIALIC ACID'\n_chem_comp.pdbx_formal_charge 0\n_chem_comp.pdbx_initial_date 1999-07-08\n_chem_comp.pdbx_modified_date 2024-09-27\n_chem_comp.pdbx_ambiguous_flag N\n_chem_comp.pdbx_release_status REL\n_chem_comp.pdbx_replaced_by ?\n_chem_comp.pdbx_replaces NAN\n_chem_comp.formula_weight 309.270\n_chem_comp.one_letter_code ?\n_chem_comp.three_letter_code SIA\n_chem_comp.pdbx_model_coordinates_details ?\n_chem_comp.pdbx_model_coordinates_missing_flag N\n_chem_comp.pdbx_ideal_coordinates_details ?\n_chem_comp.pdbx_ideal_coordinates_missing_flag N\n_chem_comp.pdbx_model_coordinates_db_code ?\n_chem_comp.pdbx_subcomponent_list ?\n_chem_comp.pdbx_processing_site EBI\n_chem_comp.pdbx_pcm Y\n#\nloop_\n_pdbx_chem_comp_synonyms.ordinal\n_pdbx_chem_comp_synonyms.comp_id\n_pdbx_chem_comp_synonyms.name\n_pdbx_chem_comp_synonyms.provenance\n_pdbx_chem_comp_synonyms.type\n1 SIA 'N-acetylneuraminic acid' PDB ?\n2 SIA 'sialic acid' PDB ?\n3 SIA 'alpha-sialic acid' PDB ?\n4 SIA 'O-SIALIC ACID' PDB ?\n#\nloop_\n_chem_comp_atom.comp_id\n_chem_comp_atom.atom_id\n_chem_comp_atom.alt_atom_id\n_chem_comp_atom.type_symbol\n_chem_comp_atom.charge\n_chem_comp_atom.pdbx_align\n_chem_comp_atom.pdbx_aromatic_flag\n_chem_comp_atom.pdbx_leaving_atom_flag\n_chem_comp_atom.pdbx_stereo_config\n_chem_comp_atom.pdbx_backbone_atom_flag\n_chem_comp_atom.pdbx_n_terminal_atom_flag\n_chem_comp_atom.pdbx_c_terminal_atom_flag\n_chem_comp_atom.model_Cartn_x\n_chem_comp_atom.model_Cartn_y\n_chem_comp_atom.model_Cartn_z\n_chem_comp_atom.pdbx_model_Cartn_x_ideal\n_chem_comp_atom.pdbx_model_Cartn_y_ideal\n_chem_comp_atom.pdbx_model_Cartn_z_ideal\n_chem_comp_atom.pdbx_component_atom_id\n_chem_comp_atom.pdbx_component_comp_id\n_chem_comp_atom.pdbx_ordinal\nSIA C1 C1 C 0 1 N N N N N N -2.196 58.872 -5.981 -2.502 -0.832 0.174 C1 SIA 1\nSIA C2 C2 C 0 1 N N R N N N -1.870 58.021 -7.211 -2.171 0.628 0.342 C2 SIA 2\nSIA C3 C3 C 0 1 N N N N N N -0.844 56.899 -7.306 -1.789 0.898 1.800 C3 SIA 3\nSIA C4 C4 C 0 1 N N S N N N -1.157 55.904 -8.413 -0.586 0.023 2.171 C4 SIA 4\nSIA C5 C5 C 0 1 N N R N N N -2.015 56.516 -9.517 0.529 0.264 1.148 C5 SIA 5\nSIA C6 C6 C 0 1 N N R N N N -3.352 56.956 -8.912 -0.026 0.043 -0.259 C6 SIA 6\nSIA C7 C7 C 0 1 N N R N N N -4.224 57.698 -9.942 1.088 0.251 -1.286 C7 SIA 7\nSIA C8 C8 C 0 1 N N R N N N -5.571 58.131 -9.360 0.535 0.021 -2.694 C8 SIA 8\nSIA C9 C9 C 0 1 N N N N N N -6.601 58.674 -10.381 1.650 0.229 -3.721 C9 SIA 9\nSIA C10 C10 C 0 1 N N N N N N -1.897 55.374 -11.759 2.632 -0.329 2.226 C10 SIA 10\nSIA C11 C11 C 0 1 N N N N N N -2.200 54.057 -12.454 3.763 -1.292 2.478 C11 SIA 11\nSIA N5 N5 N 0 1 N N N N N N -2.202 55.444 -10.478 1.629 -0.671 1.394 N5 SIA 12\nSIA O1A O1A O 0 1 N N N N N N -1.289 58.815 -5.130 -2.191 -1.408 -0.841 O1A SIA 13\nSIA O1B O1B O 0 1 N N N N N N -3.210 59.504 -5.631 -3.141 -1.493 1.152 O1B SIA 14\nSIA O4 O4 O 0 1 N N N N N N 0.072 55.523 -8.986 -0.123 0.370 3.478 O4 SIA 16\nSIA O6 O6 O 0 1 N N N N N N -3.149 57.908 -7.847 -1.082 0.968 -0.513 O6 SIA 17\nSIA O7 O7 O 0 1 N N N N N N -3.594 58.883 -10.402 1.588 1.586 -1.183 O7 SIA 18\nSIA O8 O8 O 0 1 N N N N N N -6.119 56.946 -8.828 0.035 -1.313 -2.797 O8 SIA 19\nSIA O9 O9 O 0 1 N N N N N N -6.931 57.687 -11.346 1.133 0.014 -5.035 O9 SIA 20\nSIA O10 O10 O 0 1 N N N N N N -1.423

56.357 -12.331 2.624 0.753 2.772 O10 SIA 21\nSIA H32 H31 H 0 1 N N N N
N N -0.702 56.484 -6.300 -2.631 0.655 2.448 H32 SIA 22\nSIA H31 H32 H
0 1 N N N N N N 0.120 57.408 -7.182 -1.526 1.949 1.919 H31 SIA 23\nSIA
H4 H4 H 0 1 N N N N N N -1.651 55.060 -7.897 -0.878 -1.026 2.153 H4
SIA 24\nSIA H5 H5 H 0 1 N N N N N N -1.506 57.375 -9.979 0.893 1.287
1.240 H5 SIA 25\nSIA H6 H6 H 0 1 N N N N N N -3.850 56.075 -8.492 -
0.408 -0.973 -0.341 H6 SIA 26\nSIA H7 H7 H 0 1 N N N N N N -4.339
57.176 -10.907 1.896 -0.454 -1.093 H7 SIA 27\nSIA H8 H8 H 0 1 N N N N
N N -5.473 58.871 -8.553 -0.272 0.728 -2.887 H8 SIA 28\nSIA H92 H91 H
0 1 N N N N N N -6.054 59.459 -10.925 2.031 1.247 -3.642 H92 SIA
29\nSIA H91 H92 H 0 1 N N N N N N -7.587 59.029 -10.055 2.457 -0.476 -
3.528 H91 SIA 30\nSIA H111 H111 H 0 0 N N N N N N -3.215 53.728 -
12.207 4.474 -0.844 3.172 H111 SIA 31\nSIA H113 H112 H 0 0 N N N N N N
-1.550 53.279 -12.033 3.368 -2.213 2.907 H113 SIA 32\nSIA H112 H113 H
0 0 N N N N N N -2.005 54.041 -13.531 4.266 -1.516 1.537 H112 SIA
33\nSIA HN5 HN5 H 0 1 N N N N N N -2.566 54.658 -10.003 1.635 -1.538
0.957 HN5 SIA 34\nSIA HO1B HOB1 H 0 0 N N N N N N -3.412 60.032 -4.867
-3.353 -2.430 1.044 HO1B SIA 35\nSIA HO4 HO4 H 0 1 N Y N N N N 0.427
54.801 -8.430 -0.854 0.203 4.087 HO4 SIA 37\nSIA HO7 HO7 H 0 1 N Y N N
N N -3.109 58.884 -9.548 0.844 2.177 -1.360 HO7 SIA 38\nSIA HO8 HO8 H
0 1 N Y N N N N -7.071 57.051 -8.962 0.779 -1.904 -2.620 HO8 SIA
39\nSIA HO9 HO9 H 0 1 N Y N N N N -6.783 56.885 -10.808 1.866 0.155 -
5.650 HO9 SIA
40\n#\nloop_\n_chem_comp_bond.comp_id\n_chem_comp_bond.atom_id_1\n_che
m_comp_bond.atom_id_2\n_chem_comp_bond.value_order\n_chem_comp_bond.pd
bx_aromatic_flag\n_chem_comp_bond.pdbx_stereo_config\n_chem_comp_bond.
pdbx_ordinal\nSIA C1 C2 SING N N 1\nSIA C1 O1A DOUB N N 2\nSIA C1 O1B
SING N N 3\nSIA C2 C3 SING N N 4\nSIA C2 O6 SING N N 6\nSIA C3 C4 SING
N N 7\nSIA C3 H32 SING N N 8\nSIA C3 H31 SING N N 9\nSIA C4 C5 SING N
N 10\nSIA C4 O4 SING N N 11\nSIA C4 H4 SING N N 12\nSIA C5 C6 SING N N
13\nSIA C5 N5 SING N N 14\nSIA C5 H5 SING N N 15\nSIA C6 C7 SING N N
16\nSIA C6 O6 SING N N 17\nSIA C6 H6 SING N N 18\nSIA C7 C8 SING N N
19\nSIA C7 O7 SING N N 20\nSIA C7 H7 SING N N 21\nSIA C8 C9 SING N N
22\nSIA C8 O8 SING N N 23\nSIA C8 H8 SING N N 24\nSIA C9 O9 SING N N
25\nSIA C9 H92 SING N N 26\nSIA C9 H91 SING N N 27\nSIA C10 C11 SING N
N 28\nSIA C10 N5 SING N N 29\nSIA C10 O10 DOUB N N 30\nSIA C11 H111
SING N N 31\nSIA C11 H113 SING N N 32\nSIA C11 H112 SING N N 33\nSIA
N5 HN5 SING N N 34\nSIA O1B HO1B SING N N 35\nSIA O4 HO4 SING N N
37\nSIA O7 HO7 SING N N 38\nSIA O8 HO8 SING N N 39\nSIA O9 HO9 SING N
N
40\n#\nloop_\n_pdbx_chem_comp_descriptor.comp_id\n_pdbx_chem_comp_desc
riptor.type\n_pdbx_chem_comp_descriptor.program\n_pdbx_chem_comp_descr
iptor.program_version\n_pdbx_chem_comp_descriptor.descriptor\nSIA
SMILES ACDLabs 10.04 'O=C(O)C1(O)OC(C(O)C(O)CO)C(NC(=O)C)C(O)C1'\nSIA
SMILES_CANONICAL CACTVS 3.341
'CC(=O)N[C@@H]1[C@@H](O)C[C@@](O)(O[C@H]1[C@H](O)[C@H](O)CO)C(O)=O'\nS
IA SMILES CACTVS 3.341
'CC(=O)N[CH]1[CH](O)C[C](O)(O[CH]1[CH](O)[CH](O)CO)C(O)=O'\nSIA
SMILES_CANONICAL 'OpenEye OEToolkits' 1.5.0
'CC(=O)N[C@@H]1[C@H](C[C@@](O[C@H]1[C@@H]([C@@H](CO)O)O)(C(=O)O)O)O'\n
SIA SMILES 'OpenEye OEToolkits' 1.5.0
'CC(=O)NC1C(CC(OC1C(C(CO)O)O)(C(=O)O)O)O'\nSIA InChI InChI 1.03

'InChI=1S/C11H19NO9/c1-4(14)12-7-5(15)2-11(20,10(18)19)21-
9(7)8(17)6(16)3-13/h5-9,13,15-17,20H,2-
3H2,1H3,(H,12,14)(H,18,19)/t5-,6+,7+,8+,9+,11+/m0/s1'\nSIA InChIKey
InChI 1.03 SQVRNKJHWKZAKO-YRMXFSIDSA-
N\n#\nloop_\n_pdbx_chem_comp_identifier.comp_id\n_pdbx_chem_comp_ident
ifier.type\n_pdbx_chem_comp_identifier.program\n_pdbx_chem_comp_identi
fier.program_version\n_pdbx_chem_comp_identifier.identifier\nSIA
'SYSTEMATIC NAME' ACDLabs 10.04 '5-(acetylamino)-3,5-dideoxy-D-
glycero-alpha-D-galacto-non-2-ulopyranosonic acid'\nSIA 'SYSTEMATIC
NAME' 'OpenEye OEToolkits' 1.5.0 '(2R,4S,5R,6R)-5-acetamido-2,4-
dihydroxy-6-[(1R,2R)-1,2,3-trihydroxypropyl]oxane-2-carboxylic
acid'\nSIA 'CONDENSED IUPAC CARBOHYDRATE SYMBOL' GMML 1.0
DNeup5Aca\nSIA 'COMMON NAME' GMML 1.0 'N-acetyl-a-D-neuraminic
acid'\nSIA 'IUPAC CARBOHYDRATE SYMBOL' PDB-CARE 1.0 a-D-Neup5Ac\nSIA
'SNFG CARBOHYDRATE SYMBOL' GMML 1.0
Neu5Ac\n#\nloop_\n_pdbx_chem_comp_feature.comp_id\n_pdbx_chem_comp_fea
ture.type\n_pdbx_chem_comp_feature.value\n_pdbx_chem_comp_feature.sour
ce\n_pdbx_chem_comp_feature.support\nSIA 'CARBOHYDRATE ISOMER' D
PDB ?\nSIA 'CARBOHYDRATE RING' pyranose PDB ?\nSIA 'CARBOHYDRATE
ANOMER' alpha PDB ?\nSIA 'CARBOHYDRATE PRIMARY CARBONYL GROUP' ketose
PDB ?\n#\nloop_\n_pdbx_chem_comp_audit.comp_id\n_pdbx_chem_comp_audit.
action_type\n_pdbx_chem_comp_audit.date\n_pdbx_chem_comp_audit.process
ing_site\nSIA 'Create component' 1999-07-08 EBI\nSIA 'Modify
descriptor' 2011-06-04 RCSB\nSIA 'Other modification' 2019-08-12
RCSB\nSIA 'Other modification' 2019-12-19 RCSB\nSIA 'Other
modification' 2020-07-03 RCSB\nSIA 'Modify name' 2020-07-17 RCSB\nSIA
'Modify synonyms' 2020-07-17 RCSB\nSIA 'Modify atom id' 2020-07-17
RCSB\nSIA 'Modify component atom id' 2020-07-17 RCSB\nSIA 'Modify PCM'
2024-09-27 PDBE\n#\n_pdbx_chem_comp_pcm.pcm_id
1\n_pdbx_chem_comp_pcm.comp_id
SIA\n_pdbx_chem_comp_pcm.modified_residue_id
THR\n_pdbx_chem_comp_pcm.type None\n_pdbx_chem_comp_pcm.category
Carbohydrate\n_pdbx_chem_comp_pcm.position 'Amino-acid side
chain'\n_pdbx_chem_comp_pcm.polypeptide_position 'Any
position'\n_pdbx_chem_comp_pcm.comp_id_linking_atom
C2\n_pdbx_chem_comp_pcm.modified_residue_id_linking_atom
OG1\n_pdbx_chem_comp_pcm.uniprot_specific_ptm_accession ?\n_pdbx_chem_
comp_pcm.uniprot_generic_ptm_accession ?\n#\n_pdbe_chem_comp_drugbank_
details.comp_id SIA\n_pdbe_chem_comp_drugbank_details.drugbank_id
DB03721\n_pdbe_chem_comp_drugbank_details.type 'small
molecule'\n_pdbe_chem_comp_drugbank_details.name 'N-acetyl-alpha-
neuraminic acid'\n_pdbe_chem_comp_drugbank_details.description\n'An N-
acyl derivative of neuraminic acid. N-acetylneuraminic acid occurs in
many polysaccharides, glycoproteins, and glycolipids in animals and
bacteria. (From Dorland, 28th ed,
p1518)'\n_pdbe_chem_comp_drugbank_details.cas_number 21646-00-
4\n_pdbe_chem_comp_drugbank_details.mechanism_of_action ?\n#\nloop_\n_
pdbe_chem_comp_synonyms.comp_id\n_pdbe_chem_comp_synonyms.name\n_pdbe_
chem_comp_synonyms.provenance\n_pdbe_chem_comp_synonyms.type\nSIA 'N-
acetylneuraminic acid' wwPDB ?\nSIA 'sialic acid' wwPDB ?\nSIA 'alpha-
sialic acid' wwPDB ?\nSIA 'O-SIALIC ACID' wwPDB ?\nSIA 'N-Acetyl-
alpha-D-neuraminic acid' DrugBank ?\nSIA 'O-sialic acid'

DrugBank ?\nSIA 'α-Neu5Ac'
DrugBank ?\n#\n_pdbe_chem_comp_drugbank_classification.comp_id SIA\n_pdbe_chem_comp_drugbank_classification.drugbank_id DB03721\n_pdbe_chem_comp_drugbank_classification.parent 'N-acylneuraminic acids'\n_pdbe_chem_comp_drugbank_classification.kingdom 'Organic compounds'\n_pdbe_chem_comp_drugbank_classification.class 'Organooxygen compounds'\n_pdbe_chem_comp_drugbank_classification.superclass 'Organic oxygen compounds'\n_pdbe_chem_comp_drugbank_classification.description\n'This compound belongs to the class of organic compounds known as n-acylneuraminic acids. These are neuraminic acids carrying an N-acyl substituent.'\n#\nloop_\n_pdbe_chem_comp_drugbank_targets.comp_id\n_pdbe_chem_comp_drugbank_targets.drugbank_id\n_pdbe_chem_comp_drugbank_targets.name\n_pdbe_chem_comp_drugbank_targets.organism\n_pdbe_chem_comp_drugbank_targets.uniprot_id\n_pdbe_chem_comp_drugbank_targets.pharmacologically_active\n_pdbe_chem_comp_drugbank_targets.ordinal\nSIA DB03721 P-selectin Humans P16109 yes 1\nSIA DB03721 E-selectin Humans P16581 yes 2\nSIA DB03721 'Liver carboxylesterase 1' Humans P23141 yes 3\nSIA DB03721 '3-deoxy-manno-octulosonate cytidylyltransferase' 'Escherichia coli' P42216 unknown 4\nSIA DB03721 'Tetanus toxin' 'Clostridium tetani (strain Massachusetts / E88)' P04958 unknown 5\nSIA DB03721 'Cholera enterotoxin subunit B' 'Vibrio cholerae serotype O1 (strain ATCC 39315 / El Tor Inaba N16961)' P01556 unknown 6\nSIA DB03721 'Botulinum neurotoxin type B' 'Clostridium botulinum' P10844 unknown 7\nSIA DB03721 'Mannose-binding protein C' Humans P11226 unknown 8\nSIA DB03721 Lithostathine-1-alpha Humans P05451 unknown 9\nSIA DB03721 Endo-N-acetylneuraminidase 'Enterobacteria phage K1F' Q04830 unknown 10\nSIA DB03721 'Enterotoxin type B' 'Staphylococcus aureus' P01552 unknown 11\nSIA DB03721 Neuraminidase 'Influenza A virus (strain A/Tern/Australia/G70C/1975 H11N9)' P03472 unknown 12\nSIA DB03721 Hemagglutinin-neuraminidase NDV P32884 unknown 13\nSIA DB03721 Fiber 'Human adenovirus 19' Q64822 unknown 14\nSIA DB03721 Sialoadhesin Humans Q9BZZ2 unknown 15\nSIA DB03721 Zinc-alpha-2-glycoprotein Humans P25311 unknown 16\nSIA DB03721 'Capsid protein VP1' MPyV P49302 unknown 17\nSIA DB03721 Fiber 'Human adenovirus D37' Q64823 unknown 18\n#\nloop_\n_software.name\n_software.version\n_software.description\nrdkit 2023.09.6 'Core functionality.'\npdbeccdutils 0.8.6 'Wrapper to provide 2D templates and molecular fragments.'\n#\nloop_\n_pdbe_chem_comp_atom_depiction.comp_id\n_pdbe_chem_comp_atom_depiction.atom_id\n_pdbe_chem_comp_atom_depiction.element\n_pdbe_chem_comp_atom_depiction.model_Cartn_x\n_pdbe_chem_comp_atom_depiction.model_Cartn_y\n_pdbe_chem_comp_atom_depiction.pdbx_ordinal\nSIA C1 C 5.654 -3.375 1\nSIA C2 C 6.404 -2.076 2\nSIA C3 C 5.104 -1.326 3\nSIA C4 C 5.104 0.174 4\nSIA C5 C 6.404 0.924 5\nSIA C6 C 7.702 0.174 6\nSIA C7 C 9.002 0.924 7\nSIA C8 C 10.301 0.174 8\nSIA C9 C 11.600 0.924 9\nSIA C10 C 5.104 3.174 10\nSIA C11 C 3.805 2.424 11\nSIA N5 N 6.404 2.424 12\nSIA O1A O 4.154 -3.375 13\nSIA O1B O 6.404 -4.674 14\nSIA O4 O 3.805 0.924 16\nSIA O6 O 7.702 -1.326 17\nSIA O7 O 9.002 2.424 18\nSIA O8 O 10.301 -1.326 19\nSIA O9 O 12.899 0.174 20\nSIA O10 O 5.104 4.674

21\n#\nloop_\n_pdbe_chem_comp_bond_depiction.comp_id\n_pdbe_chem_comp_bond_depiction.atom_id_1\n_pdbe_chem_comp_bond_depiction.atom_id_2\n_pdbe_chem_comp_bond_depiction.value_order\n_pdbe_chem_comp_bond_depiction.bond_dir\n_pdbe_chem_comp_bond_depiction.pdbx_ordinal\nSIA C1 C2 SINGLE NONE 1\nSIA C1 O1A DOUBLE NONE 2\nSIA C1 O1B SINGLE NONE 3\nSIA C2 C3 SINGLE NONE 4\nSIA C2 O6 SINGLE NONE 6\nSIA C3 C4 SINGLE NONE 7\nSIA C4 C5 SINGLE NONE 8\nSIA C4 O4 SINGLE BEGINDASH 9\nSIA C5 C6 SINGLE NONE 10\nSIA C5 N5 SINGLE BEGINWEDGE 11\nSIA C6 C7 SINGLE NONE 12\nSIA C6 O6 SINGLE BEGINDASH 13\nSIA C7 C8 SINGLE NONE 14\nSIA C7 O7 SINGLE BEGINDASH 15\nSIA C8 C9 SINGLE NONE 16\nSIA C8 O8 SINGLE BEGINWEDGE 17\nSIA C9 O9 SINGLE NONE 18\nSIA C10 C11 SINGLE NONE 19\nSIA C10 N5 SINGLE NONE 20\nSIA C10 O10 DOUBLE NONE 21\n#\nloop_\n_pdbe_chem_comp_substructure.comp_id\n_pdbe_chem_comp_substructure.substructure_name\n_pdbe_chem_comp_substructure.id\n_pdbe_chem_comp_substructure.substructure_type\n_pdbe_chem_comp_substructure.substructure_smiles\n_pdbe_chem_comp_substructure.substructure_inchis\n_pdbe_chem_comp_substructure.substructure_inchikeys\nSIA MurckoScaffold S1 scaffold C1CCOCC1 InChI=1S/C5H10O/c1-2-4-6-5-3-1/h1-5H2 DHXVGJBLRPWPCS-UHFFFAOYSA-N\nSIA amide F1 fragment CC(N)=O InChI=1S/C2H5NO/c1-2(3)4/h1H3,(H2,3,4) DLFVBJFMPXGRIB-UHFFFAOYSA-N\nSIA pyranose F2 fragment OC1CCCCO1 InChI=1S/C5H10O2/c6-5-3-1-2-4-7-5/h5-6H,1-4H2 CELWCAITJAEQNL-UHFFFAOYSA-N\n#\nloop_\n_pdbe_chem_comp_substructure_mapping.comp_id\n_pdbe_chem_comp_substructure_mapping.atom_id\n_pdbe_chem_comp_substructure_mapping.substructure_id\n_pdbe_chem_comp_substructure_mapping.substructure_ordinal\nSIA C2 S1 1\nSIA C3 S1 1\nSIA C4 S1 1\nSIA C5 S1 1\nSIA C6 S1 1\nSIA O6 S1 1\nSIA N5 F1 1\nSIA C10 F1 1\nSIA O10 F1 1\nSIA C11 F1 1\nSIA C5 F2 1\nSIA C6 F2 1\nSIA O6 F2 1\nSIA C2 F2 1\nSIA C3 F2 1\nSIA C4 F2 1\n#\n_pdbe_chem_comp_rdkit_properties.comp_id SIA\n_pdbe_chem_comp_rdkit_properties.exactmw 309.106\n_pdbe_chem_comp_rdkit_properties.amw 309.271\n_pdbe_chem_comp_rdkit_properties.lipinskiHBA 10\n_pdbe_chem_comp_rdkit_properties.lipinskiHBD 7\n_pdbe_chem_comp_rdkit_properties.NumRotatableBonds 11\n_pdbe_chem_comp_rdkit_properties.NumHBD 7\n_pdbe_chem_comp_rdkit_properties.NumHBA 9\n_pdbe_chem_comp_rdkit_properties.NumHeavyAtoms 21\n_pdbe_chem_comp_rdkit_properties.NumAtoms 40\n_pdbe_chem_comp_rdkit_properties.NumHeteroatoms 10\n_pdbe_chem_comp_rdkit_properties.NumAmideBonds 1\n_pdbe_chem_comp_rdkit_properties.FractionCSP3 0.818\n_pdbe_chem_comp_rdkit_properties.NumRings 1\n_pdbe_chem_comp_rdkit_properties.NumAromaticRings 0\n_pdbe_chem_comp_rdkit_properties.NumAliphaticRings 1\n_pdbe_chem_comp_rdkit_properties.NumSaturatedRings 1\n_pdbe_chem_comp_rdkit_properties.NumHeterocycles 1\n_pdbe_chem_comp_rdkit_properties.NumAromaticHeterocycles 0\n_pdbe_chem_comp_rdkit_properties.NumSaturatedHeterocycles 1\n_pdbe_chem_comp_rdkit_properties.NumAliphaticHeterocycles 1\n_pdbe_chem_comp_rdkit_properties.NumSpiroAtoms 0\n_pdbe_chem_comp_rdkit_properties.NumBridgeheadAtoms 0\n_pdbe_chem_comp_rdkit_properties.NumAtomStereoCenters

6\n_pdbe_chem_comp_rdkit_properties.NumUnspecifiedAtomStereoCenters
0\n_pdbe_chem_comp_rdkit_properties.labuteASA
146.407\n_pdbe_chem_comp_rdkit_properties.tpsa
176.780\n_pdbe_chem_comp_rdkit_properties.CrippenClogP -
3.872\n_pdbe_chem_comp_rdkit_properties.CrippenMR
64.787\n_pdbe_chem_comp_rdkit_properties.chi0v
9.621\n_pdbe_chem_comp_rdkit_properties.chi1v
4.738\n_pdbe_chem_comp_rdkit_properties.chi2v
1.941\n_pdbe_chem_comp_rdkit_properties.chi3v
1.941\n_pdbe_chem_comp_rdkit_properties.chi4v
1.128\n_pdbe_chem_comp_rdkit_properties.chi0n
28.621\n_pdbe_chem_comp_rdkit_properties.chi1n
13.635\n_pdbe_chem_comp_rdkit_properties.chi2n
1.941\n_pdbe_chem_comp_rdkit_properties.chi3n
1.941\n_pdbe_chem_comp_rdkit_properties.chi4n
1.128\n_pdbe_chem_comp_rdkit_properties.hallKierAlpha -
1.300\n_pdbe_chem_comp_rdkit_properties.kappa1
4.600\n_pdbe_chem_comp_rdkit_properties.kappa2
6.642\n_pdbe_chem_comp_rdkit_properties.kappa3
3.872\n_pdbe_chem_comp_rdkit_properties.Phi
1.455\n#\nloop_\n_pdbe_chem_comp_external_mappings.comp_id\n_pdbe_chem
_comp_external_mappings.source\n_pdbe_chem_comp_external_mappings.reso
urce\n_pdbe_chem_comp_external_mappings.resource_id\nSIA UniChem
ChEMBL CHEMBL1234621\nSIA UniChem DrugBank DB03721\nSIA UniChem ChEBI
49026\nSIA UniChem ZINC ZINC000004081651\nSIA UniChem fdasrs
04A90EXP8V\nSIA UniChem HMDB HMDB0000773\nSIA UniChem Nikkaji
J614.853K\nSIA UniChem MetaboLights MTBLC49026\nSIA UniChem BRENDA
141715\nSIA UniChem BRENDA 233672\nSIA UniChem BRENDA 6105\nSIA
UniChem BRENDA 84245\nSIA UniChem BRENDA 85625\nSIA UniChem 'Probes
And Drugs' PD041137\nSIA UniChem PubChem 444885\nSIA UniChem
eMolecules 474793\nSIA UniChem SureChEMBL SCHEMBL79085\nSIA UniChem
'PubChem TPHARMA' 14776495\nSIA UniChem 'PubChem TPHARMA'
15395566\n#\nloop_\n_pdbe_chem_comp_rdkit_conformer.comp_id\n_pdbe_che
m_comp_rdkit_conformer.atom_id\n_pdbe_chem_comp_rdkit_conformer.Cartn_
x_rdkit\n_pdbe_chem_comp_rdkit_conformer.Cartn_y_rdkit\n_pdbe_chem_com
p_rdkit_conformer.Cartn_z_rdkit\n_pdbe_chem_comp_rdkit_conformer.rdkit
_method\n_pdbe_chem_comp_rdkit_conformer.rdkit_ordinal\nSIA C1 -1.164
-2.811 1.058 ETKDGv3 1\nSIA C2 -1.666 -1.928 -0.070 ETKDGv3 2\nSIA C3
-2.639 -0.809 0.442 ETKDGv3 3\nSIA C4 -2.546 0.454 -0.428 ETKDGv3
4\nSIA C5 -1.079 0.960 -0.528 ETKDGv3 5\nSIA C6 -0.071 -0.181 -0.219
ETKDGv3 6\nSIA C7 1.341 0.133 -0.777 ETKDGv3 7\nSIA C8 2.378 -0.960 -
0.400 ETKDGv3 8\nSIA C9 3.769 -0.651 -0.979 ETKDGv3 9\nSIA C10 -0.223
3.295 0.052 ETKDGv3 10\nSIA C11 0.035 4.333 1.094 ETKDGv3 11\nSIA N5 -
0.868 2.066 0.414 ETKDGv3 12\nSIA O1A -0.936 -2.335 2.203 ETKDGv3
13\nSIA O1B -0.875 -4.149 0.806 ETKDGv3 14\nSIA O4 -3.104 0.224 -1.700
ETKDGv3 16\nSIA O6 -0.537 -1.404 -0.765 ETKDGv3 17\nSIA O7 1.298 0.320
-2.172 ETKDGv3 18\nSIA O8 2.484 -1.066 0.998 ETKDGv3 19\nSIA O9 4.246
0.587 -0.524 ETKDGv3 20\nSIA O10 0.157 3.497 -1.133 ETKDGv3 21\nSIA
H32 -2.392 -0.510 1.484 ETKDGv3 22\nSIA H31 -3.691 -1.171 0.465
ETKDGv3 23\nSIA H4 -3.171 1.239 0.053 ETKDGv3 24\nSIA H5 -0.925 1.324
-1.568 ETKDGv3 25\nSIA H6 0.030 -0.270 0.887 ETKDGv3 26\nSIA H7 1.678
1.089 -0.320 ETKDGv3 27\nSIA H8 2.053 -1.935 -0.838 ETKDGv3 28\nSIA

H92 3.719 -0.629 -2.088 ETKDGv3 29\nSIA H91 4.478 -1.464 -0.703 ETKDGv3 30\nSIA H111 0.843 3.984 1.770 ETKDGv3 31\nSIA H113 -0.888 4.508 1.686 ETKDGv3 32\nSIA H112 0.344 5.291 0.624 ETKDGv3 33\nSIA HN5 -1.113 1.913 1.419 ETKDGv3 34\nSIA HO1B -0.511 -4.749 1.536 ETKDGv3 35\nSIA HO4 -2.492 -0.372 -2.205 ETKDGv3 37\nSIA HO7 1.040 -0.545 -2.585 ETKDGv3 38\nSIA HO8 1.886 -1.804 1.285 ETKDGv3 39\nSIA HO9 4.592 0.441 0.395 ETKDGv3 40\n#\n"
}

Template 11. G2S2F (α2,3-sia)

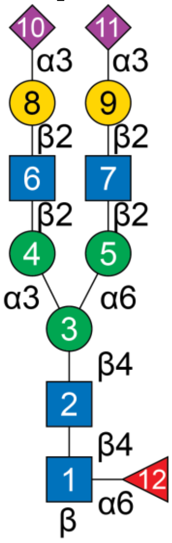

{
"name": "g2s2_33f_usersia",
"modelSeeds": [
1
],
"sequences": [
{
"ligand": {
"ccdCodes": [
"NAG", "NAG", "BMA", "MAN", "MAN", "NAG", "NAG", "GAL",
"GAL", "SIA2", "SIA2", "FUC"
],
"id": "NG"
}
}
],
"dialect": "alphafold3",
"version": 2,
"bondedAtomPairs": [
[["NG",1,"O4"],["NG",2,"C1"]],
[["NG",2,"O4"],["NG",3,"C1"]],
[["NG",3,"O3"],["NG",4,"C1"]],
[["NG",3,"O6"],["NG",5,"C1"]],
[["NG",4,"O2"],["NG",6,"C1"]],
[["NG",5,"O2"],["NG",7,"C1"]],
[["NG",6,"O4"],["NG",8,"C1"]],
[["NG",7,"O4"],["NG",9,"C1"]],
[["NG",8,"O3"],["NG",10,"C2"]],
[["NG",9,"O3"],["NG",11,"C2"]],
[["NG",1,"O6"],["NG",12,"C1"]]
],
"userCCD": "data_SIA2\n#\n_chem_comp.id SIA\n_chem_comp.name 'N-acetyl-alpha-neuraminic acid'\n_chem_comp.type 'D-saccharide, alpha linking'\n_chem_comp.pdbx_type ATOMS\n_chem_comp.formula 'C11 H19 N

O9'\n_chem_comp.mon_nstd_parent_comp_id ?\n_chem_comp.pdbx_synonyms 'N-acetylneuraminic acid; sialic acid; alpha-sialic acid; O-SIALIC ACID'\n_chem_comp.pdbx_formal_charge 0\n_chem_comp.pdbx_initial_date 1999-07-08\n_chem_comp.pdbx_modified_date 2024-09-27\n_chem_comp.pdbx_ambiguous_flag N\n_chem_comp.pdbx_release_status REL\n_chem_comp.pdbx_replaced_by ?\n_chem_comp.pdbx_replaces NAN\n_chem_comp.formula_weight 309.270\n_chem_comp.one_letter_code ?\n_chem_comp.three_letter_code SIA\n_chem_comp.pdbx_model_coordinates_details ?\n_chem_comp.pdbx_model_coordinates_missing_flag N\n_chem_comp.pdbx_ideal_coordinates_details ?\n_chem_comp.pdbx_ideal_coordinates_missing_flag N\n_chem_comp.pdbx_model_coordinates_db_code ?\n_chem_comp.pdbx_subcomponent_list ?\n_chem_comp.pdbx_processing_site EBI\n_chem_comp.pdbx_pcm Y\n#\nloop_\n_pdbx_chem_comp_synonyms.ordinal\n_pdbx_chem_comp_synonyms.comp_id\n_pdbx_chem_comp_synonyms.name\n_pdbx_chem_comp_synonyms.provenance\n_pdbx_chem_comp_synonyms.type\n1 SIA 'N-acetylneuraminic acid' PDB ?\n2 SIA 'sialic acid' PDB ?\n3 SIA 'alpha-sialic acid' PDB ?\n4 SIA 'O-SIALIC ACID' PDB ?\n#\nloop_\n_chem_comp_atom.comp_id\n_chem_comp_atom.atom_id\n_chem_comp_atom.alt_atom_id\n_chem_comp_atom.type_symbol\n_chem_comp_atom.charge\n_chem_comp_atom.pdbx_align\n_chem_comp_atom.pdbx_aromatic_flag\n_chem_comp_atom.pdbx_leaving_atom_flag\n_chem_comp_atom.pdbx_stereo_config\n_chem_comp_atom.pdbx_backbone_atom_flag\n_chem_comp_atom.pdbx_n_terminal_atom_flag\n_chem_comp_atom.pdbx_c_terminal_atom_flag\n_chem_comp_atom.model_Cartn_x\n_chem_comp_atom.model_Cartn_y\n_chem_comp_atom.model_Cartn_z\n_chem_comp_atom.pdbx_model_Cartn_x_ideal\n_chem_comp_atom.pdbx_model_Cartn_y_ideal\n_chem_comp_atom.pdbx_model_Cartn_z_ideal\n_chem_comp_atom.pdbx_component_atom_id\n_chem_comp_atom.pdbx_component_comp_id\n_chem_comp_atom.pdbx_ordinal\nSIA C1 C1 C 0 1 N N N N N N -2.196 58.872 -5.981 -2.502 -0.832 0.174 C1 SIA 1\nSIA C2 C2 C 0 1 N N R N N N -1.870 58.021 -7.211 -2.171 0.628 0.342 C2 SIA 2\nSIA C3 C3 C 0 1 N N N N N N -0.844 56.899 -7.306 -1.789 0.898 1.800 C3 SIA 3\nSIA C4 C4 C 0 1 N N S N N N -1.157 55.904 -8.413 -0.586 0.023 2.171 C4 SIA 4\nSIA C5 C5 C 0 1 N N R N N N -2.015 56.516 -9.517 0.529 0.264 1.148 C5 SIA 5\nSIA C6 C6 C 0 1 N N R N N N -3.352 56.956 -8.912 -0.026 0.043 -0.259 C6 SIA 6\nSIA C7 C7 C 0 1 N N R N N N -4.224 57.698 -9.942 1.088 0.251 -1.286 C7 SIA 7\nSIA C8 C8 C 0 1 N N R N N N -5.571 58.131 -9.360 0.535 0.021 -2.694 C8 SIA 8\nSIA C9 C9 C 0 1 N N N N N N -6.601 58.674 -10.381 1.650 0.229 -3.721 C9 SIA 9\nSIA C10 C10 C 0 1 N N N N N N -1.897 55.374 -11.759 2.632 -0.329 2.226 C10 SIA 10\nSIA C11 C11 C 0 1 N N N N N N -2.200 54.057 -12.454 3.763 -1.292 2.478 C11 SIA 11\nSIA N5 N5 N 0 1 N N N N N N -2.202 55.444 -10.478 1.629 -0.671 1.394 N5 SIA 12\nSIA O1A O1A O 0 1 N N N N N N -1.289 58.815 -5.130 -2.191 -1.408 -0.841 O1A SIA 13\nSIA O1B O1B O 0 1 N N N N N N -3.210 59.504 -5.631 -3.141 -1.493 1.152 O1B SIA 14\nSIA O4 O4 O 0 1 N N N N N N 0.072 55.523 -8.986 -0.123 0.370 3.478 O4 SIA 16\nSIA O6 O6 O 0 1 N N N N N N -3.149 57.908 -7.847 -1.082 0.968 -0.513 O6 SIA 17\nSIA O7 O7 O 0 1 N N N N N N -3.594 58.883 -10.402 1.588 1.586 -1.183 O7 SIA 18\nSIA O8 O8 O 0 1 N N N N N N -6.119 56.946 -8.828 0.035 -1.313 -2.797 O8 SIA 19\nSIA O9 O9 O 0 1 N N N N N N -6.931 57.687 -11.346

1.133 0.014 -5.035 O9 SIA 20\nSIA O10 O10 O 0 1 N N N N N N -1.423
56.357 -12.331 2.624 0.753 2.772 O10 SIA 21\nSIA H32 H31 H 0 1 N N N N
N N -0.702 56.484 -6.300 -2.631 0.655 2.448 H32 SIA 22\nSIA H31 H32 H
0 1 N N N N N N 0.120 57.408 -7.182 -1.526 1.949 1.919 H31 SIA 23\nSIA
H4 H4 H 0 1 N N N N N N -1.651 55.060 -7.897 -0.878 -1.026 2.153 H4
SIA 24\nSIA H5 H5 H 0 1 N N N N N N -1.506 57.375 -9.979 0.893 1.287
1.240 H5 SIA 25\nSIA H6 H6 H 0 1 N N N N N N -3.850 56.075 -8.492 -
0.408 -0.973 -0.341 H6 SIA 26\nSIA H7 H7 H 0 1 N N N N N N -4.339
57.176 -10.907 1.896 -0.454 -1.093 H7 SIA 27\nSIA H8 H8 H 0 1 N N N N
N N -5.473 58.871 -8.553 -0.272 0.728 -2.887 H8 SIA 28\nSIA H92 H91 H
0 1 N N N N N N -6.054 59.459 -10.925 2.031 1.247 -3.642 H92 SIA
29\nSIA H91 H92 H 0 1 N N N N N N -7.587 59.029 -10.055 2.457 -0.476 -
3.528 H91 SIA 30\nSIA H111 H111 H 0 0 N N N N N N -3.215 53.728 -
12.207 4.474 -0.844 3.172 H111 SIA 31\nSIA H113 H112 H 0 0 N N N N N N
-1.550 53.279 -12.033 3.368 -2.213 2.907 H113 SIA 32\nSIA H112 H113 H
0 0 N N N N N N -2.005 54.041 -13.531 4.266 -1.516 1.537 H112 SIA
33\nSIA HN5 HN5 H 0 1 N N N N N N -2.566 54.658 -10.003 1.635 -1.538
0.957 HN5 SIA 34\nSIA HO1B HOB1 H 0 0 N N N N N N -3.412 60.032 -4.867
-3.353 -2.430 1.044 HO1B SIA 35\nSIA HO4 HO4 H 0 1 N Y N N N N 0.427
54.801 -8.430 -0.854 0.203 4.087 HO4 SIA 37\nSIA HO7 HO7 H 0 1 N Y N N
N N -3.109 58.884 -9.548 0.844 2.177 -1.360 HO7 SIA 38\nSIA HO8 HO8 H
0 1 N Y N N N N -7.071 57.051 -8.962 0.779 -1.904 -2.620 HO8 SIA
39\nSIA HO9 HO9 H 0 1 N Y N N N N -6.783 56.885 -10.808 1.866 0.155 -
5.650 HO9 SIA
40\n#\nloop_\n_chem_comp_bond.comp_id\n_chem_comp_bond.atom_id_1\n_che
m_comp_bond.atom_id_2\n_chem_comp_bond.value_order\n_chem_comp_bond.pd
bx_aromatic_flag\n_chem_comp_bond.pdbx_stereo_config\n_chem_comp_bond.
pdbx_ordinal\nSIA C1 C2 SING N N 1\nSIA C1 O1A DOUB N N 2\nSIA C1 O1B
SING N N 3\nSIA C2 C3 SING N N 4\nSIA C2 O6 SING N N 6\nSIA C3 C4 SING
N N 7\nSIA C3 H32 SING N N 8\nSIA C3 H31 SING N N 9\nSIA C4 C5 SING N
N 10\nSIA C4 O4 SING N N 11\nSIA C4 H4 SING N N 12\nSIA C5 C6 SING N N
13\nSIA C5 N5 SING N N 14\nSIA C5 H5 SING N N 15\nSIA C6 C7 SING N N
16\nSIA C6 O6 SING N N 17\nSIA C6 H6 SING N N 18\nSIA C7 C8 SING N N
19\nSIA C7 O7 SING N N 20\nSIA C7 H7 SING N N 21\nSIA C8 C9 SING N N
22\nSIA C8 O8 SING N N 23\nSIA C8 H8 SING N N 24\nSIA C9 O9 SING N N
25\nSIA C9 H92 SING N N 26\nSIA C9 H91 SING N N 27\nSIA C10 C11 SING N
N 28\nSIA C10 N5 SING N N 29\nSIA C10 O10 DOUB N N 30\nSIA C11 H111
SING N N 31\nSIA C11 H113 SING N N 32\nSIA C11 H112 SING N N 33\nSIA
N5 HN5 SING N N 34\nSIA O1B HO1B SING N N 35\nSIA O4 HO4 SING N N
37\nSIA O7 HO7 SING N N 38\nSIA O8 HO8 SING N N 39\nSIA O9 HO9 SING N
N
40\n#\nloop_\n_pdbx_chem_comp_descriptor.comp_id\n_pdbx_chem_comp_desc
riptor.type\n_pdbx_chem_comp_descriptor.program\n_pdbx_chem_comp_descr
iptor.program_version\n_pdbx_chem_comp_descriptor.descriptor\nSIA
SMILES ACDLabs 10.04 'O=C(O)C1(O)OC(C(O)C(O)CO)C(NC(=O)C)C(O)C1'\nSIA
SMILES_CANONICAL CACTVS 3.341
'CC(=O)N[C@@H]1[C@@H](O)C[C@@](O)(O[C@H]1[C@H](O)[C@H](O)CO)C(O)=O'\nS
IA SMILES CACTVS 3.341
'CC(=O)N[CH]1[CH](O)C[C](O)(O[CH]1[CH](O)[CH](O)CO)C(O)=O'\nSIA
SMILES_CANONICAL 'OpenEye OEToolkits' 1.5.0
'CC(=O)N[C@@H]1[C@H](C[C@@](O[C@H]1[C@@H]([C@@H](CO)O)O)(C(=O)O)O)O'\n
SIA SMILES 'OpenEye OEToolkits' 1.5.0

'CC(=O)NC1C(CC(OC1C(C(CO)O)O)(C(=O)O)O)O'\nSIA InChI InChI 1.03
'InChI=1S/C11H19NO9/c1-4(14)12-7-5(15)2-11(20,10(18)19)21-
9(7)8(17)6(16)3-13/h5-9,13,15-17,20H,2-
3H2,1H3,(H,12,14)(H,18,19)/t5-,6+,7+,8+,9+,11+/m0/s1'\nSIA InChIKey
InChI 1.03 SQVRNKJHWKZAKO-YRMXFSIDSA-
N\n#\nloop_\n_pdbx_chem_comp_identifier.comp_id\n_pdbx_chem_comp_ident
ifier.type\n_pdbx_chem_comp_identifier.program\n_pdbx_chem_comp_identi
fier.program_version\n_pdbx_chem_comp_identifier.identifier\nSIA
'SYSTEMATIC NAME' ACDLabs 10.04 '5-(acetylamino)-3,5-dideoxy-D-
glycero-alpha-D-galacto-non-2-ulopyranosonic acid'\nSIA 'SYSTEMATIC
NAME' 'OpenEye OEToolkits' 1.5.0 '(2R,4S,5R,6R)-5-acetamido-2,4-
dihydroxy-6-[(1R,2R)-1,2,3-trihydroxypropyl]oxane-2-carboxylic
acid'\nSIA 'CONDENSED IUPAC CARBOHYDRATE SYMBOL' GMML 1.0
DNeup5Aca\nSIA 'COMMON NAME' GMML 1.0 'N-acetyl-a-D-neuraminic
acid'\nSIA 'IUPAC CARBOHYDRATE SYMBOL' PDB-CARE 1.0 a-D-Neup5Ac\nSIA
'SNFG CARBOHYDRATE SYMBOL' GMML 1.0
Neu5Ac\n#\nloop_\n_pdbx_chem_comp_feature.comp_id\n_pdbx_chem_comp_fea
ture.type\n_pdbx_chem_comp_feature.value\n_pdbx_chem_comp_feature.sour
ce\n_pdbx_chem_comp_feature.support\nSIA 'CARBOHYDRATE ISOMER' D
PDB ?\nSIA 'CARBOHYDRATE RING' pyranose PDB ?\nSIA 'CARBOHYDRATE
ANOMER' alpha PDB ?\nSIA 'CARBOHYDRATE PRIMARY CARBONYL GROUP' ketose
PDB ?\n#\nloop_\n_pdbx_chem_comp_audit.comp_id\n_pdbx_chem_comp_audit.
action_type\n_pdbx_chem_comp_audit.date\n_pdbx_chem_comp_audit.process
ing_site\nSIA 'Create component' 1999-07-08 EBI\nSIA 'Modify
descriptor' 2011-06-04 RCSB\nSIA 'Other modification' 2019-08-12
RCSB\nSIA 'Other modification' 2019-12-19 RCSB\nSIA 'Other
modification' 2020-07-03 RCSB\nSIA 'Modify name' 2020-07-17 RCSB\nSIA
'Modify synonyms' 2020-07-17 RCSB\nSIA 'Modify atom id' 2020-07-17
RCSB\nSIA 'Modify component atom id' 2020-07-17 RCSB\nSIA 'Modify PCM'
2024-09-27 PDBE\n#\n_pdbx_chem_comp_pcm.pcm_id
1\n_pdbx_chem_comp_pcm.comp_id
SIA\n_pdbx_chem_comp_pcm.modified_residue_id
THR\n_pdbx_chem_comp_pcm.type None\n_pdbx_chem_comp_pcm.category
Carbohydrate\n_pdbx_chem_comp_pcm.position 'Amino-acid side
chain'\n_pdbx_chem_comp_pcm.polypeptide_position 'Any
position'\n_pdbx_chem_comp_pcm.comp_id_linking_atom
C2\n_pdbx_chem_comp_pcm.modified_residue_id_linking_atom
OG1\n_pdbx_chem_comp_pcm.uniprot_specific_ptm_accession ?\n_pdbx_chem_
comp_pcm.uniprot_generic_ptm_accession ?\n#\n_pdbe_chem_comp_drugbank_
details.comp_id SIA\n_pdbe_chem_comp_drugbank_details.drugbank_id
DB03721\n_pdbe_chem_comp_drugbank_details.type 'small
molecule'\n_pdbe_chem_comp_drugbank_details.name 'N-acetyl-alpha-
neuraminic acid'\n_pdbe_chem_comp_drugbank_details.description\n'An N-
acyl derivative of neuraminic acid. N-acetylneuraminic acid occurs in
many polysaccharides, glycoproteins, and glycolipids in animals and
bacteria. (From Dorland, 28th ed,
p1518)'\n_pdbe_chem_comp_drugbank_details.cas_number 21646-00-
4\n_pdbe_chem_comp_drugbank_details.mechanism_of_action ?\n#\nloop_\n_
pdbe_chem_comp_synonyms.comp_id\n_pdbe_chem_comp_synonyms.name\n_pdbe_
chem_comp_synonyms.provenance\n_pdbe_chem_comp_synonyms.type\nSIA 'N-
acetylneuraminic acid' wwPDB ?\nSIA 'sialic acid' wwPDB ?\nSIA 'alpha-
sialic acid' wwPDB ?\nSIA 'O-SIALIC ACID' wwPDB ?\nSIA 'N-Acetyl-

alpha-D-neuraminic acid' DrugBank ?\nSIA 'O-sialic acid'
DrugBank ?\nSIA 'α-Neu5Ac'
DrugBank ?\n#\n_pdbe_chem_comp_drugbank_classification.comp_id
SIA\n_pdbe_chem_comp_drugbank_classification.drugbank_id
DB03721\n_pdbe_chem_comp_drugbank_classification.parent 'N-
acylneuraminic acids'\n_pdbe_chem_comp_drugbank_classification.kingdom
'Organic compounds'\n_pdbe_chem_comp_drugbank_classification.class
'Organooxygen
compounds'\n_pdbe_chem_comp_drugbank_classification.superclass
'Organic oxygen
compounds'\n_pdbe_chem_comp_drugbank_classification.description\n'This
compound belongs to the class of organic compounds known as n-
acylneuraminic acids. These are neuraminic acids carrying an N-acyl
substituent.'\n#\nloop_\n_pdbe_chem_comp_drugbank_targets.comp_id\n_pd
be_chem_comp_drugbank_targets.drugbank_id\n_pdbe_chem_comp_drugbank_ta
rgets.name\n_pdbe_chem_comp_drugbank_targets.organism\n_pdbe_chem_comp
_drugbank_targets.uniprot_id\n_pdbe_chem_comp_drugbank_targets.pharmac
ologically_active\n_pdbe_chem_comp_drugbank_targets.ordinal\nSIA
DB03721 P-selectin Humans P16109 yes 1\nSIA DB03721 E-selectin Humans
P16581 yes 2\nSIA DB03721 'Liver carboxylesterase 1' Humans P23141 yes
3\nSIA DB03721 '3-deoxy-manno-octulosonate cytidylyltransferase'
'Escherichia coli' P42216 unknown 4\nSIA DB03721 'Tetanus toxin'
'Clostridium tetani (strain Massachusetts / E88)' P04958 unknown
5\nSIA DB03721 'Cholera enterotoxin subunit B' 'Vibrio cholerae
serotype O1 (strain ATCC 39315 / El Tor Inaba N16961)' P01556 unknown
6\nSIA DB03721 'Botulinum neurotoxin type B' 'Clostridium botulinum'
P10844 unknown 7\nSIA DB03721 'Mannose-binding protein C' Humans
P11226 unknown 8\nSIA DB03721 Lithostathine-1-alpha Humans P05451
unknown 9\nSIA DB03721 Endo-N-acetylneuraminidase 'Enterobacteria
phage K1F' Q04830 unknown 10\nSIA DB03721 'Enterotoxin type B'
'Staphylococcus aureus' P01552 unknown 11\nSIA DB03721 Neuraminidase
'Influenza A virus (strain A/Tern/Australia/G70C/1975 H11N9)' P03472
unknown 12\nSIA DB03721 Hemagglutinin-neuraminidase NDV P32884 unknown
13\nSIA DB03721 Fiber 'Human adenovirus 19' Q64822 unknown 14\nSIA
DB03721 Sialoadhesin Humans Q9BZZ2 unknown 15\nSIA DB03721 Zinc-alpha-
2-glycoprotein Humans P25311 unknown 16\nSIA DB03721 'Capsid protein
VP1' MPyV P49302 unknown 17\nSIA DB03721 Fiber 'Human adenovirus D37'
Q64823 unknown
18\n#\nloop_\n_software.name\n_software.version\n_software.description
\nrdkit 2023.09.6 'Core functionality.'\npdbeccdutils 0.8.6 'Wrapper
to provide 2D templates and molecular
fragments.'\n#\nloop_\n_pdbe_chem_comp_atom_depiction.comp_id\n_pdbe_c
hem_comp_atom_depiction.atom_id\n_pdbe_chem_comp_atom_depiction.elemen
t\n_pdbe_chem_comp_atom_depiction.model_Cartn_x\n_pdbe_chem_comp_atom_
depiction.model_Cartn_y\n_pdbe_chem_comp_atom_depiction.pdbx_ordinal\n
SIA C1 C 5.654 -3.375 1\nSIA C2 C 6.404 -2.076 2\nSIA C3 C 5.104 -
1.326 3\nSIA C4 C 5.104 0.174 4\nSIA C5 C 6.404 0.924 5\nSIA C6 C
7.702 0.174 6\nSIA C7 C 9.002 0.924 7\nSIA C8 C 10.301 0.174 8\nSIA C9
C 11.600 0.924 9\nSIA C10 C 5.104 3.174 10\nSIA C11 C 3.805 2.424
11\nSIA N5 N 6.404 2.424 12\nSIA O1A O 4.154 -3.375 13\nSIA O1B O
6.404 -4.674 14\nSIA O4 O 3.805 0.924 16\nSIA O6 O 7.702 -1.326
17\nSIA O7 O 9.002 2.424 18\nSIA O8 O 10.301 -1.326 19\nSIA O9 O

12.899 0.174 20\nSIA O10 O 5.104 4.674
21\n#\nloop_\n_pdbe_chem_comp_bond_depiction.comp_id\n_pdbe_chem_comp_
bond_depiction.atom_id_1\n_pdbe_chem_comp_bond_depiction.atom_id_2\n_p
dbe_chem_comp_bond_depiction.value_order\n_pdbe_chem_comp_bond_depicti
on.bond_dir\n_pdbe_chem_comp_bond_depiction.pdbx_ordinal\nSIA C1 C2
SINGLE NONE 1\nSIA C1 O1A DOUBLE NONE 2\nSIA C1 O1B SINGLE NONE 3\nSIA
C2 C3 SINGLE NONE 4\nSIA C2 O6 SINGLE NONE 6\nSIA C3 C4 SINGLE NONE
7\nSIA C4 C5 SINGLE NONE 8\nSIA C4 O4 SINGLE BEGINDASH 9\nSIA C5 C6
SINGLE NONE 10\nSIA C5 N5 SINGLE BEGINWEDGE 11\nSIA C6 C7 SINGLE NONE
12\nSIA C6 O6 SINGLE BEGINDASH 13\nSIA C7 C8 SINGLE NONE 14\nSIA C7 O7
SINGLE BEGINDASH 15\nSIA C8 C9 SINGLE NONE 16\nSIA C8 O8 SINGLE
BEGINWEDGE 17\nSIA C9 O9 SINGLE NONE 18\nSIA C10 C11 SINGLE NONE
19\nSIA C10 N5 SINGLE NONE 20\nSIA C10 O10 DOUBLE NONE
21\n#\nloop_\n_pdbe_chem_comp_substructure.comp_id\n_pdbe_chem_comp_su
bstructure.substructure_name\n_pdbe_chem_comp_substructure.id\n_pdbe_c
hem_comp_substructure.substructure_type\n_pdbe_chem_comp_substructure.
substructure_smiles\n_pdbe_chem_comp_substructure.substructure_inchis\
n_pdbe_chem_comp_substructure.substructure_inchikeys\nSIA
MurckoScaffold S1 scaffold C1CCOCC1 InChI=1S/C5H10O/c1-2-4-6-5-3-1/h1-
5H2 DHXVGJBLRPWPCS-UHFFFAOYSA-N\nSIA amide F1 fragment CC(N)=O
InChI=1S/C2H5NO/c1-2(3)4/h1H3,(H2,3,4) DLFVBJFMPXGRIB-UHFFFAOYSA-
N\nSIA pyranose F2 fragment OC1CCCCO1 InChI=1S/C5H10O2/c6-5-3-1-2-4-7-
5/h5-6H,1-4H2 CELWCAITJAEQNL-UHFFFAOYSA-
N\n#\nloop_\n_pdbe_chem_comp_substructure_mapping.comp_id\n_pdbe_chem_
comp_substructure_mapping.atom_id\n_pdbe_chem_comp_substructure_mappin
g.substructure_id\n_pdbe_chem_comp_substructure_mapping.substructure_o
rdinal\nSIA C2 S1 1\nSIA C3 S1 1\nSIA C4 S1 1\nSIA C5 S1 1\nSIA C6 S1
1\nSIA O6 S1 1\nSIA N5 F1 1\nSIA C10 F1 1\nSIA O10 F1 1\nSIA C11 F1
1\nSIA C5 F2 1\nSIA C6 F2 1\nSIA O6 F2 1\nSIA C2 F2 1\nSIA C3 F2
1\nSIA C4 F2 1\n#\n_pdbe_chem_comp_rdkit_properties.comp_id
SIA\n_pdbe_chem_comp_rdkit_properties.exactmw
309.106\n_pdbe_chem_comp_rdkit_properties.amw
309.271\n_pdbe_chem_comp_rdkit_properties.lipinskiHBA
10\n_pdbe_chem_comp_rdkit_properties.lipinskiHBD
7\n_pdbe_chem_comp_rdkit_properties.NumRotatableBonds
11\n_pdbe_chem_comp_rdkit_properties.NumHBD
7\n_pdbe_chem_comp_rdkit_properties.NumHBA
9\n_pdbe_chem_comp_rdkit_properties.NumHeavyAtoms
21\n_pdbe_chem_comp_rdkit_properties.NumAtoms
40\n_pdbe_chem_comp_rdkit_properties.NumHeteroatoms
10\n_pdbe_chem_comp_rdkit_properties.NumAmideBonds
1\n_pdbe_chem_comp_rdkit_properties.FractionCSP3
0.818\n_pdbe_chem_comp_rdkit_properties.NumRings
1\n_pdbe_chem_comp_rdkit_properties.NumAromaticRings
0\n_pdbe_chem_comp_rdkit_properties.NumAliphaticRings
1\n_pdbe_chem_comp_rdkit_properties.NumSaturatedRings
1\n_pdbe_chem_comp_rdkit_properties.NumHeterocycles
1\n_pdbe_chem_comp_rdkit_properties.NumAromaticHeterocycles
0\n_pdbe_chem_comp_rdkit_properties.NumSaturatedHeterocycles
1\n_pdbe_chem_comp_rdkit_properties.NumAliphaticHeterocycles
1\n_pdbe_chem_comp_rdkit_properties.NumSpiroAtoms
0\n_pdbe_chem_comp_rdkit_properties.NumBridgeheadAtoms

0\n_pdbe_chem_comp_rdkit_properties.NumAtomStereoCenters
6\n_pdbe_chem_comp_rdkit_properties.NumUnspecifiedAtomStereoCenters
0\n_pdbe_chem_comp_rdkit_properties.labuteASA
146.407\n_pdbe_chem_comp_rdkit_properties.tpsa
176.780\n_pdbe_chem_comp_rdkit_properties.CrippenClogP -
3.872\n_pdbe_chem_comp_rdkit_properties.CrippenMR
64.787\n_pdbe_chem_comp_rdkit_properties.chi0v
9.621\n_pdbe_chem_comp_rdkit_properties.chi1v
4.738\n_pdbe_chem_comp_rdkit_properties.chi2v
1.941\n_pdbe_chem_comp_rdkit_properties.chi3v
1.941\n_pdbe_chem_comp_rdkit_properties.chi4v
1.128\n_pdbe_chem_comp_rdkit_properties.chi0n
28.621\n_pdbe_chem_comp_rdkit_properties.chi1n
13.635\n_pdbe_chem_comp_rdkit_properties.chi2n
1.941\n_pdbe_chem_comp_rdkit_properties.chi3n
1.941\n_pdbe_chem_comp_rdkit_properties.chi4n
1.128\n_pdbe_chem_comp_rdkit_properties.hallKierAlpha -
1.300\n_pdbe_chem_comp_rdkit_properties.kappa1
4.600\n_pdbe_chem_comp_rdkit_properties.kappa2
6.642\n_pdbe_chem_comp_rdkit_properties.kappa3
3.872\n_pdbe_chem_comp_rdkit_properties.Phi
1.455\n#\nloop_\n_pdbe_chem_comp_external_mappings.comp_id\n_pdbe_chem
_comp_external_mappings.source\n_pdbe_chem_comp_external_mappings.reso
urce\n_pdbe_chem_comp_external_mappings.resource_id\nSIA UniChem
ChEMBL CHEMBL1234621\nSIA UniChem DrugBank DB03721\nSIA UniChem ChEBI
49026\nSIA UniChem ZINC ZINC000004081651\nSIA UniChem fdasrs
04A90EXP8V\nSIA UniChem HMDB HMDB0000773\nSIA UniChem Nikkaji
J614.853K\nSIA UniChem MetaboLights MTBLC49026\nSIA UniChem BRENDA
141715\nSIA UniChem BRENDA 233672\nSIA UniChem BRENDA 6105\nSIA
UniChem BRENDA 84245\nSIA UniChem BRENDA 85625\nSIA UniChem 'Probes
And Drugs' PD041137\nSIA UniChem PubChem 444885\nSIA UniChem
eMolecules 474793\nSIA UniChem SureChEMBL SCHEMBL79085\nSIA UniChem
'PubChem TPHARMA' 14776495\nSIA UniChem 'PubChem TPHARMA'
15395566\n#\nloop_\n_pdbe_chem_comp_rdkit_conformer.comp_id\n_pdbe_che
m_comp_rdkit_conformer.atom_id\n_pdbe_chem_comp_rdkit_conformer.Cartn_
x_rdkit\n_pdbe_chem_comp_rdkit_conformer.Cartn_y_rdkit\n_pdbe_chem_com
p_rdkit_conformer.Cartn_z_rdkit\n_pdbe_chem_comp_rdkit_conformer.rdkit
_method\n_pdbe_chem_comp_rdkit_conformer.rdkit_ordinal\nSIA C1 -1.164
-2.811 1.058 ETKDGv3 1\nSIA C2 -1.666 -1.928 -0.070 ETKDGv3 2\nSIA C3
-2.639 -0.809 0.442 ETKDGv3 3\nSIA C4 -2.546 0.454 -0.428 ETKDGv3
4\nSIA C5 -1.079 0.960 -0.528 ETKDGv3 5\nSIA C6 -0.071 -0.181 -0.219
ETKDGv3 6\nSIA C7 1.341 0.133 -0.777 ETKDGv3 7\nSIA C8 2.378 -0.960 -
0.400 ETKDGv3 8\nSIA C9 3.769 -0.651 -0.979 ETKDGv3 9\nSIA C10 -0.223
3.295 0.052 ETKDGv3 10\nSIA C11 0.035 4.333 1.094 ETKDGv3 11\nSIA N5 -
0.868 2.066 0.414 ETKDGv3 12\nSIA O1A -0.936 -2.335 2.203 ETKDGv3
13\nSIA O1B -0.875 -4.149 0.806 ETKDGv3 14\nSIA O4 -3.104 0.224 -1.700
ETKDGv3 16\nSIA O6 -0.537 -1.404 -0.765 ETKDGv3 17\nSIA O7 1.298 0.320
-2.172 ETKDGv3 18\nSIA O8 2.484 -1.066 0.998 ETKDGv3 19\nSIA O9 4.246
0.587 -0.524 ETKDGv3 20\nSIA O10 0.157 3.497 -1.133 ETKDGv3 21\nSIA
H32 -2.392 -0.510 1.484 ETKDGv3 22\nSIA H31 -3.691 -1.171 0.465
ETKDGv3 23\nSIA H4 -3.171 1.239 0.053 ETKDGv3 24\nSIA H5 -0.925 1.324
-1.568 ETKDGv3 25\nSIA H6 0.030 -0.270 0.887 ETKDGv3 26\nSIA H7 1.678

1.089 -0.320 ETKDGv3 27\nSIA H8 2.053 -1.935 -0.838 ETKDGv3 28\nSIA H92 3.719 -0.629 -2.088 ETKDGv3 29\nSIA H91 4.478 -1.464 -0.703 ETKDGv3 30\nSIA H111 0.843 3.984 1.770 ETKDGv3 31\nSIA H113 -0.888 4.508 1.686 ETKDGv3 32\nSIA H112 0.344 5.291 0.624 ETKDGv3 33\nSIA HN5 -1.113 1.913 1.419 ETKDGv3 34\nSIA HO1B -0.511 -4.749 1.536 ETKDGv3 35\nSIA HO4 -2.492 -0.372 -2.205 ETKDGv3 37\nSIA HO7 1.040 -0.545 -2.585 ETKDGv3 38\nSIA HO8 1.886 -1.804 1.285 ETKDGv3 39\nSIA HO9 4.592 0.441 0.395 ETKDGv3 40\n#\n"
}

Template 12. G2S2F (α2,6-sia)

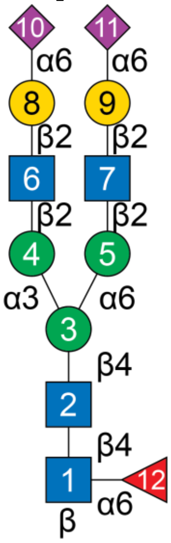

{
"name": "g2s2_66f_usersia",
"modelSeeds": [
1
],
"sequences": [
{
"ligand": {
"ccdCodes": [
"NAG", "NAG", "BMA", "MAN", "MAN", "NAG", "NAG", "GAL",
"GAL", "SIA2", "SIA2", "FUC"
],
"id": "NG"
}
}
],
"dialect": "alphafold3",
"version": 2,
"bondedAtomPairs": [
[["NG",1,"O4"],["NG",2,"C1"]],
[["NG",2,"O4"],["NG",3,"C1"]],
[["NG",3,"O3"],["NG",4,"C1"]],
[["NG",3,"O6"],["NG",5,"C1"]],
[["NG",4,"O2"],["NG",6,"C1"]],
[["NG",5,"O2"],["NG",7,"C1"]],
[["NG",6,"O4"],["NG",8,"C1"]],
[["NG",7,"O4"],["NG",9,"C1"]],
[["NG",8,"O6"],["NG",10,"C2"]],
[["NG",9,"O6"],["NG",11,"C2"]],
[["NG",1,"O6"],["NG",12,"C1"]]
],
"userCCD": "data_SIA2\n#\n_chem_comp.id SIA\n_chem_comp.name 'N-acetyl-alpha-neuraminic acid'\n_chem_comp.type 'D-saccharide, alpha linking'\n_chem_comp.pdbx_type ATOMS\n_chem_comp.formula 'C11 H19 N

O9'\n_chem_comp.mon_nstd_parent_comp_id ?\n_chem_comp.pdbx_synonyms 'N-acetylneuraminic acid; sialic acid; alpha-sialic acid; O-SIALIC ACID'\n_chem_comp.pdbx_formal_charge 0\n_chem_comp.pdbx_initial_date 1999-07-08\n_chem_comp.pdbx_modified_date 2024-09-27\n_chem_comp.pdbx_ambiguous_flag N\n_chem_comp.pdbx_release_status REL\n_chem_comp.pdbx_replaced_by ?\n_chem_comp.pdbx_replaces NAN\n_chem_comp.formula_weight 309.270\n_chem_comp.one_letter_code ?\n_chem_comp.three_letter_code SIA\n_chem_comp.pdbx_model_coordinates_details ?\n_chem_comp.pdbx_model_coordinates_missing_flag N\n_chem_comp.pdbx_ideal_coordinates_details ?\n_chem_comp.pdbx_ideal_coordinates_missing_flag N\n_chem_comp.pdbx_model_coordinates_db_code ?\n_chem_comp.pdbx_subcomponent_list ?\n_chem_comp.pdbx_processing_site EBI\n_chem_comp.pdbx_pcm Y\n#\nloop_\n_pdbx_chem_comp_synonyms.ordinal\n_pdbx_chem_comp_synonyms.comp_id\n_pdbx_chem_comp_synonyms.name\n_pdbx_chem_comp_synonyms.provenance\n_pdbx_chem_comp_synonyms.type\n1 SIA 'N-acetylneuraminic acid' PDB ?\n2 SIA 'sialic acid' PDB ?\n3 SIA 'alpha-sialic acid' PDB ?\n4 SIA 'O-SIALIC ACID' PDB ?\n#\nloop_\n_chem_comp_atom.comp_id\n_chem_comp_atom.atom_id\n_chem_comp_atom.alt_atom_id\n_chem_comp_atom.type_symbol\n_chem_comp_atom.charge\n_chem_comp_atom.pdbx_align\n_chem_comp_atom.pdbx_aromatic_flag\n_chem_comp_atom.pdbx_leaving_atom_flag\n_chem_comp_atom.pdbx_stereo_config\n_chem_comp_atom.pdbx_backbone_atom_flag\n_chem_comp_atom.pdbx_n_terminal_atom_flag\n_chem_comp_atom.pdbx_c_terminal_atom_flag\n_chem_comp_atom.model_Cartn_x\n_chem_comp_atom.model_Cartn_y\n_chem_comp_atom.model_Cartn_z\n_chem_comp_atom.pdbx_model_Cartn_x_ideal\n_chem_comp_atom.pdbx_model_Cartn_y_ideal\n_chem_comp_atom.pdbx_model_Cartn_z_ideal\n_chem_comp_atom.pdbx_component_atom_id\n_chem_comp_atom.pdbx_component_comp_id\n_chem_comp_atom.pdbx_ordinal\nSIA C1 C1 C 0 1 N N N N N N -2.196 58.872 -5.981 -2.502 -0.832 0.174 C1 SIA 1\nSIA C2 C2 C 0 1 N N R N N N -1.870 58.021 -7.211 -2.171 0.628 0.342 C2 SIA 2\nSIA C3 C3 C 0 1 N N N N N N -0.844 56.899 -7.306 -1.789 0.898 1.800 C3 SIA 3\nSIA C4 C4 C 0 1 N N S N N N -1.157 55.904 -8.413 -0.586 0.023 2.171 C4 SIA 4\nSIA C5 C5 C 0 1 N N R N N N -2.015 56.516 -9.517 0.529 0.264 1.148 C5 SIA 5\nSIA C6 C6 C 0 1 N N R N N N -3.352 56.956 -8.912 -0.026 0.043 -0.259 C6 SIA 6\nSIA C7 C7 C 0 1 N N R N N N -4.224 57.698 -9.942 1.088 0.251 -1.286 C7 SIA 7\nSIA C8 C8 C 0 1 N N R N N N -5.571 58.131 -9.360 0.535 0.021 -2.694 C8 SIA 8\nSIA C9 C9 C 0 1 N N N N N N -6.601 58.674 -10.381 1.650 0.229 -3.721 C9 SIA 9\nSIA C10 C10 C 0 1 N N N N N N -1.897 55.374 -11.759 2.632 -0.329 2.226 C10 SIA 10\nSIA C11 C11 C 0 1 N N N N N N -2.200 54.057 -12.454 3.763 -1.292 2.478 C11 SIA 11\nSIA N5 N5 N 0 1 N N N N N N -2.202 55.444 -10.478 1.629 -0.671 1.394 N5 SIA 12\nSIA O1A O1A O 0 1 N N N N N N -1.289 58.815 -5.130 -2.191 -1.408 -0.841 O1A SIA 13\nSIA O1B O1B O 0 1 N N N N N N -3.210 59.504 -5.631 -3.141 -1.493 1.152 O1B SIA 14\nSIA O4 O4 O 0 1 N N N N N N 0.072 55.523 -8.986 -0.123 0.370 3.478 O4 SIA 16\nSIA O6 O6 O 0 1 N N N N N N -3.149 57.908 -7.847 -1.082 0.968 -0.513 O6 SIA 17\nSIA O7 O7 O 0 1 N N N N N N -3.594 58.883 -10.402 1.588 1.586 -1.183 O7 SIA 18\nSIA O8 O8 O 0 1 N N N N N N -6.119 56.946 -8.828 0.035 -1.313 -2.797 O8 SIA 19\nSIA O9 O9 O 0 1 N N N N N N -6.931 57.687 -11.346

1.133 0.014 -5.035 O9 SIA 20\nSIA O10 O10 O 0 1 N N N N N N -1.423 56.357 -12.331 2.624 0.753 2.772 O10 SIA 21\nSIA H32 H31 H 0 1 N N N N N N -0.702 56.484 -6.300 -2.631 0.655 2.448 H32 SIA 22\nSIA H31 H32 H 0 1 N N N N N N 0.120 57.408 -7.182 -1.526 1.949 1.919 H31 SIA 23\nSIA H4 H4 H 0 1 N N N N N N -1.651 55.060 -7.897 -0.878 -1.026 2.153 H4 SIA 24\nSIA H5 H5 H 0 1 N N N N N N -1.506 57.375 -9.979 0.893 1.287 1.240 H5 SIA 25\nSIA H6 H6 H 0 1 N N N N N N -3.850 56.075 -8.492 -0.408 -0.973 -0.341 H6 SIA 26\nSIA H7 H7 H 0 1 N N N N N N -4.339 57.176 -10.907 1.896 -0.454 -1.093 H7 SIA 27\nSIA H8 H8 H 0 1 N N N N N N -5.473 58.871 -8.553 -0.272 0.728 -2.887 H8 SIA 28\nSIA H92 H91 H 0 1 N N N N N N -6.054 59.459 -10.925 2.031 1.247 -3.642 H92 SIA 29\nSIA H91 H92 H 0 1 N N N N N N -7.587 59.029 -10.055 2.457 -0.476 -3.528 H91 SIA 30\nSIA H111 H111 H 0 0 N N N N N N -3.215 53.728 -12.207 4.474 -0.844 3.172 H111 SIA 31\nSIA H113 H112 H 0 0 N N N N N N -1.550 53.279 -12.033 3.368 -2.213 2.907 H113 SIA 32\nSIA H112 H113 H 0 0 N N N N N N -2.005 54.041 -13.531 4.266 -1.516 1.537 H112 SIA 33\nSIA HN5 HN5 H 0 1 N N N N N N -2.566 54.658 -10.003 1.635 -1.538 0.957 HN5 SIA 34\nSIA HO1B HOB1 H 0 0 N N N N N N -3.412 60.032 -4.867 -3.353 -2.430 1.044 HO1B SIA 35\nSIA HO4 HO4 H 0 1 N Y N N N N 0.427 54.801 -8.430 -0.854 0.203 4.087 HO4 SIA 37\nSIA HO7 HO7 H 0 1 N Y N N N N -3.109 58.884 -9.548 0.844 2.177 -1.360 HO7 SIA 38\nSIA HO8 HO8 H 0 1 N Y N N N N -7.071 57.051 -8.962 0.779 -1.904 -2.620 HO8 SIA 39\nSIA HO9 HO9 H 0 1 N Y N N N N -6.783 56.885 -10.808 1.866 0.155 -5.650 HO9 SIA
40\n#\nloop_\n_chem_comp_bond.comp_id\n_chem_comp_bond.atom_id_1\n_chem_comp_bond.atom_id_2\n_chem_comp_bond.value_order\n_chem_comp_bond.pdbx_aromatic_flag\n_chem_comp_bond.pdbx_stereo_config\n_chem_comp_bond.pdbx_ordinal\nSIA C1 C2 SING N N 1\nSIA C1 O1A DOUB N N 2\nSIA C1 O1B SING N N 3\nSIA C2 C3 SING N N 4\nSIA C2 O6 SING N N 6\nSIA C3 C4 SING N N 7\nSIA C3 H32 SING N N 8\nSIA C3 H31 SING N N 9\nSIA C4 C5 SING N N 10\nSIA C4 O4 SING N N 11\nSIA C4 H4 SING N N 12\nSIA C5 C6 SING N N 13\nSIA C5 N5 SING N N 14\nSIA C5 H5 SING N N 15\nSIA C6 C7 SING N N 16\nSIA C6 O6 SING N N 17\nSIA C6 H6 SING N N 18\nSIA C7 C8 SING N N 19\nSIA C7 O7 SING N N 20\nSIA C7 H7 SING N N 21\nSIA C8 C9 SING N N 22\nSIA C8 O8 SING N N 23\nSIA C8 H8 SING N N 24\nSIA C9 O9 SING N N 25\nSIA C9 H92 SING N N 26\nSIA C9 H91 SING N N 27\nSIA C10 C11 SING N N 28\nSIA C10 N5 SING N N 29\nSIA C10 O10 DOUB N N 30\nSIA C11 H111 SING N N 31\nSIA C11 H113 SING N N 32\nSIA C11 H112 SING N N 33\nSIA N5 HN5 SING N N 34\nSIA O1B HO1B SING N N 35\nSIA O4 HO4 SING N N 37\nSIA O7 HO7 SING N N 38\nSIA O8 HO8 SING N N 39\nSIA O9 HO9 SING N N
40\n#\nloop_\n_pdbx_chem_comp_descriptor.comp_id\n_pdbx_chem_comp_descriptor.type\n_pdbx_chem_comp_descriptor.program\n_pdbx_chem_comp_descriptor.program_version\n_pdbx_chem_comp_descriptor.descriptor\nSIA SMILES ACDLabs 10.04 'O=C(O)C1(O)OC(C(O)C(O)CO)C(NC(=O)C)C(O)C1'\nSIA SMILES_CANONICAL CACTVS 3.341
'CC(=O)N[C@@H]1[C@@H](O)C[C@@](O)(O[C@H]1[C@H](O)[C@H](O)CO)C(O)=O'\nSIA SMILES CACTVS 3.341
'CC(=O)N[CH]1[CH](O)C[C](O)(O[CH]1[CH](O)[CH](O)CO)C(O)=O'\nSIA SMILES_CANONICAL 'OpenEye OEToolkits' 1.5.0
'CC(=O)N[C@@H]1[C@H](C[C@@](O[C@H]1[C@@H]([C@@H](CO)O)O)(C(=O)O)O)O'\nSIA SMILES 'OpenEye OEToolkits' 1.5.0

'CC(=O)NC1C(CC(OC1C(C(CO)O)O)(C(=O)O)O)O'\nSIA InChI InChI 1.03
'InChI=1S/C11H19NO9/c1-4(14)12-7-5(15)2-11(20,10(18)19)21-
9(7)8(17)6(16)3-13/h5-9,13,15-17,20H,2-
3H2,1H3,(H,12,14)(H,18,19)/t5-,6+,7+,8+,9+,11+/m0/s1'\nSIA InChIKey
InChI 1.03 SQVRNKJHWKZAKO-YRMXFSIDSA-
N\n#\nloop_\n_pdbx_chem_comp_identifier.comp_id\n_pdbx_chem_comp_ident
ifier.type\n_pdbx_chem_comp_identifier.program\n_pdbx_chem_comp_identi
fier.program_version\n_pdbx_chem_comp_identifier.identifier\nSIA
'SYSTEMATIC NAME' ACDLabs 10.04 '5-(acetylamino)-3,5-dideoxy-D-
glycero-alpha-D-galacto-non-2-ulopyranosonic acid'\nSIA 'SYSTEMATIC
NAME' 'OpenEye OEToolkits' 1.5.0 '(2R,4S,5R,6R)-5-acetamido-2,4-
dihydroxy-6-[(1R,2R)-1,2,3-trihydroxypropyl]oxane-2-carboxylic
acid'\nSIA 'CONDENSED IUPAC CARBOHYDRATE SYMBOL' GMML 1.0
DNeup5Aca\nSIA 'COMMON NAME' GMML 1.0 'N-acetyl-a-D-neuraminic
acid'\nSIA 'IUPAC CARBOHYDRATE SYMBOL' PDB-CARE 1.0 a-D-Neup5Ac\nSIA
'SNFG CARBOHYDRATE SYMBOL' GMML 1.0
Neu5Ac\n#\nloop_\n_pdbx_chem_comp_feature.comp_id\n_pdbx_chem_comp_fea
ture.type\n_pdbx_chem_comp_feature.value\n_pdbx_chem_comp_feature.sour
ce\n_pdbx_chem_comp_feature.support\nSIA 'CARBOHYDRATE ISOMER' D
PDB ?\nSIA 'CARBOHYDRATE RING' pyranose PDB ?\nSIA 'CARBOHYDRATE
ANOMER' alpha PDB ?\nSIA 'CARBOHYDRATE PRIMARY CARBONYL GROUP' ketose
PDB ?\n#\nloop_\n_pdbx_chem_comp_audit.comp_id\n_pdbx_chem_comp_audit.
action_type\n_pdbx_chem_comp_audit.date\n_pdbx_chem_comp_audit.process
ing_site\nSIA 'Create component' 1999-07-08 EBI\nSIA 'Modify
descriptor' 2011-06-04 RCSB\nSIA 'Other modification' 2019-08-12
RCSB\nSIA 'Other modification' 2019-12-19 RCSB\nSIA 'Other
modification' 2020-07-03 RCSB\nSIA 'Modify name' 2020-07-17 RCSB\nSIA
'Modify synonyms' 2020-07-17 RCSB\nSIA 'Modify atom id' 2020-07-17
RCSB\nSIA 'Modify component atom id' 2020-07-17 RCSB\nSIA 'Modify PCM'
2024-09-27 PDBE\n#\n_pdbx_chem_comp_pcm.pcm_id
1\n_pdbx_chem_comp_pcm.comp_id
SIA\n_pdbx_chem_comp_pcm.modified_residue_id
THR\n_pdbx_chem_comp_pcm.type None\n_pdbx_chem_comp_pcm.category
Carbohydrate\n_pdbx_chem_comp_pcm.position 'Amino-acid side
chain'\n_pdbx_chem_comp_pcm.polypeptide_position 'Any
position'\n_pdbx_chem_comp_pcm.comp_id_linking_atom
C2\n_pdbx_chem_comp_pcm.modified_residue_id_linking_atom
OG1\n_pdbx_chem_comp_pcm.uniprot_specific_ptm_accession ?\n_pdbx_chem_
comp_pcm.uniprot_generic_ptm_accession ?\n#\n_pdbe_chem_comp_drugbank_
details.comp_id SIA\n_pdbe_chem_comp_drugbank_details.drugbank_id
DB03721\n_pdbe_chem_comp_drugbank_details.type 'small
molecule'\n_pdbe_chem_comp_drugbank_details.name 'N-acetyl-alpha-
neuraminic acid'\n_pdbe_chem_comp_drugbank_details.description\n'An N-
acyl derivative of neuraminic acid. N-acetylneuraminic acid occurs in
many polysaccharides, glycoproteins, and glycolipids in animals and
bacteria. (From Dorland, 28th ed,
p1518)'\n_pdbe_chem_comp_drugbank_details.cas_number 21646-00-
4\n_pdbe_chem_comp_drugbank_details.mechanism_of_action ?\n#\nloop_\n_
pdbe_chem_comp_synonyms.comp_id\n_pdbe_chem_comp_synonyms.name\n_pdbe_
chem_comp_synonyms.provenance\n_pdbe_chem_comp_synonyms.type\nSIA 'N-
acetylneuraminic acid' wwPDB ?\nSIA 'sialic acid' wwPDB ?\nSIA 'alpha-
sialic acid' wwPDB ?\nSIA 'O-SIALIC ACID' wwPDB ?\nSIA 'N-Acetyl-

alpha-D-neuraminic acid' DrugBank ?\nSIA 'O-sialic acid'
DrugBank ?\nSIA 'α-Neu5Ac'
DrugBank ?\n#\n_pdbe_chem_comp_drugbank_classification.comp_id
SIA\n_pdbe_chem_comp_drugbank_classification.drugbank_id
DB03721\n_pdbe_chem_comp_drugbank_classification.parent 'N-
acylneuraminic acids'\n_pdbe_chem_comp_drugbank_classification.kingdom
'Organic compounds'\n_pdbe_chem_comp_drugbank_classification.class
'Organooxygen
compounds'\n_pdbe_chem_comp_drugbank_classification.superclass
'Organic oxygen
compounds'\n_pdbe_chem_comp_drugbank_classification.description\n'This
compound belongs to the class of organic compounds known as n-
acylneuraminic acids. These are neuraminic acids carrying an N-acyl
substituent.'\n#\nloop_\n_pdbe_chem_comp_drugbank_targets.comp_id\n_pd
be_chem_comp_drugbank_targets.drugbank_id\n_pdbe_chem_comp_drugbank_ta
rgets.name\n_pdbe_chem_comp_drugbank_targets.organism\n_pdbe_chem_comp
_drugbank_targets.uniprot_id\n_pdbe_chem_comp_drugbank_targets.pharmac
ologically_active\n_pdbe_chem_comp_drugbank_targets.ordinal\nSIA
DB03721 P-selectin Humans P16109 yes 1\nSIA DB03721 E-selectin Humans
P16581 yes 2\nSIA DB03721 'Liver carboxylesterase 1' Humans P23141 yes
3\nSIA DB03721 '3-deoxy-manno-octulosonate cytidylyltransferase'
'Escherichia coli' P42216 unknown 4\nSIA DB03721 'Tetanus toxin'
'Clostridium tetani (strain Massachusetts / E88)' P04958 unknown
5\nSIA DB03721 'Cholera enterotoxin subunit B' 'Vibrio cholerae
serotype O1 (strain ATCC 39315 / El Tor Inaba N16961)' P01556 unknown
6\nSIA DB03721 'Botulinum neurotoxin type B' 'Clostridium botulinum'
P10844 unknown 7\nSIA DB03721 'Mannose-binding protein C' Humans
P11226 unknown 8\nSIA DB03721 Lithostathine-1-alpha Humans P05451
unknown 9\nSIA DB03721 Endo-N-acetylneuraminidase 'Enterobacteria
phage K1F' Q04830 unknown 10\nSIA DB03721 'Enterotoxin type B'
'Staphylococcus aureus' P01552 unknown 11\nSIA DB03721 Neuraminidase
'Influenza A virus (strain A/Tern/Australia/G70C/1975 H11N9)' P03472
unknown 12\nSIA DB03721 Hemagglutinin-neuraminidase NDV P32884 unknown
13\nSIA DB03721 Fiber 'Human adenovirus 19' Q64822 unknown 14\nSIA
DB03721 Sialoadhesin Humans Q9BZZ2 unknown 15\nSIA DB03721 Zinc-alpha-
2-glycoprotein Humans P25311 unknown 16\nSIA DB03721 'Capsid protein
VP1' MPyV P49302 unknown 17\nSIA DB03721 Fiber 'Human adenovirus D37'
Q64823 unknown
18\n#\nloop_\n_software.name\n_software.version\n_software.description
\nrdkit 2023.09.6 'Core functionality.'\npdbeccdutils 0.8.6 'Wrapper
to provide 2D templates and molecular
fragments.'\n#\nloop_\n_pdbe_chem_comp_atom_depiction.comp_id\n_pdbe_c
hem_comp_atom_depiction.atom_id\n_pdbe_chem_comp_atom_depiction.elemen
t\n_pdbe_chem_comp_atom_depiction.model_Cartn_x\n_pdbe_chem_comp_atom_
depiction.model_Cartn_y\n_pdbe_chem_comp_atom_depiction.pdbx_ordinal\n
SIA C1 C 5.654 -3.375 1\nSIA C2 C 6.404 -2.076 2\nSIA C3 C 5.104 -
1.326 3\nSIA C4 C 5.104 0.174 4\nSIA C5 C 6.404 0.924 5\nSIA C6 C
7.702 0.174 6\nSIA C7 C 9.002 0.924 7\nSIA C8 C 10.301 0.174 8\nSIA C9
C 11.600 0.924 9\nSIA C10 C 5.104 3.174 10\nSIA C11 C 3.805 2.424
11\nSIA N5 N 6.404 2.424 12\nSIA O1A O 4.154 -3.375 13\nSIA O1B O
6.404 -4.674 14\nSIA O4 O 3.805 0.924 16\nSIA O6 O 7.702 -1.326
17\nSIA O7 O 9.002 2.424 18\nSIA O8 O 10.301 -1.326 19\nSIA O9 O

12.899 0.174 20\nSIA O10 O 5.104 4.674
21\n#\nloop_\n_pdbe_chem_comp_bond_depiction.comp_id\n_pdbe_chem_comp_
bond_depiction.atom_id_1\n_pdbe_chem_comp_bond_depiction.atom_id_2\n_p
dbe_chem_comp_bond_depiction.value_order\n_pdbe_chem_comp_bond_depicti
on.bond_dir\n_pdbe_chem_comp_bond_depiction.pdbx_ordinal\nSIA C1 C2
SINGLE NONE 1\nSIA C1 O1A DOUBLE NONE 2\nSIA C1 O1B SINGLE NONE 3\nSIA
C2 C3 SINGLE NONE 4\nSIA C2 O6 SINGLE NONE 6\nSIA C3 C4 SINGLE NONE
7\nSIA C4 C5 SINGLE NONE 8\nSIA C4 O4 SINGLE BEGINDASH 9\nSIA C5 C6
SINGLE NONE 10\nSIA C5 N5 SINGLE BEGINWEDGE 11\nSIA C6 C7 SINGLE NONE
12\nSIA C6 O6 SINGLE BEGINDASH 13\nSIA C7 C8 SINGLE NONE 14\nSIA C7 O7
SINGLE BEGINDASH 15\nSIA C8 C9 SINGLE NONE 16\nSIA C8 O8 SINGLE
BEGINWEDGE 17\nSIA C9 O9 SINGLE NONE 18\nSIA C10 C11 SINGLE NONE
19\nSIA C10 N5 SINGLE NONE 20\nSIA C10 O10 DOUBLE NONE
21\n#\nloop_\n_pdbe_chem_comp_substructure.comp_id\n_pdbe_chem_comp_su
bstructure.substructure_name\n_pdbe_chem_comp_substructure.id\n_pdbe_c
hem_comp_substructure.substructure_type\n_pdbe_chem_comp_substructure.
substructure_smiles\n_pdbe_chem_comp_substructure.substructure_inchis\
n_pdbe_chem_comp_substructure.substructure_inchikeys\nSIA
MurckoScaffold S1 scaffold C1CCOCC1 InChI=1S/C5H10O/c1-2-4-6-5-3-1/h1-
5H2 DHXVGJBLRPWPCS-UHFFFAOYSA-N\nSIA amide F1 fragment CC(N)=O
InChI=1S/C2H5NO/c1-2(3)4/h1H3,(H2,3,4) DLFVBJFMPXGRIB-UHFFFAOYSA-
N\nSIA pyranose F2 fragment OC1CCCCO1 InChI=1S/C5H10O2/c6-5-3-1-2-4-7-
5/h5-6H,1-4H2 CELWCAITJAEQNL-UHFFFAOYSA-
N\n#\nloop_\n_pdbe_chem_comp_substructure_mapping.comp_id\n_pdbe_chem_
comp_substructure_mapping.atom_id\n_pdbe_chem_comp_substructure_mappin
g.substructure_id\n_pdbe_chem_comp_substructure_mapping.substructure_o
rdinal\nSIA C2 S1 1\nSIA C3 S1 1\nSIA C4 S1 1\nSIA C5 S1 1\nSIA C6 S1
1\nSIA O6 S1 1\nSIA N5 F1 1\nSIA C10 F1 1\nSIA O10 F1 1\nSIA C11 F1
1\nSIA C5 F2 1\nSIA C6 F2 1\nSIA O6 F2 1\nSIA C2 F2 1\nSIA C3 F2
1\nSIA C4 F2 1\n#\n_pdbe_chem_comp_rdkit_properties.comp_id
SIA\n_pdbe_chem_comp_rdkit_properties.exactmw
309.106\n_pdbe_chem_comp_rdkit_properties.amw
309.271\n_pdbe_chem_comp_rdkit_properties.lipinskiHBA
10\n_pdbe_chem_comp_rdkit_properties.lipinskiHBD
7\n_pdbe_chem_comp_rdkit_properties.NumRotatableBonds
11\n_pdbe_chem_comp_rdkit_properties.NumHBD
7\n_pdbe_chem_comp_rdkit_properties.NumHBA
9\n_pdbe_chem_comp_rdkit_properties.NumHeavyAtoms
21\n_pdbe_chem_comp_rdkit_properties.NumAtoms
40\n_pdbe_chem_comp_rdkit_properties.NumHeteroatoms
10\n_pdbe_chem_comp_rdkit_properties.NumAmideBonds
1\n_pdbe_chem_comp_rdkit_properties.FractionCSP3
0.818\n_pdbe_chem_comp_rdkit_properties.NumRings
1\n_pdbe_chem_comp_rdkit_properties.NumAromaticRings
0\n_pdbe_chem_comp_rdkit_properties.NumAliphaticRings
1\n_pdbe_chem_comp_rdkit_properties.NumSaturatedRings
1\n_pdbe_chem_comp_rdkit_properties.NumHeterocycles
1\n_pdbe_chem_comp_rdkit_properties.NumAromaticHeterocycles
0\n_pdbe_chem_comp_rdkit_properties.NumSaturatedHeterocycles
1\n_pdbe_chem_comp_rdkit_properties.NumAliphaticHeterocycles
1\n_pdbe_chem_comp_rdkit_properties.NumSpiroAtoms
0\n_pdbe_chem_comp_rdkit_properties.NumBridgeheadAtoms

0\n_pdbe_chem_comp_rdkit_properties.NumAtomStereoCenters
6\n_pdbe_chem_comp_rdkit_properties.NumUnspecifiedAtomStereoCenters
0\n_pdbe_chem_comp_rdkit_properties.labuteASA
146.407\n_pdbe_chem_comp_rdkit_properties.tpsa
176.780\n_pdbe_chem_comp_rdkit_properties.CrippenClogP -
3.872\n_pdbe_chem_comp_rdkit_properties.CrippenMR
64.787\n_pdbe_chem_comp_rdkit_properties.chi0v
9.621\n_pdbe_chem_comp_rdkit_properties.chi1v
4.738\n_pdbe_chem_comp_rdkit_properties.chi2v
1.941\n_pdbe_chem_comp_rdkit_properties.chi3v
1.941\n_pdbe_chem_comp_rdkit_properties.chi4v
1.128\n_pdbe_chem_comp_rdkit_properties.chi0n
28.621\n_pdbe_chem_comp_rdkit_properties.chi1n
13.635\n_pdbe_chem_comp_rdkit_properties.chi2n
1.941\n_pdbe_chem_comp_rdkit_properties.chi3n
1.941\n_pdbe_chem_comp_rdkit_properties.chi4n
1.128\n_pdbe_chem_comp_rdkit_properties.hallKierAlpha -
1.300\n_pdbe_chem_comp_rdkit_properties.kappa1
4.600\n_pdbe_chem_comp_rdkit_properties.kappa2
6.642\n_pdbe_chem_comp_rdkit_properties.kappa3
3.872\n_pdbe_chem_comp_rdkit_properties.Phi
1.455\n#\nloop_\n_pdbe_chem_comp_external_mappings.comp_id\n_pdbe_chem_comp_external_mappings.source\n_pdbe_chem_comp_external_mappings.resource\n_pdbe_chem_comp_external_mappings.resource_id\nSIA UniChem ChEMBL CHEMBL1234621\nSIA UniChem DrugBank DB03721\nSIA UniChem ChEBI 49026\nSIA UniChem ZINC ZINC000004081651\nSIA UniChem fdasrs 04A90EXP8V\nSIA UniChem HMDB HMDB0000773\nSIA UniChem Nikkaji J614.853K\nSIA UniChem MetaboLights MTBLC49026\nSIA UniChem BRENDA 141715\nSIA UniChem BRENDA 233672\nSIA UniChem BRENDA 6105\nSIA UniChem BRENDA 84245\nSIA UniChem BRENDA 85625\nSIA UniChem 'Probes And Drugs' PD041137\nSIA UniChem PubChem 444885\nSIA UniChem eMolecules 474793\nSIA UniChem SureChEMBL SCHEMBL79085\nSIA UniChem 'PubChem TPHARMA' 14776495\nSIA UniChem 'PubChem TPHARMA' 15395566\n#\nloop_\n_pdbe_chem_comp_rdkit_conformer.comp_id\n_pdbe_chem_comp_rdkit_conformer.atom_id\n_pdbe_chem_comp_rdkit_conformer.Cartn_x_rdkit\n_pdbe_chem_comp_rdkit_conformer.Cartn_y_rdkit\n_pdbe_chem_comp_rdkit_conformer.Cartn_z_rdkit\n_pdbe_chem_comp_rdkit_conformer.rdkit_method\n_pdbe_chem_comp_rdkit_conformer.rdkit_ordinal\nSIA C1 -1.164 -2.811 1.058 ETKDGv3 1\nSIA C2 -1.666 -1.928 -0.070 ETKDGv3 2\nSIA C3 -2.639 -0.809 0.442 ETKDGv3 3\nSIA C4 -2.546 0.454 -0.428 ETKDGv3 4\nSIA C5 -1.079 0.960 -0.528 ETKDGv3 5\nSIA C6 -0.071 -0.181 -0.219 ETKDGv3 6\nSIA C7 1.341 0.133 -0.777 ETKDGv3 7\nSIA C8 2.378 -0.960 -0.400 ETKDGv3 8\nSIA C9 3.769 -0.651 -0.979 ETKDGv3 9\nSIA C10 -0.223 3.295 0.052 ETKDGv3 10\nSIA C11 0.035 4.333 1.094 ETKDGv3 11\nSIA N5 -0.868 2.066 0.414 ETKDGv3 12\nSIA O1A -0.936 -2.335 2.203 ETKDGv3 13\nSIA O1B -0.875 -4.149 0.806 ETKDGv3 14\nSIA O4 -3.104 0.224 -1.700 ETKDGv3 16\nSIA O6 -0.537 -1.404 -0.765 ETKDGv3 17\nSIA O7 1.298 0.320 -2.172 ETKDGv3 18\nSIA O8 2.484 -1.066 0.998 ETKDGv3 19\nSIA O9 4.246 0.587 -0.524 ETKDGv3 20\nSIA O10 0.157 3.497 -1.133 ETKDGv3 21\nSIA H32 -2.392 -0.510 1.484 ETKDGv3 22\nSIA H31 -3.691 -1.171 0.465 ETKDGv3 23\nSIA H4 -3.171 1.239 0.053 ETKDGv3 24\nSIA H5 -0.925 1.324 -1.568 ETKDGv3 25\nSIA H6 0.030 -0.270 0.887 ETKDGv3 26\nSIA H7 1.678

1.089 -0.320 ETKDGv3 27\nSIA H8 2.053 -1.935 -0.838 ETKDGv3 28\nSIA H92 3.719 -0.629 -2.088 ETKDGv3 29\nSIA H91 4.478 -1.464 -0.703 ETKDGv3 30\nSIA H111 0.843 3.984 1.770 ETKDGv3 31\nSIA H113 -0.888 4.508 1.686 ETKDGv3 32\nSIA H112 0.344 5.291 0.624 ETKDGv3 33\nSIA HN5 -1.113 1.913 1.419 ETKDGv3 34\nSIA HO1B -0.511 -4.749 1.536 ETKDGv3 35\nSIA HO4 -2.492 -0.372 -2.205 ETKDGv3 37\nSIA HO7 1.040 -0.545 -2.585 ETKDGv3 38\nSIA HO8 1.886 -1.804 1.285 ETKDGv3 39\nSIA HO9 4.592 0.441 0.395 ETKDGv3 40\n#\n"
}

Template 13. M3

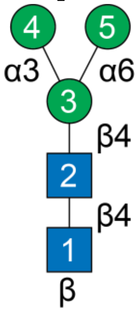


```
{
    "name": "m3",
    "modelSeeds": [
      1
    ],
    "sequences": [
      {
        "ligand": {
          "ccdCodes": [
            "NAG", "NAG", "BMA", "MAN", "MAN"
          ],
          "id": "NG"
        }
      }
    ],
    "dialect": "alphafold3",
    "version": 2,
    "bondedAtomPairs": [
    [["NG",1,"O4"],["NG",2,"C1"]],
    [["NG",2,"O4"],["NG",3,"C1"]],
    [["NG",3,"O3"],["NG",4,"C1"]],
    [["NG",3,"O6"],["NG",5,"C1"]]
    ]
  }
```

Template 14. M5

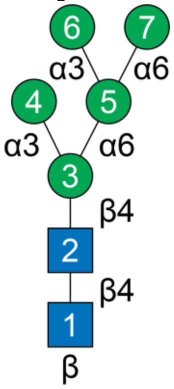


```
{
    "name": "m5",
    "modelSeeds": [
      1
    ],
    "sequences": [
      {
        "ligand": {
          "ccdCodes": [
            "NAG", "NAG", "BMA", "MAN", "MAN", "MAN", "MAN"
          ],
          "id": "NG"
        }
      }
    ],
    "dialect": "alphafold3",
    "version": 2,
    "bondedAtomPairs": [
    [["NG",1,"O4"],["NG",2,"C1"]],
    [["NG",2,"O4"],["NG",3,"C1"]],
    [["NG",3,"O3"],["NG",4,"C1"]],
    [["NG",3,"O6"],["NG",5,"C1"]],
    [["NG",5,"O3"],["NG",6,"C1"]],
    [["NG",5,"O6"],["NG",7,"C1"]]
    ]
  }
```

Template 15. M5F

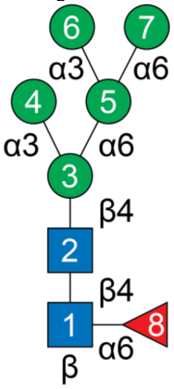


```
{
    "name": "m5f",
    "modelSeeds": [
      1
    ],
    "sequences": [
      {
        "ligand": {
          "ccdCodes": [
            "NAG", "NAG", "BMA", "MAN", "MAN", "MAN", "MAN", "FUC"
          ],
          "id": "NG"
        }
      }
    ],
    "dialect": "alphafold3",
    "version": 2,
    "bondedAtomPairs": [
    [["NG",1,"O4"],["NG",2,"C1"]],
    [["NG",2,"O4"],["NG",3,"C1"]],
    [["NG",3,"O3"],["NG",4,"C1"]],
    [["NG",3,"O6"],["NG",5,"C1"]],
    [["NG",5,"O3"],["NG",6,"C1"]],
    [["NG",5,"O6"],["NG",7,"C1"]],
    [["NG",1,"O6"],["NG",8,"C1"]]
    ]
  }
```
